# Chance and necessity in the genome evolution of endosymbiotic bacteria of insects

**DOI:** 10.1038/ismej.2017.18

**Published:** 2017-03-21

**Authors:** Beatriz Sabater-Muñoz, Christina Toft, David Alvarez-Ponce, Mario A Fares

**Affiliations:** 1 Instituto de Biología Molecular y Celular de Plantas, Consejo Superior de Investigaciones Científicas (CSIC)-Universidad Politécnica de Valencia (UPV), Valencia, Spain; 2 Systems Biology of Molecular Interactions and Regulation, Institute for Integrative Systems Biology (I2SysBio), Consejo Superior de Investigaciones Científicas (CSIC) - Universidad de Valencia (UV), Valencia, Spain; 3 Department of Genetics, Smurfit Institute of Genetics, University of Dublin, Trinity College, Dublin, Ireland; 4 Department of Genetics, Universidad de Valencia, Valencia, Spain; 5 Instituto de Agroquímica y Tecnología de los Alimentos, Consejo Superior de Investigaciones Científicas (CSIC), Valencia, Spain; 6 Department of Biology, University of Nevada, Reno, NV, USA

## Abstract

An open question in evolutionary biology is how does the selection–drift balance determine the fates of biological interactions. We searched for signatures of selection and drift in genomes of five endosymbiotic bacterial groups known to evolve under strong genetic drift. Although most genes in endosymbiotic bacteria showed evidence of relaxed purifying selection, many genes in these bacteria exhibited stronger selective constraints than their orthologs in free-living bacterial relatives. Remarkably, most of these highly constrained genes had no role in the host–symbiont interactions but were involved in either buffering the deleterious consequences of drift or other host-unrelated functions, suggesting that they have either acquired new roles or their role became more central in endosymbiotic bacteria. Experimental evolution of *Escherichia coli* under strong genetic drift revealed remarkable similarities in the mutational spectrum, genome reduction patterns and gene losses to endosymbiotic bacteria of insects. Interestingly, the transcriptome of the experimentally evolved lines showed a generalized deregulation of the genome that affected genes encoding proteins involved in mutational buffering, regulation and amino acid biosynthesis, patterns identical to those found in endosymbiotic bacteria. Our results indicate that drift has shaped endosymbiotic associations through a change in the functional landscape of bacterial genes and that the host had only a small role in such a shift.

## Introduction

The interactions between biological entities and their role in evolution has enthralled scientists for decades, but the causes and consequences of such interactions remain poorly characterized. Starring these biological interactions is the symbiosis of bacteria with plants and animals, considered an important engine of eukaryote ecological diversification (McFall-Ngai *et al.*, 2013; Archibald, 2014).

The mutualistic symbiosis between bacteria and insects is one of the most widespread associations in nature. The obligate symbiosis between the pea aphid (*Acyrthosiphon pisum*) and its bacterial endosymbiont *Buchnera aphidicola* (Shigenobu *et al.*, 2000) provides a good example of such mutualistic associations. These bacteria are restricted to highly specialized host cells, are maternally inherited (Koga *et al.*, 2012) and exhibit specific molecular trafficking with the host (Nakabachi *et al.*, 2014; Price *et al.*, 2014). The genomes of the aphid and the bacterium have been co-transmitted for millions of generations and each seems to influence its partner: the bacterium provides nutrients to the host (Shigenobu *et al.*, 2000; Tamas *et al.*, 2002; van Ham *et al.*, 2003) and allows it to thrive on an otherwise unbalanced diet (Law and Lewis, 1983), while the host houses and transmits the bacterium under benign environmental conditions (Hansen and Moran, 2011; Macdonald *et al.*, 2012). This association, however stable, is constrained by the small effective population sizes and asexual reproduction of endosymbiotic bacteria (Moran, 1996; Mira *et al.*, 2001; Nilsson *et al.*, 2005) and the inherent nucleotide deletion bias of their genomes (Mira *et al.*, 2001; Kuo and Ochman, 2009), leading to their genome size reduction (McCutcheon and Moran, 2012). The population bottlenecks during bacterial transmission to the host offspring makes natural selection less efficient, which combined with the lack of recombination and repair genes has led to an increase in the mutational load of endosymbiotic genomes (Moran, 1996; Fares *et al.*, 2002b; van Ham *et al.*, 2003; Wernegreen, 2011). Nonetheless, signatures of purifying selection (Toft and Fares, 2008) and positive selection (Fares *et al.*, 2002a) have been identified in endosymbiotic genes not directly linked to the purpose of providing the host with nutrients.

Patterns of selection in endosymbiotic bacteria may result from different levels of selection, including selection imposed by the host and that emerging in a symbiotic context but being independent from the host. Teasing apart these levels of selection remains a major challenge. In this study, we investigate whether the evolutionary landscape of endosymbiotic genes has changed as a result of genetic drift or selection imposed by the diet requirements of the host. To address this question, here we investigate the selective patterns of five major endosymbiotic bacterial groups and characterize the genome and transcriptome changes of the bacterium *Escherichia coli* K12 evolving experimentally under population dynamics that emulate those of maternally inherited endosymbiotic bacteria.

## Materials and methods

### Endosymbiotic and free-living bacterial genomes

Endosymbiotic bacterial genomes and those of their free-living relatives were downloaded from the SymbioGenomesDB database (Reyes-Prieto *et al.*, 2015). We used endosymbiotic genomes of: aphids (*B. aphidicola* strain JF98, from *A. pisum*; *B. aphidicola* strain Sg, from *Schizaphis graminum*), carpenter ants (*Candidatus* Blochmannia floridanus and *Ca.* Blochmannia pennsylvanicus strain BPEN), tsetse flies (*Wigglesworthia glossinidia*, from *Glossina brevipalpis*; and *W. morsitans* from *Glossina morsitans morsitans*), sharpshooters (*Candidatus* Baumannia cicadellinicola strains HC and BGSS) and cockroaches (*Blattabacterium* strain Bge, from *Blatella germanica*; and strain BPLAN, from *Periplaneta americana*). Pairs of endosymbiotic genomes used in this study were similar in size. *B. aphidicola* strain Ak from *Acyrthosiphon kondoi* was used to ascertain the lack of saturation of synonymous sites. We used *E. coli* strain K12 substrain MG1655 and *Salmonella enterica* serovar Typhi (*S. typhi*) as free-living relatives of gamma-proteobacteria endosymbionts. *Flavobacterium branchiophilum* and *F. psychrophilum* were used as free-living relatives of Bacteroidetes endosymbionts.

### Analysis of selective constraints

**Figure 1:**
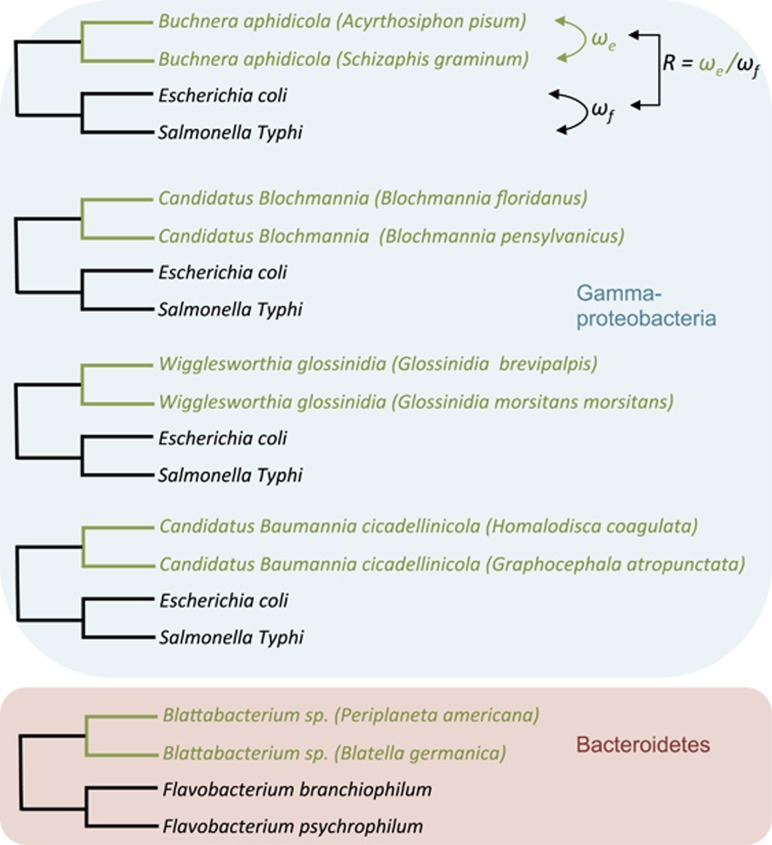
Determining the relative strength of selective constraints in endosymbiotic bacterial genomes. We calculated the ratio between non-synonymous nucleotide substitutions per non-synonymous site (*d*_N_) and nucleotide substitutions per synonymous site (*d*_S_) (*ω*=*d*_N_/*d*_S_) to estimate the strength of selection on protein-coding genes. This ratio was estimated between pairs of endosymbiotic genomes within each of the five endosymbiotic systems (*ω*_e_) and between pairs of relative free-living bacteria (*ω*_f_). We used as endosymbiotic gamma-proteobacteria: (1) *Buchnera aphidicola* (strains from aphids *Acyrthosiphon pisum* and from *Schizaphis graminum*), (2) *Candidatus* Blochmannia (Blochmannia floridanus and Blochmannia pennsylvanicus), (3) *Wigglesworthia* sp. (*Wigglesworthia glossinidia* and *Wigglesworthia morsitans*) and (4) *Candidatus* Baumannia cicadellinicola (*Homalodisca coagulata and Graphocephala atropunctata*). We used as endosymbiotic bacteria from the Bacteroidetes group *Blattabacterium* sp. (*Blattabacterium* from *Blatella germanica* and from *Periplaneta americana*). We used *Escherichia coli* and *Salmonella enterica* as the external free-living bacteria pair relatives of gamma-proteobacteria and *Flavobacterium branchiophilum* and *F. psychrophilum* as free-living bacterial relatives of Bacteroidetes endosymbionts. We analyzed how the selective constraints on genes varied when comparing endosymbiotic bacteria (in green) with their free-living relatives (in black). The selective constraints on endosymbiotic bacterial genomes relative to their free-living bacterial relatives was calculated as *R*=*ω*_e_/*ω*_f_.

For each pair of bacterial genomes, we built pairwise sequence alignments for orthologous genes. This resulted in seven groups of alignments corresponding to five pairs of endosymbiotic bacteria (namely *Buchnera*, *Ca.* Blochmannia, *Wigglesworthia*, *Ca.* Baumannia and *Blattabacterium*) and two pairs of free-living bacterial genomes (*E. coli*/*S. typhi* and *Flavobacterium*). We used MAFFT version 7 (Katoh and Standley, 2013) to align amino acid sequences and then used these alignments to guide the alignment of nucleotide sequences. In total, we obtained reliable multiple sequence alignments for 483, 462, 514, 448 and 348 protein-coding genes in *Buchnera*, *Ca.* Blochmannia, *Wigglesworthia*, *Ca*. Baumannia and *Blattabacterium* sp., respectively. We estimated the strength of selection by calculating the non-synonymous-to-synonymous divergence ratio (*ω*=*d*_N_/*d*_S_) using yn00 implemented in PAML version 4.7 (Yang, 2007) (Figure 1). The parameter *ω* is an indicator of selective pressure, with values of *ω*=1, *ω*<1 and *ω*>1, indicating neutral evolution, purifying selection and positive selection, respectively. The closer *ω* is to 0, the stronger is purifying selection in purging deleterious nonsynonymous mutations. Conversely, the closer *ω* is to 1, the weaker is selection in eliminating deleterious mutations. To compare the selective pressures acting in endosymbiotic bacteria vs those acting in free-living bacteria, we used two strains within each of the endosymbiotic bacterial groups and obtained two *ω* estimates for each of the genes: one *ω* was estimated by comparing the gene sequences of the endosymbiotic strains (we called these *ω*_e_) and another was estimated from the comparison of the sequences of their free-living bacterial strain relatives (*ω*_f_) (Figure 1). Then, we compared *ω*_e_ with *ω*_f_ using the ratio *R* (*R*=*ω*_e_/*ω*_f_). In general, *R*>1 is expected because the efficacy of selection is higher in free-living bacteria (Kuo *et al.*, 2009). Therefore, *R* is a measure of the relative constraints on endosymbiotic bacterial genes, with *R*=1, *R*<1 and *R*>1 indicating equal constraints, stronger constraints and weaker constraints in endosymbiotic than in free-living bacteria, respectively.

### Non-parametric bootstrapping

To test the significance of the convergent selective constraints for genes among endosymbiotic bacteria, we performed a test based on non-parametric bootstrap. Briefly, we randomly selected a set of genes from each symbiotic bacterial group genome alignment generated to determine the gene selective constraint. In total, we generated five lists of constrained genes, one for each bacterial group. Then we asked how many genes were found convergently in one, two, three or four of the lists. We repeated this procedure 10^5^ times and drew a distribution that we used as the null distribution from which we calculated the probability of the observed convergences in the real data sets.

### Experimental evolution of bacteria under genetic drift

**Figure 2:**
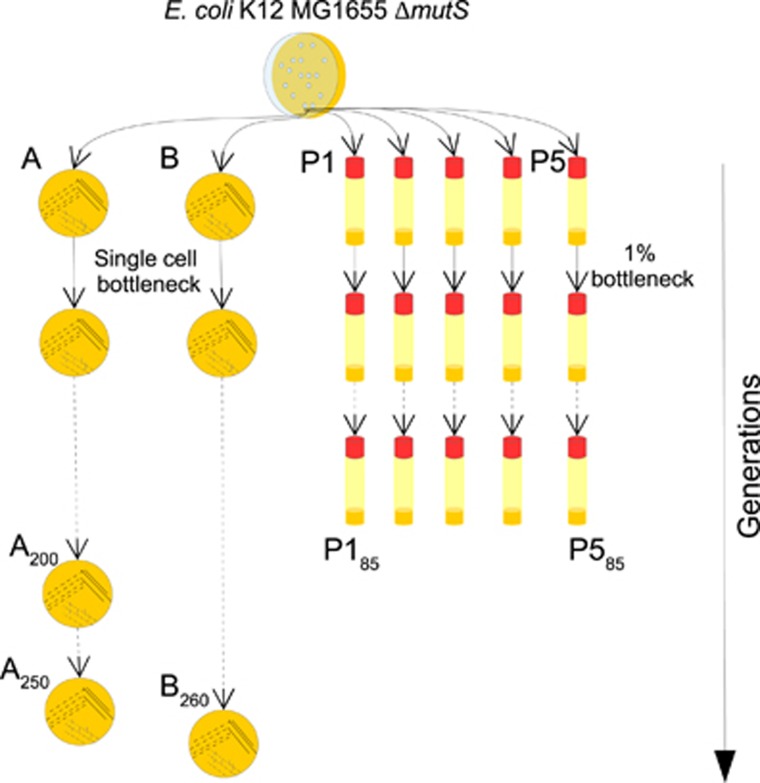
Experimental evolution of *Escherichia coli* under two population dynamics. We evolved two independent clonal lines (A and B) and five population lines (P1–P5) derived from a single ancestral population of a hypermutagenic strain of *E. coli* lacking the repair gene *mutS* under strong population bottlenecks and rich growth medium. Evolution proceeded with daily passaging a single colony to a new plate for 250 days in line A and 260 days in line B or by daily passaging 1% (100 μl) of populations P1–P5 to fresh LB broth (10 ml). Genomes were sequenced at the end of the evolution experiment.

The long-term evolution experiment under strong genetic drift of lines A and B, each of which represent lines of *E. coli* K12 strain MG1655, is described elsewhere (Alvarez-Ponce *et al.*, 2016). Additionally, from the same ancestral colony, another five independent experimental evolution lines were established in liquid Luria Broth media (LB, Conda laboratory, Madrid, Spain). Each population lineage was serially passaged each 24 h by diluting 1:100 into 10 ml of fresh LB medium in 50 ml Falcon tubes (Corning, Mexico DF, Mexico). Population lineages were passaged 85 times (an estimated 561 generations, 6.6 generations per passage) (Figure 2).

### Whole-genome sequencing

Genome sequences for lines A and B were obtained from our recent work (Alvarez-Ponce *et al.*, 2016). For the evolution experiment in liquid media, we performed paired-end Illumina whole-genome sequencing (TrueSeq DNA PCR-free HT, Illumina Inc., San Diego, CA, USA) at final time (561 generations). Sequencing was performed in a MiSeq benchtop sequencer (Illumina Inc.), using a 2 × 150 bp with 300 cycles configuration. Libraries and sequencing were performed at ValGenetics SL sequencing facility (Valencia, Spain). Single-nucleotide polymorphisms (SNPs) and indels were identified with the breseq v 0.24rc4 (version 4) pipeline (Deatherage and Barrick, 2014) using our *E. coli* parental genome reported previously (Sabater-Muñoz *et al.*, 2015). We identified radical and non-radical amino-acid substitutions in the evolved populations by classifying substitutions into two categories: (a) radical substitutions: are those changes that involve a change in the charge, polarity or polarity and volume of the original amino acids, and (b) non-radical substitutions: are those replacements that occur between physically and chemically equivalent amino acids. We used the classification of amino acids according to their physical–chemical properties following a previous study (Wernegreen, 2011).

### RNA sequencing and analysis

Triplicate cultures were set for the ancestral population and clonal lines at time points 200 and 250 from freshly recovered glycerol stocks in LB at 37 °C with continuous shaking (220 r.p.m.) for ~4 h (until achieving an OD_600_≃0.6). Cultures were stopped with RNAprotect bacterial reagent (Qiagen, Valencia, CA, USA). Total RNA was extracted from 1.5 ml of stopped–harvested cells using the RNeasy Mini Kit (Qiagen) following the manufacturer's protocol. RNA (integrity number >8) was depleted of ribosomal RNA using the Ribo-Zero rRNA Gram-Negative Removal Kit MRZGN126 (Epicentre-Illumina, Madison, WI, USA). Indexed RNAseq libraries were constructed using strand-specific cDNA synthesis (TruSeq RNA Library Preparation Kit, Illumina), pooled in equimolar concentration and subjected to single-end 50 bp Illumina sequencing in an Illumina HiSeq2000 platform using a 2 × 100 cycles configuration. RNA ribosomal depletion, library construction and sequencing were carried out at LifeSequencing S.L. (Valencia, Spain).

Raw sequences were processed with the RobiNA (Lohse *et al.*, 2012) and Rockhopper v.2.0.3 (McClure *et al.*, 2013) software to determine differentially expressed genes among time points. Briefly, fastq files were subjected to quality trimming, removal of short sequences (<20 pb) and mapped against the *E. coli* K-12 str. MG1655 NCBI reference genome NC_000913 using Bowtie2 with 2 bp mismatch option. After gene counts, edgeR (Robinson *et al.*, 2010) or DEseq (Anders and Huber, 2010) were used to identify significant differential expression once corrected for multiple testing using the Benjamini–Yekutieli method (Benjamini and Yekutieli, 2005). Each list of differentially expressed genes was subjected to gene ontology (GO) term classification using the PANTHER classification System (http://www.pantherdb.org/), including a GO enrichment analysis, with a *P*-value cutoff of <0.05. A semantic similarity score, simRel (Schlicker *et al.*, 2006), was used to summarize and remove redundant GO terms in each list, as implemented in the REVIGO software with medium (0.7) allowed similarity (Supek *et al.*, 2011).

Files containing reads for the nine RNA libraries have been deposited in the Sequence Read Archive (http:/ncbi.nlm.nih.gov/sra) under accession number SRP074670.

## Results

### Signatures of drift and selection in endosymbiotic bacteria of insects

Endosymbiotic bacterial genomes evolve under more relaxed selective constraints when compared with their closest free-living relatives (Moran, 1996; Wernegreen, 2002; Moran *et al.*, 2008; Wernegreen, 2011). However, selection has been proposed to be stronger upon endosymbiotic bacterial genes that are key in producing metabolites for the insect host than on other genes unrelated to the metabolism of the host (Clark *et al.*, 2001; Hansen and Moran, 2014; Price *et al.*, 2014; Bennett and Moran, 2015). To determine the strength of selection on endosymbiotic bacterial genes relative to their free-living bacterial orthologs, we estimated the non-synonymous-to-synonymous rates ratio (*ω*=*d*_N_/*d*_S_) for genes of five independent groups of endosymbiotic bacteria (*ω*_e_) and two independent groups of free-living bacteria (*ω*_f_) (see Material and methods section; Figure 1). To compare *ω*_e_ with *ω*_f_, we calculated *R* (*R*=*ω*_e_/*ω*_f_).

**Figure 3:**
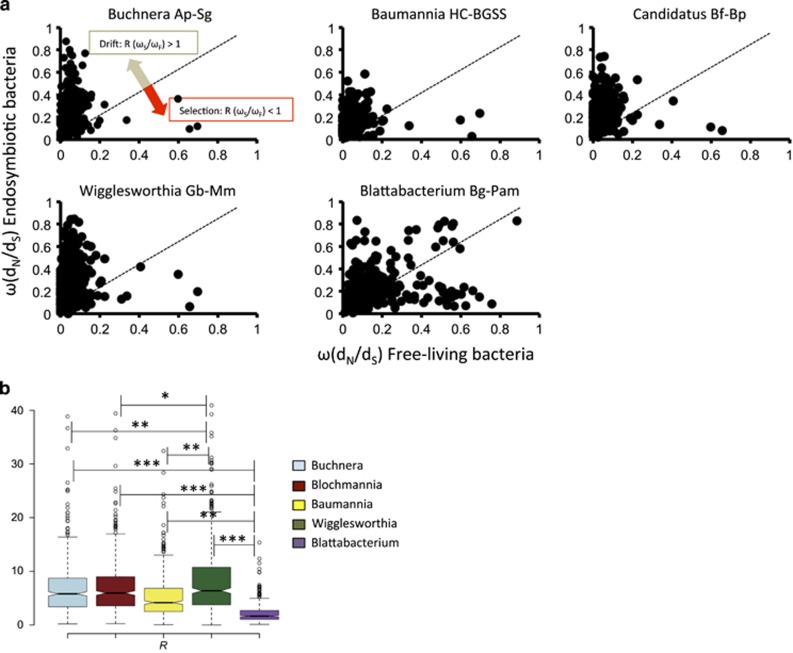
Signatures of natural selection and genetic drift in endosymbiotic bacteria of insects. The strength of selection was determined as the ratio between non-synonymous nucleotide substitutions per non-synonymous site (*d*_N_) and synonymous nucleotide substitutions per synonymous site (*d*_S_) (*ω*=*d*_N_/*d*_S_) for each genome pair. (**a**) To determine the relative strength of selection on endosymbiotic genomes, we divided the *ω* of each symbiotic gene (*ω*_e_) by that of its ortholog in its free-living bacterial relatives (*ω*_f_) and compared this ratio (*R*) with 1. Values of *R*>1 imply that *ω*_e_>*ω*_f_, hence endosymbiotic genes evolved under relaxed selective constrains or under increased genetic drift. Conversely, *R*<1 implies stronger constrains on endosymbiotic genes than on their free-living bacterial orthologs. (**b**) The relative efficiency of natural selection, or genetic drift, for each of the endosymbiotic genomes of this study was compared. Differences were tested using Wilcoxon's rank test with significant values being indicated with *P<0.05, **P<0.01 and ***P<10^−6^.

Most of the genes in endosymbiotic bacterial genomes (Table 1) exhibited relaxed constraints compared with their free-living relatives (*R*>1) (Figure 3a). Comparison of endosymbiotic genomes (*Buchnera A. pisum* and *A. kondoi*) that were phylogenetically closer did not change the results (Supplementary Table S1), suggesting that saturation of synonymous sites was not affecting our observations. The more relaxed constraints in endosymbiotic than their free-living bacterial relatives is consistent with reduced efficacy of natural selection in endosymbionts. However, a substantial number of genes in endosymbiotic bacteria (Table 1) showed stronger selective constraints than in their free-living relatives (*R*<1). The endosymbionts *Blattabacterium* sp. showed the lowest median *R*-value (median *R*=1.65), followed by *Ca.* Baumannia (median *R*=4.17), *Buchnera* (median *R*=5.81), *Ca.* Blochmannia (median *R*=5.95) and *Wigglesworthia* (median *R*=6.39) (Figure 3b). *Blattabacterium* sp. showed significantly stronger relative constraints than the *Buchnera*–*Ca.* Blochmannia group (Wilcoxon's rank test: *P*<2.2 × 10^−16^), *Ca.* Baumannia (Wilcoxon's rank test: *P*<2.2 × 10^−16^) and the *Wigglesworthia* endosymbiont (Wilcoxon's rank test: *P*<2.2 × 10^−16^). *Wigglesworthia* endosymbionts showed more relaxed constraints than *Ca.* Baumannia (Wilcoxon's rank test: *P*=3.16 × 10^−15^), *Buchnera* (Wilcoxon's rank test: *P*=0.003) and *Ca.* Blochmannia (Wilcoxon's rank test: *P*=0.02). *Buchnera* and *Ca.* Blochmannia showed more relaxed constraints than *Ca.* Baumannia (Wilcoxon's rank test: *P*=1.99 × 10^−7^), but there was no difference in the relative constraints between *Ca.* Blochmannia and *Buchnera* (Wilcoxon's rank test: *P*=0.37). The stronger constraints in *Blattabacetrium* sp. stem from a greater proportion of this endosymbiont's genes being involved in the urea metabolism of the host (Gonzalez-Domenech *et al.*, 2012; Patino-Navarrete *et al.*, 2013), with these genes evolving under strong constraints. All together, these results indicate shifts in the selective constraints and, perhaps, changes in the encoded functions, of some bacterial genes in endosymbionts compared with free-living bacteria.

### Convergent host-independent evolution in endosymbiotic bacteria of insects

**Figure 4:**
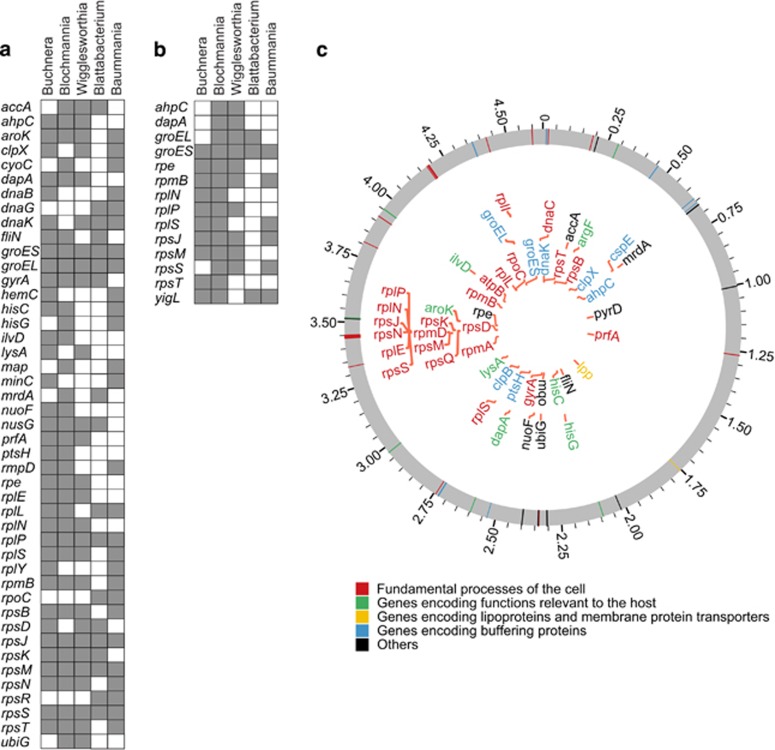
Endosymbiotic bacteria of insects converge in their selective constraints at genes that are unrelated to the insect host. We analyzed the distribution of the selective constraints among endosymbiotic genes and studied the convergences among the five independent endosymbiotic groups. We studied two sets of genes, (**a**) one in which the ratio between the symbiotic and free-living bacterial non-synonymous-to-synonymous rates ratio (*R*=*ω*_e_/*ω*_f_) is *R*<2, hence this set includes genes with strong selective constraints (*R*<1) and slightly relaxed constraints (1<*R*<2), and (**b**) a set of strongly constrained symbiotic genes when compared with their free-living bacterial orthologs *R*<1. Gray-colored squares in the matrix indicate genes convergently constrained between two or more endosymbiotic groups; white squares are genomes in which such genes are under relaxed constraints. (**c**) Convergently constrained genes in endosymbiotic genomes. These genes are color-coded according to their functional classification using GO terms. Only seven genes were relevant to the metabolism of the bacterial host (green-colored genes).

A number of endosymbiotic bacterial genes evolved under relatively moderate selective constraints (that is, 1<*R*<2) or under stronger selective constraints than in their free-living bacterial relatives (*R*<1) (Table 1 and Supplementary Table S1). Six of the genes were highly constrained in all the 5 endosymbiotic bacterial groups, 11 in four, 17 in three and 17 in two (Figure 4a). The number of genes constrained convergently in different endosymbiotic bacteria was significantly higher than expected (Material and methods section) (Randomization test: *P*<10^−6^, *P*<10^−5^ and *P*=0.001 for convergences in five, four and three endosymbionts, respectively). The set of strongly constrained genes (*R*<1) (Supplementary Table S1) included one, five and eight genes found in five, four and three independent endosymbiotic bacteria (Figure 4b), respectively, with these convergences being significant (*P*<10^−3^, *P*<10^−3^ and *P*<10^−3^, respectively). Genes constrained in endosymbiotic bacteria encoded chaperones and proteins involved in transcription and translation (Figure 4c).

Among all the constrained genes, 7 (*lysA*, argF, *hisC*, *hisG*, *ilvD*, *aroK* and *dapA*) were involved in the synthesis of amino acids, perhaps important to supplementing host diets, while at least 35 of them were host unrelated and linked to translation (ribosomal-coding genes *rplP*, *rplN*, *rpsJ*, *rpsN*, *rplE*, *rpsS*, *rpsK*, *rpsM*, *rpsQ* and *rpmD*; Hypergeometric test with Bonferroni's correction: *P*=1.70 × 10^−12^) and protein-binding or stress-related functions (chaperones and chaperonins *clpX*, *dnaK*, *groES*, *groEL* and *ahpC*; enrichment of the category ‘binding': *P*=1.53 × 10^−8^) (Figure 4c). Some of the constrained genes (*dnaK*, *groES* and *groEL*) have been previously reported to buffer the effects of deleterious mutations (Fares *et al.*, 2002b; Bogumil and Dagan, 2010; Williams and Fares, 2010; Sabater-Muñoz *et al.*, 2015; Aguilar-Rodriguez *et al.*, 2016; Kadibalban *et al.*, 2016) (Figure 4c). The strong selective constraints in genes overlapping among endosymbionts from hosts with different diet requirements support host-independent, however contextual to endosymbiosis, selective constraints of such genes.

**Figure 5:**
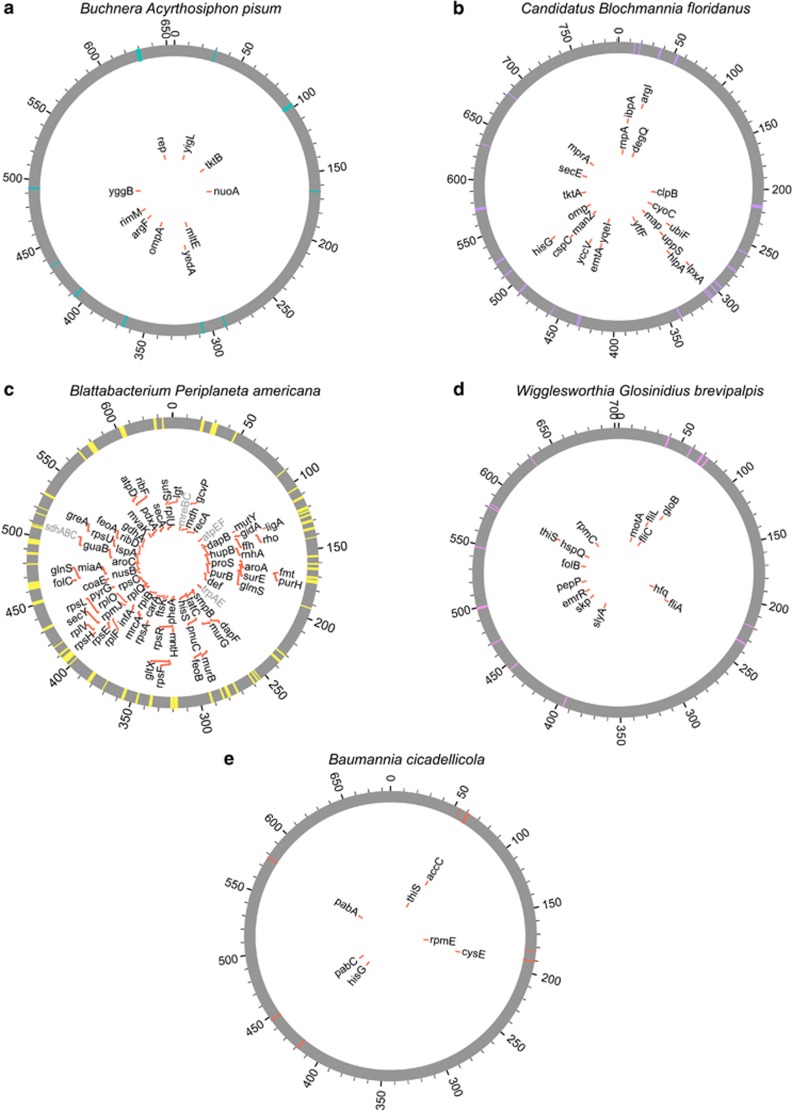
Genome distribution of genes with strong selective constraints in endosymbiotic bacteria. We identified genes with strong constraints in each of the five endosymbiotic bacterial lineages: (**a**) *Buchera aphidicola* (from *Acyrthosiphon pisum*); (**b**) *Candidatus* Blochmannia (Blochmannia floridanus); (**c**) *Blattabacterium* from *Periplaneta americana*; (**d**) *Wigglesworthia glossinidia*; and (**e**) *Ca.* Baumannia cicadellinicola. Genes indicated in the circle-representation of the endosymbiotic genomes are those that were specifically identified in that endosymbiotic genome and not the others from the same group. These genes are mostly related to the metabolism of the bacterium that interacts with the metabolism of the insect host.

Some of the genes specifically constrained (*R*<1) in a single endosymbiont lineage but not in others were associated with bacterial pathways that are key in supplementing the metabolism of the insect host (Figures 5a–e). This includes genes from *Buchnera* involved in amino-acid metabolism (*tktB*, *mltE*, *ompA*, *argF*) and export of amino acids to the host (*yedA*, *yggB*) (Figure 5a) or genes involved in nitrogen metabolism in the symbionts *Blochmannia*, *Wigglesworthia* and *Blattabacterium* (Figures 5b–d). Other symbiont-specific constrained genes were, nevertheless, host unrelated, being involved in stress response in the *Blochmannia* (*clpB*, *yccV*, *cspC*, *ibpA*) and *Wigglesworthia* (*hfq*, *skp*, *hspQ*) endosymbionts or the flagellum biosynthesis pathway in *Wigglesworthia* endosymbiont (*fliC*, *fliL*, *fliA*).

### The mutational spectrum of experimentally evolving bacteria under drift

**Figure 6:**
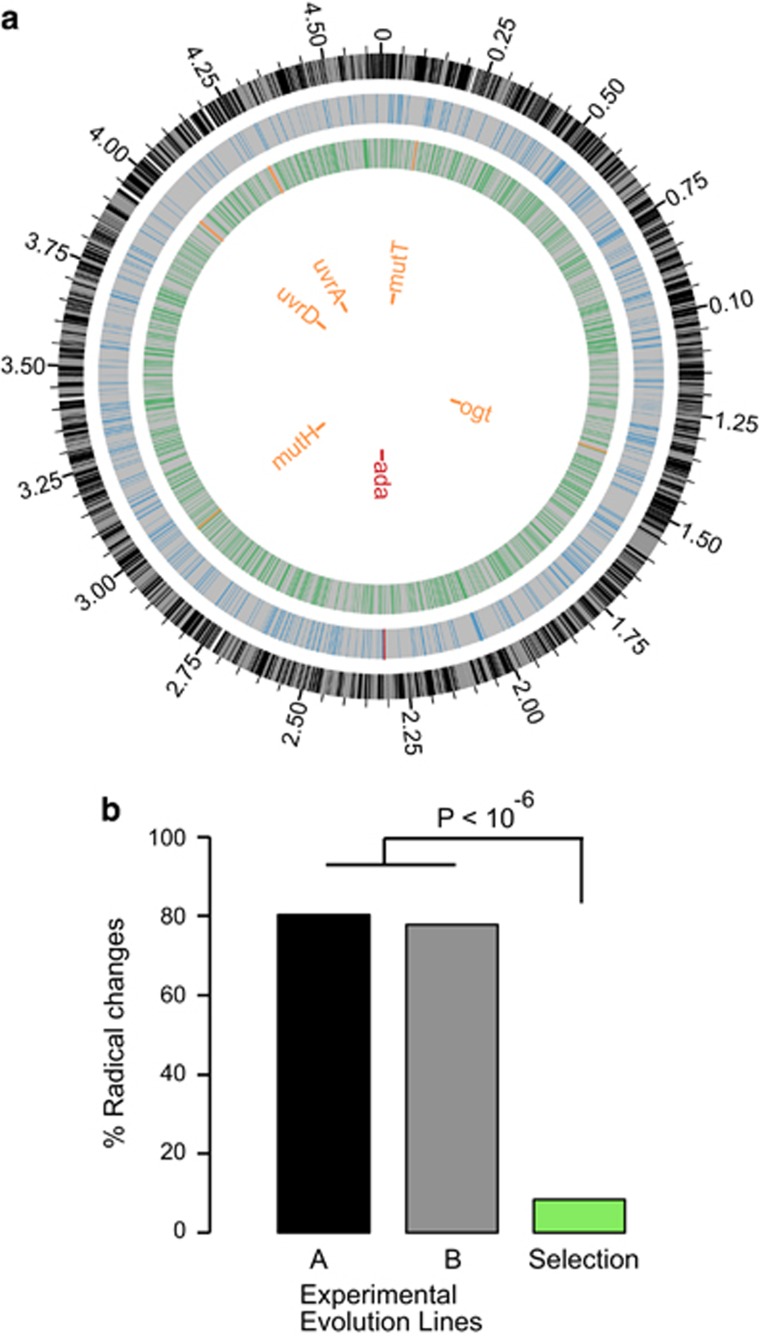
Experimental evolution of *Escherichia coli* reveals the contribution of the selection–drift balance to the evolution of endosymbiotic genes. (**a**) Distribution of mutations of lines A and B at the end of the evolution experiment. The outermost circle refers to the genome of *E. coli* K12 MG1655, used as reference for mapping the mutations in the evolution experiment. The blue circle refers to line A, whereas the green circle represents line B. Genes are indicated with vertical lines to each of the circles. Mutated repair genes for line A (red) and line B (orange) are indicated. (**b**) Proportion of radical mutations during the evolution experiment of *E. coli* under strong genetic drift (black and grey columns) and mild genetic drift (green column). Roughly 80% of the mutations in lines A and B were radical amino-acid changes, such that the original amino acid underwent a replacement to an amino acid with different charge, polarity or volume and polarity. The population under milder drift exhibited a significantly lower proportion of its amino-acid replacements being radical (about 8%).

To determine how genetic drift alone affects the genome evolution of bacteria, we examined the mutational dynamics of *E. coli* bottlenecked populations through an evolution experiment conducted in our laboratory (Alvarez-Ponce *et al.*, 2016). The fact that endosymbiotic bacteria of insects are uncultivable implies that our experimental setup does not emulate the metabolic flux from the host to the endosymbiotic bacteria in nature but allows emulating the population dynamics of endosymbiotic bacteria. Genome sequencing after 5500–5750 generations of evolution and comparison of these genomes with the ancestral population identified 723 and 1268 mutations in lines A and B, respectively (Supplementary Tables S2 and S3). The differences in the mutational profiles found between lines A and B (Supplementary Table S4) were likely due to a greater number of repair genes affected by mutations in line B (including genes *ogt*, *mutH*, *uvrD*, *uvrA*, *mutT*) than in line A (*ada*) (Figure 6a). Importantly, repair genes that mutated in line B are absent from the genomes of the primary symbiotic bacteria of aphids (Dale *et al.*, 2003; Moran *et al.*, 2008; Moran *et al.*, 2009).

To compare the spectrum of mutations of our experimentally evolved lines to that of endosymbiotic bacteria of insects, we classified proteins mutated with non-synonymous SNPs in line B, which presented the greatest number of mutations, into the different GO categories (Carbon *et al.*, 2009) as provided by AmiGO2 (http://amigo.geneontology.org). Enzymes (catalytic activities), transmembrane transporters and receptors and binding proteins (Supplementary Table S5), most of which are involved in the bacterial–environment interface and likely dispensable in a rich stable environment, were enriched for genes that mutated in our evolution experiment. Importantly, genes that are present in *Buchnera* were under-represented among the set of mutated genes in line A (39 out of the 408 mutated genes, 9.55%, Fisher's exact test: odds ratio F=0.27, *P*<2.2 × 10^−16^) and line B (73 out of the 820 mutated genes, 8.9%, F=0.47, *P*=5.17 × 10^−10^).

Three hundred and twenty-eight (80.39%) of the mutations of line A and 638 (77.80%) in line B were radical in terms of amino-acid charge, polarity or polarity and volume (Figure 6b). In a different evolution experiment of *E. coli* populations under moderate genetic drift for 85 passages (that is, ~6.6 generations per passage; Figure 2), we found 298 polymorphisms, of which 147 were non-synonymous (Supplementary Table S6), and of these, only 85 (57%) were radical. Among the mutations identified in 100% of the reads (that is, mutations fixed in the population) (Supplementary Table S7), only 12 were non-synonymous mutations, of which only 1 mutation (Asp613 to Gly in the gene *bcsB*) was radical (8.33%). This population thus exhibited fewer radical SNPs than lines A and B (Figure 6b, Fisher's exact test: F=44.61, *P*=2.96 × 10^−7^ for line A, and F=38.37, *P*=7.99 × 10^−7^ for line B), consistent with its larger effective population size, and hence its higher efficacy of natural selection in removing radical amino acid mutations. Therefore, this experiment suggests that, as in endosymbiotic bacteria of insects, most of the mutations accumulated in lines A and B were deleterious and were accumulated owing to the strong genetic drift imposed during their evolution.

### Genome reduction in experimentally evolved bacterial populations

The estimated rate of nucleotide loss in *B. aphidicola* endosymbionts is 2.9 × 10^−8^ nucleotide losses per site per year (Gomez-Valero *et al.*, 2004). The number of endosymbiotic bacterial generations per year varies between 15 and 50 (Humphreys and Douglas, 1997). This means that the rate of nucleotide loss in *Buchnera* ranges between 1.9 × 10^−9^ and 5.8 × 10^−10^ nucleotide losses per site per generation. In our experimentally evolved lines, we found a total of 41 and 62 events of gene deletion affecting 38 355 and 3106 base pairs in lines A and B, respectively. The ancestral *E. coli* strain used in our evolution experiments has a genome size of 4.64 Mb (Sabater-Muñoz *et al.*, 2015), which, taking into account the number of deleted base pairs, yields rates of 1.58 × 10^−6^ and 1.28 × 10^−7^ nucleotides deleted per site per generation for lines A and B, respectively. These rates are 831 and 67 times greater, respectively, than the fastest loss rate reported for *Buchnera* (Gomez-Valero *et al.*, 2004; Moran *et al.*, 2009). Moreover, we identified the deletion of a 35 590 bp region in line A, encompassing 42 genes located in the *E. coli* chromosome between the pseudogene *yoeG* and the IS5 transposase and transactivator gene *wbbL*. As most deletion events only affected a few nucleotides, genome reduction through evolution by genetic drift is likely a gradual process with punctuated events of big deletions, as has also been demonstrated in *B. aphidicola* (Gomez-Valero *et al.*, 2007). In total, we found more insertion events (83 insertions) than deletion events (41 deletions) in line A (binomial test: *P*=2 × 10^−4^), despite a greater number of deleted nucleotides than inserted nucleotides (binomial test: *P*<2.2 × 10^−16^). By contrast, in line B we found equivalent deletion events (62 deletions) and insertion events (56 insertions) (binomial test: *P*=0.32) but a greater number of nucleotides deleted than inserted (binomial test: *P*<2.2 × 10^−22^).

There were differences in the rates of nucleotide loss and gain between protein-coding and intergenic regions in the experimentally evolved lines. The protein-coding regions of line A exhibited a total of 38 355 lost nucleotides vs 62 inserted nucleotides, while intergenic regions exhibited 15 nucleotides deleted and 35 inserted. Therefore, while coding regions were shrinking (binomial test: *P*<2.2 × 10^−22^), intergenic regions were expanding in size (binomial test: *P*=0.007). Line B exhibited a similar pattern, with protein-coding regions bearing more deletions (3085 nucleotides) than insertions (36 nucleotides); hence these regions were shrinking (binomial test: *P*<2.2 × 10^−22^), while intergenic regions did not reveal significant differences between insertions (24 insertions) and deletions (21 deletions) (binomial test: *P*=0.76). The difference in the gene deletion–insertion pattern between coding and intergenic regions was significant for lines A (Fisher's exact test: F=1370.99, *P*<2.2 × 10^−16^) and B (F=96.69, *P*<2.2 × 10^−16^).

### Regulatory evolution of experimentally evolved bacteria

We compared the transcriptome of *E. coli* from line A at different times of the evolution experiment with that of its ancestral, non-evolved line. We observed a genome-wide deregulation along the evolution experiment, affecting >65% of all the genes (Supplementary Table S8). We identified 1303 overexpressed genes and 1251 repressed genes at 200 passages and 1171 overexpressed and 1097 repressed genes at 250 passages. An additional 200 overexpressed genes and 181 repressed genes were observed between the time points 200 and 250. Therefore, during the first 200 single-colony passages, gene regulation was altered at an average rate of 12.8 genes per passage (that is, 2554 genes were deregulated during the 200 passages of evolution: rate of deregulation=2554/200=12.8), while this rate was 7.6 between 200 and 250 (381 deregulated genes/50 passages). Therefore, most regulatory changes took place at the beginning of the evolution experiment.

Genes that became upregulated during the evolution experiment participated in metabolic and regulation processes, while downregulated genes were enriched for cell localization, cellular components and biogenesis processes. Taking the category of molecular functions, upregulated genes were mainly classified in the categories of regulation of translation, electron carrier activity, nucleic acid binding, protein-binding transcription, catalytic and receptor activities and antioxidant activities (Supplementary Figure S1a). Downregulated genes on the other hand were mainly involved in transporter activity (Supplementary Figure S1b). Interestingly, in the transcriptomic analyses at different time points, the enrichment of regulatory activities changed with genes being downregulated during the first 200 passages and upregulated from 200 to 250 passages.

**Figure 7:**
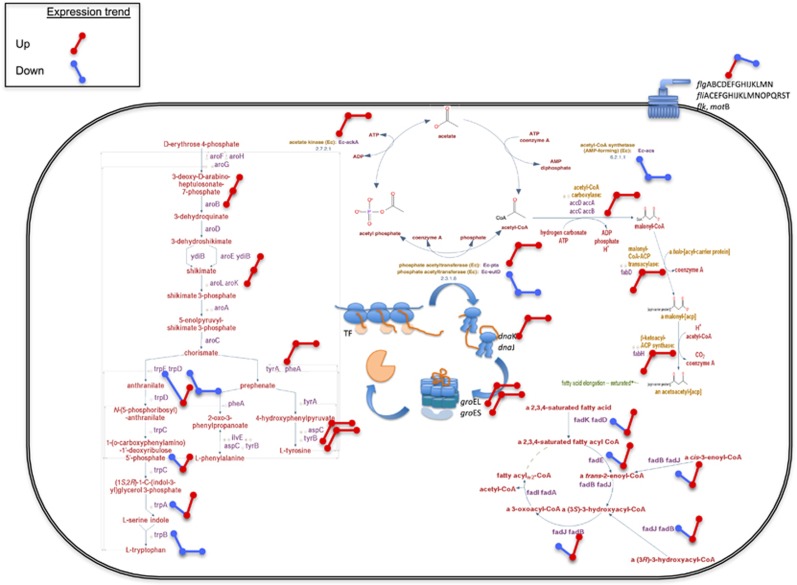
Transcriptional changes due to genetic drift in experimentally evolved *E. coli* bacteria resembles main transcriptional changes in some endosymbiotic bacteria. In the final cell of *E. coli* subjected to experimental evolution under strong genetic drift, some important pathways show overexpression (in red) or downregulation (in blue) when comparing evolved transcriptomes against the ancestral transcriptome. These pathways resemble those observed in *B. aphidicola*, some of which are involved in the host–bacterium interaction, including synthesis of essential amino acids, and others are more linked to functions unrelated with the host, including the chaperone systems GroEL and DnaK, and those corresponding to the flagellum and bacteria motility.

Differentially expressed genes were distributed in a total of 129 pathways (Supplementary Figure S2). Of relevance among these pathways is the one involved in acetate utilization, with many of its genes exhibiting upregulation during the experimental evolution. Strikingly, *fadB*, a central gene in the acetate production pathway encoding a 729 amino acid-long protein, fixed a nonsense mutation (Tyr539*) and a non-synonymous mutation (Cys494Gly) very close to the substrate-binding site (residue 500) that has very likely affected the function of this gene. The upregulation of other genes in this pathway may have represented a compensatory response to *fadB* mutations. Regulation was also altered for genes involved in amino-acid biosynthesis (including chorismate, isoleucine, leucine and tryptophan) and synthesis of vitamins biotin, B_6_ and D (Figure 7). Noticeably, amino-acid biosynthesis pathway genes underwent downregulation during the first stage of the evolution experiment but recovered their expression at the end of the experiment (Figure 7), perhaps resulting from the silencing of the regulatory genes of the operons *leu*ABCD and *trp*EDCBA during the evolution experiment, an event paralleled by some endosymbiotic bacteria of insects (Moran *et al.*, 2005). Moreover, some chaperones (*groESL*, *dnaK*), transporters and transcription factors increased their transcriptional levels along the evolution experiment, in concordance with observations in endosymbiotic bacteria of insects (Baumann *et al.*, 1996; Moran, 1996; Douglas, 2003).

Among the fully silent genes, that is, genes present in the genome but for which we obtained no RNA reads throughout our evolution experiments, 60% comprised transposons and prophages, known to have been lost soon after the establishment of endosymbiosis between bacteria and insects (van Ham *et al.*, 2003). Noticeable is also the missing coverage of eight tRNA genes, involved in the transfer of anticodons for alanine, glutamate and isoleucine, which represents 10% of all tRNA genes in the genome. In these genes, we detected no silencing mutations, hence the absence of reads for them may be due to the silencing of their regulators. Other genes involved in central metabolism (*sgrT*: inhibitor of glucose uptake), regulatory genes (*sdsR*: stationary phase sRNA, *mutS* regulator; *esrE*: putative sRNA essential for aerobic growth; *rdlB*: antisense sRNA toxic peptide LdrB) and detoxification-related genes (*iroK*, *ralA*) were silenced during the evolution experiment. Among these, *esrE* is interesting in that it codes for an essential sRNA postulated to complement growth defects of *ubiJ* (*yipP*) deletion strains (Chen *et al.*, 2012; Aussel *et al.*, 2014). Silencing of this gene may therefore lead to a declined growth rate, a feature characteristic of endosymbiotic bacteria of insects housed within the limited space of insect bacteriocytes.

## Discussion

As heritable symbionts are clonal, being transmitted through matrilines, the population structure and size results in much less efficient selection acting on the genomes compared with their free-living relatives (Moran, 1996; Wernegreen, 2002; Pettersson and Berg, 2007; Wernegreen, 2011). Against this general pattern, we observed higher selective pressures in some of the endosymbiotic bacterial genes compared with their free-living relatives. Such constrained genes mainly encode functions contributing to buffering the deleterious effects of mutations. It is possible, however, that stronger constraints at some of these genes, such as *groE* (Henderson *et al.*, 2013), may result from the usage of alternative, previously un-exploited, functions that compensate those that were lost after symbiosis. Moreover, the range of functions of protein interaction partners increases with decreased genome size (Kelkar and Ochman, 2013). This increase in the number of functions of a gene would lead to increased protein functional density and selective constraints on symbiotic genes (that is, *d*_N_/*d*_S_ would decrease as the number of functions increases). Moonlighting proteins have been identified among chaperones, transcription and translation proteins (Henderson *et al.*, 2013; Liu *et al.*, 2014; Gancedo *et al.*, 2016), categories that include the strongly constrained proteins found in our study. This supports a possible shift in the function of such constrained genes after symbiosis.

Has the genome dynamics of the endosymbiont been driven by selection imposed by the host? There is extensive genomic and metabolic integration between the host and the endosymbiotic bacterium. Roughly, 10% of the genes in *B. aphidicola*, the symbiotic bacteria of aphids, are devoted to the synthesis of essential amino acids needed by the insect host (Moran *et al.*, 2005), many of which are being regulated by proteins from the aphid (Price *et al.*, 2014). An aphid-encoded protein has been shown to localize within *Buchnera* cells (Nakabachi *et al.*, 2014), and the aphid host has allowed bacteria by the loss of genes underlying immune responses to Gram-negative bacteria (Gerardo *et al.*, 2010; International Aphid Genomics, 2010). Finally, host developmental age seems to impact the transcriptome of the endosymbiotic bacterium in aphids (Bermingham *et al.*, 2009). Despite this integration, we find that signatures of sequence evolution are unrelated to the host, with evidence for strong constraints being found in genes encoding proteins that buffer the consequences of genetic drift.

In support of the predominant role of bacterial population dynamics on the evolution of their genomes, we found that the genomic and transcriptomic evolutionary trajectories of experimentally evolved *E. coli* populations exhibit striking coincidences with the evolution of *Buchnera* and many other endosymbiotic bacteria. Given the short evolutionary time of our evolution experiment, such similarities between experimentally evolved and endosymbiotic bacteria support that most events of gene loss and evolution may have taken place during the first stages of bacterial symbiosis with insects and are the product of chance. The role of the host in these genome evolutionary dynamics would therefore be limited to the provisioning of a stable and rich cellular environment to the bacterium, hence relaxing the selective constraints on most endosymbiotic bacterial genes. Therefore, the successful relationship between the aphids and their bacteria is likely the result of three main events: (a) the maintenance after the infection of bacterial genes essential for the host, (b) the evolution in the bacterium of mechanisms for mutational buffering (Moran, 1996; Fares *et al.*, 2002b; Sabater-Muñoz *et al.*, 2015), and (c) an increase in the functional complexity of retained proteins in endosymbionts to compensate for their irreversible degenerative functional evolution.

Genome size reduction is symptomatic of all known symbiotic bacteria (Moran, 1996; Wernegreen, 2002). The compensation of bacterial functions by the host has been proposed to facilitate gene or functional loss in symbiotic bacteria over time, forcing a vertiginous fall of the lineage into what some authors call ‘symbiosis rabbit hole' (Bennett and Moran, 2015). This hypothesis predicts a faster gene loss in endosymbionts than in host-devoid systems in which bacteria evolve under genetic drift. Our observation of a faster rate of genome reduction in experimentally short-time evolved bacteria suggests faster rates of gene loss in endosymbiotic bacteria at the beginning of the symbiosis, which may have slowed down as the density of essential genes for sustaining minimal bacterial life, the host or both increased (Tamas *et al.*, 2002).

The finding in our experimentally evolved lines of genome-wide deregulatory dynamics similar to those of endosymbiotic bacteria supports a prominent role for chance in the evolution of endosymbiotic bacteria. Under this view, chance would lead to convergent patterns of gene evolution and loss in bacteria while the survival of bacteria–host associations would be possible if such patterns were compatible with the metabolisms of the host. This view does not require invoking the necessity generated by the host of having a balanced diet but has likely emerged neutrally as a result of the irreversible genomic decay of endosymbiotic bacteria (Bennett and Moran, 2015).

## Figures and Tables

**Table 1:** Relative selective constraints in endosymbiotic bacterial genomes

*Endosymbiotic group*	*No. of genes with R⩽1 (%)*	*No. of genes with R>1 (%)*
*Buchnera*	33 (6.8%)	454 (93.2%)
*Candidatus* Blochmannia	45 (9.6%)	422 (90.4%)
*Wigglesworthia*	34 (6.6%)	484 (93.4%)
*Candidatus* Baumannia	17 (3.8%)	431 (96.2%)
*Blattabacterium*	99 (28.1%)	253 (71.9%)
