# Supplementary material for: Chance and necessity in the genome evolution of endosymbiotic bacteria of insects
Source: ISME J. 2017 Mar 21;11(6):1291–304. doi: 10.1038/ismej.2017.18 (PMC5437351; doi:10.1038/ismej.2017.18)
Supplement: Supplementary Table S1 [file ismej201718x1.pdf]

Table S1: Shifts in the selective constraints between free-living bacteria and endosymbiotic bacteria. BAp, BSg, Bflo, Bpen, Bcic_HC, Bcic_BGSS, Wgbrev, Wgmm, Bger, Pam, refer to Buchnera Acyrthosiphon pisum, Buchnera Schizaphis graminum, Blochmannia floridanus, Blochmannia pennsylvanicus, Baumannia cicadellinicola strain HC, Baumannia cicadellinicola strain BGSS, Wigglesworthia glossinidia brevipalpis, Wigglesworthia glossinidia morsitans morsitans, Blattela germanica, and Periplaneta americana, respectively.

**BAp-BSg**		**BAp-BAk**		**Bcic_HC-Bcic_BGSS**		**Bflo-Bpen**		**Wgbrev-Wgmm**		**Bger-Pam**	
**Gene**	**R**	**Gene**	**R**	**Gene**	**R**	**Gene**	**R**	**Gene**	**R**	**Gene**	**R**
dnaC	0.194	rpmD	0.02426766	rpsR	0.04169837	accA	0.258	cspE	0.000	hisH	0.113
rpsK	0.290	rpsR	0.07030893	groES	0.24256651	ilvD	0.277	pal	0.224	gdhA	0.114
dapA	0.301	rpsS	0.1684958	rpsS	0.25112976	ahpC	0.305	clpX	0.249	glgA	0.121
ilvD	0.305	rpsT	0.17523364	rpsJ	0.2872093	dapA	0.336	glnA	0.396	mscS	0.144
clpX	0.307	rpmE	0.22288557	rpsT	0.29238985	hisC	0.361	pyrB	0.415	plsC	0.164
rpe	0.317	rpmA	0.23943662	rpmD	0.33429064	rpsT	0.372	mqo	0.420	H38	0.182
rpmD	0.350	groES	0.3028169	rpmB	0.35682558	argI	0.381	groES	0.471	atpE	0.193
rpsJ	0.370	dnaB	0.30382294	rplS	0.38745387	mqo	0.384	rpmB	0.475	speB	0.216
lysA	0.430	rpsQ	0.31578947	thiS	0.81117534	clpB	0.390	fliC	0.489	sucB	0.232
fba	0.503	groEL	0.3526971	rpmE	0.81343284	groES	0.391	ahpC	0.500	dxs	0.235
rpmB	0.525	dnaC	0.44176707	hisG	0.87799043	rnhA	0.398	ubiG	0.508	fabH	0.250
rpsS	0.544	rpsM	0.48032787	hemD	0.89480355	rpmB	0.405	rpsS	0.551	greA	0.256
pyrD	0.574	rpmB	0.50014806	accC	0.90116279	ibpA	0.431	rpmD	0.578	fabG	0.263
yhfC	0.594	rplS	0.50342646	pabA	0.99330357	ubiG	0.445	fliN	0.587	yigI	0.273
ahpC	0.608	rplP	0.5597723	cysE	1.0375	gltP	0.455	fbaA	0.656	wzxC	0.286
cbiO	0.616	rplL	0.58090615	pabC	1.05180534	rpsS	0.456	rpe	0.666	murG	0.290
rplS	0.695	rpsK	0.59330144	bioC	1.1138535	ubiD	0.458	ubiD	0.678	murC	0.308
rplP	0.704	rplE	0.59824047	ribH	1.11553273	glnA	0.502	hspQ	0.692	hisA	0.311
hycE	0.712	rplI	0.6539961	dnaK	1.11943794	fabG	0.533	dapA	0.761	nusG	0.317
hisC	0.717	rpsN	0.78289474	cspE	1.13648294	fbaA	0.560	mltC	0.766	argF	0.328
mltC	0.724	fusA	0.86768448	recA	1.14176245	rpe	0.560	rplP	0.778	H8	0.345
rpsM	0.724	rpsB	0.88044486	rpsB	1.15199259	cysK	0.569	emrR	0.794	skp	0.346
hslS	0.762	rplR	0.88711036	ileS	1.16047431	lysA	0.597	rpsM	0.795	H19	0.353
ptsH	0.807	rpsI	0.93459916	groEL	1.19087137	pal	0.622	rpsJ	0.798	paaD	0.362
rplE	0.900	rpmC	0.97091932	rplY	1.208	yqeI	0.632	rplS	0.856	rpsU	0.367
dnaB	0.916	rplJ	0.99912664	rplP	1.23244782	rplP	0.705	slyA	0.948	trxA	0.369
rpmA	0.922	rplN	1.00436047	hslU	1.24873096	cspC	0.730	pepP	0.983	rpsR	0.369
nusG	0.939	rpoC	1.01362398	rpsM	1.2704918	ybhL	0.734	hfq	0.986	feoA	0.377
rplN	0.996	gyrA	1.05486284	tilS	1.27911516	groEL	0.796	lpp	1.023	H43	0.380
rplI	1.051	hupA	1.12941176	rplI	1.291423	prfA	0.798	groEL	1.026	rluB	0.381
rpsN	1.127	rplW	1.1360799	cysC	1.29350105	lpp	0.840	rpsN	1.080	H3	0.384
groES	1.132	minC	1.15472482	map	1.31518325	aroK	0.891	accA	1.117	H4	0.405
glpF	1.134	map	1.16544503	pabB	1.31537861	rpsM	0.898	pyrD	1.135	cdsA	0.431
minC	1.192	aroD	1.21041667	bioD	1.32773939	yigL	0.905	rpmC	1.179	kdsA	0.438
rpsB	1.231	rho	1.22641509	hfq	1.34554974	serC	0.911	rplN	1.183	hemD	0.443
rpsI	1.246	dnaK	1.23653396	metR	1.36315789	crp	0.923	rpsT	1.202	rpsK	0.450
rpsT	1.248	yoaE	1.25940594	glyA	1.40069686	rpsJ	0.941	rpsK	1.215	rpsQ	0.450
rnt	1.261	rplY	1.27955556	bioF	1.43233358	rplS	0.993	mrdA	1.219	proS	0.458
rplJ	1.269	yeeX	1.3195122	pyrG	1.43956044	aroE	0.995	ackA	1.250	rpsL	0.467
gyrA	1.377	ybeY	1.36703601	ssb	1.4469496	mrdA	1.020	folB	1.293	recG	0.468
rplY	1.390	rpsD	1.38131313	bioA	1.47890819	ptsH	1.056	fliL	1.343	actE	0.498
atpB	1.423	rnt	1.41140216	thiL	1.50057803	rplN	1.088	aroK	1.396	trpE	0.506
nuoF	1.435	rnc	1.41176471	hemC	1.56007067	lpxA	1.158	nusG	1.446	ribD	0.528
rplL	1.499	aroB	1.45578231	rpoC	1.57493188	nupC	1.162	rpsB	1.448	dedA	0.566
rpsD	1.541	aceE	1.46917808	fusA	1.57888041	ubiF	1.163	prfA	1.474	dnaG	0.623
mscK	1.575	pnp	1.50596659	cysI	1.62790698	nuoCD	1.172	ppa	1.482	rluD	0.675
ackA	1.585	ileS	1.51857708	hflC	1.65423729	yjgF	1.196	gloB	1.523	H17	0.676
tkt	1.591	ppa	1.55310881	accB	1.7068323	rpsK	1.222	skp	1.611	infB	0.682
aroK	1.626	yabI	1.55981417	cyoC	1.71620326	emtA	1.231	thiS	1.621	mreD	0.691
yggW	1.649	hemC	1.60424028	dxr	1.72714681	rpsB	1.282	rpmA	1.632	groES	0.712
pitA	1.670	pfkA	1.63870968	ribB	1.728125	emrE	1.367	rplE	1.700	rmuC	0.745
pyrB	1.721	ompA	1.64083939	dnaB	1.73440644	pitA	1.507	rpsD	1.744	rseP	0.757
ppa	1.754	bioA	1.64516129	rpsN	1.74868421	tktA	1.530	motA	1.826	folC	0.763
dnaK	1.760	ispD	1.77963526	pitA	1.78467153	rplL	1.535	fliA	1.873	rho	0.767
groEL	1.774	rpoB	1.78181818	pfkA	1.83225806	hisG	1.561	gyrA	1.915	secE	0.774
rpoC	1.809	cyoC	1.78906999	cyoB	1.83492823	degQ	1.618	ispU	2.014	atpF	0.777
aceE	1.854	nuoA	1.79801325	rnt	1.83821263	metC	1.627	ubiH	2.155	mreC	0.778
hemC	1.894	rpoD	1.80952381	deaD	1.87228261	omp	1.640	rplY	2.173	menB	0.778
ompC	1.894	ssb	1.82095491	ispE	1.87893232	ytfF	1.665	hemD	2.179	lysC	0.786
fliN	1.993	dnaT	1.85120532	purE	1.8793456	map	1.666	pitA	2.233	rnz	0.791
ssb	1.999	hisG	1.86602871	rpsI	1.89345992	mprA	1.685	degQ	2.275	uppP	0.792
rpsQ	2.077	dapA	1.90621469	folK	1.90872483	uppS	1.709	lpxA	2.302	pheA	0.797
yheL	2.091	gshB	1.91590214	dnaG	1.91269841	gyrA	1.761	rnt	2.344	sfp	0.822
thiL	2.107	rpsG	1.91666667	fabG	1.91693291	manZ	1.790	bioC	2.383	ribF	0.823
prfA	2.124	nusA	1.92137097	minC	1.93457944	dnaK	1.858	glyA	2.402	H46	0.827
rplR	2.129	cysC	1.98637317	rplE	1.94428152	rplE	1.863	cyoB	2.405	leuA	0.835
yabI	2.133	rplC	2.01573034	aroK	1.96238245	nuoF	1.906	ftn	2.446	H40	0.837
yeeX	2.137	pyrF	2.01944895	priA	1.96485062	cyoC	1.929	pyrI	2.446	gltP	0.850
secE	2.140	guaC	2.02941176	hisC	1.99726402	hlpA	1.950	rpoC	2.474	birA	0.858
mesJ	2.166	dnaJ	2.08627451	nadD	2.03809524	yccV	1.981	hslV	2.518	mreB	0.868
nuoA	2.172	glyA	2.09291521	slyA	2.04669887	secE	2.004	fliE	2.615	sodA	0.870
map	2.174	ilvI	2.1061008	nusA	2.07459677	rnpA	2.053	rpsQ	2.615	spr	0.880
pfkA	2.192	ftsW	2.12929624	purN	2.07549361	rne	2.056	dnaK	2.630	H39	0.889
glyA	2.264	cyoB	2.13636364	hflK	2.0921659	rpsN	2.126	secY	2.658	H36	0.890
carA	2.287	thiL	2.13641618	lnt	2.09288824	rpsI	2.129	panC	2.713	mrdA	0.895
ydiK	2.352	coaD	2.15079365	aroA	2.09939148	hemD	2.147	ssb	2.718	lgt	0.900
cyoC	2.396	typA	2.1678487	nuoM	2.11547344	plsX	2.154	tmk	2.718	yajC	0.904
hemD	2.407	fliE	2.16813294	rpsP	2.13662457	nusG	2.156	bioF	2.773	rpiB	0.921
pyrF	2.444	bioD	2.19151037	cysJ	2.14532374	rpsD	2.186	secE	2.797	aroA	0.935
ftsH	2.459	flgJ	2.23753666	cysN	2.15495495	ppa	2.209	dnaB	2.809	guaA	0.976
cls	2.507	deaD	2.24184783	hns	2.15902579	aceE	2.216	map	2.848	ygfA	0.981
flgA	2.546	trmD	2.2503639	ppa	2.15932642	lpxK	2.295	cyoC	2.856	rnhA	0.998
ompA	2.554	atpB	2.26018397	ispD	2.15957447	ptsI	2.352	sdhC	2.885	gyrA	1.011
rpoB	2.555	holB	2.28060046	hisH	2.18353345	rplY	2.367	ispE	2.890	mntH	1.016
pnp	2.590	ybeD	2.28358209	trmD	2.18922853	ilvE	2.381	gltX	2.923	sdhA	1.036
ybeD	2.591	secE	2.28631876	rplL	2.23867314	rpoC	2.383	rplJ	2.973	groEL	1.056
rho	2.667	flgA	2.34854151	pyrB	2.2733564	gloB	2.405	flgI	2.976	H6	1.058
nusA	2.673	prfB	2.38297872	rpsK	2.29186603	metG	2.447	bioH	3.031	aroE	1.060
atpG	2.679	dnaG	2.3968254	rpsD	2.32828283	bfr	2.450	rpsP	3.070	rplI	1.061
ptsI	2.701	argA	2.39939024	aspA	2.32921811	glyA	2.493	ompC	3.072	H21	1.067
rnc	2.788	aroH	2.40909091	tktA	2.34018265	tilS	2.505	yqjA	3.077	ddl	1.077
dnaT	2.804	gloB	2.43478261	rpmC	2.35553471	cls	2.679	tilS	3.099	nusB	1.079
ychA	2.835	cyaY	2.47248577	cca	2.36594203	holC	2.682	rplR	3.118	atpB	1.087
cysC	2.863	pyrG	2.47912088	queA	2.37191651	rplJ	2.686	thiL	3.246	hinT	1.087
ilvI	2.910	rpsP	2.53501722	rho	2.39622642	ygdP	2.748	pykA	3.270	tatC	1.092
argA	2.971	mutT	2.54609929	rplR	2.41449031	sdhC	2.752	rne	3.281	sdhC	1.100
pyrG	2.980	cysI	2.55426357	rnfB	2.42525773	rplR	2.790	ftsI	3.303	dnaK	1.111
endA	3.009	smpA	2.56381798	nuoH	2.44	lpxL	2.804	pgi	3.311	rpsJ	1.117
rplC	3.095	ftsY	2.60488246	thiF	2.44525547	rpmA	2.825	ftsH	3.322	glnS	1.117
hemK	3.149	gmk	2.60635697	coaD	2.46560847	nlpD	2.858	arnT	3.325	phnP	1.133
gltX	3.158	nuoH	2.61142857	thiE	2.46564885	gltX	2.878	hflC	3.363	leuS	1.138
smrA	3.188	ptsI	2.67316017	thiG	2.47665848	cyoB	2.929	pyrG	3.385	aroD	1.146
lspA	3.201	sohB	2.67318982	cysQ	2.4911032	sufD	2.934	ribB	3.404	rplO	1.157
gloB	3.262	atpG	2.68708609	metC	2.49759615	dnaJ	2.947	yceG	3.406	rpmJ	1.190
rplB	3.288	yedA	2.7173913	ptsI	2.504329	manY	2.947	nusA	3.419	pssA	1.190
dnaX	3.289	gyrB	2.77023499	pnp	2.51312649	trmD	2.952	flgE	3.440	rpsN	1.199
cyoB	3.304	pyrC	2.78171642	pdxB	2.54587156	psd	2.974	hslU	3.448	rpoC	1.208

R = (dN/dS)endosymbiont/(dN/dS)free-living
BAp: ***Buchnera Acyrthosiphon pisum***
BSg: ***Buchnera Schizaphis graminum***
Bak: Buchnera Acyrthosiphon Kondoi
Bcic_HC: Baumannia cicadellinicola str. HC
Bcic_BGSS: Baumannia cicadellinicola str.BGSS
Bflo: ***Candidatus Blochmannia floridanus***
Bpen: ***Candidatus Blochmannia Pennsylvanicus***
Wgbrev: ***Wigglesworthia Glossinidia brevipalpis***
Wgmm: ***Wigglesworthia Glossinidia morsitans morsitans***

panC	3.312	rplA	2.80474934	folC	2.54753723	ftsH	2.987	thiC	3.456	rplQ	1.210
rsmD	3.316	hisH	2.80617496	carA	2.592827	ssb	3.098	carA	3.476	ndh	1.211
trmD	3.320	rpsO	2.81525424	thiC	2.59308511	rplX	3.113	rseP	3.526	rpsM	1.223
atpD	3.382	rpoH	2.82333333	holB	2.60277136	yoaE	3.124	aceE	3.530	tktA	1.245
ispU	3.400	pitA	2.82846715	rpoD	2.62337662	minC	3.135	cls	3.635	pnuC	1.251
carB	3.420	yggS	2.83275663	nfo	2.625	rplI	3.215	yhhF	3.651	H7	1.252
deaD	3.455	rplO	2.86842105	metK	2.62962963	asd	3.258	holE	3.665	rpsD	1.258
rsxB	3.484	fpr	2.88248337	holD	2.66536357	holD	3.267	rplL	3.681	mvaD	1.262
apbE	3.503	rne	2.88356164	rpsQ	2.66801619	ftsW	3.345	dnaJ	3.718	H27	1.263
hisG	3.506	hisC	2.88372093	rplJ	2.69432314	rpmE	3.371	yadH	3.743	acnA	1.264
rpsP	3.512	thrC	2.88803089	ribD	2.6970297	yaeL	3.383	sufD	3.770	sucA	1.268
fpr	3.517	dut	2.89158879	rnb	2.71309771	pnp	3.399	mntH	3.798	mrcA	1.272
ybeY	3.527	deoB	2.89697802	nadB	2.73474178	rpsP	3.400	rpsI	3.836	aspC	1.274
secA	3.544	carA	2.90084388	aceE	2.74828767	kdsA	3.418	atpB	3.837	murB	1.285
cyaY	3.551	dxr	2.90858726	sufD	2.76610768	dnaB	3.436	flhC	3.894	sdhB	1.288
secY	3.560	rpsF	2.92468619	serC	2.78657074	nuoH	3.462	rplC	3.897	mdh	1.306
alr	3.608	fabG	2.95367412	corA	2.82407407	atpG	3.544	pdxA	3.932	tatD	1.308
hpt	3.621	cysN	2.98198198	panE	2.83022388	tolR	3.577	fliO	3.953	def	1.310
cspE	3.625	htpG	3.05026455	sufS	2.83303411	ilvA	3.582	folC	3.979	fjo17	1.317
purH	3.663	amiB	3.1095176	rpoH	2.85	atpB	3.584	rpoB	4.014	rpsC	1.327
fliE	3.678	infB	3.12152778	dsbA	2.85572139	cysE	3.602	rplI	4.028	rplF	1.338
rne	3.686	panC	3.14227086	atpB	2.87253614	pyrG	3.608	dcd	4.061	rpsF	1.339
rfaE	3.691	miaA	3.14385475	ribA	2.87336245	yeeX	3.622	ftsB	4.076	rplK	1.351
hslV	3.721	mutY	3.14831804	rpmA	2.88419405	yeaZ	3.633	lpxH	4.087	prsA	1.354
ipk	3.727	ribB	3.16875	pdxA	2.89026063	grpE	3.635	guaB	4.091	H48	1.375
guaC	3.737	hslV	3.18090452	ribF	2.90199336	nuoA	3.696	putA	4.158	secY	1.380
glmS	3.843	atpD	3.18396226	cysG	2.90408163	cysC	3.696	pnp	4.182	rpsA	1.385
thrC	3.851	nrdA	3.20363636	rpoB	2.93090909	rplO	3.722	phr	4.266	mvaK	1.388
fsaA	3.862	yajR	3.20530973	mutL	2.93344426	rpoB	3.739	rplB	4.277	serA	1.391
dnaA	3.865	dnaX	3.22222222	fabH	2.93710692	sucD	3.755	hemH	4.334	H23	1.396
ftsW	3.880	glpF	3.24343675	infB	2.95486111	yhhF	3.757	flgA	4.355	H28	1.400
htpG	3.953	cyoD	3.26621924	rimM	2.96737589	ygbQ	3.764	glyS	4.373	mutY	1.403
aroH	3.956	fliI	3.27254098	pyrD	2.97222222	xthA	3.775	glmU	4.386	iscS	1.412
aroC	3.965	obgE	3.27900552	grpE	2.97427653	yqgE	3.809	ubiX	4.424	gcp	1.422
rpsG	3.991	folC	3.29095074	tmk	2.98489933	rnt	3.841	gshB	4.425	pcrA	1.423
rpmC	4.004	pncB	3.3248731	secE	3.00141044	yjcE	3.908	rho	4.431	accA	1.430
tmk	4.019	nlpD	3.34318902	miaA	3.00698324	rplB	3.928	atpH	4.442	pyrE	1.451
pyrC	4.030	infC	3.36912752	dut	3.00934579	nagA	3.957	hflK	4.486	infA	1.453
folC	4.083	hslU	3.41624365	nuoA	3.01655629	sufE	4.022	pgsA	4.508	actB	1.454
mutY	4.129	argH	3.41706161	panC	3.04103967	yidC	4.025	yggS	4.534	secDF	1.463
yidC	4.142	mdlB	3.44598338	hisF	3.046875	ilvM	4.036	folD	4.568	ccoH	1.487
flgJ	4.162	argG	3.45283019	panB	3.05066667	purH	4.091	ycaR	4.572	clpB	1.487
ribD	4.171	tsf	3.5	purA	3.05594406	guaB	4.138	rplX	4.586	rplJ	1.502
hslU	4.184	dsbA	3.5199005	purK	3.08950086	hisI	4.176	flgB	4.591	surE	1.510
yggS	4.185	frr	3.52446184	purM	3.09108159	slyA	4.206	dxr	4.619	secA	1.519
dxr	4.185	rplQ	3.52941176	nuoG	3.10135135	ribD	4.267	rplW	4.662	tilS	1.528
rplA	4.272	lgt	3.54094293	cysB	3.1037037	mntH	4.276	ybeY	4.678	secG	1.528
rplO	4.274	pyrB	3.56747405	pyrF	3.10372771	lnt	4.285	lnt	4.688	pdxA	1.529
speD	4.305	purH	3.56873823	pcnB	3.11086475	folC	4.291	purE	4.702	H26	1.532
ddl	4.347	flgC	3.57024793	rplW	3.15855181	yqjA	4.298	sdhB	4.755	gidA	1.534
trpC	4.369	smg	3.60681115	dnaJ	3.2	pabB	4.303	dxs	4.770	ftsK	1.550
infC	4.404	orn	3.63333333	dnaX	3.22735043	hemC	4.370	nadD	4.781	guaB	1.555
rlmG	4.438	nadE	3.66201117	rpsO	3.23559322	leuA	4.372	yidC	4.789	truB	1.574
nusB	4.438	ptsG	3.67153285	glyS	3.26158038	carA	4.375	fpr	4.793	rplL	1.577
entA	4.443	ribF	3.68438538	purD	3.26470588	aroC	4.381	purH	4.813	sufS	1.582
rluB	4.446	rnb	3.69022869	sufE	3.28571429	pykA	4.421	pyrC	4.829	actA	1.583
csdA	4.464	nuoJ	3.71528998	pyrC	3.29104478	dsbB	4.477	nrdI	4.884	rplV	1.583
panB	4.532	atpH	3.77876896	htpG	3.30687831	dxr	4.497	coaD	4.908	H14	1.588
degQ	4.540	cysG	3.78571429	nadC	3.30892144	hemK	4.500	thiE	4.951	yqfO	1.593
pykA	4.563	mtlA	3.80705009	nuoL	3.3164557	cysG	4.504	kdsA	4.955	gldE	1.595
pgi	4.571	lysA	3.82240437	bioH	3.35186722	fpr	4.514	sucD	4.958	H13	1.596
ftsB	4.578	pyrI	3.82625483	gloB	3.40771124	cysJ	4.532	purU	4.990	rplP	1.605
trpD	4.605	gapA	3.83076923	eno	3.41803279	atpC	4.557	damX	5.039	glmS	1.620
rpoD	4.610	pgi	3.8337469	aroC	3.42675159	purB	4.647	ribE	5.040	purB	1.622
rplW	4.612	flgK	3.8379085	asnS	3.42925659	aroB	4.654	rplO	5.073	cysK	1.624
hflC	4.617	argB	3.84097035	ispA	3.4519774	trxB	4.749	eno	5.078	H35	1.631
dnaJ	4.664	nfo	3.84745763	aroB	3.49886621	rpsQ	4.758	purM	5.093	hemC	1.651
ribF	4.668	nusG	3.87234043	rnpA	3.50134048	yceL	4.770	ksgA	5.093	dapB	1.654
nuoH	4.688	cyoE	3.90301724	recB	3.53706755	rfaC	4.776	fliF	5.101	miaA	1.657
hisA	4.691	talA	3.91242938	purH	3.54425612	lplA	4.786	cyoA	5.123	trmU	1.663
cysJ	4.722	hpt	3.92887029	cyoA	3.57437071	rplC	4.791	tolR	5.151	pyrG	1.694
rluA	4.748	flgB	3.93730408	frr	3.60861057	apt	4.847	nadC	5.162	gltX	1.697
argC	4.816	carB	3.94222222	lgt	3.62282878	pdxA	4.857	atpD	5.165	psd	1.706
aroB	4.819	yggW	3.97766749	pgi	3.62779156	infC	4.882	hemC	5.171	hupB	1.715
glyS	4.848	cca	4.01811594	tsf	3.64615385	atpH	4.899	sdhD	5.176	ygcM	1.732
hisH	4.853	recD	4.01915709	rluD	3.64705882	gpmA	4.902	rplQ	5.253	lspA	1.737
pncB	4.920	panB	4.05333333	hslV	3.67839196	ilvG	4.905	prlC	5.271	carA	1.743
thrB	4.924	cysQ	4.09074733	valS	3.68614719	yjeP	4.921	flgF	5.288	rpsH	1.745
flgB	4.957	rpoA	4.09352518	lspA	3.69117647	rmuC	4.947	ispD	5.313	rplU	1.752
nuoN	4.967	yeaZ	4.10178817	murF	3.69875776	leuD	4.953	panB	5.342	ygcF	1.755
atpA	4.968	folD	4.1043771	gpsA	3.70588235	pgi	4.978	metG	5.398	smpB	1.768
secB	5.021	cysJ	4.10647482	dxs	3.72401434	rho	4.980	minC	5.412	luxE	1.769
cysE	5.041	atpC	4.12525667	purF	3.76811594	miaA	4.982	rlmB	5.436	lolC	1.775
lysS	5.080	ung	4.14095745	minD	3.7712766	secA	4.988	tolB	5.439	trpA	1.776
nuoK	5.087	tmk	4.14261745	rpsA	3.77664975	tpiA	5.038	secA	5.451	atpD	1.781
bamE	5.143	pyrD	4.15277778	accD	3.77777778	leuB	5.083	lgt	5.533	ribH	1.791
nfo	5.145	aroA	4.1643002	metE	3.80225989	metB	5.087	orn	5.555	hisS	1.796
rnpA	5.187	murE	4.16548043	rplO	3.80451128	rlpB	5.134	folK	5.562	pdhA	1.815
fdx	5.191	atpA	4.18823529	aceF	3.81233596	cutA	5.149	cvrA	5.573	H33	1.854
hflK	5.197	nuoG	4.24324324	thyA	3.82417582	ubiX	5.157	pdxB	5.672	lepB	1.860
argH	5.217	rplB	4.247557	pyrI	3.83783784	mutY	5.177	flgD	5.678	pckA	1.873
miaA	5.255	rnpA	4.28820375	atpG	3.83940397	trpA	5.189	ddlA	5.699	rpsE	1.878
ispD	5.291	fkpA	4.31123389	secD	3.85780886	leuC	5.241	ispA	5.708	rlmB	1.885
phr	5.299	purA	4.34265734	cysD	3.90419162	atpF	5.241	pyrF	5.781	purH	1.893
tsf	5.329	ribA	4.34934498	hisS	3.92191436	hflC	5.251	secG	5.791	dapA	1.902
nuoJ	5.332	hflC	4.36271186	purC	3.94369973	dxs	5.261	tufA	5.794	dapF	1.919
trxB	5.406	flgF	4.39036545	dcd	3.95731707	thrA	5.281	dfp	5.805	aroC	1.938
queA	5.417	speD	4.39790576	psd	4.00203666	cca	5.284	fliP	5.806	gcvP	1.943
asnC	5.460	htpX	4.4097561	hisI	4.03209877	ybgF	5.286	asd	5.845	recA	1.975
argG	5.496	ilvD	4.41853933	tyrS	4.04727273	gmk	5.290	trmD	5.880	mltD	1.977
rplU	5.500	aceF	4.43307087	degQ	4.07100592	pdxB	5.318	atpG	5.911	H52	1.986
pyrI	5.507	thrB	4.45	sufC	4.13953488	rlmB	5.328	pta	5.922	coaE	1.991
ybeX	5.538	glyS	4.47547684	rplB	4.14006515	nusA	5.344	hemF	5.929	queA	2.002
gmk	5.588	fliF	4.48178138	metG	4.15073529	folK	5.349	rodZ	5.933	argD	2.016
flgF	5.609	secA	4.48948949	sppA	4.18309859	trpC	5.394	ygjD	5.936	H37	2.047
deoD	5.610	argD	4.4968815	gyrB	4.18798956	ftsQ	5.396	panE	5.937	galU	2.070
flgK	5.653	minD	4.51595745	rpoA	4.1942446	nuoL	5.396	asnB	5.965	rodA	2.093
obgE	5.660	tldD	4.52027027	purL	4.23896104	dut	5.396	sufE	5.989	pdxH	2.102
asd	5.661	aroK	4.52351097	rnc	4.24434389	gapA	5.467	fliQ	5.995	purC	2.111
rpsF	5.690	nuoK	4.54508197	holC	4.24956672	metE	5.501	rpsF	6.030	infC	2.124
tsaB	5.703	serC	4.57074341	leuS	4.25	smpA	5.508	ribF	6.056	oxaA	2.125
nuoG	5.704	flhA	4.57647059	bioB	4.27616279	folE	5.527	yhcM	6.063	atpH	2.129
metE	5.729	atpF	4.60329068	ftsE	4.28680203	rpsO	5.531	rpoD	6.081	tyrS	2.129
ftsI	5.760	nuoE	4.61242604	lolC	4.29700855	lspA	5.532	frr	6.082	pyrD	2.136
bioB	5.806	lysS	4.61486486	lolE	4.30989011	yrbA	5.538	miaA	6.086	ccoP	2.162

frr	5.810	nuoN	4.62403101	atpD	4.31603774	orn	5.541	purC	6.115	gcvT	2.167
coaD	5.814	ispA	4.64124294	nadE	4.32402235	tolA	5.550	yoaE	6.118	rpoB	2.185
trmB	5.820	dnaA	4.64133739	accA	4.35294118	rpsG	5.575	flgH	6.122	sufE	2.191
gyrB	5.849	aroC	4.64649682	cysH	4.36969697	gcp	5.597	htpG	6.152	ileS	2.219
gshB	5.917	lspA	4.65909091	murE	4.38434164	ksgA	5.638	rnpA	6.218	rnc	2.226
atpH	5.918	secY	4.665	emrA	4.40458015	sucA	5.640	murB	6.241	rpmH	2.229
fliQ	5.918	argC	4.67102397	gshB	4.42966361	yigB	5.646	tadA	6.277	purL	2.233
ispA	5.925	murB	4.70117647	mutS	4.45637584	gyrB	5.659	lspA	6.293	gyrB	2.243
purB	5.944	trpC	4.73613767	ispH	4.4764268	rplQ	5.704	cvpA	6.295	norM	2.251
rplF	5.947	hisA	4.76027397	purB	4.48550725	asnC	5.710	bioB	6.321	rnr	2.267
ratB	5.950	ynfM	4.82264151	hisD	4.5227882	murE	5.719	yciC	6.339	cafA	2.269
tadA	5.994	ftsI	4.83571429	lysP	4.52678571	ureG	5.775	mreC	6.367	thrS	2.285
metK	6.025	rlmL	4.84482759	rnhA	4.53164557	nuoG	5.915	ribA	6.414	ksgA	2.288
hisF	6.034	dcd	4.87804878	fabB	4.53763441	glmU	5.926	yigB	6.421	dksA	2.298
minD	6.055	eno	4.88114754	murB	4.55294118	amiB	5.952	ndk	6.450	rplY	2.306
trpA	6.061	deoD	4.89589905	atpC	4.55441478	trpD	5.955	bolA	6.453	H16	2.311
bolA	6.064	murI	4.89655172	obgE	4.55801105	nuoJ	5.960	rluD	6.473	dnaB	2.317
ileS	6.081	valS	4.9004329	pgl	4.58481013	tolB	5.966	murA	6.494	atpA	2.318
glnS	6.122	metE	4.93220339	proS	4.59217877	yrbK	5.973	ribD	6.516	H51	2.318
ung	6.141	dapB	4.94701987	trpS	4.66386555	rplW	6.011	flgJ	6.520	menC	2.325
ydgQ	6.159	pgk	4.99680511	rplC	4.68764045	ipk	6.016	flgK	6.527	alaS	2.336
fkpA	6.165	fliH	5.0302521	mreC	4.6951567	yhgN	6.049	nadE	6.580	atpC	2.362
mraW	6.182	flgE	5.0528169	secF	4.70449173	hflK	6.060	atpC	6.585	rpoA	2.370
atpC	6.210	rpsA	5.06598985	lepA	4.74297189	pepA	6.089	arnD	6.661	purE	2.372
nadD	6.223	ispH	5.09181141	emrB	4.77906977	atpA	6.163	accD	6.694	hemK	2.436
eno	6.283	purB	5.11594203	fumC	4.78508772	nagB	6.222	dapB	6.749	lysS	2.440
cyoA	6.303	nuoL	5.13562387	metF	4.79207921	ygfA	6.262	purD	6.805	rplR	2.440
gapA	6.357	rpe	5.13580247	glmU	4.80379747	pheT	6.265	gapA	6.956	lipB	2.445
glmU	6.372	hisS	5.15365239	surA	4.80415945	rplU	6.277	trxB	6.962	pheS	2.449
murG	6.396	rplU	5.16019417	cysK	4.83435583	thrC	6.281	metK	7.013	topA	2.484
pth	6.399	tig	5.22905983	pheT	4.85815603	ribF	6.294	hemG	7.054	rplN	2.501
yggX	6.419	recB	5.24217463	fabD	4.88220551	lysS	6.294	lpxD	7.059	cmk	2.539
dapB	6.420	argS	5.34948097	gapA	4.88461538	lpxD	6.327	thiG	7.087	trmE	2.544
trxA	6.475	cls	5.40425532	thiH	4.89527027	ygfE	6.380	gpsA	7.151	H29	2.550
recD	6.516	ychE	5.40659341	folE	4.90421456	glnS	6.402	rpoH	7.182	H18	2.619
grpE	6.521	fliQ	5.42105263	fre	4.94202899	minD	6.403	xthA	7.196	hisB	2.624
tufB	6.547	mnmA	5.42801556	rnr	4.9566787	cyoE	6.427	ydiK	7.232	dnaX	2.676
orn	6.548	fliP	5.43152455	guaB	4.97183099	cysP	6.482	ygfY	7.289	nth	2.700
thrA	6.564	leuS	5.43465909	hisA	5.02968037	fabZ	6.491	pepA	7.298	rplS	2.713
rplQ	6.593	gnd	5.47282609	nuoN	5.05232558	sufS	6.500	tktB	7.309	mgtE	2.720
rsmE	6.624	ppiD	5.48297214	nuoE	5.05325444	lgt	6.511	dut	7.332	adk	2.724
rpsA	6.630	mrcB	5.512	orn	5.05333333	ribA	6.536	gyrB	7.366	folB	2.724
dut	6.713	ihfA	5.53146853	ftsK	5.12075472	sdhA	6.554	fabA	7.403	H15	2.758
fliF	6.717	aspS	5.54304636	nusB	5.14356436	fabH	6.570	surE	7.467	rplA	2.786
nuoM	6.808	prfC	5.56521739	hisB	5.15178571	secD	6.664	rpsG	7.561	rpoN	2.804
pgk	6.833	pheT	5.59574468	atpH	5.20606601	ftsI	6.707	rsmE	7.605	H44	2.826
nadE	6.838	flhB	5.61428571	glnS	5.21299639	pmbA	6.729	tolA	7.605	H9	2.846
eutD	6.845	lepA	5.61445783	cls	5.22340426	pdxJ	6.780	sufC	7.636	cysS	2.862
murD	6.869	hisD	5.63538874	sufA	5.23434705	hisS	6.848	yggV	7.647	grpE	2.872
ygfZ	6.932	dxs	5.64516129	lolD	5.23863636	sdhB	6.915	rplF	7.660	H42	2.919
dxs	6.982	nuoM	5.64665127	recC	5.25625	hisD	6.927	lysS	7.759	rplW	2.939
atpF	7.007	rimM	5.66099291	prlC	5.28253968	yraL	6.963	dnaX	7.762	metK	2.940
cyoE	7.008	apaH	5.69615385	pdxJ	5.3538961	ung	7.011	cca	7.792	mutL	2.964
pgl	7.015	fliG	5.71586716	clpB	5.40201005	ybeX	7.024	ydjM	7.845	H2	2.970
smg	7.023	rplF	5.76699029	recD	5.41762452	ygfZ	7.035	pheT	7.866	carB	2.982
secG	7.028	cyoA	5.82379863	gshA	5.42446043	ftsK	7.040	minD	7.935	rplC	3.037
cca	7.035	pheA	5.83468835	rpsF	5.52301255	prlC	7.074	pck	7.960	xth	3.038
rsmI	7.050	glnS	5.84837545	alaS	5.53665689	nuoN	7.109	lpxB	7.961	murA	3.058
dcd	7.059	sucA	5.87782805	folB	5.55524079	pgk	7.122	fliH	7.992	dut	3.085
trpB	7.085	nusB	5.89108911	tpiA	5.59136213	mrcB	7.142	talA	8.017	dnaJ	3.087
rsxD	7.087	ftsZ	5.93589744	cysW	5.63636364	rimM	7.143	murC	8.030	gapA	3.114
murB	7.141	rluD	5.94117647	cysS	5.71137026	hisF	7.156	zapA	8.035	pth	3.118
rlmL	7.159	coaE	5.95041322	ygfZ	5.71493213	ybhE	7.214	cyoE	8.065	rimN	3.124
hisD	7.207	glmU	5.96624473	aspS	5.72406181	aspS	7.227	ygfZ	8.108	manB	3.131
sucA	7.247	secB	5.97238659	holA	5.74789916	nuoM	7.244	murD	8.120	H41	3.140
ychF	7.293	ygfZ	6.02111614	tgt	5.75247525	sbcB	7.303	mraW	8.134	asnS	3.180
nuoL	7.293	bolA	6.03726708	aroE	5.75409836	hldE	7.306	mdoH	8.162	fmt	3.203
prs	7.305	thrS	6.05839416	folD	5.84848485	cyoA	7.308	proS	8.229	speA	3.319
efp	7.348	tyrS	6.08363636	pdxH	5.85384615	thrB	7.324	infC	8.241	frvX	3.345
tusE	7.353	tktB	6.10126582	atpF	5.89213894	tsf	7.333	sdhA	8.282	rplB	3.448
yfgM	7.377	mutS	6.12080537	murC	5.92622951	sodA	7.337	rimM	8.288	ribE	3.474
ispH	7.382	trpS	6.17226891	thiD	5.93114754	surA	7.398	psd	8.289	trmD	3.482
flgI	7.432	flgD	6.17738791	ligA	5.94385027	ybeY	7.424	ybgF	8.351	aspS	3.543
rnr	7.488	hflK	6.17741935	dksA	5.95555556	carB	7.429	sucA	8.357	trpB	3.596
ksgA	7.512	sbcB	6.178125	pth	6.00245098	pheA	7.494	rplU	8.403	rpmE	3.596
lgt	7.538	sodA	6.23589744	truB	6.02121212	metA	7.496	ftsQ	8.412	ybdB	3.611
cysH	7.546	hscB	6.26373626	carB	6.02666667	frr	7.511	atpA	8.542	H30	3.656
htpX	7.597	metG	6.29044118	tig	6.04615385	ribE	7.521	rplA	8.552	idi	3.678
ribA	7.662	pth	6.35539216	recG	6.06097561	cysS	7.525	yajC	8.566	H20	3.758
hscB	7.683	yfgM	6.3800738	mutM	6.1013986	ndk	7.532	envC	8.690	fbp	3.785
alaS	7.700	yigL	6.38487395	dnaE	6.13888889	tadA	7.557	ribC	8.697	purA	3.796
pduS	7.716	murG	6.51005025	prfA	6.20454545	yajC	7.562	purL	8.799	serC	3.828
hisS	7.729	cysH	6.54242424	ackA	6.2661597	tmk	7.582	mnmA	8.807	eno	3.828
argS	7.781	gshA	6.54676259	thrS	6.27737226	cysQ	7.629	greA	8.845	trpC	3.838
rpsO	7.929	alaS	6.58357771	nudE	6.28308824	recD	7.681	purB	8.853	purD	3.904
gcp	7.962	murD	6.59773371	nuoJ	6.30755712	atpD	7.693	yraL	8.883	folE	3.907
tatD	7.978	bioB	6.61918605	ppnK	6.39896373	ddl	7.707	cutA	8.947	rplT	3.980
cysD	8.000	dnaE	6.64930556	folP	6.47687861	gpsA	7.724	hupA	9.007	coaD	4.016
fliP	8.059	yggX	6.66153846	artP	6.49056604	hflX	7.768	ftsW	9.052	asd	4.048
yciA	8.103	surA	6.67417678	atpA	6.55686275	mreC	7.778	hemB	9.074	trpD	4.171
flgE	8.142	flgH	6.67819149	polA	6.61471861	mreD	7.827	nhaA	9.116	H49	4.265
flgD	8.156	metK	6.70987654	ung	6.63297872	rplF	7.830	ytfN	9.144	nadD	4.329
tgt	8.181	hisF	6.734375	nrdB	6.64634146	rluC	7.842	aspS	9.205	rpmF	4.402
nuoE	8.224	ffh	6.7382199	glmM	6.80808081	hisB	7.902	glnS	9.299	purM	4.418
aspS	8.251	mtlD	6.73951435	rplQ	6.84095861	ytfN	7.949	hemK	9.308	rpsS	4.437
cysG	8.268	fdx	6.76950355	murI	6.85775862	manX	8.128	rlmN	9.350	H50	4.461
flgC	8.271	prfA	6.77272727	pssA	6.89864865	murD	8.128	ppiD	9.408	dnaN	4.593
dksA	8.275	cysD	6.83832335	lipB	6.90344828	ubiA	8.130	atpF	9.417	rpsG	4.695
rbfA	8.318	proS	6.8575419	yajC	6.92376682	sdhD	8.174	mfd	9.620	pgk	4.730
tig	8.335	asnC	6.94247788	fldA	6.94936709	cyoD	8.175	htpX	9.627	sufA	4.737
pepA	8.424	flgI	6.96794872	gltX	7.06624606	argS	8.186	dapD	9.655	polA	4.789
flgH	8.503	fliJ	7.02972028	murA	7.07608696	murA	8.193	sodA	9.705	H12	4.863
ynfM	8.504	gltX	7.05362776	adk	7.13271605	yrdC	8.229	coaE	9.718	rplD	4.967
proS	8.573	yceA	7.06940063	ruvB	7.1375	pssA	8.326	hisS	9.724	serS	5.254
mnmA	8.655	fmt	7.08571429	dapF	7.13809524	ycfH	8.330	tpiA	9.726	era	5.280
sodA	8.660	hscA	7.09634551	lepB	7.16246499	sufB	8.342	ubiA	9.767	trpS	5.538
coaE	8.678	murC	7.16120219	gmk	7.19804401	rnc	8.394	bamA	9.772	tgt	5.590
murI	8.698	glmS	7.17777778	clpP	7.1981982	proS	8.419	fliG	9.801	H5	5.662
pheA	8.700	secG	7.28024194	pgsA	7.22887324	rpoH	8.436	asnS	9.802	rplE	5.755
purA	8.720	yciA	7.35294118	mnmA	7.25680934	cysI	8.437	yfgM	9.886	uvrD	5.823
ppnK	8.730	crr	7.40758294	artQ	7.30412371	pth	8.467	truB	9.914	feoB	5.910
hisI	8.754	thyA	7.46153846	fabA	7.33333333	ftsZ	8.500	rpsA	9.989	acpP	5.944
engB	8.779	trpA	7.47433265	fmt	7.33428571	truB	8.506	murG	9.995	H32	6.267
rsxG	9.030	mutL	7.49084859	rplU	7.38834951	udp	8.511	carB	10.012	rpmC	6.524
rpoH	9.079	argE	7.50675676	ffh	7.39790576	smpB	8.572	iscS	10.159	fdx1	6.656

mutL	9.099	cysS	7.55393586	mltC	7.43614458	*ycaR*	8.577	*lptC*	10.222	*rbfA*	6.836
hisB	9.111	trpB	7.6119403	pta	7.5087108	*murB*	8.581	*lolC*	10.275	*engD*	7.046
truB	9.121	ytfN	7.62865948	murD	7.60056657	*upp*	8.586	*purK*	10.308	*glyS*	7.111
mfd	9.132	ibpA	7.66666667	cyoE	7.625	*dapF*	8.615	*rnc*	10.361	*rpmA*	7.123
amiB	9.170	uup	7.66666667	infC	7.67114094	*trmE*	8.615	*glyQ*	10.439	*atpG*	7.221
fliJ	9.217	pyrE	7.7012987	rluC	7.68531469	*murG*	8.652	*cysS*	10.565	*trxB*	7.456
ytfN	9.279	dnaQ	7.7325228	gnd	7.76086957	*yccK*	8.715	*lolA*	10.594	*obg*	9.792
thrS	9.371	hisB	7.73660714	mraY	7.88461538	*thrS*	8.797	*proC*	10.607	*cca*	9.833
sbcB	9.550	fliR	7.74049217	murG	8.01507538	*trpB*	8.809	*argS*	10.638	*rplX*	10.470
dnaN	9.553	thrA	7.74716981	dnaN	8.01657459	*imp*	8.878	*alaS*	10.654	*H10*	11.528
yajC	9.598	speE	7.8061674	fabI	8.13414634	*pdxH*	8.879	*rbfA*	10.662	*prfA*	12.351
fliH	9.600	ilvC	7.8590604	mreD	8.13648294	*tdk*	8.908	*hemA*	10.671	*miaB*	15.369
nifU	9.729	hisI	7.90864198	artI	8.29617834	*hisA*	8.952	*rpsO*	10.681		
yheN	9.799	truB	8.04848485	dacB	8.30604982	*tyrA*	8.996	*fbp*	10.683		
ilvH	9.809	greA	8.05633803	miaB	8.31355932	*trxA*	9.204	*ubiB*	10.828		
pheT	9.812	yqgF	8.08095238	plsC	8.33846154	*dnaN*	9.214	*folE*	10.967		
hscA	9.869	smpB	8.09803922	iscS	8.40092166	*nuoE*	9.240	*dapE*	11.087		
ychE	9.880	cysK	8.13496933	smpB	8.44771242	*fumC*	9.253	*adk*	11.089		
murJ	9.890	acpS	8.195	fbaA	8.46460177	*aarF*	9.277	*thrA*	11.175		
trpS	9.898	dnaN	8.22099448	coaE	8.60950413	*rplA*	9.459	*nusB*	11.235		
yrdC	9.971	minE	8.26373626	sufB	8.67114094	*truA*	9.470	*gmk*	11.304		
fabD	10.015	znuC	8.28708134	plsB	8.85276074	*yjgP*	9.532	*purA*	11.328		
amiA	10.027	mfd	8.36398467	aspC	8.92307692	*yfaE*	9.533	*ispH*	11.339		
gnd	10.058	yhiQ	8.48958333	clpX	9.00854701	*alaS*	9.562	*yqgF*	11.376		
cyoD	10.065	yfjF	8.61484099	cvpA	9.02821317	*yraP*	9.570	*thrS*	11.449		
gshA	10.221	tpiA	8.75415282	ppk	9.03773585	*ygiH*	9.605	*gshA*	11.614		
argE	10.267	ybgI	8.81395349	pepA	9.08053691	*purA*	9.618	*tsaB*	11.714		
surA	10.312	fabB	8.82437276	nuoK	9.2295082	*hldD*	9.634	*ubiE*	11.738		
mpl	10.313	truA	8.87301587	ruvA	9.23636364	*cysH*	9.656	*pth*	11.807		
lipB	10.331	pyrH	8.89312977	truA	9.29365079	*eno*	9.656	*fliY*	11.842		
rplX	10.331	dapE	8.91044776	rpsG	9.30701754	*yhbN*	9.767	*fabG*	11.850		
lolC	10.341	clpP	8.91891892	cdsA	9.31698113	*acpS*	9.800	*lpxC*	11.957		
glyQ	10.390	ycfH	8.93706294	birA	9.40148699	*tyrS*	9.805	*cyoD*	12.013		
pol	10.573	ahpC	9.00595238	greA	9.47183099	*rpsA*	9.842	*sufB*	12.081		
yciC	10.625	mltA	9.08571429	lon	9.49206349	*ppnK*	10.021	*gidA*	12.100		
clpP	10.667	ihfB	9.1299435	thiI	9.5093633	*tgt*	10.066	*trpS*	12.200		
priA	10.723	adk	9.19135802	pyrE	9.52813853	*polA*	10.149	*yrdC*	12.294		
tyrS	10.743	yajC	9.19730942	guaA	9.535	*ychF*	10.162	*pgk*	12.303		
ftsZ	10.744	topA	9.24663677	pgk	9.53674121	*murC*	10.360	*mnmE*	12.404		
mutS	10.760	flgN	9.33756345	htpX	9.57073171	*fabD*	10.552	*tsf*	12.438		
apaH	10.820	dksA	9.34074074	lolB	9.59876543	*apaH*	10.644	*murI*	12.589		
minE	10.857	clpX	9.39316239	lysS	9.61261261	*mnmA*	10.767	*dnaN*	12.625		
lipA	10.957	aroE	9.41256831	nuoF	10	*aspC*	10.824	*mviN*	12.776		
cysS	10.975	lepB	9.41736695	mrdA	10.0909091	*gidA*	10.889	*flgC*	12.800		
ppiD	11.217	ackA	9.42965779	mrdB	10.3207547	*pgpA*	10.898	*coaA*	12.872		
ribC	11.232	holA	9.60784314	ndk	10.4465409	*lpxB*	10.938	*pdxJ*	12.883		
pyrH	11.244	sucB	9.73789174	topA	10.7040359	*obgE*	10.957	*recC*	13.104		
speE	11.641	grpE	9.74758842	recJ	10.743662	*aroF*	11.075	*mreD*	13.306		
gidA	11.650	nrdB	9.86585366	def	10.7822581	*pyrH*	11.137	*clpP*	13.352		
adk	11.751	fliN	9.99017682	cmk	11.1548673	*fabA*	11.216	*smpB*	13.376		
mltA	11.808	dapF	10.0190476	zwf	11.270073	*mviN*	11.322	*acpS*	13.645		
recC	11.876	nuoF	10.1544715	ispF	11.3095238	*ybeB*	11.332	*polA*	13.834		
def	12.079	asd	10.2986425	lipA	11.3129252	*yjeE*	11.335	*tyrS*	13.857		
truA	12.168	fabI	10.3780488	artM	11.4501511	*cysD*	11.391	*obgE*	13.867		
pmbA	12.193	lipB	10.4172414	pgpA	11.5119454	*metK*	11.413	*ispG*	13.907		
gpt	12.207	pmbA	10.6	secY	11.585	*lpxC*	11.483	*plsB*	13.922		
tpiA	12.329	pykA	10.6272727	rbfA	11.8081181	*fabI*	11.507	*suhB*	14.023		
rlmN	12.523	polA	10.7532468	pykA	11.8636364	*accD*	11.527	*pyrE*	14.093		
ybgI	12.677	trxB	10.8474576	era	12.2166667	*mraW*	11.586	*dadX*	14.332		
ihfB	13.236	yibN	11.0512821	pyrH	12.3206107	*dapE*	11.598	*thiH*	14.385		
dapF	13.410	serS	11.1627907	acpS	12.74	*gntY*	11.797	*ftsY*	14.415		
ilvC	13.611	dapD	11.2484472	ispG	12.7938144	*znuB*	11.864	*trxA*	14.657		
topA	13.740	cysE	11.34375	dnaQ	12.9574468	*prsA*	11.983	*znuB*	14.742		
ispZ	13.777	tgt	11.3465347	fabZ	13.0175439	*glmM*	12.016	*tig*	14.777		
serS	14.060	ftsL	11.5384615	ruvC	13.610687	*lolE*	12.085	*lipB*	14.788		
bacA	14.119	ilvH	11.6153846	glmS	13.7833333	*yhcB*	12.088	*ybgI*	14.855		
yceA	14.382	yciC	11.7707317	glyQ	14.1071429	*nusB*	12.127	*pheS*	15.180		
nfuA	14.418	gpt	11.7752809	serS	14.2267442	*def*	12.159	*truA*	15.185		
murA	14.443	ispF	11.8968254	trxB	14.4011299	*rnhB*	12.182	*ycfH*	15.221		
trmE	14.453	rluC	11.986014	ftsZ	14.6025641	*tonB*	12.295	*apaH*	15.619		
yqgF	14.568	metF	12.0643564	rpe	14.6049383	*adk*	12.317	*serS*	16.005		
greA	14.930	znuB	12.1212121	gpmA	14.9333333	*gmhA*	12.346	*pgpA*	16.051		
holA	15.156	yfaE	12.14	nusG	15.1702128	*pgsA*	12.395	*fldA*	16.146		
znuB	15.247	pta	12.2508711	sodA	15.2974359	*glyQ*	12.449	*ftsZ*	16.321		
suhB	15.301	pheS	12.8457143	nuoI	15.9473684	*trpS*	12.502	*mrdB*	16.373		
miaB	15.504	rep	12.9148936	suhB	16.7	*rpsF*	12.539	*plsC*	16.373		
ftsY	15.688	trpD	13.0949367	folA	17.1612903	*ycfM*	12.610	*mraY*	16.500		
gmhA	15.741	yrbA	13.2517986	pheS	17.1828571	*ispB*	12.627	*birA*	16.566		
acpS	15.775	lpdA	13.4153005	rpiA	18.6	*efp*	12.797	*aroF*	16.671		
bamD	16.048	fldA	13.5	lolA	22.1144068	*gnd*	12.903	*aspC*	16.850		
pheS	16.112	rpiA	13.5058824	efp	22.7230769	*yfiO*	12.980	*def*	16.896		
smpB	16.389	gpmA	13.5083333	minE	23.7252747	*holA*	13.081	*lipA*	16.907		
ispG	16.791	rbfA	13.6789668	nuoB	24.3454545	*dapB*	13.580	*bioA*	17.038		
era	17.107	efp	14.0615385	coaA	28.3797468	*cdsA*	13.657	*yraP*	17.648		
engA	17.341	ispG	14.556701	crp	32.4150943	*nuoK*	13.866	*yceA*	18.633		
fliG	17.919	lipA	15.8503401	mreB	99	*cysN*	13.874	*cmk*	18.691		
zwf	18.013	pepA	16.0872483	rpsL	99	*sufC*	14.430	*waaA*	18.744		
nth	19.361	folA	16.1451613	rpoZ	99	*zur*	14.466	*lon*	18.980		
rpiA	19.422	lon	16.6984127	ptsH	99	*greA*	14.620	*erpA*	19.016		
rnfA	19.933	glmM	16.7272727	rplD	99	*lolA*	14.622	*imp*	19.187		
fmt	20.310	era	16.9388889	rpmF	99	*ilvC*	15.000	*ispB*	19.339		
fabI	20.893	fliM	17.0477941	mutH	99	*ubiE*	15.169	*ndh*	19.600		
lon	20.920	zwf	17.6131387	rplT	99	*yggX*	15.225	*pdxH*	19.854		
valS	21.832	mraY	18.2461538	atpE	99	*rbfA*	15.256	*hemE*	19.995		
fliM	22.500	flgG	18.8333333	rpsU	99	*yqgF*	15.688	*yhcB*	20.009		
mtn	24.906	glyQ	19.047619	trxA	99	*plsC*	16.057	*cdsA*	20.227		
mraY	25.175	def	19.4193548	rpmJ	99	*bcp*	16.297	*holA*	20.409		
glpE	25.219	suhB	21.6111111	rpmI	99	*yicC*	16.360	*yggX*	21.040		
glmM	26.531	nuoB	21.6181818	nadA	99	*zwf*	16.408	*fliM*	21.609		
nuoB	32.887	nth	25.655914	acpP	99	*pheS*	16.635	*nadK*	21.857		
nuoI	36.648	ftsA	29.4081633	rpmG	99	*serS*	16.692	*dapF*	21.880		
ftsA	38.837	nuoI	39.5263158	csrA	99	*pabA*	16.959	*fabI*	21.886		
fabZ	52.211	fliK	99	rplM	99	*speB*	17.182	*efp*	22.171		
erpA	61.145	rpsL	99	rplV	99	*cmk*	17.768	*nth*	22.194		
ftsJ	82.633	ptsH	99	Gene	99	*talA*	18.081	*yjgQ*	22.435		
hinT	89.623	cspC	99	rpmH	99	*yfjG*	18.280	*sucC*	22.631		
ridA	99.000	rplD	99	rplX	99	*nth*	18.382	*fabZ*	22.719		
yhhP	99.000	rpmF	99	rpsH	99	*rpiA*	18.530	*gntY*	22.945		
rpsL	99.000	rplT	99	rpsE	99	*clpP*	18.759	*kup*	23.142		
mtlA	99.000	atpE	99	infA	99	*lon*	19.080	*ftsA*	23.245		
fliR	99.000	trxA	99	rpsC	99	*engA*	19.443	*ispF*	24.681		
rplD	99.000	rpmJ	99	rplK	99	*kdtA*	19.588	*yicC*	24.839		
yheM	99.000	rpmI	99			*fmt*	20.213	*galU*	24.867		
rnhA	99.000	acpP	99			*ftsA*	20.612	*mtn*	25.875		
yigL	99.000	ybaB	99			*mrdB*	21.392	*aspA*	26.201		

rpsR	99.000
rpmF	99.000
rplT	99.000
atpE	99.000
rpsU	99.000
flgN	99.000
flhB	99.000
deoB	99.000
rpmI	99.000
aroE	99.000
gpmA	99.000
acpP	99.000
ybaB	99.000
rpmG	99.000
ftsL	99.000
csrA	99.000
rplM	99.000
rplV	99.000
serC	99.000
Gene	99.000
rpmH	99.000
rpsH	99.000
rpsE	99.000
infA	99.000
dsbA	99.000
fliI	99.000
cysK	99.000
rpsC	99.000
rpoA	99.000
rpmE	99.000
sitA	99.000
rplK	99.000

rpmG	99
csrA	99
rplM	99
rplV	99
Gene	99
fis	99
rplX	99
rpsH	99
rpsE	99
infA	99
rpsC	99
rplK	99

sucC	22.656
ftsY	22.858
dksA	24.840
tolQ	25.423
nuoB	29.623
nuoI	34.815
mraY	36.254
sufA	39.403
ycfF	45.057
kdsB	48.709
minE	52.615

rpiA	28.928
pgm	29.086
tolQ	29.991
bamD	30.185
b2619	30.258
trkA	30.644
glpE	31.181
kdsB	35.140
ruvC	35.806
minE	39.264
prsA	40.898
hinT	47.981
guaA	48.204
fmt	50.819
ftsJ	54.939
yhgN	57.625
flhD	99.000
mreB	99.000
trkH	99.000
fliR	99.000
dacA	99.000
rplT	99.000
atpE	99.000
flgN	99.000
fliS	99.000
rpmJ	99.000
lolE	99.000
rpmI	99.000
acpP	99.000
rpmG	99.000
rplV	99.000
sdaC	99.000
rpmH	99.000
rpsE	99.000
fliI	99.000
rpsC	99.000
rpmE	99.000
ugd	99.000
rpsL	99.000
yccA	99.000
rpoZ	99.000
rplD	99.000
rnhA	99.000
rpsR	99.000
rpmF	99.000
emrE	99.000
rpsU	99.000
flhB	99.000
nadA	99.000
gpmA	99.000
ybaB	99.000
rplM	99.000
serC	99.000
ybiS	99.000
Gene	99.000
rpsH	99.000
crp	99.000
infA	99.000
dsbA	99.000
wecE	99.000
rpoA	99.000
yqeI	99.000
rplK	99.000
