# Supplementary material for: Chance and necessity in the genome evolution of endosymbiotic bacteria of insects
Source: ISME J. 2017 Mar 21;11(6):1291–304. doi: 10.1038/ismej.2017.18 (PMC5437351; doi:10.1038/ismej.2017.18)
Supplement: Supplementary Table S2 [file ismej201718x2.pdf]

Table S2. Mutational spectrum of Escherichia coli evolving populations under genetic drift.

kind	position	mutation	annotation	gene	locus	description
DEL	302	_1bp	intergenic (+	thrL/thrA	b0001/b0002	thr operon leader peptide/fused aspartokinase I and homoserine dehydrogenase I
SNP	22796	G_A	G136S(GGT_	ileS	b0026	isoleucyl-tRNA synthetase
SNP	33671	G_A	G952D(GGC_	carB	b0033	carbamoyl-phosphate synthase large subunit
DEL	34112	_1bp	intergenic (+	carB/caiF	b0033/b0034	carbamoyl-phosphate synthase large subunit/DNA-binding transcriptional activator
SNP	41251	A_G	H227H(CAT_	caiT	b0040	predicted transporter
SNP	47130	A_G	M442V(ATG_	yaaU	b0045	predicted transporter
SNP	49456	A_G	N563S(AAC_	kefC	b0047	potassium:proton antiporter
SNP	51834	C_T	T199T(ACG_	rsmA	b0051	16S rRNA dimethyladenosine transferase, SAM- dependent
SNP	54597	C_T	V36I(GTC_AT	surA	b0053	peptidyl-prolyl cis-trans isomerase (PPIase)
SNP	56877	C_T	G78D(GGT_G	lptD	b0054	LPS assembly OM complex LptDE, beta-barrel component
SNP	69631	G_A	R179C(CGT_	thiP	b0067	fused thiamin transporter subunits of ABC superfamily: membrane components
SNP	69962	C_T	L68L(CTG_CT	thiP	b0067	fused thiamin transporter subunits of ABC superfamily: membrane components
SNP	70218	A_G	L235L(TTG_C	thiB	b0068	thiamin transporter subunit
SNP	97806	T_C	G466G(GGT_	murC	b0091	UDP-N-acetylmuramate:L-alanine ligase
SNP	115546	A_G	Y208C(TAT_T	ampE	b0111	predicted inner membrane protein
SNP	121896	G_A	A187T(GCT_	aceF	b0115	pyruvate dehydrogenase, dihydrolipoyltransacetylase component E2
SNP	124735	A_G	D394G(GAC_	lpd	b0116	lipoamide dehydrogenase, E3 component is part of three enzyme complexes
SNP	129203	A_G	E700G(GAA_	acnB	b0118	bifunctional aconitate hydratase 2/2- methylisocitrate dehydratase
SNP	129595	G_A	A831T(GCG_	acnB	b0118	bifunctional aconitate hydratase 2/2- methylisocitrate dehydratase
SNP	134429	C_T	intergenic (+	cueO/gcd	b0123/b0124	multicopper oxidase (laccase)/glucose dehydrogenase
SNP	135262	C_T	S536N(AGC_	gcd	b0124	glucose dehydrogenase
SNP	144358	C_T	V9V(GTG_GT	panC	b0133	pantothenate synthetase
SNP	163295	C_G	P57A(CCT_G	fhuA	b0150	ferrichrome outer membrane transporter
SNP	170068	G_A	A153V(GCC_	hemL	b0154	glutamate-1-semialdehyde aminotransferase (aminomutase)
SNP	174561	C_T	G79D(GGC_G	mtn	b0159	5'-methylthioadenosine/S-adenosylhomocysteine nucleosidase
SNP	181933	C_T	Q787Q(CAG_	glnD	b0167	uridylyltransferase
SNP	183623	C_T	G224D(GGC_	glnD	b0167	uridylyltransferase
SNP	184133	C_T	G54E(GGG_G	glnD	b0167	uridylyltransferase
SNP	184868	A_G	V94V(GTT_G	map	b0168	methionine aminopeptidase
SNP	185151	A_G	intergenic (-2	map/rpsB	b0168/b0169	methionine aminopeptidase/30S ribosomal subunit protein S2
SNP	187671	T_C	G58G(GGT_G	pyrH	b0171	uridylate kinase
SNP	202373	A_G	K535K(AAA_	dnaE	b0184	DNA polymerase III alpha subunit
SNP	207997	A_G	Q8Q(CAA_CA	tilS	b0188	tRNA(Ile)-lysidine synthetase
SNP	213167	C_T	G418S(GGT_	proS	b0194	prolyl-tRNA synthetase
INS	229775	#NAME?	coding (651/	gloB	b0212	hydroxyacylglutathione hydrolase
DEL	238047	_1bp	coding (900/	fadE	b0221	acyl coenzyme A dehydrogenase
SNP	238824	A_G	G41G(GGT_G	fadE	b0221	acyl coenzyme A dehydrogenase
SNP	243002	A_G	P216P(CCA_	yafL	b0227	predicted lipoprotein and C40 family peptidase
SNP	251688	G_A	L23L(CTG_CT	gpt	b0238	guanine-hypoxanthine phosphoribosyltransferase
INS	257855	#NAME?	intergenic (+	thrW/ykfN	b0244/b4626	tRNA-Thr/pseudogene, YdiA family
SNP	279871	C_T	R602W(CGG_	yagF	b0269	CP4-6 prophage; predicted dehydratase
SNP	302400	A_G	R600R(CGT_	yagX	b0291	predicted aromatic compound dioxygenase
INS	304918	#NAME?	intergenic (-2	matC/matB	b0292/b0293	predicted protein/cryptic Mat fimbrillin gene
INS	311442	#NAME?	pseudogene	ykgA	b0300	pseudogene, AraC family;putative regulator; Not classified; putative ARAC-type regulatory protein
SNP	313511	C_T	intergenic (-7	ykgI/ykgC	b0303/b0304	predicted protein/predicted pyridine nucleotide-disulfide oxidoreductase
DEL	316425	_1bp	intergenic (+	ykgD/ykgE	b0305/b0306	predicted DNA-binding transcriptional regulator/predicted oxidoreductase
SNP	321440	G_A	I179I(ATC_AT	betA	b0311	choline dehydrogenase, a flavoprotein
SNP	322200	C_T	V467V(GTG_	betB	b0312	betaine aldehyde dehydrogenase, NAD-dependent
SNP	325685	C_T	V452V(GTC_	betT	b0314	choline transporter of high affinity
SNP	326005	C_T	A559V(GCG_	betT	b0314	choline transporter of high affinity
SNP	326676	A_G	intergenic (+	betT/yahA	b0314/b0315	choline transporter of high affinity/c-di-GMP-specific phosphodiesterase
SNP	329731	C_T	S53S(TCG_TC	yahC	b0317	predicted inner membrane protein
SNP	329922	C_T	intergenic (-3	yahC/yahD	b0317/b0318	predicted inner membrane protein/ankyrin repeat protein
SNP	336428	A_G	D146G(GAC_	yahJ	b0324	predicted deaminase with metallo-dependent hydrolase domain
SNP	336730	C_T	P247S(CCG_T	yahJ	b0324	predicted deaminase with metallo-dependent hydrolase domain
SNP	338189	C_T	Q147*(CAA_	yahK	b0325	predicted oxidoreductase, Zn-dependent and NAD(P)-binding
SNP	338824	T_A	intergenic (+	yahK/yahL	b0325/b0326	predicted oxidoreductase, Zn-dependent and NAD(P)-binding/predicted protein
DEL	362709	_2bp	coding (578-	mhpR	b0346	DNA-binding transcriptional activator, 3HPP- binding
SNP	365730	G_A	R196H(CGT_	mhpB	b0348	2,3-dihydroxyphenylpropionate 1,2-dioxygenase
INS	374879	#NAME?	intergenic (-1	frmR/yaiO	b0357/b0358	regulator protein that represses frmRAB operon/outer membrane protein
SNP	375276	A_G	V145A(GTA_	yaiO	b0358	outer membrane protein
SNP	382118	C_T	A94V(GCG_G	tauC	b0367	taurine transporter subunit
SNP	388155	A_G	pseudogene	yaiT	b4580	predicted protein;putative structure; Not classified; putative flagellin structural protein; interrupted by IS3
SNP	388390	A_G	pseudogene	yaiT	b4580	predicted protein;putative structure; Not classified; putative flagellin structural protein; interrupted by IS3
SNP	391783	T_C	L93P(CTG_CC	sbmA	b0377	microcin B17 transporter
SNP	395048	A_G	V248A(GTA_	ddlA	b0381	D-alanine-D-alanine ligase A
SNP	398444	T_C	S99S(AGT_AC	psiF	b0384	conserved protein, PsiF family, pho regulon
DEL	402275	_1bp	intergenic (+	yaiA/aroM	b0389/b0390	predicted protein/conserved protein, AroM family; regulated by aroR
SNP	402488	A_G	N65S(AAC_A	aroM	b0390	conserved protein, AroM family; regulated by aroR
SNP	416097	A_G	N82S(AAC_A	proY	b0402	proline-specific permease
SNP	423013	C_T	T156I(ACT_A	secD	b0408	SecYEG protein translocase auxillary subunit
SNP	425029	C_T	R235C(CGT_	secF	b0409	SecYEG protein translocase auxillary subunit
SNP	426515	T_C	K122K(AAA_	tsx	b0411	nucleoside channel, receptor of phage T6 and colicin K
SNP	434536	A_G	I170T(ATC_A	dxs	b0420	1-deoxyxylulose-5-phosphate synthase, thiamine- requiring, FAD-requiring
SNP	434579	T_C	T156A(ACC_	dxs	b0420	1-deoxyxylulose-5-phosphate synthase, thiamine- requiring, FAD-requiring
SNP	441822	C_T	G251S(GGT_	cyoE	b0428	protoheme IX farnesyltransferase
SNP	442461	G_A	L38L(CTG_TT	cyoE	b0428	protoheme IX farnesyltransferase
SNP	442724	A_G	C64R(TGC_C	cyoD	b0429	cytochrome o ubiquinol oxidase subunit IV
SNP	442739	G_A	L59L(CTG_TT	cyoD	b0429	cytochrome o ubiquinol oxidase subunit IV
SNP	453184	T_C	F298L(TTT_C	clpX	b0438	ATPase and specificity subunit of ClpX-ClpP ATP- dependent serine protease
INS	453600	#NAME?	intergenic (+	clpX/lon	b0438/b0439	ATPase and specificity subunit of ClpX-ClpP ATP- dependent serine protease/DNA-binding ATP-dependent protease La
SNP	476519	A_G	F918L(TTC_C	acrB	b0462	multidrug efflux system protein
SNP	478405	A_G	L289P(CTG_	acrB	b0462	multidrug efflux system protein
SNP	479413	G_A	R358R(CGC_	acrA	b0463	multidrug efflux system
SNP	495884	A_G	D298G(GAC_	gsk	b0477	inosine/guanosine kinase
SNP	496125	A_G	V378V(GTA_	gsk	b0477	inosine/guanosine kinase
SNP	497791	C_T	A105A(GCG_	ybaL	b0478	predicted transporter with NAD(P)-binding Rossmann-fold domain
SNP	499630	C_T	intergenic (-6	fsr/ushA	b0479/b0480	predicted fosmidomycin efflux system/bifunctional UDP-sugar hydrolase/5'- nucleotidase
SNP	506229	G_A	D6D(GAC_GA	copA	b0484	copper transporter
SNP	507905	C_T	R155W(CGG_	ybaT	b0486	predicted transporter
SNP	509491	A_C	V79G(GTG_G	ybbJ	b0488	inner membrane protein that stimulates the ftsH htpX mutant suppressor activity of QmcA
SNP	510725	T_C	intergenic (-8	qmcA/ybbL	b0489/b0490	multicopy suppressor of ftsH htpX double mutant; membrane-anchored predicted protease with C-terminal cytoplasmic PHB domain/predicted transporter subunit: ATP-binding component of ABC superfamily
SNP	511679	C_T	A77V(GCG_G	ybbM	b0491	inner membrane protein, UPF0014 family
SNP	512387	T_C	T254A(ACG_	ybbN	b0492	DnaK co-chaperone, thioredoxin-like protein
SNP	514124	A_G	V170A(GTC_	tesA	b0494	multifunctional acyl-CoA thioesterase I and protease I and lysophospholipase L1
SNP	517351	C_T	A690V(GCC_	ybbP	b0496	predicted ABC transporter permease
INS	518073	#NAME?	intergenic (+	ybbP/rhsD	b0496/b0497	predicted ABC transporter permease/rhsD element protein
SNP	520270	A_G	N715S(AAC_	rhsD	b0497	rhsD element protein
SNP	520769	C_T	G881G(GGC_	rhsD	b0497	rhsD element protein
SNP	528954	C_T	A58V(GCG_G	gcl	b0507	glyoxylate carboligase
SNP	531649	A_G	T66T(ACA_A	glxR	b0509	tartronate semialdehyde reductase, NADH- dependent
SNP	535709	G_A	G93G(GGG_	ybbY	b0513	predicted uracil/xanthine transporter
SNP	544297	C_T	Q359*(CAG_	ylbE	b4572	were a pseudo gene - frameshift fixed
SNP	545709	A_G	A135A(GCA_	ybcF	b0521	predicted carbamate kinase
SNP	548437	C_T	G66S(GGT_A	lpxH	b0524	UDP-2,3-diacylglucosamine pyrophosphatase
SNP	552068	C_T	L180L(CTG_C	folD	b0529	bifunctional 5,10-methylene-tetrahydrofolate dehydrogenase/ 5,10-methylene-tetrahydrofolate cyclohydrolase
SNP	553139	A_G	Y21C(TAT_TC	sfmA	b0530	predicted fimbrial-like adhesin protein
INS	557533	+C	coding (326/	sfmH	b0533	predicted fimbrial-like adhesin protein
SNP	570818	G_A	pseudogene	nmpC	b0553	DLP12 prophage; truncated outer membrane porin (pseudogene);IS, phage, Tn; Phage or Prophage Related; outer membrane porin protein; locus of qsr prophage
INS	573318	+C	coding (187/	rzpD	b0556	DLP12 prophage; predicted murein endopeptidase
SNP	575940	A_G	T81A(ACT_G	nohD	b0560	DLP12 prophage; DNA packaging protein
SNP	588914	C_T	L241L(CTG_C	cusS	b0570	sensory histidine kinase in two-component regulatory system with CusR, senses copper ions
SNP	597622	G_A	A216A(GCG_	pheP	b0576	phenylalanine transporter
SNP	599924	A_G	F123L(TTC_C	nfsB	b0578	dihydropteridine reductase, NAD(P)H-dependent, oxygen-insensitive
SNP	612804	A_G	E1261G(GAG	entF	b0586	enterobactin synthase multienzyme complex component, ATP-dependent
SNP	613013	T_C	intergenic (+	entF/fepE	b0586/b0587	enterobactin synthase multienzyme complex component, ATP-dependent/regulator of length of O-antigen component of lipopolysaccharide chains
SNP	620331	C_T	A194V(GCC_	entC	b0593	isochorismate synthase 1
SNP	624775	A_G	K6E(AAA_GA	cstA	b0598	carbon starvation protein
SNP	625804	A_G	T349A(ACC_	cstA	b0598	carbon starvation protein
SNP	629843	G_A	R134C(CGT_	ybdM	b0601	conserved protein
SNP	632802	C_T	G213E(GGG_	dsbG	b0604	thiol:disulfide interchange protein, periplasmic
SNP	640011	C_T	G479D(GGC_	citT	b0612	citrate:succinate antiporter
SNP	655556	C_T	R141K(AGA_	ybeF	b0629	predicted DNA-binding transcriptional regulator
SNP	661116	G_A	L22L(CTG_TT	mrdB	b0634	cell wall shape-determining protein
SNP	669767	T_C	intergenic (-1	leuS/ybeL	b0642/b0643	leucyl-tRNA synthetase/conserved protein, DUF1451 family
SNP	682278	A_G	G112G(GGT_	gltI	b0655	glutamate, aspartate binding protein, periplasmic; part of GltJKLI ABC transporter
SNP	691412	T_C	noncoding (7	glnV	b0665	tRNA-Gln
SNP	692819	A_G	F409L(TTC_C	asnB	b0674	asparagine synthetase B
SNP	693321	T_C	S241S(TCA_T	asnB	b0674	asparagine synthetase B
SNP	707910	C_T	H20Y(CAT_T	seqA	b0687	regulatory protein for replication initiation
DEL	718387	_1bp	coding (894/	kdpD	b0695	fused sensory histidine kinase in two-component regulatory system with KdpE: signal sensing protein
SNP	730627	A_G	T38A(ACT_G	ybfC	b0704	predicted protein
SNP	731841	G_A	pseudogene	ybfL	b0705	predicted transposase (pseudogene);putative factor; Not classified; putative receptor protein
SNP	743135	C_T	L238L(TTG_T	ybgO	b0716	predicted fimbrial-like adhesin protein
INS	746597	#NAME?	coding (448/	ybgQ	b0718	predicted outer membrane protein
SNP	748134	T_C	S401G(AGT_	gltA	b0720	citrate synthase
SNP	751391	C_T	R207C(CGT_	sdhA	b0723	succinate dehydrogenase, flavoprotein subunit
SNP	751705	G_A	A311A(GCG_	sdhA	b0723	succinate dehydrogenase, flavoprotein subunit
SNP	754880	G_A	A437T(GCG_	sucA	b0726	2-oxoglutarate decarboxylase, thiamin-requiring
DEL	761818	_1bp	coding (969/	mngA	b0731	fused 2-O-a-mannosyl-D-glycerate specific PTS enzymes: IIA component/IIB component/IIC component
SNP	765276	C_T	D811D(GAC_	mngB	b0732	alpha-mannosidase
SNP	776956	C_T	S2S(AGC_AG	nadA	b0750	quinolinate synthase, subunit A
SNP	790344	G_A	W130*(TGG_	modA	b0763	molybdate transporter subunit
SNP	790594	T_C	S214P(TCA_C	modA	b0763	molybdate transporter subunit
MOB	795542		intergenic (-1	ybhD/ybhH	b0768/b0769	predicted DNA-binding transcriptional regulator/conserved protein
SNP	797981	C_T	A410V(GCG_	ybhI	b0770	predicted transporter
SNP	802773	T_G	intergenic (-9	ybhC/cI	b0772/_	acyl-CoA thioesterase, lipoprotein/phage lambda repressor protein CI
SNP	802776	C_G	intergenic (-9	ybhC/cI	b0772/_	acyl-CoA thioesterase, lipoprotein/phage lambda repressor protein CI
SNP	809922	A_G	I373V(ATC_G	bioF	b0776	8-amino-7-oxononanoate synthase
SNP	814536	A_G	A179A(GCT_	ybhK	b0780	predicted transferase with NAD(P)-binding Rossmann-fold domain
SNP	816398	C_T	T310T(ACC_	moaA	b0781	molybdopterin biosynthesis protein A
SNP	817723	G_A	A2T(GCA_AC	moaE	b0785	molybdopterin synthase, large subunit
SNP	819085	A_G	intergenic (+	ybhL/ybhM	b0786/b0787	inner membrane protein, UPF0005 family/inner membrane protein, UPF0005 family
SNP	831403	C_T	intergenic (-2	ybiA/dinG	b0798/b0799	conserved protein, DUF1768 family/ATP-dependent DNA helicase
INS	833087	#NAME?	coding (1593	dinG	b0799	ATP-dependent DNA helicase
SNP	833716	A_G	N15S(AAC_A	ybiB	b0800	predicted family 3 glycosyltransferase
DEL	835860	_1bp	coding (1085	ybiC	b0801	predicted dehydrogenase
SNP	836935	G_A	intergenic (-5	ybiI/ybiX	b0803/b0804	DksA-type zinc finger protein, function unknown/conserved protein, Fe(II)-dependent oxygenase superfamily
SNP	856792	T_C	Y232C(TAT_T	ybiU	b0821	predicted protein
SNP	857653	G_A	S267S(TCC_T	ybiV	b0822	sugar phosphatase; preference for fructose-1-P, ribose-5-P and glucose-6-P
INS	866839	+C	coding (1003	gsiA	b0829	glutathione transporter ATP-binding protein, ABC superfamily
SNP	868253	C_T	P140S(CCG_T	gsiB	b0830	glutathione periplasmic binding protein, ABC superfamily transporter
INS	882765	#NAME?	coding (668/	mdfA	b0842	multidrug efflux system protein
SNP	883525	A_G	V44A(GTT_G	ybjH	b0843	predicted protein
INS	899264	+C	intergenic (-2	artJ/artM	b0860/b0861	arginine binding protein, periplasmic/arginine transporter subunit
SNP	910826	C_T	A472T(GCA_	hcp	b0873	hybrid-cluster [4Fe-2S-2O] protein in anaerobic terminal reductases
SNP	920538	A_G	T589T(ACA_	macB	b0879	fused macrolide transporter subunits of ABC superfamily: ATP-binding component/membrane component
SNP	933933	A_G	Q725R(CAG_	ftsK	b0890	DNA translocase at septal ring sorting daughter chromsomes
SNP	940138	T_G	V252G(GTG_	dmsA	b0894	dimethyl sulfoxide reductase, anaerobic, subunit A
SNP	944745	C_T	V150V(GTC_	ycaD	b0898	predicted MFS-type transporter
SNP	949011	C_T	W165*(TGG_	pflA	b0902	pyruvate formate lyase activating enzyme 1

SNP	963258	C_T	P172S(CCG_	ycaI	b0913	inner membrane protein, ComEC family of competence proteins
SNP	982146	T_C	R24R(CGT_C	ycbL	b0927	predicted metal-binding enzyme
INS	987463	#NAME?	intergenic (-	asnS/pncB	b0930/b0931	asparaginyl tRNA synthetase/nicotinate phosphoribosyltransferase
SNP	997267	C_T	A118V(GCA_	elfD	b0939	predicted periplasmic pilin chaperone
INS	1002578	#NAME?	coding (207/	ycbF	b0944	predicted periplasmic pilini chaperone
INS	1003105	#NAME?	intergenic (+	ycbF/pyrD	b0944/b0945	predicted periplasmic pilini chaperone/dihydro-orotate oxidase, FMN-linked
SNP	1011629	C_T	P402S(CCG_	pqiA	b0950	paraquat-inducible membrane protein A
SNP	1016768	T_C	intergenic (-	ycbZ/matP	b0955/b0956	predicted peptidase/Ter macrodomain organizer matS-binding protein
DEL	1017402	_1bp	intergenic (+	matP/ompA	b0956/b0957	Ter macrodomain organizer matS-binding protein/outer membrane protein A (3a;II*;G;d)
SNP	1018428	G_A	T17T(ACC_A	ompA	b0957	outer membrane protein A (3a;II*;G;d)
SNP	1019952	A_G	E130E(GAA_	sxy	b0959	required for expression of CRP-S-dependent promoters
INS	1029131	#NAME?	intergenic (-	tusE/yccA	b0969/b0970	sulfurtransferase required for 2-thiolation step of mnm(5)-s(2)U34-tRNA synthesis/HflBKC-binding inner membrane protein, UPF0005 family
SNP	1031054	G_A	G164D(GGC_	hyaA	b0972	hydrogenase 1, small subunit
SNP	1031983	A_G	N102S(AAC_	hyaB	b0973	hydrogenase 1, large subunit
INS	1051935	#NAME?	coding (2639	torS	b0993	hybrid sensory histidine kinase in two-component regulatory system with TorR
SNP	1052252	C_T	P774P(CCG_	torS	b0993	hybrid sensory histidine kinase in two-component regulatory system with TorR
SNP	1053187	G_A	P463S(CCC_T	torS	b0993	hybrid sensory histidine kinase in two-component regulatory system with TorR
SNP	1054999	C_T	A105V(GCC_	torT	b0994	periplasmic sensory protein associated with the TorRS two-component regulatory system
SNP	1055026	C_T	S114L(TCA_T	torT	b0994	periplasmic sensory protein associated with the TorRS two-component regulatory system
SNP	1056909	A_G	N134S(AAC_	torC	b0996	trimethylamine N-oxide (TMAO) reductase I, cytochrome c-type subunit
SNP	1063315	T_C	S285S(AGT_	yccE	b1001	predicted protein
INS	1070970	+C	coding (319/	rutB	b1011	ureidoacrylate amidohydrolase
SNP	1074970	A_G	F780L(TTC_C	putA	b1014	fused DNA-binding transcriptional regulator/proline dehydrogenase/pyrroline-5-carboxylate dehydrogenase
SNP	1076351	G_A	G319G(GGC_	putA	b1014	fused DNA-binding transcriptional regulator/proline dehydrogenase/pyrroline-5-carboxylate dehydrogenase
INS	1095636	#NAME?	intergenic (+	ycdU/serX	b1029/b1032	predicted inner membrane protein/tRNA-Ser
INS	1095998	#NAME?	noncoding (8	serX	b1032	tRNA-Ser
SNP	1113240	C_T	V226I(GTC_A	mdtG	b1053	predicted drug efflux system
SNP	1118656	G_A	A119V(GCG_	solA	b1059	N-methyltryptophan oxidase, FAD-binding
SNP	1118855	C_T	A53T(GCT_A	solA	b1059	N-methyltryptophan oxidase, FAD-binding
SNP	1119121	T_C	intergenic (-1	solA/bssS	b1059/b1060	N-methyltryptophan oxidase, FAD-binding/biofilm regulator
SNP	1133524	A_G	Q124R(CAG_	flgG	b1078	flagellar component of cell-distal portion of basal-body rod
SNP	1134511	A_G	T175A(ACC_	flgH	b1079	flagellar protein of basal-body outer-membrane L ring
SNP	1139032	A_G	D192G(GAC_	flgL	b1083	flagellar hook-filament junction protein
SNP	1140309	C_T	L828L(TTG_T	rne	b1084	fused ribonucleaseE: endoribonuclease/RNA- binding protein/RNA degradosome binding protein
SNP	1155924	A_G	E241E(GAA_	ycfH	b1100	predicted DNAse
SNP	1160591	A_G	G94G(GGA_	hinT	b1103	purine nucleoside phosphoramidase, dadA activator protein
SNP	1165117	G_A	G184D(GGC_	ndh	b1109	respiratory NADH dehydrogenase 2/cupric reductase
SNP	1166159	A_G	T42A(ACG_G	ycfJ	b1110	predicted protein
SNP	1170555	A_G	V612A(GTA_	mfd	b1114	transcription-repair coupling factor
SNP	1170584	C_T	T602T(ACG_	mfd	b1114	transcription-repair coupling factor
SNP	1175012	T_C	A387A(GCT_	lolC	b1116	lipoprotein-releasing system transmembrane protein
SNP	1184314	A_G	T16A(ACC_G	pepT	b1127	peptidase T
SNP	1186772	C_T	G477D(GGT_	phoQ	b1129	sensory histidine kinase in two-component regulatory system with PhoP
SNP	1196751	C_T	intergenic (-	ymfE/lit	b1138/b1139	e14 prophage; predicted inner membrane protein/e14 prophage; cell death peptidase, inhibitor of T4 late gene expression
SNP	1206652	G_A	W32*(TGG_T	tfaP	b1155	e14 prophage; predicted protein
INS	1210505	+C	intergenic (-	iraM/ycgX	b1160/b1161	RpoS stabilzer during Mg starvation, anti-RssB factor/predicted protein
SNP	1219781	A_G	pseudogene	ycgH	b4491	Probable pseudogene; putative ATP-binding component of a transport system
SNP	1221510	T_C	N27D(AAT_G	ymgI	b4593	hypothetical protein
DEL	1222403	_1bp	intergenic (+	ycgI/minE	b4521/b1174	pseudogene/cell division topological specificity factor
SNP	1225808	C_T	E30E(GAG_G	pliG	b1178	predicted protein
INS	1226193	#NAME?	coding (55/2	ycgL	b1179	conserved protein
SNP	1228996	C_T	intergenic (-1	hlyE/umuD	b1182/b1183	hemolysin E/DNA polymerase V, subunit D
SNP	1233366	G_A	V2I(GTC_ATC	fadR	b1187	DNA-binding transcriptional dual regulator of fatty acid metabolism
SNP	1233622	C_T	A87V(GCG_G	fadR	b1187	DNA-binding transcriptional dual regulator of fatty acid metabolism
SNP	1237270	C_T	G425G(GGC_	dadA	b1189	D-amino acid dehydrogenase
SNP	1241502	A_G	S2P(TCT_CCT	ldcA	b1192	L,D-carboxypeptidase A
SNP	1241889	T_C	R59R(CGT_C	emtA	b1193	lytic murein endotransglycosylase E
SNP	1247581	A_G	M201T(ATG_	dhaL	b1199	dihydroxyacetone kinase, C-terminal domain
SNP	1252983	A_G	G465G(GGT_	ycgV	b1202	predicted adhesin
SNP	1261097	C_T	R69H(CGC_C	ispE	b1208	4-diphosphocytidyl-2-C-methylerythritol kinase
SNP	1276147	A_G	intergenic (-1	narX/narK	b1222/b1223	sensory histidine kinase in two-component regulatory system with NarL/nitrate/nitrite transporter
SNP	1291211	G_A	A46V(GCG_G	hns	b1237	global DNA-binding transcriptional dual regulator H-NS
SNP	1294614	A_G	S645P(TCC_	adhE	b1241	fused acetaldehyde-CoA dehydrogenase/iron- dependent alcohol dehydrogenase/pyruvate-formate lyase deactivase
MOB	1297920		intergenic (+	ychE/oppA	b1242/b1243	predicted inner membrane protein/oligopeptide transporter subunit
SNP	1299279	A_G	H291R(CAC_	oppA	b1243	oligopeptide transporter subunit
SNP	1300229	T_C	F35F(TTT_TT	oppB	b1244	oligopeptide transporter subunit
SNP	1300959	A_G	T279A(ACC_	oppB	b1244	oligopeptide transporter subunit
DEL	1311722	_1bp	coding (477/	ompW	b1256	outer membrane protein W
SNP	1321140	C_T	S198L(TCG_T	yciV	b1266	conserved protein
SNP	1323770	A_G	H600R(CAC_	yciQ	b1268	predicted inner membrane protein
DEL	1325931	_1bp	coding (408/	yciK	b1271	predicted oxoacyl-(acyl carrier protein) reductase, EmrKY-TolC system
SNP	1331393	C_T	R105C(CGC_	cysB	b1275	DNA-binding transcriptional dual regulator, O- acetyl-L-serine-binding
SNP	1331956	A_G	A292A(GCA_	cysB	b1275	DNA-binding transcriptional dual regulator, O- acetyl-L-serine-binding
SNP	1333412	C_T	P119L(CCG_	acnA	b1276	aconitate hydratase 1
SNP	1336698	T_C	V48A(GTC_G	pgpB	b1278	phosphatidylglycerophosphatase B
SNP	1339571	G_A	G142D(GGC_	pyrF	b1281	orotidine-5'-phosphate decarboxylase
SNP	1343643	C_T	G109E(GGG_	gmr	b1285	cyclic-di-GMP phosphodiesterase; csgD regulator; modulator of Rnase II stability
SNP	1346180	A_G	intergenic (-	rnb/yciW	b1286/b1287	ribonuclease II/predicted oxidoreductase
SNP	1347525	A_G	V247V(GTT_	fabI	b1288	enoyl-[acyl-carrier-protein] reductase, NADH- dependent
SNP	1359099	C_T	Q252*(CAG_	puuD	b1298	gamma-Glu-GABA hydrolase
SNP	1361292	C_T	R442*(CGA_	puuC	b1300	gamma-Glu-gamma-aminobutyraldehyde dehydrogenase, NAD(P)H-dependent
SNP	1370435	A_G	intergenic (+	ycjN/ycjO	b1310/b1311	predicted sugar transporter subunit: periplasmic- binding component of ABC superfamily/predicted sugar transporter subunit: membrane component of ABC superfamily
SNP	1371109	C_T	A221V(GCG_	ycjO	b1311	predicted sugar transporter subunit: membrane component of ABC superfamily
INS	1389979	#NAME?	coding (763/	ycjZ	b1328	predicted DNA-binding transcriptional regulator
DEL	1416324	_1bp	coding (428/	sieB	b1353	Rac prophage; phage superinfection exclusion protein
SNP	1418882	A_G	N180D(AAT_	ydaU	b1359	Rac prophage; conserved protein
DEL	1423233	_1bp	intergenic (+	ydaY/ynaA	b1366/b1368	pseudogene, Rac prophage;Phage or Prophage Related/Rac prophage; pseudogene, tail protein homology;Phage or Prophage Related; putative alpha helix protein
DEL	1429333	_2bp	coding (3059	stfR	b1372	Rac prophage; predicted tail fiber protein
SNP	1436053	G_A	A653V(GCC_	ydbK	b1378	fused predicted pyruvate-flavodoxin oxidoreductase: conserved protein/conserved protein/FeS binding protein
SNP	1437217	G_A	A265V(GCG_	ydbK	b1378	fused predicted pyruvate-flavodoxin oxidoreductase: conserved protein/conserved protein/FeS binding protein
SNP	1449937	A_G	V311A(GTC_	paaZ	b1387	fused oxepin-CoA hydrolase/3-oxo-5,6- dehydrosuberyl-CoA semialdehyde dehydrogenase
INS	1450172	+C	coding (697/	paaZ	b1387	fused oxepin-CoA hydrolase/3-oxo-5,6- dehydrosuberyl-CoA semialdehyde dehydrogenase
SNP	1451320	A_G	A34A(GCA_G	paaA	b1388	ring 1,2-phenylacetyl-CoA epoxidase subunit
SNP	1463355	T_C	pseudogene	ydbA	b4492	pseudogene, autotransporter homolog; interrupted by IS2 and IS30
SNP	1466392	A_G	pseudogene	ydbA	b4492	pseudogene, autotransporter homolog; interrupted by IS2 and IS30
SNP	1467666	A_G	pseudogene	ydbA	b4492	pseudogene, autotransporter homolog; interrupted by IS2 and IS30
SNP	1473606	C_T	Q413*(CAA_	ydbD	b1407	predicted PF10971 family periplasmic methylglyoxal resistance protein
SNP	1477986	A_G	K541K(AAA_	ynbC	b1410	predicted hydrolase
SNP	1479185	G_A	E383K(GAA_	ynbD	b1411	predicted phosphatase inner membrane protein
SNP	1509152	T_C	V91V(GTT_G	ydcS	b1440	polyhydroxybutyrate (PHB) synthase, ABC transporter periplasmic binding protein homolog
SNP	1511914	G_A	A286A(GCG_	ydcU	b1442	predicted spermidine/putrescine transporter subunit
SNP	1520319	A_G	intergenic (-	yncD/yncE	b1451/b1452	predicted iron outer membrane transporter/ATP-binding protein, periplasmic, function unknown
SNP	1536002	A_G	C26C(TGT_T	narY	b1467	nitrate reductase 2 (NRZ), beta subunit
SNP	1548021	G_A	C112Y(TGC_	fdnH	b1475	formate dehydrogenase-N, Fe-S (beta) subunit, nitrate-inducible
SNP	1548324	T_C	V213A(GTC_	fdnH	b1475	formate dehydrogenase-N, Fe-S (beta) subunit, nitrate-inducible
INS	1552874	#NAME?	coding (22/1	maeA	b1479	malate dehydrogenase, (decarboxylating, NAD- requiring) (malic enzyme)
SNP	1555318	C_T	C309Y(TGT_	ddpD	b1484	D,D-dipeptide permease system, ATP-binding component
SNP	1558083	T_C	intergenic (-	ddpB/ddpA	b1486/b1487	D-ala-D-ala transporter subunit/D-ala-D-a la transporter subunit
SNP	1560090	G_A	R71R(CGC_C	ddpX	b1488	D-ala-D-ala dipeptidase, Zn-dependent
SNP	1570185	C_T	S748S(TCG_	pqqL	b1494	predicted peptidase
SNP	1574326	A_G	F174L(TTC_C	yddB	b1495	predicted porin protein
SNP	1579291	T_C	T154A(ACC_	ydeN	b1498	conserved protein
SNP	1581112	A_G	V25A(GTT_G	safA	b1500	Two component system connector membrane protein, EvgSA to PhoQP
SNP	1582188	C_T	V509I(GTC_A	ydeP	b1501	predicted oxidoreductase
SNP	1582490	A_G	V408A(GTA_	ydeP	b1501	predicted oxidoreductase
DEL	1591351	_1bp	pseudogene	yneO	b4696	pseudogene, AidA homolog
INS	1591474	+C	pseudogene	yneO	b4696	pseudogene, AidA homolog
DEL	1594533	_1bp	pseudogene	yneO	b4696	pseudogene, AidA homolog
SNP	1601583	A_G	N104S(AAC_	lsrD	b1515	autoinducer 2 import system permease protein
SNP	1604064	G_A	A247T(GCT_	lsrF	b1517	putative autoinducer-2 (AI-2) aldolase
DEL	1615024	_1bp	coding (771/	ydeA	b1528	predicted arabinose transporter
SNP	1616154	T_C	intergenic (-	marC/marR	b1529/b1530	inner membrane protein, UPF0056 family/DNA-binding transcriptional repressor of multiple antibiotic resistance
INS	1621575	#NAME?	coding (149/	ydeI	b1536	conserved protein
SNP	1629659	T_C	pseudogene	ydfJ	b4600	pseudogene, MFS transporter family; interrupted by Qin prophage;Phage or Prophage Related; putative transport protein
SNP	1631282	T_C	pseudogene	ydfJ	b4600	pseudogene, MFS transporter family; interrupted by Qin prophage;Phage or Prophage Related; putative transport protein
SNP	1634635	A_G	pseudogene	ydfJ	b4600	pseudogene, MFS transporter family; interrupted by Qin prophage;Phage or Prophage Related; putative transport protein
SNP	1645797	G_A	pseudogene	ydfJ	b4600	pseudogene, MFS transporter family; interrupted by Qin prophage;Phage or Prophage Related; putative transport protein
SNP	1646535	T_C	pseudogene	ydfJ	b4600	pseudogene, MFS transporter family; interrupted by Qin prophage;Phage or Prophage Related; putative transport protein
SNP	1656457	C_T	A388V(GCA_	ynfE	b1587	probable selenate reductase, periplasmic
SNP	1657146	G_A	G618S(GGC_	ynfE	b1587	probable selenate reductase, periplasmic
SNP	1666440	T_C	T127A(ACC_	ynfL	b1595	predicted DNA-binding transcriptional regulator
SNP	1678839	T_C	V213A(GTG_	folM	b1606	dihydromonapterin reductase, NADPH-dependent; dihydrofolate reductase isozyme
SNP	1685476	C_T	A43T(GCA_A	fumA	b1612	fumarate hydratase (fumarase A), aerobic Class I
SNP	1687953	G_A	G293S(GGC_	ydgA	b1614	conserved protein, DUF945 family
SNP	1700311	T_C	V285A(GTG_	add	b1623	adenosine deaminase
SNP	1705030	T_C	V296A(GTT_	rsxC	b1629	electron transport complex protein required for the reduction of SoxR; predicted membrane-associated NADH oxidoreductase
DEL	1711543	_1bp	intergenic (+	dtpA/gstA	b1634/b1635	dipeptide and tripeptide permease A/glutathionine S-transferase
SNP	1711567	A_G	intergenic (+	dtpA/gstA	b1634/b1635	dipeptide and tripeptide permease A/glutathionine S-transferase
SNP	1713748	T_C	T234A(ACC_	tyrS	b1637	tyrosyl-tRNA synthetase
DEL	1719838	_3bp	coding (493-	ydhK	b1645	Efflux protein family (PET) component of predicted YdhJK efflux pump
SNP	1720760	T_C	M472T(ATG_	ydhK	b1645	Efflux protein family (PET) component of predicted YdhJK efflux pump
SNP	1721498	A_G	V128A(GTC_	sodC	b1646	superoxide dismutase, Cu, Zn
SNP	1727141	T_C	I277T(ATC_A	lhr	b1653	predicted ATP-dependent helicase
SNP	1727326	G_A	A339T(GCA_	lhr	b1653	predicted ATP-dependent helicase
SNP	1727882	T_C	V524A(GTC_	lhr	b1653	predicted ATP-dependent helicase
SNP	1738104	T_C	R323R(CGT_	ydhC	b1660	predicted transporter
SNP	1749958	C_T	V366V(GTG_	ydhV	b1673	predicted oxidoreductase
SNP	1751343	C_T	A120A(GCG_	ydhY	b1674	predicted 4Fe-4S ferridoxin-type protein
SNP	1756085	G_A	intergenic (-1	ynhG/sufE	b1678/b1679	murein L,D-transpeptidase/sulfur acceptor protein
SNP	1759563	T_C	T59A(ACG_G	sufC	b1682	component of SufBCD complex, ATP-binding component of ABC superfamily
SNP	1763081	C_T	E944K(GAA_	ydiJ	b1687	predicted FAD-linked oxidoreductase
INS	1765660	#NAME?	coding (251/	ydiJ	b1687	predicted FAD-linked oxidoreductase
INS	1781742	#NAME?	coding (1487	fadK	b1701	short chain acyl-CoA synthetase, anaerobic
SNP	1782401	G_A	P646L(CCG_	ppsA	b1702	phosphoenolpyruvate synthase
SNP	1796410	G_A	intergenic (-	pheS/pheM	b1714/b1715	phenylalanine tRNA synthetase, alpha subunit/phenylalanyl-tRNA synthetase operon leader peptide
SNP	1799034	G_A	Y254Y(TAC_	thrS	b1719	threonyl-tRNA synthetase
INS	1803261	+C	coding (48/7	ydiY	b1722	predicted outer membrane protein, acid- inducible
SNP	1808810	G_A	A218T(GCT_	ydjN	b1729	predicted transporter
SNP	1810636	T_C	intergenic (-	ydjO/cedA	b1730/b1731	predicted protein/cell division modulator
INS	1811038	#NAME?	intergenic (-1	cedA/katE	b1731/b1732	cell division modulator/catalase HPII, heme d-containing
INS	1830430	+C	coding (778/	xthA	b1749	exonuclease III
SNP	1831135	A_G	L170L(CTA_C	ydjX	b1750	inner membrane protein, TVP38/TMEM64 family
SNP	1855003	C_T	intergenic (-	ydjI/ydjJ	b1773/b1774	predicted aldolase/predicted oxidoreductase, Zn-dependent and NAD(P)-binding
SNP	1862351	C_T	G171S(GGT_	yeaE	b1781	aldo-keto reductase, methylglyoxal to acetol, NADPH-dependent
SNP	1866765	G_A	A196T(GCC_	yeaH	b1784	conserved protein
SNP	1870836	G_A	R13Q(CGG_C	yeaK	b1787	conserved protein
DEL	1876580	_1bp	intergenic (-1	yeaQ/yoaG	b1795/b1796	conserved protein, UPF0410 family/predicted protein
SNP	1879043	T_C	intergenic (-	dmlR/dmlA	b1799/b1800	DNA-binding transcriptional activator for dmlA/D-malate oxidase, NAD-dependent; probable tartrate dehydrogenase
SNP	1879301	T_C	G55G(GGT_G	dmlA	b1800	D-malate oxidase, NAD-dependent; probable tartrate dehydrogenase
SNP	1879691	A_G	T185T(ACA_	dmlA	b1800	D-malate oxidase, NAD-dependent; probable tartrate dehydrogenase
SNP	1881319	T_C	F298L(TTC_C	yeaV	b1801	predicted transporter
SNP	1890484	G_A	intergenic (-1	yoaA/yoaB	b1808/b1809	conserved protein with nucleoside triphosphate hydrolase domain/conserved protein, UPF0076 famiily
SNP	1898950	G_A	intergenic (-1	yoaE/manX	b1816/b1817	fused predicted membrane protein/conserved protein/fused mannose-specific PTS enzymes: IIA component/IIB component

SNP	1907902	A_G	P134P(CCA_(	yebQ	b1828	predicted transporter
SNP	1925235	T_C	H277R(CAC_	ptrB	b1845	protease II
INS	1933749	#NAME?	intergenic (-2	zwf/yebK	b1852/b1853	glucose-6-phosphate 1-dehydrogenase/predicted DNA-binding transcriptional regulator
SNP	1935745	T_C	I291I(ATT_AT	pykA	b1854	pyruvate kinase II
SNP	1944629	G_A	intergenic (-2	ruvC/yebC	b1863/b1864	component of RuvABC resolvasome, endonuclease/conserved protein, UPF0082 family
SNP	1952061	T_C	N724S(AAC_	torZ	b1872	trimethylamine N-oxide reductase system III, catalytic subunit
SNP	1958042	G_A	D239N(GAT_	argS	b1876	arginyl-tRNA synthetase
SNP	1967415	T_C	D265G(GAT_	tap	b1885	methyl-accepting protein IV
SNP	1979724	T_C	intergenic (-5	otsB/araH	b1897/b4460	trehalose-6-phosphate phosphatase, biosynthetic/fused L-arabinose transporter subunits of ABC superfamily: membrane components
INS	1982739	+C	coding (614/	araF	b1901	L-arabinose transporter subunit
SNP	1991403	T_C	G154G(GGA_	uvrC	b1913	excinuclease UvrABC, endonuclease subunit
SNP	2009961	A_G	pseudogene	intG	b1936	predicted defective phage integrase (pseudogene)
SNP	2029470	C_T	L24L(CTG_CT	dcm	b1961	DNA cytosine methyltransferase
SNP	2033727	G_A	M223I(ATG_	hchA	b1967	Glyoxalase III and Hsp31 molecular chaperone
SNP	2036489	T_C	F104L(TTC_C	hiuH	b1970	Hydroxyisourate hydrolase
SNP	2046400	T_C	T1413T(ACT_	yeeJ	b1978	probable adhesin
SNP	2049107	C_T	Q2316*(CAA	yeeJ	b1978	probable adhesin
SNP	2051785	G_A	A307T(GCC_	shiA	b1981	shikimate transporter
SNP	2055292	A_G	noncoding (3	asnW	b1984	tRNA-Asn
SNP	2058768	A_G	G130G(GGT_	nac	b1988	DNA-binding transcriptional dual regulator of nitrogen assimilation
SNP	2063416	G_A	intergenic (-3	yeeH/insH	b4639/b1994	pseudogene/IS5 transposase and trans-activator
DEL	2064578	_35590bp	pseudogene/	[yoeG]_[wbb	[b4640]_[b45	<i>**42 genes** [yoeG], yoeH, yoeA, insD, insC, yeeP, flu, yeeR, yeeS, yeeT, yeeU, cbtA, yeeW, yoeD, yoeF, yeeX, yeeA, sbmC, dacD, sbcB, yeeD, yeeE, plaP, yoeI, yeeY, yeeZ, yoeB, yefM, hisL, hisG, hisD, hisC, hisB, hisH, hisA, hisF, hisI, cld, ugd, gnd, insH, [wbbL]</i>
SNP	2107559	T_C	T229A(ACT_(	rfbA	b2039	glucose-1-phosphate thymidylyltransferase
SNP	2109313	T_C	S261G(AGC_	rfbB	b2041	dTDP-glucose 4,6 dehydratase, NAD(P)-binding
SNP	2117469	T_C	Y437C(TAC_T	wcaJ	b2047	predicted UDP-glucose lipid carrier transferase
SNP	2143240	G_A	A917A(GCG_	yegE	b2067	predicted diguanylate cyclase, GGDEF domain signaling protein
SNP	2144679	G_A	T29M(ACG_A	alkA	b2068	3-methyl-adenine DNA glycosylase II
SNP	2156727	C_T	T343M(ACG_	mdtC	b2076	multidrug efflux system, subunit C
SNP	2166084	T_C	V50A(GTA_G	yegS	b2086	phosphatidylglycerol kinase, metal-dependent
INS	2183092	#NAME?	intergenic (-7	rcnR/rcnA	b2105/b2106	DNA-binding transcriptional repressor of rcnA/membrane protein conferring nickel and cobalt resistance
SNP	2183183	T_C	F15L(TTC_CT	rcnA	b2106	membrane protein conferring nickel and cobalt resistance
SNP	2183989	T_C	intergenic (+	rcnA/rcnB	b2106/b2107	membrane protein conferring nickel and cobalt resistance/periplasmic modulator of Ni and Co efflux
SNP	2192433	C_T	L304L(CTG_T	metG	b2114	methionyl-tRNA synthetase
SNP	2199926	T_A	D808E(GAT_	yehI	b2118	conserved protein
DEL	2208780	_1bp	coding (332/	yehR	b2123	lipoprotein, DUF1307 family
SNP	2209908	T_C	E93E(GAA_G	yehT	b2125	predicted response regulator in two-component system withYehU
SNP	2213732	A_G	H300H(CAT_	yehX	b2129	predicted transporter subunit: ATP-binding component of ABC superfamily
SNP	2221259	G_A	T279I(ACC_A	pbpG	b2134	D-alanyl-D-alanine endopeptidase
SNP	2227264	G_A	P116S(CCT_T	dusC	b2140	tRNA-dihydrouridine synthase C
SNP	2230166	G_A	G22D(GGC_G	sanA	b2144	vancomycin high temperature exclusion protein; mutants have a defective envelope more permeable to vancomycin at 42 degrees
SNP	2247282	T_C	S114S(AGT_A	yeiH	b2158	inner membrane protein, UPF0324 family
INS	2266740	#NAME?	coding (663/	lpxT	b2174	Lipid A 1-diphosphate synthase; undecaprenyl pyrophosphate:lipid A 1-phosphate phosphotransferase
SNP	2269332	G_A	T461T(ACG_	rtn	b2176	resistance protein for phages lambda and N4, when overexpressed
DEL	2284189	_1bp	pseudogene	yejO	b2190	pseudogene, autotransporter outer membrane homology;putative transport; Not classified; putative ATP- binding component of a transport system
INS	2285778	+C	pseudogene	yejO	b2190	pseudogene, autotransporter outer membrane homology;putative transport; Not classified; putative ATP- binding component of a transport system
SNP	2288086	T_C	R121R(CGT_(	narP	b2193	DNA-binding response regulator in two-component regulatory system with NarQ or NarX
SNP	2302099	C_T	intergenic (+	eco/mqo	b2209/b2210	ecotin, a serine protease inhibitor/malate dehydrogenase, FAD/NAD(P)-binding domain
SNP	2302212	T_C	intergenic (+	eco/mqo	b2209/b2210	ecotin, a serine protease inhibitor/malate dehydrogenase, FAD/NAD(P)-binding domain
SNP	2307536	G_A	R32C(CGT_T	ada	b2213	fused DNA-binding transcriptional dual regulator/O6-methylguanine-DNA methyltransferase
SNP	2312629	G_A	D640N(GAC_	rcsD	b2216	phosphotransfer intermediate protein in two- component regulatory system with RcsBC
SNP	2322417	T_C	G146G(GGT_	atoE	b2223	short chain fatty acid transporter
DEL	2323419	_1bp	coding (87/1	atoB	b2224	acetyl-CoA acetyltransferase
SNP	2328863	C_T	pseudogene	yfaS	b4500	pseudogene, UPF0192 family; putative membrane protein
SNP	2344780	G_A	R58H(CGC_C	nrdB	b2235	ribonucleoside-diphosphate reductase 1, beta subunit, ferritin-like protein
SNP	2349724	A_G	intergenic (-1	glpT/glpA	b2240/b2241	sn-glycerol-3-phosphate transporter/sn-glycerol-3-phosphate dehydrogenase (anaerobic), large subunit, FAD/NAD(P)-binding
SNP	2353189	T_C	Y149H(TAT_(	glpC	b2243	anaerobic sn-glycerol-3-phosphate dehydrogenase, C subunit, 4Fe-4S iron-sulfur cluster
SNP	2364234	G_A	M361I(ATG_	arnB	b2253	uridine 5'-(beta-1-threo-pentapyranosyl-4-ulose diphosphate) aminotransferase, PLP-dependent
INS	2365208	+C	coding (914/	arnC	b2254	undecaprenyl phosphate-L-Ara4FN transferase
DEL	2383087	_1bp	coding (4/97	yfbL	b2271	predicted peptidase
SNP	2404881	A_G	N33D(AAC_G	alaA	b2290	valine-pyruvate aminotransferase 2
SNP	2409979	C_T	K126K(AAG_	yfbV	b2295	inner membrane protein, UPF0208 family
INS	2424382	#NAME?	coding (634/	argT	b2310	lysine/arginine/ornithine transporter subunit
SNP	2429940	T_C	Y76C(TAC_TG	folC	b2315	bifunctional folylpolyglutamate synthase/ dihydrofolate synthase
SNP	2436224	C_T	G374E(GGG_	yfcJ	b2322	predicted transporter
SNP	2440141	T_C	V253A(GTT_(	mnmC	b2324	fused 5-methylaminomethyl-2-thiouridine-forming enzyme methyltransferase and FAD-dependent demodification enzyme
SNP	2443940	G_A	A253V(GCG_	aroC	b2329	chorismate synthase
SNP	2447892	G_A	A136A(GCC_	yfcQ	b2334	predicted fimbrial-like adhesin protein
INS	2448797	+C	coding (12/5	yfcR	b2335	predicted fimbrial-like adhesin protein
SNP	2449289	T_C	N91S(AAC_A	yfcS	b2336	predicted periplasmic pilus chaperone
INS	2452974	#NAME?	intergenic (-1	yfcV/sixA	b2339/b2340	predicted fimbrial-like adhesin protein/phosphohistidine phosphatase
SNP	2456356	T_C	N10D(AAT_G	fadJ	b2341	fused enoyl-CoA hydratase and epimerase and isomerase/3-hydroxyacyl-CoA dehydrogenase
SNP	2459985	C_T	intergenic (+	fadL/yfdF	b2344/b2345	long-chain fatty acid outer membrane transporter/predicted protein
INS	2460104	+C	intergenic (+	fadL/yfdF	b2344/b2345	long-chain fatty acid outer membrane transporter/predicted protein
SNP	2467459	G_A	A369T(GCT_	gtrS	b2352	serotype-specific glucosyl transferase, CPS-53 (KpLE1) prophage
SNP	2484105	A_G	L836L(TTA_T	evgS	b2370	hybrid sensory histidine kinase in two-component regulatory system with EvgA
SNP	2484157	T_C	S854P(TCC_C	evgS	b2370	hybrid sensory histidine kinase in two-component regulatory system with EvgA
SNP	2496304	G_A	R131H(CGC_	ypdA	b2380	predicted sensory kinase in two-component system with YpdB
SNP	2498924	C_T	Q191*(CAG_	ypdC	b2382	predicted DNA-binding protein
SNP	2502349	A_G	R141R(CGT_(	ypdE	b2384	aminopeptidase
SNP	2509194	G_A	A246V(GCC_	mntH	b2392	manganese/divalent cation transporter
SNP	2513360	T_C	H566R(CAC_	yfeA	b2395	predicted diguanylate cyclase
SNP	2514376	T_C	L227L(CTA_C	yfeA	b2395	predicted diguanylate cyclase
SNP	2517177	T_C	K240K(AAA_	gltX	b2400	glutamyl-tRNA synthetase
SNP	2523319	T_C	P254P(CCA_(	yfeR	b2409	predicted DNA-binding transcriptional regulator
SNP	2529347	A_G	M221V(ATG_	cysZ	b2413	predicted inner membrane protein
SNP	2541500	G_A	G116G(GGC_	ucpA	b2426	predicted short-chain oxidoreductase
SNP	2542141	G_A	L231L(CTC_C	murR	b2427	Repressor for murPQ, MurNAc 6-P inducible
SNP	2544645	T_C	M250T(ATG_	murP	b2429	N-acetylmuramic acid permease, EIIBC component, PTS system
SNP	2547423	G_C	A116G(GCC_	yfeX	b2431	deferrrochelatase, cytoplasmic
INS	2557303	#NAME?	intergenic (+	intZ/yffL	b2442/b2443	CPZ-55 prophage; predicted integrase/CPZ-55 prophage; predicted protein
INS	2557533	+C	coding (53/6	yffL	b2443	CPZ-55 prophage; predicted protein
SNP	2567072	C_T	D164N(GAT_	eutJ	b2454	predicted chaperonin, ethanolamine utilization protein
SNP	2574154	A_G	I483T(ATT_A	maeB	b2463	fused malic enzyme predicted oxidoreductase/predicted phosphotransacetylase
SNP	2585731	C_T	L305L(CTG_T	acrD	b2470	aminoglycoside/multidrug efflux system
SNP	2586958	G_A	V714M(GTG_	acrD	b2470	aminoglycoside/multidrug efflux system
SNP	2598478	T_C	C18C(TGT_TG	hyfA	b2481	hydrogenase 4, 4Fe-4S subunit
INS	2599234	+C	coding (193/	hyfB	b2482	hydrogenase 4, membrane subunit
SNP	2603666	T_C	S61P(TCC_CC	hyfE	b2485	hydrogenase 4, membrane subunit
SNP	2607424	A_G	T13A(ACC_G	hyfH	b2488	hydrogenase 4, Fe-S subunit
SNP	2609933	A_G	I270M(ATA_	hyfR	b2491	DNA-binding transcriptional activator, formate sensing
SNP	2610593	T_C	D490D(GAT_	hyfR	b2491	DNA-binding transcriptional activator, formate sensing
SNP	2618379	A_G	intergenic (-2	upp/purM	b2498/b2499	uracil phosphoribosyltransferase/phosphoribosylaminoimidazole synthetase
SNP	2624982	C_T	G394D(GGT_	yfgF	b2503	cyclic-di-GMP phosphodiesterase, anaerobic
SNP	2628064	A_G	N172S(AAC_	yfgI	b2506	conserved protein
SNP	2630795	G_A	A167V(GCC_	guaB	b2508	IMP dehydrogenase
DEL	2635245	_1bp	coding (632/	bamB	b2512	lipoprotein required for OM biogenesis, in BamABCDE complex
SNP	2641836	G_A	R85C(CGT_TG	ndk	b2518	multifunctional nucleoside diphosphate kinase and apyrimidinic endonuclease and 3'-phosphodiesterase
SNP	2655740	C_T	R140R(CGG_	hscA	b2526	DnaK-like molecular chaperone specific for IscU
SNP	2662132	C_T	G173G(GGC_	yfhR	b2534	S9 peptidase family protein, function unknown
SNP	2671062	G_A	S284L(TCG_T	yphB	b2544	conserved protein
SNP	2681542	G_A	A397V(GCC_	glyA	b2551	serine hydroxymethyltransferase
SNP	2704762	T_C	G88G(GGA_(	rseC	b2570	RseC protein involved in reduction of the SoxR iron-sulfur cluster
SNP	2718105	C_T	S310L(TCA_T	yfiQ	b2584	inhibiting acetyltransferase for acetyl-CoA synthetase
SNP	2719711	T_C	G845G(GGT_	yfiQ	b2584	inhibiting acetyltransferase for acetyl-CoA synthetase
INS	2731759	#NAME?	coding (500/	yfiH	b2593	conserved protein, UPF0124 family
MOB	2734342		intergenic (+	bamD/raiA	b2595/b2597	lipoprotein required for OM biogenesis, in BamABCDE complex/cold shock protein associated with 30S ribosomal subunit
SNP	2738375	G_A	intergenic (-1	aroF/yfiL	b2601/b2602	3-deoxy-D-arabino-heptulosonate-7-phosphate synthase, tyrosine-repressible/lipoprotein
SNP	2747094	G_A	W351*(TGG_	yfjD	b4461	inner membrane protein, UPF0053 family
SNP	2756344	T_C	C46R(TGT_C	yfjI	b2625	CP4-57 prophage; predicted protein
SNP	2765091	G_A	C53Y(TGC_TA	yfjP	b2632	CP4-57 prophage; predicted GTP-binding protein
SNP	2769023	A_G	intergenic (+	yfjT/yfjU	b2637/b2638	CP4-57 prophage; predicted protein/CP4-57 prophage; conserved protein;Phage or Prophage Related
SNP	2775384	T_C	N1523D(AAC	ypjA	b2647	adhesin-like autotransporter
SNP	2776150	C_T	G1267G(GGC	ypjA	b2647	adhesin-like autotransporter
SNP	2784976	A_G	pseudogene	ygaQ	b4462	predicted protein; putative enzyme
SNP	2789141	G_A	A215A(GCG_	gabD	b2661	succinate-semialdehyde dehydrogenase I, NADP- dependent
SNP	2790102	C_T	G48G(GGC_G	gabT	b2662	4-aminobutyrate aminotransferase, PLP-dependent
SNP	2790579	G_A	P207P(CCG_(	gabT	b2662	4-aminobutyrate aminotransferase, PLP-dependent
SNP	2797905	T_C	intergenic (+	ygaM/nrdH	b2672/b2673	predicted protein/Hydrogen donor for NrdEF electron transport system; glutaredoxin-like protein
SNP	2805944	A_G	pseudogene	ygaY	b2681	predicted transporter (pseudogene);putative transport; Not classified; putative transport protein
SNP	2816785	G_A	G817G(GGC_	alaS	b2697	alanyl-tRNA synthetase
SNP	2820868	A_G	S42S(TCT_TC	recA	b2699	DNA strand exchange and recombination protein with protease and nuclease activity
INS	2822302	+C	coding (499/	mltB	b2701	membrane-bound lytic murein transglycosylase B
SNP	2831790	A_G	T219A(ACG_	norW	b2711	NADH:flavorubredoxin oxidoreductase
SNP	2840540	C_T	C44Y(TGC_TA	hycH	b2718	protein required for maturation of hydrogenase 3
SNP	2842426	C_T	G424S(GGC_	hycE	b2721	hydrogenase 3, large subunit
SNP	2842823	G_A	A291A(GCC_	hycE	b2721	hydrogenase 3, large subunit
SNP	2850095	G_A	A246T(GCC_	hypD	b2729	protein required for maturation of hydrogenases
SNP	2850570	G_A	W17*(TGG_T	hypE	b2730	carbamoyl dehydratase, hydrogenases 1,2,3 maturation protein
SNP	2880050	T_C	I617V(ATT_G	ygcB	b2761	Cas3 predicted helicase needed for Cascade anti- viral activity
SNP	2887104	A_G	H71R(CAC_C	queD	b2765	6-pyruvoyl tetrahydrobiopterin synthase (PTPS)
SNP	2896522	G_A	A418T(GCC_	yqcE	b2775	inner membrane protein, predicted transporter
SNP	2900205	T_C	intergenic (-1	queE/yqcG	b2777/b4682	7-carboxy-7-deazaguanine synthase; queosine biosynthesis/expressed protein
SNP	2908424	T_C	H419R(CAC_	rlmC	b2785	23S rRNA m(5)U1939 methyltransferase, SAM- dependent
SNP	2916521	C_T	V87I(GTC_AT	gudP	b2789	predicted D-glucarate transporter
INS	2923099	#NAME?	coding (192/	sdaC	b2796	predicted serine transporter
SNP	2923910	T_C	S335P(TCT_C	sdaC	b2796	predicted serine transporter
SNP	2933197	G_A	A361T(GCT_	fucK	b2803	L-fuculokinase
SNP	2940843	G_A	H339Y(CAC_	mltA	b2813	membrane-bound lytic murein transglycosylase A
INS	2942350	+C	noncoding (6	metV	b2816	tRNA-Met
SNP	2946266	A_G	V292A(GTG_	recD	b2819	exonuclease V (RecBCD complex), alpha chain
SNP	2956493	C_T	P205P(CCG_(	recC	b2822	exonuclease V (RecBCD complex), gamma chain
SNP	2956636	G_A	L158L(CTG_T	recC	b2822	exonuclease V (RecBCD complex), gamma chain
INS	2957165	+C	coding (279/	ppdC	b2823	predicted protein
SNP	2959982	G_A	F245F(TTC_T	lgt	b2828	phosphatidylglycerol-prolipoprotein diacylglyceryl transferase
SNP	2965449	G_A	G117G(GGG_	ygdQ	b2832	inner membrane protein, UPF0053 family
SNP	2974608	T_C	A303A(GCT_	lysR	b2839	DNA-binding transcriptional dual regulator
SNP	2979758	G_A	R172C(CGT_	yqeF	b2844	predicted acyltransferase
SNP	2987223	G_A	D151N(GAT_	ygeH	b2852	predictedtranscriptional regulator
INS	2994352	#NAME?	coding (177/	ygeR	b2865	novel lipoprotein, function unknown
INS	2994428	+C	coding (101/	ygeR	b2865	novel lipoprotein, function unknown
SNP	3003569	C_T	H43Y(CAT_TA	ygeY	b2872	predicted peptidase
SNP	3003901	T_C	G153G(GGT_	ygeY	b2872	predicted peptidase
INS	3009627	#NAME?	intergenic (-5	yqeC/mocA	b2876/b2877	conserved protein/CTP:molybdopterin cytidylyltransferase
SNP	3012064	G_A	E442E(GAG_	ygfK	b2878	predicted oxidoreductase, Fe-S subunit
SNP	3012889	G_A	M717I(ATG_	ygfK	b2878	predicted oxidoreductase, Fe-S subunit
SNP	3023044	G_A	M415I(ATG_	ygfQ	b4464	predicted purine permease
SNP	3023578	T_C	L38L(TTA_TT	ygfS	b2886	predicted oxidoreductase, 4Fe-4S ferredoxin-type subunit
SNP	3023900	G_A	P571S(CCG_T	ygfT	b2887	fused predicted oxidoreductase: Fe-S subunit/nucleotide-binding subunit
SNP	3030670	G_A	R98*(CGA_TG	prfB	b2891	peptide chain release factor RF-2
SNP	3051424	C_T	P169S(CCT_T	fau	b2912	conserved protein, 5-formyltetrahydrofolate cyclo-ligase family
SNP	3053912	G_A	T31T(ACC_AC	rpiA	b2914	ribose 5-phosphate isomerase, constitutive
SNP	3071788	G_A	A116A(GCC_	yggP	b4465	predicted dehydrogenase
SNP	3078743	A_T	D616E(GAT_	speA	b2938	biosynthetic arginine decarboxylase, PLP- binding

SNP	3081809	C_T	A142V(GCA_	metK	b2942	S-adenosylmethionine synthetase
SNP	3091281	G_A	G74S(GGC_A	yggU	b2953	conserved protein, UPF0235 family
SNP	3093341	T_C	S290G(AGT_	yggM	b2956	conserved protein
DEL	3095712	_1bp	coding (591/	yggN	b2958	predicted protein
SNP	3096674	T_C	N47D(AAC_G	yggL	b2959	conserved protein, DUF469 family
SNP	3096971	A_G	H187H(CAT_	trmI	b2960	tRNA m(7)G46 methyltransferase, SAM-dependent
SNP	3104125	A_G	intergenic (-	speC/yqgA	b2965/b2966	ornithine decarboxylase, constitutive/predicted inner membrane protein, DUF554 family
SNP	3105091	T_C	noncoding (4	pheV	b2967	tRNA-Phe
SNP	3115501	G_A	P153L(CCG_	glcA	b2975	glycolate transporter
SNP	3116307	A_G	intergenic (-	glcA/glcB	b2975/b2976	glycolate transporter/malate synthase G
SNP	3117751	T_C	N245S(AAT_	glcB	b2976	malate synthase G
SNP	3124197	T_C	pseudogene	yghO	b2981	pseudogene, DNA-binding transcriptional regulator homology
SNP	3128873	G_A	V22M(GTG_	yghT	b2986	predicted ATP-binding protein
INS	3129030	#NAME?	coding (221/	yghT	b2986	predicted ATP-binding protein
SNP	3129245	G_A	E146K(GAA_	yghT	b2986	predicted ATP-binding protein
SNP	3130836	A_G	V72A(GTT_G	pitB	b2987	phosphate transporter
SNP	3139861	A_G	R360R(CGT_	hybO	b2997	hydrogenase 2, small subunit
SNP	3140914	A_T	H9Q(CAT_CA	hybO	b2997	hydrogenase 2, small subunit
SNP	3152045	T_C	R248R(CGT_	dkgA	b3012	2,5-diketo-D-gluconate reductase A
SNP	3160152	G_A	N167N(AAC_	parC	b3019	DNA topoisomerase IV, subunit A
INS	3170470	+C	coding (216/	yqiA	b3031	acyl CoA esterase in vitro
DEL	3172183	_1bp	pseudogene	nudF	b3034	ADP-ribose pyrophosphatase
SNP	3176821	T_C	A89A(GCA_G	ygiD	b3039	predicted dioxygenase, LigB family
SNP	3177383	A_G	M52V(ATG_	zupT	b3040	zinc transporter
INS	3179794	+C	coding (275/	yqiC	b3042	conserved protein
SNP	3182657	G_A	pseudogene	yqiG	b3046	pseudogene; outer membrane usher homology;putative membrane; Not classified; putative membrane protein
SNP	3186448	G_A	Q58*(CAG_T	glgS	b3049	predicted glycogen synthesis protein
DEL	3188029	_1bp	coding (486/	yqiK	b3051	PHB family membrane protein, function unknown
INS	3189036	#NAME?	coding (1493	yqiK	b3051	PHB family membrane protein, function unknown
SNP	3195785	C_T	intergenic (-1	ygiF/ygiM	b3054/b3055	predicted adenylate cyclase/SH3 domain protein
SNP	3202668	A_C	intergenic (+	ttdB/ttdT	b3062/b3063	L-tartrate dehydratase, beta subunit/L-tartrate/succinate antiporte
SNP	3223480	C_T	R334C(CGC_	ygjJ	b3079	conserved protein
SNP	3229936	A_G	V207A(GTC_	rlmG	b3084	23S rRNA mG1835 methyltransferase, SAM- dependent
SNP	3245842	G_A	G47S(GGT_A	yqjG	b3102	predicted S-transferase
SNP	3254590	G_A	S67S(AGC_A	tdcF	b3113	predicted L-PSP (mRNA) endoribonuclease
INS	3260939	#NAME?	coding (807/	tdcA	b3118	DNA-binding transcriptional activator
SNP	3266929	A_G	V168A(GTC_	garR	b3125	tartronate semialdehyde reductase
SNP	3270982	G_A	V341I(GTC_A	garD	b3128	(D)-galactarate dehydrogenase
SNP	3273798	G_A	G63S(GGC_A	kbaZ	b3132	tagatose 6-phosphate aldolase 1, kbaZ subunit
SNP	3275521	T_C	pseudogene	agaW	b3134	N-acetylgalactosamine-specific enzyme IIC component of PTS, fragment (pseudogene);enzyme; Central intermediary metabolism: Amino sugars; PTS system N- acetylgalactosameine-specific IIC component 2
SNP	3281518	G_A	R190H(CGC_	agaI	b3141	galactosamine-6-phosphate isomerase
SNP	3285656	G_A	T721T(ACG_	yraJ	b3144	predicted outer membrane protein
SNP	3291072	A_G	D164G(GAT_	diaA	b3149	DnaA initiator-associating factor for replication initiation
SNP	3295666	C_T	L97L(CTG_CT	yhbT	b3157	predicted lipid carrier protein, COG3154 family
SNP	3304538	G_A	S460S(TCC_T	pnp	b3164	polynucleotide phosphorylase/polyadenylase
SNP	3313755	A_G	T147A(ACC_	argG	b3172	argininosuccinate synthetase
SNP	3320912	T_C	D235G(GAC_	ftsH	b3178	protease, ATP-dependent zinc-metallo
INS	3323489	#NAME?	intergenic (-	greA/dacB	b3181/b3182	transcript cleavage factor/D-alanyl-D-alanine carboxypeptidase
DEL	3323918	_1bp	coding (276/	dacB	b3182	D-alanyl-D-alanine carboxypeptidase
SNP	3327245	C_T	K57K(AAG_A	yhbE	b3184	predicted inner membrane permease
SNP	3330157	G_A	R340C(CGT_	murA	b3189	UDP-N-acetylglucosamine 1- carboxyvinyltransferase
INS	3331348	#NAME?	coding (136/	yrbA	b3190	predicted DNA-binding transcriptional regulator, BolA family
INS	3331774	#NAME?	coding (163/	mlaB	b3191	ABC transporter maintaining OM lipid asymmetry, cytoplasmic STAS component
DEL	3331775	_1bp	coding (162/	mlaB	b3191	ABC transporter maintaining OM lipid asymmetry, cytoplasmic STAS component
INS	3331775	#NAME?	coding (162/	mlaB	b3191	ABC transporter maintaining OM lipid asymmetry, cytoplasmic STAS component
SNP	3332238	A_G	Y112H(TAT_	mlaC	b3192	ABC transporter maintaining OM lipid asymmetry, periplasmic binding protein
SNP	3335221	G_A	A89A(GCG_G	yrbG	b3196	predicted calcium/sodium:proton antiporter
SNP	3341793	A_G	intergenic (+	ptsN/yhbJ	b3204/b3205	sugar-specific enzyme IIA component of PTS/glmZ(sRNA)-inactivating NTPase, glucosamine-6- phosphate regulated
SNP	3342953	T_C	intergenic (+	npr/yrbL	b3206/b3207	phosphohistidinoprotein-hexose phosphotransferase component of N-regulated PTS system (Npr)/predicted protein
INS	3344857	#NAME?	coding (283/	elbB	b3209	isoprenoid biosynthesis protein with amidotransferase-like domain
SNP	3351556	G_A	G718S(GGC_	gltB	b3212	glutamate synthase, large subunit
SNP	3362330	G_A	intergenic (+	yhcF/yhcG	b3219/b3220	predicted transcriptional regulator/conserved protein
SNP	3363153	T_C	V216A(GTG_	yhcG	b3220	conserved protein
SNP	3363602	G_A	A366T(GCG_	yhcG	b3220	conserved protein
SNP	3366232	G_A	R341R(CGC_	nanT	b3224	sialic acid transporter
SNP	3368618	A_G	G184G(GGT_	nanR	b3226	DNA-binding transcriptional repressor of the nan operon, induced by sialic acid
INS	3373939	+C	coding (739/	yhcM	b3232	conserved protein with nucleoside triphosphate hydrolase domain
SNP	3379896	T_C	intergenic (+	argR/yhcN	b3237/b3238	DNA-binding transcriptional dual regulator, L- arginine-binding/conserved protein
SNP	3381013	T_C	E619G(GAA_	aaeB	b3240	p-hydroxybenzoic acid efflux system component
SNP	3384682	G_A	M161I(ATG_	aaeR	b3243	transcriptional regulator for aaeXAB operon
SNP	3391730	T_C	Y249C(TAC_T	rng	b3247	ribonuclease G
SNP	3394215	G_A	G148G(GGC_	mreC	b3250	cell wall structural complex MreBCD transmembrane component MreC
DEL	3397191	_1bp	coding (822/	csrD	b3252	targeting factor for csrBC sRNA degradation
SNP	3397802	A_G	S71P(TCG_C	csrD	b3252	targeting factor for csrBC sRNA degradation
SNP	3411569	G_A	Q619Q(CAG_	acrF	b3266	multidrug efflux system protein
SNP	3424214	T_C	I100T(ATT_A	yrdA	b3279	conserved protein
SNP	3435540	G_A	T57I(ACC_AT	rpoA	b3295	RNA polymerase, alpha subunit
SNP	3442286	T_C	Q54Q(CAA_C	rplX	b3309	50S ribosomal subunit protein L24
SNP	3449533	T_C	R182R(AGA_	gspA	b3323	general secretory pathway component, cryptic
SNP	3450953	A_G	L232L(TTA_T	gspC	b3324	general secretory pathway component, cryptic
SNP	3451477	G_C	A141P(GCC_	gspD	b3325	general secretory pathway component, cryptic
SUB	3451477	1bp_CC	coding (421/	gspD	b3325	general secretory pathway component, cryptic
SNP	3456114	T_C	W138R(TGG_	gspG	b3328	pseudopilin, cryptic, general secretion pathway
SNP	3473420	G_A	A18V(GCG_G	yheV	b4551	predicted protein
SNP	3476289	G_A	G107G(GGG_	yheS	b3352	fused predicted transporter subunits of ABC superfamily: ATP-binding components
SNP	3497261	A_G	D81G(GAT_G	frlC	b4474	fructoselysine 3-epimerase
SNP	3497853	A_C	T3P(ACC_CC	frlD	b3374	fructoselysine 6-kinase
SNP	3514908	T_C	D159G(GAT_	hofQ	b3391	protein required for the utilization of DNA as a carbon source; fimbrial transporter homolog
SNP	3548440	G_A	A226T(GCC_	malT	b3418	DNA-binding transcriptional activator for the mal regulon and maltotriose-ATP-binding protein
SNP	3554361	A_G	M472V(ATG_	rtcR	b3422	sigma 54-dependent transcriptional regulator of rtcBA expression
INS	3564762	+C	coding (1238	glgX	b3431	glycogen debranching enzyme
SNP	3592026	T_C	Y72C(TAT_T	livK	b3458	leucine transporter subunit
SNP	3606105	T_C	A103A(GCA_	yhhS	b3473	inner membrane protein, predicted transporter
SNP	3606217	G_A	T66I(ACC_AT	yhhS	b3473	inner membrane protein, predicted transporter
SNP	3612020	T_C	L109S(TTA_T	nikD	b3479	nickel transporter subunit
INS	3630694	#NAME?	intergenic (+	yhiM/yhiN	b3491/b3492	inner membrane protein, DUF1323 family/predicted oxidoreductase with FAD/NAD(P)-binding domain
INS	3645699	#NAME?	intergenic (+	arsC/yhiS	b3503/b3504	arsenate reductase/pseudogene
SNP	3649664	T_C	L101P(CTC_C	dctR	b3507	predicted DNA-binding ranscriptional regulator
SNP	3659590	T_C	intergenic (-	gadW/gadX	b3515/b3516	transcriptional activator of gadA and gadBC in absence of GadX/DNA-binding transcriptional dual regulator
SNP	3660491	G_A	intergenic (-	gadX/gadA	b3516/b3517	DNA-binding transcriptional dual regulator/glutamate decarboxylase A, PLP-dependent
INS	3663217	+C	coding (651/	yhjA	b3518	predicted cytochrome C peroxidase
SNP	3663565	T_C	A101A(GCA_	yhjA	b3518	predicted cytochrome C peroxidase
SNP	3663701	T_C	E56G(GAA_G	yhjA	b3518	predicted cytochrome C peroxidase
SNP	3665353	C_T	A361A(GCC_	treF	b3519	cytoplasmic trehalase
SNP	3677959	G_A	T56T(ACC_A	dctA	b3528	C4-dicarboxylic acid, orotate and citrate transporter
SNP	3685330	A_G	S653P(TCA_C	bcsB	b3532	regulator of cellulose synthase, cyclic di-GMP binding
SNP	3689264	A_G	Y218H(TAC_	bcsA	b3533	cellulose synthase, catalytic subunit
SNP	3694595	T_C	intergenic (+	bcsG/ldrD	b3538/b4453	inner membrane protein, predicted endoglucanase, DUF3260 family/toxic polypeptide, small
SNP	3696739	G_A	P270L(CCG_	dppF	b3540	dipeptide transporter
SNP	3701088	A_G	W433R(TGG_	dppA	b3544	dipeptide transporter
SNP	3713000	G_A	intergenic (+	ghrB/yiaF	b3553/b3554	glyoxylate/hydroxypyruvate reductase B/conserved protein
SNP	3738106	T_C	V232A(GTG_	yiaK	b3575	2,3-diketo-L-gulonate reductase, NADH-dependent
SNP	3742796	G_A	G345D(GGC_	lyxK	b3580	L-xylulose kinase
INS	3761710	#NAME?	coding (695/	yibA	b3594	Predicted lyase containing HEAT-repeat
SNP	3763826	A_G	pseudogene	yibW	b4651	pseudogene, rhsA-linked
SNP	3780719	A_G	N261S(AAC_	gpmM	b3612	phosphoglycero mutase III, cofactor-independent
SNP	3786601	T_C	D210G(GAC_	kbl	b3617	glycine C-acetyltransferase
SNP	3791415	G_A	G253G(GGG_	rfaC	b3621	ADP-heptose:LPS heptosyl transferase I
SNP	3798000	T_C	L272L(CTA_C	rfaB	b3628	UDP-D-galactose:(glucosyl)lipopolysaccharide-1, 6-D-galactosyltransferase
SNP	3801476	C_T	M90I(ATG_A	rfaG	b3631	glucosyltransferase I
SNP	3802400	G_A	T126I(ACA_A	rfaQ	b3632	lipopolysaccharide core biosynthesis protein
SNP	3804104	T_C	V296A(GTC_	waaA	b3633	3-deoxy-D-manno-octulosonic-acid transferase (KDO transferase)
SNP	3820271	G_A	M128I(ATG_	recG	b3652	ATP-dependent DNA helicase
SNP	3827687	T_C	H510R(CAC_	yicI	b3656	predicted alpha-glucosidase
SNP	3847963	T_C	intergenic (-	ivbL/tisB	b3672/b4618	ilvB operon leader peptide/lexA-regulated toxic peptide involved in persister formation; membrane peptide that decreases proton motive force and ATP levels
SNP	3873773	G_A	S342F(TCT_T	gyrB	b3699	DNA gyrase, subunit B
SNP	3883180	G_A	R23H(CGC_C	tnaC	b3707	tryptophanase leader peptide
SNP	3888115	C_T	L225L(CTG_T	yidZ	b3711	predicted DNA-binding transcriptional regulator
SNP	3892611	G_A	G143E(GGA_	cbrB	b3716	inner membrane protein, creBC regulon
SNP	3893912	T_C	K54E(AAG_G	yieK	b3718	predicted 6-phosphogluconolactonase
SNP	3904573	G_A	I168I(ATC_A	pstC	b3727	phosphate transporter subunit
SNP	3919976	C_T	L112L(CTG_C	mnmG	b3741	5-methylaminomethyl-2-thiouridine modification at tRNA U34
SNP	3921055	C_T	E27K(GAA_A	mioC	b3742	FMN-binding protein MioC
INS	3926232	#NAME?	coding (239/	kup	b3747	potassium transporter
SNP	3938090	C_T	intergenic (+	rrsC/gltU	b3756/b3757	16S ribosomal RNA of rrnC operon/tRNA-Glu
SNP	3951956	C_T	V209V(GTG_	ilvY	b3773	DNA-binding transcriptional dual regulator
SNP	3953451	C_T	G268G(GGC_	ilvC	b3774	ketol-acid reductoisomerase, NAD(P)-binding
SNP	3964397	C_T	A230V(GCG_	wzzE	b3785	Entobacterial Common Antigen (ECA) polysaccharide chain length modulation protein
SNP	3972122	C_T	G389G(GGC_	wzxE	b3792	O-antigen translocase
SNP	3972451	C_T	H83H(CAC_C	rffT	b4481	TDP-Fuc4NAc:lipidIIFuc4NAc transferase
SNP	3983262	C_T	L161L(TTG_T	hemX	b3803	predicted uroporphyrinogen III methyltransferase
SNP	3985036	C_T	A137T(GCT_	hemC	b3805	hydroxymethylbilane synthase
INS	4002416	+C	intergenic (+	recQ/rhtC	b3822/b3823	ATP-dependent DNA helicase/threonine efflux pump
SNP	4010953	G_A	T8T(ACG_AC	yigP	b3834	conserved protein, SCP2 family
SNP	4042354	C_T	intergenic (-	yihA/yihI	b3865/b3866	GTP-binding protein required for normal cell division/activator of Der GTPase
DEL	4051578	_1bp	intergenic (+	typA/yihL	b3871/b3872	GTP-binding protein/predicted DNA-binding transcriptional regulator
SNP	4060606	A_G	intergenic (-	yihQ/yihR	b3878/b3879	alpha-glucosidase/predicted aldose-1-epimerase
SNP	4063293	C_T	W222*(TGG_	yihT	b3881	predicted aldolase
SNP	4064595	A_G	S95P(TCC_C	yihU	b3882	gamma-hydroxybutyrate dehydrogenase, NADH- dependent
SNP	4088183	T_C	D191G(GAC_	rhaB	b3904	rhamnulokinase
SNP	4104759	C_T	A54V(GCA_G	uspD	b3923	stress-induced protein
SNP	4106697	T_C	A63A(GCA_G	glpX	b3925	fructose 1,6-bisphosphatase II
SNP	4112687	C_T	A123T(GCT_	hslU	b3931	molecular chaperone and ATPase component of HslUV protease
SNP	4120196	T_C	T73T(ACT_A	metB	b3939	cystathionine gamma-synthase, PLP-dependent
SNP	4137048	T_C	I583T(ATC_A	pflD	b3951	predicted formate acetyltransferase 2 (pyruvate formate lyase II)
SNP	4148762	A_G	T203A(ACG_	argH	b3960	argininosuccinate lyase
SNP	4166153	A_G	R60R(CGT_C	coaA	b3974	pantothenate kinase
SNP	4201133	T_G	noncoding (5	gltV	b4008	tRNA-Glu
SNP	4207574	C_T	A264V(GCG_	aceB	b4014	malate synthase A
SNP	4225208	A_G	M49V(ATG_	pgi	b4025	glucosephosphate isomerase
SNP	4234883	G_A	A500V(GCG_	malF	b4033	maltose transporter subunit
SNP	4243819	A_G	H3R(CAC_CG	ubiC	b4039	chorismate--pyruvate lyase
SNP	4260633	C_T	intergenic (-	yjbS/aphA	b4621/b4055	hypothetical protein/acid phosphatase/phosphotransferase, class B, non-specific
SNP	4265154	G_A	G8G(GGC_G	uvrA	b4058	ATPase and DNA damage recognition protein of nucleotide excision repair excinuclease UvrABC
INS	4269249	#NAME?	intergenic (+	soxR/yjcD	b4063/b4064	DNA-binding transcriptional dual regulator, Fe-S center for redox-sensing/predicted permease
SNP	4275252	C_T	A319A(GCG_	actP	b4067	acetate transporter
SNP	4280860	T_C	Y104H(TAC_	nrfB	b4071	nitrite reductase, formate-dependent, penta-heme cytochrome c
SNP	4281308	C_T	G65G(GGC_	nrfC	b4072	formate-dependent nitrite reductase, 4Fe4S subunit
SNP	4283239	G_A	R124H(CGC_	nrfE	b4074	heme lyase (NrfEFG) for insertion of heme into c552, subunit NrfE
INS	4287686	#NAME?	intergenic (+	gltP/yjcO	b4077/b4078	glutamate/aspartate:proton symporter/conserved protein
SNP	4288716	T_C	T653A(ACC_	fdhF	b4079	formate dehydrogenase-H, selenopolypeptide subunit
SNP	4289091	A_G	W528R(TGG_	fdhF	b4079	formate dehydrogenase-H, selenopolypeptide subunit
SNP	4325404	A_G	V498A(GTT_	eptA	b4114	predicted metal dependent hydrolase

DEL	4325457	_1bp	coding (1440	eptA	b4114	predicted metal dependent hydrolase
SNP	4349100	A_G	I275T(ATC_A	cadA	b4131	lysine decarboxylase, acid-inducible
SNP	4355497	G_A	G284G(GGC_	dsbD	b4136	fused thiol:disulfide interchange protein: activator of DsbC/conserved protein
SNP	4358824	C_T	A270A(GCG_	aspA	b4139	aspartate ammonia-lyase
SNP	4373280	A_G	R74R(CGT_C	frdA	b4154	fumarate reductase (anaerobic) catalytic and NAD/flavoprotein subunit
SNP	4374714	C_T	R256C(CGT_	yjeA	b4155	EF-P-lysine34-lysine ligase
SNP	4398757	A_G	L266L(TTA_T	rnr	b4179	exoribonuclease R, RNase R
SNP	4400788	A_G	I70V(ATT_GT	rlmB	b4180	23S rRNA (Gm2251)-methyltransferase
SNP	4411014	C_T	intergenic (-	ulaG/ulaA	b4192/b419	L-ascorbate 6-phosphate lactonase/L-ascorbate-specific enzyme IIC component of PTS
SNP	4413069	C_T	A19V(GCA_G	ulaC	b4195	L-ascorbate-specific enzyme IIA component of PTS
SNP	4419228	A_G	pseudogene	ytfA	b4205	pseudogene, related to transcriptional regulators
SNP	4425612	A_G	Q65R(CAG_C	ytfH	b4212	predicted transcriptional regulator, HxlR-type, DUF24 family
SNP	4438300	A_G	V961V(GTA_	ytfN	b4221	large conserved protein, DUF490 family
DEL	4447073	_1bp	intergenic (-1	fbp/mpl	b4232/b423	fructose-1,6-bisphosphatase I/UDP-N-acetylmuramate:L-alanyl-gamma-D-glutamyl- meso-diaminopimelate ligase
SNP	4448855	T_G	N106T(AAC_	yjgA	b4234	conserved protein, ribosome-associated
INS	4471270	#NAME?	pseudogene	yjgN	b4257	inner membrane protein, DUF898 family
SNP	4473728	A_G	V472A(GTT_	valS	b4258	valyl-tRNA synthetase
SNP	4474935	C_T	G70S(GGC_A	valS	b4258	valyl-tRNA synthetase
SNP	4482671	C_T	S387N(AGC_	idnT	b4265	L-idonate and D-gluconate transporter
SNP	4491422	T_C	pseudogene	yjgX	b4575	KpLE2 phage-like element; predicted protein, C-ter fragment (pseudogene)
SNP	4503345	C_T	L125L(TTG_T	fecD	b4288	KpLE2 phage-like element; iron-dicitrate transporter subunit
SNP	4512640	C_T	G229E(GGG_	yjhF	b4296	KpLE2 phage-like element; predicted transporter
SNP	4513876	C_T	G508G(GGG_	yjhG	b4297	KpLE2 phage-like element; predicted dehydratase
SNP	4523281	G_A	intergenic (-	sgcX/yjhY	b4305/b465	KpLE2 phage-like element; predicted endoglucanase with Zn-dependent exopeptidase domain/pseudogene, novel zinc finger family
SNP	4528939	G_C	intergenic (-	nanS/nanM	b4309/b431	9-O-acetyl N-acetylneuraminic acid esterase/N-acetylneuraminic acid mutarotase
INS	4541716	#NAME?	coding (886/	gntP	b4321	fructuronate transporter
DEL	4544240	_1bp	coding (35/1	uxuB	b4323	D-mannonate oxidoreductase, NAD-dependent
INS	4548065	#NAME?	intergenic (-	yjiC/iraD	b4325/b432	predicted protein/RpoS stabilzer after DNA damage, anti-RssB factor
SNP	4556091	A_G	I355T(ATT_A	yjiM	b4335	predicted 2-hydroxyglutaryl-CoA dehydratase
SNP	4564710	C_T	pseudogene	yjiT	b4342	pseudogene
SNP	4564845	C_T	pseudogene	yjiT	b4342	pseudogene
SNP	4569673	C_T	P323P(CCG_	mcrB	b4346	5-methylcytosine-specific restriction enzyme McrBC, subunit McrB
SNP	4578853	G_A	G201S(GGC_	mrr	b4351	methylated adenine and cytosine restriction protein
SNP	4580254	A_G	Y44H(TAT_C	yjiX	b4353	conserved protein
SNP	4590564	A_G	intergenic (-	opgB/yjjA	b4359/b436	phosphoglycerol transferases I and II/conserved protein
SNP	4590910	T_C	intergenic (-	opgB/yjjA	b4359/b436	phosphoglycerol transferases I and II/conserved protein
INS	4597390	#NAME?	noncoding (8	leuV	b4368	tRNA-Leu
INS	4597511	#NAME?	noncoding (8	leuP	b4369	tRNA-Leu
INS	4597626	#NAME?	noncoding (8	leuQ	b4370	tRNA-Leu
DEL	4597627	_1bp	noncoding (7	leuQ	b4370	tRNA-Leu
SNP	4600543	C_T	H198H(CAC_	yjjG	b4374	dUMP phosphatase
INS	4600633	+C	intergenic (+	yjjG/prfC	b4374/b437	dUMP phosphatase/peptide chain release factor RF-3
SNP	4601290	T_C	Y191Y(TAT_T	prfC	b4375	peptide chain release factor RF-3
SNP	4606903	G_A	G489G(GGC_	yjjI	b4380	conserved protein
SNP	4612348	A_G	K54K(AAA_A	deoD	b4384	purine-nucleoside phosphorylase
SNP	4612459	C_T	S91S(TCC_TC	deoD	b4384	purine-nucleoside phosphorylase
SNP	4613568	C_T	R166*(CGA_	yjjJ	b4385	HipA family protein
SNP	4614026	A_G	Q318Q(CAA_	yjjJ	b4385	HipA family protein
SNP	4614061	T_C	V330A(GTG_	yjjJ	b4385	HipA family protein
SNP	4615538	C_T	G186S(GGT_	ytjB	b4387	conserved membrane protein
SNP	4621836	C_T	intergenic (-	yjjK/slt	b4391/b439	fused predicted transporter subunits of ABC superfamily: ATP-binding components/lytic murein transglycosylase, soluble
SNP	4623593	C_T	S528S(AGC_	slt	b4392	lytic murein transglycosylase, soluble
SNP	4629474	A_G	intergenic (+	creC/creD	b4399/b440	sensory histidine kinase in two-component regulatory system with CreB or PhoB, regulator of the CreBC regulon/inner membrane protein
INS	4630868	#NAME?	intergenic (+	creD/arcA	b4400/b440	inner membrane protein/DNA-binding response regulator in two-component regulatory system with ArcB or CpxA
