# Supplementary material for: Chance and necessity in the genome evolution of endosymbiotic bacteria of insects
Source: ISME J. 2017 Mar 21;11(6):1291–304. doi: 10.1038/ismej.2017.18 (PMC5437351; doi:10.1038/ismej.2017.18)
Supplement: Supplementary Table S3 [file ismej201718x3.pdf]

Table S3. Mutational spectrum of Escherichia coli belonging to the evolved population of line B evolving populations under genetic drift.

kind	position	mutation	annotation	gene	locus	lineC_t150	lineC_t200	lineC_t250	description
SNP	1055	T→C	V240A(GTG→GCG)	thrA	b0002	-	C	C	fused aspartokinase I and homoserine dehydrogenase I
SNP	6049	A→C	N137K(AAT→AAG)	yaaA	b0006	C	C	C	Peroxide resistance protein, lowers intracellular iron
SNP	6154	A→G	D102D(GAT→GAC)	yaaA	b0006	G	G	G	Peroxide resistance protein, lowers intracellular iron
SNP	11776	A→G	V4A(GTT→GCT)	yaaI	b0013	G	G	G	conserved protein, UPF0412 family
SNP	14916	T→G	V250G(GTT→GGT)	dnaJ	b0015	G	G	G	chaperone Hsp40, co-chaperone with DnaK
SNP	15367	T→C	intergenic (+69/-78)	dnaJ/insL	b0015/b0016	C	C	C	chaperone Hsp40, co-chaperone with DnaK/IS186 transposase
SNP	18012	A→G	Y150C(TAC→TGC)	nhaA	b0019	G	G	G	sodium-proton antiporter
SNP	18860	A→C	Q49P(CAA→CCA)	nhaR	b0020	-	-	C	DNA-binding transcriptional activator
SNP	31401	T→C	G195G(GGT→GGC)	carB	b0033	-	C	C	carbamoyl-phosphate synthase large subunit
SNP	31990	C→T	Q392*(CAG→TAG)	carB	b0033	T	T	T	carbamoyl-phosphate synthase large subunit
SNP	33705	A→G	K963K(AAA→AAG)	carB	b0033	G	G	G	carbamoyl-phosphate synthase large subunit
DEL	34112	Δ1bp	intergenic (+74/-188)	carB/caiF	b0033/b0034	Δ1bp	Δ1bp	Δ1bp	carbamoyl-phosphate synthase large subunit/DNA-binding transcriptional activator
SNP	34321	A→C	K8Q(AAA→CAA)	caiF	b0034	-	C	C	DNA-binding transcriptional activator
SNP	34634	T→G	L112R(CTC→CGC)	caiF	b0034	-	G	G	DNA-binding transcriptional activator
SNP	36621	T→G	T402P(ACC→CCC)	caiC	b0037	-	G	G	probable crotonobetaine/carnitine-CoA ligase
SNP	40497	C→T	G479R(GGA→AGA)	caiT	b0040	T	T	T	predicted transporter
SNP	40513	T→G	Q473H(CAA→CAC)	caiT	b0040	G	G	G	predicted transporter
SNP	44701	A→G	K174K(AAA→AAG)	fixC	b0043	G	G	G	predicted oxidoreductase with FAD/NAD(P)-binding domain
SNP	49345	G→A	G526E(GGG→GAG)	kefC	b0047	A	A	A	potassium:proton antiporter
SNP	49459	A→G	D564G(GAC→GGC)	kefC	b0047	-	-	G	potassium:proton antiporter
SNP	52576	C→T	G281S(GGT→AGT)	pdxA	b0052	T	T	T	4-hydroxy-L-threonine phosphate dehydrogenase, NAD-dependent
SNP	65330	T→G	K62Q(AAG→CAG)	polB	b0060	-	G	G	DNA polymerase II
SNP	73059	A→G	T10A(ACC→GCC)	sgrT	b4662	G	G	G	Inhibitor of glucose uptake
SNP	73144	A→G	D38G(GAC→GGC)	sgrT	b4662	-	-	G	Inhibitor of glucose uptake
DEL	73230	Δ1bp	intergenic (+67/-35)	sgrT/setA	b4662/b0070	Δ1bp	Δ1bp	Δ1bp	Inhibitor of glucose uptake/broad specificity sugar efflux system
SNP	79833	A→C	intergenic (-481/-179)	leuL/leuO	b0075/b0076	C	C	C	leu operon leader peptide/DNA-binding transcriptional activator
SNP	82291	G→T	A340S(GCC→TCC)	ilvI	b0077	T	T	T	acetolactate synthase III, large subunit
SNP	82337	A→C	E355A(GAA→GCA)	ilvI	b0077	-	-	C	acetolactate synthase III, large subunit
SNP	84251	A→C	S156R(AGC→CGC)	cra	b0080	C	C	C	DNA-binding transcriptional repressor-activator for carbon metabolism
SNP	90575	T→G	V94V(GTT→GTG)	murF	b0086	G	G	G	UDP-N-acetylmuramoyl-tripeptide:D-alanyl-D- alanine ligase
SNP	95490	A→C	K68T(AAA→ACA)	murG	b0090	C	C	C	N-acetylglucosaminyl transferase
SNP	97806	T→C	G466G(GGT→GGC)	murC	b0091	C	C	C	UDP-N-acetylmuramate:L-alanine ligase
INS	106867	+C	coding (181/390 nt)	mutT	b0099	+C	+C	+C	nucleoside triphosphate pyrophosphohydrolase, marked preference for dGTP
SNP	107521	T→G	D241A(GAT→GCT)	yacF	b0102	-	G	G	conserved protein
SNP	108624	T→G	E80A(GAG→GCG)	coaE	b0103	-	G	G	dephospho-CoA kinase
SNP	109912	A→C	K276Q(AAA→CAA)	guaC	b0104	-	C	C	GMP reductase
SNP	123097	A→C	K587T(AAA→ACA)	aceF	b0115	C	C	C	pyruvate dehydrogenase, dihydrolipoyltransacetylase component E2
SNP	128308	C→T	R402C(CGT→TGT)	acnB	b0118	T	T	T	bifunctional aconitate hydratase 2/2- methylisocitrate dehydratase
SNP	136983	T→G	intergenic (-115/-91)	gcd/hpt	b0124/b0125	-	-	G	glucose dehydrogenase/hypoxanthine phosphoribosyltransferase
SNP	139895	T→G	L184W(TTG→TGG)	yadH	b0128	-	G	G	predicted transporter subunit: membrane component of ABC superfamily
SNP	142059	C→T	M93I(ATG→ATA)	panD	b0131	-	T	T	aspartate 1-decarboxylase
SNP	145143	A→G	A34A(GCT→GCC)	panB	b0134	G	G	G	3-methyl-2-oxobutanoate hydroxymethyltransferase
SNP	149251	T→G	K567Q(AAG→CAG)	htrE	b0139	-	-	G	predicted outer membrane usher protein
SNP	164363	T→G	F413V(TTT→GTT)	fhuA	b0150	G	G	G	ferrichrome outer membrane transporter
SNP	166729	A→G	K171R(AAG→AGG)	fhuD	b0152	G	G	G	iron-hydroxamate transporter subunit
SNP	167493	T→G	L105*(TTA→TGA)	fhuB	b0153	G	G	G	fused iron-hydroxamate transporter subunits of ABC superfamily: membrane components
SNP	173166	T→C	V34V(GTA→GTG)	yadS	b0157	C	C	C	inner membrane protein, UPF0126 family
SNP	178977	T→G	L291*(TTA→TGA)	cdaR	b0162	-	G	G	DNA-binding transcriptional regulator for gar and gud operons; carbohydrate diacid regulator
SNP	183764	T→G	E177A(GAA→GCA)	glnD	b0167	G	G	G	uridylyltransferase
SNP	185151	A→G	intergenic (-2/-366)	map/rpsB	b0168/b0169	G	G	G	methionine aminopeptidase/30S ribosomal subunit protein S2
SNP	185475	A→G	intergenic (-326/-42)	map/rpsB	b0168/b0169	-	G	G	methionine aminopeptidase/30S ribosomal subunit protein S2
SNP	186422	C→G	intergenic (+180/-78)	rpsB/tsf	b0169/b0170	-	G	G	30S ribosomal subunit protein S2/protein chain elongation factor EF-Ts
SNP	190229	A→C	I356L(ATC→CTC)	dxr	b0173	C	C	C	1-deoxy-D-xylulose 5-phosphate reductoisomerase
SNP	193861	T→G	I97M(ATT→ATG)	bamA	b0177	-	G	G	outer membrane protein assembly factor, forms pores; required for OM biogenesis; in BamABCDE OM protein complex
SNP	199620	C→T	A210A(GCC→GCT)	lpxB	b0182	T	T	T	tetraacyldisaccharide-1-P synthase
SNP	201665	C→T	G299G(GGC→GGT)	dnaE	b0184	T	T	T	DNA polymerase III alpha subunit
DEL	210777	Δ1bp	coding (302/423 nt)	yaeJ	b0191	-	-	Δ1bp	petidyl-tRNA hydrolase, ribosome-attached; stalled ribosome rescue factor
SNP	213397	A→G	V341A(GTA→GCA)	proS	b0194	-	G	G	prolyl-tRNA synthetase
SNP	217374	A→C	G305G(GGT→GGG)	metN	b0199	-	C	C	DL-methionine transporter subunit
SNP	218862	C→T	A91A(GCC→GCT)	gmhB	b0200	-	-	T	D,D-heptose 1,7-bisphosphate phosphatase
DEL	224680	Δ1bp	intergenic (+33/-130)	aspU/dkgB	b0206/b0207	Δ1bp	Δ1bp	Δ1bp	tRNA-Asp/2,5-diketo-D-gluconate reductase B
SNP	227793	T→G	H75Q(CAT→CAG)	yafE	b0210	-	G	G	predicted S-adenosyl-L-methionine-dependent methyltransferase
SNP	227847	G→A	R93R(AGG→AGA)	yafE	b0210	A	A	A	predicted S-adenosyl-L-methionine-dependent methyltransferase
SNP	229272	A→C	V109V(GTT→GTG)	mltD	b0211	-	-	C	predicted membrane-bound lytic murein transglycosylase D
SNP	233582	T→G	V202G(GTG→GGG)	yafT	b0217	-	G	G	lipoprotein
SNP	235076	A→C	F238V(TTT→GTT)	yafV	b0219	C	C	C	predicted C-N hydrolase family amidase, NAD(P)- binding
SNP	237595	A→C	V451G(GTT→GGT)	fadE	b0221	C	C	C	acyl coenzyme A dehydrogenase
SNP	237628	A→C	L440R(CTC→CGC)	fadE	b0221	-	C	C	acyl coenzyme A dehydrogenase
SNP	240454	T→G	L162R(CTG→CGG)	yafJ	b0223	-	G	G	predicted amidotransfease
SNP	245095	T→G	pseudogene (646/1740 nt)	lfhA	b0229	G	G	G	pseudogene, flagellar system protein, promoterless fragment; flagellar biosynthesis
SNP	247340	A→C	Y267S(TAT→TCT)	dinB	b0231	C	-	-	DNA polymerase IV
DEL	248310	Δ1bp	coding (367/399 nt)	yafO	b0233	-	-	Δ1bp	mRNA interferase toxin of the YafO-YafN toxin- antitoxin system
INS	250811	+C	coding (357/1266 nt)	pepD	b0237	+C	+CC	+CC	aminoacyl-histidine dipeptidase (peptidase D)
SNP	252983	T→G	F272V(TTC→GTC)	frsA	b0239	-	-	G	fermentation-respiration switch protein; PTS Enzyme IIA(Glc)-binding protein; pNP-butyrate esterase activity
SNP	266347	A→G	pseudogene (1203/1978 nt)	insN	b4587	G	G	-	pseudogene, IS911 transposase A;IS, phage, Tn; Transposon-related functions; extrachromosomal; transposon related
SNP	268307	A→C	pseudogene (579/1131 nt)	ykfC	b0258	C	C	C	CP4-6 prophage; conserved protein;Phage or Prophage Related
SNP	270277	G→A	G29D(GGC→GAC)	mmuP	b0260	A	A	A	CP4-6 prophage; predicted S-methylmethionine transporter
SNP	274902	A→G	pseudogene (328/339 nt)	insX	b4505	G	G	G	pseudogene, predicted IS3 family transposase
SNP	275094	A→G	pseudogene (136/339 nt)	insX	b4505	G	G	G	pseudogene, predicted IS3 family transposase
SNP	278655	A→G	E196E(GAA→GAG)	yagF	b0269	G	-	G	CP4-6 prophage; predicted dehydratase
SNP	281690	A→G	N12S(AAC→AGC)	yagH	b0271	G	G	G	CP4-6 prophage; predicted xylosidase/arabinosidase
SNP	284429	A→C	I248M(ATT→ATG)	argF	b0273	-	C	C	ornithine carbamoyltransferase 2, chain F; CP4-6 prophage
SNP	289458	A→G	V70A(GTG→GCG)	yagM	b0279	G	G	G	CP4-6 prophage; predicted protein
SNP	289687	T→G	intergenic (-21/+319)	yagM/yagN	b0279/b0280	-	-	G	CP4-6 prophage; predicted protein/CP4-6 prophage; predicted protein
SNP	292771	T→G	pseudogene (20/543 nt)	yagP	b4694	-	-	G	pseudogene, LysR family, fragment;putative regulator; Not classified; putative transcriptional regulator LYSR-type
SNP	296881	T→G	D187A(GAT→GCT)	paoA	b0286	-	-	G	PaoABC aldehyde oxidoreductase, 2Fe-2S subunit
SNP	297394	T→C	H16R(CAC→CGC)	paoA	b0286	C	C	C	PaoABC aldehyde oxidoreductase, 2Fe-2S subunit
SNP	298425	A→C	I190L(ATT→CTT)	yagU	b0287	C	C	C	inner membrane protein, DUF1440 family
SNP	299570	A→G	V168A(GTA→GCA)	yagV	b0289	G	G	G	conserved protein
SNP	300142	T→G	N515H(AAC→CAC)	yagW	b0290	-	-	G	predicted receptor
SNP	304597	T→C	G99G(GGA→GGG)	matC	b0292	C	C	C	predicted protein
INS	304918	+G	intergenic (-25/+33)	matC/matB	b0292/b0293	+G	+G	+G	predicted protein/cryptic Mat fimbrillin gene
SNP	306826	A→G	intergenic (-671/-153)	matA/ykgL	b0294/b0295	G	G	G	predicted regulator/predicted protein
SNP	313511	C→T	intergenic (-77/+32)	ykgI/ykgC	b0303/b0304	T	T	T	predicted protein/predicted pyridine nucleotide-disulfide oxidoreductase
SNP	316279	A→C	intergenic (+331/-247)	ykgD/ykgE	b0305/b0306	C	C	C	predicted DNA-binding transcriptional regulator/predicted oxidoreductase
SNP	316755	A→C	E77A(GAA→GCA)	ykgE	b0306	-	-	C	predicted oxidoreductase
SNP	325685	C→T	V452V(GTC→GTT)	betT	b0314	T	T	T	choline transporter of high affinity
SNP	331747	T→G	F35V(TTT→GTT)	yahF	b0320	G	G	G	predicted acyl-CoA synthetase with NAD(P)- binding domain and succinyl-CoA synthetase domain
SNP	333220	T→G	L10W(TTG→TGG)	yahG	b0321	-	-	G	conserved protein
SNP	333310	G→A	G40E(GGG→GAG)	yahG	b0321	A	A	A	conserved protein
SNP	335183	A→G	H51R(CAC→CGC)	yahI	b0323	G	G	G	carbamate kinase-like protein
SNP	336472	T→C	Y161H(TAC→CAC)	yahJ	b0324	-	C	C	predicted deaminase with metallo-dependent hydrolase domain
SNP	339542	A→G	Y167C(TAC→TGC)	yahL	b0326	G	G	G	predicted protein
SNP	346685	T→C	W202R(TGG→CGG)	prpD	b0334	C	C	C	2-methylcitrate dehydratase
SNP	346759	C→T	G226G(GGC→GGT)	prpD	b0334	T	T	T	2-methylcitrate dehydratase
SNP	347787	T→G	I72S(ATT→AGT)	prpE	b0335	-	G	G	propionate--CoA ligase
INS	351579	+G	coding (557/1299 nt)	codA	b0337	+G	+G	+G	cytosine/isoguanine deaminase
SNP	353604	T→G	intergenic (-47/-371)	cynR/cynT	b0338/b0339	-	-	G	transcriptional activator of cyn operon; autorepressor/carbonic anhydrase
SNP	354970	G→A	A38T(GCC→ACC)	cynX	b0341	A	A	A	predicted cyanate transporter
SNP	359046	C→T	W709*(TGG→TGA)	lacZ	b0344	T	T	T	beta-D-galactosidase
SNP	360422	A→G	L251L(TTG→CTG)	lacZ	b0344	G	G	G	beta-D-galactosidase
SNP	361341	A→C	L305*(TTA→TGA)	lacI	b0345	-	C	C	DNA-binding transcriptional repressor
SNP	363495	T→G	P6P(CCT→CCG)	mhpA	b0347	G	G	G	3-(3-hydroxyphenyl)propionate hydroxylase
SNP	366646	A→G	T186A(ACC→GCC)	mhpC	b0349	G	G	G	2-hydroxy-6-ketonona-2,4-dienedioic acid hydrolase
SNP	373346	T→G	T365P(ACC→CCC)	frmA	b0356	-	G	G	alcohol dehydrogenase class III/glutathione- dependent formaldehyde dehydrogenase
DEL	374880	Δ3bp	intergenic (-132/+54)	frmR/yaiO	b0357/b0358	-	-	Δ3bp	regulator protein that represses frmRAB operon/outer membrane protein
SNP	382556	A→G	D240G(GAC→GGC)	tauC	b0367	-	G	G	taurine transporter subunit
SNP	396084	T→C	intergenic (-294/-169)	ddlA/iraP	b0381/b0382	C	C	C	D-alanine-D-alanine ligase A/anti-RssB factor, RpoS stabilzer during Pi starvation; anti-adapter protein
SNP	399965	T→G	I183L(ATC→CTC)	proC	b0386	G	G	G	pyrroline-5-carboxylate reductase, NAD(P)- binding
SNP	404939	C→T	R19W(CGG→TGG)	mak	b0394	T	T	T	manno(fructo)kinase
SNP	405254	T→C	W124R(TGG→CGG)	mak	b0394	C	C	C	manno(fructo)kinase
SNP	406633	A→C	F239C(TTT→TGT)	araJ	b0396	-	C	C	arabinose-inducible predicted transporter, MFS family
SNP	408194	C→T	Q809Q(CAG→CAA)	sbcC	b0397	T	T	T	exonuclease, dsDNA, ATP-dependent
SNP	416097	A→G	N82S(AAC→AGC)	proY	b0402	G	G	G	proline-specific permease
SNP	417131	A→G	I427V(ATC→GTC)	proY	b0402	-	G	G	proline-specific permease
SNP	418174	C→T	R264C(CGT→TGT)	malZ	b0403	T	T	T	maltodextrin glucosidase
SNP	418185	T→C	G267G(GGT→GGC)	malZ	b0403	-	C	C	maltodextrin glucosidase
SNP	421993	C→T	N330N(AAC→AAT)	tgt	b0406	T	T	T	tRNA-guanine transglycosylase
SNP	426515	T→C	K122K(AAA→AAG)	tsx	b0411	C	C	C	nucleoside channel, receptor of phage T6 and colicin K
SNP	428020	T→C	V51A(GTT→GCT)	nrdR	b0413	C	C	C	conserved protein
SNP	432755	A→C	V83G(GTG→GGG)	yajO	b0419	-	C	C	2-carboxybenzaldehyde reductase, function unknown
SNP	434579	T→C	T156A(ACC→GCC)	dxs	b0420	C	C	C	1-deoxyxylulose-5-phosphate synthase, thiamine- requiring, FAD-requiring
SNP	436617	A→G	T68A(ACC→GCC)	thiI	b0423	G	G	G	tRNA s(4)U8 sulfurtransferase
SNP	439723	C→T	V58V(GTC→GTT)	yajQ	b0426	T	T	T	Phi6 host factor; binds ATP and GTP
SNP	441387	T→G	L49F(TTA→TTC)	yajR	b0427	-	-	G	predicted transporter
SNP	443269	C→T	A87T(GCT→ACT)	cyoC	b0430	T	T	T	cytochrome o ubiquinol oxidase subunit III
SNP	443551	T→C	E653G(GAG→GGG)	cyoB	b0431	-	-	C	cytochrome o ubiquinol oxidase subunit I
SNP	455622	A→C	K623T(AAA→ACA)	lon	b0439	-	C	C	DNA-binding ATP-dependent protease La
SNP	455945	A→C	I731L(ATT→CTT)	lon	b0439	C	C	C	DNA-binding ATP-dependent protease La
SNP	458732	A→G	intergenic (+79/-72)	ppiD/ybaV	b0441/b0442	G	G	G	periplasmic folding chaperone, has an inactive PPIase domain/conserved protein, ComEA homolog
SNP	458999	A→C	N66H(AAT→CAT)	ybaV	b0442	-	C	C	conserved protein, ComEA homolog
SNP	459384	A→G	D39G(GAC→GGC)	fadM	b0443	G	G	G	long-chain acyl-CoA thioesterase III
DEL	473978	Δ1bp	coding (141/471 nt)	ylaC	b0458	Δ1bp	Δ1bp	Δ1bp	inner membrane protein, DUF1449 family
SNP	479726	A→C	F254C(TTC→TGC)	acrA	b0463	-	-	C	multidrug efflux system
SNP	485851	G→T	A35S(GCG→TCG)	ybaN	b0468	T	T	T	inner membrane protein, DUF454 family
SNP	486408	A→G	S44G(AGC→GGC)	apt	b0469	G	G	G	adenine phosphoribosyltransferase
SNP	487031	C→T	L25L(CTG→TTG)	dnaX	b0470	T	T	T	DNA polymerase III/DNA elongation factor III, tau and gamma subunits
SNP	488053	G→A	L365L(CTG→CTA)	dnaX	b0470	A	A	A	DNA polymerase III/DNA elongation factor III, tau and gamma subunits
SNP	489062	T→C	G40G(GGT→GGC)	ybaB	b0471	C	C	C	conserved protein
SNP	489106	T→C	V55A(GTA→GCA)	ybaB	b0471	C	C	C	conserved protein
SNP	493512	C→T	P197P(CCC→CCT)	hemH	b0475	T	T	T	ferrochelatase
SNP	497791	C→T	A105A(GCG→GCA)	ybaL	b0478	T	T	T	predicted transporter with NAD(P)-binding Rossmann-fold domain
SNP	497986	A→G	G40G(GGT→GGC)	ybaL	b0478	-	G	G	predicted transporter with NAD(P)-binding Rossmann-fold domain
SNP	498564	A→C	S334A(TCT→GCT)	fsr	b0479	-	-	C	predicted fosmidomycin efflux system
SNP	499498	T→C	T22T(ACA→ACG)	fsr	b0479	C	C	C	predicted fosmidomycin efflux system
SNP	499541	T→G	Q8P(CAG→CCG)	fsr	b0479	-	G	G	predicted fosmidomycin efflux system
SNP	501206	A→C	N476H(AAC→CAC)	ushA	b0480	C	C	C	bifunctional UDP-sugar hydrolase/5'- nucleotidase
SNP	503408	T→C	R108R(CGT→CGC)	ybaQ	b0483	C	C	C	predicted DNA-binding transcriptional regulator
SNP	506887	T→G	I127S(ATT→AGT)	ybaS	b0485	-	-	G	glutaminase
DEL	508007	Δ1bp	coding (565/1293 nt)	ybaT	b0486	Δ1bp	Δ1bp	Δ1bp	predicted transporter
SNP	508864	A→G	N2S(AAC→AGC)	cueR	b0487	-	-	G	DNA-binding transcriptional activator of copper- responsive regulon genes
SNP	511971	T→G	I174M(ATT→ATG)	ybbM	b0491	-	-	G	inner membrane protein, UPF0014 family
SNP	512040	A→G	L197L(TTA→TTG)	ybbM	b0491	G	G	G	inner membrane protein, UPF0014 family
INS	518073	+T	intergenic (+376/-54)	ybbP/rhsD	b0496/b0497	+T	+T	+T	predicted ABC transporter permease/rhsD element protein
SNP	518379	A→G	T85A(ACG→GCG)	rhsD	b0497	G	G	G	rhsD element protein
SNP	523727	A→C	pseudogene (222/491 nt)	ybbD	b0501	C	C	C	pseudogene
SNP	524991	T→C	intergenic (+491/+7)	ylbI/selU	b4632/b0503	C	C	C	pseudogene, Rhs family/tRNA 2-selenouridine synthase, selenophosphate- dependent
SNP	528965	A→G	T62A(ACC→GCC)	gcl	b0507	G	G	G	glyoxylate carboligase
SNP	529180	T→G	V133V(GTT→GTG)	gcl	b0507	-	-	G	glyoxylate carboligase
SNP	534503	T→C	V164A(GTA→GCA)	allB	b0512	C	C	C	allantoinase
SNP	540046	T→G	E38A(GAA→GCA)	allC	b0516	-	-	G	allantoate amidohydrolase
SNP	540114	C→T	W15*(TGG→TGA)	allC	b0516	T	T	T	allantoate amidohydrolase
SNP	541173	A→C	A19A(GCT→GCG)	allD	b0517	C	C	C	ureidoglycolate dehydrogenase
SNP	543161	C→T	P539L(CCA→CTA)	fdrA	b0518	T	T	T	multicopy suppressor of dominant negative ftsH mutations; predicted acyl-CoA synthetase with NAD(P)- binding Rossmann-fold domain
SNP	546565	A→C	L299R(CTG→CGG)	purK	b0522	C	C	C	N5-carboxyaminoimidazole ribonucleotide synthase

SNP	548883	T→G	T141P(ACC→CCC)	ppiB	b0525	G	G	G	peptidyl-prolyl cis-trans isomerase B (rotamase B)
SNP	550576	A→G	N367S(AAC→AGC)	cysS	b0526	G	G	G	cysteinyl-tRNA synthetase
SNP	569031	A→C	L70F(TTA→TTC)	quuD	b0551	-	C	C	DLP12 prophage; predicted antitermination protein
SNP	572708	A→C	K77T(AAA→ACA)	rrrD	b0555	C	C	C	DLP12 prophage; predicted lysozyme
SNP	576479	T→G	pseudogene (260/744 nt)	aaaD	b4634	G	G	G	DLP12 prophage; predicted tail fiber assembly protein (pseudogene);Phage or Prophage Related
SNP	576921	T→G	pseudogene (702/744 nt)	aaaD	b4634	-	G	G	DLP12 prophage; predicted tail fiber assembly protein (pseudogene);Phage or Prophage Related
SNP	578302	T→G	intergenic (+376/-245)	tfaX/appY	b0563/b0564	-	G	G	pseudogene, DLP12 prophage;Phage or Prophage Related/DNA-binding global transcriptional activator; DLP12 prophage
SNP	579756	T→G	Q248H(CAA→CAC)	ompT	b0565	G	G	G	DLP12 prophage; outer membrane protease VII (outer membrane protein 3b)
SNP	580908	A→C	intergenic (-409/-15)	ompT/pauD	b0565/b4635	C	C	C	DLP12 prophage; outer membrane protease VII (outer membrane protein 3b)/tRNA-OTHER
SNP	590201	A→G	Y37H(TAC→CAC)	cusR	b0571	G	G	G	DNA-binding response regulator in two-component regulatory system with CusS
SNP	590646	A→C	N61H(AAT→CAT)	cusC	b0572	-	C	C	copper/silver efflux system, outer membrane component
SNP	598592	T→G	E313A(GAG→GCG)	mscM	b0577	G	G	G	mechanosensitive channel protein, miniconductance
DEL	600982	Δ1bp	coding (84/249 nt)	ybdJ	b0580	Δ1bp	Δ1bp	Δ1bp	predicted inner membrane protein
SNP	601099	C→T	intergenic (-34/+32)	ybdJ/ybdK	b0580/b0581	T	T	T	predicted inner membrane protein/weak gamma-glutamyl:cysteine ligase
SNP	606469	T→G	K298Q(AAA→CAA)	fepA	b0584	G	G	G	iron-enterobactin outer membrane transporter
SNP	614728	A→C	F113C(TTT→TGT)	fepC	b0588	-	C	C	iron-enterobactin transporter subunit
SNP	616089	T→C	I323V(ATC→GTC)	fepD	b0590	-	C	C	iron-enterobactin transporter subunit
SNP	618132	T→C	W323R(TGG→CGG)	entS	b0591	C	C	C	enterobactin exporter, iron-regulated
SNP	629619	A→C	V208V(GTT→GTG)	ybdM	b0601	C	C	C	conserved protein
SNP	632821	T→G	K207Q(AAA→CAA)	dsbG	b0604	-	-	G	thiol:disulfide interchange protein, periplasmic
SNP	642780	A→G	V41A(GTC→GCC)	citX	b0614	G	G	G	apo-citrate lyase phosphoribosyl-dephospho-CoA transferase
SNP	644733	A→G	A208A(GCT→GCC)	citE	b0616	G	G	G	citrate lyase, citryl-ACP lyase (beta) subunit
SNP	647509	A→G	T137A(ACC→GCC)	dpiB	b0619	-	G	G	sensory histidine kinase in two-component regulatory system with citB
SNP	649988	T→G	I283L(ATT→CTT)	dcuC	b0621	-	G	G	anaerobic C4-dicarboxylate transport
SNP	650732	C→T	G35S(GGC→AGC)	dcuC	b0621	T	T	T	anaerobic C4-dicarboxylate transport
SNP	650825	A→G	F4L(TTC→CTC)	dcuC	b0621	-	G	G	anaerobic C4-dicarboxylate transport
SNP	655395	A→G	S195P(TCC→CCC)	ybeF	b0629	G	G	G	predicted DNA-binding transcriptional regulator
SNP	658673	C→T	D53N(GAC→AAC)	dacA	b0632	T	T	T	D-alanyl-D-alanine carboxypeptidase (penicillin- binding protein 5)
SNP	665284	T→G	N52T(AAC→ACC)	nadD	b0639	G	G	G	nicotinic acid mononucleotide adenylyltransferase, NAD(P)-dependent
SNP	666711	A→C	D82E(GAT→GAG)	lptE	b0641	C	C	C	LPS assembly OM complex LptDE, lipoprotein component
SNP	676859	A→C	I467M(ATT→ATG)	hscC	b0650	C	C	C	Hsp70 family chaperone Hsc62, binds to RpoD and inhibits transcription
SNP	677325	A→G	V312A(GTG→GCG)	hscC	b0650	G	G	G	Hsp70 family chaperone Hsc62, binds to RpoD and inhibits transcription
INS	685462	+CAC	coding (286/1539 nt)	lnt	b0657	-	-	+CAC	apolipoprotein N-acyltransferase
SNP	696274	T→G	K63Q(AAA→CAA)	nagC	b0676	G	G	G	DNA-binding transcriptional dual regulator, repressor of N-acetylglucosamine
SNP	706447	A→G	intergenic (-116/+24)	fldA/ybfE	b0684/b0685	G	G	G	flavodoxin 1/LexA-regulated conserved protein
SNP	710995	A→C	pseudogene (469/651 nt)	ybfG	b0691	-	C	C	pseudogene
SNP	711869	A→G	G421G(GGT→GGC)	potE	b0692	G	G	G	putrescine/proton symporter: putrescine/ornithine antiporter
SNP	712265	T→G	L289L(CTA→CTC)	potE	b0692	G	G	G	putrescine/proton symporter: putrescine/ornithine antiporter
SNP	723250	A→C	S117A(TCT→GCT)	kdpA	b0698	C	C	C	potassium translocating ATPase, subunit A
SNP	730396	T→C	pseudogene (1311/1434 nt)	ybfO	b0703	C	C	C	pseudogene, rhs-like
SNP	730480	T→C	pseudogene (1395/1434 nt)	ybfO	b0703	C	C	C	pseudogene, rhs-like
SNP	730627	A→G	T38A(ACT→GCT)	ybfC	b0704	G	G	G	predicted protein
DEL	730631	Δ1bp	coding (116/570 nt)	ybfC	b0704	-	-	Δ1bp	predicted protein
SNP	731439	T→C	pseudogene (129/255 nt)	ybfQ	b4514	C	C	C	pseudogene, H repeat-associated protein
SNP	734894	A→C	K174N(AAA→AAC)	phr	b0708	-	C	C	deoxyribodipyrimidine photolyase, FAD-binding
SNP	735337	C→T	T322M(ACG→ATG)	phr	b0708	T	T	T	deoxyribodipyrimidine photolyase, FAD-binding
SNP	735632	A→C	L420L(CTA→CTC)	phr	b0708	C	C	C	deoxyribodipyrimidine photolyase, FAD-binding
SNP	738664	A→C	E30A(GAG→GCG)	ybgJ	b0711	C	C	C	predicted enzyme subunit
SNP	749576	A→C	intergenic (-242/-467)	gltA/sdhC	b0720/b0721	C	C	C	citrate synthase/succinate dehydrogenase, membrane subunit, binds cytochrome b556
SNP	749952	A→C	intergenic (-618/-91)	gltA/sdhC	b0720/b0721	-	C	C	citrate synthase/succinate dehydrogenase, membrane subunit, binds cytochrome b556
SNP	750344	A→C	E101A(GAA→GCA)	sdhC	b0721	-	C	C	succinate dehydrogenase, membrane subunit, binds cytochrome b556
SNP	752576	T→G	Y8D(TAT→GAT)	sdhB	b0724	G	G	G	succinate dehydrogenase, FeS subunit
SNP	755678	T→G	S703A(TCT→GCT)	sucA	b0726	-	G	G	2-oxoglutarate decarboxylase, thiamin-requiring
SNP	756350	A→C	N927H(AAT→CAT)	sucA	b0726	-	C	C	2-oxoglutarate decarboxylase, thiamin-requiring
SNP	761532	A→C	E228A(GAA→GCA)	mngA	b0731	-	-	C	fused 2-O-a-mannosyl-D-glycerate specific PTS enzymes: IIA component/IIB component/IIC component
SNP	762940	A→C	N33H(AAT→CAT)	mngB	b0732	-	-	C	alpha-mannosidase
SNP	763656	A→C	E271D(GAA→GAC)	mngB	b0732	-	C	C	alpha-mannosidase
SNP	766030	G→A	intergenic (+553/-294)	mngB/cydA	b0732/b0733	A	A	A	alpha-mannosidase/cytochrome d terminal oxidase, subunit I
SNP	772416	A→G	P350P(CCA→CCG)	tolA	b0739	-	G	G	membrane anchored protein in TolA-TolQ-TolR complex
SNP	775181	T→C	Y240H(TAC→CAC)	ybgF	b0742	C	C	C	periplasmic TolA-binding protein
SNP	778914	A→G	V259A(GTG→GCG)	zitB	b0752	G	G	G	zinc efflux system
SNP	784098	A→C	F250V(TTC→GTC)	galK	b0757	-	C	C	galactokinase
DEL	787967	Δ44bp	coding (645-688/1473 nt)	modF	b0760	Δ44bp	Δ44bp	Δ44bp	fused molybdate transporter subunits of ABC superfamily: ATP-binding components
SNP	789065	A→G	V149A(GTC→GCC)	modE	b0761	G	G	G	DNA-binding transcriptional repressor for the molybdenum transport operon modABC
SNP	793351	T→G	intergenic (-54/-101)	ybhA/pgl	b0766/b0767	-	-	G	pyridoxal phosphate (PLP) phosphatase/6-phosphogluconolactonase
SNP	797470	A→C	I240L(ATT→CTT)	ybhI	b0770	-	-	C	predicted transporter
SNP	800831	T→G	intergenic (+201/+33)	ybhJ/ybhC	b0771/b0772	-	-	G	predicted hydratase/acyl-CoA thioesterase, lipoprotein
SNP	802773	T→G	intergenic (-977/+101)	ybhC/cI	b0772/plasmid_gene01	G	-	-	acyl-CoA thioesterase, lipoprotein/phage lambda repressor protein CI
SNP	802776	C→G	intergenic (-980/+98)	ybhC/cI	b0772/plasmid_gene01	G	-	-	acyl-CoA thioesterase, lipoprotein/phage lambda repressor protein CI
SNP	802784	A→C	intergenic (-988/+90)	ybhC/cI	b0772/plasmid_gene01	C	-	-	acyl-CoA thioesterase, lipoprotein/phage lambda repressor protein CI
SNP	804361	A→C	L142F(TTA→TTC)	tetA	plasmid_gene02	-	C	C	TetA protein
SNP	809804	A→G	Q333Q(CAA→CAG)	bioF	b0776	-	G	G	8-amino-7-oxononanoate synthase
SNP	821664	C→T	L167L(CTG→CTA)	ybhO	b0789	T	T	T	cardiolipin synthase 2
SNP	829597	T→G	Y101D(TAC→GAC)	rhlE	b0797	-	-	G	ATP-dependent RNA helicase
SNP	839166	C→T	G264D(GGC→GAC)	fiu	b0805	T	T	T	catecholate siderophore receptor Fiu
SNP	845401	A→G	G48G(GGT→GGC)	glnP	b0810	G	G	G	glutamine transporter subunit
SNP	846666	T→G	intergenic (-237/+167)	glnH/dps	b0811/b0812	G	G	G	glutamine transporter subunit/Fe-binding and storage protein
SNP	847475	A→C	intergenic (-139/+160)	dps/rhtA	b0812/b0813	-	C	C	Fe-binding and storage protein/threonine and homoserine efflux system
SNP	847609	A→C	intergenic (-273/+26)	dps/rhtA	b0812/b0813	C	C	C	Fe-binding and storage protein/threonine and homoserine efflux system
DEL	848057	Δ1bp	coding (466/888 nt)	rhtA	b0813	-	Δ1bp	Δ1bp	threonine and homoserine efflux system
SNP	848086	T→C	D146G(GAT→GGT)	rhtA	b0813	-	C	C	threonine and homoserine efflux system
SNP	854868	A→C	K153Q(AAG→CAG)	ybiT	b0820	-	-	C	fused predicted transporter subunits of ABC superfamily: ATP-binding components
SNP	855372	T→G	F321V(TTT→GTT)	ybiT	b0820	-	G	G	fused predicted transporter subunits of ABC superfamily: ATP-binding components
SNP	857557	T→G	intergenic (-71/+81)	ybiU/ybiV	b0821/b0822	-	G	G	predicted protein/sugar phosphatase; preference for fructose-1-P, ribose-5-P and glucose-6-P
SNP	858301	A→C	L51L(CTT→CTG)	ybiV	b0822	-	-	C	sugar phosphatase; preference for fructose-1-P, ribose-5-P and glucose-6-P
SNP	865316	T→G	L108L(CTT→CTG)	iaaA	b0828	G	G	G	Isoaspartyl peptidase
DEL	865532	Δ1bp	coding (540/966 nt)	iaaA	b0828	-	Δ1bp	Δ1bp	Isoaspartyl peptidase
DEL	866840	Δ1bp	coding (1004/1980 nt)	gsiA	b0829	Δ1bp	Δ1bp	Δ1bp	glutathione transporter ATP-binding protein, ABC superfamily
SNP	878030	C→T	P288P(CCC→CCT)	yliI	b0837	T	T	T	soluble aldose sugar dehydrogenase
SNP	881060	A→C	S34A(TCG→GCG)	deoR	b0840	-	-	C	DNA-binding transcriptional repressor
SNP	889245	T→G	intergenic (-67/-93)	grxA/ybjC	b0849/b0850	G	G	G	glutaredoxin 1, redox coenzyme for ribonucleotide reductase (RNR1a)/conserved protein, DUF1418 family
INS	895070	+G	coding (512/954 nt)	potH	b0856	-	+G	+G	putrescine transporter subunit: membrane component of ABC superfamily
SNP	897170	T→G	H76Q(CAT→CAG)	rlmD	b0859	-	G	G	23S rRNA m(5)U747 methyltransferase
INS	899264	+C	intergenic (-264/+27)	artJ/artM	b0860/b0861	+C	+C	+C	arginine binding protein, periplasmic/arginine transporter subunit
SNP	902468	C→T	G142D(GGC→GAC)	ybjP	b0865	T	T	T	lipoprotein
SNP	905583	C→T	L299L(CTG→CTA)	ybjT	b0869	T	T	T	conserved protein with NAD(P)-binding Rossmann- fold domain
SNP	915006	A→C	S37R(AGC→CGC)	ybjD	b0876	-	C	C	conserved protein with nucleoside triphosphate hydrolase domain
SNP	915895	T→G	V333G(GTA→GGA)	ybjD	b0876	-	G	G	conserved protein with nucleoside triphosphate hydrolase domain
SNP	916006	T→G	F370C(TTT→TGT)	ybjD	b0876	-	-	G	conserved protein with nucleoside triphosphate hydrolase domain
SNP	920618	T→G	L616R(CTG→CGG)	macB	b0879	-	-	G	fused macrolide transporter subunits of ABC superfamily: ATP-binding component/membrane component
INS	924317	+GG	noncoding (80/88 nt)	serW	b0883	+GG	+GG	+G	tRNA-Ser
SNP	926113	T→G	E503A(GAA→GCA)	cydC	b0886	-	G	G	fused glutathione, cysteine exporter subunits of ABC superfamily: membrane component/ATP-binding component
SNP	932183	A→G	T142A(ACC→GCC)	ftsK	b0890	-	G	G	DNA translocase at septal ring sorting daughter chromsomes
SNP	934053	A→C	Q765P(CAG→CCG)	ftsK	b0890	C	C	C	DNA translocase at septal ring sorting daughter chromsomes
SNP	940138	T→G	V252G(GTG→GGG)	dmsA	b0894	G	-	-	dimethyl sulfoxide reductase, anaerobic, subunit A
SNP	942250	A→C	K138Q(AAG→CAG)	dmsB	b0895	-	-	C	dimethyl sulfoxide reductase, anaerobic, subunit B
SNP	949011	C→T	W165*(TGG→TGA)	pflA	b0902	T	T	T	pyruvate formate lyase activating enzyme 1
SNP	958645	T→C	intergenic (+125/-44)	aroA/ycaL	b0908/b0909	-	C	C	5-enolpyruvylshikimate-3-phosphate synthetase/predicted peptidase with chaperone function
SNP	963846	A→C	I368L(ATC→CTC)	ycaI	b0913	-	C	C	inner membrane protein, ComEC family of competence proteins
SNP	978757	A→C	E1336A(GAA→GCA)	mukB	b0924	C	C	C	chromosome condensin MukBEF, ATPase and DNA- binding subunit
SNP	982288	T→G	Y72D(TAC→GAC)	ycbL	b0927	G	G	G	predicted metal-binding enzyme
SNP	992195	A→G	L92P(CTA→CCA)	ssuB	b0933	G	G	G	alkanesulfonate transporter subunit
SNP	992821	C→T	G146D(GGC→GAC)	ssuC	b0934	T	T	T	putative alkanesulfonate transporter subunit
SNP	996575	A→G	T95A(ACC→GCC)	elfA	b0938	G	G	G	predicted fimbrial-like adhesin protein
SNP	997449	A→G	T179A(ACC→GCC)	elfD	b0939	G	G	G	predicted periplasmic pilin chaperone
SNP	998745	A→C	K369Q(AAA→CAA)	elfC	b0940	C	C	C	predicted outer membrane usher protein
DEL	999441	Δ1bp	coding (1801/2601 nt)	elfC	b0940	Δ1bp	Δ1bp	Δ1bp	predicted outer membrane usher protein
SNP	999811	T→G	L724R(CTG→CGG)	elfC	b0940	G	G	G	predicted outer membrane usher protein
INS	1000642	+G	coding (411/1071 nt)	elfG	b0941	+G	+G	+G	predicted fimbrial-like adhesin protein
SNP	1000933	T→C	G234G(GGT→GGC)	elfG	b0941	C	C	C	predicted fimbrial-like adhesin protein
SNP	1003023	T→G	W218G(TGG→GGG)	ycbF	b0944	-	-	G	predicted periplasmic pilini chaperone
SNP	1010215	T→G	A609A(GCT→GCG)	uup	b0949	-	G	G	fused predicted transporter subunits of ABC superfamily: ATP-binding components
SNP	1011629	C→T	P402S(CCG→TCG)	pqiA	b0950	T	T	T	paraquat-inducible membrane protein A
SNP	1013631	T→C	V104A(GTT→GCT)	ymbA	b0952	C	C	C	conserved protein
SNP	1016248	A→G	R101R(CGT→CGC)	ycbZ	b0955	G	G	G	predicted peptidase
INS	1017393	+A	intergenic (+31/+45)	matP/ompA	b0956/b0957	+A	-	-	Ter macrodomain organizer matS-binding protein/outer membrane protein A (3a;II*;G;d)
SNP	1021781	T→G	Q176H(CAA→CAC)	yccS	b0960	-	-	G	inner membrane protein
SNP	1023613	T→C	S240P(TCC→CCC)	helD	b0962	-	C	C	DNA helicase IV
SNP	1024537	G→A	A548T(GCT→ACT)	helD	b0962	A	A	A	DNA helicase IV
SNP	1027528	A→G	D289D(GAT→GAC)	rlmI	b0967	-	G	G	23S rRNA m(5)C1962 methyltransferase, SAM- dependent
SNP	1036461	A→G	E99E(GAA→GAG)	appC	b0978	-	G	G	cytochrome bd-II oxidase, subunit I
SNP	1036864	A→G	T234A(ACC→GCC)	appC	b0978	-	-	G	cytochrome bd-II oxidase, subunit I
SNP	1038711	T→G	S331A(TCC→GCC)	appB	b0979	G	G	G	cytochrome bd-II oxidase, subunit II
SNP	1038870	A→C	M1M(ATG→CTG)	yccB	b4592	C	C	C	hypothetical protein
SNP	1039750	T→G	S237A(TCA→GCA)	appA	b0980	-	G	G	phosphoanhydride phosphorylase
SNP	1041171	A→C	S489A(TCT→GCT)	etk	b0981	-	-	C	cryptic autophosphorylating protein tyrosine kinase Etk
SNP	1041981	T→C	T219A(ACC→GCC)	etk	b0981	C	C	C	cryptic autophosphorylating protein tyrosine kinase Etk
SNP	1042061	A→C	I192S(ATT→AGT)	etk	b0981	C	C	C	cryptic autophosphorylating protein tyrosine kinase Etk
DEL	1042157	Δ1bp	coding (479/2181 nt)	etk	b0981	-	Δ1bp	Δ1bp	cryptic autophosphorylating protein tyrosine kinase Etk
SNP	1045151	T→G	E407A(GAA→GCA)	gfcD	b0984	-	-	G	conserved protein
SNP	1049821	A→C	intergenic (-233/-65)	cspH/cspG	b0989/b0990	-	C	C	stress protein, member of the CspA-family/cold shock protein homolog, cold-inducible
INS	1051935	+G	coding (2639/2715 nt)	torS	b0993	+G	+G	+G	hybrid sensory histidine kinase in two-component regulatory system with TorR
SNP	1054229	T→C	E115E(GAA→GAG)	torS	b0993	-	C	C	hybrid sensory histidine kinase in two-component regulatory system with TorR
SNP	1056909	A→G	N134S(AAC→AGC)	torC	b0996	G	G	G	trimethylamine N-oxide (TMAO) reductase I, cytochrome c-type subunit
INS	1057855	+G	coding (175/2547 nt)	torA	b0997	-	+G	+GG	trimethylamine N-oxide (TMAO) reductase I, catalytic subunit
SNP	1059926	A→C	Q749P(CAG→CCG)	torA	b0997	-	C	C	trimethylamine N-oxide (TMAO) reductase I, catalytic subunit
SNP	1064053	T→G	L15*(TTA→TGA)	agp	b1002	-	-	G	glucose-1-phosphatase/inositol phosphatase
SNP	1065942	T→G	Q64H(CAA→CAC)	wrbA	b1004	-	-	G	NAD(P)H:quinone oxidoreductase
SNP	1071707	T→G	K244Q(AAA→CAA)	rutA	b1012	G	G	G	pyrimidine oxygenase, FMN-dependent
SNP	1075600	T→G	I570L(ATT→CTT)	putA	b1014	-	-	G	fused DNA-binding transcriptional regulator/proline dehydrogenase/pyrroline-5-carboxylate dehydrogenase
SNP	1083674	A→C	K86N(AAA→AAC)	phoH	b1020	-	-	C	conserved protein with nucleoside triphosphate hydrolase domain
SNP	1088079	T→C	S68S(TCA→TCG)	pgaB	b1023	C	C	C	biofilm adhesin polysaccharide PGA export lipoprotein with a polysaccharide deacetylase activity needed for export
INS	1095455	+G	intergenic (+201/+535)	ycdU/serX	b1029/b1032	+G	-	+G	predicted inner membrane protein/tRNA-Ser
SNP	1100363	T→C	T64A(ACA→GCA)	csgF	b1038	C	C	C	outer membrane protein required for curli nucleation by CsgB
SNP	1104183	A→C	intergenic (+33/-62)	ymdA/ymdB	b1044/b1045	C	C	C	predicted protein/O-acetyl-ADP-ribose deacetylase; RNase III inhibitor during cold shock
SNP	1104470	A→C	K76Q(AAA→CAA)	ymdB	b1045	-	C	C	O-acetyl-ADP-ribose deacetylase; RNase III inhibitor during cold shock
SNP	1106738	A→C	F210C(TTC→TGC)	opgC	b1047	C	C	C	membrane protein required for succinylation of osmoregulated periplasmic glucans (OPGs)
SNP	1109578	C→T	N97N(AAC→AAT)	opgH	b1049	T	T	T	membrane glycosyltransferase
SNP	1114260	A→G	Y250H(TAT→CAT)	lpxL	b1054	-	G	G	lauryl-acyl carrier protein (ACP)-dependent acyltransferase
SNP	1118250	T→G	E254D(GAA→GAC)	solA	b1059	G	G	G	N-methyltryptophan oxidase, FAD-binding
SNP	1118419	A→G	V198A(GTC→GCC)	solA	b1059	G	G	G	N-methyltryptophan oxidase, FAD-binding
SNP	1129160	A→C	V43V(GTT→GTG)	flgA	b1072	C	C	C	assembly protein for flagellar basal-body periplasmic P ring
SNP	1132308	A→C	N28H(AAT→CAT)	flgF	b1077	-	-	C	flagellar component of cell-proximal portion of basal-body rod
INS	1136345	+G	coding (550/942 nt)	flgJ	b1081	-	+G	+G	muramidase
SNP	1151565	T→G	F389C(TTC→TGC)	fabF	b1095	-	G	G	3-oxoacyl-[acyl-carrier-protein] synthase II
SNP	1158245	C→T	E578K(GAA→AAA)	fhuE	b1102	T	T	T	ferric-rhodotorulic acid outer membrane transporter
SNP	1163603	T→C	S12S(AGT→AGC)	ycfP	b1108	-	C	C	conserved protein
SNP	1164004	A→G	E146G(GAG→GGG)	ycfP	b1108	G	G	G	conserved protein
SNP	1182620	G→A	P94S(CCA→TCA)	potB	b1125	A	A	A	polyamine transporter subunit
DEL	1182942	Δ1bp	coding (1078/1137 nt)	potA	b1126	Δ1bp	Δ1bp	Δ1bp	polyamine transporter subunit
SNP	1186772	C→T	G477D(GGT→GAT)	phoQ	b1129	T	T	T	sensory histidine kinase in two-component regulatory system with PhoP
SNP	1199644	T→C	intergenic (-187/-278)	xisE/ymfI	b1141/b1143	C	C	C	e14 prophage; predicted excisionase/e14 prophage; predicted protein
SNP	1211342	T→G	K64T(AAA→ACA)	ycgX	b1161	-	-	G	predicted protein
SNP	1212689	T→G	*404Y(TAA→TAC)	ycgF	b1163	G	-	-	anti-repressor for YcgE, blue light-responsive; FAD-binding; has c-di-GMP phosphodiesterase-like EAL domain, but does not degrade c-di-GMP
SNP	1213602	T→C	H100R(CAC→CGC)	ycgF	b1163	-	C	C	anti-repressor for YcgE, blue light-responsive; FAD-binding; has c-di-GMP phosphodiesterase-like EAL domain, but does not degrade c-di-GMP
SNP	1216694	A→C	Q314H(CAA→CAC)	ycgG	b1168	C	C	C	predicted cyclic-di-GMP phosphodiesterase
SNP	1217237	T→G	I495M(ATT→ATG)	ycgG	b1168	-	-	G	predicted cyclic-di-GMP phosphodiesterase

SNP	1228892	A→C	intergenic (-73/-300)	hlyE/umuD	b1182/b1183	-	-	C	hemolysin E/DNA polymerase V, subunit D
SNP	1233622	C→T	A87V(GCG→GTG)	fadR	b1187	T	T	T	DNA-binding transcriptional dual regulator of fatty acid metabolism
SNP	1238020	T→G	I239M(ATT→ATG)	dadX	b1190	G	G	G	alanine racemase 2, PLP-binding
SNP	1238656	T→G	intergenic (+282/+104)	dadX/cvrA	b1190/b1191	G	G	G	alanine racemase 2, PLP-binding/predicted cation/proton antiporter
SNP	1241889	T→C	R59R(CGT→CGC)	emtA	b1193	C	C	C	lytic murein endotransglycosylase E
SNP	1245317	T→C	Y162C(TAC→TGC)	treA	b1197	C	C	C	periplasmic trehalase
DEL	1254257	Δ1bp	coding (121/2868 nt)	ycgV	b1202	-	Δ1bp	Δ1bp	predicted adhesin
SNP	1255771	T→G	K156T(AAA→ACA)	ychF	b1203	-	G	G	ATPase, K+-dependent, ribosome-associated
SNP	1257846	A→C	V452V(GTT→GTG)	ychM	b1206	-	C	C	predicted transporter
SNP	1261873	A→G	V18A(GTG→GCG)	lolB	b1209	G	G	G	OM lipoprotein required for localization of lipoproteins
SNP	1265109	C→T	G197G(GGC→GGT)	prmC	b1212	T	T	T	N5-glutamine methyltransferase, modifies release factors RF-1 and RF-2
SNP	1266904	T→G	V105V(GTT→GTG)	kdsA	b1215	-	G	G	3-deoxy-D-manno-octulosonate 8-phosphate synthase
SNP	1276147	A→G	intergenic (-104/-235)	narX/narK	b1222/b1223	G	G	G	sensory histidine kinase in two-component regulatory system with NarL/nitrate/nitrite transporter
SNP	1284621	A→G	P116P(CCA→CCG)	narI	b1227	-	G	G	nitrate reductase 1, gamma (cytochrome b(NR)) subunit
SNP	1287485	T→G	intergenic (-27/-185)	ychJ/rssA	b1233/b1234	G	G	G	conserved protein, UPF0225 family/predicted phospholipase, patatin-like family
SNP	1288515	A→G	G282G(GGA→GGG)	rssA	b1234	G	G	G	predicted phospholipase, patatin-like family
SNP	1294614	A→G	S645P(TCC→CCC)	adhE	b1241	G	G	G	fused acetaldehyde-CoA dehydrogenase/iron- dependent alcohol dehydrogenase/pyruvate-formate lyase deactivase
MOB	1297920		intergenic (+250/-485)	ychE/oppA	b1242/b1243	MOB	MOB	MOB	predicted inner membrane protein/oligopeptide transporter subunit
SNP	1302769	T→G	Y264D(TAT→GAT)	oppD	b1246	G	G	G	oligopeptide transporter subunit
SNP	1307660	T→G	intergenic (-285/+135)	kch/yciI	b1250/b1251	-	-	G	voltage-gated potassium channel/predicted enzyme
SNP	1308206	C→T	intergenic (-19/-109)	yciI/tonB	b1251/b1252	-	-	T	predicted enzyme/membrane spanning protein in TonB-ExbB-ExbD transport complex
SNP	1315256	T→G	K129T(AAA→ACA)	trpB	b1261	-	G	G	tryptophan synthase, beta subunit
SNP	1326479	A→C	intergenic (-141/-238)	yciK/sohB	b1271/b1272	C	C	C	predicted oxoacyl-(acyl carrier protein) reductase, EmrKY-TolC system/predicted inner membrane peptidase; multicopy suppressor of htrA(degP)
SNP	1326850	A→C	D45A(GAC→GCC)	sohB	b1272	-	C	C	predicted inner membrane peptidase; multicopy suppressor of htrA(degP)
SNP	1331956	A→G	A292A(GCA→GCG)	cysB	b1275	G	G	G	DNA-binding transcriptional dual regulator, O- acetyl-L-serine-binding
SNP	1332406	A→G	E7E(GAA→GAG)	ymiA	b4522	G	G	G	hypothetical protein
SNP	1332942	T→G	intergenic (+258/-115)	yciX/acnA	b4523/b1276	G	G	G	hypothetical protein/aconitate hydratase 1
SNP	1339708	A→G	T188A(ACG→GCG)	pyrF	b1281	-	G	G	orotidine-5'-phosphate decarboxylase
SNP	1346040	A→C	F33L(TTT→TTG)	rnb	b1286	-	C	C	ribonuclease II
SNP	1347669	A→G	G199G(GGT→GGC)	fabI	b1288	G	G	G	enoyl-[acyl-carrier-protein] reductase, NADH- dependent
SNP	1352480	C→T	V73I(GTC→ATC)	sapB	b1293	-	-	T	antimicrobial peptide transport ABC transporter permease
SNP	1352489	A→G	L70L(TTA→CTA)	sapB	b1293	G	G	G	antimicrobial peptide transport ABC transporter permease
SNP	1355399	T→G	T339P(ACT→CCT)	puuP	b1296	-	G	G	putrescine importer
SNP	1364239	A→G	Y300Y(TAT→TAC)	pspF	b1303	G	G	G	DNA-binding transcriptional activator
SNP	1366845	A→C	intergenic (+5/-70)	pspD/pspE	b1307/b1308	-	C	C	peripheral inner membrane phage-shock protein/thiosulfate:cyanide sulfurtransferase (rhodanese)
SNP	1370497	T→C	L17P(CTT→CCT)	ycjO	b1311	-	C	C	predicted sugar transporter subunit: membrane component of ABC superfamily
SNP	1374075	A→C	T2T(ACA→ACC)	ycjS	b1315	-	C	C	predicted oxidoreductase, NADH-binding
SNP	1374443	A→G	D125G(GAC→GGC)	ycjS	b1315	G	G	G	predicted oxidoreductase, NADH-binding
SNP	1375927	A→C	E273A(GAA→GCA)	ycjT	b1316	C	C	C	predicted hydrolase
SNP	1377799	A→G	Q142Q(CAA→CAG)	ycjU	b1317	G	G	G	beta-phosphoglucomutase
SNP	1377995	A→G	T208A(ACC→GCC)	ycjU	b1317	G	G	G	beta-phosphoglucomutase
SNP	1378982	T→G	pseudogene (936/1082 nt)	ycjV	b4524	G	G	G	pseudogene; putative ATP-binding component of a transport system
SNP	1395564	A→C	R95R(CGT→CGG)	uspE	b1333	-	C	C	stress-induced protein
DEL	1397205	Δ1bp	coding (258/516 nt)	ogt	b1335	Δ1bp	Δ1bp	Δ1bp	O-6-alkylguanine-DNA:cysteine-protein methyltransferase
DEL	1397947	Δ1bp	coding (1053/1527 nt)	abgT	b1336	Δ1bp	Δ1bp	Δ1bp	p-aminobenzoyl-glutamate transporter; membrane protein
SNP	1399320	C→T	D388N(GAT→AAT)	abgB	b1337	-	-	T	p-aminobenzoyl-glutamate hydrolase, B subunit
SNP	1402449	T→G	F161L(TTT→TTG)	abgR	b1339	-	G	G	predicted DNA-binding transcriptional regulator of abgABT operon
INS	1416940	+G	intergenic (-258/+51)	ydaG/racR	b1355/b1356	+G	+G	+G	Rac prophage; predicted protein/Rac prophage; predicted DNA-binding transcriptional regulator
SNP	1420156	A→G	pseudogene (230/612 nt)	ydaW	b1361	G	G	G	Rac prophage; pseudogene, DNA-binding protein homoolgy;Phage or Prophage Related
SNP	1420474	T→G	pseudogene (548/612 nt)	ydaW	b1361	-	G	G	Rac prophage; pseudogene, DNA-binding protein homoolgy;Phage or Prophage Related
SNP	1424384	A→C	pseudogene (705/1029 nt)	ynaA	b1368	C	C	C	Rac prophage; pseudogene, tail protein homology;Phage or Prophage Related; putative alpha helix protein
SNP	1426664	A→G	S130S(TCA→TCG)	stfR	b1372	G	G	G	Rac prophage; predicted tail fiber protein
SNP	1431355	T→C	G32G(GGA→GGG)	ynaE	b1375	-	C	C	cold shock protein, function unknown, Rac prophage
SNP	1434605	T→G	N1136H(AAT→CAT)	ydbK	b1378	-	G	G	fused predicted pyruvate-flavodoxin oxidoreductase: conserved protein/conserved protein/FeS binding protein
SNP	1435081	A→G	V977A(GTA→GCA)	ydbK	b1378	G	G	G	fused predicted pyruvate-flavodoxin oxidoreductase: conserved protein/conserved protein/FeS binding protein
SNP	1442776	A→C	N834H(AAT→CAT)	ydbH	b1381	-	-	C	predicted protein
SNP	1448971	T→G	K633T(AAG→ACG)	paaZ	b1387	-	G	G	fused oxepin-CoA hydrolase/3-oxo-5,6- dehydrosuberyl-CoA semialdehyde dehydrogenase
SNP	1451320	A→G	A34A(GCA→GCG)	paaA	b1388	G	G	G	ring 1,2-phenylacetyl-CoA epoxidase subunit
SNP	1456187	A→C	D233A(GAC→GCC)	paaG	b1394	-	-	C	1,2-epoxyphenylacetyl-CoA isomerase, oxepin-CoA- forming
SNP	1459526	A→G	D59G(GAC→GGC)	paaK	b1398	-	G	G	phenylacetyl-CoA ligase
SNP	1460162	T→C	V271A(GTT→GCT)	paaK	b1398	C	C	C	phenylacetyl-CoA ligase
SNP	1462172	A→G	Y159C(TAC→TGC)	paaY	b1400	G	G	G	predicted hexapeptide repeat acetyltransferase
SNP	1464342	T→G	pseudogene (1725/8622 nt)	ydbA	b4492	-	-	G	pseudogene, autotransporter homolog; interrupted by IS2 and IS30
SNP	1466392	A→G	pseudogene (3775/8622 nt)	ydbA	b4492	G	G	G	pseudogene, autotransporter homolog; interrupted by IS2 and IS30
SNP	1467912	T→C	pseudogene (5295/8622 nt)	ydbA	b4492	-	C	C	pseudogene, autotransporter homolog; interrupted by IS2 and IS30
DEL	1471536	Δ1bp	coding (90/861 nt)	ydbC	b1406	Δ1bp	Δ1bp	Δ1bp	predicted oxidoreductase, NAD(P)-binding
INS	1472082	+G	coding (636/861 nt)	ydbC	b1406	+G	+G	+G	predicted oxidoreductase, NAD(P)-binding
SNP	1473106	A→G	E246G(GAG→GGG)	ydbD	b1407	G	G	G	predicted PF10971 family periplasmic methylglyoxal resistance protein
SNP	1473865	A→G	Y499C(TAC→TGC)	ydbD	b1407	G	G	G	predicted PF10971 family periplasmic methylglyoxal resistance protein
SNP	1475255	A→C	N137H(AAC→CAC)	ynbA	b1408	C	C	C	inner membrane protein
SNP	1477986	A→G	K541K(AAA→AAG)	ynbC	b1410	G	G	G	predicted hydrolase
SNP	1479346	T→G	I436M(ATT→ATG)	ynbD	b1411	G	G	G	predicted phosphatase inner membrane protein
SNP	1482635	A→G	S783S(TCA→TCG)	hrpA	b1413	G	G	G	predicted ATP-dependent helicase
SNP	1484704	A→C	I82L(ATC→CTC)	ydcF	b1414	-	C	C	conserved SAM-binding protein, DUF218 superfamily
SNP	1484935	A→G	T159A(ACG→GCG)	ydcF	b1414	G	G	G	conserved SAM-binding protein, DUF218 superfamily
SNP	1487284	A→C	pseudogene (656/1001 nt)	gapC	b4493	C	C	C	pseudogene, GAP dehydrogenase; glyceraldehyde-3- phosphate dehydrogenase (second fragment)
SNP	1490054	C→T	T120I(ACA→ATA)	trg	b1421	T	T	T	methyl-accepting chemotaxis protein III, ribose and galactose sensor receptor
SNP	1503442	A→G	L15L(CTA→CTG)	ydcN	b1434	G	G	G	predicted DNA-binding transcriptional regulator
SNP	1504765	T→G	N153K(AAT→AAG)	ydcP	b1435	G	G	G	predicted peptidase
SNP	1507111	A→C	A126A(GCA→GCC)	hicB	b1438	C	C	C	antitoxin for the HicAB toxin-antitoxin system; mutational suppressor of null rpoE lethality
SNP	1507804	T→G	D192E(GAT→GAG)	ydcR	b1439	G	G	G	fused predicted DNA-binding transcriptional regulator/predicted amino transferase
SNP	1513831	A→G	H343R(CAC→CGC)	prr	b1444	G	G	G	gamma-aminobutyraldehyde dehydrogenase
SNP	1517724	A→G	K79K(AAA→AAG)	mcbR	b1450	G	G	G	colanic acid and biofilm gene transcriptional regulator, MqsR-controlled
SNP	1518425	T→G	M623L(ATG→CTG)	yncD	b1451	-	G	G	predicted iron outer membrane transporter
SNP	1519937	G→A	Q119*(CAA→TAA)	yncD	b1451	-	A	A	predicted iron outer membrane transporter
SNP	1521024	C→T	S164S(AGC→AGT)	yncE	b1452	-	T	T	ATP-binding protein, periplasmic, function unknown
SNP	1530822	A→G	W86R(TGG→CGG)	yddH	b1462	-	-	G	conserved protein
SNP	1531565	A→C	T106P(ACC→CCC)	nhoA	b1463	C	C	C	N-hydroxyarylamine O-acetyltransferase
SNP	1536829	A→C	F996L(TTT→TTG)	narZ	b1468	-	-	C	nitrate reductase 2 (NRZ), alpha subunit
SNP	1544040	T→C	N119S(AAC→AGC)	yddG	b1473	-	C	C	aromatic amino acid exporter
SNP	1546850	A→G	S742G(AGC→GGC)	fdnG	b1474	G	G	G	formate dehydrogenase-N, alpha subunit, nitrate- inducible
SNP	1548202	A→G	E172E(GAA→GAG)	fdnH	b1475	G	G	G	formate dehydrogenase-N, Fe-S (beta) subunit, nitrate-inducible
SNP	1549212	A→G	I217V(ATA→GTA)	fdnI	b1476	-	G	G	formate dehydrogenase-N, cytochrome B556 (gamma) subunit, nitrate-inducible
SNP	1553213	A→C	intergenic (-24/+78)	sra/bdm	b1480/b1481	-	C	C	Stationary-phase-induced ribosome-associated protein/biofilm-dependent modulation protein
SNP	1559419	A→G	F97L(TTC→CTC)	ddpA	b1487	G	G	G	D-ala-D-a la transporter subunit
SNP	1560916	T→G	K682Q(AAG→CAG)	dosP	b1489	-	G	G	oxygen sensor, c-di-GMP phosphodiesterase, heme- regulated; cold- and stationary phase-induced bioflim regulator
SNP	1574326	A→G	F174L(TTC→CTC)	yddB	b1495	G	G	G	predicted porin protein
SNP	1580149	T→G	intergenic (-399/+3)	ydeN/ydeO	b1498/b1499	G	G	G	conserved protein/transcriptional activator for mdtEF
SNP	1580974	T→G	intergenic (-61/+14)	ydeO/safA	b1499/b1500	G	G	G	transcriptional activator for mdtEF/Two component system connector membrane protein, EvgSA to PhoQP
SNP	1581045	T→G	E47D(GAA→GAC)	safA	b1500	-	-	G	Two component system connector membrane protein, EvgSA to PhoQP
SNP	1581952	C→T	V587V(GTG→GTA)	ydeP	b1501	T	T	T	predicted oxidoreductase
SNP	1582490	A→G	V408A(GTA→GCA)	ydeP	b1501	G	G	G	predicted oxidoreductase
SNP	1583800	A→G	intergenic (-88/+246)	ydeP/ydeQ	b1501/b1502	G	G	G	predicted oxidoreductase/predicted fimbrial-like adhesin protein
SNP	1584759	T→G	N68H(AAT→CAT)	ydeQ	b1502	-	-	G	predicted fimbrial-like adhesin protein
INS	1586806	+C	pseudogene (422/1149 nt)	ydeT	b1505	-	+C	+C	pseudogene; putative outer membrane protein
SNP	1587507	T→G	pseudogene (256/283 nt)	yneL	b1506	-	G	G	pseudogene, AraC family
SNP	1588852	A→G	I184T(ATT→ACT)	hipA	b1507	G	G	G	serine protein kinase required for perister formation; toxin of HipAB toxin-antitoxin system
INS	1591350	+C	pseudogene (3963/5422 nt)	yneO	b4696	+C	+C	-	pseudogene, AidA homolog
INS	1591474	+C	pseudogene (3839/5422 nt)	yneO	b4696	+C	+C	+C	pseudogene, AidA homolog
INS	1594532	+C	pseudogene (781/5422 nt)	yneO	b4696	+C	+CC	+CC	pseudogene, AidA homolog
DEL	1595561	Δ3bp	intergenic (-249/+280)	yneO/lsrK	b4696/b1511	Δ3bp	Δ3bp	Δ3bp	pseudogene, AidA homolog/autoinducer-2 (AI-2) kinase
SNP	1600520	T→G	C92W(TGT→TGG)	lsrC	b1514	-	-	G	Autoinducer 2 import system permease protein
SNP	1600898	G→A	A218A(GCG→GCA)	lsrC	b1514	A	A	A	Autoinducer 2 import system permease protein
SNP	1612034	A→T	D2V(GAT→GTT)	yneJ	b1526	-	T	T	predicted DNA-binding transcriptional regulator
SNP	1613516	T→G	H176Q(CAT→CAG)	yneK	b1527	G	G	G	predicted protein
SNP	1614602	A→C	I117L(ATT→CTT)	ydeA	b1528	-	-	C	predicted arabinose transporter
SNP	1615469	T→G	*222Y(TAA→TAC)	marC	b1529	-	-	G	inner membrane protein, UPF0056 family
SNP	1619087	T→G	F177C(TTC→TGC)	ydeE	b1534	-	G	G	predicted transporter
SNP	1619149	T→G	S198A(TCG→GCG)	ydeE	b1534	-	G	G	predicted transporter
SNP	1619477	T→C	V307A(GTA→GCA)	ydeE	b1534	-	C	C	predicted transporter
SNP	1623219	T→C	D463G(GAT→GGT)	dcp	b1538	-	C	C	dipeptidyl carboxypeptidase II
SNP	1624215	T→G	E131A(GAA→GCA)	dcp	b1538	G	G	G	dipeptidyl carboxypeptidase II
SNP	1624481	T→G	E42D(GAA→GAC)	dcp	b1538	-	-	G	dipeptidyl carboxypeptidase II
SNP	1628531	A→C	pseudogene (21534/21837 nt)	ydfJ	b4600	C	C	C	pseudogene, MFS transporter family; interrupted by Qin prophage;Phage or Prophage Related; putative transport protein
SNP	1633929	T→G	pseudogene (16136/21837 nt)	ydfJ	b4600	-	-	G	pseudogene, MFS transporter family; interrupted by Qin prophage;Phage or Prophage Related; putative transport protein
SNP	1634635	A→G	pseudogene (15430/21837 nt)	ydfJ	b4600	G	G	G	pseudogene, MFS transporter family; interrupted by Qin prophage;Phage or Prophage Related; putative transport protein
SNP	1639008	C→T	pseudogene (11057/21837 nt)	ydfJ	b4600	T	T	T	pseudogene, MFS transporter family; interrupted by Qin prophage;Phage or Prophage Related; putative transport protein
SNP	1642588	A→G	pseudogene (7477/21837 nt)	ydfJ	b4600	G	G	G	pseudogene, MFS transporter family; interrupted by Qin prophage;Phage or Prophage Related; putative transport protein
SNP	1645797	G→A	pseudogene (4268/21837 nt)	ydfJ	b4600	A	A	A	pseudogene, MFS transporter family; interrupted by Qin prophage;Phage or Prophage Related; putative transport protein
SNP	1646635	T→G	pseudogene (3430/21837 nt)	ydfJ	b4600	G	G	G	pseudogene, MFS transporter family; interrupted by Qin prophage;Phage or Prophage Related; putative transport protein
SNP	1647771	A→C	pseudogene (2294/21837 nt)	ydfJ	b4600	C	C	C	pseudogene, MFS transporter family; interrupted by Qin prophage;Phage or Prophage Related; putative transport protein
SNP	1650276	A→C	F289C(TTC→TGC)	rspB	b1580	-	C	C	predicted oxidoreductase, Zn-dependent and NAD(P)-binding
SNP	1650511	T→G	I211L(ATT→CTT)	rspB	b1580	-	G	G	predicted oxidoreductase, Zn-dependent and NAD(P)-binding
SNP	1655420	A→G	A42A(GCA→GCG)	ynfE	b1587	G	G	G	probable selenate reductase, periplasmic
DEL	1658799	Δ1bp	coding (1018/2424 nt)	ynfF	b1588	-	Δ1bp	Δ1bp	S- and N-oxide reductase, A subunit, periplasmic
SNP	1669911	T→C	S242S(AGT→AGC)	ydgD	b1598	C	C	C	predicted peptidase
SNP	1672337	G→A	A417V(GCG→GTG)	pntB	b1602	A	A	A	pyridine nucleotide transhydrogenase, beta subunit
SNP	1673202	T→G	I129L(ATC→CTC)	pntB	b1602	-	-	G	pyridine nucleotide transhydrogenase, beta subunit
SNP	1674031	T→C	T367A(ACC→GCC)	pntA	b1603	C	C	C	pyridine nucleotide transhydrogenase, alpha subunit
SNP	1677913	G→A	L377L(TTG→TTA)	ydgI	b1605	A	A	A	predicted arginine/ornithine antiporter transporter
SNP	1679187	T→G	K24Q(AAA→CAA)	ydgC	b1607	G	G	G	inner membrane protein, GlpM family
SNP	1686796	A→C	E332D(GAA→GAC)	manA	b1613	-	-	C	mannose-6-phosphate isomerase
SNP	1687651	C→T	S192F(TCC→TTC)	ydgA	b1614	T	T	T	conserved protein, DUF945 family
SNP	1690787	G→A	C234C(TGC→TGT)	uidB	b1616	A	A	A	glucuronide transporter
SNP	1697000	A→T	I141F(ATC→TTC)	malX	b1621	T	T	T	fused maltose and glucose-specific PTS enzymes: IIB component, IIC component
SNP	1697316	T→G	L246*(TTA→TGA)	malX	b1621	-	-	G	fused maltose and glucose-specific PTS enzymes: IIB component, IIC component
SNP	1700001	T→G	S182A(TCT→GCT)	add	b1623	-	G	G	adenosine deaminase
SNP	1701166	T→C	N123S(AAC→AGC)	ydgJ	b1624	-	-	C	predicted oxidoreductase
SNP	1709610	A→C	intergenic (+227/-513)	nth/dtpA	b1633/b1634	-	C	C	DNA glycosylase and apyrimidinic (AP) lyase (endonuclease III)/dipeptide and tripeptide permease A
INS	1724713	+G	coding (830/1098 nt)	nemA	b1650	+G	-	-	N-ethylmaleimide reductase, FMN-linked
INS	1729870	+A	coding (3559/4617 nt)	lhr	b1653	+A	-	-	predicted ATP-dependent helicase
SNP	1738104	T→C	R323R(CGT→CGC)	ydhC	b1660	C	C	C	predicted transporter
SNP	1740831	T→C	G23G(GGT→GGC)	mdtK	b1663	-	-	C	multidrug efflux system transporter
SNP	1744696	T→C	V114A(GTA→GCA)	ydhS	b1668	C	C	C	conserved protein with FAD/NAD(P)-binding domain
SNP	1745076	T→C	L241L(TTG→CTG)	ydhS	b1668	C	C	C	conserved protein with FAD/NAD(P)-binding domain
SNP	1745478	T→C	F375L(TTC→CTC)	ydhS	b1668	C	C	C	conserved protein with FAD/NAD(P)-binding domain
SNP	1745693	T→C	R446R(CGT→CGC)	ydhS	b1668	-	C	C	conserved protein with FAD/NAD(P)-binding domain
SNP	1748212	A→C	V9V(GTT→GTG)	ydhX	b1671	C	C	C	predicted 4Fe-4S ferridoxin-type protein
SNP	1751285	T→G	I140L(ATT→CTT)	ydhY	b1674	-	-	G	predicted 4Fe-4S ferridoxin-type protein
SNP	1751312	A→C	C131G(TGC→GGC)	ydhY	b1674	-	-	C	predicted 4Fe-4S ferridoxin-type protein
SNP	1753985	A→G	T355A(ACC→GCC)	pykF	b1676	G	G	G	pyruvate kinase I
SNP	1758490	T→G	D176A(GAT→GCT)	sufD	b1681	-	G	G	FeS cluster assembly protein of SufBCD complex
SNP	1760156	T→G	K360T(AAG→ACG)	sufB	b1683	-	G	G	component of SufBCD complex
SNP	1767253	T→G	L319V(TTG→GTG)	ydiK	b1688	-	G	G	inner membrane protein, UPF0118 family
SNP	1778213	T→C	R196R(CGT→CGC)	ydiR	b1698	-	C	C	predicted electron transfer flavoprotein, FAD- binding subunit
SNP	1782056	A→C	F761C(TTT→TGT)	ppsA	b1702	-	C	C	phosphoenolpyruvate synthase
SNP	1782401	G→A	P646L(CCG→CTG)	ppsA	b1702	A	A	A	phosphoenolpyruvate synthase
SNP	1786785	A→G	intergenic (+79/-53)	aroH/ydiE	b1704/b1705	-	-	G	3-deoxy-D-arabino-heptulosonate-7-phosphate synthase, tryptophan repressible/hemin uptake protein HemP homolog
SNP	1787701	A→C	Y257D(TAC→GAC)	ydiU	b1706	-	C	C	conserved protein, UPF0061 family
SNP	1789440	A→C	intergenic (-195/+52)	cdgR/nlpC	b1707/b1708	-	C	C	probable cyclic di-GMP regulator, inactive EAL family phosphodiesterase/predicted peptidase, C40 clan, lipoprotein
SNP	1790647	A→C	M46R(ATG→AGG)	btuD	b1709	-	C	C	vitamin B12 transporter subunit : ATP-binding component of ABC superfamily
SNP	1798012	A→G	V595A(GTC→GCC)	thrS	b1719	G	G	G	threonyl-tRNA synthetase
SNP	1804933	A→C	intergenic (+18/-88)	ydiZ/yniA	b1724/b1725	C	C	C	predicted protein/predicted phosphotransferase/kinase
SNP	1809020	A→C	S288R(AGT→CGT)	ydjN	b1729	C	C	C	predicted transporter
SNP	1817269	T→C	T391A(ACC→GCC)	chbC	b1737	C	C	C	N,N'-diacetylchitobiose-specific enzyme IIC component of PTS
SNP	1824156	T→G	G443G(GGA→GGC)	astB	b1745	-	G	G	succinylarginine dihydrolase
SNP	1824191	T→G	N432H(AAT→CAT)	astB	b1745	G	G	G	succinylarginine dihydrolase

SNP	1831135	A→G	L170L(CTA→CTG)	ydjX	b1750	G	G	G	inner membrane protein, TVP38/TMEM64 family
SNP	1842516	A→C	L557R(CTA→CGA)	topB	b1763	-	C	C	DNA topoisomerase III
SNP	1844040	G→A	A49V(GCG→GTG)	topB	b1763	A	A	A	DNA topoisomerase III
SNP	1846150	T→G	F30C(TTC→TGC)	sppA	b1766	-	G	G	protease IV (signal peptide peptidase)
SNP	1846297	A→C	K79T(AAA→ACA)	sppA	b1766	C	C	C	protease IV (signal peptide peptidase)
SNP	1849645	T→G	C178W(TGT→TGG)	pncA	b1768	-	-	G	nicotinamidase/pyrazinamidase
SNP	1852050	A→C	intergenic (-7/+166)	ydjF/ydjG	b1770/b1771	C	C	C	predicted DNA-binding transcriptional regulator/alpha-Keto reductase, NADH-dependent; can use methylglyoxal as substrate
DEL	1853140	Δ1bp	coding (57/981 nt)	ydjG	b1771	Δ1bp	Δ1bp	Δ1bp	alpha-Keto reductase, NADH-dependent; can use methylglyoxal as substrate
SNP	1858237	T→G	T107T(ACA→ACC)	ydjL	b1776	G	G	G	predicted oxidoreductase, Zn-dependent and NAD(P)-binding
SNP	1858732	A→C	intergenic (-175/+195)	ydjL/yeaC	b1776/b1777	-	-	C	predicted oxidoreductase, Zn-dependent and NAD(P)-binding/conserved protein
SNP	1861278	A→C	K68N(AAA→AAC)	yeaD	b1780	C	C	C	conserved protein
SNP	1866788	T→C	P203P(CCT→CCC)	yeaH	b1784	-	-	C	conserved protein
SNP	1866926	A→G	A249A(GCA→GCG)	yeaH	b1784	G	G	G	conserved protein
SNP	1869809	T→C	S182P(TCC→CCC)	yeaJ	b1786	C	C	C	predicted diguanylate cyclase
SNP	1870384	T→C	G373G(GGT→GGC)	yeaJ	b1786	-	C	C	predicted diguanylate cyclase
SNP	1876397	T→C	G28G(GGA→GGG)	yeaQ	b1795	C	C	C	conserved protein, UPF0410 family
DEL	1876580	Δ1bp	intergenic (-100/+48)	yeaQ/yoaG	b1795/b1796	Δ1bp	Δ1bp	Δ1bp	conserved protein, UPF0410 family/predicted protein
SNP	1878940	G→A	S32L(TCA→TTA)	dmlR	b1799	A	A	A	DNA-binding transcriptional activator for dmlA
SNP	1883494	A→C	Q142P(CAG→CCG)	yeaX	b1803	C	C	C	predicted oxidoreductase
SNP	1886231	A→C	H269Q(CAT→CAG)	fadD	b1805	-	C	C	acyl-CoA synthetase (long-chain-fatty-acid--CoA ligase)
SNP	1889517	A→C	L289R(CTG→CGG)	yoaA	b1808	C	C	C	conserved protein with nucleoside triphosphate hydrolase domain
SNP	1892428	T→G	D133E(GAT→GAG)	pabB	b1812	G	G	G	aminodeoxychorismate synthase, subunit I
SNP	1895005	T→G	T277T(ACT→ACG)	sdaA	b1814	-	G	G	L-serine deaminase I
SNP	1895029	T→C	G285G(GGT→GGC)	sdaA	b1814	C	-	-	L-serine deaminase I
SNP	1896937	T→G	L429R(CTT→CGT)	adrB	b1815	G	G	G	predicted phosphodiesterase
SNP	1900124	T→G	D262E(GAT→GAG)	manX	b1817	-	G	G	fused mannose-specific PTS enzymes: IIA component/IIB component
SNP	1903684	T→G	H201P(CAT→CCT)	rlmA	b1822	G	G	G	23S rRNA m(1)G745 methyltransferase
SNP	1910096	T→C	Y649C(TAC→TGC)	prc	b1830	C	C	C	carboxy-terminal protease for penicillin-binding protein 3
SNP	1911879	A→C	S55A(TCT→GCT)	prc	b1830	-	C	C	carboxy-terminal protease for penicillin-binding protein 3
SNP	1912744	T→G	K6Q(AAG→CAG)	proQ	b1831	-	G	G	RNA chaperone, probable regulator of ProP translation
SNP	1914666	T→G	F395C(TTC→TGC)	yebS	b1833	-	-	G	inner membrane protein
SNP	1921917	G→A	A93V(GCC→GTC)	yobA	b1841	A	A	A	conserved protein
SNP	1923192	T→G	N176K(AAT→AAG)	yobB	b1843	-	G	G	conserved protein
INS	1924460	+C	coding (1605/2061 nt)	ptrB	b1845	-	-	+C	protease II
SNP	1927525	T→C	T31A(ACT→GCT)	yebF	b1847	C	C	C	secreted protein
SNP	1930411	A→C	V473V(GTT→GTG)	edd	b1851	-	C	C	6-phosphogluconate dehydratase
INS	1933749	+A	intergenic (-210/-127)	zwf/yebK	b1852/b1853	+A	+A	+A	glucose-6-phosphate 1-dehydrogenase/predicted DNA-binding transcriptional regulator
SNP	1946038	A→C	F570C(TTT→TGT)	aspS	b1866	-	C	C	aspartyl-tRNA synthetase
SNP	1967415	T→C	D265G(GAT→GGT)	tap	b1885	C	C	C	methyl-accepting protein IV
SNP	1970546	A→C	N6K(AAT→AAG)	cheW	b1887	-	C	C	purine-binding chemotaxis protein
SNP	1974425	T→G	intergenic (-119/+65)	motA/flhC	b1890/b1891	-	-	G	proton conductor component of flagella motor/DNA-binding transcriptional dual regulator with FlhD
SNP	1974973	T→G	E32D(GAA→GAC)	flhC	b1891	-	G	G	DNA-binding transcriptional dual regulator with FlhD
SNP	1978940	A→C	T243T(ACT→ACG)	otsB	b1897	-	C	C	trehalose-6-phosphate phosphatase, biosynthetic
SNP	1983009	A→C	F115C(TTT→TGT)	araF	b1901	C	C	C	L-arabinose transporter subunit
SNP	1988377	A→C	V156G(GTA→GGA)	yecA	b1908	C	C	C	conserved protein, UPF0149 family
SNP	1989858	A→C	F62V(TTT→GTT)	pgsA	b1912	-	C	C	phosphatidylglycerophosphate synthetase
SNP	1995737	G→A	A323V(GCC→GTC)	dcyD	b1919	-	A	A	D-cysteine desulfhydrase, PLP-dependent
INS	1996572	+G	coding (133/987 nt)	dcyD	b1919	-	-	+G	D-cysteine desulfhydrase, PLP-dependent
SNP	2000983	T→G	intergenic (-153/-113)	fliC/fliD	b1923/b1924	G	G	G	flagellar filament structural protein (flagellin)/flagellar filament capping protein
SNP	2003433	T→C	G18G(GGT→GGC)	amyA	b1927	-	C	C	cytoplasmic alpha-amylase
SNP	2006342	A→C	E281A(GAA→GCA)	yedE	b1929	C	C	C	predicted inner membrane protein
SNP	2011359	T→G	S303A(TCC→GCC)	fliF	b1938	-	G	G	flagellar basal-body MS-ring and collar protein
SNP	2021267	A→C	K26Q(AAA→CAA)	rcsA	b1951	-	C	C	DNA-binding transcriptional activator, co- regulator with RcsB
SNP	2035425	A→C	I208S(ATT→AGT)	yedW	b1969	-	C	C	predicted DNA-binding response regulator in two- component system with YedV
SNP	2040809	A→C	intergenic (-28/-66)	serU/mtfA	b1975/b1976	C	C	C	tRNA-Ser/anti-repressor for DgsA(Mlc)
SNP	2040895	A→C	K7N(AAA→AAC)	mtfA	b1976	C	C	C	anti-repressor for DgsA(Mlc)
SNP	2045139	G→A	G993E(GGA→GAA)	yeeJ	b1978	A	A	A	probable adhesin
SNP	2050932	A→C	R22R(CGA→CGC)	shiA	b1981	-	C	C	shikimate transporter
INS	2056638	+A	coding (277/1488 nt)	yeeO	b1985	-	-	+A	predicted multdrug exporter, MATE family
SNP	2058768	A→G	G130G(GGT→GGC)	nac	b1988	G	G	G	DNA-binding transcriptional dual regulator of nitrogen assimilation
SNP	2059202	T→C	intergenic (-45/-282)	nac/asnV	b1988/b1989	-	C	C	DNA-binding transcriptional dual regulator of nitrogen assimilation/tRNA-Asn
SNP	2063222	T→G	intergenic (-234/+67)	cobU/yeeH	b1993/b4639	G	G	G	bifunctional cobinamide kinase/ cobinamide phosphate guanylyltransferase/pseudogene
SNP	2063647	A→C	F300C(TTC→TGC)	insH	b1994	-	-	C	IS5 transposase and trans-activator
SNP	2065634	T→C	pseudogene (10/15 nt)	yoeH	b4641	-	C	C	pseudogene, CP4-44 putative prophage remnant;Phage or Prophage Related
SNP	2065732	A→C	intergenic (-89/-127)	yoeH/yoeA	b4641/b4582	-	C	C	pseudogene, CP4-44 putative prophage remnant;Phage or Prophage Related/CP4-44 prophage; predicted disrupted hemin or colicin receptor;Phage or Prophage Related; interrupted by IS2 and C-terminal deletion
SNP	2065996	T→G	pseudogene (138/1870 nt)	yoeA	b4582	-	G	G	CP4-44 prophage; predicted disrupted hemin or colicin receptor;Phage or Prophage Related; interrupted by IS2 and C-terminal deletion
SNP	2068605	A→G	intergenic (+170/-158)	yeeP/flu	b1999/b2000	G	G	G	pseudogene, CP4-44 prophage; predicted GTP-binding protein;Phage or Prophage Related; putative histone/CP4-44 prophage; antigen 43 (Ag43) phase- variable biofilm formation autotransporter
SNP	2068623	A→C	intergenic (+188/-140)	yeeP/flu	b1999/b2000	C	C	C	pseudogene, CP4-44 prophage; predicted GTP-binding protein;Phage or Prophage Related; putative histone/CP4-44 prophage; antigen 43 (Ag43) phase- variable biofilm formation autotransporter
SNP	2070255	G→A	G498D(GGC→GAC)	flu	b2000	-	A	A	CP4-44 prophage; antigen 43 (Ag43) phase- variable biofilm formation autotransporter
SNP	2071386	T→C	L875P(CTG→CCG)	flu	b2000	C	C	C	CP4-44 prophage; antigen 43 (Ag43) phase- variable biofilm formation autotransporter
SNP	2074834	T→G	S14S(TCT→TCG)	cbtA	b2005	-	G	G	CP4-44 prophage; toxin of the YeeV-YeeU toxin- antitoxin system
SNP	2078125	C→T	S121N(AGT→AAT)	sbmC	b2009	T	T	T	DNA gyrase inhibitor
SNP	2078515	A→G	intergenic (-29/+90)	sbmC/dacD	b2009/b2010	G	G	G	DNA gyrase inhibitor/D-alanyl-D-alanine carboxypeptidase (penicillin- binding protein 6b)
SNP	2080459	T→C	G160G(GGT→GGC)	sbcB	b2011	-	C	C	exonuclease I
SNP	2080987	T→G	I336M(ATT→ATG)	sbcB	b2011	G	G	G	exonuclease I
SNP	2082583	A→C	V56G(GTT→GGT)	yeeE	b2013	C	C	C	inner membrane protein, UPF0394 family
SNP	2093702	C→T	T212I(ACC→ATC)	hisF	b2025	-	T	T	imidazole glycerol phosphate synthase, catalytic subunit with HisH
SNP	2098120	T→C	T125A(ACC→GCC)	gnd	b2029	C	C	C	6-phosphogluconate dehydrogenase, decarboxylating
SNP	2100961	A→C	F258C(TTC→TGC)	wbbK	b2032	-	-	C	lipopolysaccharide biosynthesis protein
SNP	2102589	A→C	C231W(TGT→TGG)	wbbI	b2034	-	-	C	d-Galf:alpha-d-Glc beta-1,6- galactofuranosyltransferase
SNP	2106136	A→C	S225A(TCT→GCT)	rfbX	b2037	C	C	C	predicted polisoprenol-linked O-antigen transporter
SNP	2108715	G→A	F162F(TTC→TTT)	rfbD	b2040	-	-	A	dTDP-4-dehydrorhamnose reductase subunit, NAD(P)- binding, of dTDP-L-rhamnose synthase
SNP	2110655	T→G	intergenic (-562/+3)	rfbB/galF	b2041/b2042	G	G	G	dTDP-glucose 4,6 dehydratase, NAD(P)-binding/putative regulatory subunit for GalU
SNP	2112335	T→C	I262M(ATA→ATG)	wcaM	b2043	C	C	C	colanic acid biosynthesis protein
SNP	2117860	G→A	P307S(CCG→TCG)	wcaJ	b2047	A	A	A	predicted UDP-glucose lipid carrier transferase
SNP	2121696	T→G	S17R(AGC→CGC)	cpsB	b2049	-	-	G	mannose-1-phosphate guanylyltransferase
SNP	2123270	T→C	E59E(GAA→GAG)	gmm	b2051	C	C	C	GDP-mannose mannosyl hydrolase
SNP	2125114	T→G	E142A(GAA→GCA)	gmd	b2053	G	G	G	GDP-D-mannose dehydratase, NAD(P)-binding
SNP	2127426	A→C	L226W(TTG→TGG)	wcaD	b2056	-	-	C	predicted colanic acid polymerase
SNP	2133306	A→C	L6*(TTA→TGA)	wzb	b2061	C	C	C	protein-tyrosine phosphatase
SNP	2134163	T→G	E102A(GAA→GCA)	wza	b2062	G	G	G	lipoprotein required for capsular polysaccharide translocation through the outer membrane
SNP	2141916	A→G	Y476C(TAC→TGC)	yegE	b2067	-	G	G	predicted diguanylate cyclase, GGDEF domain signaling protein
SNP	2147970	T→C	G80G(GGA→GGG)	yegI	b2070	-	-	C	conserved protein
SNP	2154510	A→G	Y675C(TAC→TGC)	mdtB	b2075	G	G	G	multidrug efflux system, subunit B
SNP	2155204	G→A	L906L(CTG→CTA)	mdtB	b2075	-	-	A	multidrug efflux system, subunit B
SNP	2163750	A→C	K287Q(AAA→CAA)	yegQ	b2081	-	C	C	predicted peptidase
SNP	2166117	T→G	V61G(GTG→GGG)	yegS	b2086	G	G	G	phosphatidylglycerol kinase, metal-dependent
SNP	2171819	A→C	intergenic (-29/+2)	gatB/gatA	b2093/b2094	C	C	C	galactitol-specific enzyme IIB component of PTS/galactitol-specific enzyme IIA component of PTS
SNP	2181925	A→C	C201G(TGT→GGT)	thiM	b2104	C	C	C	hydoxyethylthiazole kinase
SNP	2183517	A→C	E126A(GAG→GCG)	rcnA	b2106	-	C	C	membrane protein conferring nickel and cobalt resistance
SNP	2183989	T→C	intergenic (+24/-195)	rcnA/rcnB	b2106/b2107	C	C	C	membrane protein conferring nickel and cobalt resistance/periplasmic modulator of Ni and Co efflux
SNP	2186163	A→G	S658P(TCG→CCG)	yehB	b2109	G	G	G	predicted outer membrane protein
SNP	2197498	A→C	intergenic (+5/-5)	yehH/yehI	b4499/b2118	C	C	C	pseudogene; molybdate metabolism regulator, first fragment; molybdate metabolism regulator, second fragment 2/conserved protein
SNP	2201803	A→C	intergenic (+290/-17)	yehK/yehL	b4541/b2119	-	C	C	predicted protein/predicted transporter subunit: ATP-binding component of ABC superfamily
SNP	2204143	T→G	L409V(TTA→GTA)	yehM	b2120	-	-	G	predicted protein
SNP	2206833	A→C	pseudogene (510/2001 nt)	yehQ	b2122	C	C	C	pseudogene
DEL	2211558	Δ1bp	coding (311/1686 nt)	yehU	b2126	Δ1bp	Δ1bp	Δ1bp	predicted sensory kinase in two-component system with YehT, inner membrane protein
SNP	2211580	A→C	L97V(TTA→GTA)	yehU	b2126	C	C	C	predicted sensory kinase in two-component system with YehT, inner membrane protein
SNP	2215631	T→G	M1M(ATG→CTG)	yehY	b2130	-	-	G	predicted transporter subunit: membrane component of ABC superfamily
SNP	2218755	C→T	M153I(ATG→ATA)	bglX	b2132	T	T	T	beta-D-glucoside glucohydrolase, periplasmic
SNP	2223388	A→C	I122L(ATC→CTC)	yohD	b2136	-	C	C	inner membrane protein, DedA family
SNP	2228789	A→G	T183A(ACT→GCT)	yohK	b2142	G	G	G	inner membrane protein, LrgB family
SNP	2237615	A→C	intergenic (-43/+237)	mglB/galS	b2150/b2151	-	C	C	methyl-galactoside transporter subunit/DNA-binding transcriptional repressor
SNP	2250869	A→G	R167R(CGT→CGC)	nupX	b2161	G	G	G	nucleoside permease
SNP	2252401	T→G	R4R(AGA→CGA)	rihB	b2162	-	G	G	ribonucleoside hydrolase 2
SNP	2253694	A→C	F289C(TTC→TGC)	psuT	b2164	-	-	C	predicted nucleoside transporter
SNP	2255137	T→G	E152A(GAA→GCA)	psuG	b2165	-	-	G	pseudouridine 5'-phosphate glycosidase
SNP	2264795	A→C	T443P(ACC→CCC)	yeiQ	b2172	-	-	C	Predicted D-mannonate oxidoreductase, NAD- dependent
SNP	2266224	T→C	S49S(AGT→AGC)	lpxT	b2174	C	C	C	Lipid A 1-diphosphate synthase; undecaprenyl pyrophosphate:lipid A 1-phosphate phosphotransferase
SNP	2271870	T→G	I156M(ATT→ATG)	yejB	b2178	-	-	G	microcin C transporter YejABEF, permease subunit; ABC family
SNP	2276228	A→C	Y234D(TAT→GAT)	bcr	b2182	C	C	C	bicyclomycin/multidrug efflux system
SNP	2279134	T→G	F427V(TTC→GTC)	yejH	b2184	-	-	G	predicted ATP-dependent DNA or RNA helicase
SNP	2279561	A→C	E569A(GAA→GCA)	yejH	b2184	-	-	C	predicted ATP-dependent DNA or RNA helicase
SNP	2280501	T→G	Q224P(CAG→CCG)	yejK	b2186	G	G	G	nucleotide associated protein
SNP	2282031	T→G	V144V(GTT→GTG)	yejM	b2188	G	G	G	predicted hydrolase, inner membrane
SNP	2294540	A→C	L108R(CTG→CGG)	ccmA	b2201	C	C	C	heme exporter subunit
SNP	2297492	T→G	*829S(TAA→TCA)	napA	b2206	-	G	G	nitrate reductase, periplasmic, large subunit
SNP	2304686	T→G	K385T(AAA→ACA)	yojI	b2211	G	G	G	Microcin J25 efflux pump, TolC-dependent; fused ABC transporter permease and ATP-binding components
SNP	2315396	A→C	C553G(TGC→GGC)	rcsC	b2218	-	C	C	hybrid sensory kinase in two-component regulatory system with RcsB and YojN
SNP	2319290	A→C	L67L(CTA→CTC)	atoC	b2220	-	-	C	fused response regulator of ato operon, in two- component system with AtoS: response regulator/sigma54 interaction protein
SNP	2319465	A→C	K126Q(AAA→CAA)	atoC	b2220	-	-	C	fused response regulator of ato operon, in two- component system with AtoS: response regulator/sigma54 interaction protein
SNP	2322276	G→A	M99I(ATG→ATA)	atoE	b2223	A	A	A	short chain fatty acid transporter
SNP	2322394	C→T	L139F(CTC→TTC)	atoE	b2223	-	T	T	short chain fatty acid transporter
SNP	2322417	T→C	G146G(GGT→GGC)	atoE	b2223	C	C	C	short chain fatty acid transporter
SNP	2328571	A→C	pseudogene (3056/4605 nt)	yfaS	b4500	-	C	C	pseudogene, UPF0192 family; putative membrane protein
SNP	2342848	A→C	I254L(ATC→CTC)	nrdA	b2234	-	-	C	ribonucleoside-diphosphate reductase 1, alpha subunit
SNP	2348808	A→C	F264C(TTC→TGC)	glpT	b2240	-	C	C	sn-glycerol-3-phosphate transporter
SNP	2350542	T→G	I224M(ATT→ATG)	glpA	b2241	-	-	G	sn-glycerol-3-phosphate dehydrogenase (anaerobic), large subunit, FAD/NAD(P)-binding
SNP	2360299	A→C	F187V(TTT→GTT)	yfaY	b2249	-	-	C	conserved protein
SNP	2361247	A→C	Y85D(TAC→GAC)	yfaZ	b2250	-	-	C	outer membrane protein, possible porin
SNP	2362786	G→A	A20V(GCG→GTG)	ais	b2252	-	A	A	predicted LPS core heptose(II)-phosphate phosphatase
SNP	2368114	A→C	Q291H(CAA→CAC)	arnD	b2256	-	-	C	Undecaprenyl phosphate-alpha-L-ara4FN deformylase
SNP	2369837	T→G	C19W(TGT→TGG)	arnE	b4544	-	G	G	undecaprenyl phosphate-alpha-L-ara4N exporter; flippase ArnEF subunit
SNP	2372147	T→C	Q27Q(CAA→CAG)	menE	b2260	C	C	C	o-succinylbenzoate-CoA ligase
SNP	2374806	T→G	H4P(CAC→CCC)	menH	b2263	-	-	G	2-succinyl-6-hydroxy-2, 4-cyclohexadiene-1- carboxylate synthase
SNP	2375339	T→G	E382A(GAA→GCA)	menD	b2264	-	-	G	bifunctional 2-oxoglutarate decarboxylase/ SHCHC synthase
SNP	2383839	T→G	V252V(GTT→GTG)	yfbL	b2271	G	G	G	predicted peptidase
SNP	2384079	A→C	intergenic (+24/-79)	yfbL/yfbM	b2271/b2272	C	C	C	predicted peptidase/conserved protein, DUF1877 family
SNP	2389786	T→G	K160N(AAA→AAC)	nuoM	b2277	G	G	G	NADH:ubiquinone oxidoreductase, membrane subunit M
SNP	2400609	A→C	I154S(ATC→AGC)	nuoC	b2286	-	-	C	NADH:ubiquinone oxidoreductase, fused CD subunit
SNP	2409033	T→G	H100P(CAT→CCT)	yfbT	b2293	G	G	G	sugar phosphatas
SNP	2412033	T→G	L16L(CTT→CTG)	pta	b2297	-	-	G	phosphate acetyltransferase
SNP	2423181	T→C	intergenic (-41/+49)	hisQ/hisJ	b2308/b2309	C	C	C	histidine/lysine/arginine/ornithine transporter permease subunit/histidine/lysine/arginine/ornithine transporter subunit
SNP	2424153	T→C	intergenic (-141/+80)	hisJ/argT	b2309/b2310	C	C	C	histidine/lysine/arginine/ornithine transporter subunit/lysine/arginine/ornithine transporter subunit
SNP	2429233	A→C	Y312D(TAT→GAT)	folC	b2315	C	C	C	bifunctional folylpolyglutamate synthase/ dihydrofolate synthase
SNP	2434871	T→C	T61A(ACC→GCC)	pdxB	b2320	-	C	C	erythronate-4-phosphate dehydrogenase
SNP	2437630	T→G	V400V(GTA→GTC)	fabB	b2323	G	G	G	3-oxoacyl-[acyl-carrier-protein] synthase I
SNP	2442785	T→G	*275S(TAA→TCA)	mepA	b2328	-	G	G	murein DD-endopeptidase
SNP	2444710	A→C	intergenic (-13/+22)	aroC/prmB	b2329/b2330	C	C	C	chorismate synthase/N5-glutamine methyltransferase
SNP	2448794	A→C	V5V(GTT→GTG)	yfcR	b2335	C	C	C	predicted fimbrial-like adhesin protein
SNP	2451203	A→C	pseudogene (1023/2646 nt)	yfcU	b4661	C	C	C	pseudogene, export usher homology;putative membrane; Not classified; putative outer membrane protein
INS	2452974	+A	intergenic (-104/+577)	yfcV/sixA	b2339/b2340	+A	+A	+A	predicted fimbrial-like adhesin protein/phosphohistidine phosphatase
SNP	2459083	G→A	G185D(GGC→GAC)	fadL	b2344	A	A	A	long-chain fatty acid outer membrane transporter
DEL	2460105	Δ1bp	intergenic (+235/-131)	fadL/yfdF	b2344/b2345	-	-	Δ1bp	long-chain fatty acid outer membrane transporter/predicted protein
SNP	2460608	A→C	N125H(AAT→CAT)	yfdF	b2345	-	-	C	predicted protein
SNP	2462752	A→G	A76A(GCA→GCG)	yfdC	b2347	G	G	G	predicted inner membrane protein
SNP	2465018	A→C	intergenic (+92/-61)	intS/gtrA	b2349/b2350	C	C	C	CPS-53 (KpLE1) prophage; predicted prophage CPS- 53 integrase/CPS-53 (KpLE1) prophage; bactoprenol-linked glucose translocase (flippase)
SNP	2466208	T→C	A257A(GCT→GCC)	gtrB	b2351	C	C	C	CPS-53 (KpLE1) prophage; bactoprenol glucosyl transferase
SNP	2490475	A→G	S2P(TCA→CCA)	frc	b2374	G	G	G	formyl-CoA transferase, NAD(P)-binding
SNP	2490548	A→G	intergenic (-70/+443)	frc/yfdX	b2374/b2375	G	G	G	formyl-CoA transferase, NAD(P)-binding/predicted protein
SNP	2498831	T→G	L160V(TTG→GTG)	ypdC	b2382	G	G	G	predicted DNA-binding protein
SNP	2500424	A→G	V429A(GTG→GCG)	fryA	b2383	G	G	G	fused predicted PTS enzymes: Hpr component/enzyme I component/enzyme IIA component
SNP	2501676	T→G	N12H(AAC→CAC)	fryA	b2383	-	-	G	fused predicted PTS enzymes: Hpr component/enzyme I component/enzyme IIA component
DEL	2505355	Δ1bp	coding (112/327 nt)	fryB	b2387	-	Δ1bp	Δ1bp	predicted enzyme IIB component of PTS
SNP	2507216	T→G	H121Q(CAT→CAG)	yfeO	b2389	G	G	G	predicted ion channel protein
SNP	2508229	T→G	F2C(TTC→TGC)	ypeC	b2390	-	-	G	conserved protein
SNP	2516647	A→C	L417R(CTG→CGG)	gltX	b2400	-	-	C	glutamyl-tRNA synthetase

SNP	2519483	T→C	E73E(GAA→GAG)	xapR	b2405	C	C	C	DNA-binding transcriptional activator for for xapAB
SNP	2521481	T→G	I208L(ATT→CTT)	xapA	b2407	-	-	G	purine nucleoside phosphorylase II
SNP	2521516	A→C	F196C(TTC→TGC)	xapA	b2407	C	C	C	purine nucleoside phosphorylase II
SNP	2522160	G→A	intergenic (-58/-191)	xapA/yfeN	b2407/b2408	-	A	A	purine nucleoside phosphorylase II/predicted outer membrane protein
SNP	2523368	C→T	G238D(GGC→GAC)	yfeR	b2409	-	-	T	predicted DNA-binding transcriptional regulator
SNP	2524006	C→T	E25E(GAG→GAA)	yfeR	b2409	T	T	T	predicted DNA-binding transcriptional regulator
SNP	2527108	A→G	V98A(GTG→GCG)	ligA	b2411	-	G	G	DNA ligase, NAD(+)-dependent
SNP	2529348	T→C	M221T(ATG→ACG)	cysZ	b2413	-	C	C	predicted inner membrane protein
SNP	2531986	A→C	N233H(AAC→CAC)	ptsI	b2416	-	-	C	PEP-protein phosphotransferase of PTS system (enzyme I)
SNP	2533148	A→C	I31L(ATC→CTC)	crr	b2417	-	-	C	glucose-specific enzyme IIA component of PTS
SNP	2537000	T→G	N347H(AAC→CAC)	cysA	b2422	G	G	G	sulfate/thiosulfate transporter subunit
SNP	2537771	T→C	T90A(ACG→GCG)	cysA	b2422	-	-	C	sulfate/thiosulfate transporter subunit
SNP	2549656	A→C	S27S(TCA→TCC)	amiA	b2435	-	C	C	N-acetylmuramoyl-l-alanine amidase I
SNP	2553892	T→G	E210D(GAA→GAC)	eutC	b2440	G	G	G	ethanolamine ammonia-lyase, small subunit (light chain)
SNP	2559630	A→C	D99A(GAT→GCT)	yffO	b2446	-	C	C	CPZ-55 prophage; predicted protein
DEL	2560940	Δ1bp	coding (125/378 nt)	yffQ	b2448	-	Δ1bp	Δ1bp	CPZ-55 prophage; predicted protein
SNP	2561075	T→G	V87G(GTG→GGG)	yffQ	b2448	G	G	G	CPZ-55 prophage; predicted protein
SNP	2565735	T→G	D334A(GAC→GCC)	eutG	b2453	G	G	G	ethanol dehydrogenase involved in ethanolamine utilization; aldehyde reductase, converts acetaldehyde to ethanol
SNP	2570596	A→C	V45G(GTC→GGC)	eutD	b2458	-	C	C	phosphate acetyltransferase
SNP	2573023	T→G	K3Q(AAA→CAA)	eutS	b2462	G	G	G	predicted carboxysome structural protein with predicted role in ethanol utilization
SNP	2573620	T→C	E661G(GAA→GGA)	maeB	b2463	C	C	C	fused malic enzyme predicted oxidoreductase/predicted phosphotransacetylase
SNP	2573821	A→C	F594C(TTT→TGT)	maeB	b2463	-	C	C	fused malic enzyme predicted oxidoreductase/predicted phosphotransacetylase
SNP	2574776	T→G	K276Q(AAA→CAA)	maeB	b2463	-	G	G	fused malic enzyme predicted oxidoreductase/predicted phosphotransacetylase
SNP	2575534	A→C	V23G(GTT→GGT)	maeB	b2463	-	-	C	fused malic enzyme predicted oxidoreductase/predicted phosphotransacetylase
SNP	2575953	T→C	S22P(TCC→CCC)	talA	b2464	-	C	C	transaldolase A
SNP	2580907	T→C	T615A(ACC→GCC)	aegA	b2468	C	C	C	fused predicted oxidoreductase: FeS binding subunit/NAD/FAD-binding subunit
SNP	2582838	T→C	intergenic (-89/-267)	aegA/narQ	b2468/b2469	C	C	C	fused predicted oxidoreductase: FeS binding subunit/NAD/FAD-binding subunit/sensory histidine kinase in two-component regulatory system with NarP (NarL)
SNP	2587943	A→C	intergenic (+11/+88)	acrD/ypfM	b2470/b4606	-	C	C	aminoglycoside/multidrug efflux system/hypothetical protein
SNP	2588367	T→G	intergenic (-277/-104)	ypfM/yffB	b4606/b2471	G	G	G	hypothetical protein/predicted reductase, function unknown, ArsC family; low abundance protein
SNP	2596158	T→C	D276G(GAC→GGC)	dapA	b2478	C	C	C	dihydrodipicolinate synthase
SNP	2599350	G→A	M103I(ATG→ATA)	hyfB	b2482	A	A	A	hydrogenase 4, membrane subunit
SNP	2603281	T→G	M416R(ATG→AGG)	hyfD	b2484	-	G	G	hydrogenase 4, membrane subunit
SNP	2603666	T→C	S61P(TCC→CCC)	hyfE	b2485	C	C	C	hydrogenase 4, membrane subunit
SNP	2604470	T→G	V110V(GTT→GTG)	hyfF	b2486	G	G	G	hydrogenase 4, membrane subunit
SNP	2607424	A→G	T13A(ACC→GCC)	hyfH	b2488	G	G	G	hydrogenase 4, Fe-S subunit
SNP	2609518	A→C	Q132P(CAA→CCA)	hyfR	b2491	-	C	C	DNA-binding transcriptional activator, formate sensing
SNP	2609524	G→A	W134*(TGG→TAG)	hyfR	b2491	-	-	A	DNA-binding transcriptional activator, formate sensing
SNP	2609938	T→G	L272R(CTT→CGT)	hyfR	b2491	-	-	G	DNA-binding transcriptional activator, formate sensing
SNP	2616409	T→G	I326L(ATC→CTC)	uraA	b2497	-	G	G	uracil permease
SNP	2616594	T→G	H264P(CAC→CCC)	uraA	b2497	-	G	G	uracil permease
SNP	2618851	A→C	E106A(GAA→GCA)	purM	b2499	-	-	C	phosphoribosylaminoimidazole synthetase
DEL	2620170	Δ1bp	intergenic (+74/-98)	purN/ppk	b2500/b2501	-	Δ1bp	Δ1bp	phosphoribosylglycinamide formyltransferase 1/polyphosphate kinase, component of RNA degradosome
DEL	2636277	Δ1bp	coding (231/621 nt)	yfgM	b2513	Δ1bp	Δ1bp	Δ1bp	conserved protein, UPF0070 family
SNP	2648602	C→T	D304N(GAT→AAT)	yfhM	b2520	T	T	T	conserved protein
SNP	2649382	T→G	N44H(AAC→CAC)	yfhM	b2520	G	G	G	conserved protein
SNP	2649899	A→G	H61R(CAC→CGC)	sseA	b2521	G	G	G	3-mercaptopyruvate sulfurtransferase
SNP	2650280	G→A	R188H(CGC→CAC)	sseA	b2521	-	A	A	3-mercaptopyruvate sulfurtransferase
DEL	2651952	Δ1bp	coding (206/777 nt)	sseB	b2522	Δ1bp	Δ1bp	Δ1bp	rhodanase-like enzyme, sulfur transfer from thiosulfate
SNP	2659486	T→C	intergenic (-131/+321)	iscR/trmJ	b2531/b2532	C	C	C	DNA-binding transcriptional repressor/tRNA mC32/mU32 methyltransferase, SAM-dependent
SNP	2660550	T→C	intergenic (-3/-116)	trmJ/suhB	b2532/b2533	C	C	C	tRNA mC32/mU32 methyltransferase, SAM-dependent/inositol monophosphatase
DEL	2664800	Δ1bp	coding (271/1140 nt)	hcaT	b2536	-	-	Δ1bp	predicted 3-phenylpropionic transporter
SNP	2673775	A→G	V92A(GTG→GCG)	yphD	b2546	G	G	G	predicted sugar transporter subunit: membrane component of ABC superfamily
SNP	2681724	A→C	A336A(GCT→GCG)	glyA	b2551	-	C	C	serine hydroxymethyltransferase
SNP	2682917	T→C	intergenic (-186/-142)	glyA/hmp	b2551/b2552	C	C	C	serine hydroxymethyltransferase/fused nitric oxide dioxygenase/dihydropteridine reductase 2
SNP	2686660	A→C	L24R(CTG→CGG)	yfhG	b2555	-	C	C	conserved protein
SNP	2687087	T→C	E397E(GAA→GAG)	glrK	b2556	-	C	C	Sensor protein kinase regulating glmY sRNA in two-component system with response regulator GlrR
SNP	2688707	T→G	intergenic (-430/+173)	glrK/purL	b2556/b2557	-	-	G	Sensor protein kinase regulating glmY sRNA in two-component system with response regulator GlrR/phosphoribosylformyl-glycineamide synthetase
SNP	2692434	A→C	Y112D(TAC→GAC)	purL	b2557	-	C	C	phosphoribosylformyl-glycineamide synthetase
SNP	2697213	T→G	intergenic (+66/+128)	yfhL/shoB	b2562/b4687	-	G	G	predicted 4Fe-4S cluster-containing protein/toxic membrane protein
SNP	2703390	T→C	N320S(AAC→AGC)	lepA	b2569	C	C	C	back-translocating Elongation Factor EF4, GTPase
SNP	2705362	A→C	L206W(TTG→TGG)	rseB	b2571	-	-	C	anti-sigma E factor, binds RseA
SNP	2705731	A→G	V83A(GTG→GCG)	rseB	b2571	-	G	G	anti-sigma E factor, binds RseA
SNP	2708898	T→C	L419L(TTA→CTA)	nadB	b2574	C	C	C	quinolinate synthase, L-aspartate oxidase (B protein) subunit
SNP	2712108	T→C	Y146C(TAT→TGT)	yfiE	b2577	C	C	C	predicted DNA-binding transcriptional regulator
SNP	2713705	G→A	intergenic (-32/-273)	yfiD/ung	b2579/b2580	A	A	A	autonomous glycyl radical cofactor/uracil-DNA-glycosylase
DEL	2715041	Δ1bp	coding (712/1038 nt)	yfiF	b2581	-	-	Δ1bp	predicted methyltransferase
SNP	2715822	T→G	intergenic (-70/-137)	yfiF/trxC	b2581/b2582	-	-	G	predicted methyltransferase/thioredoxin 2
SNP	2718105	C→T	S310L(TCA→TTA)	yfiQ	b2584	T	T	T	inhibiting acetyltransferase for acetyl-CoA synthetase
SNP	2718198	A→T	D341V(GAT→GTT)	yfiQ	b2584	T	T	T	inhibiting acetyltransferase for acetyl-CoA synthetase
SNP	2734184	T→G	intergenic (+77/-194)	bamD/raiA	b2595/b2597	G	G	G	lipoprotein required for OM biogenesis, in BamABCDE complex/cold shock protein associated with 30S ribosomal subunit
SNP	2734310	A→C	intergenic (+203/-68)	bamD/raiA	b2595/b2597	C	C	C	lipoprotein required for OM biogenesis, in BamABCDE complex/cold shock protein associated with 30S ribosomal subunit
MOB	2734342		intergenic (+235/-29)	bamD/raiA	b2595/b2597	MOB	MOB	MOB	lipoprotein required for OM biogenesis, in BamABCDE complex/cold shock protein associated with 30S ribosomal subunit
SNP	2736582	G→A	H238Y(CAT→TAT)	tyrA	b2600	A	A	A	fused chorismate mutase T/prephenate dehydrogenase
DEL	2738603	Δ1bp	coding (20/366 nt)	yfiL	b2602	-	-	Δ1bp	lipoprotein
SNP	2739631	A→C	N9H(AAT→CAT)	yfiN	b2604	C	C	C	probable inner membrane diguanylate cyclase
SNP	2743172	T→G	K80Q(AAA→CAA)	rpsP	b2609	-	G	G	30S ribosomal subunit protein S16
SNP	2744449	T→C	T191A(ACC→GCC)	ffh	b2610	C	C	C	Signal Recognition Particle (SRP) component with 4.5S RNA (ffs)
SNP	2747156	A→T	I372F(ATC→TTC)	yfjD	b4461	T	T	T	inner membrane protein, UPF0053 family
SNP	2753219	T→C	intergenic (+617/-164)	smpB/intA	b2620/b2622	-	-	C	trans-translation protein/CP4-57 prophage; integrase
SNP	2753472	G→A	G30G(GGG→GGA)	intA	b2622	A	A	A	CP4-57 prophage; integrase
SNP	2758791	T→G	E658D(GAA→GAC)	yfjK	b2627	-	-	G	CP4-57 prophage; conserved protein
SNP	2760012	T→C	G251G(GGA→GGG)	yfjK	b2627	C	C	C	CP4-57 prophage; conserved protein
INS	2763011	+C	intergenic (-11/-131)	yfjM/rnlA	b2629/b2630	+C	-	-	CP4-57 prophage; predicted protein/CP4-57 prophage; RNase LS
SNP	2764735	A→C	intergenic (+156/-199)	rnlB/yfjP	b2631/b2632	C	-	-	CP4-57 prophage; predicted protein/CP4-57 prophage; predicted GTP-binding protein
SNP	2767825	T→C	F105L(TTC→CTC)	ypjK	b2635	C	C	C	CP4-57 prophage; predicted inner membrane protein
SNP	2772154	A→C	K538T(AAG→ACG)	yfjW	b2642	-	C	C	CP4-57 prophage; predicted inner membrane protein
SNP	2774538	A→C	E67A(GAA→GCA)	yfjZ	b2645	C	C	C	CP4-57 prophage; antitoxin of the YpjF-YfjZ toxin-antitoxin system
SNP	2775602	T→C	E1450G(GAG→GGG)	ypjA	b2647	C	C	C	adhesin-like autotransporter
SNP	2776150	C→T	G1267G(GGG→GGA)	ypjA	b2647	T	T	T	adhesin-like autotransporter
SNP	2781140	T→C	pseudogene (1096/1374 nt)	ypjC	b2650	-	C	C	pseudogene
SNP	2782113	A→G	pseudogene (123/1374 nt)	ypjC	b2650	G	G	G	pseudogene
SNP	2786050	A→G	intergenic (+177/-159)	ygaQ/csiD	b4462/b2659	-	G	G	predicted protein; putative enzyme/carbon starvation induced gene
SNP	2789500	A→C	K335T(AAA→ACA)	gabD	b2661	C	C	C	succinate-semialdehyde dehydrogenase I, NADP- dependent
INS	2791387	+A	intergenic (+148/-90)	gabT/gabP	b2662/b2663	-	+A	+A	4-aminobutyrate aminotransferase, PLP-dependent/gamma-aminobutyrate transporter
SNP	2792580	C→T	G368G(GGC→GGT)	gabP	b2663	T	T	T	gamma-aminobutyrate transporter
SNP	2795648	G→A	D24D(GAC→GAT)	stpA	b2669	-	-	A	DNA binding protein, nucleoid-associated
DEL	2798335	Δ3bp	coding (147-149/411 nt)	nrdI	b2674	-	-	Δ3bp	flavodoxin required for NrdEF cluster assembly
INS	2800564	+T	coding (1954/2106 nt)	nrdE	b2675	+T	+T	+T	ribonucleoside-diphosphate reductase 2, alpha subunit
SNP	2800871	C→T	T49I(ACT→ATT)	nrdF	b2676	T	T	T	ribonucleoside-diphosphate reductase 2, beta subunit, ferritin-like protein
SNP	2800874	T→C	V50A(GTC→GCC)	nrdF	b2676	C	C	C	ribonucleoside-diphosphate reductase 2, beta subunit, ferritin-like protein
SNP	2803831	G→A	V200I(GTC→ATC)	proW	b2678	A	A	A	glycine betaine transporter subunit
SNP	2805636	T→G	pseudogene (97/1178 nt)	ygaY	b2681	-	G	G	predicted transporter (pseudogene);putative transport; Not classified; putative transport protein
SNP	2805944	A→G	pseudogene (405/1178 nt)	ygaY	b2681	G	G	G	predicted transporter (pseudogene);putative transport; Not classified; putative transport protein
SNP	2808822	G→A	A58T(GCA→ACA)	emrA	b2685	-	A	A	multidrug efflux system
SNP	2809301	T→C	R217R(CGT→CGC)	emrA	b2685	-	C	C	multidrug efflux system
SNP	2809938	T→G	A33A(GCT→GCG)	emrB	b2686	-	G	G	multidrug efflux system protein
SNP	2820868	A→G	S42S(TCT→TCC)	recA	b2699	G	G	G	DNA strand exchange and recombination protein with protease and nuclease activity
SNP	2821688	A→C	intergenic (-118/+27)	ygaD/mltB	b2700/b2701	-	C	C	conserved protein/membrane-bound lytic murein transglycosylase B
SNP	2821807	A→G	F332L(TTC→CTC)	mltB	b2701	G	G	G	membrane-bound lytic murein transglycosylase B
SNP	2822501	T→G	P100P(CCA→CCC)	mltB	b2701	-	G	G	membrane-bound lytic murein transglycosylase B
SNP	2823204	A→C	Q50P(CAG→CCG)	srlA	b2702	-	C	C	glucitol/sorbitol-specific enzyme IIC component of PTS
SNP	2824279	T→G	S222A(TCC→GCC)	srlE	b2703	-	-	G	glucitol/sorbitol-specific enzyme IIB component of PTS
INS	2828916	+T	coding (598/1515 nt)	norR	b2709	+T	+T	+T	Anaerobic nitric oxide reductase DNA-binding transcriptional activator
SNP	2836966	A→C	Q73H(CAA→CAC)	ascF	b2715	C	C	C	fused cellobiose/arbutin/salicin-specific PTS enzymes: IIB component/IC component
SNP	2837111	A→C	I122L(ATC→CTC)	ascF	b2715	-	C	C	fused cellobiose/arbutin/salicin-specific PTS enzymes: IIB component/IC component
SNP	2841829	A→C	C50G(TGC→GGC)	hycF	b2720	-	-	C	formate hydrogenlyase complex iron-sulfur protein
SNP	2842433	G→A	S421S(AGC→AGT)	hycE	b2721	-	A	A	hydrogenase 3, large subunit
SNP	2846505	T→G	D157A(GAT→GCT)	hycB	b2724	-	G	G	hydrogenase 3, Fe-S subunit
SNP	2847230	G→A	Q144*(CAA→TAA)	hycA	b2725	A	A	A	regulator of the transcriptional regulator FhlA
INS	2852408	+C	coding (847/2079 nt)	fhlA	b2731	-	-	+C	DNA-binding transcriptional activator
SNP	2853510	T→C	V650A(GTG→GCG)	fhlA	b2731	C	C	C	DNA-binding transcriptional activator
SNP	2854737	A→G	D100G(GAC→GGC)	pphB	b2734	-	G	G	serine/threonine-specific protein phosphatase 2
SNP	2856626	T→G	L173R(CTA→CGA)	ygbJ	b2736	-	-	G	predicted dehydrogenase, with NAD(P)-binding Rossmann-fold domain
SNP	2862279	C→T	intergenic (-49/+14)	rpoS/nlpD	b2741/b2742	-	T	T	RNA polymerase, sigma S (sigma 38) factor/activator of AmiC murein hydrolase activity, lipoprotein
SNP	2865559	T→G	D142A(GAT→GCT)	truD	b2745	-	-	G	tRNA(Glu) U13 pseudouridine synthase
SNP	2876037	A→G	V262A(GTC→GCC)	casC	b2758	-	-	G	CRISP RNA (crRNA) containing Cascade antiviral complex protein
SNP	2878110	T→G	P236P(CCA→CCC)	casA	b2760	G	G	G	CRISP RNA (crRNA) containing Cascade antiviral complex protein
SNP	2878205	T→G	T205P(ACG→CCG)	casA	b2760	G	G	G	CRISP RNA (crRNA) containing Cascade antiviral complex protein
SNP	2882326	T→C	E222E(GAA→GAG)	cysH	b2762	C	C	C	3'-phosphoadenosine 5'-phosphosulfate reductase
SNP	2887868	G→A	G178E(GGG→GAG)	ygcN	b2766	A	A	A	predicted oxidoreductase with FAD/NAD(P)-binding domain
SNP	2894918	T→C	D12G(GAT→GGT)	ygcW	b2774	C	C	C	predicted dehydrogenase
DEL	2894977	Δ1bp	intergenic (-25/-294)	ygcW/yqcE	b2774/b2775	-	Δ1bp	Δ1bp	predicted dehydrogenase/inner membrane protein, predicted transporter
SNP	2910985	T→C	V417A(GTG→GCG)	barA	b2786	-	-	C	hybrid sensory histidine kinase, in two- component regulatory system with UvrY
SNP	2911971	G→A	A746T(GCG→ACG)	barA	b2786	-	-	A	hybrid sensory histidine kinase, in two- component regulatory system with UvrY
SNP	2914802	G→A	G208G(GGC→GGT)	gudX	b2788	-	-	A	glucarate dehydratase-related protein, substrate unknown
SNP	2923359	T→G	M151R(ATG→AGG)	sdaC	b2796	-	G	G	predicted serine transporter
SNP	2931157	T→C	F299L(TTC→CTC)	fucI	b2802	C	C	C	L-fucose isomerase
DEL	2937573	Δ1bp	intergenic (-327/+24)	gcvA/ygdI	b2808/b2809	-	-	Δ1bp	DNA-binding transcriptional dual regulator/novel lipoprotein
SNP	2941514	T→G	N115T(AAC→ACC)	mltA	b2813	G	G	G	membrane-bound lytic murein transglycosylase A
SNP	2942788	G→A	A301V(GCC→GTC)	amiC	b2817	A	A	A	N-acetylmuramoyl-L-alanine amidase
SNP	2944028	C→T	A6A(GCC→GCT)	argA	b2818	T	T	T	fused acetylglutamate kinase homolog (inactive)/amino acid N-acetyltransferase
SNP	2953994	T→C	L1038L(TTA→TTG)	recC	b2822	C	C	C	exonuclease V (RecBCD complex), gamma chain
SNP	2954677	T→G	S811R(AGC→CGC)	recC	b2822	-	G	G	exonuclease V (RecBCD complex), gamma chain
SNP	2963050	A→G	L22L(TTA→CTA)	ptsP	b2829	-	G	G	fused PTS enzyme: PEP-protein phosphotransferase (enzyme I)/GAF domain containing protein
SNP	2963649	T→C	intergenic (-59/-692)	rppH/mutH	b2830/b2831	C	-	-	RNA pyrophosphohydrolase/methyl-directed mismatch repair protein
DEL	2965077	Δ1bp	intergenic (+47/-22)	mutH/ygdQ	b2831/b2832	Δ1bp	Δ1bp	Δ1bp	methyl-directed mismatch repair protein/inner membrane protein, UPF0053 family
SNP	2965418	T→G	I107S(ATC→AGC)	ygdQ	b2832	-	G	G	inner membrane protein, UPF0053 family
SNP	2965650	G→A	S184S(TCG→TCA)	ygdQ	b2832	A	A	A	inner membrane protein, UPF0053 family
SNP	2972527	T→G	Q351P(CAG→CCG)	lysA	b2838	G	G	G	diaminopimelate decarboxylase, PLP-binding
SNP	2974480	T→C	F261L(TTC→CTC)	lysR	b2839	-	-	C	DNA-binding transcriptional dual regulator
SNP	2981331	T→G	V269G(GTC→GGC)	yqeG	b2845	G	G	G	predicted transporter
SNP	2982151	T→C	intergenic (+396/-64)	yqeG/yqeH	b2845/b2846	C	C	C	predicted transporter/conserved protein with bipartite regulator domain
SNP	2989016	T→C	pseudogene (399/503 nt)	pbl	b2854	-	C	C	predicted peptidoglycan-binding enzyme (pseudogene)
INS	2994352	+G	coding (177/714 nt)	ygeR	b2865	+G	+G	+G	novel lipoprotein, function unknown
SNP	2997387	G→A	G32D(GGC→GAC)	xdhB	b2867	-	-	A	xanthine dehydrogenase, FAD-binding subunit
SNP	3001296	T→G	I119S(ATT→AGT)	ygeW	b2870	G	G	G	predicted carbamoyltransferase
SNP	3007270	T→C	intergenic (+198/+23)	yqeA/yqeB	b2874/b2875	-	C	C	predicted amino acid kinase/conserved protein with NAD(P)-binding Rossman fold
SNP	3008737	G→A	S61L(TCG→TTG)	yqeB	b2875	A	A	A	conserved protein with NAD(P)-binding Rossman fold
INS	3009627	+A	intergenic (-59/-212)	yqeC/mocA	b2876/b2877	+A	+A	+A	conserved protein/CTP:molybdopterin cytidylyltransferase
SNP	3013072	A→C	V778V(GTA→GTC)	ygfK	b2878	-	C	C	predicted oxidoreductase, Fe-S subunit
SNP	3021025	A→C	E194A(GAA→GCA)	guaD	b2883	C	C	C	guanine deaminase
SNP	3033548	T→G	Q258P(CAA→CCA)	xerD	b2894	G	G	G	site-specific tyrosine recombinase
SNP	3045032	A→C	T105T(ACT→ACG)	gcvT	b2905	-	C	C	aminomethyltransferase, tetrahydrofolate- dependent, subunit (T protein) of glycine cleavage complex
SNP	3047537	A→C	S221A(TCG→GCG)	ubiH	b2907	-	-	C	2-octaprenyl-6-methoxyphenol hydroxylase, FAD/NAD(P)-binding
SNP	3051147	G→A	L76L(TTG→TTA)	fau	b2912	A	A	A	conserved protein, 5-formyltetrahydrofolate cyclo-ligase family
SNP	3055707	T→C	V60A(GTT→GCT)	scpA	b2917	C	C	C	methylmalonyl-CoA mutase
SNP	3060438	T→G	Y320D(TAC→GAC)	scpC	b2920	-	-	G	propionyl-CoA:succinate-CoA transferase
SNP	3063883	T→C	K202E(AAG→GAG)	mscS	b2924	C	C	C	mechanosensitive channel protein, small conductance
SNP	3072628	T→C	Y304C(TAC→TGC)	cmtA	b2933	C	C	C	predicted fused mannitol-specific PTS enzymes: IIB component/IIC component
SNP	3076330	G→A	intergenic (-16/-262)	tktA/yggG	b2935/b2936	-	-	A	transketolase 1, thiamin-binding/heat shock protein binding to Era protein; predicted peptidase
SNP	3076992	A→G	H134R(CAC→CGC)	yggG	b2936	-	G	G	heat shock protein binding to Era protein; predicted peptidase
SNP	3077396	A→C	intergenic (+46/+160)	yggG/speB	b2936/b2937	-	-	C	heat shock protein binding to Era protein; predicted peptidase/agmatinase
SNP	3081070	C→T	L69F(CTT→TTT)	yqgC	b2940	-	T	T	predicted protein
SNP	3084077	T→C	V372A(GTA→GCA)	galP	b2943	C	C	C	D-galactose transporter
SNP	3090393	A→G	D206G(GAC→GGC)	yggS	b2951	-	G	G	predicted enzyme, binds pyridoxal 5'-phosphate
SNP	3092673	A→C	K243T(AAA→ACA)	yggW	b2955	-	C	C	predicted oxidoreductase, HemN family
SNP	3104125	A→G	intergenic (-291/-206)	speC/yqgA	b2965/b2966	G	G	G	ornithine decarboxylase, constitutive/predicted inner membrane protein, DUF554 family

SNP	3105393	T→C	D138G(GAC→GGC)	yghD	b2968	C	C	C	predicted secretion pathway M-type protein, membrane anchored
DEL	3109363	Δ1bp	coding (4429/4563 nt)	yghJ	b4466	-	Δ1bp	Δ1bp	predicted inner membrane lipoprotein
SNP	3113101	T→G	K231Q(AAA→CAA)	yghJ	b4466	G	G	G	predicted inner membrane lipoprotein
SNP	3119148	A→C	L331V(TTA→GTA)	glcF	b4467	-	-	C	glycolate oxidase 4Fe-4S iron-sulfur cluster subunit
SNP	3120599	T→C	L201L(TTA→TTG)	glcE	b4468	-	C	C	glycolate oxidase FAD binding subunit
SNP	3121566	A→C	L379V(TTA→GTA)	glcD	b2979	C	C	C	glycolate oxidase subunit, FAD-linked
SNP	3122651	T→C	D17G(GAC→GGC)	glcD	b2979	C	C	C	glycolate oxidase subunit, FAD-linked
SNP	3122822	T→C	intergenic (-122/-129)	glcD/glcC	b2979/b2980	C	C	C	glycolate oxidase subunit, FAD-linked/DNA-binding transcriptional dual regulator, glycolate-binding
SNP	3125969	T→G	intergenic (+96/+51)	insH/yghQ	b2982/b2983	G	G	G	IS5 transposase and trans-activator/predicted inner membrane protein
SNP	3130428	A→C	F208C(TTC→TGC)	pitB	b2987	C	C	C	phosphate transporter
SNP	3136218	T→C	H484R(CAC→CGC)	hybC	b2994	C	C	C	hydrogenase 2, large subunit
SNP	3137339	C→T	Q110Q(CAG→CAA)	hybC	b2994	T	T	T	hydrogenase 2, large subunit
SNP	3139053	C→T	G217D(GGC→GAC)	hybA	b2996	-	-	T	hydrogenase 2 4Fe-4S ferredoxin-type component
SNP	3144163	T→C	intergenic (-13/-178)	yqhA/yghA	b3002/b3003	C	C	C	predicted inner membrane protein, UPF0114 family/predicted oxidoreductase
INS	3151580	+A	coding (279/828 nt)	dkgA	b3012	-	+A	+A	2,5-diketo-D-gluconate reductase A
SNP	3153953	T→G	S625R(AGC→CGC)	ygiQ	b4469	G	G	G	conserved protein
SNP	3155817	A→G	S3S(TCT→TCC)	ygiQ	b4469	G	G	G	conserved protein
SNP	3159372	T→G	K427N(AAA→AAC)	parC	b3019	G	G	G	DNA topoisomerase IV, subunit A
DEL	3172183	Δ2bp	pseudogene (407-408/631 nt)	nudF	b3034	Δ2bp	Δ2bp	Δ2bp	ADP-ribose pyrophosphatase
SNP	3174549	A→G	E42E(GAA→GAG)	ygiB	b3037	G	G	G	conserved protein, UPF0441 family
SNP	3175601	A→G	E167E(GAA→GAG)	ygiC	b3038	G	G	G	Glutathionylspermidine synthase homolog
SNP	3182999	T→G	pseudogene (2257/3803 nt)	yqiG	b3046	-	G	G	pseudogene; outer membrane usher homology;putative membrane; Not classified; putative membrane protein
SNP	3192112	T→G	H737P(CAT→CCT)	glnE	b3053	-	G	G	fused deadenylyltransferase/adenylyltransferase for glutamine synthetase
SNP	3195449	A→C	M66R(ATG→AGG)	ygiF	b3054	C	C	C	predicted adenylate cyclase
SNP	3198487	A→G	F109L(TTC→CTC)	bacA	b3057	G	G	G	undecaprenyl pyrophosphate phosphatase
SNP	3219386	A→G	I692V(ATC→GTC)	ebgA	b3076	G	G	G	cryptic beta-D-galactosidase, alpha subunit
SNP	3222766	T→G	W96G(TGG→GGG)	ygjJ	b3079	G	G	G	conserved protein
SNP	3229936	A→G	V207A(GTC→GCC)	rlmG	b3084	G	G	G	23S rRNA mG1835 methyltransferase, SAM- dependent
SNP	3230227	A→G	V110A(GTG→GCG)	rlmG	b3084	G	G	G	23S rRNA mG1835 methyltransferase, SAM- dependent
SNP	3231161	A→C	intergenic (+18/-59)	ygjP/ygjQ	b3085/b3086	-	-	C	predicted metal dependent hydrolase/conserved protein, SanA family, DUF218 superfamily
SNP	3234284	T→G	intergenic (+59/-340)	alx/sstT	b3088/b3089	G	G	G	inner membrane protein, part of terminus/sodium:serine/threonine symporter
INS	3235657	+G	coding (1034/1245 nt)	sstT	b3089	-	+G	+G	sodium:serine/threonine symporter
SNP	3238968	T→G	K152Q(AAA→CAA)	uxaC	b3092	-	-	G	uronate isomerase
SNP	3239692	T→G	intergenic (-271/-92)	uxaC/exuT	b3092/b3093	-	G	G	uronate isomerase/hexuronate transporter
SNP	3240480	C→T	H233Y(CAT→TAT)	exuT	b3093	T	T	T	hexuronate transporter
SNP	3242437	T→G	intergenic (+329/-16)	exuR/yqjA	b3094/b3095	G	G	G	DNA-binding transcriptional repressor/required, with yghB, for membrane integrity; inner membrane protein
SNP	3248678	G→A	R73*(CGA→TGA)	yhaJ	b3105	A	A	A	predicted DNA-binding transcriptional regulator
SNP	3250447	A→C	D295E(GAT→GAG)	yhaM	b4470	C	C	C	conserved protein
SNP	3252618	T→C	S25G(AGC→GGC)	yhaO	b3110	C	C	C	predicted transporter
SNP	3254370	A→G	intergenic (-41/+31)	tdcG/tdcF	b4471/b3113	G	G	G	L-serine dehydratase 3/predicted L-PSP (mRNA) endoribonuclease
SNP	3260526	G→A	G61G(GGC→GGT)	tdcB	b3117	A	A	A	catabolic threonine dehydratase, PLP-dependent
SNP	3261351	T→G	E132A(GAA→GCA)	tdcA	b3118	-	G	G	DNA-binding transcriptional activator
SNP	3263513	A→G	N140S(AAC→AGC)	yhaC	b3121	-	-	G	predicted protein
SNP	3273798	G→A	G63S(GGC→AGC)	kbaZ	b3132	A	A	A	tagatose 6-phosphate aldolase 1, kbaZ subunit
SNP	3275469	A→C	pseudogene (89/402 nt)	agaW	b3134	-	-	C	N-acetylgalactosamine-specific enzyme IIC component of PTS, fragment (pseudogene);enzyme; Central intermediary metabolism: Amino sugars; PTS system N- acetylgalactosameine-specific IIC component 2
INS	3279410	+T	coding (46/804 nt)	agaC	b3139	-	-	+T	N-acetylgalactosamine-specific enzyme IIC component of PTS
SNP	3284488	A→G	N332S(AAC→AGC)	yraJ	b3144	G	G	G	predicted outer membrane protein
SNP	3288506	A→C	I143L(ATC→CTC)	lpoA	b3147	C	C	C	conserved protein
SNP	3288808	G→A	T243T(ACG→ACA)	lpoA	b3147	A	A	A	conserved protein
SNP	3290049	A→C	D657A(GAT→GCT)	lpoA	b3147	-	C	C	conserved protein
SNP	3293041	A→C	L162L(CTT→CTG)	yraR	b3152	C	C	C	predicted nucleoside-diphosphate-sugar epimerase
SNP	3296484	A→C	Y107S(TAT→TCT)	yhbU	b3158	-	-	C	predicted peptidase (collagenase-like)
SNP	3296797	C→T	R211R(CGC→CGT)	yhbU	b3158	-	T	T	predicted peptidase (collagenase-like)
INS	3299486	+C	coding (1012/1245 nt)	mtr	b3161	+C	-	-	tryptophan transporter of high affinity
SNP	3306409	A→C	intergenic (-45/+104)	rpsO/truB	b3165/b3166	C	C	C	30S ribosomal subunit protein S15/tRNA U55 pseudouridine synthase
SNP	3311826	A→C	V127V(GTT→GTG)	nusA	b3169	-	C	C	transcription termination/antitermination L factor
SNP	3313112	A→C	intergenic (-143/-205)	metY/argG	b3171/b3172	-	-	C	tRNA-Met/argininosuccinate synthetase
SNP	3327245	C→T	K57K(AAG→AAA)	yhbE	b3184	T	T	T	predicted inner membrane permease
INS	3327354	+C	coding (62/966 nt)	yhbE	b3184	-	+C	+C	predicted inner membrane permease
SNP	3332238	A→G	Y112H(TAT→CAT)	mlaC	b3192	G	G	G	ABC transporter maintaining OM lipid asymmetry, periplasmic binding protein
SNP	3343044	T→G	intergenic (+126/-88)	npr/yrbL	b3206/b3207	G	G	G	phosphohistidinoprotein-hexose phosphotransferase component of N-regulated PTS system (Npr)/predicted protein
SNP	3343336	A→G	T69A(ACC→GCC)	yrbL	b3207	G	G	G	predicted protein
SNP	3345345	T→G	intergenic (-206/+24)	elbB/arcB	b3209/b3210	-	-	G	isoprenoid biosynthesis protein with amidotransferase-like domain/aerobic respiration control sensor histidine protein kinase, cognate to two-component response regulators ArcA and RssB
SNP	3347961	A→C	I257S(ATC→AGC)	yhcC	b3211	-	-	C	predicted Fe-S oxidoreductase
SNP	3356764	T→G	intergenic (+144/-28)	gltF/yhcA	b3214/b3215	-	G	G	periplasmic protein/predicted periplasmic chaperone protein
SNP	3363468	T→C	V321A(GTT→GCT)	yhcG	b3220	C	C	C	conserved protein
SNP	3366075	A→G	Y394H(TAT→CAT)	nanT	b3224	G	G	G	sialic acid transporter
SNP	3368703	T→G	E156A(GAA→GCA)	nanR	b3226	-	-	G	DNA-binding transcriptional repressor of the nan operon, induced by sialic acid
SNP	3370843	T→C	V432A(GTG→GCG)	dcuD	b3227	C	C	C	predicted transporter
SNP	3372027	A→G	V25A(GTC→GCC)	sspA	b3229	G	G	G	stringent starvation protein A
INS	3373939	+C	coding (739/1128 nt)	yhcM	b3232	+C	+C	+C	conserved protein with nucleoside triphosphate hydrolase domain
SNP	3374416	T→G	M88L(ATG→CTG)	yhcM	b3232	-	G	G	conserved protein with nucleoside triphosphate hydrolase domain
SNP	3377881	A→C	V334V(GTA→GTC)	degS	b3235	C	C	C	serine endoprotease, periplasmic
SNP	3381338	T→C	T511A(ACC→GCC)	aaeB	b3240	C	C	C	p-hydroxybenzoic acid efflux system component
SNP	3381585	A→C	N428K(AAT→AAG)	aaeB	b3240	-	-	C	p-hydroxybenzoic acid efflux system component
SNP	3382653	G→A	I72I(ATC→ATT)	aaeB	b3240	A	A	A	p-hydroxybenzoic acid efflux system component
SNP	3383864	T→C	T52A(ACC→GCC)	aaeX	b3242	-	-	C	membrane protein of efflux system
SNP	3384290	T→G	S31A(TCG→GCG)	aaeR	b3243	-	-	G	transcriptional regulator for aaeXAB operon
SNP	3389386	A→C	L469W(TTG→TGG)	yhdP	b4472	-	-	C	conserved membrane protein, predicted transporter
SNP	3392139	C→T	G113S(GGC→AGC)	rng	b3247	T	T	T	ribonuclease G
SNP	3394215	G→A	G148G(GGC→GGT)	mreC	b3250	A	A	A	cell wall structural complex MreBCD transmembrane component MreC
SNP	3395576	G→A	G64G(GGC→GGT)	mreB	b3251	-	A	A	cell wall structural complex MreBCD, actin-like component MreB
SNP	3396397	A→C	L539*(TTA→TGA)	csrD	b3252	-	-	C	targeting factor for csrBC sRNA degradation
SNP	3398286	T→G	Y41*(TAT→TAG)	yhdH	b3253	-	G	G	predicted oxidoreductase, Zn-dependent and NAD(P)-binding
SNP	3410472	A→C	N254H(AAC→CAC)	acrF	b3266	-	C	C	multidrug efflux system protein
SNP	3413036	A→G	intergenic (+219/-34)	acrF/yhdV	b3266/b3267	G	G	G	multidrug efflux system protein/predicted outer membrane protein
SNP	3424146	T→G	D77E(GAT→GAG)	yrdA	b3279	-	G	G	conserved protein
SNP	3424518	G→A	D34D(GAC→GAT)	yrdB	b3280	A	A	A	conserved protein
SNP	3431613	A→C	Y139S(TAT→TCT)	trkA	b3290	-	C	C	NAD-binding component of TrK potassium transporter
SNP	3439597	T→G	I105L(ATC→CTC)	rpsE	b3303	G	G	G	30S ribosomal subunit protein S5
SNP	3440574	A→C	F83V(TTC→GTC)	rplF	b3305	-	-	C	50S ribosomal subunit protein L6
SNP	3443750	T→G	K34Q(AAA→CAA)	rplP	b3313	G	G	G	50S ribosomal subunit protein L16
SNP	3444319	T→G	E82A(GAA→GCA)	rpsC	b3314	-	G	G	30S ribosomal subunit protein S3
SNP	3449533	T→C	R182R(AGA→AGG)	gspA	b3323	C	C	C	general secretory pathway component, cryptic
SNP	3452854	T→G	Y600D(TAT→GAT)	gspD	b3325	-	-	G	general secretory pathway component, cryptic
SNP	3458148	T→G	L182R(CTT→CGT)	gspK	b3332	G	G	G	general secretory pathway component, cryptic
SNP	3468809	T→G	intergenic (-48/+49)	rpsG/rpsL	b3341/b3342	G	G	G	30S ribosomal subunit protein S7/30S ribosomal subunit protein S12
INS	3471121	+A	intergenic (-1/+166)	yheO/fkpA	b3346/b3347	+A	+A	+A	conserved protein/FKBP-type peptidyl-prolyl cis-trans isomerase (rotamase)
SNP	3474149	A→C	L380R(CTA→CGA)	kefB	b3350	-	C	C	potassium:proton antiporter
SNP	3477182	A→G	E405G(GAG→GGG)	yheS	b3352	G	G	G	fused predicted transporter subunits of ABC superfamily: ATP-binding components
SNP	3479173	T→C	S2P(TCT→CCT)	prkB	b3355	C	C	C	predicted phosphoribulokinase
SNP	3488333	T→G	L362L(CTT→CTG)	tsgA	b3364	-	-	G	predicted transporter
SNP	3492228	T→G	A182A(GCT→GCG)	nirC	b3367	-	G	G	nitrite transporter
SNP	3493530	T→C	G285G(GGT→GGC)	cysG	b3368	-	-	C	fused siroheme synthase 1,3- dimethyluroporphyriongen III dehydrogenase and siroheme ferrochelatase/uroporphyrinogen methyltransferase
SNP	3495229	A→C	I214L(ATC→CTC)	frlA	b3370	-	-	C	predicted fructoselysine transporter
SNP	3501114	A→G	G301G(GGT→GGC)	yhfT	b3377	-	-	G	predicted inner membrane protein
SNP	3503443	T→C	T351A(ACC→GCC)	yhfW	b3380	C	C	C	predicted mutase
INS	3508568	+T	coding (502/759 nt)	gph	b3385	+T	+T	+T	phosphoglycolate phosphatase
SNP	3509291	A→C	F150C(TTC→TGC)	rpe	b3386	C	C	C	D-ribulose-5-phosphate 3-epimerase
SNP	3511163	T→G	E275A(GAG→GCG)	damX	b3388	-	G	G	cell division protein that binds to the septal ring
SNP	3513892	A→C	intergenic (-148/+253)	aroK/hofQ	b3390/b3391	-	-	C	shikimate kinase I/protein required for the utilization of DNA as a carbon source; fimbrial transporter homolog
SNP	3516445	G→A	P70S(CCA→TCA)	hofN	b3394	A	A	A	protein required for the utilization of DNA as a carbon source; fimbrial assembly protein homolog
SNP	3518251	A→C	Y242S(TAT→TCT)	mrcA	b3396	-	C	C	fused penicillin-binding protein 1a: murein transglycosylase/murein transpeptidase
SNP	3523986	A→C	D213A(GAC→GCC)	yrfG	b3399	C	C	C	GMP/IMP nucleotidase
SNP	3524065	A→C	K13T(AAA→ACA)	hslR	b3400	C	C	C	ribosome-associated heat shock protein Hsp15
SNP	3538927	A→C	L200V(TTG→GTG)	bioH	b3412	-	C	C	pimeloyl-ACP carboxylesterase
SNP	3542440	T→G	F401C(TTC→TGC)	gntT	b3415	G	G	G	gluconate transporter, high-affinity GNT I system
DEL	3544467	Δ1bp	coding (284/2085 nt)	malQ	b3416	Δ1bp	Δ1bp	Δ1bp	4-alpha-glucanotransferase (amylomaltase)
SNP	3545251	C→T	A635T(GCA→ACA)	malP	b3417	T	T	T	maltodextrin phosphorylase
SNP	3545368	T→G	I596L(ATC→CTC)	malP	b3417	-	G	G	maltodextrin phosphorylase
SNP	3551529	T→G	M1M(ATG→CTG)	rtcA	b4475	-	G	G	RNA 3'-terminal phosphate cyclase
SNP	3552845	T→G	intergenic (-86/-103)	rtcB/rtcR	b3421/b3422	-	-	G	conserved protein/sigma 54-dependent transcriptional regulator of rtcBA expression
SNP	3556238	A→C	I89S(ATT→AGT)	glpE	b3425	C	C	C	thiosulfate:cyanide sulfurtransferase (rhodanese)
SNP	3557683	A→C	T331P(ACC→CCC)	glpD	b3426	-	C	C	sn-glycerol-3-phosphate dehydrogenase, aerobic, FAD/NAD(P)-binding
SNP	3559004	T→C	H753R(CAC→CGC)	glgP	b3428	C	C	C	glycogen phosphorylase
SNP	3563908	T→G	K34T(AAG→ACG)	glgC	b3430	G	G	G	glucose-1-phosphate adenylyltransferase
SNP	3569771	A→C	A7A(GCA→GCC)	yhgN	b3434	-	-	C	predicted antibiotic transporter
SNP	3574838	A→C	Y216*(TAT→TAG)	yhhX	b3440	-	-	C	predicted oxidoreductase with NAD(P)-binding Rossmann-fold domain
SNP	3587746	A→C	F124L(TTT→TTG)	livF	b3454	-	C	C	leucine/isoleucine/valine transporter subunit
SNP	3593277	T→G	V354V(GTA→GTC)	livJ	b3460	-	-	G	leucine/isoleucine/valine transporter subunit
SNP	3595434	T→C	L10L(TTA→TTG)	rpoH	b3461	C	C	C	RNA polymerase, sigma 32 (sigma H) factor
SNP	3596144	T→C	D208G(GAC→GGC)	ftsX	b3462	-	C	C	predicted transporter subunit: membrane component of ABC superfamily
SNP	3597731	T→C	K398R(AAG→AGG)	ftsY	b3464	-	-	C	Signal Recognition Particle (SRP) receptor
SNP	3604474	T→G	F193C(TTC→TGC)	yhhQ	b3471	G	G	G	inner membrane protein, DUF165 family
SNP	3605244	T→G	L390F(TTA→TTC)	yhhS	b3473	-	-	G	inner membrane protein, predicted transporter
DEL	3605468	Δ1bp	coding (946/1218 nt)	yhhS	b3473	Δ1bp	Δ1bp	Δ1bp	inner membrane protein, predicted transporter
SNP	3606105	T→C	A103A(GCA→GCG)	yhhS	b3473	C	C	C	inner membrane protein, predicted transporter
SNP	3608621	A→C	D92A(GAC→GCC)	nikA	b3476	C	C	C	Nickel-binding, heme-binding periplasmic protein
SNP	3613357	A→C	E30D(GAA→GAC)	nikR	b3481	-	-	C	DNA-binding transcriptional repressor, Ni- binding
SNP	3613666	T→G	D133E(GAT→GAG)	nikR	b3481	-	G	G	DNA-binding transcriptional repressor, Ni- binding
SNP	3626172	T→G	K367Q(AAA→CAA)	yhiJ	b3488	-	G	G	predicted protein
SNP	3626508	T→G	N255H(AAC→CAC)	yhiJ	b3488	G	G	G	predicted protein
SNP	3627489	T→G	intergenic (-219/+43)	yhiJ/yhiL	b3488/b4660	-	-	G	predicted protein/pseudogene
SNP	3630124	T→G	C235G(TGT→GGT)	yhiM	b3491	-	G	G	inner membrane protein, DUF1323 family
INS	3630694	+T	intergenic (+121/+194)	yhiM/yhiN	b3491/b3492	+T	+T	+T	inner membrane protein, DUF1323 family/predicted oxidoreductase with FAD/NAD(P)-binding domain
DEL	3634552	Δ1bp	intergenic (-152/-239)	uspB/uspA	b3494/b3495	-	Δ1bp	Δ1bp	universal stress (ethanol tolerance) protein B/universal stress global response regulator
SNP	3639772	T→G	N31H(AAC→CAC)	prlC	b3498	G	G	G	oligopeptidase A
INS	3645353	+C	intergenic (+11/-618)	arsC/yhiS	b3503/b3504	-	+C	-	arsenate reductase/pseudogene
SNP	3649275	A→C	intergenic (+68/-88)	slp/dctR	b3506/b3507	-	-	C	outer membrane lipoprotein/predicted DNA-binding ranscriptional regulator
SNP	3653333	A→C	K96N(AAA→AAC)	gadE	b3512	-	C	C	DNA-binding transcriptional activator
SNP	3655205	A→C	I38L(ATT→CTT)	mdtF	b3514	-	-	C	anaerobic multidrug efflux transporter, ArcA- regulated
SNP	3657265	G→A	M724I(ATG→ATA)	mdtF	b3514	-	-	A	anaerobic multidrug efflux transporter, ArcA- regulated
SNP	3659628	T→C	intergenic (-330/+37)	gadW/gadX	b3515/b3516	C	C	C	transcriptional activator of gadA and gadBC in absence of GadX/DNA-binding transcriptional dual regulator
DEL	3663218	Δ1bp	coding (650/1398 nt)	yhjA	b3518	-	Δ1bp	Δ1bp	predicted cytochrome C peroxidase
SNP	3665941	A→C	intergenic (+21/+30)	treF/yhjB	b3519/b3520	C	C	C	cytoplasmic trehalase/predicted two-component system response regulator, LuxR-like HTH domain, function unknown
SNP	3668216	A→C	E59A(GAA→GCA)	yhjD	b3522	-	C	C	putative alternate lipid exporter, suppressor of msbA and KDO essentiality, inner membrane protein
SNP	3670992	T→G	I680L(ATT→CTT)	yhjG	b3524	-	G	G	Inner membrane protein, AsmA family
SNP	3671638	A→C	I464M(ATT→ATG)	yhjG	b3524	-	-	C	Inner membrane protein, AsmA family
SNP	3675703	A→C	F306C(TTC→TGC)	yhjJ	b3527	C	C	C	predicted zinc-dependent peptidase
INS	3684805	+G	coding (136/1107 nt)	bcsZ	b3531	-	+G	+G	endo-1,4-D-glucanase
DEL	3686830	Δ1bp	coding (457/2340 nt)	bcsB	b3532	-	-	Δ1bp	regulator of cellulose synthase, cyclic di-GMP binding
SNP	3689002	T→G	K305T(AAG→ACG)	bcsA	b3533	-	-	G	cellulose synthase, catalytic subunit
SNP	3692468	T→C	N444N(AAT→AAC)	bcsE	b3536	-	-	C	cellulose production protein
SNP	3696944	T→G	N202H(AAT→CAT)	dppF	b3540	-	-	G	dipeptide transporter
SNP	3711219	A→C	intergenic (-200/-7)	bisC/yiaD	b3551/b3552	C	C	C	biotin sulfoxide reductase/multicopy suppressor of bamB; outer membrane lipoprotein
SNP	3712132	T→G	G48G(GGT→GGG)	ghrB	b3553	G	G	G	glyoxylate/hydroxypyruvate reductase B
SNP	3713000	G→A	intergenic (+37/+13)	ghrB/yiaF	b3553/b3554	A	A	A	glyoxylate/hydroxypyruvate reductase B/conserved protein
SNP	3717170	T→C	D636G(GAT→GGT)	glyS	b3559	C	C	C	glycine tRNA synthetase, beta subunit
SNP	3720008	G→A	intergenic (-11/+84)	glyQ/ysaB	b3560/b4553	-	-	A	glycine tRNA synthetase, alpha subunit/predicted protein
SNP	3720808	A→C	L81F(TTA→TTC)	wecH	b3561	C	C	C	O-acetyltransferase for enterobacterial common antigen (ECA)
DEL	3722790	Δ1bp	coding (1261/1455 nt)	xylB	b3564	-	Δ1bp	Δ1bp	xylulokinase
SNP	3731078	A→C	F260C(TTC→TGC)	bax	b3570	-	C	C	conserved protein
SNP	3732649	A→C	Q158H(CAA→CAC)	malS	b3571	-	C	C	alpha-amylase
SNP	3734937	T→C	V185A(GTC→GCC)	avtA	b3572	C	C	C	valine-pyruvate aminotransferase 1
SNP	3738250	A→C	K280T(AAG→ACG)	yiaK	b3575	C	C	C	2,3-diketo-L-gulonate reductase, NADH-dependent
SNP	3740654	A→C	D391A(GAT→GCT)	yiaN	b3578	C	C	C	L-dehydroascorbate transporter, TRAP permease large subunit for TRAP (TRipartite ATP-independent Periplasmic) family transporter YiaMNO
SNP	3743770	T→G	I172S(ATT→AGT)	yiaQ	b3581	-	-	G	3-keto-L-gulonate 6-phosphate decarboxylase

SNP	3747299	T→G	I210S(ATC→AGC)	yiaU	b3585	-	G	G	predicted DNA-binding transcriptional regulator
SNP	3748637	T→G	N48H(AAC→CAC)	yiaV	b3586	G	G	G	membrane fusion protein (MFP) component of efflux pump, signal anchor
SNP	3750715	T→G	E159A(GAA→GCA)	aldB	b3588	G	G	G	aldehyde dehydrogenase B
SNP	3751021	G→A	A57V(GCG→GTG)	aldB	b3588	A	A	A	aldehyde dehydrogenase B
SNP	3755880	G→A	R17C(CGC→TGC)	selA	b3591	A	A	A	selenocysteine synthase
SNP	3756832	A→G	intergenic (-198/-30)	yibF/rhsA	b3592/b3593	-	-	G	glutathione S-transferase homolog/rhsA element core protein RshA
SNP	3756939	G→A	V26V(GTG→GTA)	rhsA	b3593	-	-	A	rhsA element core protein RshA
SNP	3756942	C→T	R27R(CGC→CGT)	rhsA	b3593	-	-	T	rhsA element core protein RshA
SNP	3761079	A→C	K22Q(AAA→CAA)	yibA	b3594	-	C	C	Predicted lyase containing HEAT-repeat
INS	3761710	+A	coding (695/744 nt)	yibA	b3594	+A	+A	+A	Predicted lyase containing HEAT-repeat
SNP	3763861	T→G	pseudogene (325/398 nt)	yibW	b4651	-	-	G	pseudogene, rhsA-linked
DEL	3787453	Δ1bp	intergenic (-224/+51)	kbl/yibB	b3617/b3618	Δ1bp	Δ1bp	Δ1bp	glycine C-acetyltransferase/conserved protein, pfam09612 family
SNP	3789372	T→C	G236G(GGT→GGC)	rfaD	b3619	-	C	C	ADP-L-glycero-D-mannoheptose-6-epimerase, NAD(P)- binding
SNP	3792857	T→G	I411S(ATT→AGT)	rfaL	b3622	G	G	G	O-antigen ligase
DEL	3794503	Δ1bp	coding (372/852 nt)	rfaZ	b3624	Δ1bp	Δ1bp	Δ1bp	lipopolysaccharide core biosynthesis protein
DEL	3794939	Δ1bp	intergenic (-65/+6)	rfaZ/rfaY	b3624/b3625	Δ1bp	Δ1bp	Δ1bp	lipopolysaccharide core biosynthesis protein/lipopolysaccharide core biosynthesis protein
SNP	3797435	G→A	P101L(CCT→CTT)	rfaI	b3627	A	A	A	UDP-D-galactose:(glucosyl)lipopolysaccharide- alpha-1,3-D-galactosyltransferase
SNP	3803400	T→G	T61T(ACT→ACG)	waaA	b3633	-	-	G	3-deoxy-D-manno-octulosonic-acid transferase (KDO transferase)
SNP	3803539	T→C	Y108H(TAT→CAT)	waaA	b3633	-	C	C	3-deoxy-D-manno-octulosonic-acid transferase (KDO transferase)
SNP	3804104	T→C	V296A(GTC→GCC)	waaA	b3633	C	C	C	3-deoxy-D-manno-octulosonic-acid transferase (KDO transferase)
SNP	3806416	T→G	intergenic (-64/+153)	rpmB/yicR	b3637/b3638	G	G	G	50S ribosomal subunit protein L28/conserved protein, UPF0758 family; not radC
SNP	3808793	T→G	S62A(TCA→GCA)	dut	b3640	-	-	G	deoxyuridinetriphosphatase
SNP	3819671	A→C	E160A(GAA→GCA)	trmH	b3651	-	C	C	tRNA mG18-2'-O-methyltransferase, SAM-dependen
SNP	3826249	T→G	M372R(ATG→AGG)	yicH	b3655	-	-	G	conserved protein
SNP	3829906	A→G	G234G(GGT→GGC)	yicJ	b3657	-	G	G	predicted transporter
SNP	3833075	T→G	I50M(ATT→ATG)	yicL	b3660	G	G	G	predicted inner membrane protein
INS	3834115	+C	coding (557/819 nt)	nlpA	b3661	-	+C	+C	cytoplasmic membrane lipoprotein-28
SNP	3838131	T→C	T113A(ACC→GCC)	yicO	b3664	-	-	C	Probable adenine permease
SNP	3847072	A→C	S131A(TCT→GCT)	ilvB	b3671	-	C	C	acetolactate synthase I, large subunit
SNP	3854112	T→G	H228P(CAC→CCC)	yidK	b3679	-	-	G	predicted transporter
SNP	3854704	A→C	S31A(TCG→GCG)	yidK	b3679	C	C	C	predicted transporter
DEL	3861805	Δ12bp	coding (285-296/414 nt)	ibpA	b3687	Δ12bp	Δ12bp	Δ12bp	heat shock chaperone
SNP	3865044	A→C	R330R(CGA→CGC)	cbrA	b3690	C	C	C	predicted oxidoreductase with FAD/NAD(P)-binding domain
SNP	3867023	T→C	E218E(GAA→GAG)	dgoD	b4478	C	-	-	galactonate dehydratase
SNP	3873372	A→C	C476G(TGT→GGT)	gyrB	b3699	-	-	C	DNA gyrase, subunit B
SNP	3880070	T→G	F106C(TTT→TGT)	yidC	b3705	G	G	G	membrane protein insertase
SNP	3888928	T→G	S124A(TCC→GCC)	yieE	b3712	G	G	G	predicted phosphopantetheinyl transferase, COG2091 family
SNP	3898220	T→C	I54V(ATC→GTC)	bglB	b3721	-	C	C	cryptic phospho-beta-glucosidase B
SNP	3899709	A→C	A189A(GCT→GCG)	bglF	b3722	-	-	C	fused beta-glucoside-specific PTS enzymes: IIA component/IIB component/IIC component
SNP	3900750	T→G	M166L(ATG→CTG)	bglG	b3723	-	-	G	transcriptional antiterminator of the bgl operon
DEL	3901102	Δ1bp	coding (144/837 nt)	bglG	b3723	Δ1bp	Δ1bp	Δ1bp	transcriptional antiterminator of the bgl operon
SNP	3905177	T→G	K343Q(AAG→CAG)	pstS	b3728	G	G	G	periplasmic phosphate binding protein, high- affinity
SNP	3912719	G→A	R75R(CGC→CGT)	atpG	b3733	A	A	A	F1 sector of membrane-bound ATP synthase, gamma subunit
SNP	3914013	T→G	K175Q(AAA→CAA)	atpA	b3734	-	G	G	F1 sector of membrane-bound ATP synthase, alpha subunit
SNP	3915694	A→C	G58G(GGT→GGG)	atpE	b3737	C	C	C	F0 sector of membrane-bound ATP synthase, subunit c
SNP	3917799	T→G	K158T(AAA→ACA)	rsmG	b3740	-	G	G	16S rRNA m(7)G527 methyltransferase, SAM- dependent; glucose-inhibited cell-division protein
SNP	3918820	G→A	H498Y(CAC→TAC)	mnmG	b3741	A	A	A	5-methylaminomethyl-2-thiouridine modification at tRNA U34
SNP	3923391	A→G	G297G(GGT→GGC)	viaA	b3745	G	G	G	predicted von Willibrand factor containing protein
SNP	3926359	T→G	A122A(GCT→GCG)	kup	b3747	-	G	G	potassium transporter
SNP	3929269	T→G	S272A(TCT→GCT)	rbsA	b3749	G	G	G	fused D-ribose transporter subunits of ABC superfamily: ATP-binding components
SNP	3932902	C→T	intergenic (+1/-12)	rbsK/rbsR	b3752/b3753	T	T	T	ribokinase/DNA-binding transcriptional repressor of ribose metabolism
SNP	3943677	T→G	H324P(CAT→CCT)	yifB	b3765	-	G	G	predicted bifunctional enzyme and transcriptional regulator
SNP	3976422	T→G	I286M(ATT→ATG)	yifK	b3795	-	G	G	predicted transporter
INS	3977354	+C	noncoding (71/87 nt)	leuT	b3798	-	+C	+C	tRNA-Leu
SNP	3978282	A→C	K216T(AAG→ACG)	aslB	b3800	C	C	C	predicted regulator of arylsulfatase activity
SNP	3987642	A→G	E578E(GAA→GAG)	cyaA	b3806	G	G	G	adenylate cyclase
SNP	3988852	A→C	intergenic (-115/-348)	cyaY/yifL	b3807/b4558	C	C	C	iron-dependent inhibitor of iron-sulfur cluster formation; frataxin; iron-binding and oxidizing protein/predicted lipoprotein
SNP	3994657	A→G	N666S(AAC→AGC)	uvrD	b3813	G	G	G	DNA-dependent ATPase I and helicase II
SNP	4013819	A→G	N18S(AAC→AGC)	tatB	b3838	-	G	G	TatABCE protein translocation system subunit
SNP	4019303	A→C	L259*(TTA→TGA)	fadA	b3845	C	C	C	3-ketoacyl-CoA thiolase (thiolase I)
SNP	4020661	A→C	Y539*(TAT→TAG)	fadB	b3846	C	C	C	fused 3-hydroxybutyryl-CoA epimerase/delta(3)- cis-delta(2)-trans-enoyl-CoA isomerase/enoyl-CoA hydratase/3-hydroxyacyl-CoA dehydrogenase
SNP	4020798	A→C	C494G(TGC→GGC)	fadB	b3846	-	-	C	fused 3-hydroxybutyryl-CoA epimerase/delta(3)- cis-delta(2)-trans-enoyl-CoA isomerase/enoyl-CoA hydratase/3-hydroxyacyl-CoA dehydrogenase
SNP	4023638	A→C	E391A(GAA→GCA)	pepQ	b3847	-	-	C	proline dipeptidase
SNP	4032075	T→G	intergenic (+133/+137)	rrfA/mobB	b3855/b3856	G	-	-	5S ribosomal RNA of rrnA operon/molybdopterin-guanine dinucleotide biosynthesis protein B
SNP	4032078	C→G	intergenic (+136/+134)	rrfA/mobB	b3855/b3856	G	-	-	5S ribosomal RNA of rrnA operon/molybdopterin-guanine dinucleotide biosynthesis protein B
SNP	4034495	G→A	D259N(GAC→AAC)	rdoA	b3859	A	A	A	Thr/Ser kinase implicated in Cpx stress response
SNP	4047175	T→G	K157N(AAA→AAC)	glnL	b3869	G	G	G	sensory histidine kinase in two-component regulatory system with GlnG
SNP	4049160	T→G	I61L(ATT→CTT)	glnA	b3870	-	G	G	glutamine synthetase
SNP	4057264	T→C	T413A(ACC→GCC)	yihP	b3877	-	C	C	predicted transporter
SNP	4060515	T→G	H23P(CAT→CCT)	yihQ	b3878	-	G	G	alpha-glucosidase
SNP	4064861	A→C	F6C(TTT→TGT)	yihU	b3882	-	C	C	gamma-hydroxybutyrate dehydrogenase, NADH- dependent
SNP	4067329	A→G	S157S(TCA→TCG)	yihX	b3885	-	G	G	alpha-D-Glucose-1-P phosphatase, anomer- specific
SNP	4077101	T→G	K10Q(AAG→CAG)	fdoG	b3894	G	G	G	formate dehydrogenase-O, large subunit
SNP	4077797	A→C	D105A(GAT→GCT)	fdhD	b3895	-	-	C	formate dehydrogenase formation protein
SNP	4079297	A→C	E330D(GAA→GAC)	yiiG	b3896	C	C	C	conserved lipoprotein
SNP	4089027	C→T	intergenic (-273/-15)	rhaB/rhaS	b3904/b3905	T	T	T	rhamnulokinase/DNA-binding transcriptional activator for rhaBAD and rhaT, L-rhamnose-binding
SNP	4094124	A→G	intergenic (+145/-4)	kdgT/yiiM	b3909/b3910	G	G	G	2-keto-3-deoxy-D-gluconate transporter/6-N-hydroxylaminopurine resistance protein
SNP	4094417	A→C	N97T(AAT→ACT)	yiiM	b3910	-	-	C	6-N-hydroxylaminopurine resistance protein
SNP	4098099	A→G	T109A(ACA→GCA)	fieF	b3915	G	G	G	ferrous iron and zinc transporter
SNP	4099314	C→T	R153C(CGT→TGT)	pfkA	b3916	T	T	T	6-phosphofructokinase I
SNP	4100334	T→G	G65G(GGT→GGG)	sbp	b3917	G	G	G	sulfate transporter subunit
SNP	4101001	A→C	K288Q(AAA→CAA)	sbp	b3917	-	-	C	sulfate transporter subunit
SNP	4101822	T→G	L196R(CTG→CGG)	cdh	b3918	-	-	G	CDP-diacylglycerol phosphotidylhydrolase
SNP	4105206	A→G	S191S(AGT→AGC)	fpr	b3924	G	G	G	ferredoxin-NADP reductase
SNP	4111928	T→G	I376L(ATC→CTC)	hslU	b3931	-	G	G	molecular chaperone and ATPase component of HslUV protease
SNP	4119160	A→C	V14G(GTA→GGA)	yiiX	b3937	C	C	C	conserved protein, DUF830 family
SNP	4122894	A→C	Q585P(CAG→CCG)	metL	b3940	C	C	C	fused aspartokinase II/homoserine dehydrogenase II
SNP	4126417	A→C	E426A(GAA→GCA)	katG	b3942	C	C	C	catalase-peroxidase HPI, heme b-containing
SNP	4127051	A→C	V637V(GTA→GTC)	katG	b3942	C	C	C	catalase-peroxidase HPI, heme b-containing
SNP	4133096	A→G	G144G(GGT→GGC)	ptsA	b3947	-	G	G	fused predicted PTS enzymes: Hpr component/enzyme I component/enzyme IIA component
SNP	4133354	C→T	T58T(ACG→ACA)	ptsA	b3947	T	T	T	fused predicted PTS enzymes: Hpr component/enzyme I component/enzyme IIA component
SNP	4142096	A→G	V770A(GTC→GCC)	ppc	b3956	G	G	G	phosphoenolpyruvate carboxylase
SNP	4144876	G→A	intergenic (-472/+126)	ppc/argE	b3956/b3957	-	-	A	phosphoenolpyruvate carboxylase/acetylornithine deacetylase
SNP	4151277	T→G	N274H(AAT→CAT)	sthA	b3962	-	G	G	pyridine nucleotide transhydrogenase, soluble
SNP	4170146	A→G	T132A(ACT→GCT)	rplK	b3983	G	G	G	50S ribosomal subunit protein L11
SNP	4174834	A→C	N762H(AAC→CAC)	rpoB	b3987	-	C	C	RNA polymerase, beta subunit
SNP	4183776	C→T	E56K(GAA→AAA)	thiG	b3991	T	T	T	thiamin biosynthesis ThiGH complex subunit
SNP	4184302	A→C	V194G(GTC→GGC)	thiF	b3992	C	C	C	adenylyltransferase, modifies ThiS C-terminus
SNP	4187235	G→A	G57G(GGC→GGT)	thiC	b3994	A	A	A	thiamin (pyrimidine moiety) biosynthesis protein
SNP	4189591	T→G	T190T(ACT→ACG)	hemE	b3997	G	G	G	uroporphyrinogen decarboxylase
INS	4191455	+C	intergenic (+55/-132)	yjaG/hupA	b3999/b4000	-	+C	+C	conserved protein/HU, DNA-binding transcriptional regulator, alpha subunit
SNP	4193334	A→C	I35L(ATT→CTT)	zraS	b4003	C	C	C	sensory histidine kinase in two-component regulatory system with ZraR
SNP	4208719	C→T	A102V(GCG→GTG)	aceA	b4015	T	T	T	isocitrate lyase
SNP	4208793	A→G	T127A(ACC→GCC)	aceA	b4015	G	G	G	isocitrate lyase
SNP	4213725	G→A	H23H(CAC→CAT)	arpA	b4017	A	A	A	ankyrin repeat protein
SNP	4214197	A→G	R246R(CGT→CGC)	iclR	b4018	-	G	G	DNA-binding transcriptional repressor
SNP	4217666	A→C	K823Q(AAA→CAA)	metH	b4019	-	-	C	homocysteine-N5-methyltetrahydrofolate transmethylase, B12-dependent
SNP	4233360	A→G	G67G(GGT→GGC)	xylE	b4031	G	G	G	D-xylose transporter
SNP	4237747	A→C	intergenic (-22/-343)	malE/malK	b4034/b4035	C	C	C	maltose transporter subunit/fused maltose transport subunit, ATP-binding component of ABC superfamily/regulatory protein
SNP	4243410	A→C	pseudogene (1402/1581 nt)	yjbI	b4038	C	C	C	pseudogene, pentapeptide repeat-related
SNP	4243819	A→G	H3R(CAC→CGC)	ubiC	b4039	G	G	G	chorismate--pyruvate lyase
SNP	4248144	A→C	I68L(ATT→CTT)	dgkA	b4042	-	-	C	diacylglycerol kinase
SNP	4251758	A→C	I44I(ATA→ATC)	yjbL	b4047	C	C	C	predicted protein
SNP	4253062	G→A	V15I(GTT→ATT)	dusA	b4049	A	A	A	tRNA-dihydrouridine synthase A
SNP	4260467	A→C	intergenic (-149/-253)	yjbS/aphA	b4621/b4055	-	-	C	hypothetical protein/acid phosphatase/phosphotransferase, class B, non-specific
SNP	4264513	T→G	E222A(GAA→GCA)	uvrA	b4058	-	G	G	ATPase and DNA damage recognition protein of nucleotide excision repair excinuclease UvrABC
SNP	4265066	A→C	S38A(TCC→GCC)	uvrA	b4058	-	-	C	ATPase and DNA damage recognition protein of nucleotide excision repair excinuclease UvrABC
SNP	4280860	T→C	Y104H(TAC→CAC)	nrfB	b4071	C	C	C	nitrite reductase, formate-dependent, penta-heme cytochrome c
SNP	4284426	A→C	I520L(ATT→CTT)	nrfE	b4074	-	C	C	heme lyase (NrfEFG) for insertion of heme into c552, subunit NrfE
SNP	4286045	T→G	Y87D(TAC→GAC)	gltP	b4077	G	G	G	glutamate/aspartate:proton symporter
INS	4287686	+CG	intergenic (+586/+56)	gltP/yjcO	b4077/b4078	+CG	+CG	+CG	glutamate/aspartate:proton symporter/conserved protein
SNP	4288716	T→C	T653A(ACC→GCC)	fdhF	b4079	C	C	C	formate dehydrogenase-H, selenopolypeptide subunit
SNP	4295378	A→C	F13C(TTC→TGC)	mdtN	b4082	-	C	C	membrane fusion protein of efflux pump
SNP	4299800	T→G	K326Q(AAG→CAG)	alsC	b4086	-	-	G	D-allose transporter subunit
SNP	4309087	G→A	L39F(CTC→TTC)	phnL	b4096	A	A	A	carbon-phosphorus lyase complex subunit
SNP	4309541	T→G	D177A(GAT→GCT)	phnK	b4097	G	G	G	carbon-phosphorus lyase complex subunit
SNP	4313261	C→T	W156*(TGG→TGA)	phnF	b4102	-	-	T	predicted DNA-binding transcriptional regulator of phosphonate uptake and biodegradation
SNP	4316707	A→C	M114R(ATG→AGG)	yjdN	b4107	C	C	C	conserved protein
SNP	4320501	A→G	L687L(TTA→TTG)	yjdA	b4109	G	G	G	mutational suppressor of yhjH motility mutation, function unknown; related to Dynamin GTPase
SNP	4325286	A→C	D537E(GAT→GAG)	eptA	b4114	-	C	C	predicted metal dependent hydrolase
SNP	4326479	A→C	C140G(TGC→GGC)	eptA	b4114	-	C	C	predicted metal dependent hydrolase
DEL	4329416	Δ1bp	intergenic (-181/+144)	adiY/adiA	b4116/b4117	-	Δ1bp	Δ1bp	DNA-binding transcriptional activator/arginine decarboxylase
SNP	4329862	A→C	S656A(TCC→GCC)	adiA	b4117	-	-	C	arginine decarboxylase
SNP	4332012	T→C	intergenic (-185/+14)	adiA/melR	b4117/b4118	C	C	C	arginine decarboxylase/DNA-binding transcriptional dual regulator
SNP	4336521	A→C	V115G(GTG→GGG)	yjdF	b4121	-	-	C	conserved inner membrane protein
SNP	4348154	A→G	A590A(GCT→GCC)	cadA	b4131	G	G	G	lysine decarboxylase, acid-inducible
SNP	4350857	T→G	T161P(ACT→CCT)	cadB	b4132	-	-	G	predicted lysine/cadaverine transporter
SNP	4351175	A→G	Y55H(TAT→CAT)	cadB	b4132	G	G	G	predicted lysine/cadaverine transporter
SNP	4351331	A→C	S3A(TCT→GCT)	cadB	b4132	-	-	C	predicted lysine/cadaverine transporter
DEL	4352747	Δ1bp	coding (494/1539 nt)	cadC	b4133	Δ1bp	Δ1bp	Δ1bp	DNA-binding transcriptional activator
SNP	4356245	T→G	Q35P(CAA→CCA)	dsbD	b4136	-	-	G	fused thiol:disulfide interchange protein: activator of DsbC/conserved protein
SNP	4360227	A→G	L43L(TTA→TTG)	fxsA	b4140	G	G	G	suppressor of F exclusion of phage T7
SNP	4362688	A→C	I120L(ATC→CTC)	groL	b4143	-	C	C	Cpn60 chaperonin GroEL, large subunit of GroESL
SNP	4364520	T→C	intergenic (+52/+151)	yjeI/yjeJ	b4144/b4145	C	C	C	conserved protein/predicted protein
SNP	4364725	C→T	A272A(GCG→GCA)	yjeJ	b4145	T	T	T	predicted protein
SNP	4365230	T→G	E104A(GAA→GCA)	yjeJ	b4145	-	-	G	predicted protein
SNP	4365915	T→G	intergenic (-375/+20)	yjeJ/yjeK	b4145/b4146	-	G	G	predicted protein/EF-P lysylation protein; weak lysine 2,3- aminomutase
DEL	4380628	Δ1bp	coding (25/3300 nt)	yjeP	b4159	-	Δ1bp	Δ1bp	predicted mechanosensitive channel
INS	4383574	+G	intergenic (+119/-92)	orn/glyV	b4162/b4163	-	+G	+G	oligoribonuclease/tRNA-Gly
SNP	4384006	T→G	intergenic (+42/-192)	glyY/yjeV	b4165/b4670	-	-	G	tRNA-Gly/expressed protein
SNP	4385599	C→T	G76G(GGC→GGT)	yjeF	b4167	T	T	T	predicted carbohydrate kinase
SNP	4387286	C→T	G132G(GGC→GGT)	yjeE	b4168	T	T	T	ADP-binding protein essential for nucleoid integrity
SNP	4392875	A→G	T277A(ACG→GCG)	hflX	b4173	G	G	G	GTPase, stimulated by 50S subunit binding
SNP	4397781	T→G	C80G(TGC→GGC)	nsrR	b4178	G	G	G	nitric oxide-sensitive repressor for NO regulon
SNP	4398281	T→G	L108V(TTG→GTG)	rnr	b4179	-	G	G	exoribonuclease R, RNase R
SNP	4400818	T→G	Y80D(TAT→GAT)	rlmB	b4180	G	G	G	23S rRNA (Gm2251)-methyltransferase
SNP	4402380	A→G	K174K(AAA→AAG)	yjfJ	b4182	G	G	G	conserved protein, PspA/IM30 family
SNP	4403951	T→C	C87R(TGC→CGC)	yjfM	b4185	C	C	C	conserved protein, DUF1190 family
SNP	4403995	T→G	D101E(GAT→GAG)	yjfM	b4185	G	G	G	conserved protein, DUF1190 family
SNP	4405529	T→G	intergenic (+32/-37)	yjfC/aidB	b4186/b4187	-	G	G	glutathionylspermidine synthase homolog/isovaleryl CoA dehydrogenase
SNP	4407065	T→C	G500G(GGT→GGC)	aidB	b4187	-	C	C	isovaleryl CoA dehydrogenase
SNP	4411418	A→G	T45A(ACG→GCG)	ulaA	b4193	G	G	G	L-ascorbate-specific enzyme IIC component of PTS
SNP	4416355	A→G	intergenic (-258/-69)	yjfY/rpsF	b4199/b4200	-	G	G	predicted protein/30S ribosomal subunit protein S6
SNP	4420387	A→G	K49K(AAA→AAG)	fklB	b4207	G	G	G	FKBP-type peptidyl-prolyl cis-trans isomerase (rotamase)
SNP	4421488	T→G	Y107D(TAC→GAC)	cycA	b4208	-	-	G	D-alanine/D-serine/glycine transporter
SNP	4421759	T→C	F197S(TTC→TCC)	cycA	b4208	C	C	C	D-alanine/D-serine/glycine transporter
SNP	4424102	T→C	Q87Q(CAA→CAG)	ytfF	b4210	C	C	C	inner membrane protein, DMT transporter family
SNP	4427772	T→G	S34R(AGC→CGC)	cpdB	b4213	-	-	G	2':3'-cyclic-nucleotide 2'-phosphodiesterase
SNP	4432461	A→G	S21P(TCC→CCC)	ytfL	b4218	-	G	G	inner membrane protein, UPF0053 family
SNP	4434615	A→G	T310A(ACC→GCC)	ytfM	b4220	-	-	G	predicted outer membrane protein and surface antigen
SNP	4438594	A→G	K1059K(AAA→AAG)	ytfN	b4221	G	G	G	large conserved protein, DUF490 family
SNP	4441438	A→C	K57N(AAA→AAC)	ytfQ	b4227	C	C	C	galactofuranose binding proteint: periplasmic- binding component of ABC superfamily
SNP	4443901	A→G	T8A(ACC→GCC)	ytfT	b4230	G	G	G	predicted sugar transporter subunit: membrane component of ABC superfamily
SNP	4445049	T→G	F46C(TTT→TGT)	yjfF	b4231	-	-	G	predicted sugar transporter subunit: membrane component of ABC superfamily
SNP	4449929	T→G	V222G(GTT→GGT)	pmbA	b4235	G	G	G	predicted peptidase required for the maturation and secretion of the antibiotic peptide MccB17
SNP	4451363	A→G	V103A(GTG→GCG)	nrdG	b4237	-	G	G	anaerobic ribonucleotide reductase activating protein
SNP	4459122	A→C	L64F(TTA→TTC)	mgtA	b4242	-	C	C	magnesium transporter

SNP	4460510	A→G	Y527C(TAC→TGC)	mgtA	b4242	G	G	G	magnesium transporter
SNP	4465606	T→C	S59S(AGT→AGC)	yjgJ	b4251	-	C	C	predicted transcriptional regulator
SNP	4466180	A→C	N5H(AAT→CAT)	tabA	b4252	-	-	C	biofilm modulator regulated by toxins
SNP	4466471	T→G	F102V(TTT→GTT)	tabA	b4252	G	G	G	biofilm modulator regulated by toxins
SNP	4466632	C→T	intergenic (+12/-111)	tabA/yjgL	b4252/b4253	T	T	T	biofilm modulator regulated by toxins/predicted protein
SNP	4467331	A→C	K197Q(AAA→CAA)	yjgL	b4253	C	C	C	predicted protein
SNP	4468167	T→G	F475L(TTT→TTG)	yjgL	b4253	G	G	G	predicted protein
SNP	4470536	C→T	G103E(GGG→GAG)	yjgM	b4256	T	T	T	predicted acetyltransferase
INS	4471270	+T	pseudogene (262/1223 nt)	yjgN	b4257	+T	+T	+T	inner membrane protein, DUF898 family
SNP	4473728	A→G	V472A(GTT→GCT)	valS	b4258	G	G	G	valyl-tRNA synthetase
SNP	4474369	A→G	R258R(CGT→CGC)	valS	b4258	G	G	G	valyl-tRNA synthetase
SNP	4475570	A→G	F6L(TTC→CTC)	holC	b4259	G	G	G	DNA polymerase III, chi subunit
SNP	4478534	T→G	Y280D(TAT→GAT)	lptF	b4261	G	G	G	lipopolysaccharide export ABC permease of the LptBFGC export complex
SNP	4487472	C→T	E15K(GAA→AAA)	yjgB	b4269	T	T	T	predicted alcohol dehydrogenase, Zn-dependent and NAD(P)-binding
SNP	4489280	A→C	intergenic (+35/-297)	intB/insC	b4271/b4272	C	C	C	pseudogene, integrase homology;IS, phage, Tn; Phage- related functions and prophages; KpLE2 phage-like element; P4-like integrase/IS2 repressor TnpA
SNP	4489560	T→C	intergenic (+315/-17)	intB/insC	b4271/b4272	-	-	C	pseudogene, integrase homology;IS, phage, Tn; Phage- related functions and prophages; KpLE2 phage-like element; P4-like integrase/IS2 repressor TnpA
SNP	4492642	A→C	R26S(AGA→AGC)	yjgZ	b4277	-	C	C	KpLE2 phage-like element; predicted protein
SNP	4494004	T→G	K245Q(AAA→CAA)	insG	b4278	G	G	G	IS4 transposase
SNP	4500526	T→C	pseudogene (2025/2597 nt)	insO	b4623	-	C	C	pseudogene, IS911 transposase B;IS, phage, Tn; Transposon-related functions; extrachromosomal; transposon related
SNP	4506427	A→G	V519A(GTG→GCG)	fecA	b4291	G	G	G	KpLE2 phage-like element; ferric citrate outer membrane transporter
SNP	4509156	A→G	F129L(TTT→CTT)	fecI	b4293	-	G	G	KpLE2 phage-like element; RNA polymerase, sigma 19 factor
SNP	4513257	A→C	N23K(AAT→AAG)	yjhF	b4296	C	C	C	KpLE2 phage-like element; predicted transporter
SNP	4524212	T→G	N93H(AAC→CAC)	yjhP	b4306	-	-	G	KpLE2 phage-like element; predicted methyltransferase
SNP	4525478	A→G	intergenic (-120/+371)	yjhX/yjhZ	b4566/b4657	-	-	G	conserved protein/pseudogene, rimK paralog, C-terminal fragment
SNP	4531018	A→C	intergenic (-212/-1244)	nanC/fimB	b4311/b4312	-	-	C	N-acetylnuraminic acid outer membrane channel protein/tyrosine recombinase/inversion of on/off regulator of fimA
SNP	4543752	G→A	C271Y(TGC→TAC)	uxuA	b4322	A	A	A	mannonate hydrolase
INS	4548065	+T	intergenic (-440/-232)	yjiC/iraD	b4325/b4326	+T	+T	+T	predicted protein/RpoS stabilzer after DNA damage, anti-RssB factor
SNP	4555513	T→G	T44P(ACA→CCA)	yjiL	b4334	G	G	G	predicted ATPase, activator of (R)- hydroxyglutaryl-CoA dehydratase
SNP	4568662	A→C	Y211D(TAT→GAT)	mcrC	b4345	C	C	C	5-methylcytosine-specific restriction enzyme McrBC, subunit McrC
SNP	4569079	A→C	Y72D(TAC→GAC)	mcrC	b4345	-	C	C	5-methylcytosine-specific restriction enzyme McrBC, subunit McrC
SNP	4572649	A→C	Y40D(TAT→GAT)	hsdS	b4348	-	-	C	specificity determinant for hsdM and hsdR
SNP	4573472	T→C	Q294R(CAG→CGG)	hsdM	b4349	C	C	C	DNA methyltransferase M
SNP	4576999	A→G	V356A(GTG→GCG)	hsdR	b4350	G	G	G	endonuclease R Type I restriction enzyme
SNP	4578853	G→A	G201S(GGC→AGC)	mrr	b4351	A	A	A	methylated adenine and cytosine restriction protein
SNP	4582270	A→G	V105A(GTG→GCG)	yjiY	b4354	G	G	G	predicted inner membrane protein
SNP	4588684	T→G	N474H(AAC→CAC)	opgB	b4359	-	G	G	phosphoglycerol transferases I and II
INS	4597390	+G	noncoding (80/87 nt)	leuV	b4368	+G	+G	+G	tRNA-Leu
INS	4597511	+G	noncoding (80/87 nt)	leuP	b4369	+G	+G	+G	tRNA-Leu
SNP	4602461	T→G	intergenic (+154/-239)	prfC/osmY	b4375/b4376	-	-	G	peptide chain release factor RF-3/periplasmic protein
SNP	4603009	A→C	K104Q(AAA→CAA)	osmY	b4376	-	C	C	periplasmic protein
SNP	4605501	A→G	E171E(GAA→GAG)	yjjV	b4378	G	G	G	predicted DNase
SNP	4606045	A→G	L268S(TTG→TCG)	yjjW	b4379	G	G	G	predicted pyruvate formate lyase activating enzyme
SNP	4606657	A→C	I64S(ATT→AGT)	yjjW	b4379	-	C	C	predicted pyruvate formate lyase activating enzyme
SNP	4606903	G→A	G489G(GGC→GGT)	yjjI	b4380	A	A	A	conserved protein
SNP	4608645	A→C	S7R(AGC→CGC)	deoC	b4381	-	C	C	2-deoxyribose-5-phosphate aldolase, NAD(P)- linked
SNP	4609744	A→C	D28A(GAC→GCC)	deoA	b4382	C	C	C	thymidine phosphorylase
SNP	4611163	A→C	Y86S(TAC→TCC)	deoB	b4383	-	-	C	phosphopentomutase
SNP	4611935	C→T	I343I(ATC→ATT)	deoB	b4383	T	T	T	phosphopentomutase
SNP	4624699	A→C	G117G(GGT→GGG)	yjjX	b4394	-	-	C	inosine/xanthosine triphosphatase
SNP	4629068	A→G	T357A(ACC→GCC)	creC	b4399	G	G	G	sensory histidine kinase in two-component regulatory system with CreB or PhoB, regulator of the CreBC regulon
SNP	4632851	A→G	Q202Q(CAA→CAG)	yjtD	b4403	G	G	G	predicted rRNA methyltransferase
