# Supplementary material for: Chance and necessity in the genome evolution of endosymbiotic bacteria of insects
Source: ISME J. 2017 Mar 21;11(6):1291–304. doi: 10.1038/ismej.2017.18 (PMC5437351; doi:10.1038/ismej.2017.18)
Supplement: Supplementary Table S4 [file ismej201718x4.pdf]

Table S4. Mutational spectrum of experimentally evolving clonal bacteria.

Line	Coding Regions				Non-coding regions		
	NSNPs[a]	SSNPs[b]	Insertions (bp)	Deletions (bp)	SNPs	Insertions (bp)	Deletions (bp)
A	339	180	52 (62)	26 (38340)	70	31 (35)	15 (15)
B	752	228	33 (36)	46 (3085)	139	23 (24)	16 (21)

[a]Non-Synonymous Single Nucleotide Polymorphisms

[b]Synonymous Single Nucleotide Polymorphisms
