# Supplementary material for: Chance and necessity in the genome evolution of endosymbiotic bacteria of insects
Source: ISME J. 2017 Mar 21;11(6):1291–304. doi: 10.1038/ismej.2017.18 (PMC5437351; doi:10.1038/ismej.2017.18)
Supplement: Supplementary Table S5 [file ismej201718x5.pdf]

Table S5. GO categories corresponding to Molecular Function enriched for mutated Line B of the evolution experiment of Escherichia coli.

GO Term	# genes	#Observed	#Expected	Fold Enrichment
Receptor Activity	50	22	7.57	2.91
Anion Binding	687	145	104.02	1.39
Small Molecule Binding	740	153	112.04	1.37
Catalytic Activity	2005	373	303	1.23

genes in

P-value
1.04E-02
2.47E-02
2.16E-02
2.24E-05
