# Supplementary material for: Chance and necessity in the genome evolution of endosymbiotic bacteria of insects
Source: ISME J. 2017 Mar 21;11(6):1291–304. doi: 10.1038/ismej.2017.18 (PMC5437351; doi:10.1038/ismej.2017.18)
Supplement: Supplementary Table S6 [file ismej201718x6.pdf]

Table S6. Polymorphisms and fixed mutations in an *E. coli* evolving population under mild genetic drift effect

Genome position	Mutation	%individuals	Kind	Gene	Description
34,112	Δ1 bp	6.40%	intergenic (+74/−188)	*carB* → /	carbamoyl−phosphate synthase large subunit/DNA−binding transcriptional activator
45,078	C→T	100%	A300V (GCC→GTC)	*fixC* →	predicted oxidoreductase with FAD/NAD(P)−binding domain
83,967	C→T	100%	S61L (TCA→TTA)	*cra* →	DNA−binding transcriptional repressor−activator for carbon metabolism
94,792	C→T	5.10%	A249V (GCA→GTA)	*ftsW* →	Lipid II flippase; integral membrane protein involved in stabilizing FstZ ring during cell division
103,188	+T	100%	intergenic (+70/−229)	*lpxC* → /	UDP−3−O−acyl N−acetylglucosamine deacetylase/regulator of secA translation
115,381	+G	2.60%	coding (458/855 nt)	*ampE* →	predicted inner membrane protein
125,879	A→G	4.10%	V342A (GTC→GCC)	*yacH* ←	predicted protein
134,998	T→C	5.60%	N624S (AAC→AGC)	*gcd* ←	glucose dehydrogenase
143,452	A→G	7.90%	D281G (GAC→GGC)	*yadD* →	predicted transposase
162,606	G→A	2.60%	R745H (CGT→CAT)	*mrcB* →	fused glycosyl transferase and transpeptidase
221,872	A→G	3.70%	noncoding (471/2904 nt)	*rrlH* →	23S ribosomal RNA of rrnH operon
256,437	Δ1 bp	4.60%	coding (68/1254 nt)	*proA* →	gamma−glutamylphosphate reductase
263,342	G→A	2.10%	S162S (AGC→AGT)	*ykfA* ←	CP4−6 prophage; predicted GTP−binding protein
302,914	+C	100%	coding (1286/2526 nt)	*yagX* ←	predicted aromatic compound dioxygenase
305,276	G→A	100%	S88L (TCA→TTA)	*matB* ←	cryptic Mat fimbrillin gene
332,612	C→T	66.00%	T323M (ACG→ATG)	*yahF* →	predicted acyl−CoA synthetase with NAD(P)− binding domain and succinyl−CoA synthetase domain
354,567	Δ1 bp	4.70%	coding (212/471 nt)	*cynS* →	cyanate aminohydrolase
374,880	Δ1 bp	46.00%	intergenic (−132/+56)	*frmR* ← /	regulator protein that represses frmRAB operon/outer membrane protein
374,881	Δ1 bp	5.80%	intergenic (−133/+55)	*frmR* ← /	regulator protein that represses frmRAB operon/outer membrane protein
382,904	G→A	3.20%	E81E (GAG→GAA)	*tauD* →	taurine dioxygenase, 2−oxoglutarate−dependent
421,890	G→A	2.30%	G296D (GGC→GAC)	*tgt* →	tRNA−guanine transglycosylase
428,446	A→G	6.20%	Y36C (TAC→TGC)	*ribD* →	fused diaminohydroxyphosphoribosylaminopyrimidine deaminase and 5−amino−6−(5−phosphoribosylamino) uracil reductase
441,376	G→A	4.70%	A53V (GCC→GTC)	*yajR* ←	predicted transporter
446,939	A→G	14.30%	*492Q (TAA→CAA)	*ampG* ←	muropeptide transporter
459,067	C→T	46.30%	V88V (GTC→GTT)	*ybaV* →	conserved protein, ComEA homolog
464,431	Δ1 bp	2.90%	coding (607/1686 nt)	*mdlA* →	fused predicted multidrug transporter subunits of ABC superfamily: ATP−binding components
500,867	A→G	21.90%	K363E (AAA→GAA)	*ushA* →	bifunctional UDP−sugar hydrolase/5'− nucleotidase
518,073	+T	100%	intergenic (+376/−54)	*ybbP* → /	predicted ABC transporter permease/rhsD element protein
532,746	C→T	10.40%	A83V (GCG→GTG)	*ybbW* →	predicted allantoin transporter
538,167	T→C	2.00%	Y249C (TAT→TGT)	*ylbA* ←	conserved protein
539,430	A→G	100%	S243S (AGT→AGC)	*allC* ←	allantoate amidohydrolase
539,932	A→G	100%	V76A (GTT→GCT)	*allC* ←	allantoate amidohydrolase
556,086	Δ1 bp	2.30%	coding (1524/2604 nt)	*sfmD* →	predicted outer membrane export usher protein
566,913	T→C	100%	R198R (CGT→CGC)	*ybcM* →	DLP12 prophage; predicted DNA−binding transcriptional regulator
566,924	+G	21.10%	coding (605/798 nt)	*ybcM* →	DLP12 prophage; predicted DNA−binding transcriptional regulator
624,845	G→A	1.70%	G29E (GGG→GAG)	*cstA* →	carbon starvation protein
652,106	A→G	5.50%	intergenic (+123/−52)	*pagP* → /	phospholipid:lipid A palmitoyltransferase/DNA−binding transcriptional repressor
655,155	Δ1 bp	2.50%	intergenic (−73/+136)	*lipA* ← /	lipoate synthase/predicted DNA−binding transcriptional regulator
686,553	Δ1 bp	2.50%	coding (98/879 nt)	*ybeX* ←	predicteed ion transport
692,983	A→G	2.10%	V354A (GTG→GCG)	*asnB* ←	asparagine synthetase B
697,752	A→G	5.10%	P242P (CCT→CCC)	*nagB* ←	glucosamine−6−phosphate deaminase
704,595	A→G	100%	T466A (ACC→GCC)	*chiP* →	chitoporin, uptake of chitosugars
710,510	C→T	4.80%	S78L (TCA→TTA)	*ybfP* →	lipoprotein
730,003	A→G	14.00%	pseudogene (918/1434 nt	*ybfO* →	pseudogene, rhs−like
736,011	Δ1 bp	2.20%	coding (1412/1482 nt)	*dtpD* ←	dipeptide and tripeptide permease D
825,134	A→G	2.40%	F182L (TTC→CTC)	*ybhS* ←	predicted transporter subunit: membrane component of ABC superfamily
873,149	C→T	2.70%	T582T (ACC→ACT)	*yliE* →	predicted cyclic−di−GMP phosphodiesterase, inner membrane protein
909,164	G→A	5.60%	S104F (TCC→TTC)	*poxB* ←	pyruvate dehydrogenase (pyruvate oxidase), thiamin−dependent, FAD−binding
919,281	A→G	100%	E170E (GAA→GAG)	*macB* →	fused macrolide transporter subunits of ABC superfamily: ATP−binding component/membrane component
962,053	A→G	6.30%	N545S (AAC→AGC)	*rpsA* →	30S ribosomal subunit protein S1
975,763	A→G	2.80%	N338S (AAC→AGC)	*mukB* →	chromosome condensin MukBEF, ATPase and DNA− binding subunit
980,270	Δ1 bp	100%	coding (799/1848 nt)	*ycbB* →	murein L,D−transpeptidase
983,826	+C	3.40%	coding (309/1191 nt)	*aspC* ←	aspartate aminotransferase, PLP−dependent
985,426	Δ1 bp	5.30%	intergenic (−19/+584)	*ompF* ←	outer membrane porin 1a (Ia;b;F)/asparaginyl tRNA synthetase
1,000,642	+G	19.40%	coding (411/1071 nt)	*elfG* →	predicted fimbrial−like adhesin protein
1,002,578	+G	7.30%	coding (207/711 nt)	*ycbF* →	predicted periplasmic pilini chaperone
1,007,074	A→G	3.00%	Q269R (CAA→CGA)	*rlmL* →	23S rRNA mG2445 methyltransferase, SAM− dependent
1,017,394	Δ1 bp	4.20%	intergenic (+32/+44)	*matP* → /	Ter macrodomain organizer matS−binding protein/outer membrane protein A (3a;II*;G;d)
1,027,406	T→C	13.00%	N330S (AAT→AGT)	*rlmI* ←	23S rRNA m(5)C1962 methyltransferase, SAM− dependent
1,064,599	+A	4.00%	coding (590/1242 nt)	*agp* →	glucose−1−phosphatase/inositol phosphatase
1,064,599	+G	4.00%	coding (590/1242 nt)	*agp* →	glucose−1−phosphatase/inositol phosphatase
1,080,375	C→T	4.80%	pseudogene (392/627 nt)	*efeU* →	ferrous iron permease (pseudogene)
1,087,096	T→G	2.50%	N396T (AAC→ACC)	*pgaB* ←	biofilm adhesin polysaccharide PGA export lipoprotein with a polysaccharide deacetylase activity needed for export
1,087,350	Δ1 bp	100%	coding (933/2019 nt)	*pgaB* ←	biofilm adhesin polysaccharide PGA export lipoprotein with a polysaccharide deacetylase activity needed for export
1,103,366	Δ1 bp	1.70%	intergenic (+39/−20)	*csgA* → /	curlin subunit, amyloid curli fibers, cryptic/curli assembly protein
1,104,841	Δ1 bp	2.60%	coding (122/1482 nt)	*ymdC* →	predicted hydrolase
1,124,216	A→G	100%	Y77C (TAC→TGC)	*rimJ* →	ribosomal−protein−S5−alanine N− acetyltransferase
1,146,270	T→C	9.30%	P65P (CCT→CCC)	*plsX* →	probable phosphate acyltransferase
1,166,483	Δ1 bp	1.80%	coding (448/528 nt)	*ycfJ* →	predicted protein
1,167,205	A→G	3.90%	V18A (GTG→GCG)	*ycfQ* ←	repressor for bhsA(ycfR)
1,172,430	Δ1 bp	100%	intergenic (−41/+87)	*mfd* ← /	transcription−repair coupling factor/inner membrane protein
1,189,968	A→G	2.80%	R148R (CGT→CGC)	*purB* ←	adenylosuccinate lyase
1,203,259	Δ1 bp	4.00%	pseudogene (665/1368 nt	*oweE* →	pseudogene, lambda replication protein O homolog; CPS−53/KpLE1 prophage;Phage or Prophage Related
1,210,277	A→C	14.70%	I51S (ATC→AGC)	*iraM* ←	RpoS stabilzer during Mg starvation, anti−RssB factor
1,214,628	G→A	100%	D46N (GAT→AAT)	*ymgA* →	connector protein for RcsB regulation of biofilm
1,249,884	G→A	3.90%	A132T (GCC→ACC)	*dhaR* →	DNA−binding transcription activator of the dhaKLM operon
1,252,302	A→G	2.40%	G692G (GGT→GGC)	*ycgV* ←	predicted adhesin
1,252,993	+C	2.80%	coding (1385/2868 nt)	*ycgV* ←	predicted adhesin
1,254,337	C→T	1.90%	R14H (CGT→CAT)	*ycgV* ←	predicted adhesin
1,267,766	Δ1 bp	17.80%	intergenic (−66/+362)	*ldrA* ← /	toxic polypeptide, small/toxic polypeptide, small
1,274,249	A→G	18.60%	S2S (AGT→AGC)	*narL* ←	DNA−binding response regulator in two−component regulatory system with NarX (or NarQ)
1,294,680	C→T	30.70%	A623T (GCA→ACA)	*adhE* ←	fused acetaldehyde−CoA dehydrogenase/iron− dependent alcohol dehydrogenase/pyruvate−formate lyase deactivase
1,294,800	A→G	10.10%	F583L (TTC→CTC)	*adhE* ←	fused acetaldehyde−CoA dehydrogenase/iron− dependent alcohol dehydrogenase/pyruvate−formate lyase deactivase
1,295,249	A→G	18.00%	V433A (GTT→GCT)	*adhE* ←	fused acetaldehyde−CoA dehydrogenase/iron− dependent alcohol dehydrogenase/pyruvate−formate lyase deactivase
1,295,433	A→G	45.50%	Y372H (TAC→CAC)	*adhE* ←	fused acetaldehyde−CoA dehydrogenase/iron− dependent alcohol dehydrogenase/pyruvate−formate lyase deactivase
1,295,898	T→C	2.40%	T217A (ACT→GCT)	*adhE* ←	fused acetaldehyde−CoA dehydrogenase/iron− dependent alcohol dehydrogenase/pyruvate−formate lyase deactivase
1,297,920	IS*5* (+) +4 bp	100%	intergenic (+250/−485)	*ychE* → /	predicted inner membrane protein/oligopeptide transporter subunit
1,308,563	A→G	7.30%	K83K (AAA→AAG)	*tonB* →	membrane spanning protein in TonB−ExbB−ExbD transport complex
1,333,625	A→T	2.60%	K190I (AAA→ATA)	*acnA* →	aconitate hydratase 1
1,336,353	Δ1 bp	2.60%	coding (34/591 nt)	*ribA* ←	GTP cyclohydrolase II
1,336,354	Δ1 bp	2.60%	coding (33/591 nt)	*ribA* ←	GTP cyclohydrolase II
1,348,415	+G	5.50%	intergenic (−150/+218)	*fabI* ← /	enoyl−[acyl−carrier−protein] reductase, NADH− dependent/conserved protein
1,352,793	G→A	3.50%	A515V (GCC→GTC)	*sapA* ←	antimicrobial peptide transport ABC transporter periplasmic binding protein
1,353,750	G→A	3.80%	A196V (GCC→GTC)	*sapA* ←	antimicrobial peptide transport ABC transporter periplasmic binding protein
1,367,261	+T	3.00%	intergenic (+32/−181)	*pspE* → /	thiosulfate:cyanide sulfurtransferase (rhodanese)/predicted glucosyltransferase
1,367,262	Δ1 bp	5.10%	intergenic (+33/−180)	*pspE* → /	thiosulfate:cyanide sulfurtransferase (rhodanese)/predicted glucosyltransferase
1,377,956	A→G	100%	T195A (ACC→GCC)	*ycjU* →	beta−phosphoglucomutase
1,388,677	C→T	12.90%	A135T (GCC→ACC)	*ycjY* ←	predicted hydrolase
1,392,081	G→T	16.20%	intergenic (+15/+36)	*mppA* →	murein tripeptide (L−ala−gamma−D−glutamyl−meso− DAP) transporter subunit/MscS family inner membrane protein
1,396,692	G→A	4.30%	Q21* (CAG→TAG)	*fnr* ←	DNA−binding transcriptional dual regulator, global regulator of anaerobic growth
1,397,205	Δ1 bp	2.90%	coding (258/516 nt)	*ogt* ←	O−6−alkylguanine−DNA:cysteine−protein methyltransferase
1,400,464	A→G	100%	R6R (CGT→CGC)	*abgB* ←	p−aminobenzoyl−glutamate hydrolase, B subunit
1,418,869	+C	6.50%	coding (525/858 nt)	*ydaU* →	Rac prophage; conserved protein
1,428,060	A→G	4.50%	T596A (ACT→GCT)	*stfR* →	Rac prophage; predicted tail fiber protein
1,429,120	Δ1 bp	2.70%	coding (2846/3363 nt)	*stfR* →	Rac prophage; predicted tail fiber protein
1,432,066	Δ1 bp	5.30%	intergenic (−616/+115)	*ynaE* ← /	cold shock protein, function unknown, Rac prophage/pseudogene, prophage Rac integration site ttcA duplication;Phage or Prophage Related
1,443,974	A→G	2.40%	L179P (CTG→CCG)	*feaR* ←	DNA−binding transcriptional activator for tynA and feaB
1,472,335	+C	3.30%	intergenic (+28/−35)	*ydbC* → /	predicted oxidoreductase, NAD(P)−binding/predicted PF10971 family periplasmic methylglyoxal resistance protein
1,483,712	T→C	2.40%	R1142R (CGT→CGC)	*hrpA* →	predicted ATP−dependent helicase
1,517,957	A→G	100%	Q157R (CAA→CGA)	*mcbR* →	colanic acid and biofilm gene transcriptional regulator, MqsR−controlled
1,521,372	C→T	3.20%	N280N (AAC→AAT)	*yncE* →	ATP−binding protein, periplasmic, function unknown
1,537,360	T→C	100%	S819S (TCA→TCG)	*narZ* ←	nitrate reductase 2 (NRZ), alpha subunit
1,546,080	A→G	100%	N289S (AAC→AGC)	*fdnG* →	formate dehydrogenase−N, alpha subunit, nitrate− inducible
1,584,892	Δ1 bp	4.00%	coding (69/915 nt)	*ydeQ* ←	predicted fimbrial−like adhesin protein
1,588,542	C→T	10.10%	E287E (GAG→GAA)	*hipA* ←	serine protein kinase required for perister formation; toxin of HipAB toxin−antitoxin system
1,591,350	+C	10.30%	pseudogene (3963/3975 r	*yneO* ←	pseudogene, AidA homolog
1,591,351	Δ1 bp	4.30%	pseudogene (3962/3975 r	*yneO* ←	pseudogene, AidA homolog
1,591,474	+C	8.30%	pseudogene (3839/3975 r	*yneO* ←	pseudogene, AidA homolog
1,594,532	+C	5.00%	pseudogene (781/3975 nt	*yneO* ←	pseudogene, AidA homolog
1,598,677	Δ1 bp	3.30%	intergenic (−210/−39)	*lsrR* ← /	lsr operon transcriptional repressor/Autoinducer 2 import ATP−binding protein
1,684,382	A→G	2.50%	G407G (GGT→GGC)	*fumA* ←	fumarate hydratase (fumarase A), aerobic Class I
1,711,543	Δ1 bp	100%	intergenic (+47/−59)	*dtpA* → /	dipeptide and tripeptide permease A/glutathionine S−transferase
1,731,522	+T	3.80%	intergenic (−196/−207)	*grxD* ← /	glutaredoxin−4/predicted peptidase, C40 clan
1,736,700	C→T	7.10%	R108R (CGG→CGA)	*ydhB* ←	predicted DNA−binding transcriptional regulator
1,766,697	Δ1 bp	2.80%	coding (399/1113 nt)	*ydiK* →	inner membrane protein, UPF0118 family
1,775,491	T→C	63.80%	C357C (TGT→TGC)	*ydiO* →	predicted acyl−CoA dehydrogenase
1,796,172	T→C	11.40%	intergenic (−5/+279)	*pheS* ← /	phenylalanine tRNA synthetase, alpha subunit/phenylalanyl−tRNA synthetase operon leader peptide
1,805,182	Δ1 bp	3.80%	coding (162/861 nt)	*yniA* →	predicted phosphotransferase/kinase
1,815,768	+C	5.50%	intergenic (−43/+62)	*chbF* ← /	phospho−chitobiase; general 6−phospho−beta− glucosidase activity/rRepressor, chb operon for N,N'− diacetylchitobiose utilization

1,832,193	G→A	25.60%	R54H (CGC→CAC)	*ydjZ* →	Inner membrane protein, TVP38/TMEM64 family
1,854,456	+C	100%	coding (539/837 nt)	*ydjI* ←	predicted aldolase
1,866,170	+G	5.00%	intergenic (+103/-10)	*yeaG* → /	protein kinase, function unknown; autokinase/conserved protein
1,899,185	C→T	3.50%	intergenic (-375/-154)	*yoaE* ← /	fused predicted membrane protein/conserved protein/fused mannose-specific PTS enzymes: IIA component/IIB component
1,911,715	G→A	42.60%	L109L (CTC→CTT)	*prc* ←	carboxy-terminal protease for penicillin-binding protein 3
1,922,176	A→G	23.20%	S7P (TCC→CCC)	*yobA* ←	conserved protein
1,933,749	+A	100%	intergenic (-210/-127)	*zwf* ← / –	glucose-6-phosphate 1-dehydrogenase/predicted DNA-binding transcriptional regulator
1,942,586	A→G	7.60%	intergenic (-6/+3)	*ruvB* ← /	ATP-dependent DNA helicase, component of RuvABC resolvasome/component of RuvABC resolvasome, regulatory subunit
1,946,737	Δ1 bp	2.90%	coding (1010/1773 nt)	*aspS* ←	aspartyl-tRNA synthetase
1,946,738	Δ1 bp	2.90%	coding (1009/1773 nt)	*aspS* ←	aspartyl-tRNA synthetase
1,960,440	Δ1 bp	41.50%	coding (1835/2079 nt)	*flhA* ←	predicted flagellar export pore protein
1,963,408	T→C	4.40%	intergenic (-167/+209)	*flhB* ← /	flagellin export apparatus, substrate specificity protein/chemotaxis regulator, protein phosphatase for CheY
1,974,753	A→G	3.90%	C106R (TGC→CGC)	*flhC* ←	DNA-binding transcriptional dual regulator with FlhD
1,975,658	+G	9.50%	intergenic (-237/+84)	*flhD* ← /	DNA-binding transcriptional dual regulator with FlhC/IS1 transposase B
2,046,754	A→G	16.50%	K1531K (AAA→AAG)	*yeeJ* →	probable adhesin
2,055,782	T→C	24.10%	Y378C (TAC→TGC)	*yeeO* ←	predicted multdrug exporter, MATE family
2,062,965	+C	2.30%	coding (24/546 nt)	*cobU* ←	bifunctional cobinamide kinase/ cobinamide phosphate guanylyltransferase
2,067,482	+G	5.00%	intergenic (-78/-12)	*insC* ← /	IS2 repressor TnpA/CP4-44 prophage; predicted disrupted hemin or colicin receptor;Phage or Prophage Related; interrupted by IS2 and C-terminal deletion
2,105,881	A→G	3.10%	L310L (TTG→CTG)	*rfbX* ←	predicted polisoprenol-linked O-antigen transporter
2,106,695	A→G	100%	G38G (GGT→GGC)	*rfbX* ←	predicted polisoprenol-linked O-antigen transporter
2,186,176	+C	3.50%	coding (1959/2481 nt)	*yehB* ←	predicted outer membrane protein
2,196,354	G→T	2.80%	pseudogene (1832/2022 r	*yehH* →	pseudogene; molybdate metabolism regulator, first fragment; molybdate metabolism regulator, second fragment 2
2,197,907	+G	3.40%	coding (405/3633 nt)	*yehI* →	conserved protein
2,197,907	+A	3.40%	coding (405/3633 nt)	*yehI* →	conserved protein
2,223,832	C→T	4.10%	V221V (GTG→GTA)	*yohF* ←	predicted oxidoreductase with NAD(P)-binding Rossmann-fold domain
2,230,704	Δ1 bp	3.40%	coding (603/720 nt)	*sanA* →	vancomycin high temperature exclusion protein; mutants have a defective envelope more permeable to vancomycin at 42 degrees
2,236,692	Δ1 bp	2.90%	coding (881/999 nt)	*mglB* ←	methyl-galactoside transporter subunit
2,274,969	C→T	2.60%	I482I (ATC→ATT)	*yejF* →	microcin C transporter, ATP-binding subunit; ABC family
2,293,254	G→A	41.80%	A97A (GCC→GCT)	*ccmC* ←	heme exporter subunit
2,306,078	T→A	3.10%	D163V (GAT→GTT)	*alkB* ←	oxidative demethylase of N1-methyladenine or N3- methylcytosine DNA lesions
2,351,275	A→G	8.00%	T469A (ACC→GCC)	*glpA* →	sn-glycerol-3-phosphate dehydrogenase (anaerobic), large subunit, FAD/NAD(P)-binding
2,368,640	+C	3.20%	coding (509/1653 nt)	*arnT* →	4-amino-4-deoxy-L-arabinose transferase
2,368,640	+A	3.20%	coding (509/1653 nt)	*arnT* →	4-amino-4-deoxy-L-arabinose transferase
2,418,153	G→A	3.00%	W103* (TGG→TGA)	*yfcG* →	GSH-dependent disulfide bond oxidoreductase
2,460,104	+C	11.30%	intergenic (+234/-132)	*fadL* → /	long-chain fatty acid outer membrane transporter/predicted protein
2,479,121	Δ1 bp	2.60%	coding (280/1539 nt)	*emrY* ←	predicted multidrug efflux system
2,480,693	+A	3.40%	intergenic (-238/-286)	*emrK* ← /	EmrKY-TolC multidrug resistance efflux pump, membrane fusion protein component/DNA-binding response regulator in two-component regulatory system with EvgS
2,514,988	T→C	4.90%	V23V (GTA→GTG)	*yfeA* ←	predicted diguanylate cyclase
2,561,005	Δ1 bp	2.50%	coding (190/378 nt)	*yffQ* →	CPZ-55 prophage; predicted protein
2,561,006	Δ1 bp	2.60%	coding (191/378 nt)	*yffQ* →	CPZ-55 prophage; predicted protein
2,561,014	C→T	2.40%	P67S (CCG→TCG)	*yffQ* →	CPZ-55 prophage; predicted protein
2,598,024	A→G	10.10%	E108G (GAA→GGA)	*bcp* →	thiol peroxidase, thioredoxin-dependent
2,612,192	A→G	31.20%	V305A (GTT→GCT)	*yfgO* ←	predicted inner membrane permease, UPF0118 family
2,625,155	A→G	7.40%	V336V (GTT→GTC)	*yfgF* ←	cyclic-di-GMP phosphodiesterase, anaerobic
2,650,487	C→T	39.10%	P257L (CCA→CTA)	*sseA* →	3-mercaptopyruvate sulfurtransferase
2,661,742	T→C	100%	G43G (GGT→GGC)	*yfhR* →	S9 peptidase family protein, function unknown
2,661,979	G→T	2.80%	T122T (ACG→ACT)	*yfhR* →	S9 peptidase family protein, function unknown
2,662,022	Δ1 bp	4.00%	coding (409/855 nt)	*yfhR* →	S9 peptidase family protein, function unknown
2,677,863	T→C	6.10%	S703G (AGC→GGC) ‡	*yphG* ←	conserved protein
2,677,864	G→A	6.10%	A702A (GCC→GCT) ‡	*yphG* ←	conserved protein
2,712,880	G→A	7.80%	A61A (GCG→GCA)	*eamB* →	cysteine and O-acetylserine exporter
2,731,759	+C	14.30%	coding (500/732 nt)	*yfiH* ←	conserved protein, UPF0124 family
2,734,342	IS*1* (+) +8 bp	100%	intergenic (+235/-29)	*bamD* →	lipoprotein required for OM biogenesis, in BamABCDE complex/cold shock protein associated with 30S ribosomal subunit
2,737,330	+G	2.60%	coding (1045/1071 nt)	*aroF* ←	3-deoxy-D-arabino-heptulosonate-7-phosphate synthase, tyrosine-repressible
2,756,552	Δ1 bp	5.50%	coding (344/1410 nt)	*yfjI* →	CP4-57 prophage; predicted protein
2,758,567	T→C	14.20%	intergenic (+170/+8)	*yfjJ* → / ←	CP4-57 prophage; predicted protein/CP4-57 prophage; conserved protein
2,768,588	G→A	15.80%	G73R (GGG→AGG)	*yfjT* →	CP4-57 prophage; predicted protein
2,774,389	Δ1 bp	2.10%	coding (51/318 nt)	*yfjZ* →	CP4-57 prophage; antitoxin of the YpjF-YfjZ toxin-antitoxin system
2,807,977	A→G	3.00%	intergenic (+74/-17)	*ygaH* → /	probable L-valine exporter, norvaline resistance/DNA-binding transcriptional repressor of microcin B17 synthesis and multidrug efflux
2,815,563	Δ1 bp	17.00%	intergenic (-65/+134)	*argY* ← /	tRNA-Arg/tRNA-Arg
2,861,341	A→G	5.40%	L297P (CTC→CCC)	*rpoS* ←	RNA polymerase, sigma S (sigma 38) factor
2,867,739	G→A	20.20%	T93I (ACA→ATA)	*ygbE* ←	conserved inner membrane protein
2,872,618	+C	12.90%	intergenic (+321/+630)	*iap* → / ←	aminopeptidase in alkaline phosphatase isozyme conversion/probable ssRNA endonuclease, CRISP-associated protein
2,878,908	G→A	4.00%	intergenic (-91/+324)	*casA* ← /	CRISP RNA (crRNA) containing Cascade antiviral complex protein/Cas3 predicted helicase needed for Cascade anti- viral activity
2,881,844	T→C	19.70%	T19A (ACG→GCG)	*ygcB* ←	Cas3 predicted helicase needed for Cascade anti- viral activity
2,908,616	Δ1 bp	3.00%	coding (1064/1302 nt)	*rlmC* ←	23S rRNA m(5)U1939 methyltransferase, SAM- dependent
2,908,750	A→G	7.70%	S310S (AGT→AGC)	*rlmC* ←	23S rRNA m(5)U1939 methyltransferase, SAM- dependent
2,941,247	Δ1 bp	2.60%	coding (611/1098 nt)	*mltA* ←	membrane-bound lytic murein transglycosylase A
2,984,222	T→C	33.80%	S50S (AGT→AGC)	*yqeJ* →	predicted protein
2,998,152	+G	15.40%	coding (860/879 nt)	*xdhB* →	xanthine dehydrogenase, FAD-binding subunit
2,999,210	+G	2.30%	coding (1256/1779 nt)	*ygeV* ←	predicted DNA-binding transcriptional regulator
2,999,700	T→C	2.60%	T256A (ACC→GCC)	*ygeV* ←	predicted DNA-binding transcriptional regulator
3,004,979	T→C	100%	I91I (ATT→ATC)	*hyuA* →	D-stereospecific phenylhydantoinase
3,022,599	T→C	3.80%	V267A (GTA→GCA)	*ygfQ* →	predicted purine permease
3,025,014	A→T	3.20%	D199E (GAT→GAA)	*ygfT* ←	fused predicted oxidoreductase: Fe-S subunit/nucleotide-binding subunit
3,042,722	G→A	3.60%	N333N (AAC→AAT)	*gcvP* ←	glycine decarboxylase, PLP-dependent, subunit (protein P) of glycine cleavage complex
3,095,711	+C	5.10%	coding (592/720 nt)	*yggN* ←	predicted protein
3,104,125	A→G	62.30%	intergenic (-291/-206)	*speC* ← /	ornithine decarboxylase, constitutive/predicted inner membrane protein, DUF554 family
3,114,771	A→G	10.90%	G396G (GGT→GGC)	*glcA* ←	glycolate transporter
3,120,093	Δ1 bp	5.50%	coding (46/1224 nt)	*glcF* ←	glycolate oxidase 4Fe-4S iron-sulfur cluster subunit
3,120,094	Δ1 bp	5.50%	coding (45/1224 nt)	*glcF* ←	glycolate oxidase 4Fe-4S iron-sulfur cluster subunit
3,123,962	G→A	4.50%	pseudogene (933/1173 nt	*yghO* ←	pseudogene, DNA-binding transcriptional regulator homology
3,136,536	C→T	4.40%	G378E (GGG→GAG)	*hybC* ←	hydrogenase 2, large subunit
3,153,678	Δ1 bp	6.00%	coding (2148/2220 nt)	*ygiQ* ←	conserved protein
3,159,661	A→G	3.40%	M331T (ATG→ACG)	*parC* ←	DNA topoisomerase IV, subunit A
3,183,493	A→G	5.70%	pseudogene (1393/2445 r	*yqiG* →	pseudogene; outer membrane usher homology;putative membrane; Not classified; putative membrane protein
3,186,115	+A	2.70%	coding (804/1065 nt)	*yqiI* →	conserved protein
3,188,759	G→A	8.00%	A406T (GCC→ACC)	*yqiK* →	PHB family membrane protein, function unknown
3,192,418	C→G	3.80%	G635A (GGT→GCT)	*glnE* ←	fused deadenylyltransferase/adenylyltransferase for glutamine synthetase
3,209,438	A→G	45.30%	Y571C (TAC→TGC)	*rpoD* →	RNA polymerase, sigma 70 (sigma D) factor
3,212,940	T→C	7.50%	T273A (ACG→GCG)	*aer* ←	fused signal transducer for aerotaxis sensory component/methyl accepting chemotaxis component
3,216,198	T→C	4.30%	V18A (GTA→GCA)	*ebgR* →	DNA-binding transcriptional repressor
3,262,760	T→C	2.30%	V76A (GTC→GCC)	*yhaB* →	predicted protein
3,299,486	+C	44.90%	coding (1012/1245 nt)	*mtr* ←	tryptophan transporter of high affinity
3,309,669	C→T	3.40%	M342I (ATG→ATA)	*infB* ←	fused protein chain initiation factor 2, IF2: membrane protein/conserved protein
3,316,099	T→C	100%	A65A (GCA→GCG)	*yhbX* ←	predicted hydrolase, inner membrane
3,342,775	G→A	7.50%	A44T (GCT→ACT)	*npr* →	phosphohistidinoprotein-hexose phosphotransferase component of N-regulated PTS system (Npr)
3,346,233	G→A	42.60%	S491S (AGC→AGT)	*arcB* ←	aerobic respiration control sensor histidine protein kinase, cognate to two-component response regulators ArcA and RssB
3,349,579	T→C	100%	L59L (TTA→CTA)	*gltB* →	glutamate synthase, large subunit
3,370,569	+G	15.60%	coding (1021/1368 nt)	*dcuD* →	predicted transporter
3,392,252	G→A	3.30%	P75L (CCG→CTG)	*rng* ←	ribonuclease G
3,406,122	Δ1 bp	1.80%	coding (172/297 nt)	*fis* →	global DNA-binding transcriptional dual regulator
3,406,123	Δ1 bp	1.80%	coding (173/297 nt)	*fis* →	global DNA-binding transcriptional dual regulator
3,406,124	Δ1 bp	1.80%	coding (174/297 nt)	*fis* →	global DNA-binding transcriptional dual regulator
3,406,125	Δ1 bp	1.80%	coding (175/297 nt)	*fis* →	global DNA-binding transcriptional dual regulator
3,411,319	C→T	2.50%	S536F (TCC→TTC)	*acrF* →	multidrug efflux system protein
3,427,668	+C	2.60%	coding (573/1125 nt)	*smf* ←	conserved protein
3,432,306	T→C	44.70%	V370A (GTT→GCT)	*trkA* →	NAD-binding component of TrK potassium transporter
3,451,478	Δ1 bp	3.40%	coding (422/1953 nt)	*gspD* →	general secretory pathway component, cryptic
3,471,744	T→C	4.30%	K119R (AAA→AGA)	*fkpA* ←	FKBP-type peptidyl-prolyl cis-trans isomerase (rotamase)
3,543,623	C→T	100%	A376A (GCG→GCA)	*malQ* ←	4-alpha-glucanotransferase (amylomaltase)
3,545,374	C→T	3.40%	D594N (GAC→AAC)	*malP* ←	maltodextrin phosphorylase
3,548,610	Δ1 bp	4.00%	coding (846/2706 nt)	*malT* →	DNA-binding transcriptional activator for the mal regulon and maltotriose-ATP-binding protein
3,571,562	G→A	12.20%	G39G (GGC→GGT)	*gntU* ←	gluconate transporter, low affinity GNT 1 system
3,579,523	C→A	2.90%	R29S (CGC→AGC)	*yrhB* →	predicted protein
3,600,138	Δ1 bp	2.30%	coding (153/360 nt)	*yhhM* ←	conserved protein, DUF2500 family
3,608,549	T→C	5.30%	V68A (GTG→GCG)	*nikA* →	Nickel-binding, heme-binding periplasmic protein
3,634,552	Δ1 bp	4.90%	intergenic (-152/-239)	*uspB* ← /	universal stress (ethanol tolerance) protein B/universal stress global response regulator
3,655,686	T→C	2.50%	L198P (CTG→CCG)	*mdtF* →	anaerobic multidrug efflux transporter, ArcA- regulated
3,664,698	Δ1 bp	3.50%	coding (428/1650 nt)	*treF* →	cytoplasmic trehalase
3,672,140	A→G	5.70%	V297A (GTT→GCT)	*yhjG* ←	Inner membrane protein, AsmA family
3,685,449	T→C	100%	D613G (GAC→GGC)	*bcsB* ←	regulator of cellulose synthase, cyclic di-GMP binding
3,710,670	G→C	3.00%	A117G (GCG→GGG)	*bisC* ←	biotin sulfoxide reductase
3,764,113	G→T	1.90%	intergenic (+179/-30)	*yibW* → /	pseudogene, rhsA-linked/hypothetical protein, no homologs
3,787,366	T→A	2.40%	intergenic (-137/+138)	*kbl* ← / ←	glycine C-acetyltransferase/conserved protein, pfam09612 family
3,793,369	T→C	100%	T208A (ACA→GCA)	*waaU* ←	lipopolysaccharide core biosynthesis
3,810,479	Δ1 bp	3.30%	intergenic (-33/+62)	*pyrE* ← /	orotate phosphoribosyltransferase/defective ribonuclease PH
3,810,557	+C	25.00%	coding (671/687 nt)	*rph* ←	defective ribonuclease PH
3,837,297	T→C	2.90%	T391A (ACC→GCC)	*yicO* ←	Probable adenine permease
3,879,160	+T	7.30%	intergenic (+6/-11)	*rpmH* → /	50S ribosomal subunit protein L34/protein C5 component of RNase P
3,883,353	G→T	2.10%	intergenic (+166/-55)	*tnaC* → /	tryptophanase leader peptide/tryptophanase/L-cysteine desulfhydrase, PLP- dependent

3,900,309	Δ1 bp	3.40%	intergenic (−34/+100)	*bglF* ← /	fused beta−glucoside−specific PTS enzymes: IIA component/IIB component/IIC component/transcriptional antiterminator of the bgl operon
3,960,228	Δ1 bp	13.50%	coding (81/1266 nt)	*rhlB* ←	ATP−dependent RNA helicase
4,003,806	+A	3.10%	intergenic (−69/−42)	*rhtB* ← /	homoserine, homoserine lactone and S−methyl− methionine efflux pump/lysophospholipase L2
4,004,074	A→G	4.10%	D76G (GAC→GGC)	*pldB* →	lysophospholipase L2
4,037,965	A→G	100%	intergenic (−57/−307)	*yihG* ← /	inner membrane protein, Predicted acyltransferas/fused DNA polymerase I 5'−>3' polymerase/3'−>5' exonuclease/5'−>3' exonuclease
4,094,124	A→G	100%	intergenic (+145/−4)	*kdgT* → /	2−keto−3−deoxy−D−gluconate transporter/6−N−hydroxylaminopurine resistance protein
4,113,515	C→T	2.10%	intergenic (−3/+171)	*hslV* ← /	peptidase component of the HslUV protease/essential cell division protein
4,124,006	Δ1 bp	2.70%	coding (85/891 nt)	*metF* →	5,10−methylenetetrahydrofolate reductase
4,156,496	C→T	18.10%	R518C (CGT→TGT)	*btuB* →	vitamin B12/cobalamin outer membrane transporter
4,175,120	T→C	11.80%	V857A (GTG→GCG)	*rpoB* →	RNA polymerase, beta subunit
4,188,448	A→G	2.30%	Q80Q (CAA→CAG)	*nudC* →	NADH pyrophosphatase
4,190,927	A→G	5.60%	T40A (ACC→GCC)	*yjaG* →	conserved protein
4,225,439	Δ1 bp	2.10%	coding (376/1650 nt)	*pgi* →	glucosephosphate isomerase
4,284,406	Δ1 bp	3.60%	coding (1538/1608 nt)	*nrfE* →	heme lyase (NrfEFG) for insertion of heme into c552, subunit NrfE
4,287,686	+CG	100%	intergenic (+586/+56)	*gltP* → /	glutamate/aspartate:proton symporter/conserved protein
4,303,503	Δ1 bp	22.40%	coding (795/891 nt)	*rpiR* ←	DNA−binding transcriptional repressor
4,313,141	G→A	6.60%	C196C (TGC→TGT)	*phnF* ←	predicted DNA−binding transcriptional regulator of phosphonate uptake and biodegradation
4,330,536	T→C	4.20%	N431S (AAC→AGC)	*adiA* ←	arginine decarboxylase
4,381,847	T→C	3.50%	E274E (GAA→GAG)	*rsgA* ←	ribosome small subunit−dependent GTPase A
4,401,351	Δ1 bp	10.50%	intergenic (+39/−88)	*rlmB* → /	23S rRNA (Gm2251)−methyltransferase/conserved protein, DUF2170 family
4,402,611	T→C	4.80%	S2P (TCT→CCT)	*yjfK* →	conserved protein, DUF2491 family
4,447,338	Δ1 bp	4.50%	coding (248/1374 nt)	*mpl* →	UDP−N−acetylmuramate:L−alanyl−gamma−D−glutamyl− meso−diaminopimelate ligase
4,453,646	C→G	2.20%	E107D (GAG→GAC)	*nrdD* ←	anaerobic ribonucleoside−triphosphate reductase
4,453,651	C→G	2.50%	V106L (GTC→CTC)	*nrdD* ←	anaerobic ribonucleoside−triphosphate reductase
4,457,492	C→T	15.40%	intergenic (−6/+113)	*treB* ← /	fused trehalose(maltose)−specific PTS enzyme: IIB component/IIC component/DNA−binding transcriptional repressor
4,457,497	G→A	26.80%	intergenic (−11/+108)	*treB* ← /	fused trehalose(maltose)−specific PTS enzyme: IIB component/IIC component/DNA−binding transcriptional repressor
4,457,498	G→A	7.70%	intergenic (−12/+107)	*treB* ← /	fused trehalose(maltose)−specific PTS enzyme: IIB component/IIC component/DNA−binding transcriptional repressor
4,464,733	G→A	2.20%	V209V (GTC→GTT)	*yjgI* ←	predicted oxidoreductase with NAD(P)−binding Rossmann−fold domain
4,471,270	+T	100%	pseudogene (262/291 nt)	*yjgN* →	inner membrane protein, DUF898 family
4,471,854	G→A	6.80%	pseudogene (550/927 nt)	*yjgN* →	inner membrane protein, DUF898 family
4,479,877	+C	5.20%	coding (1492/1503 nt)	*yjgR* ←	conserved protein, DUF853 family with NTPase fold
4,500,691	G→A	100%	pseudogene (378/543 nt)	*insO* →	pseudogene, IS911 transposase B;IS, phage, Tn; Transposon−related functions; extrachromosomal; transposon related
4,548,065	+T	96.80%	intergenic (−440/−232)	*yjiC* ← /	predicted protein/RpoS stabilzer after DNA damage, anti−RssB factor
4,594,767	G→C	2.20%	intergenic (−605/−14)	*yjjP* ← /	inner membrane protein, H−NS−repressed, DUF1212 family/DNA−binding transcriptional regulator
4,597,511	+G	4.60%	noncoding (80/87 nt)	*leuP* ←	tRNA−Leu
4,597,626	+G	6.40%	noncoding (80/87 nt)	*leuQ* ←	tRNA−Leu
4,605,748	A→G	19.70%	intergenic (+184/+236)	*yjjV* → /	predicted DNase/predicted pyruvate formate lyase activating enzyme
4,605,807	T→C	20.60%	intergenic (+243/+177)	*yjjV* → /	predicted DNase/predicted pyruvate formate lyase activating enzyme
4,631,366	C→T	10.60%	G82D (GGC→GAC)	*arcA* ←	DNA−binding response regulator in two−component regulatory system with ArcB or CpxA
