# Supplementary material for: Chance and necessity in the genome evolution of endosymbiotic bacteria of insects
Source: ISME J. 2017 Mar 21;11(6):1291–304. doi: 10.1038/ismej.2017.18 (PMC5437351; doi:10.1038/ismej.2017.18)
Supplement: Supplementary Table S7 [file ismej201718x7.pdf]

Table S7. Fixed mutations in an *E. coli* evolving population under mild genetic drift effect

Genome position	Mutation	%individuals	Kind	Gene	Description
45,078	C→T	100%	A300V (G	*fixC* →	predicted oxidoreductase with FAD/NAD(P)-binding domain
83,967	C→T	100%	S61L (TC	*cra* →	DNA-binding transcriptional repressor-activator for carbon metabolism
103,188	+T	100%	intergenic	*lpxC* → /	UDP-3-O-acyl N-acetylglucosamine deacetylase/regulator of secA translation
302,914	+C	100%	coding (1	*yagX* ←	predicted aromatic compound dioxygenase
305,276	G→A	100%	S88L (TC	*matB* ←	cryptic Mat fimbrillin gene
518,073	+T	100%	intergenic	*ybbP* → /	predicted ABC transporter permease/rhsD element protein
539,430	A→G	100%	S243S (A	*allC* ←	allantoate amidohydrolase
539,932	A→G	100%	V76A (GT	*allC* ←	allantoate amidohydrolase
566,913	T→C	100%	R198R (C	*ybcM* →	DLP12 prophage; predicted DNA-binding transcriptional regulator
704,595	A→G	100%	T466A (A	*chiP* →	chitoporin, uptake of chitosugars
919,281	A→G	100%	E170E (G	*macB* →	fused macrolide transporter subunits of ABC superfamily: ATP-binding component/membrane component
980,270	Δ1 bp	100%	coding (7	*ycbB* →	murein L,D-transpeptidase
1,087,350	Δ1 bp	100%	coding (9	*pgaB* ←	biofilm adhesin polysaccharide PGA export lipoprotein with a polysaccharide deacetylase activity needed for export
1,124,216	A→G	100%	Y77C (TA	*rimJ* →	ribosomal-protein-S5-alanine N- acetyltransferase
1,172,430	Δ1 bp	100%	intergenic	*mfd* ← / ←	transcription-repair coupling factor/inner membrane protein
1,214,628	G→A	100%	D46N (GA	*ymgA* →	connector protein for RcsB regulation of biofilm
1,297,920	IS*5* (+) +4	100%	intergenic	*ychE* → /	predicted inner membrane protein/oligopeptide transporter subunit
1,377,956	A→G	100%	T195A (A	*ycjU* →	beta-phosphoglucomutase
1,400,464	A→G	100%	R6R (CG	*abgB* ←	p-aminobenzoyl-glutamate hydrolase, B subunit
1,517,957	A→G	100%	Q157R (C	*mcbR* →	colanic acid and biofilm gene transcriptional regulator, MqsR-controlled
1,537,360	T→C	100%	S819S (T	*narZ* ←	nitrate reductase 2 (NRZ), alpha subunit
1,546,080	A→G	100%	N289S (A	*fdnG* →	formate dehydrogenase-N, alpha subunit, nitrate- inducible
1,711,543	Δ1 bp	100%	intergenic	*dtpA* → /	dipeptide and tripeptide permease A/glutathionine S-transferase
1,854,456	+C	100%	coding (5	*ydjI* ←	predicted aldolase
1,933,749	+A	100%	intergenic	*zwf* ← / –	glucose-6-phosphate 1-dehydrogenase/predicted DNA-binding transcriptional regulator
2,106,695	A→G	100%	G38G (G	*rfbX* ←	predicted polisoprenol-linked O-antigen transporter
2,661,742	T→C	100%	G43G (G	*yfhR* →	S9 peptidase family protein, function unknown
2,734,342	IS*1* (+) +8	100%	intergenic	*bamD* →	lipoprotein required for OM biogenesis, in BamABCDE complex/cold shock protein associated with 30S ribosomal subunit
3,004,979	T→C	100%	I91I (ATT	*hyuA* →	D-stereospecific phenylhydantoinase
3,316,099	T→C	100%	A65A (GC	*yhbX* ←	predicted hydrolase, inner membrane
3,349,579	T→C	100%	L59L (TTA	*gltB* →	glutamate synthase, large subunit
3,543,623	C→T	100%	A376A (G	*malQ* ←	4-alpha-glucanotransferase (amylomaltase)
3,685,449	T→C	100%	D613G (C	*bcsB* ←	regulator of cellulose synthase, cyclic di-GMP binding
3,793,369	T→C	100%	T208A (A	*waaU* ←	lipopolysaccharide core biosynthesis
4,037,965	A→G	100%	intergenic	*yihG* ← /	inner membrane protein, Predicted acyltransferas/fused DNA polymerase I 5'->3' polymerase/3'->5' exonuclease/5'->3' exonuclease
4,094,124	A→G	100%	intergenic	*kdgT* → /	2-keto-3-deoxy-D-gluconate transporter/6-N-hydroxylaminopurine resistance protein
4,287,686	+CG	100%	intergenic	*gltP* → / ←	glutamate/aspartate:proton symporter/conserved protein
4,471,270	+T	100%	pseudoge	*yjgN* →	inner membrane protein, DUF898 family
4,500,691	G→A	100%	pseudoge	*insO* →	pseudogene, IS911 transposase B;IS, phage, Tn; Transposon-related functions; extrachromosomal; transposon related
