# Supplementary material for: Chance and necessity in the genome evolution of endosymbiotic bacteria of insects
Source: ISME J. 2017 Mar 21;11(6):1291–304. doi: 10.1038/ismej.2017.18 (PMC5437351; doi:10.1038/ismej.2017.18)
Supplement: Supplementary Table S8 [file ismej201718x8.pdf]

Table S8. Transcriptomic data for the experimentally evolving Escherichia coli line A. Line A was evolved for 250 passages and the transcriptomic analyses was performed at three time points (ancestral, at 200 passages, and at 250 passages). Fold change was calculated as the fraction of read of the evolved line and the ancestral line. the column designed as "d" highlights genes which show no evidence for differential expression (identified as 0), higher expression (identified as 1) or lower expression (identified as -1) when the evolved line was compared to its ancestral line.

	t200 vs t0					t250 vs t0					t250 vs t200				
Identifier	foldChange	log2FoldCha	pval	padj	d	foldChange	log2FoldCha	pval	padj	d	foldChange	log2FoldCha	pval	padj	d
thrL	1.3142671	0.39425851	0.62980627	0.68875793	0	0.63764571	-0.649173	0.05699533	0.09609399	0	0.48517209	-1.0434315	0.01501101	0.12585276	0
thrA	1.08736094	0.12083091	0.82854623	0.86191443	0	0.9089445	-0.1377359	0.95785571	0.97114015	0	0.83591792	-0.2585668	0.74015049	0.95683847	0
yaaW	4.43799086	2.14990669	0.00032137	0.00079837	1	4.72438003	2.24012502	0.0002497	0.00080485	1	1.06453127	0.09021833	0.60850223	0.92806272	0
zapD	0.34176737	-1.5489134	0.00017497	0.00046369	-1	0.39350317	-1.3455528	0.00456386	0.01049308	-1	1.15137724	0.2033606	0.72890523	0.95488877	0
gfcC	7.95515176	2.99188945	5.47E-05	0.00016302	1	7.84953151	2.97260665	6.15E-05	0.00023325	1	0.98672304	-0.0192829	0.69239633	0.94603882	0
gfcB	5.89375234	2.55918644	1.48E-05	4.88E-05	1	5.99448128	2.58363492	0.00014908	0.00051308	1	1.0170908	0.02444848	0.90502039	0.99890653	0
gfcA	1.25940897	0.33274685	0.67026232	0.72420466	0	1.54302083	0.62575754	0.37214625	0.46350459	0	1.22519441	0.29301069	0.60277254	0.92806272	0
insA4	NA	NA	NA	NA	0	Inf	Inf	0.65167999	0.72678285	0	Inf	Inf	0.72317611	0.95399541	0
insB1	1.54215482	0.62494761	0.96526652	0.97589785	0	1.37866307	0.46326992	0.88814786	0.91528111	0	0.89398486	-0.1616777	1	1	0
cspH	2.32316415	1.2160911	0.17859154	0.23373578	0	3.33134942	1.73610668	0.10448155	0.16228611	0	1.43397074	0.52001559	0.60247426	0.92806272	0
cspG	0.51675537	-0.9524466	0.27069977	0.33709638	0	1.30575565	0.38488495	0.94687891	0.96322695	0	2.52683517	1.33733156	0.27547314	0.77762465	0
ymcE	1.48896183	0.57430677	0.56096737	0.62633793	0	1.33418732	0.41596124	0.78536304	0.8362697	0	0.89605206	-0.1583455	0.82511109	0.97661485	0
gnsA	0.54290999	-0.8812151	0.06820506	0.09932557	0	0.52909413	-0.9184037	0.09056021	0.14364163	0	0.97455221	-0.0371886	0.83086332	0.97888613	0
yccM	0.687695	-0.5401592	0.13809143	0.18624423	0	0.94145244	-0.0870399	0.84042866	0.8795278	0	1.36899708	0.45311937	0.28750098	0.78998378	0
coaE	0.36392268	-1.4582961	0.00058918	0.00138648	-1	0.6598912	-0.5996999	0.22884128	0.31339007	0	1.81327309	0.85859622	0.08017776	0.40043479	0
torS	1.81519433	0.86012401	0.03588761	0.05649166	0	1.83603622	0.87659452	0.02730949	0.0507488	0	1.01148191	0.01647052	0.67769802	0.94128448	0
torT	1.3144351	0.39444291	0.35719806	0.42769338	0	1.70473197	0.76954493	0.14518554	0.21392962	0	1.29693126	0.37510202	0.45663899	0.88755478	0
torR	0.66606843	-0.5862577	0.12642348	0.17219011	0	0.72807531	-0.4578404	0.46811431	0.55757335	0	1.09309386	0.12841728	0.60688352	0.92806272	0
torC	3.97890869	1.99237279	0.02048183	0.03399783	1	4.04835882	2.01733717	0.02178081	0.04164017	1	1.01745457	0.02496437	0.78636625	0.9635071	0
torA	4.00198313	2.00071509	6.54E-07	2.71E-06	1	6.16246407	2.62350733	2.70E-08	2.20E-07	1	1.53985259	0.62279224	0.14742181	0.58035154	0
torD	1.1769428	0.23504421	0.76846358	0.81320164	0	1.04118684	0.05822898	0.84692288	0.884555	0	0.88465373	-0.1768152	0.90647563	0.99909148	0
cbpM	1.24433745	0.31537778	0.56208551	0.62742777	0	0.33443822	-1.5801884	0.00091039	0.00253498	-1	0.26876811	-1.8955661	8.25E-05	0.00229478	-1
cbpA	0.87934393	-0.1855005	0.57061327	0.63566174	0	0.23631438	-2.0812207	5.13E-06	2.57E-05	-1	0.26873942	-1.8957201	2.06E-05	0.00074913	-1
yccE	4.9514766	2.30785882	0.00012209	0.00033371	1	4.28003979	2.09762421	0.00540322	0.01220697	1	0.86439665	-0.2102346	0.68497931	0.943596	0
agp	0.34441234	-1.5377913	5.24E-07	2.19E-06	-1	0.06657205	-3.9089397	8.36E-21	4.68E-19	-1	0.1932917	-2.3711484	7.64E-10	1.16E-07	-1
guaC	0.43207616	-1.2106425	9.10E-05	0.0002557	-1	0.51290881	-0.9632257	0.01189573	0.02442715	0	1.18707964	0.24741673	0.49851842	0.90539057	0
yccJ	1.00587518	0.00845129	0.84063528	0.87264066	0	0.1365925	-2.8720499	7.00E-05	0.00026193	-1	0.13579468	-2.8805011	6.43E-05	0.0019081	-1
wrbA	0.86580207	-0.2078908	0.54188412	0.60918864	0	0.14940989	-2.7426525	1.75E-05	7.63E-05	-1	0.17256818	-2.5347616	5.59E-05	0.00171324	-1
ymdF	9.53102981	3.2526321	0.00875933	0.01585654	1	0.61131447	-0.7100134	0.5304996	0.61691519	0	0.06413939	-3.9626455	0.00871118	0.08461744	0
rutG	2.91108163	1.54155529	0.00012353	0.00033703	1	4.48065134	2.16370847	4.68E-07	2.96E-06	1	1.53917063	0.62215318	0.07402192	0.37957754	0
rutF	2.45313022	1.29462382	0.02866367	0.04608935	1	3.4976388	1.80638131	0.02996451	0.05519514	0	1.42578603	0.51175749	0.62035773	0.92923437	0
rutE	7.07173556	2.82206433	7.25E-07	2.98E-06	1	9.13128349	3.19081766	2.37E-07	1.61E-06	1	1.29123656	0.36875333	0.36991301	0.85484012	0
rutD	5.2345593	2.38806808	0.00029451	0.00073829	1	9.06129289	3.17971691	9.45E-06	4.46E-05	1	1.73105172	0.79164883	0.16784687	0.61890024	0
rutC	3.95021589	1.9819315	0.00302876	0.00609756	1	6.07608074	2.60314104	0.00013044	0.00045457	1	1.53816422	0.62120954	0.20590721	0.68468503	0
rutB	6.0206314	2.58991479	6.12E-05	0.00018049	1	4.94554692	2.30613008	0.00029284	0.00092566	1	0.82143327	-0.2837847	1	1	0
rutA	9.96960672	3.31753659	2.83E-10	1.86E-09	1	12.307062	3.62141449	2.13E-10	2.50E-09	1	1.23445812	0.3038779	0.39675595	0.86854717	0
hofC	2.16901417	1.11703948	0.00868888	0.01573545	1	3.92521758	1.97277263	9.29E-05	0.00033707	1	1.80967816	0.85573315	0.07493372	0.38291737	0
rutR	0.78724836	-0.3451092	0.44928509	0.51963661	0	0.51572004	-0.95534	0.14109781	0.20846246	0	0.65509193	-0.6102307	0.3689392	0.85484012	0
putA	1.9750829	0.98191321	0.00316063	0.00633129	0	0.73876536	-0.4368119	0.32794917	0.41742208	0	0.37404271	-1.4187251	0.00033991	0.00707072	-1
putP	2.23763583	1.16197526	0.00041736	0.00101013	1	1.4338661	0.51991031	0.10003599	0.15670296	0	0.64079511	-0.6420649	0.16584831	0.61513122	0
efeU	2.23068744	1.15748838	0.00674769	0.01251766	1	2.62176957	1.39054089	0.00387602	0.00909136	1	1.17531911	0.23305251	0.52931929	0.91648924	0
efeO	0.83600497	-0.2584166	0.65501555	0.71068624	0	1.36460472	0.44848311	0.35479138	0.44603787	0	1.6322926	0.70689969	0.16447016	0.61352961	0
efeB	16.9558793	4.0837137	1.03E-13	9.36E-13	1	7.94220067	2.98953881	4.02E-05	0.00016157	1	0.46840394	-1.0941749	0.01596021	0.13124263	0
phoH	7.72156928	2.94889408	0.00074189	0.00171657	1	0.86116348	-0.215641	0.89343911	0.91878132	0	0.111527	-3.164535	0.0036627	0.04377371	-1
pgaD	1.75668771	0.81285775	0.27892169	0.3456782	0	4.16835286	2.05947741	0.00721581	0.01578549	1	2.37284796	1.24661966	0.06995233	0.36812622	0
pgaC	6.87407427	2.78116544	3.60E-10	2.33E-09	1	9.58390113	3.26061302	1.15E-08	1.01E-07	1	1.39420972	0.47944759	0.61017551	0.92872856	0
pgaB	5.15683158	2.36648493	2.96E-06	1.12E-05	1	10.2298957	3.35471952	1.63E-07	1.15E-06	1	1.98375601	0.9882346	0.15137956	0.58738081	0
hofB	1.9460104	0.96051942	0.01878776	0.03142201	0	1.7561795	0.81244031	0.02178594	0.04164017	0	0.90245124	-0.1480791	0.82493359	0.97661485	0
pgaA	3.01056106	1.59003238	1.11E-05	3.77E-05	1	7.47588071	2.90224355	2.42E-09	2.39E-08	1	2.48321843	1.31221117	0.01014696	0.09400856	0
ycdT	1.93645449	0.95341759	0.03520879	0.05560693	0	2.00293653	1.0021167	0.10429584	0.16216866	0	1.03433184	0.04869911	0.91085186	0.99971546	0
insF1	0	#NAME?	1	1	0	1.08414595	0.11655899	0.76786623	0.82159827	0	Inf	Inf	0.78945554	0.96413706	0
ymdE	12.8046129	3.67859174	0.00022321	0.00057656	1	9.2989701	3.21707094	0.00197435	0.00502573	1	0.72622032	-0.4615208	0.82633121	0.97697604	0
ycdU	6.09869291	2.60850007	6.48E-05	0.00018989	1	3.28971596	1.71796302	0.00700482	0.0153903	1	0.53941328	-0.890537	0.37983545	0.85988523	0
serX	1.61756825	0.69382658	0.73746653	0.78604963	0	1.53298234	0.61634108	0.80273649	0.84904082	0	0.94770798	-0.0774855	1	1	0
ghrA	0.29669268	-1.7529588	3.53E-07	1.51E-06	-1	0.28841676	-1.7937731	1.35E-05	6.14E-05	-1	0.97210609	-0.0408143	0.90545477	0.99890653	0
ycdX	0.35786306	-1.4825205	4.91E-06	1.78E-05	-1	0.4009002	-1.318685	0.00148995	0.00393315	-1	1.12026147	0.1638355	0.58415441	0.92806272	0
ycdY	0.23970532	-2.0606662	4.43E-09	2.49E-08	-1	0.24998343	-2.0000956	2.47E-05	0.00010392	-1	1.04287812	0.06057056	0.56624954	0.92590304	0
ppdD	7.41636337	2.89071193	5.74E-05	0.00017003	1	6.96951272	2.80105779	7.59E-05	0.00028083	1	0.93974801	-0.0896541	0.72622429	0.95487077	0
ycdZ	0.07648999	-3.7085852	2.61E-24	5.36E-23	-1	0.11062565	-3.1762421	7.65E-14	1.49E-12	-1	1.44627613	0.53234303	0.19008018	0.66370038	0
csgG	2.02421662	1.01736369	0.01708572	0.02895918	1	1.5744307	0.65483026	0.08996228	0.14279573	0	0.77779754	-0.3625334	0.75430284	0.9573569	0
csgF	6.51633402	2.70406056	0.00192397	0.00406028	1	4.45506686	2.15544708	0.02442515	0.04595759	1	0.68367687	-0.5486135	0.71239655	0.95223057	0
csgE	6.11011614	2.6111998	0.00223089	0.00462404	1	7.10161634	2.82814742	0.00549447	0.01238148	1	1.16227191	0.21694762	0.87679658	0.98956956	0
csgD	4.30229617	2.10510684	0.00084458	0.00192394	1	1.92884564	0.94773769	0.19371095	0.27278798	0	0.44832935	-1.1573691	0.11203201	0.49574884	0
csgB	5.30112214	2.40629778	0.00012149	0.00033227	1	2.71178234	1.43924139	0.04676416	0.08126262	0	0.51154874	-0.9670564	0.21196915	0.69345989	0
csgA	3.52793457	1.81882381	0.00029073	0.00073089	1	2.19311578	1.13298198	0.02874341	0.0531008	0	0.62164299	-0.6858418	0.33379529	0.83424743	0
csgC	1.54952817	0.63182899	0.18900795	0.24612268	0	1.08743922	0.12093477	0.98435687	0.99585005	0	0.70178732	-0.5108942	0.28519691	0.78928465	0
ymdA	0.17116648	-2.5465279	1.37E-05	4.54E-05	-1	0.18298609	-2.4501941	0.00081902	0.0022994	-1	1.06905331	0.09633379	0.73148195	0.95551996	0
ymdB	0.64271582	-0.6377471	0.06145413	0.09090519	0	0.41980591	-1.2522056	0.01124871	0.02330429	-1	0.65317502	-0.6144585	0.24848964	0.74826869	0
nadC	0.47218034	-1.0825901	0.00211259	0.00441197	-1	0.57231251	-0.8051249	0.04989175	0.08582003	0	1.2120634	0.27746517	0.56980227	0.92806272	0
clsC	2.18558823	1.12802162	0.00095413	0.00215129	1	1.75239023	0.80932408	0.02004145	0.03869078	0	0.80179341	-0.3186975	0.61516172	0.92897598	0
opgC	2.77157499	1.47070604	0.00102316	0.00229174	1	2.85998209	1.51600611	0.00227133	0.00569315	1	1.03189778	0.04530007	0.78014276	0.9635071	0
opgG	0.44632983	-1.1638179	0.00039723	0.00096723	-1	0.44609492	-1.1645774	0.00188115	0.00481066	-1	0.99947368	-0.0007595	0.86216928	0.98425227	0
opgH	0.53060476	-0.9142905	0.00596675	0.01120542	0	0.77208547	-0.3731675	0.29426071	0.38426067	0	1.45510468	0.54112294	0.1788939	0.64296831	0
yceK	7.60424214	2.92680447	0.00024809	0.00063489	1	1.66123953	0.73226011	0.36607715	0.45749291	0	0.21846221	-2.1945444	0.02028489	0.15732979	0
msyB	5.34943396	2.41938624	6.63E-05	0.00019384	1	1.11349919	0.1551005	0.92027892	0.94115079	0	0.20815271	-2.2642857	0.00073856	0.01340345	-1
mdtG	1.62494184	0.70038808	0.03823322	0.05992766	0	2.05718149	1.04066907	0.03885639	0.06940436	0	1.26600315	0.34028099	0.62982197	0.93109012	0
lpxL	0.98936095	-0.0154311	0.9676273	0.97803428	0	2.85017837	1.51105221	0.29490317	0.38484919	0	2.88082764	1.52648335	0.24567354	0.74461877	0
yceA	1.9798563	0.98539572	0.01797125	0.03025114	0	4.26447433	2.09236791	5.85E-05	0.00022348	1	2.15393124	1.10697219	0.02995462	0.21001573	0
yceI	0.93554865	-0.0961154	0.97368819	0.98258641	0	0.54008656	-0.8887374	0.08993536	0.14279573	0	0.57729394	-0.792622	0.09850729	0.45734597	0
ampD	0.47905141	-1.0617476	0.00790147	0.01444965	-1	0.4975088	-1.0072061	0.02160007	0.04137439	-1	1.03852902	0.05454153	0.94470146	1	0
yceJ	2.92412468	1.54800483	0.00516081	0.00980865	1	2.46468753	1.30140475	0.01557003	0.03093702	1	0.84288045	-0.2466001	0.99286979	1	0
yceO	3.07010532	1.61828815	0.27350342	0.33982127	0	3.79481503	1.92402957	0.21486204	0.2970824	0	1.2360537	0.30574142	0.76218653	0.95858924	0
solA	0.49522685	-1.0138385	0.00517479	0.00983098	-1	0.5467409	-0.8710708	0.08726362	0.13926252	0	1.10402111	0.14276776	0.50777689	0.90925684	0
bssS	0.20794231	-2.2657448	1.03E-10	6.99E-10	-1	0.10324491	-3.2758574	8.94E-15	1.98E-13	-1	0.49650747	-1.0101127	0.00174373	0.02597927	-1
dinI	0.30581342	-1.7092764	0.00053783	0.00127651	-1	0.56960497	-0.8119664	0.03869108	0.06919299	0	1.86258983	0.89731	0.41234717	0.87131399	0
pyrC	0.70744573	-0.4993086	0.28828134	0.35564685	0	0.88240974	-0.1804794	0.84051678	0.8795278	0	1.24731793	0.31823924	0.45194837	0.88755478	0
yceB	0.56729114	-0.8178388	0.02689728	0.04356629	0	0.67299948	-0.5713227	0.0839298	0.13506402	0	1.18633878	0.24651606	0.95475938	1	0
grxB	0.65862109	-0.6024794	0.14795494	0.19827578	0	0.20724589	-2.2705846	7.48E-05	0.0002776	-1	0.31466635	-1.6681052	0.00237107	0.03193373	-1
mdtH	3.57124789	1.83642828	0.00023165	0.00059731	1	3.94423851	1.9797468	0.00058351	0.00169639	1	1.10444266	0.14331852	0.69941702	0.94869712	0
rimJ	0.1652118	-2.5976114	9.81E-14	8.94E-13	-1	0.14206492	-2.8153777	6.14E-11	7.67E-10	-1	0.85989574	-0.2177663	0.58348626	0.92806272	0
ampE	0.39141057	-1.3532454	0.00032527	0.00080759	-1	0.33890275	-1.5610568	0.00077873	0.00219884	-1	0.86584977	-0.2078114	0.76449815	0.95942995	0
yceH	0.14554225	-2.78049	2.72E-15	2.87E-14	-1	0.13832833	-2.8538314	1.17E-11	1.61E-10	-1	0.95043418	-0.0733414	0.76058907	0.95840749	0
yceM	0.45678862	-1.1304014	0.00064847	0.0015107	-1	0.39229802	-1.349978	0.00052695	0.00154416	-1	0.85881742	-0.2195766	0.47469256	0.89845244	0
murJ	2.0032453	1.00233909	0.00443182	0.00853701	1	2.48518836	1.3133552	0.00132079	0.00353947	1	1.24058115	0.31101611	0.38295365	0.86038969	0
flgN	0.12839202	-2.9613725	1.70E-14	1.65E-13	-1	0.16771398	-2.5759251	2.58E-08	2.12E-07	-1	1.30626478	0.38544736	0.38135564	0.85988523	0

flgM	0.02143804	-5.5436835	1.90E-37	9.77E-36	-1	0.09199744	-3.4422625	2.72E-13	4.86E-12	-1	4.29131852	2.10142099	8.96E-07	5.81E-05	1
flgA	0.03464604	-4.8511657	4.50E-37	2.18E-35	-1	0.08456987	-3.5637125	3.75E-17	1.21E-15	-1	2.44096778	1.28745325	0.0014497	0.02259077	1
flgB	0.00586812	-7.4128866	9.98E-73	7.35E-70	-1	0.02630594	-5.2484678	1.69E-32	4.97E-30	-1	4.48285791	2.16441877	3.73E-08	3.50E-06	1
flgC	0.00639196	-7.2895261	2.81E-68	1.24E-65	-1	0.02624663	-5.2517242	1.51E-31	3.92E-29	-1	4.10619438	2.03780192	3.80E-07	2.75E-05	1
flgD	0.00875827	-6.8351378	4.88E-64	1.08E-61	-1	0.03094887	-5.0139692	1.07E-29	2.25E-27	-1	3.53367295	1.82116852	1.27E-06	7.35E-05	1
flgE	0.00848011	-6.8817013	1.99E-68	9.75E-66	-1	0.03106034	-5.0087826	7.87E-31	1.93E-28	-1	3.66272849	1.87291876	2.11E-07	1.61E-05	1
aroP	0.91602287	-0.1265445	0.74181959	0.78973659	0	1.24720337	0.31869673	0.3896969	0.48075672	0	1.36154174	0.44524121	0.24359564	0.74035615	0
flgF	0.0073405	-7.0899066	7.12E-66	1.97E-63	-1	0.04381375	-4.5124725	3.90E-25	4.42E-23	-1	5.96877211	2.57743417	2.43E-11	5.37E-09	1
flgG	0.01304988	-6.2598194	2.50E-57	3.69E-55	-1	0.04790638	-4.3836384	6.79E-24	7.01E-22	-1	3.67102007	1.876181	1.21E-07	1.01E-05	1
flgH	0.01379635	-6.1795696	1.17E-48	1.12E-46	-1	0.06731248	-3.8929822	1.02E-17	3.64E-16	-1	4.87900667	2.28658745	7.66E-09	8.66E-07	1
flgI	0.04769872	-4.3899056	6.65E-31	2.37E-29	-1	0.09445954	-3.4041598	2.00E-13	3.67E-12	-1	1.98033681	0.98574582	0.00086128	0.01489515	0
flgJ	0.10551228	-3.2445172	2.58E-17	3.23E-16	-1	0.16362185	-2.6115627	1.82E-08	1.53E-07	-1	1.55073758	0.63295457	0.04793811	0.29039433	0
flgK	0.11385731	-3.1347012	7.97E-17	9.59E-16	-1	0.15361495	-2.7026095	3.36E-09	3.22E-08	-1	1.34918826	0.43209167	0.15629653	0.59676856	0
flgL	0.0917469	-3.4461967	4.49E-22	7.75E-21	-1	0.07365434	-3.7630857	1.45E-17	5.09E-16	-1	0.80279917	-0.316889	0.42215943	0.87445893	0
rne	0.18317857	-2.4486773	1.77E-14	1.71E-13	-1	0.19404382	-2.3655456	1.02E-09	1.07E-08	-1	1.05931506	0.08313174	0.80992732	0.96990832	0
yceQ	0.41103538	-1.2826655	0.00042784	0.00103154	-1	0.36355022	-1.4597734	0.00070336	0.00200527	-1	0.88447426	-0.1771079	0.5305472	0.91717489	0
rluC	1.02386132	0.03402032	0.87902743	0.90568327	0	1.52176897	0.60574935	0.27655908	0.36459265	0	1.4863038	0.57172903	0.29582356	0.79986628	0
yaaI	1.9578412	0.96926375	0.2413339	0.3047835	0	4.64483423	2.21562711	0.03526356	0.06378619	0	2.37242644	1.24636336	0.23004042	0.71837124	0
pdhR	1.20539561	0.26950671	0.5119352	0.58110575	0	0.85870086	-0.2197725	0.26057552	0.34777506	0	0.71238095	-0.4892792	0.08418949	0.41436912	0
yceF	0.4868085	-1.0385737	0.00122076	0.00268933	-1	0.31526347	-1.6653701	0.00019186	0.00063603	-1	0.64761292	-0.6267963	0.16623945	0.61554659	0
psrD	0.04499103	-4.4742188	1.07E-23	2.06E-22	-1	0.17749251	-2.4941699	2.67E-08	2.18E-07	-1	3.9450645	1.98004889	0.00036315	0.00744883	1
yceD	0.03641148	-4.7794627	4.75E-40	3.04E-38	-1	0.18074988	-2.4679334	3.09E-12	4.64E-11	-1	4.96409008	2.31152929	4.99E-08	4.49E-06	1
rpmF	0.02258274	-5.4686359	2.03E-49	1.99E-47	-1	0.11443971	-3.1273403	5.59E-17	1.75E-15	-1	5.06757523	2.3412956	1.56E-08	1.68E-06	1
plsX	0.12845263	-2.9606917	1.06E-17	1.37E-16	-1	0.30793811	-1.6992877	1.82E-06	1.01E-05	-1	2.39728928	1.26140401	0.00522691	0.05719769	0
fabH	0.33403799	-1.5819159	4.53E-06	1.66E-05	-1	0.55034515	-0.8615914	0.02010551	0.03876555	0	1.64755256	0.72032449	0.09669038	0.45507426	0
fabD	0.17725889	-2.4960701	2.91E-14	2.78E-13	-1	0.20333322	-2.2980822	3.84E-09	3.65E-08	-1	1.14709746	0.19798797	0.64471836	0.93208609	0
fabG	0.11607599	-3.1068585	3.06E-21	4.93E-20	-1	0.14403726	-2.795486	3.34E-13	5.85E-12	-1	1.24088764	0.31137249	0.52423642	0.91290318	0
acpP	0.0932426	-3.4228669	1.25E-25	2.92E-24	-1	0.13133367	-2.9286912	3.48E-16	9.51E-15	-1	1.40851575	0.49417569	0.658057	0.93664426	0
fabF	0.14112143	-2.824991	4.65E-18	6.23E-17	-1	0.26662693	-1.9071056	3.24E-07	2.12E-06	-1	1.88934398	0.91788539	0.01274177	0.11149052	0
aceE	0.07762082	-3.6874125	1.37E-28	3.92E-27	-1	0.09681346	-3.3686486	1.80E-17	6.26E-16	-1	1.24726142	0.31876388	0.39542027	0.86854717	0
pabC	0.95056286	-0.0731461	0.75249464	0.79898289	0	1.25599604	0.32883191	0.42798466	0.51872155	0	1.32131824	0.40197798	0.30398219	0.80892599	0
yceG	1.08758391	0.12112671	0.66340673	0.71850795	0	1.58614742	0.66552686	0.18387584	0.26084986	0	1.45841384	0.54440015	0.29566837	0.79986628	0
tmk	1.23286696	0.30201712	0.45116623	0.52140274	0	1.42373518	0.50968083	0.1960819	0.27533711	0	1.15481656	0.20766371	0.51178293	0.90925684	0
holB	1.2234005	0.29089677	0.45745849	0.52743257	0	1.0336148	0.04769864	0.89428514	0.91924774	0	0.84487035	-0.2431981	0.59760278	0.92806272	0
ycfH	1.11501626	0.15706475	0.70953849	0.75921306	0	0.72793811	-0.4581123	0.23283501	0.31765913	0	0.65284976	-0.6151771	0.11426587	0.50090704	0
ptsG	0.06848693	-3.8680274	2.20E-30	7.21E-29	-1	0.04723838	-4.4038967	1.03E-25	1.30E-23	-1	0.68974298	-0.5358692	0.11304067	0.49751433	0
fhuE	1.96739947	0.97628992	0.00724076	0.01334268	0	2.17576345	1.12152171	0.00421924	0.0097976	1	1.10590832	0.14523179	0.54620423	0.91914725	0
hinT	0.35587869	-1.4905425	3.46E-06	1.29E-05	-1	0.19552736	-2.3545576	7.22E-09	6.59E-08	-1	0.54942136	-0.8640151	0.01572619	0.12987356	0
ycfL	0.53659857	-0.8980849	0.00251077	0.00514143	0	0.29363123	-1.7679227	9.28E-06	4.40E-05	-1	0.54720837	-0.8698378	0.02320643	0.17358733	0
lpoB	0.42589245	-1.231439	0.00015702	0.00042111	-1	0.27119722	-1.8825857	4.49E-06	2.27E-05	-1	0.63677396	-0.6511468	0.0992559	0.458344	0
aceF	0.06000203	-4.0588449	3.11E-33	1.23E-31	-1	0.09280213	-3.4296983	6.98E-18	2.55E-16	-1	1.54664989	0.62914665	0.07157096	0.37176435	0
thiK	1.0398887	0.05642912	1	1	0	0.83750943	-0.2558227	0.83574267	0.87577588	0	0.80538372	-0.3122518	0.76064087	0.95840749	0
nagZ	0.36306751	-1.4616903	1.09E-05	3.70E-05	-1	0.2735157	-1.8703044	1.20E-05	5.52E-05	-1	0.75334668	-0.4086142	0.40034717	0.86854717	0
ycfP	0.1119662	-3.1588648	1.34E-19	1.92E-18	-1	0.07308836	-3.7742145	3.75E-18	1.47E-16	-1	0.65277165	-0.6153497	0.1263641	0.5319293	0
ndh	0.4570514	-1.1295717	0.00063666	0.00148477	-1	0.45089697	-1.1491303	0.00248395	0.00612532	-1	0.9865345	-0.0195586	0.83049916	0.97888613	0
ycfJ	22.806985	4.51140384	2.15E-10	1.43E-09	1	14.8060923	3.88811902	1.47E-05	6.58E-05	1	0.64919113	-0.6232848	0.19221727	0.66728286	0
ycfQ	0.47297354	-1.0801686	0.0064431	0.0119929	-1	0.49642586	-1.0103498	0.05036438	0.08649832	0	1.04958485	0.0698188	0.75161061	0.95683847	0
bhsA	0.53222623	-0.9098885	0.02159011	0.03566324	0	0.21149716	-2.2412898	0.0003691	0.00113032	-1	0.39738207	-1.3314013	0.04453571	0.27430512	0
ldtC	3.20762079	1.68150359	0.00029898	0.00074694	1	2.29165297	1.19638859	0.02018745	0.03890464	1	0.71444012	-0.485115	0.41619244	0.87131399	0
mfd	0.54304131	-0.8808661	0.00507369	0.00966387	0	0.61983072	-0.6900538	0.11347143	0.17296662	0	1.1414062	0.1908123	0.44052461	0.88087171	0
ycfT	4.37297863	2.1286163	1.23E-06	4.91E-06	1	3.98283614	1.99379613	7.81E-05	0.00028851	1	0.91078335	-0.1348202	0.87536133	0.98905525	0
lpd	0.16113475	-2.6336604	1.22E-16	1.43E-15	-1	0.1490799	-2.7458423	4.77E-13	8.10E-12	-1	0.92518777	-0.1121819	0.58323845	0.92806272	0
lolC	0.81210834	-0.3002559	0.37071652	0.4413625	0	0.98808106	-0.0172987	1	1	0	1.21668626	0.2829572	0.45312659	0.88755478	0
lolD	0.68983857	-0.5356693	0.26851673	0.33522848	0	0.70501855	-0.5042669	0.41573545	0.50654454	0	1.02200512	0.03140243	0.83744207	0.98067952	0
lolE	0.76427479	-0.3878367	0.32618825	0.39549094	0	0.85413501	-0.227464	0.67485403	0.74741352	0	1.1175758	0.16037269	0.65132962	0.9347567	0
nagK	0.68968713	-0.535986	0.08252127	0.11761744	0	0.70302176	-0.5083588	0.11667586	0.17723981	0	1.01933431	0.02762729	0.85747296	0.98347354	0
cobB	0.3326475	-1.5879339	2.16E-05	6.92E-05	-1	0.40853596	-1.291465	0.00169521	0.00439362	-1	1.22813477	0.29646888	0.58283628	0.92806272	0
ycfZ	7.57123488	2.92052862	0.00523723	0.00994107	1	4.7703114	2.25408345	0.02084472	0.04003165	1	0.63005725	-0.6664452	0.78112854	0.9635071	0
ymfA	7.81454791	2.96616241	7.51E-07	3.08E-06	1	6.36459657	2.67006907	1.12E-05	5.18E-05	1	0.81445487	-0.2960933	0.95618148	1	0
potD	0.06239747	-4.0023686	4.46E-29	1.34E-27	-1	0.10674571	-3.22775	5.29E-15	1.21E-13	-1	1.71073783	0.77461869	0.04964134	0.29478208	0
potC	0.45880859	-1.1240357	0.01242571	0.02177088	-1	0.4214497	-1.2465676	0.08705312	0.13902702	0	0.91857413	-0.1225319	0.69905861	0.94869712	0
potB	1.42137336	0.50728556	0.17570364	0.23070243	0	1.72540209	0.78693261	0.15771162	0.22932796	0	1.21389787	0.27964704	0.70483827	0.94896888	0
yacH	3.73828502	1.90237657	2.86E-07	1.24E-06	1	3.2714872	1.70994663	7.05E-05	0.00026294	1	0.87513049	-0.1924299	0.69084464	0.94569182	0
potA	0.54160545	-0.8846858	0.03407649	0.05394834	0	0.81418701	-0.2965679	0.53007968	0.61675147	0	1.50328439	0.58811796	0.21494612	0.69750727	0
pepT	0.1451334	-2.7845485	3.97E-17	4.91E-16	-1	0.16773177	-2.5757721	1.00E-12	1.61E-11	-1	1.15570758	0.20877641	0.76240915	0.95858924	0
roxA	0.47753301	-1.0663276	0.00090031	0.00204036	-1	0.42940447	-1.2195909	0.00149088	0.00393324	-1	0.8992142	-0.1532633	0.59521755	0.92806272	0
phoQ	1.0797903	0.1107508	0.77257838	0.81638246	0	0.67388377	-0.5694283	0.3280463	0.41742208	0	0.62408779	-0.6801791	0.18758356	0.6609229	0
phoP	0.46202704	-1.1139508	0.00024034	0.00061721	-1	0.21417068	-2.2231671	3.22E-08	2.58E-07	-1	0.46354577	-1.1092163	0.0035863	0.04321198	-1
purB	0.39346606	-1.3456889	9.93E-05	0.00027736	-1	0.47360837	-1.0782335	0.0104186	0.02180793	-1	1.20368292	0.2674554	0.47783698	0.89855925	0
hflD	0.6197426	-0.6902589	0.10847703	0.15010746	0	0.99185247	-0.0118026	1	1	0	1.60042647	0.67845639	0.14761848	0.58035154	0
mnmA	0.53711632	-0.8966935	0.00627879	0.01172659	0	1.03625542	0.05137964	0.89135877	0.91773402	0	1.92929422	0.94807317	0.01212595	0.10759642	0
nudJ	0.66515616	-0.588235	0.36224268	0.4330246	0	0.94504182	-0.0815499	0.69590613	0.76478716	0	1.42078188	0.50668509	0.6081222	0.92806272	0
rluE	1.2900968	0.36747932	0.28585774	0.3528881	0	1.63048189	0.70529842	0.10627983	0.16440262	0	1.26384461	0.3378191	0.42817891	0.87784805	0
acnB	0.22266777	-2.1670353	6.92E-12	5.34E-11	-1	0.25102232	-1.9941124	6.93E-08	5.29E-07	-1	1.12734018	0.17292291	0.7464734	0.95683847	0
icd	0.15050901	-2.7320783	6.79E-18	8.95E-17	-1	0.1049243	-3.2525792	2.47E-16	6.99E-15	-1	0.69712975	-0.5205009	0.14499956	0.57607933	0
ymfD	0.10212114	-3.2916465	3.69E-11	2.67E-10	-1	0.45785227	-1.1270459	0.01314017	0.02662376	-1	4.48342297	2.16460061	0.00049864	0.00960263	1
ymfE	0.23416291	-2.0944155	4.24E-07	1.80E-06	-1	0.42445658	-1.2363111	0.00158216	0.00413947	-1	1.81265505	0.8581044	0.30725194	0.81011621	0
lit	0.60542452	-0.723981	0.05874111	0.08750741	0	0.78150244	-0.3556777	0.24341136	0.3286388	0	1.2908338	0.36830326	0.77894807	0.9635071	0
intE	3.50129603	1.80788904	0.0006293	0.00146913	1	3.71066173	1.89167649	0.01593164	0.03152795	1	1.05979663	0.08378745	0.74892236	0.95683847	0
xisE	5.64949797	2.49812267	0.01075144	0.01908726	1	3.56220015	1.83276858	0.11930007	0.18060535	0	0.63053393	-0.6653541	0.54509182	0.91914725	0
ymfI	0.29252777	-1.7733545	1.26E-06	5.01E-06	-1	0.30254738	-1.724767	0.00017202	0.00057893	-1	1.03425182	0.0485875	1	1	0
ymfJ	4.6099332	2.20474585	0.00415598	0.00807255	1	3.01653128	1.59289054	0.15504699	0.22627234	0	0.65435466	-0.6118553	0.30990966	0.81158053	0
cohE	0.45798687	-1.1266219	0.0040405	0.00787278	-1	0.77538297	-0.3670191	0.19282308	0.27162423	0	1.69302446	0.75960282	0.28721639	0.78998378	0
croE	1.30839725	0.38780063	0.60827156	0.66967484	0	2.9020981	1.53709629	0.26395878	0.35088209	0	2.2180558	1.14929566	0.43657795	0.88087171	0
yacL	0.51923079	-0.9455522	0.00150672	0.00325597	0	0.2664389	-1.9081234	7.68E-07	4.64E-06	-1	0.51314156	-0.9625712	0.00655542	0.0685057	0
ymfL	3.35252113	1.74524642	0.03900504	0.06105885	0	6.2488262	2.64358522	0.00902576	0.0192216	1	1.86391852	0.89833879	0.36404586	0.85329623	0
ymfM	2.29392072	1.19781553	0.10686118	0.14828961	0	5.51968507	2.46458595	0.00519266	0.0117734	1	2.40622312	1.26677043	0.13007953	0.53863917	0
oweE	7.42379883	2.89215762	0.00045416	0.00109084	1	10.036751	3.32722042	7.26E-05	0.00027036	1	1.35196969	0.43506281	0.37442179	0.85857675	0
aaaE	10.0122544	3.32369495	1.47E-14	1.45E-13	1	12.6065173	3.65609787	2.20E-13	3.98E-12	1	1.25910877	0.33240292	0.4833271	0.89935549	0
ymfR	14.1488892	3.82261689	5.29E-05	0.00015852	1	12.0920338	3.59598501	0.00015672	0.00053447	1	0.85462778	-0.2266319	0.91072905	0.99971546	0
beeE	7.5640074	2.91915077	5.38E-06	1.94E-05	1	10.8081363	3.43404586	2.58E-06	1.39E-05	1	1.42889023	0.51489509	0.38955344	0.86808018	0
jayE	10.3561566	3.37241679	4.60E-13	3.94E-12	1	16.0563996	4.00507652	3.47E-13	6.04E-12	1	1.55042069	0.63265973	0.2390401	0.73256904	0
ymfQ	8.06086357	3.01093441	3.74E-06	1.38E-05	1	13.7227996	3.77850293	3.50E-07	2.27E-06	1	1.70239819	0.76756852	0.22807212	0.71536133	0
stfP	9.49564069	3.24726534	3.14E-10	2.05E-09	1	7.67540846	2.94024353	1.02E-06	6.03E-06	1	0.80830865	-0.3070218	0.5900401	0.92806272	0
tfaP	3.39273188	1.76244742	0.04573903	0.07034447	0	2.98327761	1.57689824	0.10378768	0.16166294	0	0.87931429	-0.1855492	0.97251037	1	0
speD	0.16849518	-2.5692208	1.16E-12	9.65E-12	-1	0.2072926	-2.2702594	8.87E-07	5.28E-06	-1	1.23025837	0.29896133	0.26482783	0.76482694	0
tfaE	1.52856447	0.6121774	0.33316589	0.40273501	0	2.4447862	1.28970831	0.12558512	0.18850566	0	1.59940013	0.67753091	0.42917016	0.87784805	0
stfE	10.5982804	3.4057583	1.77E-05	5.75E-05	1	7.43990729	2.89528464	0.00150855	0.00396803	1	0.70199193	-0.5104737	0.60907352	0.92806272	0
pinE	7.91365766	2.98434466	3.73E-09	2.12E-08	1	7.84536182	2.97183998	2.30E-07	1.57E-06	1	0.99136988	-0.0125047	0.93799182	1	0
mcrA	3.62695191	1.85875762	9.35E-05	0.00026213	1	3.91502562	1.96902175	0.00111348	0.00304863	1	1.07942584	0.11026413	1	1	0
icdC	21.3424797	4.4156559	4.51E-08	2.19E-07	1	6.37128512	2.6715844	0.00922416	0.01957808	1	0.298526	-1.7440715	0.03112946	0.21551164	0
iraM	7.07045532	2.82180312	9.78E-05	0.00027374	1	4.40579322	2.13940178	0.00820026	0.01760784	1	0.62312723	-0.6824013	0.45453305	0.88755478	0

ycgX	2.99353388	1.5818496	0.07176637	0.10396591	0	1.42069095	0.50659275	0.36767867	0.45909125	0	0.47458656	-1.0752568	0.53539887	0.9187668	0
bluR	0.65986984	-0.5997466	0.07998464	0.11437104	0	0.92725854	-0.1089564	0.51670914	0.60421744	0	1.40521432	0.49079018	0.40824967	0.86959974	0
bluF	4.11549176	2.04106483	8.88E-06	3.07E-05	1	1.98629041	0.99007657	0.04387572	0.07700032	0	0.48263744	-1.0509883	0.07385051	0.37957754	0
ycgZ	4.56133735	2.18945687	0.02699526	0.04367695	1	1.37879044	0.4634032	0.39094918	0.48162934	0	0.30227767	-1.7260537	0.33366992	0.83424743	0
speE	0.4897625	-1.0298458	0.00133354	0.00291307	-1	0.53594664	-0.8998441	0.00340041	0.00810048	0	1.09429497	0.13000167	0.73610176	0.95683847	0
ymgA	1.73716205	0.79673234	0.41288042	0.48618843	0	2.01093884	1.0078692	0.2409629	0.32594484	0	1.15760003	0.21113686	0.63616363	0.93163024	0
ariR	2.88148593	1.52681297	0.26974209	0.33622936	0	1.12788302	0.17361745	0.5854735	0.66783877	0	0.3914241	-1.3531955	0.68536502	0.943596	0
ymgC	1.17364864	0.23100057	0.95752697	0.96966412	0	1.06361787	0.08897993	0.67266132	0.74521491	0	0.90624897	-0.1420206	0.73057704	0.9551867	0
ycgG	8.74298506	3.12812593	2.03E-12	1.65E-11	1	8.16667043	3.02974801	4.00E-10	4.52E-09	1	0.93408262	-0.0983779	0.96266666	1	0
ymgF	4.40331384	2.13858967	0.00308047	0.00617913	1	3.7581478	1.91002181	0.0702354	0.11598289	0	0.8534817	-0.2285679	0.61030734	0.92872856	0
ycgH	7.23087831	2.8541709	1.93E-13	1.71E-12	1	7.47791338	2.90263576	1.70E-10	2.02E-09	1	1.03416391	0.04846486	0.93464653	1	0
ymgD	1.28369347	0.36030075	0.49675734	0.56623926	0	1.30788781	0.3872388	0.43628294	0.52675801	0	1.01884745	0.02693805	0.83075838	0.97888613	0
ymgG	1.18339396	0.24293044	0.5721862	0.63677159	0	2.60571669	1.38168023	0.03338009	0.0608024	0	2.20190128	1.13874979	0.08265338	0.40863385	0
ymgI	0.66680307	-0.5846673	0.89266563	0.91695444	0	0.43526336	-1.2000395	0.94529458	0.96266899	0	0.65276147	-0.6153722	1	1	0
ymgJ	9.76801491	3.2880654	0.01029982	0.01836669	1	2.38740517	1.25544343	0.17181809	0.24699549	0	0.24441048	-2.032622	0.32745493	0.82802536	0
yacC	1.11727831	0.1599886	0.83890596	0.87105022	0	2.53836044	1.34389694	0.20897568	0.28985044	0	2.27191418	1.18390834	0.16058213	0.60475422	0
ycgI	7.0711763	2.82195023	4.19E-05	0.00012759	1	8.8427205	3.14449029	5.37E-05	0.0002076	1	1.25053034	0.32254006	0.58776448	0.92806272	0
minE	0.06812111	-3.8757543	1.23E-25	2.89E-24	-1	0.05499737	-4.1844936	8.22E-19	3.52E-17	-1	0.807347	-0.3087392	0.64348517	0.93208609	0
minD	0.09722175	-3.3625771	1.90E-23	3.60E-22	-1	0.10430998	-3.2610509	2.55E-16	7.18E-15	-1	1.07290787	0.1015262	1	1	0
minC	0.25353618	-1.9797364	1.81E-09	1.06E-08	-1	0.33485528	-1.5783904	4.05E-05	0.00016205	-1	1.32073959	0.40134604	0.34323826	0.84269271	0
ycgJ	0.37510996	-1.4146145	0.00287123	0.00581754	-1	0.26161508	-1.9344824	0.00260552	0.00637302	-1	0.69743572	-0.5198678	0.50875134	0.90925684	0
pliG	0.23769728	-2.0728027	6.40E-08	3.04E-07	-1	0.25382724	-1.9780812	4.74E-05	0.00018602	-1	1.06785925	0.0947215	0.73648834	0.95683847	0
ycgL	0.46498732	-1.1047367	0.00439727	0.00848525	-1	0.55958533	-0.83757	0.01855501	0.03615282	0	1.20344213	0.26716676	1	1	0
ycgM	0.37879475	-1.4005118	3.62E-05	0.00011136	-1	0.49697277	-1.0087613	0.00759441	0.01654002	-1	1.31198431	0.39175047	0.47535645	0.89855206	0
ycgN	1.46962613	0.55544918	0.17832331	0.23351736	0	1.11945038	0.16279059	0.72545275	0.78990674	0	0.7617246	-0.3926586	0.44055984	0.88087171	0
hlyE	1.18155999	0.24069288	0.61973761	0.68009467	0	0.95364663	-0.0684733	1	1	0	0.80710809	-0.3091662	0.67333106	0.93997784	0
dnaK	0.28229006	-1.8247498	2.29E-09	1.33E-08	-1	0.30342915	-1.7205684	1.16E-06	6.76E-06	-1	1.07488429	0.10418137	0.98560624	1	0
cueO	1.41311832	0.49888227	0.05929071	0.08808848	0	1.65588177	0.72759967	0.03583024	0.06465245	0	1.17179272	0.22871739	0.50955118	0.90925684	0
umuD	33.5004926	5.0661104	5.71E-05	0.00016943	1	46.4733594	5.53833203	0.00037091	0.00113428	1	1.38724406	0.47222163	0.97526161	1	0
umuC	11.8151445	3.56256536	3.95E-12	3.09E-11	1	14.1777486	3.82555655	9.04E-10	9.61E-09	1	1.19996405	0.26299119	0.88805006	0.99411016	0
dsbB	0.7258637	-0.4622294	0.08870127	0.12529374	0	0.88575677	-0.1750175	0.51031175	0.59757012	0	1.22027974	0.28721191	0.53475281	0.9187668	0
nhaB	0.28413039	-1.815375	1.75E-06	6.84E-06	-1	0.38273467	-1.3855835	0.00206644	0.00522105	-1	1.34703883	0.42979144	0.26088836	0.75954558	0
fadR	0.41966011	-1.2527067	0.00026831	0.00067878	-1	0.55119063	-0.8593767	0.01507249	0.03004299	0	1.31342153	0.39333002	0.52674998	0.91534998	0
ycgB	3.12703804	1.64479677	0.06630749	0.0971005	0	0.60829375	-0.7171599	0.48819804	0.57636846	0	0.19452713	-2.3619567	0.0270511	0.19524607	0
dadA	5.29319471	2.40413873	3.29E-09	1.88E-08	1	0.79807898	-0.3253966	0.58181411	0.66400738	0	0.15077454	-2.7295353	4.74E-08	4.35E-06	-1
dadX	7.38388458	2.88438	3.70E-18	5.00E-17	1	1.33316506	0.41485541	0.32190278	0.41243502	0	0.18055064	-2.4695246	3.03E-10	5.34E-08	-1
cvrA	1.10051425	0.13817782	0.61764327	0.67830192	0	1.21195163	0.27733212	0.38672622	0.4774918	0	1.10125938	0.1391543	0.63679007	0.93163024	0
ldcA	1.1304437	0.17688914	0.97553587	0.98332769	0	1.34248832	0.42490954	0.47657453	0.56612442	0	1.18757646	0.2480204	0.56667526	0.92591251	0
gcd	5.25041039	2.39243019	1.01E-05	3.44E-05	1	2.41826587	1.27397287	0.01499554	0.0299166	1	0.46058607	-1.1184573	0.14001872	0.56339648	0
emtA	0.68611941	-0.5434684	0.16699647	0.22084533	0	0.94148655	-0.0869876	0.88169878	0.91097192	0	1.37219052	0.4564808	0.31842033	0.81858662	0
ycgR	4.60419116	2.20294773	0.00018998	0.00049949	1	4.23998214	2.08405819	0.00222091	0.00557625	1	0.9208962	-0.1188895	0.92840769	1	0
ymgE	3.04342789	1.60569718	0.00040609	0.0009861	1	1.29232493	0.36996885	0.3381561	0.42866087	0	0.42462808	-1.2357283	0.04065787	0.25948079	0
ycgY	5.45215622	2.4468269	0.00069533	0.00161221	1	2.08833612	1.06235393	0.05739054	0.09664992	0	0.3830294	-1.384473	0.2215132	0.70531966	0
treA	1.68379896	0.75171989	0.05178883	0.07852773	0	0.88485109	-0.1764934	0.94612195	0.96267854	0	0.52550875	-0.9282133	0.05710876	0.32074131	0
dhaM	0.44663715	-1.1628249	0.00029093	0.00073097	-1	0.41996991	-1.2516421	0.00160582	0.00419145	-1	0.94029328	-0.0888173	0.7825268	0.9635071	0
dhaL	0.37573251	-1.4122222	5.93E-05	0.00017514	-1	0.27634051	-1.855481	0.00014698	0.00050663	-1	0.73547138	-0.4432589	0.53761265	0.9187668	0
dhaK	0.35313192	-1.5017209	3.59E-06	1.34E-05	-1	0.29335181	-1.7692962	5.20E-05	0.0002019	-1	0.8307145	-0.2675754	0.76417594	0.95937995	0
dhaR	1.12498642	0.16990758	0.52643422	0.59378446	0	1.14845497	0.1996943	0.29678268	0.38649735	0	1.02086119	0.02978671	0.59763681	0.92806272	0
ycgV	2.18141516	1.12526437	0.00084742	0.00192743	1	2.33335308	1.22240463	0.00099161	0.00274729	1	1.06965108	0.09714026	0.61457999	0.92897598	0
hpt	0.27727052	-1.8506339	3.01E-07	1.30E-06	-1	0.83913733	-0.2530212	0.30163119	0.39177861	0	3.02642101	1.5976127	0.00036637	0.00746166	1
ychF	0.23702517	-2.0768878	1.12E-09	6.78E-09	-1	0.25269546	-1.9845284	1.04E-05	4.85E-05	-1	1.06611232	0.09235944	0.49972418	0.90541644	0
pth	1.99711067	0.99791428	0.02445887	0.03993893	0	1.28781276	0.36492285	0.48263252	0.57132416	0	0.64483796	-0.6329914	0.20865907	0.69006731	0
ychH	0.08335032	-3.5846684	4.61E-26	1.11E-24	-1	0.04804548	-4.3794556	8.62E-27	1.31E-24	-1	0.57642818	-0.7947872	0.00319976	0.03952642	0
dauA	1.18863103	0.24930095	0.33940515	0.40875841	0	1.44336744	0.52943862	0.16282648	0.23606634	0	1.21431075	0.28013767	0.49187458	0.90539057	0
prs	0.13172229	-2.9244286	8.64E-18	1.13E-16	-1	0.49980887	-1.0005516	0.00326639	0.00780508	-1	3.79441382	1.92387703	1.07E-06	6.56E-05	1
ispE	0.31892474	-1.6487121	3.40E-06	1.27E-05	-1	0.47282493	-1.080622	0.00548769	0.01237881	-1	1.48255956	0.56809006	0.22550682	0.7094579	0
lolB	0.4575874	-1.1278808	0.00291586	0.00589445	-1	0.52540498	-0.9284982	0.03356443	0.06103755	0	1.14820684	0.19938256	0.70334781	0.94896888	0
hemA	0.58892432	-0.7638458	0.01544773	0.02644675	0	0.79880658	-0.3240819	0.31986449	0.41002247	0	1.35638239	0.43976396	0.30601953	0.81005169	0
prfA	0.63336683	-0.6588868	0.04661059	0.07151059	0	0.60739268	-0.7192986	0.08777371	0.13992497	0	0.95899035	-0.0604118	0.90825473	0.99960154	0
prmC	1.20266214	0.2662314	0.5169712	0.58512803	0	1.58501856	0.66449974	0.17520548	0.25071809	0	1.31792506	0.39826834	0.40280349	0.86854717	0
can	0.64589358	-0.6306316	0.05169846	0.07844456	0	0.5786912	-0.7891344	0.02298101	0.04354764	0	0.8959544	-0.1585028	0.45562056	0.88755478	0
ychQ	0.41230116	-1.2782296	0.00965375	0.01728359	-1	0.54075336	-0.8869574	0.19689057	0.2760257	0	1.31154943	0.39127219	0.42187003	0.87427012	0
ychA	0.307367	-1.7019658	1.23E-06	4.91E-06	-1	0.35143651	-1.508664	0.00012495	0.00043717	-1	1.14337748	0.19330178	0.93257269	1	0
kdsA	0.218795	-2.1923483	1.91E-11	1.41E-10	-1	0.27410856	-1.8671807	1.60E-07	1.13E-06	-1	1.25280997	0.3251676	0.76558729	0.96052346	0
rdlA	0.4184522	-1.2568652	0.13518957	0.18276337	0	0.30028689	-1.7355866	0.1752587	0.25071809	0	0.71761335	-0.4787214	0.78741198	0.96350911	0
ldrB	0.50784855	-0.9775298	1	1	0	0	#NAME?	1	1	0	0	#NAME?	1	1	0
rdlC	3.46151693	1.7914044	0.35563596	0.4262806	0	0.31741223	-1.6555704	0.99582857	1	0	0.09169744	-3.4469748	0.49892138	0.90539057	0
chaA	0.9579875	-0.0619213	0.95707201	0.96958419	0	1.23481113	0.30429039	0.71574834	0.78134681	0	1.28896372	0.36621166	0.6772394	0.94128448	0
yadG	0.65822021	-0.6033578	0.05897251	0.08779547	0	0.61103769	-0.7106667	0.20084409	0.28015468	0	0.92831803	-0.107309	0.83877896	0.98067952	0
chaB	1.54699638	0.62946982	0.2558016	0.32071407	0	0.50636412	-0.9817529	0.08542674	0.13717324	0	0.32732082	-1.6112227	0.00675859	0.06996568	0
chaC	2.31491714	1.21096056	0.0023375	0.00482464	1	1.10982887	0.15033724	0.74631992	0.80419111	0	0.47942488	-1.0606233	0.02247161	0.17027457	0
ychN	0.12909337	-2.9535132	6.81E-18	8.96E-17	-1	0.08260912	-3.5975552	4.64E-16	1.23E-14	-1	0.63991757	-0.644042	0.16666288	0.61557317	0
ychO	1.46649878	0.55237587	0.15478682	0.20624235	0	1.42268973	0.50862106	0.17452681	0.24983283	0	0.97012677	-0.0437548	0.90407976	0.99884485	0
narL	0.13224327	-2.9187338	2.78E-16	3.13E-15	-1	0.13872732	-2.8496762	5.51E-11	6.98E-10	-1	1.04903119	0.06905757	0.74526005	0.95683847	0
narX	0.20022928	-2.3202751	4.47E-11	3.14E-10	-1	0.2146236	-2.2201194	9.99E-07	5.89E-06	-1	1.07188918	0.10015576	0.47121432	0.89609968	0
narK	0.02444527	-5.354301	2.00E-48	1.88E-46	-1	0.06604602	-3.9203845	3.94E-22	2.90E-20	-1	2.70179186	1.43391654	0.00016286	0.00399016	1
narG	0.05621133	-4.1529952	1.87E-34	7.85E-33	-1	0.15532569	-2.6866316	9.94E-13	1.61E-11	-1	2.76324532	1.46636365	4.65E-05	0.00145348	1
narH	0.09165084	-3.447708	2.15E-25	4.89E-24	-1	0.41665067	-1.2630898	0.00018933	0.00062858	-1	4.54606469	2.18461821	3.48E-09	4.63E-07	1
narJ	0.14879292	-2.7486222	5.90E-14	5.48E-13	-1	0.6223322	-0.6842432	0.05099012	0.0873832	0	4.18253898	2.06437899	1.00E-06	6.33E-05	1
yadH	0.53481918	-0.9028769	0.01550777	0.02652898	0	0.60188785	-0.7324334	0.16387766	0.23741927	0	1.12540439	0.17044349	0.52748357	0.91582777	0
narI	0.24361426	-2.0373295	4.89E-09	2.73E-08	-1	0.73021881	-0.4535993	0.28811985	0.37761911	0	2.99743867	1.58373024	2.98E-05	0.00098019	1
rttR	1.11448627	0.15637884	0.60269018	0.66518804	0	1.19952695	0.26246557	0.5996594	0.68033245	0	1.07630483	0.10608673	0.85130143	0.98212032	0
tyrV	0.75307176	-0.4091408	0.64387537	0.70049199	0	1.68920993	0.75634863	0.77146505	0.82448656	0	2.24309293	1.16548939	0.47094134	0.89609045	0
tyrT	0.36898609	-1.4383617	0.0178057	0.03001824	-1	1.3571472	0.44057721	0.88320464	0.91217703	0	3.67804435	1.87893888	0.02762224	0.1987179	0
purU	0.61062818	-0.7116339	0.10091259	0.14096487	0	0.80225818	-0.3178615	0.37464952	0.46583461	0	1.31382437	0.39377244	0.63705646	0.93163024	0
ychJ	0.32003221	-1.643711	5.65E-05	0.00016783	-1	0.37010075	-1.43401	0.00123763	0.00332873	-1	1.15644845	0.20970096	0.94307172	1	0
rssA	0.48309432	-1.0496232	0.00195783	0.00412386	-1	0.33202723	-1.5906265	7.89E-05	0.00029116	-1	0.68729276	-0.5410033	0.12645713	0.5319293	0
rssB	0.88368187	-0.178401	0.4352569	0.5065734	0	0.36524006	-1.4530831	0.00450303	0.0103586	-1	0.41331622	-1.2746821	0.01468254	0.12380494	0
galU	0.29396575	-1.76628	2.92E-08	1.46E-07	-1	0.1471234	-2.7649014	3.19E-11	4.17E-10	-1	0.50047803	-0.9986214	0.00776993	0.07769927	0
yadI	0.5513907	-0.8588531	0.03502239	0.05536647	0	0.61604636	-0.6988892	0.08618407	0.13798807	0	1.11725924	0.15996397	0.96960706	1	0
hns	0.07787768	-3.6826464	7.08E-29	2.09E-27	-1	0.03519787	-4.8283682	9.20E-32	2.54E-29	-1	0.4519635	-1.1457218	8.74E-05	0.00237616	-1
tdk	0.96924295	-0.0450698	0.89704382	0.9201678	0	0.63103044	-0.6642185	0.24453887	0.33006025	0	0.65105497	-0.6191487	0.26574197	0.76696471	0
insZ	2.01720493	1.01235766	0.01274011	0.02225992	1	3.28507792	1.71592759	0.0054371	0.01227096	1	1.62852959	0.70356993	0.36375117	0.85329623	0
adhE	0.485798	-1.0415715	0.00024948	0.00063806	-1	0.29014684	-1.7851449	2.78E-07	1.86E-06	-1	0.59725821	-0.7435733	0.01527754	0.12688123	0
ychE	0.79334327	-0.3339829	0.22054787	0.28154912	0	1.09367691	0.12918661	0.93431511	0.95351928	0	1.37856708	0.46316947	0.32145809	0.82229128	0
oppA	1.33482016	0.41664538	0.64653963	0.70321732	0	0.44793186	-1.1586488	0.05095494	0.0873832	0	0.33557469	-1.5752942	0.01350946	0.11704657	0
oppB	3.09345978	1.62922128	1.62E-05	5.28E-05	1	1.84466309	0.88335734	0.05692707	0.09601554	0	0.59631068	-0.7458639	0.06602014	0.3524804	0
oppC	1.85193803	0.88903582	0.05490197	0.08256792	0	0.97591349	-0.0351748	0.88077522	0.91044578	0	0.52696876	-0.9242107	0.05197703	0.30506977	0
oppD	1.22124106	0.288348	0.563952	0.62935219	0	0.57986851	-0.7862023	0.07109006	0.11730656	0	0.47481904	-1.0745503	0.01279659	0.11152756	0
oppF	1.03237724	0.04597024	0.97665292	0.98422906	0	0.5582383	-0.841047	0.01655641	0.0325747	0	0.54073093	-0.8870172	0.01224434	0.10842878	0
yadE	2.35203833	1.23391157	0.00100242	0.00225107	1	2.29749944	1.20006451	0.00247476	0.00611288	1	0.97681207	-0.0338471	0.84624353	0.98157127	0
yciU	0.68916642	-0.5370757	0.32544998	0.39481256	0	1.44403466	0.53010537	0.90005478	0.92367121	0	2.09533521	1.06718106	0.35201117	0.84775072	0

clsA	0.50243875	-0.9929803	0.00103205	0.00230791	0	0.59208693	-0.7561191	0.01723603	0.0338215	0	1.17842608	0.23686126	0.79450957	0.96579786	0
yciY	0.58948059	-0.7624838	0.05523314	0.08291218	0	0.66954938	-0.5787376	0.16029538	0.2327786	0	1.13582939	0.18374615	0.95527716	1	0
kch	0.49538393	-1.013381	0.00624729	0.01168259	-1	0.68298226	-0.55008	0.12926078	0.19330063	0	1.3786928	0.46330103	0.46576219	0.89266026	0
yciI	0.0980389	-3.3505019	6.56E-20	9.56E-19	-1	0.14420504	-2.7938065	2.89E-12	4.39E-11	-1	1.47089616	0.5566954	0.54115381	0.91914725	0
tonB	0.92527959	-0.1120387	0.55612524	0.62250512	0	0.78198784	-0.3547819	0.49594317	0.58348585	0	0.84513681	-0.2427432	0.74628696	0.95683847	0
yciA	0.68405693	-0.5478117	0.14693582	0.19708943	0	1.46691783	0.55278806	0.66291615	0.73686525	0	2.14443823	1.10059976	0.10404365	0.47400052	0
yciB	0.57537732	-0.7974197	0.05325821	0.08036949	0	1.01212589	0.01738875	0.73223748	0.79463591	0	1.75906463	0.81480849	0.18927082	0.66370038	0
yciC	0.79585792	-0.3294172	0.3917021	0.46372237	0	1.51381953	0.59819323	0.62867696	0.70712975	0	1.90212285	0.92761043	0.2286141	0.71633217	0
ompW	0.05659251	-4.1432452	5.85E-27	1.50E-25	-1	0.10851452	-3.20404	3.52E-12	5.22E-11	-1	1.91747158	0.93920519	0.00664233	0.06906944	0
panD	0.35242614	-1.5046072	1.59E-05	5.19E-05	-1	0.59577329	-0.7471647	0.02159919	0.04137439	0	1.6904912	0.75744251	0.18995734	0.66370038	0
yciE	0.90870554	-0.1381152	0.90798741	0.92859004	0	0.63734574	-0.6498519	0.19149329	0.27015965	0	0.70137763	-0.5117367	0.23015875	0.71837124	0
yciF	3.50667145	1.81010227	0.02971378	0.04755282	1	1.30839855	0.38780206	0.43174743	0.52271011	0	0.37311695	-1.4223002	0.19816484	0.67679424	0
yciG	6.17093705	2.62548958	0.14725762	0.19746111	0	1.65059451	0.72298575	0.64649344	0.72325431	0	0.26747875	-1.9025038	0.42311565	0.87479608	0
trpA	2.41139068	1.26986541	0.00074108	0.0017156	1	0.80206914	-0.3182015	0.7287225	0.79253185	0	0.33261684	-1.5880669	0.00099107	0.01661826	-1
trpB	2.83506227	1.50338042	0.00562604	0.01061975	1	1.00396713	0.00571204	0.78861924	0.83852464	0	0.35412525	-1.4976684	0.02952997	0.2076988	0
trpC	4.97593041	2.31496631	7.49E-10	4.65E-09	1	1.49892966	0.58393269	0.14495641	0.21373452	0	0.30123606	-1.7310336	0.00013328	0.00335878	-1
trpD	9.73013067	3.28245918	3.65E-18	4.94E-17	1	4.39070317	2.134452	3.21E-08	2.58E-07	1	0.45124812	-1.1480072	0.01915864	0.15007035	0
trpE	22.9635244	4.52127218	1.58E-28	4.47E-27	1	8.89614809	3.1531808	7.00E-14	1.38E-12	1	0.38740343	-1.3680914	0.0042545	0.04860707	-1
trpL	6.59944673	2.72234508	0.06367488	0.09387581	0	2.89805578	1.53508536	0.54654439	0.63092468	0	0.43913617	-1.1872597	0.39861082	0.86854717	0
yciV	1.24410829	0.31511207	0.77580899	0.8190125	0	2.27473283	1.18569711	0.31851705	0.40868956	0	1.82840421	0.87058504	0.43028503	0.87809207	0
yadD	1.86374051	0.898201	0.02257149	0.0371454	0	2.63551222	1.39808338	0.01141131	0.023619	1	1.41409827	0.49988238	0.4498399	0.88755478	0
yciO	0.46409569	-1.1075058	0.00098502	0.00221417	-1	0.42869515	-1.221976	0.00035292	0.0010868	-1	0.92372148	-0.1144702	0.3173531	0.81786403	0
yciQ	2.68651279	1.4257347	0.00010688	0.00029672	1	1.83969685	0.87946805	0.01962754	0.03799129	0	0.68478991	-0.5462667	0.30039741	0.80487807	0
rluB	0.51610519	-0.954263	0.01276579	0.02229596	0	0.94565161	-0.0806193	0.90245057	0.92527356	0	1.83228464	0.87364364	0.03994199	0.25602349	0
btuR	0.20736193	-2.269777	1.09E-06	4.40E-06	-1	0.2331224	-2.1008404	0.00036152	0.00110863	-1	1.1242295	0.16893657	0.49710582	0.90539057	0
yciK	0.34811545	-1.5223623	9.34E-05	0.00026199	-1	0.26605142	-1.910223	6.14E-05	0.00023301	-1	0.76426203	-0.3878607	0.43195221	0.88005519	0
sohB	0.516023	-0.9544927	0.00155646	0.00335359	0	0.46828327	-1.0945466	0.00430931	0.00996485	-1	0.90748526	-0.1400539	0.69960915	0.94869712	0
yciN	0.08706185	-3.5218155	3.59E-22	6.25E-21	-1	0.10741422	-3.2187432	2.70E-15	6.64E-14	-1	1.23376907	0.30307238	1	1	0
topA	0.30310384	-1.722116	1.21E-07	5.49E-07	-1	0.48586141	-1.0413832	0.0039981	0.00932819	-1	1.60295365	0.68073271	0.09246766	0.44228024	0
cysB	0.38574334	-1.3742868	8.57E-05	0.0002431	-1	0.45902678	-1.1233498	0.00485573	0.01107198	-1	1.1899798	0.25093709	0.61352517	0.92897598	0
ymiA	0.4982128	-1.005166	0.27190538	0.33821629	0	1.0747016	0.10393613	0.68070588	0.75219787	0	2.15711358	1.10910214	0.59760518	0.92806272	0
dnaJ	1.25028343	0.32225518	0.41457192	0.48766023	0	1.37012082	0.45430312	0.32777158	0.41741285	0	1.09584818	0.13204794	0.79399422	0.96579786	0
panC	0.22575978	-2.1471396	6.32E-11	4.40E-10	-1	0.23349063	-2.0985635	1.82E-07	1.27E-06	-1	1.03424367	0.04857613	0.82729657	0.97733133	0
yciX	0.61421386	-0.703187	0.26907297	0.33573313	0	0.7463234	-0.4221272	0.39139494	0.4820441	0	1.21508719	0.28105984	1	1	0
acnA	10.6928054	3.41856851	3.16E-09	1.81E-08	1	1.0932763	0.12865805	0.93577913	0.95479288	0	0.1022441	-3.2899105	1.68E-06	9.16E-05	-1
ribA	0.71394062	-0.486124	0.10013903	0.14015022	0	0.35464958	-1.4955339	0.00012435	0.00043543	-1	0.49674941	-1.0094098	0.00683993	0.07037554	0
pgpB	60.8495424	5.92717451	2.40E-16	2.72E-15	1	30.7065874	4.94047628	7.59E-09	6.90E-08	1	0.50463136	-0.9866982	0.0584762	0.32643043	0
yciS	202.514552	7.66188177	2.86E-13	2.49E-12	1	154.910673	7.27529273	5.45E-10	6.02E-09	1	0.76493601	-0.386589	0.42077949	0.87375001	0
yciM	2395.97133	11.2263949	2.32E-27	6.06E-26	1	1496.71603	10.5475848	5.34E-22	3.87E-20	1	0.62468028	-0.6788101	0.19961625	0.67679424	0
pyrF	1736.3203	10.7618174	1.02E-39	6.29E-38	1	807.199427	9.65678134	1.75E-29	3.23E-27	1	0.46489085	-1.1050361	0.02734994	0.19708045	0
yciH	1832.41152	10.8395278	7.75E-41	5.35E-39	1	754.986844	9.5603077	1.23E-29	2.37E-27	1	0.41201817	-1.2792201	0.01377397	0.11863905	0
osmB	29.0921897	4.86255998	1.22E-12	1.01E-11	1	7.66620413	2.93851241	0.00114075	0.00310979	1	0.26351417	-1.9240476	0.0021187	0.02994491	-1
yciT	1.6297605	0.70465997	0.08869655	0.12529374	0	0.87434587	-0.193724	0.18721224	0.26524234	0	0.53648734	-0.898384	0.00562286	0.06048007	0
panB	0.16116305	-2.633407	1.00E-14	9.97E-14	-1	0.24669197	-2.0192173	5.61E-07	3.46E-06	-1	1.53069803	0.6141897	0.08903204	0.42947991	0
yciZ	21.3311386	4.41488907	4.92E-26	1.17E-24	1	6.83728687	2.77342396	1.43E-06	8.17E-06	1	0.3205308	-1.6414651	1.05E-05	0.00043367	-1
gmr	6.96304902	2.79971918	8.49E-13	7.16E-12	1	2.61891713	1.38897041	0.02258882	0.04289643	1	0.37611643	-1.4107488	0.00017248	0.00415739	-1
ymiB	7.19648352	2.84729212	0.00018062	0.00047687	1	0.61747476	-0.6955479	0.24295642	0.32822513	0	0.08580229	-3.54284	8.46E-05	0.00233297	-1
rnb	0.51719264	-0.9512263	0.00304612	0.00612693	0	0.85004678	-0.2343859	0.58853853	0.67003696	0	1.64357863	0.71684048	0.04882493	0.2937489	0
yciW	3.34534409	1.74215461	2.08E-05	6.67E-05	1	3.44482889	1.78443233	8.41E-05	0.00030786	1	1.02973829	0.04227772	0.77202933	0.9625811	0
fabI	0.18133317	-2.4632853	1.64E-14	1.60E-13	-1	0.19581249	-2.3524553	2.46E-10	2.83E-09	-1	1.07984929	0.11082997	1	1	0
ycjD	1.21391335	0.27966545	0.73798286	0.78622053	0	1.34458939	0.42716567	0.32736049	0.41712976	0	1.10764857	0.14750023	0.5513496	0.92038542	0
sapF	0.60710699	-0.7199773	0.030258	0.0483362	0	0.38214195	-1.3878194	0.0034239	0.00815204	-1	0.62944746	-0.6678421	0.17703413	0.63784355	0
sapD	0.78789476	-0.3439252	0.26731771	0.33382593	0	0.51206902	-0.9655898	0.03402547	0.06177426	0	0.64992059	-0.6216646	0.18302854	0.65210078	0
sapC	0.86109208	-0.2157606	0.63754919	0.69515052	0	0.846686	-0.2401011	0.76293848	0.81829081	0	0.98326999	-0.0243405	0.93665101	1	0
yadC	4.16451273	2.0581477	0.00933068	0.01678762	1	2.83507567	1.50338724	0.05241069	0.08945649	0	0.68077008	-0.5547605	0.72145226	0.95399541	0
sapB	0.8374429	-0.2559373	0.48433821	0.55387927	0	0.5475457	-0.8689487	0.1463835	0.21512094	0	0.65383049	-0.6130614	0.33865475	0.83714543	0
sapA	0.88802136	-0.1713337	0.71512464	0.76428438	0	0.87036439	-0.2003086	0.66809662	0.74122997	0	0.9801165	-0.0289749	0.92941207	1	0
ymjA	2.06494539	1.04610363	0.11833172	0.16226986	0	4.70029176	2.23275031	0.01409121	0.02822714	1	2.27623054	1.18664668	0.20526093	0.68468503	0
puuP	6.92491556	2.79179648	4.29E-12	3.35E-11	1	5.95039788	2.57298614	7.35E-09	6.70E-08	1	0.85927371	-0.2188103	0.79310092	0.96579786	0
puuA	2.98998052	1.58013608	0.00034013	0.00084024	1	2.49208075	1.31735081	0.00299275	0.00720706	1	0.83347725	-0.2627853	0.92107745	1	0
puuD	3.30100329	1.72290458	3.92E-05	0.00012013	1	2.53593393	1.34251716	0.01448558	0.0289515	1	0.76823126	-0.3803874	0.36665255	0.85342842	0
puuR	1.89205088	0.91995088	0.0413431	0.06429033	0	1.89823233	0.92465658	0.08740114	0.13943164	0	1.00326707	0.0047057	1	1	0
puuC	4.73652206	2.2438281	0.00029686	0.00074249	1	4.56402381	2.19030632	0.00064298	0.00185346	1	0.96358124	-0.0535218	0.7892165	0.96411212	0
puuB	4.2898121	2.10091446	0.14130738	0.19028963	0	3.7429089	1.90415994	0.10022891	0.15689393	0	0.87251115	-0.1967545	0.80469777	0.96990832	0
puuE	1.90433597	0.92928803	0.06801755	0.09915298	0	2.27468722	1.18566818	0.01152288	0.02378892	1	1.19447789	0.25638015	0.3556463	0.8482424	0
yadK	3.91553322	1.96920879	0.03858453	0.06045685	0	5.87653783	2.55496644	0.0252072	0.04729963	1	1.50082696	0.58575765	0.60527542	0.92806272	0
pspF	0.29131403	-1.7793529	3.09E-05	9.61E-05	-1	0.30366406	-1.7194519	0.00057324	0.00166875	-1	1.04239424	0.05990102	1	1	0
pspA	1.16354635	0.21852868	0.71785217	0.76680847	0	0.62469016	-0.6787873	0.01344131	0.02707253	0	0.53688464	-0.897316	0.00408355	0.04739064	0
pspB	1.7353157	0.79519815	0.16737881	0.22115232	0	1.00384794	0.00554075	0.50873747	0.59631589	0	0.57848145	-0.7896574	0.07486981	0.38291737	0
pspC	1.67539517	0.74450142	0.39670918	0.46889603	0	1.18388492	0.24352885	0.90706835	0.92936124	0	0.70663026	-0.5009726	0.37062456	0.85484012	0
pspD	1.4267199	0.51270213	0.43917742	0.50994918	0	1.17884715	0.23737667	0.8402991	0.8795278	0	0.8262639	-0.2753255	0.64705533	0.93208609	0
pspE	0.06707686	-3.897998	2.86E-28	8.05E-27	-1	0.15547753	-2.685222	1.19E-13	2.25E-12	-1	2.31783214	1.21277609	0.03088418	0.21452268	0
ycjM	1.02895767	0.04118364	0.79374395	0.83356325	0	0.84912184	-0.2359565	0.91732572	0.93878239	0	0.82522524	-0.2771401	0.95501113	1	0
ycjN	1.82170344	0.86528812	0.09265119	0.13037283	0	1.46999421	0.55581047	0.13537963	0.20129292	0	0.80693387	-0.3094776	0.9556374	1	0
ycjO	4.07575078	2.02706584	0.00336647	0.00668601	1	3.9175689	1.96995865	0.0026716	0.00652254	1	0.96118951	-0.0571072	0.71368401	0.95223057	0
ycjP	2.88279262	1.52746706	0.08024054	0.11466273	0	3.51373865	1.81300689	0.04550345	0.07944676	0	1.21886626	0.28553983	0.59799714	0.92806272	0
yadL	3.15638596	1.65827363	0.04358745	0.06743459	0	3.28983975	1.71801731	0.04202341	0.07428058	0	1.04228057	0.05974369	0.74098882	0.95683847	0
ycjQ	1.18655237	0.24677577	0.44117754	0.5117335	0	1.48890453	0.57425125	0.18196831	0.25836648	0	1.25481569	0.32747547	0.44105196	0.88087171	0
ycjR	1.65958201	0.73081992	0.23072365	0.29309933	0	1.75503468	0.81149954	0.10161882	0.15873226	0	1.05751609	0.08067962	0.56210273	0.92432422	0
ycjS	2.47738127	1.30881592	0.00116327	0.00257036	1	3.99552539	1.99838522	7.01E-05	0.00026202	1	1.61280196	0.6895693	0.16358904	0.61189794	0
ycjT	5.12963186	2.35885529	1.49E-07	6.72E-07	1	6.13124185	2.61617931	2.98E-07	1.97E-06	1	1.19525962	0.25732402	0.49411463	0.90539057	0
ycjU	6.20987524	2.63456428	2.54E-08	1.27E-07	1	11.7105178	3.54973296	6.35E-10	6.90E-09	1	1.88578954	0.91516867	0.10760097	0.48409953	0
ycjV	8.68243478	3.11809967	4.70E-07	1.98E-06	1	8.11621828	3.02080767	1.71E-05	7.47E-05	1	0.93478598	-0.097292	0.94185909	1	0
ompG	4.81320521	2.26699794	2.42E-05	7.59E-05	1	6.75091083	2.75508216	1.36E-05	6.16E-05	1	1.40258113	0.48808423	0.39917416	0.86854717	0
ycjW	1.27974464	0.35585596	0.47983316	0.54992815	0	2.3697405	1.24472909	0.01312549	0.02661605	1	1.85172919	0.88887312	0.05587914	0.31756056	0
ycjX	1.05643375	0.0792023	0.8842401	0.90935705	0	1.23604308	0.30572903	0.45033781	0.53989223	0	1.17001476	0.22652673	0.44212609	0.88145391	0
ycjF	1.21405246	0.27983076	0.37989848	0.45132103	0	1.37387801	0.45825391	0.23678455	0.32145958	0	1.13164633	0.17842315	0.62422153	0.92989404	0
yadM	3.28647852	1.71654256	0.01657952	0.02816606	1	2.1684387	1.11665666	0.09253139	0.14661034	0	0.65980614	-0.5998859	0.70256088	0.94896888	0
tyrR	0.15803337	-2.6616988	4.54E-15	4.65E-14	-1	0.16812469	-2.5723965	1.86E-10	2.20E-09	-1	1.06385563	0.08930238	0.86154562	0.98424873	0
tpx	0.15245753	-2.7135207	9.48E-18	1.23E-16	-1	0.16329428	-2.6144539	9.66E-12	1.34E-10	-1	1.07108042	0.09906681	0.80857137	0.96990832	0
ycjG	0.53049381	-0.9145922	0.0125091	0.02189961	0	1.53400048	0.61729893	0.13577049	0.20173911	0	2.89164634	1.53189111	0.00015036	0.00374619	1
mpaA	0.7222787	-0.4693725	0.16407115	0.21730202	0	0.72221624	-0.4694972	0.2454133	0.33103828	0	0.99991352	-0.0001248	0.97739236	1	0
ymjC	1.01376907	0.01972906	0.79674508	0.83572145	0	2.69114229	1.42821868	0.30584243	0.39599112	0	2.65459104	1.40848962	0.34503087	0.84269271	0
ymjD	0.16407362	-2.6075848	0.71900756	0.76785697	0	0.50604996	-0.9826483	1	1	0	3.08428593	1.62493652	0.66707316	0.93861876	0
ycjY	2.17180715	1.118896	0.01471237	0.02531551	1	1.63806787	0.71199513	0.16711228	0.24156663	0	0.75424186	-0.4069009	0.49269778	0.90539057	0
pgrR	1.40883764	0.49450536	0.26671178	0.33329409	0	1.36688588	0.4508928	0.27174332	0.35985428	0	0.97022243	-0.0436126	0.89268802	0.99488354	0
mppA	0.29315984	-1.7702406	9.14E-07	3.70E-06	-1	0.32223516	-1.6338142	0.00022625	0.00073839	-1	1.09917909	0.13642646	0.61759969	0.92897598	0
ynaI	1.14979587	0.20137776	0.82150431	0.85715491	0	0.94422614	-0.0827957	1	1	0	0.82121198	-0.2841734	0.78446898	0.9635071	0
htrE	3.96249055	1.9864075	0.0044623	0.00859197	1	3.68457848	1.88149958	0.00785959	0.01702526	1	0.92986429	-0.1049079	0.84669603	0.98157978	0
insH1	0	#NAME?	1	1	0	6.29618563	2.65447808	0.73898333	0.79881412	0	Inf	Inf	0.72299474	0.95399541	0
ynaJ	0.49920394	-1.0022988	0.00229556	0.00474474	-1	0.45665196	-1.1308331	0.00123376	0.00332237	-1	0.91476033	-0.1285343	0.36377723	0.85329623	0
uspE	0.26370871	-1.9229829	2.89E-10	1.89E-09	-1	0.08122825	-3.6218746	1.25E-19	5.89E-18	-1	0.30802263	-1.6988918	1.78E-06	9.44E-05	-1

fnr	0.68577311	-0.5441968	0.17280391	0.22750368	0	1.12797222	0.17373154	0.88546316	0.91315326	0	1.64481838	0.71792829	0.30660521	0.81011621	0
ogt	1.26016037	0.33360735	0.67938385	0.73232392	0	1.67102084	0.74072973	0.17003986	0.24515698	0	1.32603824	0.40712238	0.34451715	0.84269271	0
abgT	3.626805	1.85869918	5.49E-05	0.00016351	1	4.00118805	2.00042844	0.0001398	0.00048452	1	1.10322668	0.14172926	0.70173469	0.94869712	0
abgB	3.02765836	1.59820242	7.79E-05	0.00022395	1	4.29640772	2.10313091	9.97E-06	4.68E-05	1	1.41905301	0.50492849	0.24811167	0.74826869	0
abgA	6.44997905	2.68929447	4.97E-10	3.16E-09	1	7.98808383	2.99784947	4.43E-10	4.96E-09	1	1.23846663	0.308555	0.36619219	0.85342842	0
abgR	1.04210231	0.05949692	0.81381463	0.84999272	0	1.50941053	0.59398525	0.25027099	0.33605211	0	1.44842836	0.53448833	0.30930633	0.81096368	0
mcaS	1.15884445	0.21268693	0.77728467	0.81982881	0	3.81602784	1.9320717	0.21269991	0.29427705	0	3.2929595	1.71938477	0.27620369	0.77880965	0
yadV	3.44588944	1.78487641	0.00834301	0.01517753	1	2.71358275	1.4401989	0.03692406	0.06638218	0	0.78748399	-0.3446775	0.8851503	0.99348376	0
smrA	5.74403834	2.52206538	2.85E-09	1.64E-08	1	4.24997898	2.0874557	0.00033887	0.0010501	1	0.73989391	-0.4346097	0.15877266	0.60153558	0
ydaM	16.0166435	4.00149994	1.48E-09	8.75E-09	1	9.20197407	3.20194339	0.00033198	0.00102948	1	0.57452575	-0.7995566	0.09666707	0.45507426	0
ydaN	0.9886186	-0.016514	0.90458068	0.92617823	0	0.87879635	-0.1863992	0.89590092	0.92069446	0	0.88891343	-0.1698852	0.83462421	0.98014902	0
fnrS	0.08019643	-3.6403181	1.64E-12	1.34E-11	-1	0.05358025	-4.2221549	1.13E-09	1.18E-08	-1	0.66811259	-0.5818369	0.54544163	0.91914725	0
dbpA	2.84616636	1.50901999	0.0001254	0.00034087	1	1.92182546	0.94247732	0.02082857	0.04001802	0	0.675233	-0.5665427	0.36504368	0.85342842	0
ttcA	0.67598686	-0.5649329	0.14438002	0.19401476	0	0.93693466	-0.0939797	0.82515729	0.86716267	0	1.38602495	0.47095323	0.30164449	0.80606277	0
intR	3.77309963	1.91575019	6.82E-06	2.41E-05	1	2.7772456	1.47365477	0.00149775	0.00394901	1	0.73606474	-0.4420954	0.4768371	0.89855925	0
ydaQ	7.02745581	2.81300248	0.01369313	0.02378393	1	2.13545674	1.09454467	0.26149894	0.34858637	0	0.30387338	-1.7184578	0.35871596	0.85183882	0
ydaC	30.3580329	4.92400641	0.19274352	0.25008232	0	9.49455069	3.24709973	0.38603147	0.4769005	0	0.3127525	-1.6769067	0.91236748	1	0
yadN	5.65689247	2.50000975	0.00668057	0.01240357	1	9.27439304	3.21325287	0.0016892	0.00438063	1	1.63948548	0.71324312	0.40060401	0.86854717	0
ralR	3.70118863	1.88798867	0.53042039	0.59790744	0	1.94298731	0.95827647	0.56636118	0.65023384	0	0.52496306	-0.9297122	0.97385796	1	0
recT	1.68818468	0.75547274	0.10085878	0.14093427	0	2.02629748	1.01884599	0.0399397	0.07113511	0	1.20028188	0.26337325	0.47378262	0.89828993	0
recE	4.80046439	2.26317398	0.00064163	0.00149555	1	5.11262906	2.35406536	0.00047626	0.00140492	1	1.06502801	0.09089138	0.61306746	0.92897598	0
racC	1.73807087	0.79748691	0.62538955	0.68561569	0	2.11643596	1.08163684	0.26556539	0.35265355	0	1.21769256	0.28414993	0.54694089	0.91914725	0
ydaE	4.68346422	2.22757604	0.37698549	0.44810143	0	2.8861759	1.52915923	0.48958873	0.57785593	0	0.61624809	-0.6984168	0.94219551	1	0
kilR	7.50399638	2.90765913	0.00257987	0.00527072	1	11.3218256	3.5010347	0.00205098	0.00518494	1	1.50877279	0.59337556	0.57838763	0.92806272	0
sieB	2.90835263	1.5402022	0.23498309	0.29765424	0	1.92079951	0.94170694	0.4812242	0.5702681	0	0.66044244	-0.5984953	0.7222991	0.95399541	0
ydaF	3.91699135	1.96974594	0.19287402	0.25009813	0	2.11678495	1.08187471	0.44842624	0.53789239	0	0.54041093	-0.8878712	0.73393996	0.95599369	0
ydaG	10.1874333	3.34871871	9.08E-05	0.00025523	1	6.76889185	2.75891967	0.00213289	0.00536746	1	0.66443545	-0.589799	0.66082125	0.93664426	0
racR	0.56504697	-0.8235573	0.20335582	0.26240802	0	0.92609839	-0.1107626	0.48433935	0.57273096	0	1.63897593	0.71279467	0.72352869	0.95417509	0
folK	1.58980787	0.66885242	0.25518691	0.32003424	0	2.9610316	1.56609989	0.01857197	0.0361699	1	1.86250909	0.89724746	0.15039579	0.58623767	0
ydaS	1.38197587	0.46673242	0.62936902	0.68875793	0	0.28415546	-1.8152477	0.30954432	0.40008083	0	0.20561535	-2.2819801	0.14949162	0.58444861	0
ydaT	1.84557766	0.88407244	0.38439932	0.45596289	0	2.33923865	1.22603905	0.19153885	0.27015965	0	1.26748319	0.34196661	0.60435213	0.92806272	0
ydaU	3.33316101	1.73689101	0.00720692	0.01328587	1	3.32388488	1.73287042	0.00322876	0.00772073	1	0.99721702	-0.0040206	0.6334005	0.93163024	0
ydaV	9.23662001	3.20736502	3.94E-05	0.0001205	1	7.04302146	2.81619448	0.00022407	0.00073184	1	0.76251069	-0.3911705	0.98090268	1	0
ydaW	2.70506926	1.43566553	0.04545425	0.06997958	0	2.57410713	1.3640721	0.07203306	0.11864111	0	0.9515864	-0.0715934	0.98546302	1	0
rzpR	2.30481048	1.20464812	0.6293331	0.68875793	0	3.69901644	1.88714171	0.19919755	0.27838519	0	1.60491133	0.68249359	0.45327148	0.88755478	0
trkG	1.69884234	0.76455197	0.08231117	0.11735586	0	1.30983107	0.38938076	0.35815073	0.44936629	0	0.77101391	-0.3751712	0.55985311	0.92432422	0
ynaK	7.53477892	2.91356518	0.00135029	0.00294239	1	5.45051804	2.44639336	0.00818401	0.01758149	1	0.72338128	-0.4671718	0.87385683	0.98838384	0
ydaY	6.07857377	2.60373286	4.22E-06	1.56E-05	1	7.16223635	2.84041013	4.69E-06	2.36E-05	1	1.1782758	0.23667727	0.55160289	0.92038542	0
pcnB	0.54728908	-0.869625	0.00754973	0.01388308	0	1.12939799	0.17555397	0.60116934	0.68169548	0	2.06362239	1.045179	0.00417244	0.04791786	1
tmpR	4.35200657	2.12168073	0.03344839	0.05297294	1	1.49986417	0.58483185	0.32558399	0.41570519	0	0.34463739	-1.5368489	0.32011849	0.82031222	0
lomR	2.59228956	1.37422688	0.01951287	0.0325239	1	3.6767757	1.87844117	0.00479905	0.01097103	1	1.4183507	0.50421429	0.37344906	0.85857675	0
insH1	1.10218247	0.14036309	0.77768891	0.82001717	0	0.69920646	-0.5162096	0.57056331	0.65454809	0	0.63438358	-0.6565727	0.39473518	0.86854717	0
stfR	2.78163095	1.47593102	0.00016206	0.00043219	1	5.03396977	2.33169655	6.88E-06	3.35E-05	1	1.80971878	0.85576553	0.13200445	0.54303016	0
ynaE	0	#NAME?	1	1	0	0	#NAME?	1	1	0	NA	NA	NA	NA	0
ttcC	1.77782757	0.8301154	0.83564486	0.86786817	0	0.73438545	-0.4453906	0.87959398	0.90986091	0	0.41308025	-1.275506	0.59402875	0.92806272	0
uspF	0.19612536	-2.350152	6.92E-13	5.88E-12	-1	0.12481647	-3.0021197	2.48E-14	5.12E-13	-1	0.6364117	-0.6519677	0.02029947	0.15732979	0
ompN	27.1459527	4.76266521	1.06E-27	2.83E-26	1	12.6613793	3.66236267	1.64E-13	3.05E-12	1	0.46641867	-1.1003025	0.00904625	0.08691492	0
gluQ	1.08542581	0.11826112	0.6390872	0.69620228	0	1.57920372	0.65919729	0.10904767	0.16772769	0	1.45491631	0.54093617	0.19905435	0.67679424	0
micC	5.45016388	2.44629961	0.23015324	0.29254298	0	3.36671439	1.75134134	0.28862803	0.3779103	0	0.61772719	-0.6949583	1	1	0
pfo	1.34273049	0.42516976	0.25046748	0.31500992	0	0.81326876	-0.2981959	0.31531962	0.40541094	0	0.6056828	-0.7233656	0.03667793	0.2400068	0
ydbJ	0.36176012	-1.4668947	8.46E-05	0.00024078	-1	0.55723171	-0.8436507	0.02373396	0.04482066	0	1.54033482	0.62324398	0.29456315	0.79886161	0
hslJ	0.43845141	-1.1895111	0.00056029	0.00132555	-1	0.59612987	-0.7463014	0.08899817	0.14172357	0	1.35962586	0.4432097	0.26704891	0.76922645	0
ldhA	0.60609403	-0.7223865	0.12696831	0.17271914	0	0.63204666	-0.661897	0.27607057	0.36416593	0	1.04281947	0.06048943	0.91108906	0.99972698	0
ydbH	1.0164743	0.02357374	0.78922611	0.83000279	0	1.19818805	0.26085435	0.49464879	0.58244285	0	1.17876866	0.23728061	0.58992848	0.92806272	0
ynbE	0.75430271	-0.4067845	0.68156925	0.73426619	0	2.02593085	1.01858493	0.49343024	0.58145819	0	2.68583267	1.42536942	0.26793815	0.76966758	0
ydbL	3.53807689	1.8229654	0.00010727	0.00029743	1	2.86149399	1.51676858	0.00647142	0.01433444	1	0.808771	-0.3061968	0.6393151	0.93163024	0
feaR	3.22089435	1.68746134	6.49E-05	0.00019004	1	2.30899244	1.20726345	0.01338063	0.02698463	1	0.71687928	-0.4801979	0.31178042	0.81168136	0
feaB	4.05795626	2.02075332	2.02E-08	1.02E-07	1	2.90509017	1.53858294	4.26E-05	0.0001691	1	0.71589982	-0.4821704	0.42078214	0.87375001	0
dksA	0.2178043	-2.1988957	3.77E-12	2.96E-11	-1	0.19350862	-2.3695302	1.28E-10	1.55E-09	-1	0.88845181	-0.1706346	0.37112747	0.85517649	0
tynA	8.0551196	3.00990601	9.56E-16	1.04E-14	1	7.44653574	2.89656941	4.41E-12	6.49E-11	1	0.92444757	-0.1133366	0.92505491	1	0
paaZ	7.28484835	2.86489894	1.85E-07	8.25E-07	1	8.35921894	3.06336815	3.13E-07	2.06E-06	1	1.14748016	0.19846921	0.5422035	0.91914725	0
paaA	3.12564896	1.64415576	0.00042684	0.00103002	1	4.08584106	2.03063308	0.00011015	0.00039192	1	1.30719768	0.38647733	0.35394239	0.84775072	0
paaB	1.5861717	0.66554894	0.42278336	0.49507796	0	1.37928547	0.46392108	0.62387255	0.70239307	0	0.86956883	-0.2016279	0.83529982	0.98014902	0
paaC	2.0707599	1.05016028	0.06770069	0.0988542	0	3.23439457	1.69349569	0.02599395	0.04854915	1	1.56193607	0.6433354	0.40995372	0.87001729	0
paaD	8.0710118	3.01274954	2.86E-07	1.24E-06	1	10.9249333	3.44955256	1.10E-07	8.06E-07	1	1.35360145	0.43680302	0.35966528	0.85183882	0
paaE	3.86640677	1.95099343	0.00011238	0.00030967	1	3.96281056	1.986524	0.00022114	0.00072385	1	1.02493369	0.03553057	0.71116078	0.95223057	0
paaF	7.10111533	2.82804564	1.51E-05	4.95E-05	1	8.16737167	3.02987188	1.81E-05	7.89E-05	1	1.15015336	0.20182624	0.59822827	0.92806272	0
paaG	5.11580188	2.35496039	0.00804327	0.01467456	1	6.57758479	2.71755794	0.00483412	0.0110329	1	1.28573876	0.36259755	0.55809267	0.92317654	0
paaH	4.00605536	2.00218236	2.54E-05	8.03E-05	1	5.92133255	2.56592188	7.63E-07	4.61E-06	1	1.47809554	0.56373952	0.17045069	0.62318331	0
sfsA	0.34908707	-1.5183412	4.58E-05	0.00013854	-1	0.17190605	-2.5403078	1.10E-07	8.06E-07	-1	0.49244463	-1.0219666	0.01821377	0.14472561	0
paaI	1.81595793	0.86073078	0.29717741	0.36502576	0	1.07686616	0.10683895	0.61146734	0.69053774	0	0.59300171	-0.7538918	0.70482871	0.94896888	0
paaJ	1.18534195	0.24530331	0.29814578	0.36601165	0	1.53520764	0.6184338	0.17765517	0.25381772	0	1.29516013	0.37313048	0.51159771	0.90925684	0
paaK	1.86522646	0.8993508	0.02218004	0.03658299	0	1.71375945	0.77716462	0.05739068	0.09664992	0	0.91879431	-0.1221862	0.96246658	1	0
paaX	0.55613481	-0.8464935	0.11064586	0.15282138	0	0.66066925	-0.5979999	0.18171467	0.25811544	0	1.187966	0.24849355	0.86064435	0.98424873	0
paaY	0.74795566	-0.4189753	0.37846424	0.44973811	0	0.47095574	-1.0863366	0.03726629	0.06691578	0	0.6296573	-0.6673613	0.17255557	0.62838155	0
ynbG	74.1182098	6.21175613	0.15251813	0.20372935	0	7.59564055	2.92517163	0.4880463	0.57634329	0	0.10248009	-3.2865845	0.67536307	0.94091029	0
ydbA	2.26685528	1.18069229	0.00015138	0.00040722	1	2.21980991	1.15043614	0.00089708	0.00250108	1	0.97924642	-0.0302562	0.89360708	0.9954996	0
insD1	0	#NAME?	0.99327103	0.99847022	0	0	#NAME?	1	1	0	NA	NA	NA	NA	0
ligT	0.7807329	-0.357099	0.53238981	0.59988923	0	1.01713906	0.02451693	0.64819656	0.72442605	0	1.3028003	0.38161595	0.30431025	0.80892599	0
ydbC	0.25766754	-1.9564173	6.66E-08	3.15E-07	-1	0.2796459	-1.8383269	3.27E-05	0.00013361	-1	1.08529737	0.1180904	0.6846177	0.943596	0
ydbD	4.06593416	2.02358685	0.0045192	0.00869395	1	3.7499127	1.90685701	0.00362119	0.00855725	1	0.92227581	-0.1167298	0.74592758	0.95683847	0
ynbA	7.41477632	2.89040317	0.00024647	0.00063159	1	6.74157854	2.75308644	0.00108072	0.00296666	1	0.90920862	-0.1373167	1	1	0
ynbB	12.5360661	3.64801279	1.86E-05	6.02E-05	1	8.86012508	3.14732707	0.00015982	0.00054369	1	0.70677077	-0.5006857	0.87755695	0.98956956	0
ynbC	6.38598789	2.67490982	1.68E-12	1.37E-11	1	7.47131652	2.90136248	3.71E-11	4.83E-10	1	1.1699547	0.22645266	0.54333507	0.91914725	0
ynbD	3.99532331	1.99831225	1.52E-07	6.82E-07	1	5.29940351	2.40582998	5.93E-07	3.63E-06	1	1.32640167	0.40751773	0.4751687	0.89855206	0
azoR	0.52887685	-0.9189963	0.00483423	0.00922766	0	0.51499429	-0.9573717	0.03419268	0.06200142	0	0.97375085	-0.0383754	0.95192662	1	0
hrpA	0.75104639	-0.4130261	0.11329594	0.15613984	0	0.55348558	-0.8533824	0.0174006	0.03408388	0	0.73695258	-0.4403563	0.22098353	0.70414551	0
ydcF	0.63690669	-0.6508461	0.13490407	0.1824468	0	0.57912241	-0.7880598	0.09265334	0.14675094	0	0.90927355	-0.1372137	0.67168012	0.93979017	0
aldA	3.18732208	1.67234481	0.01044645	0.01859064	1	0.53268846	-0.9086361	0.20698115	0.28744491	0	0.16712728	-2.5809809	0.00081064	0.01441492	-1
hrpB	1.01923983	0.02749357	0.92227559	0.94189394	0	1.03578589	0.0507258	0.74356663	0.80220238	0	1.01623372	0.02323224	0.81516537	0.9719041	0
gapC	1.05081397	0.07150729	0.86453903	0.89283818	0	0.74385623	-0.4269043	0.31992299	0.41002247	0	0.70788575	-0.4984116	0.32527335	0.82473426	0
cybB	0.27771961	-1.8482991	7.50E-08	3.52E-07	-1	0.24628184	-2.0216179	2.98E-06	1.57E-05	-1	0.88680032	-0.1733188	0.64927499	0.93327989	0
rydC	1.54549145	0.62806568	0.6142	0.67485607	0	0.70745101	-0.4992978	0.49141929	0.57939751	0	0.45775149	-1.1273635	0.21858017	0.70102268	0
ydcA	0.16304737	-2.616637	3.94E-10	2.53E-09	-1	0.21054245	-2.2478169	2.25E-07	1.54E-06	-1	1.29129624	0.36882001	1	1	0
hokB	0.60158457	-0.7331605	0.22090337	0.28192146	0	0.14840636	-2.7523751	0.00463209	0.01062231	-1	0.24669244	-2.0192146	0.04099771	0.26051858	0
mokB	0	#NAME?	1	1	0	0	#NAME?	1	1	0	NA	NA	NA	NA	0
sokB	0.18642754	-2.4233131	0.00101022	0.0022662	-1	0.30554965	-1.7105213	0.00254078	0.00623414	-1	1.63897269	0.71279182	0.91000296	0.99971546	0
trg	5.59672459	2.48458276	5.21E-11	3.65E-10	1	5.77725006	2.53038294	3.04E-08	2.45E-07	1	1.03225556	0.04580019	1	1	0
ydcI	4.00997692	2.00359393	1.78E-07	7.94E-07	1	2.61430661	1.38642835	0.00151498	0.00398255	1	0.65195054	-0.6171656	0.17681535	0.63784355	0
ydcJ	2.13272282	1.09269647	0.00177652	0.00377437	1	1.21861206	0.28523892	0.43740375	0.5275347	0	0.57138792	-0.8074576	0.05202097	0.30506977	0
mrcB	0.72059801	-0.4727334	0.14960221	0.20030099	0	0.7617533	-0.3926043	0.14959662	0.21947791	0	1.05711268	0.08012916	0.81139478	0.96998188	0
opgD	0.6255184	-0.6768758	0.05354271	0.08074365	0	0.61450187	-0.7025107	0.12248111	0.18481327	0	0.98238816	-0.0256349	0.95296648	1	0

ydcH	0.12169501	-3.0386581	3.11E-19	4.37E-18	-1	0.07984214	-3.6467058	3.45E-18	1.37E-16	-1	0.6560839	-0.6080478	0.03714458	0.24196102	0
rimL	0.78859057	-0.3426516	0.30212949	0.3700793	0	0.77001955	-0.377033	0.3732743	0.46464764	0	0.97645036	-0.0343814	0.91338219	1	0
ydcK	2.14298651	1.09962277	0.02957159	0.04734241	1	0.92960196	-0.105315	1	1	0	0.43378806	-1.2049378	0.04923775	0.29394474	0
tehA	1.95729864	0.96886389	0.01426789	0.02465621	0	1.50478011	0.58955268	0.15467413	0.22580277	0	0.76880456	-0.3793112	0.46087035	0.88879788	0
tehB	0.37914591	-1.3991749	3.98E-05	0.0001216	-1	0.4934829	-1.018928	0.00911222	0.01938704	-1	1.30156461	0.38024693	0.45756244	0.88755478	0
ydcL	0.62223552	-0.6844673	0.09859044	0.13811417	0	0.2474217	-2.0149561	0.00174046	0.00449245	-1	0.39763351	-1.3304887	0.03661518	0.2400068	0
insP	1.70438593	0.76925205	0.25463007	0.31950135	0	1.18125887	0.24032517	0.75726434	0.81384287	0	0.69307007	-0.5289269	0.47525976	0.89855206	0
insQ	0.61087887	-0.7110418	0.04596044	0.07066038	0	0.61282471	-0.7064536	0.08284425	0.13351157	0	1.00318531	0.00458813	0.92301935	1	0
ydcO	0.72837882	-0.4572391	0.36599517	0.43670928	0	1.0703766	0.09811849	0.7146261	0.780514	0	1.46953286	0.55535762	0.21904972	0.70102268	0
fhuA	0.5169619	-0.9518701	0.02593814	0.04213636	0	0.56162552	-0.8323196	0.08123097	0.13129087	0	1.08639634	0.11955052	0.93511217	1	0
ydcN	1.31822658	0.39859836	0.62902682	0.68875793	0	1.75485578	0.81135247	0.13474948	0.20062599	0	1.3312247	0.41275411	0.31750186	0.81786403	0
ydcP	1.32742808	0.4086337	0.2313954	0.29386816	0	1.24268584	0.31346162	0.48089701	0.57003323	0	0.93616058	-0.0951721	0.78380961	0.9635071	0
yncJ	2.68037904	1.42243703	0.04547594	0.06998859	0	2.05520383	1.03928148	0.12288606	0.18520924	0	0.76675866	-0.3831555	0.83616904	0.98025477	0
hicA	0.40552551	-1.3021354	0.11965041	0.16392552	0	0.7316949	-0.4506859	0.80987275	0.85474748	0	1.80431287	0.85144953	0.23281475	0.72303736	0
hicB	0.51729778	-0.9509331	0.04355833	0.06741315	0	0.43320488	-1.2068786	0.04943398	0.08516521	0	0.83743812	-0.2559455	0.73126711	0.95551996	0
ydcR	1.48551631	0.57096445	0.1257585	0.1713901	0	1.12237929	0.1665603	0.37413625	0.46541568	0	0.75554828	-0.4044041	0.6491144	0.93327989	0
ydcS	0.69507845	-0.5247523	0.12232343	0.16712113	0	0.27414837	-1.8669712	0.00014522	0.00050133	-1	0.39441356	-1.3422189	0.00605849	0.06453609	0
ydcT	1.90113004	0.92685722	0.00937181	0.01684543	0	0.86075666	-0.2163226	1	1	0	0.45276054	-1.1431799	0.02615737	0.19098347	0
ydcU	2.92662661	1.54923869	0.00016616	0.00044214	1	2.35711757	1.23702372	0.00277433	0.00674726	1	0.80540427	-0.312215	0.75836369	0.95806023	0
ydcV	5.26075362	2.39526949	0.00035805	0.00087862	1	1.4621875	0.54812832	0.32380495	0.41407674	0	0.27794259	-1.8471412	0.0301902	0.2109965	0
fhuC	0.85199414	-0.2310846	0.446747	0.51710731	0	1.04686866	0.06608045	0.88145468	0.9109327	0	1.22872753	0.29716503	0.44907869	0.88755478	0
patD	9.43790739	3.23846701	3.74E-12	2.94E-11	1	3.46267003	1.79188492	0.0057951	0.01298607	1	0.3668896	-1.4465821	0.00156876	0.02429746	-1
yncL	4.01068445	2.00384846	0.0008133	0.00185939	1	1.42918177	0.51518942	0.92335639	0.9436429	0	0.35634361	-1.488659	0.0081587	0.08049185	0
ydcX	2.05790855	1.04117888	0.11528482	0.15858395	0	2.01201085	1.00863808	0.17161063	0.24677754	0	0.97769692	-0.0325408	1	1	0
ydcY	0.15278441	-2.7104308	2.71E-13	2.37E-12	-1	0.1920421	-2.3805055	1.81E-08	1.53E-07	-1	1.25694831	0.32992533	0.78066659	0.9635071	0
ydcZ	1.43717451	0.52323525	0.285339	0.35254332	0	1.33282064	0.41448264	0.41188965	0.50252357	0	0.92738956	-0.1087526	0.91737262	1	0
mnaT	0.5234381	-0.9339092	0.00850958	0.01544875	0	0.55147279	-0.8586384	0.05199068	0.08887691	0	1.05355875	0.07527077	0.86518901	0.9856241	0
curA	2.36374402	1.24107381	0.00802274	0.01464316	1	1.23470801	0.3041699	0.37562545	0.4669167	0	0.52235267	-0.9369039	0.14447141	0.57607933	0
mcbR	4.62971554	2.21092355	6.73E-05	0.00019665	1	3.38977249	1.76118845	0.01041342	0.02180793	1	0.73217727	-0.4497351	0.40420978	0.86854717	0
yncD	3.35570994	1.74661802	0.000616	0.00144266	1	3.47467256	1.79687703	0.00192231	0.0048989	1	1.03545081	0.05025901	0.81222538	0.97044538	0
yncE	0.49728024	-1.007869	0.00421071	0.00816091	-1	0.5144457	-0.9589093	0.01526625	0.0303881	0	1.03451868	0.0489597	0.94428766	1	0
fhuD	0.52261854	-0.9361698	0.01836834	0.03082559	0	1.03211833	0.04560839	0.95774543	0.97114015	0	1.97489803	0.98177816	0.03761868	0.24408514	0
ansP	3.1570808	1.65859119	0.00128585	0.00281586	1	3.30384739	1.72414705	0.00047005	0.0013922	1	1.04648807	0.06555586	0.53642434	0.9187668	0
yncG	7.9642244	2.99353387	5.09E-09	2.83E-08	1	4.9687106	2.31287152	0.00019906	0.00065793	1	0.62387878	-0.6806624	0.17561064	0.63459078	0
yncH	8.17760753	3.03167882	0.05212705	0.07893219	0	3.47787724	1.79820701	0.31488248	0.40508462	0	0.42529276	-1.2334718	0.55974635	0.92432422	0
rhsE	3.69277591	1.88470572	0.01133022	0.02000222	1	3.01008748	1.58980541	0.03978437	0.07091856	0	0.81512866	-0.2949003	0.89256118	0.99488354	0
ydcD	4.99413113	2.3202337	0.08716273	0.12351549	0	8.04921901	3.00884881	0.10428736	0.16216866	0	1.61173562	0.68861511	0.81076415	0.96998188	0
yncI	3.93339304	1.97577435	0.00323075	0.00644837	1	2.55703149	1.35446993	0.06341224	0.10598286	0	0.65008288	-0.6213044	0.47800907	0.89855925	0
ydcC	2.17338042	1.11994072	0.79532136	0.83462448	0	2.92480401	1.54833996	0.3769314	0.46801345	0	1.34573956	0.42839923	0.60250437	0.92806272	0
pptA	0.27481478	-1.8634685	0.0004705	0.00112638	-1	0.13357866	-2.9042363	9.20E-05	0.00033403	-1	0.4860687	-1.0407678	0.17482366	0.63298224	0
yddH	1.12652087	0.17187404	0.62960551	0.68875793	0	0.72344403	-0.4670467	0.44325286	0.53327917	0	0.64219319	-0.6389207	0.23585218	0.72836703	0
nhoA	6.92798604	2.79243602	1.01E-09	6.15E-09	1	4.34137905	2.11815339	2.48E-06	1.34E-05	1	0.62664374	-0.6742826	0.40044175	0.86854717	0
mokC	0	#NAME?	1	1	0	0	#NAME?	1	1	0	NA	NA	NA	NA	0
fhuB	1.04443945	0.06272885	0.99044204	0.9965336	0	1.82768763	0.87001952	0.04745265	0.08220041	0	1.74992206	0.80729067	0.04557269	0.27952095	0
yddE	0.77762063	-0.3628616	0.21159553	0.27161217	0	0.72513948	-0.4636696	0.44454226	0.53424864	0	0.9325106	-0.100808	0.8719644	0.98773886	0
narV	7.60588212	2.92711558	8.40E-11	5.74E-10	1	2.81358221	1.49240812	0.00010893	0.0003882	1	0.36992188	-1.4347075	0.03426276	0.23033348	0
narW	9.05445015	3.17862703	3.98E-11	2.84E-10	1	2.59555609	1.37604366	0.00239166	0.00594752	1	0.28666082	-1.8025834	0.00472754	0.05264765	0
narY	9.55311101	3.25597063	9.93E-17	1.18E-15	1	3.47442622	1.79677474	8.69E-06	4.16E-05	1	0.36369579	-1.4591959	0.00228195	0.03143336	-1
narZ	11.8087958	3.56178995	9.39E-25	2.01E-23	1	4.26477888	2.09247094	6.12E-10	6.69E-09	1	0.36115273	-1.469319	0.00107345	0.01753295	-1
narU	8.99283931	3.16877669	2.71E-15	2.87E-14	1	4.21190219	2.07447194	8.13E-07	4.87E-06	1	0.46836178	-1.0943048	0.03160986	0.21686982	0
yddK	11.1701758	3.48157998	0.03609032	0.05675007	0	2.938564	1.55511132	0.28355679	0.37292781	0	0.26307232	-1.9264687	0.37115823	0.85517649	0
yddL	8.73303937	3.12648384	0.05656135	0.08474609	0	4.97514742	2.31473928	0.17353251	0.24897408	0	0.56969254	-0.8117446	0.76381785	0.95937995	0
yddG	1.35257578	0.43570942	0.1713727	0.22575401	0	1.15800948	0.21164706	0.41072048	0.50151251	0	0.85615127	-0.2240624	0.81911788	0.97429253	0
fdnG	0.1504567	-2.7325797	1.41E-15	1.51E-14	-1	0.6680297	-0.5820159	0.08584911	0.13765139	0	4.44001298	2.15056389	1.98E-08	2.08E-06	1
hemL	0.29417826	-1.7652375	5.48E-08	2.64E-07	-1	0.23670341	-2.0788476	1.35E-06	7.71E-06	-1	0.80462579	-0.3136101	0.71163999	0.95223057	0
fdnH	0.42229732	-1.243669	0.01455046	0.02506606	-1	0.79178993	-0.3368104	0.4806025	0.56983699	0	1.87495844	0.90685862	0.09287308	0.44325786	0
fdnI	0.21540566	-2.2148719	3.11E-07	1.34E-06	-1	0.38466242	-1.3783352	0.00295364	0.00714013	-1	1.78575814	0.8365367	0.13067443	0.5395826	0
yddM	0.54131423	-0.8854618	0.06741666	0.09856981	0	0.50370625	-0.9893455	0.03904157	0.06967879	0	0.93052468	-0.1038837	0.55511354	0.92101231	0
adhP	3.35483891	1.7462435	0.00065095	0.00151568	1	1.325414	0.40644307	0.35080169	0.44165033	0	0.3950753	-1.3398004	0.04566991	0.27972822	0
maeA	0.36746759	-1.4443111	1.79E-06	7.00E-06	-1	0.37086553	-1.4310319	0.00011098	0.0003939	-1	1.00924691	0.01327917	0.98463484	1	0
sra	1.07418328	0.10324017	0.8211389	0.85703462	0	0.76192798	-0.3922735	0.39038335	0.48133483	0	0.7093091	-0.4955136	0.41629446	0.87131399	0
bdm	2.9485271	1.55999446	0.43226483	0.5040427	0	7.20436468	2.84887121	0.18087355	0.25700329	0	2.44337746	1.28887675	0.49086389	0.90535749	0
osmC	2.03537137	1.02529205	0.21623976	0.27701016	0	1.1745041	0.23205176	1	1	0	0.57704659	-0.7932403	0.27639523	0.77885174	0
ddpF	4.52436404	2.17771502	1.35E-08	7.11E-08	1	4.59859563	2.20119334	1.45E-06	8.24E-06	1	1.01640708	0.02347832	1	1	0
ddpD	5.56483446	2.47633877	1.88E-06	7.31E-06	1	4.85861458	2.28054499	1.70E-05	7.45E-05	1	0.87309238	-0.1957938	1	1	0
clcA	1.14050517	0.18967298	0.63200582	0.69029914	0	0.78089103	-0.3568069	0.38595015	0.4769005	0	0.68468873	-0.5464798	0.17866503	0.64266947	0
ddpC	5.72411171	2.51705183	2.70E-07	1.17E-06	1	5.06184774	2.33966411	1.43E-05	6.45E-05	1	0.88430275	-0.1773877	0.91567556	1	0
ddpB	7.85744868	2.97406094	1.33E-11	9.91E-11	1	9.36163489	3.2267605	1.59E-10	1.90E-09	1	1.19143443	0.25269955	0.53996539	0.91914725	0
ddpA	4.72770524	2.24114009	6.15E-08	2.93E-07	1	4.26292758	2.09184455	1.09E-05	5.04E-05	1	0.90169064	-0.1492955	0.86538243	0.9856241	0
ddpX	4.52642629	2.17837246	4.24E-05	0.00012903	1	4.19211894	2.06767965	0.00031776	0.00099158	1	0.9261432	-0.1106928	1	1	0
dosP	7.00775875	2.80895311	2.33E-11	1.71E-10	1	4.70376129	2.23381485	6.80E-08	5.20E-07	1	0.67122192	-0.5751383	0.49289751	0.90539057	0
dosC	4.58344768	2.19643321	8.49E-08	3.95E-07	1	1.6578902	0.72934846	0.04329198	0.07621804	0	0.36171247	-1.4670847	0.00611739	0.06490473	0
yddW	1.47345857	0.55920649	0.16921363	0.22317605	0	1.6081561	0.68540745	0.06243775	0.10462537	0	1.09141589	0.12620096	0.52335115	0.91269012	0
gadC	5.66982757	2.50330486	0.01120376	0.01980272	1	0.84784736	-0.2381235	0.84943823	0.8865977	0	0.14953671	-2.7414284	0.01995054	0.15545662	0
gadB	5.35485269	2.42084689	0.00767402	0.01407648	1	0.78776935	-0.3441548	0.7206784	0.78575817	0	0.14711317	-2.7650017	0.00980081	0.0919608	0
pqqL	1.84388269	0.88274687	0.01735424	0.02935798	0	1.33739379	0.41942432	0.16152499	0.23448717	0	0.72531393	-0.4633225	0.54466765	0.91914725	0
erpA	0.80468114	-0.3135109	0.30258558	0.37022729	0	0.55236734	-0.8563001	0.04043404	0.07193158	0	0.68644251	-0.5427892	0.16329004	0.6114388	0
yddB	3.10103925	1.63275178	3.10E-05	9.63E-05	1	2.8686845	1.52038931	0.0002176	0.00071334	1	0.92507198	-0.1123625	0.92797859	1	0
yddA	6.79738679	2.76498022	3.21E-09	1.84E-08	1	8.23735382	3.04218096	2.19E-07	1.51E-06	1	1.21184127	0.27720074	0.77864502	0.9635071	0
ydeM	2.15099224	1.10500232	0.18618703	0.2429507	0	1.81368313	0.85892243	0.40539612	0.4959705	0	0.84318441	-0.2460799	0.74365823	0.95683847	0
ydeN	0.90695451	-0.1408979	0.58544915	0.64891565	0	0.87196045	-0.1976654	0.32486366	0.41498936	0	0.96141586	-0.0567675	0.53500992	0.9187668	0
ydeO	5.45358555	2.44720507	0.00084021	0.00191497	1	5.24118276	2.38989242	0.00704024	0.01544727	1	0.96105263	-0.0573126	0.95394606	1	0
safA	12.1155102	3.59878325	0.05496376	0.08260461	0	1.60733428	0.68467	0.537199	0.62306624	0	0.13266749	-2.9141133	0.36787392	0.85430436	0
ydeP	3.05403463	1.61071642	0.00019128	0.00050182	1	3.84272786	1.94213081	5.42E-05	0.00020846	1	1.25824633	0.33141439	0.35934061	0.85183882	0
ydeQ	6.97866468	2.80295101	1.84E-06	7.16E-06	1	7.91481022	2.98455476	2.31E-05	9.78E-05	1	1.13414393	0.18160374	0.80932489	0.96990832	0
ydeR	9.51074372	3.24955816	5.28E-07	2.21E-06	1	7.72156189	2.9488927	1.98E-05	8.52E-05	1	0.81187782	-0.3006655	0.87571433	0.98905525	0
ydeS	8.02139078	3.0038524	1.69E-06	6.64E-06	1	9.68413533	3.27562324	3.14E-06	1.63E-05	1	1.20728881	0.27177084	0.59453448	0.92806272	0
yadS	2.09241952	1.06517214	0.02992104	0.04784984	1	2.09723639	1.06848949	0.01226864	0.02506477	1	1.00230206	0.00331735	0.60060946	0.92806272	0
ydeT	6.39342424	2.67658883	0.00037306	0.00091292	1	6.28198695	2.65122095	0.00083321	0.00233478	1	0.98257002	-0.0253679	0.79097508	0.96519095	0
yneL	9.87628462	3.30396841	0.00021244	0.00055204	1	7.90600174	2.98294827	0.0025886	0.0063374	1	0.80050364	-0.3210201	0.85267849	0.98236299	0
hipA	0.48646999	-1.0395773	0.00809805	0.01476232	-1	0.43818714	-1.1903809	0.01439728	0.02880109	-1	0.90074856	-0.1508036	0.80325838	0.96977549	0
hipB	0.22286829	-2.1657367	6.91E-06	2.44E-05	-1	0.14062223	-2.8301034	1.69E-05	7.41E-05	-1	0.63096564	-0.6643667	0.39253664	0.86854717	0
yneO	8.42382128	3.07447483	6.70E-20	9.74E-19	1	9.12513552	3.18984598	3.90E-15	9.18E-14	1	1.08325369	0.11537116	0.9887739	1	0
lsrK	1.79766597	0.84612498	0.03521227	0.05560693	0	1.14657295	0.19732815	0.37276207	0.46414077	0	0.63781201	-0.6487968	0.28899496	0.79141721	0
lsrR	1.26511042	0.33926331	0.33019194	0.39974441	0	0.69519963	-0.5245008	0.25982904	0.34698838	0	0.54951696	-0.8637641	0.04892659	0.29394474	0
lsrA	2.05521563	1.03928977	0.00454602	0.00873033	1	1.45472646	0.5407479	0.09926247	0.15571205	0	0.70782182	-0.4985419	0.41740393	0.872394	0
lsrC	2.88897494	1.53055769	0.00015441	0.00041485	1	2.23056156	1.15740697	0.00660223	0.01459492	1	0.77209447	-0.3731507	0.60767038	0.92806272	0
lsrD	3.38498606	1.75914989	0.00012435	0.00033842	1	2.31570013	1.21144845	0.00603885	0.01346074	1	0.68410921	-0.5477014	0.54341019	0.91914725	0
btuF	1.11000242	0.15056282	0.76715514	0.81201156	0	1.03176537	0.04511493	0.68966575	0.75925086	0	0.92951633	-0.1054479	0.93115364	1	0
lsrB	3.05086644	1.60921902	0.00041943	0.00101429	1	1.22773048	0.29599388	0.33088973	0.42053544	0	0.40242026	-1.3132251	0.03124707	0.21564879	0
lsrF	3.18155354	1.6697314	0.00049012	0.00116892	1	1.37036013	0.45455508	0.2081866	0.28902814	0	0.43072043	-1.2151763	0.0702437	0.36820631	0
lsrG	4.16778467	2.05928074	0.00815252	0.01485548	1	1.26485295	0.33896967	0.64154926	0.71917965	0	0.30348328	-1.7203111	0.06584009	0.35237234	0

tam	2.25990755	1.17626376	0.00234323	0.00483422	1	1.53680215	0.61993144	0.15076625	0.22104713	0	0.68002877	-0.5563323	0.25478206	0.75459294	0
yneE	2.45753244	1.29721046	0.01450428	0.0250083	1	1.82551582	0.86830417	0.07210089	0.11866437	0	0.74282471	-0.4289063	0.67570587	0.94091029	0
uxaB	0.19201577	-2.3807033	8.80E-11	5.99E-10	-1	0.24962732	-2.0021523	3.17E-06	1.65E-05	-1	1.3000355	0.37855102	0.36679927	0.85342842	0
yneF	2.22614375	1.15454676	0.00953112	0.0170995	1	1.9820341	0.98698179	0.04296141	0.07572655	0	0.89034417	-0.167565	0.80322943	0.96977549	0
yneG	1.65849182	0.72987189	0.08813938	0.12457973	0	2.64051335	1.40081844	0.04104712	0.07281703	0	1.5921172	0.67094654	0.43953089	0.88087171	0
glsB	2.68254758	1.42360376	0.00034989	0.00086194	1	3.12983447	1.64608636	0.00037078	0.00113428	1	1.16673959	0.2224826	0.57734178	0.92806272	0
sad	1.05768107	0.08090466	0.74480296	0.79233976	0	0.79701039	-0.3273296	0.72675704	0.79082477	0	0.75354511	-0.4082342	0.522051	0.91269012	0
mtn	0.12714426	-2.9754618	4.18E-18	5.62E-17	-1	0.13072606	-2.9353813	1.73E-12	2.69E-11	-1	1.02817118	0.04008048	0.91688796	1	0
yneJ	2.20774371	1.1425727	0.00246874	0.00506476	1	2.41630572	1.272803	0.0131734	0.02664222	1	1.0944684	0.1302303	0.95941391	1	0
yneK	7.34416624	2.87659871	6.93E-08	3.26E-07	1	7.36206318	2.88011013	4.84E-06	2.43E-05	1	1.00243689	0.00351142	0.99357704	1	0
ydeA	3.73481811	1.90103798	0.00017534	0.00046432	1	4.0359059	2.01289254	0.00025012	0.00080561	1	1.08061645	0.11185456	0.64630392	0.93208609	0
marC	1.83854376	0.87856351	0.02486534	0.04057267	0	1.17278307	0.22993619	0.70019434	0.76841352	0	0.63788695	-0.6486273	0.11883606	0.51429541	0
marR	0.77160256	-0.3740702	0.64927516	0.70549776	0	1.08532124	0.11812213	0.82408853	0.86643998	0	1.40658066	0.49219229	0.80131871	0.96905452	0
marA	0.97927649	-0.0302118	0.88298394	0.90891169	0	0.9930062	-0.0101254	0.50278904	0.59075373	0	1.01402026	0.02008647	0.57531834	0.92806272	0
marB	0.76996278	-0.3771394	0.40198751	0.47455695	0	0.65229958	-0.6163934	0.29084903	0.38048013	0	0.84718326	-0.239254	0.63847959	0.93163024	0
eamA	5.17171779	2.37064355	5.68E-08	2.72E-07	1	4.81132516	2.2664343	8.94E-05	0.0003261	1	0.93031471	-0.1042093	0.57945805	0.92806272	0
ydeE	3.38157078	1.75769355	1.17E-05	3.93E-05	1	3.40457359	1.76747412	0.00026358	0.00084318	1	1.0068024	0.00978057	0.9869381	1	0
yneM	0.50447246	-0.9871526	0.22911473	0.29130695	0	1.08228453	0.11407983	0.68732168	0.75780302	0	2.14537883	1.10123242	0.54737475	0.91914725	0
dgt	0.5481661	-0.867315	0.01382246	0.0239897	0	0.41274869	-1.2766645	0.0080677	0.01738232	-1	0.75296282	-0.4093495	0.49849233	0.90539057	0
mgrR	1.80002528	0.84801717	0.20325327	0.26235234	0	2.9517643	1.56157753	0.24700038	0.33246868	0	1.63984602	0.71356035	0.78672905	0.9635071	0
dgcZ	2.44775426	1.29145873	0.01098741	0.01946706	1	3.044935	1.60641143	0.01013098	0.02130833	1	1.24397087	0.3149527	0.67751462	0.94128448	0
ydeI	3.85226073	1.94570535	0.00221432	0.00459401	1	2.06954994	1.04931706	0.32637162	0.41647017	0	0.53722998	-0.8963883	0.12247709	0.52159375	0
ydeJ	2.41049501	1.26932944	0.05389017	0.08121218	0	1.90547446	0.93015027	0.19687742	0.2760257	0	0.79049094	-0.3391792	0.74366757	0.95683847	0
dcp	0.38393204	-1.3810771	2.73E-05	8.58E-05	-1	0.46694617	-1.0986718	0.00944347	0.02001473	-1	1.21622091	0.2824053	0.34381791	0.84269271	0
ydfG	0.11353805	-3.1387522	4.79E-21	7.58E-20	-1	0.09602103	-3.3805058	2.38E-16	6.79E-15	-1	0.84571675	-0.2417535	0.49378565	0.90539057	0
rspR	0.59231067	-0.755574	0.02508161	0.04089534	0	0.75180692	-0.4115659	0.17923206	0.25565735	0	1.26927803	0.34400812	0.64706413	0.93208609	0
ydfZ	0.18043574	-2.470443	5.22E-06	1.89E-05	-1	0.63933833	-0.6453485	0.11420081	0.17383857	0	3.54330207	1.82509446	0.01550834	0.12831481	0
ydfI	3.98605416	1.99496131	3.24E-06	1.22E-05	1	4.57101521	2.19251462	3.45E-05	0.00014021	1	1.14675191	0.19755331	0.78652353	0.9635071	0
ydfJ	11.3064245	3.49907087	9.04E-15	9.08E-14	1	11.3686792	3.50699275	7.64E-12	1.09E-10	1	1.00550613	0.00792188	0.96850838	1	0
degP	0.87633076	-0.1904526	0.48378349	0.55345031	0	0.28920489	-1.7898362	5.27E-06	2.63E-05	-1	0.33001796	-1.5993836	3.08E-05	0.00100719	-1
pinQ	2.44717352	1.2911164	0.96476707	0.97581318	0	3.79782028	1.92517163	0.74495759	0.8031148	0	1.55192113	0.63405524	0.82232195	0.97616145	0
stfQ	6.62567837	2.72806817	3.51E-07	1.50E-06	1	7.8154387	2.96632686	2.09E-07	1.45E-06	1	1.17956808	0.23825869	0.44915435	0.88755478	0
nohQ	23.5814088	4.55957801	0.08724589	0.12359368	0	7.04439846	2.81647652	0.23117161	0.3157797	0	0.29872679	-1.7431015	0.72050739	0.95399541	0
ynfO	4.41491974	2.14238721	0.00375183	0.00736854	1	3.47255501	1.79599755	0.07968212	0.12902723	0	0.78654998	-0.3463897	0.60724867	0.92806272	0
ydfO	1.08050117	0.11170064	0.97474348	0.98314023	0	1.29726106	0.37546883	0.93408264	0.95350224	0	1.2006105	0.26376819	0.94658523	1	0
gnsB	0.04861165	-4.3625542	6.12E-14	5.67E-13	-1	0.29301997	-1.7709291	0.00043561	0.00130418	-1	6.02777321	2.59162514	0.00084933	0.01474619	1
ynfN	36.7107325	5.19813	1.56E-08	8.10E-08	1	19.9949719	4.32156535	5.67E-05	0.00021719	1	0.54466284	-0.8765646	0.29160145	0.79429425	0
cspI	4.48838395	2.16619609	0.05410184	0.08150335	0	6.36683454	2.67057627	0.0537615	0.09151467	0	1.41851379	0.50438018	0.7426705	0.95683847	0
cdaR	0.4685651	-1.0936786	0.00108231	0.00240592	-1	0.42523904	-1.233654	0.00675859	0.01489586	-1	0.9075346	-0.1399755	0.95205682	1	0
rzpQ	6.58899823	2.72005914	0.05754128	0.08595192	0	4.69162978	2.23008918	0.07472846	0.12236785	0	0.71203992	-0.48997	1	1	0
rrrQ	6.20918617	2.63440419	0.00010311	0.00028699	1	8.15203519	3.02716028	4.96E-05	0.00019428	1	1.31289914	0.39275609	0.45153882	0.88755478	0
ydfR	2.4304352	1.28121467	0.29432542	0.36222774	0	3.52112319	1.8160357	0.14927213	0.21907457	0	1.44876242	0.53482103	0.58434878	0.92806272	0
essQ	3.21538635	1.6849921	0.14572853	0.19552944	0	6.11467003	2.61227465	0.08430396	0.13556739	0	1.90169061	0.92728255	0.55990454	0.92432422	0
cspB	0.89201764	-0.1648559	0.82211398	0.85724208	0	3.25794535	1.7039624	0.46862403	0.55803007	0	3.65233287	1.86881826	0.34135209	0.8405152	0
cspF	2.7755611	1.47277945	0.32238341	0.39184577	0	4.82439783	2.27034888	0.19388248	0.27294255	0	1.73817029	0.79756943	0.63733181	0.93163024	0
quuQ	3.84796513	1.94409573	0.0031815	0.00636733	1	4.16708558	2.05903873	0.00363927	0.00859078	1	1.08293226	0.114943	0.68264008	0.94348666	0
ydfU	4.83608874	2.27384072	2.53E-06	9.61E-06	1	6.91859199	2.79047846	9.85E-07	5.82E-06	1	1.43061725	0.51663775	0.32299496	0.82325896	0
rem	3.0953887	1.63012058	0.0205399	0.03408143	1	2.55892227	1.35553632	0.08552182	0.13727603	0	0.82668851	-0.2745843	0.79781855	0.96743774	0
yaeH	0.0919023	-3.4437553	1.47E-25	3.39E-24	-1	0.05214126	-4.2614307	6.82E-24	7.01E-22	-1	0.56735537	-0.8176754	0.01830267	0.14495371	0
hokD	0.68049897	-0.5553351	0.25276489	0.31744741	0	0.22271911	-2.1667027	1.76E-06	9.82E-06	-1	0.32728795	-1.6113676	7.84E-05	0.00221717	-1
relE	0.3949288	-1.3403355	0.00030733	0.00076564	-1	0.11453528	-3.126136	2.19E-10	2.56E-09	-1	0.29001502	-1.7858005	0.00011344	0.00297781	-1
relB	0.2331152	-2.100885	9.81E-07	3.96E-06	-1	0.11191011	-3.1595877	9.05E-10	9.61E-09	-1	0.48006354	-1.0587027	0.01085326	0.09909497	0
ydfV	1.43953356	0.52560143	0.4161185	0.4892189	0	1.63855971	0.71242824	0.3909184	0.48162934	0	1.13825738	0.18682682	0.84043577	0.98067952	0
flxA	1.93146519	0.94969568	0.08409443	0.11966659	0	3.05351075	1.61046893	0.06622182	0.11022005	0	1.58092974	0.66077325	0.57996458	0.92806272	0
intK	9.57174111	3.25878138	0.07503045	0.10806716	0	5.04673193	2.33534946	0.10645506	0.16448424	0	0.52725328	-0.9234319	0.91972434	1	0
ydfX	8.79750762	3.13709486	0.0029803	0.00600547	1	4.63528487	2.21265801	0.04975252	0.08568058	0	0.52688614	-0.9244369	0.5030341	0.90620114	0
dicC	2.44282276	1.28854919	0.48285575	0.55296185	0	3.12538328	1.64403312	0.40495893	0.49571328	0	1.27941467	0.35548393	0.83568261	0.98014902	0
dicA	0.46163036	-1.11519	0.00386999	0.00758038	-1	0.45641142	-1.1315932	0.00384424	0.00903121	-1	0.98869455	-0.0164032	0.54839157	0.91919682	0
ydfA	1.00048435	0.0006986	0.73832722	0.78639772	0	2.62542254	1.39254963	0.22910212	0.31363143	0	2.62415154	1.39185103	0.16717314	0.61693183	0
yaeI	2.28249665	1.19061274	0.00458855	0.00879596	1	2.59735459	1.37704298	0.00310013	0.00743326	1	1.13794454	0.18643024	0.57395324	0.92806272	0
ydfB	2.75267632	1.46083498	0.23854135	0.30172886	0	1.02717829	0.03868661	0.63675054	0.7145253	0	0.37315622	-1.4221484	0.6040656	0.92806272	0
ydfC	1.4037585	0.48929476	0.80605497	0.8436836	0	1.44347566	0.52954678	0.56791472	0.65167882	0	1.02829344	0.04025202	0.80787807	0.96990832	0
dicF	0.64793494	-0.6260791	0.55815518	0.6238288	0	1.33426779	0.41604825	0.71098459	0.77729859	0	2.05926199	1.04212739	0.39328431	0.86854717	0
dicB	2.6972377	1.43148267	0.45716895	0.52723636	0	1.82634896	0.86896245	0.4681094	0.55757335	0	0.67711828	-0.5625202	1	1	0
ydfD	10.5017253	3.39255445	0.01108364	0.01960611	1	9.34519511	3.22422478	0.00804181	0.017335	1	0.88987237	-0.1683297	0.75741192	0.95806023	0
ydfE	5.14676814	2.36366679	4.35E-05	0.00013217	1	7.79851724	2.96319985	3.58E-06	1.84E-05	1	1.51522607	0.59953306	0.24528859	0.74396332	0
intQ	7.05939057	2.81954364	7.84E-10	4.86E-09	1	5.87214169	2.55388678	1.49E-06	8.47E-06	1	0.83181992	-0.2656569	0.61676078	0.92897598	0
rspB	42.0348566	5.39351425	3.07E-11	2.23E-10	1	31.6595914	4.98457073	5.04E-07	3.15E-06	1	0.75317472	-0.4089435	0.31910422	0.81866417	0
rspA	3.04827077	1.60799106	0.00997754	0.01782078	1	3.13145188	1.64683171	0.00550893	0.01240141	1	1.02728796	0.03884065	0.61222729	0.92897598	0
dapD	0.17894063	-2.4824471	8.46E-15	8.55E-14	-1	0.14974572	-2.7394133	2.41E-12	3.69E-11	-1	0.83684581	-0.2569663	0.40018673	0.86854717	0
ynfA	2.90865497	1.54035217	0.00030079	0.00075104	1	4.11980928	2.04257755	9.78E-05	0.00035312	1	1.41639669	0.50222538	0.33208004	0.83208693	0
ynfB	0.28937973	-1.7889642	1.21E-06	4.85E-06	-1	0.36704172	-1.445984	0.0006681	0.00191337	-1	1.26837401	0.34298022	0.64019809	0.93163024	0
speG	0.343486	-1.5416768	0.00024218	0.0006212	-1	0.27378821	-1.8688677	0.00017171	0.00057836	-1	0.79708696	-0.327191	0.41100911	0.87071045	0
ynfC	0.56746522	-0.8173961	0.03951011	0.06175377	0	0.54045178	-0.8877622	0.1055053	0.16347838	0	0.95239631	-0.0703661	1	1	0
ynfD	0.31713685	-1.6568226	2.26E-05	7.21E-05	-1	0.22199919	-2.1713737	1.63E-05	7.20E-05	-1	0.7000107	-0.5145511	0.25096208	0.74881109	0
ynfE	0.48887279	-1.032469	0.00176619	0.00375422	-1	0.54065022	-0.8872326	0.07901493	0.12813468	0	1.10591188	0.14523643	0.41309797	0.87131399	0
ynfF	0.51705793	-0.9516022	0.01582566	0.02701003	0	0.51456393	-0.9585778	0.04558458	0.07955697	0	0.99517655	-0.0069756	1	1	0
ynfG	0.85734771	-0.2220477	0.52052543	0.5883216	0	0.93713155	-0.0936765	0.88793104	0.91527112	0	1.09305891	0.12837115	0.54636252	0.91914725	0
ynfH	0.75547591	-0.4045424	0.36864423	0.43925049	0	0.72336659	-0.4672011	0.62178552	0.70040026	0	0.95749789	-0.0626588	0.79994492	0.96836594	0
dmsD	0.45804342	-1.1264437	0.00970772	0.01736696	-1	0.66366539	-0.591472	0.24330084	0.32858998	0	1.44891372	0.53497169	0.30216965	0.80663932	0
glnD	0.97758953	-0.0326993	0.93676905	0.95340358	0	1.23141823	0.30032083	0.27466079	0.36295636	0	1.25964752	0.33302009	0.24107367	0.73370247	0
clcB	4.66564787	2.22207743	0.000926	0.00209356	1	4.75460742	2.24932622	0.00136247	0.00362915	1	1.01906692	0.0272488	0.74916189	0.95683847	0
ynfK	0.12299407	-3.0233393	2.63E-17	3.28E-16	-1	0.21582719	-2.2120515	3.20E-08	2.58E-07	-1	1.75477721	0.81128787	0.08640476	0.42057944	0
mlc	0.28214226	-1.8255043	2.01E-07	8.88E-07	-1	0.40124427	-1.3174473	0.00146294	0.00386878	-1	1.42213362	0.50805702	0.19565158	0.67373807	0
ynfL	1.77901166	0.83107597	0.06753557	0.09871099	0	2.10662705	1.07493492	0.0137508	0.02764548	1	1.18415584	0.24385896	0.39381214	0.86854717	0
ynfM	1.68167427	0.7498983	0.03807938	0.05970771	0	2.04842577	1.03451562	0.02572233	0.04810283	1	1.21808712	0.28461732	0.58257586	0.92806272	0
asr	2.24828595	1.16882554	0.04857206	0.07405688	0	1.3884168	0.47344073	0.44660024	0.53635455	0	0.61754458	-0.6953848	0.38689318	0.86562571	0
ydgU	1.12305327	0.16742636	0.80980044	0.84660084	0	1.08730964	0.12076285	0.57463043	0.65835931	0	0.96817281	-0.0466635	0.74201626	0.95683847	0
ydgD	0.57289371	-0.8036606	0.01414167	0.02446681	0	0.26317235	-1.9259202	9.07E-06	4.32E-05	-1	0.45937377	-1.1222596	0.00730912	0.07409931	0
mdtI	1.11661345	0.15912984	0.75093989	0.7979075	0	2.02769111	1.0198379	0.16852055	0.2433635	0	1.81592933	0.86070806	0.2438332	0.74056777	0
mdtJ	1.54409969	0.6267659	0.5600729	0.62549735	0	2.94244597	1.55701593	0.12398428	0.18635596	0	1.90560622	0.93025003	0.2982972	0.80359844	0
map	0.19698591	-2.3438356	3.92E-12	3.07E-11	-1	0.22400578	-2.1583922	8.56E-09	7.75E-08	-1	1.13716648	0.18544347	1	1	0
tqsA	0.98601734	-0.0203151	0.95986974	0.97130461	0	1.03776659	0.05348199	0.62097667	0.69984594	0	1.0524831	0.07379707	0.60943369	0.92806272	0
pntB	0.28560989	-1.8078822	6.85E-08	3.23E-07	-1	0.36572913	-1.4511526	0.00015692	0.00053465	-1	1.28051985	0.35672961	0.40314761	0.86854717	0
pntA	0.16128628	-2.6323044	6.98E-16	7.69E-15	-1	0.17380062	-2.5244949	3.46E-10	3.95E-09	-1	1.07759086	0.10780952	0.64624543	0.93208609	0
ydgH	0.17261126	-2.5344015	4.89E-13	4.18E-12	-1	0.20409253	-2.2927047	2.78E-08	2.26E-07	-1	1.18238247	0.24169678	0.61925963	0.92897598	0
ydgI	2.95596186	1.56362765	0.00015044	0.00040567	1	3.07504686	1.62060839	0.00020433	0.00067283	1	1.04028638	0.05698074	0.66397951	0.93720148	0
folM	1.56661706	0.64765258	0.10531517	0.14628211	0	1.42748185	0.5134724	0.31320697	0.40316388	0	0.91118748	-0.1341802	0.78050396	0.9635071	0
ydgC	1.86412533	0.89849886	0.07618706	0.10958307	0	0.97023728	-0.0435905	0.73420778	0.7959922	0	0.52047856	-0.9420893	0.28943991	0.79166374	0
rstA	1.09585477	0.13205662	0.90305076	0.92482616	0	1.13379227	0.18115634	0.89697838	0.92143128	0	1.03461909	0.04909972	0.79224156	0.96566757	0
rstB	1.31219636	0.39198363	0.29877014	0.36657437	0	0.98935592	-0.0154385	0.95804234	0.97114015	0	0.75396941	-0.4074221	0.33400002	0.83424743	0
tus	1.16069781	0.21499241	0.70699323	0.7568563	0	0.94792221	-0.0771594	1	1	0	0.81668303	-0.2921518	0.68635121	0.94389281	0

tff	0.02134353	-5.5500571	5.04E-50	5.06E-48	-1	0.0962459	-3.3771311	5.10E-18	1.94E-16	-1	4.50937012	2.17292593	5.49E-08	4.84E-06	1
fumC	0.966287	-0.0494763	0.8637996	0.89228317	0	0.60818542	-0.7174169	0.11243043	0.17173524	0	0.62940453	-0.6679405	0.12765073	0.53511379	0
fumA	0.54239804	-0.8825761	0.00244784	0.00502656	0	0.39852702	-1.3272506	0.000168	0.00056717	-1	0.73475011	-0.4446744	0.15048096	0.58623767	0
manA	0.14853878	-2.7510884	1.68E-15	1.79E-14	-1	0.20093018	-2.3152338	2.63E-08	2.15E-07	-1	1.35271189	0.43585459	0.20577726	0.68468503	0
ydgA	0.75438048	-0.4066357	0.27738084	0.34405817	0	0.27700168	-1.8520334	0.00233383	0.00583656	-1	0.36719094	-1.4453976	0.0158537	0.13068188	0
uidC	7.32068341	2.87197834	8.89E-09	4.78E-08	1	6.08130772	2.60438159	8.24E-07	4.93E-06	1	0.83070219	-0.2675967	0.89727596	0.99640384	0
uidB	6.17410084	2.62622905	0.00040886	0.00099228	1	6.74843367	2.75455269	0.0004958	0.00145964	1	1.09302291	0.12832364	0.6712469	0.93979017	0
uidA	2.14872692	1.10348215	0.02539269	0.04137201	1	2.67566878	1.41989954	0.00764409	0.01661546	1	1.24523445	0.31641739	0.41560816	0.87131399	0
uidR	0.38476534	-1.3779492	0.00020835	0.00054325	-1	0.55501888	-0.8493913	0.01333426	0.02690598	0	1.44248666	0.52855798	0.52302131	0.91269012	0
hdhA	1.62940375	0.70434414	0.22641621	0.28820761	0	0.24615515	-2.0223602	0.00297372	0.00716514	-1	0.15107069	-2.7267043	6.93E-05	0.00201037	-1
malI	0.72293096	-0.4680702	0.28254648	0.34958201	0	0.80816279	-0.3072822	0.59247625	0.67383221	0	1.11789761	0.16078805	0.67569608	0.94091029	0
rpsB	0.0259005	-5.2708765	4.65E-48	4.28E-46	-1	0.10052146	-3.3144245	2.39E-17	8.08E-16	-1	3.88106333	1.95645197	7.94E-08	6.87E-06	1
malX	0.54760745	-0.868786	0.02498872	0.04075893	0	0.59357042	-0.7525089	0.18075266	0.25691412	0	1.08393414	0.1162771	0.54248629	0.91914725	0
malY	0.40149461	-1.3165475	0.00055012	0.00130359	-1	0.45106455	-1.1485942	0.0253339	0.04747689	-1	1.12346352	0.16795328	0.46123576	0.88879788	0
add	0.26168379	-1.9341035	2.19E-09	1.27E-08	-1	0.40306268	-1.3109239	0.00062036	0.0017941	-1	1.54026612	0.62317964	0.08733943	0.4232603	0
ydgJ	0.68586632	-0.5440007	0.11298018	0.15575326	0	0.66084258	-0.5976215	0.17299548	0.24828419	0	0.96351514	-0.0536208	0.95680718	1	0
blr	0.7322045	-0.4496815	0.16934503	0.22328268	0	0.72641866	-0.4611268	0.2915882	0.38122137	0	0.99209805	-0.0114454	1	1	0
cnu	0.6972085	-0.5203379	0.55647871	0.62273475	0	0.38893737	-1.3623902	0.30993698	0.4004712	0	0.55784944	-0.8420523	0.56611132	0.92590304	0
ydgK	0.66354082	-0.5917429	0.30325566	0.37084171	0	1.39806085	0.48342716	0.86364186	0.89629718	0	2.10697037	1.07517003	0.28609586	0.78944262	0
rsxA	1.27606853	0.35170581	0.34981264	0.41975616	0	2.59898698	1.37794941	0.01262231	0.02568046	1	2.03671427	1.0262436	0.06510599	0.35126976	0
rsxB	1.49665047	0.58173734	0.1977511	0.25584845	0	1.61398164	0.69062416	0.23339309	0.31802777	0	1.07839583	0.10888683	0.87871982	0.98956956	0
rsxC	1.69710827	0.76307861	0.01307008	0.02280038	0	2.88613511	1.52913884	0.00023984	0.0007776	1	1.70061932	0.76606023	0.07555396	0.38519417	0
tsf	0.09399939	-3.4112047	9.59E-25	2.05E-23	-1	0.1315826	-2.9259593	2.59E-15	6.39E-14	-1	1.39982394	0.48524539	0.49519954	0.90539057	0
rsxD	2.49950381	1.32164172	0.00060132	0.00141277	1	2.4815903	1.31126495	0.00326757	0.00780508	1	0.99283318	-0.0103768	0.95266587	1	0
rsxG	1.29951839	0.37797705	0.35720192	0.42769338	0	1.22106352	0.28813826	0.42282135	0.51345083	0	0.93962774	-0.0898388	1	1	0
rsxE	1.05695787	0.07991787	0.85422908	0.88405105	0	1.18483297	0.24468369	0.66931403	0.74220795	0	1.12098411	0.16476582	0.63888194	0.93163024	0
nth	1.89743385	0.92404959	0.09167647	0.12920708	0	2.44649266	1.29071495	0.16207883	0.2352139	0	1.28936915	0.36666537	0.92768023	1	0
dtpA	0.63761228	-0.6492487	0.02234056	0.03682025	0	1.05389551	0.07573183	0.8282337	0.86955684	0	1.65287831	0.72498051	0.0899022	0.4328261	0
gstA	0.54630856	-0.8722121	0.0530466	0.08010491	0	0.26563842	-1.9124643	0.00103872	0.00286701	-1	0.48624246	-1.0402522	0.05409349	0.3120921	0
pdxY	0.45488518	-1.1364257	0.0007991	0.00183168	-1	0.50498189	-0.9856965	0.03064561	0.05642622	0	1.11013044	0.1507292	0.6128703	0.92897598	0
tyrS	0.2852425	-1.8097392	2.02E-08	1.03E-07	-1	0.42566119	-1.2322225	0.00116213	0.00316223	-1	1.49227831	0.57751662	0.12271314	0.52159375	0
pdxH	0.40168382	-1.3158677	0.0003349	0.00082824	-1	0.41072132	-1.2837683	0.00162162	0.00422521	-1	1.02249903	0.03209948	0.83764932	0.98067952	0
mliC	0.41795664	-1.2585748	0.00560249	0.01057982	-1	0.58171919	-0.7816052	0.21721943	0.29966213	0	1.39181709	0.47696963	0.34387602	0.84269271	0
pyrH	0.1780852	-2.4893605	2.47E-12	1.98E-11	-1	0.33535463	-1.5762406	0.0002174	0.0007132	-1	1.88311341	0.91311989	0.01309929	0.11394058	0
anmK	0.5535442	-0.8532296	0.02688365	0.04356019	0	0.58520725	-0.7729804	0.22363075	0.30757058	0	1.05720058	0.08024913	0.49959836	0.90541644	0
slyB	0.23615731	-2.0821799	1.68E-11	1.24E-10	-1	0.10876439	-3.2007218	2.64E-16	7.40E-15	-1	0.46055906	-1.1185419	0.00088932	0.01526037	-1
slyA	0.1807717	-2.4677593	1.57E-13	1.38E-12	-1	0.25710331	-1.9595799	5.02E-08	3.93E-07	-1	1.42225424	0.50817938	0.52325739	0.91269012	0
ydhI	1.25820255	0.33136419	0.93688739	0.95340358	0	1.13269414	0.17975835	0.7991401	0.84624972	0	0.90024785	-0.1516058	0.73965304	0.95683847	0
ydhJ	1.42011085	0.50600355	0.43252784	0.50408324	0	1.60484461	0.68243362	0.10524873	0.16319092	0	1.13008405	0.17643007	0.40916541	0.86959974	0
ydhK	2.99358452	1.581874	7.78E-06	2.72E-05	1	2.33077929	1.2208124	0.00172401	0.00446043	1	0.77859144	-0.3610616	0.49636987	0.90539057	0
sodC	2.29368192	1.19766534	0.17697083	0.23209031	0	0.30203184	-1.7272275	0.13595283	0.20194136	0	0.13167991	-2.9248928	0.0097932	0.0919608	0
ydhF	0.21180567	-2.2391869	4.36E-11	3.09E-10	-1	0.15213738	-2.7165534	1.31E-10	1.58E-09	-1	0.7182876	-0.4773665	0.26366586	0.762024	0
ydhL	0.74047096	-0.4334849	0.52409381	0.59174907	0	0.26293814	-1.9272046	0.06209075	0.10408915	0	0.35509582	-1.4937197	0.1613536	0.60662351	0
nemR	1.21416509	0.2799646	0.45211878	0.52236689	0	1.15483922	0.20769202	0.43202411	0.52290182	0	0.95113855	-0.0722726	0.84027652	0.98067952	0
frr	0.17466189	-2.5173632	8.84E-15	8.91E-14	-1	0.15675394	-2.6734264	1.97E-12	3.06E-11	-1	0.89747079	-0.1560631	0.38523016	0.86368327	0
nemA	0.79632866	-0.3285641	0.38114239	0.45255536	0	0.83433343	-0.261304	0.48210183	0.57084888	0	1.04772498	0.06726007	0.97650074	1	0
gloA	0.13696672	-2.8681027	8.64E-16	9.45E-15	-1	0.12519607	-2.9977388	5.22E-12	7.63E-11	-1	0.91406197	-0.1296361	0.71185683	0.95223057	0
rnt	0.79549003	-0.3300842	0.28305504	0.35011317	0	1.16526029	0.22065225	0.8139312	0.85759704	0	1.4648333	0.55073649	0.28182441	0.78561671	0
lhr	1.77531957	0.82807875	0.02922036	0.04684803	0	1.82577273	0.86850719	0.02199778	0.04202689	0	1.02841919	0.04042844	0.65532101	0.93664426	0
grxD	0.22444515	-2.1555652	2.71E-09	1.56E-08	-1	0.33366017	-1.5835486	1.32E-05	6.02E-05	-1	1.48660003	0.57201655	0.56298245	0.92432422	0
mepH	0.61237937	-0.7075024	0.06320015	0.09323817	0	0.90741869	-0.1401597	0.49726242	0.58488225	0	1.48179172	0.56734268	0.43544411	0.88087171	0
sodB	0.06335488	-3.9804005	8.79E-32	3.26E-30	-1	0.04563134	-4.4538313	4.21E-26	5.63E-24	-1	0.72024978	-0.4734308	0.15563468	0.59527229	0
ydhP	3.62903056	1.85958421	1.38E-05	4.58E-05	1	3.12754325	1.64502984	0.00013428	0.00046723	1	0.86181232	-0.2145544	1	1	0
ynhF	0.2128194	-2.2322984	0.00014957	0.00040382	-1	0.32942656	-1.6019712	0.00331228	0.00790333	-1	1.54791603	0.63032721	0.85459345	0.98236299	0
purR	0.30954147	-1.6917954	1.86E-05	6.01E-05	-1	0.5809564	-0.7834982	0.06932777	0.11456971	0	1.87682901	0.90829722	0.05365196	0.31132257	0
sokC	0.52949985	-0.9172978	0.24458188	0.30830998	0	0.27044131	-1.8866125	0.06741388	0.11182505	0	0.5107486	-0.9693147	0.31911195	0.81866417	0
dxr	0.79806548	-0.325421	0.42178829	0.49417477	0	0.80911017	-0.3055919	0.54738282	0.63172753	0	1.01383934	0.01982905	0.92734187	1	0
ydhB	0.83665064	-0.2573028	0.61318705	0.67407845	0	0.64759044	-0.6268464	0.34747365	0.43808447	0	0.7740273	-0.3695436	0.59698536	0.92806272	0
ydhC	1.15302519	0.20542403	0.58676227	0.65020797	0	1.03982073	0.05633483	0.8681078	0.90008643	0	0.90181962	-0.1490892	0.75029577	0.95683847	0
cfa	0.63090328	-0.6645092	0.02182103	0.0360312	0	0.23971738	-2.0605936	1.99E-08	1.66E-07	-1	0.379959	-1.3960843	5.01E-05	0.0015544	-1
ribC	0.2226724	-2.1670053	2.74E-10	1.80E-09	-1	0.21536556	-2.2151405	1.21E-07	8.77E-07	-1	0.9671857	-0.0481352	0.87287025	0.98802818	0
mdtK	1.01449142	0.02075666	0.92336612	0.94263926	0	1.04976174	0.07006192	0.80745602	0.85294982	0	1.0347665	0.04930526	0.79190761	0.96552738	0
ydhQ	1.38025042	0.46493004	0.31704625	0.38588958	0	0.50648995	-0.9813945	0.04698571	0.08151937	0	0.36695511	-1.4463245	0.00310755	0.03860365	-1
valV	2.77127927	1.4705521	0.53632748	0.603864	0	7.82377769	2.96786538	0.29783985	0.38756016	0	2.82316466	1.49731328	0.58328477	0.92806272	0
valW	0.44724984	-1.1608471	0.790791	0.8310575	0	8.90343954	3.15436278	0.18866846	0.26690769	0	19.9070826	4.31520991	0.09940989	0.45857491	0
ydhR	0.09921831	-3.3332498	1.89E-21	3.14E-20	-1	0.11083387	-3.1735293	3.25E-15	7.72E-14	-1	1.11707073	0.15972053	0.70747252	0.95005571	0
ydhS	3.84964687	1.94472611	5.58E-05	0.00016584	1	1.2560107	0.32884875	0.57550575	0.65919127	0	0.32626647	-1.6158774	0.0043435	0.0492412	-1
ispU	0.27877306	-1.8428369	9.29E-08	4.30E-07	-1	0.67023762	-0.5772554	0.07574476	0.12373977	0	2.404241	1.26558152	0.0035403	0.04277461	1
ydhT	3.19684185	1.67664737	0.00015081	0.00040617	1	3.21258928	1.68373655	0.00035061	0.00108118	1	1.00492593	0.00708917	0.75934655	0.95807677	0
ydhU	2.40809773	1.26789395	0.00740117	0.0136326	1	1.97023914	0.97837075	0.06168934	0.10353406	0	0.81817242	-0.2895232	0.69868962	0.94869712	0
ydhX	1.24932341	0.321147	0.65831736	0.71409327	0	1.46424635	0.5501583	0.24784527	0.33350434	0	1.17203147	0.2290113	0.48502631	0.90075713	0
ydhW	0.29590007	-1.7568181	0.00307283	0.00616725	-1	0.51186987	-0.966151	0.11100693	0.17003106	0	1.72987412	0.79066706	0.33785124	0.83714543	0
ydhV	0.51326759	-0.9622169	0.00745702	0.01372402	0	0.53182781	-0.9109689	0.10284371	0.16043118	0	1.0361609	0.05124805	0.53219205	0.91785957	0
ydhY	0.25916949	-1.9480322	4.63E-05	0.00013989	-1	0.33063545	-1.5966867	0.01048075	0.0219012	-1	1.27574988	0.3513455	0.342715	0.84269271	0
ydhZ	0.4432659	-1.1737557	0.0638408	0.09402629	0	0.21557376	-2.2137465	0.00775358	0.01682863	-1	0.48633057	-1.0399908	0.20964253	0.69013336	0
pykF	1.38243952	0.46721636	0.33504718	0.40489833	0	0.46067204	-1.1181881	0.00344331	0.00818946	-1	0.33323124	-1.5854044	0.00011673	0.00302816	-1
lpp	0.27711045	-1.851467	0.00036397	0.00089166	-1	0.38659796	-1.3710941	0.00528495	0.01196423	-1	1.39510423	0.48037291	0.93412949	1	0
ldtE	8.68505315	3.11853648	2.32E-07	1.02E-06	1	1.0243297	0.03468016	0.80299009	0.84910581	0	0.11794167	-3.0838545	9.22E-06	0.0003911	-1
cdsA	0.53112283	-0.9128825	0.00902154	0.01629114	0	0.71678469	-0.4803883	0.18901028	0.26727566	0	1.34956482	0.43249427	0.35938898	0.85183882	0
sufE	5.45838776	2.44847489	2.56E-05	8.09E-05	1	2.34895235	1.23201745	0.09511248	0.15018072	0	0.43033812	-1.2164574	0.0471767	0.2865692	0
sufS	6.05666582	2.59852381	3.75E-08	1.86E-07	1	2.47450259	1.30713855	0.0105856	0.0220754	1	0.40855855	-1.2913853	0.01753788	0.14117431	0
sufD	6.57185327	2.71630027	2.44E-09	1.41E-08	1	2.0438175	1.03126638	0.03752152	0.06731937	0	0.31099561	-1.6850339	0.00123691	0.01983559	-1
sufC	7.87266901	2.97685282	8.07E-12	6.18E-11	1	2.29978972	1.20150195	0.02547877	0.04770791	1	0.29212326	-1.7753509	0.00017666	0.00420433	-1
sufB	7.44318312	2.89591973	1.87E-08	9.57E-08	1	2.05858034	1.04164975	0.0886206	0.1411732	0	0.27657258	-1.85427	0.00118177	0.01911846	-1
sufA	6.31823017	2.6595205	2.00E-07	8.84E-07	1	1.50993787	0.59448918	0.4368175	0.52711539	0	0.23898114	-2.0650313	0.00027988	0.00608026	-1
rydB	3.02443918	1.59666765	0.07482554	0.10783178	0	1.5928439	0.67160489	0.5964055	0.67716237	0	0.52665761	-0.9250628	0.35216013	0.84775072	0
ydiH	1.00992725	0.01425138	0.90575012	0.92694584	0	0.9443457	-0.082613	0.57093707	0.65480688	0	0.93506309	-0.0968644	0.60680549	0.92806272	0
menI	0.41812152	-1.2580058	0.00157394	0.00338631	-1	0.49204541	-1.0231366	0.04275531	0.07542344	0	1.17680001	0.23486917	0.58006362	0.92806272	0
ydiJ	0.48488154	-1.0442958	0.00360781	0.00711415	-1	0.59711658	-0.7439155	0.1110056	0.17003106	0	1.23146898	0.30038029	0.33407731	0.83424743	0
rseP	0.30034496	-1.7353076	3.73E-07	1.59E-06	-1	0.48929376	-1.0312272	0.00278481	0.00676902	-1	1.62910591	0.7040804	0.16998811	0.62286269	0
ydiK	1.01932953	0.02762052	0.91766455	0.93761839	0	1.008541	0.01226974	0.95067619	0.96575588	0	0.98941606	-0.0153508	0.98153796	1	0
rprA	1.58444558	0.66397811	0.77732466	0.81982881	0	2.15433482	1.10724248	0.42682651	0.51746127	0	1.35967738	0.44326438	0.63001116	0.93109012	0
ydiL	1.33032144	0.41177488	0.44725426	0.51742328	0	1.96052553	0.97124043	0.15286399	0.22360343	0	1.47372316	0.55946554	0.40348435	0.86854717	0
ydiM	6.26895798	2.64822566	3.66E-06	1.36E-05	1	5.90310054	2.56147291	2.56E-05	0.00010767	1	0.94163983	-0.0867527	0.86411711	0.98520074	0
ydiN	9.78254588	3.29020997	0.00766197	0.01406021	1	9.68869527	3.2763024	0.00800071	0.01728012	1	0.99040632	-0.0139076	0.80022629	0.96844071	0
ydiB	2.09399168	1.06625571	0.13181894	0.17865733	0	2.47656791	1.30834218	0.02973994	0.05480434	0	1.18270189	0.24208647	0.45019362	0.88755478	0
aroD	0.57176026	-0.8065177	0.04969844	0.0755916	0	0.70966448	-0.494791	0.38620995	0.47698763	0	1.24119238	0.31172674	0.42162795	0.87417925	0
ydiF	7.4006391	2.88764986	7.17E-10	4.46E-09	1	9.53847436	3.25375853	1.12E-09	1.18E-08	1	1.28887171	0.36610867	0.41306918	0.87131399	0
ydiO	8.52440264	3.09159874	1.15E-09	6.89E-09	1	7.70049662	2.94495149	2.34E-07	1.59E-06	1	0.90334736	-0.1466472	0.91210937	1	0
ydiP	0.61979726	-0.6901317	0.09244946	0.13013042	0	0.78533807	-0.3486143	0.59255758	0.67383221	0	1.26708863	0.34151744	0.41471342	0.87131399	0
bamA	0.15973046	-2.6462886	4.23E-16	4.72E-15	-1	0.18842201	-2.4079606	2.19E-10	2.56E-09	-1	1.17962475	0.238328	0.65581322	0.93664426	0
ydiQ	3.276968	1.71236159	0.01539575	0.02636798	1	3.16210183	1.66088383	0.02245347	0.04271283	1	0.96494742	-0.0514778	0.86038233	0.98424873	0

ydiR	4.70735096	2.23491542	8.04E-07	3.29E-06	1	5.92495764	2.56680484	4.80E-07	3.02E-06	1	1.2586607	0.33188942	0.40288969	0.86854717	0
ydiS	6.34463706	2.66553764	0.00034861	0.00085975	1	6.17099009	2.62550198	0.00143692	0.00380453	1	0.97263091	-0.0400357	1	1	0
ydiT	4.37617841	2.12967156	0.09611934	0.13478067	0	7.15927144	2.83981278	0.01098005	0.02281186	1	1.63596425	0.71014122	0.31166743	0.81168136	0
fadK	3.63498966	1.86195126	6.83E-05	0.000199	1	5.31398227	2.40979341	2.13E-05	9.13E-05	1	1.46189749	0.54784215	0.32417521	0.82445944	0
ppsA	0.21938659	-2.1884527	1.05E-11	7.94E-11	-1	0.57811541	-0.7905706	0.00977435	0.02065655	0	2.63514464	1.39788215	0.00062746	0.01168659	1
ppsR	0.41079234	-1.2835188	0.00027262	0.00068849	-1	0.61308656	-0.7058373	0.04471885	0.07829342	0	1.49244888	0.57768152	0.28855641	0.79106754	0
aroH	1.79486374	0.84387432	0.0508224	0.07722137	0	1.97927124	0.98496933	0.05103674	0.0873832	0	1.10274178	0.14109501	0.79150332	0.96530134	0
ydiE	2.21546746	1.14761114	0.02240671	0.03688798	1	1.4856245	0.57106952	0.43825557	0.5282872	0	0.67056932	-0.5765416	0.27285014	0.77568242	0
ydiU	2.2536004	1.17223172	0.0663238	0.0971005	0	1.69033028	0.75730517	0.37686521	0.46801345	0	0.75005768	-0.4149266	0.47780436	0.89855925	0
skp	0.12479627	-3.0023533	9.63E-20	1.39E-18	-1	0.16124832	-2.632644	2.83E-12	4.32E-11	-1	1.29209245	0.3697093	0.54756505	0.91914725	0
ydiV	1.46985712	0.55567593	0.41677123	0.48985592	0	1.5594681	0.64105404	0.69317991	0.76235989	0	1.06096577	0.08537812	0.78668267	0.9635071	0
nlpC	0.21182659	-2.2390444	4.01E-10	2.58E-09	-1	0.18816481	-2.4099313	9.54E-09	8.51E-08	-1	0.88829642	-0.1708869	0.41211674	0.8712535	0
btuD	1.08743505	0.12092924	0.64226976	0.69908959	0	1.39604994	0.48135055	0.314623	0.40486868	0	1.28380075	0.36042131	0.48816688	0.90208042	0
btuE	0.92556726	-0.1115903	0.76988193	0.81431237	0	0.69590637	-0.5230349	0.21207462	0.29359579	0	0.75187012	-0.4114446	0.2968043	0.80055472	0
btuC	1.39453632	0.47978551	0.12240143	0.16717597	0	1.43797343	0.52403702	0.14008004	0.20730532	0	1.03114807	0.04425151	0.80773075	0.96990832	0
ihfA	0.37500598	-1.4150145	1.51E-05	4.96E-05	-1	0.14265574	-2.8093903	7.34E-12	1.05E-10	-1	0.38040923	-1.3943758	7.79E-05	0.00221717	-1
pheT	0.23431978	-2.0934493	2.87E-11	2.08E-10	-1	0.17489469	-2.5154416	1.64E-10	1.96E-09	-1	0.74639318	-0.4219923	0.26955858	0.76991797	0
pheS	0.14287701	-2.8071543	2.76E-15	2.90E-14	-1	0.29108242	-1.7805004	3.34E-05	0.00013634	-1	2.03729357	1.02665389	0.00368322	0.04389999	1
pheM	0.85762732	-0.2215772	0.82721427	0.86086605	0	1.51301112	0.59742259	0.75829901	0.81459554	0	1.7641825	0.81899981	0.62127426	0.92938245	0
rplT	0.04629354	-4.4330454	5.23E-38	2.89E-36	-1	0.13406979	-2.8989439	4.19E-15	9.79E-14	-1	2.8960801	1.53410151	0.00012803	0.0032636	1
lpxD	0.29051362	-1.7833223	6.84E-09	3.74E-08	-1	0.35342035	-1.500543	2.47E-05	0.00010392	-1	1.21653626	0.28277932	0.58561664	0.92806272	0
rpmI	0.04781107	-4.3865116	1.20E-36	5.62E-35	-1	0.13261478	-2.9146865	5.65E-15	1.29E-13	-1	2.77372552	1.47182503	0.00030459	0.00652057	1
infC	0.1254418	-2.99491	1.32E-20	2.00E-19	-1	0.21300958	-2.2310097	5.61E-10	6.18E-09	-1	1.69807503	0.76390021	0.07305905	0.37771441	0
thrS	0.23321451	-2.1002706	5.06E-12	3.95E-11	-1	0.35554612	-1.4918914	3.77E-05	0.00015195	-1	1.52454547	0.60837918	0.07521022	0.3838855	0
arpB	4.89672833	2.29181816	0.00010039	0.00027994	1	4.51115098	2.17349557	0.00235833	0.0058845	1	0.92125817	-0.1183226	0.83996232	0.98067952	0
yniD	1.57827864	0.65835193	0.33215921	0.40173801	0	2.34066846	1.2269206	0.15558501	0.22698255	0	1.48305147	0.56856867	0.50724054	0.90925684	0
ydiY	2.01994107	1.01431321	0.02064561	0.03424396	1	2.94419166	1.55787159	0.00248684	0.00612904	1	1.45756314	0.54355838	0.25149968	0.74990776	0
pfkB	2.04103857	1.02930344	0.00171498	0.00365539	1	0.93099301	-0.1031578	0.70024921	0.76841352	0	0.4561369	-1.1324612	0.00267402	0.03488882	-1
ydiZ	5.06139625	2.33953543	1.53E-09	9.01E-09	1	1.54733326	0.62978395	0.61374101	0.69239763	0	0.30571273	-1.7097515	8.14E-06	0.00034837	-1
yniA	0.11161716	-3.1633692	1.11E-21	1.87E-20	-1	0.05588651	-4.1613562	1.64E-22	1.32E-20	-1	0.50069814	-0.997987	0.00860594	0.08380111	0
yniB	0.75235474	-0.410515	0.3285579	0.39792713	0	0.50746399	-0.9786227	0.12227217	0.18459882	0	0.67450095	-0.5681076	0.38691578	0.86562571	0
fabZ	0.31042052	-1.6877042	5.81E-08	2.78E-07	-1	0.36455059	-1.4558091	4.97E-05	0.00019428	-1	1.17437659	0.23189511	0.76857612	0.96147692	0
yniC	0.43159576	-1.2122474	0.00630729	0.01177484	-1	0.63457964	-0.6561269	0.23002942	0.31480334	0	1.47031019	0.55612055	0.27723553	0.77922796	0
ydjM	1.11557678	0.15778981	0.67115704	0.72485894	0	2.74202774	1.45524317	0.14561768	0.21428057	0	2.45794623	1.29745335	0.21108904	0.69212096	0
ydjN	0.81951051	-0.2871656	0.42565284	0.49803625	0	0.57566307	-0.7967034	0.10409766	0.16203155	0	0.70244745	-0.5095378	0.30403792	0.80892599	0
ydjO	4.35213594	2.12172362	0.02576243	0.04192801	1	6.02863659	2.59183177	0.0043867	0.01011207	1	1.3852133	0.47010815	0.39476785	0.86854717	0
cedA	0.87965819	-0.184985	0.72780507	0.77668881	0	1.42942942	0.51543939	0.68400525	0.7556548	0	1.62498279	0.70042444	0.4824134	0.89935549	0
katE	2.20461955	1.14052971	0.00061915	0.00144928	1	1.53597912	0.6191586	0.10045442	0.15708	0	0.69670938	-0.5213711	0.1963877	0.67373807	0
chbG	0.83397186	-0.2619294	0.82183633	0.85715491	0	0.78199399	-0.3547706	0.81162395	0.85639118	0	0.93767431	-0.0928412	1	1	0
chbF	2.02075649	1.01489548	0.01138855	0.02009717	1	2.19434303	1.13378907	0.00743244	0.01620323	1	1.08590176	0.11889359	0.59506607	0.92806272	0
chbR	0.58193978	-0.7810582	0.12575266	0.1713901	0	0.54495547	-0.8757898	0.23456389	0.31913111	0	0.93644649	-0.0947315	1	1	0
chbA	0.52274459	-0.9358219	0.11730066	0.16111366	0	0.4245219	-1.2360891	0.09629199	0.15164444	0	0.81210195	-0.3002672	0.76669658	0.96109492	0
lpxA	0.26962873	-1.8909539	7.59E-10	4.67E-09	-1	0.32092851	-1.6396761	1.71E-05	7.47E-05	-1	1.19026082	0.25127775	0.59691303	0.92806272	0
chbC	1.5502535	0.63250414	0.13767243	0.18573583	0	1.49292973	0.57814626	0.09368694	0.14820076	0	0.96302297	-0.0543579	0.76217064	0.95858924	0
chbB	0.46814189	-1.0949822	0.00330889	0.00658647	-1	0.25036658	-1.9978861	6.66E-06	3.25E-05	-1	0.53480919	-0.9029038	0.01830821	0.14495371	0
osmE	2.06316408	1.04485856	0.22035305	0.28138174	0	0.37919334	-1.3989945	0.11005049	0.16891737	0	0.18379214	-2.443853	0.00964651	0.09109449	0
nadE	0.3496715	-1.5159279	9.30E-06	3.21E-05	-1	0.31002139	-1.6895603	4.07E-05	0.00016276	-1	0.88660754	-0.1736325	0.63129784	0.93151288	0
cho	1.39947663	0.48488739	0.2758405	0.34233984	0	2.47328465	1.30642829	0.01157709	0.02388384	1	1.76729258	0.8215409	0.09482449	0.44916863	0
ves	4.92524677	2.30019601	0.00035061	0.00086323	1	4.45750788	2.15623735	0.00107525	0.00295494	1	0.90503239	-0.1439587	0.92408582	1	0
spy	0.72988125	-0.4542663	0.36004211	0.43062714	0	0.37465975	-1.4163471	0.04724862	0.0819112	0	0.51331604	-0.9620807	0.18393386	0.65309848	0
astE	11.8534746	3.56723812	1.12E-10	7.55E-10	1	6.01016942	2.58740566	1.53E-06	8.68E-06	1	0.50703862	-0.9798325	0.23017201	0.71837124	0
astB	12.1777312	3.60617347	2.69E-21	4.38E-20	1	5.24734631	2.39158801	9.77E-10	1.03E-08	1	0.43089687	-1.2145855	0.01141712	0.1031752	0
astD	14.3467932	3.8426564	2.54E-23	4.76E-22	1	7.38500292	2.88459849	4.84E-13	8.20E-12	1	0.51474938	-0.9580579	0.05112163	0.3009965	0
lpxB	0.66710716	-0.5840096	0.04873157	0.07427445	0	0.86723219	-0.2055098	0.48016086	0.56977197	0	1.29998934	0.3784998	0.36550304	0.85342842	0
astA	26.5053968	4.72821423	1.44E-30	4.98E-29	1	13.0720711	3.70841583	2.05E-18	8.40E-17	1	0.49318526	-1.0197984	0.04352788	0.27028009	0
astC	20.1037352	4.32939167	1.56E-17	1.98E-16	1	9.8966039	3.30693354	2.31E-09	2.29E-08	1	0.49227687	-1.0224581	0.06536175	0.35158639	0
xthA	0.45484052	-1.1365673	0.00060454	0.00141883	-1	0.82118786	-0.2842158	0.34461651	0.43510296	0	1.80544128	0.8523515	0.05310058	0.30934421	0
ydjX	0.5928594	-0.7542381	0.08665643	0.12283743	0	0.91032456	-0.1355471	0.95593173	0.97020264	0	1.53548138	0.61869102	0.15807074	0.59990703	0
ydjY	0.23789663	-2.0715932	9.04E-09	4.86E-08	-1	0.54132062	-0.8854447	0.0439939	0.07717707	0	2.27544466	1.1861485	0.00239953	0.03206651	1
ydjZ	0.28626932	-1.8045551	1.43E-05	4.72E-05	-1	0.89784869	-0.1554558	0.72555589	0.78990674	0	3.13637765	1.64909928	0.00043834	0.00862986	1
ynjA	0.61496277	-0.701429	0.12311798	0.16805071	0	1.7924628	0.84194317	0.17954988	0.25586291	0	2.91475011	1.5433722	0.0055985	0.06036529	0
ynjB	0.73634627	-0.4415437	0.57951887	0.64347281	0	1.89271198	0.92045489	0.02314664	0.04380513	0	2.57041023	1.36199863	0.00254414	0.0335918	1
ynjC	1.34403844	0.4265744	0.36617981	0.43670928	0	1.87521503	0.90705604	0.0283101	0.0523879	0	1.39520937	0.48048164	0.16312554	0.6114388	0
ynjD	0.62852869	-0.6699495	0.25296819	0.31761242	0	0.80894568	-0.3058853	0.82903566	0.86998541	0	1.28704654	0.36406423	0.43015755	0.87809207	0
nhaA	1.02156598	0.03078239	0.89358545	0.91747256	0	5.6001725	2.48547127	3.88E-05	0.00015609	1	5.48194889	2.45468888	1.17E-05	0.00046947	1
rnhB	0.88719913	-0.1726701	0.83335479	0.86630457	0	1.10651798	0.14602689	0.52452844	0.61158078	0	1.24720363	0.31869703	0.37374212	0.85857675	0
ynjE	0.15191979	-2.7186183	7.59E-14	6.97E-13	-1	0.35891668	-1.4782791	0.00025932	0.00083159	-1	2.36254068	1.24033917	0.00236699	0.03193373	1
ynjF	1.53500452	0.6182429	0.10056312	0.14062189	0	2.05875473	1.04177196	0.03778891	0.06777159	0	1.34120434	0.42352906	0.42069739	0.87375001	0
nudG	4.885946	2.28863792	2.07E-05	6.63E-05	1	4.18137554	2.06397762	0.00089296	0.0024943	1	0.85579651	-0.2246603	0.80744519	0.96990832	0
ynjH	1.60984741	0.68692395	0.4020358	0.47455695	0	0.92213579	-0.1169489	0.84102506	0.87964254	0	0.57280944	-0.8038728	0.30120241	0.80552009	0
gdhA	1.05166324	0.0726728	0.62707692	0.68729498	0	1.07912013	0.10985548	0.78842921	0.83852435	0	1.02610807	0.03718268	0.96623772	1	0
ynjI	5.39090715	2.43052806	4.52E-09	2.53E-08	1	6.52068862	2.70502433	1.45E-07	1.03E-06	1	1.20957168	0.27449627	0.70855254	0.95069307	0
topB	0.40605328	-1.300259	0.00011226	0.00030952	-1	0.48784088	-1.0355175	0.00651975	0.01443427	-1	1.20142084	0.26474159	0.57936338	0.92806272	0
selD	0.2941725	-1.7652657	2.71E-08	1.35E-07	-1	0.31299783	-1.6757754	6.11E-06	3.01E-05	-1	1.06399418	0.08949026	0.96553726	1	0
ydjA	0.46797004	-1.0955119	0.00374538	0.00735915	-1	0.38238705	-1.3868944	0.0043674	0.01007808	-1	0.81711867	-0.2913825	0.66746171	0.93861876	0
sppA	0.54298218	-0.8810233	0.00686446	0.0127236	0	0.72527525	-0.4633995	0.32250354	0.4129969	0	1.33572571	0.41762379	0.19316854	0.66866033	0
dnaE	0.80998085	-0.3040403	0.42512383	0.49768671	0	1.01287285	0.01845308	0.92514246	0.9450311	0	1.25048988	0.32249338	0.40204825	0.86854717	0
ansA	1.10879744	0.14899583	0.61109258	0.67221818	0	1.35991729	0.44351891	0.2616829	0.34863258	0	1.22647947	0.29452308	0.4577441	0.88755478	0
pncA	0.55147763	-0.8586257	0.06333884	0.09341157	0	0.86424291	-0.2104912	0.70197667	0.76934692	0	1.56714047	0.6481345	0.21514256	0.69763138	0
ydjE	4.24235226	2.08486442	0.0010635	0.0023678	1	4.55327743	2.18690537	0.00163023	0.00424012	1	1.07329075	0.10204095	0.74599136	0.95683847	0
ydjF	0.35399853	-1.4981847	0.0002421	0.0006212	-1	0.30337271	-1.7208368	0.00085553	0.00239581	-1	0.85698864	-0.222652	0.74104799	0.95683847	0
ydjG	2.35309287	1.23455826	0.00793541	0.01449573	1	2.26825503	1.18158286	0.0234825	0.04440273	1	0.96394624	-0.0529754	1	1	0
ydjH	5.14800996	2.36401484	0.00106185	0.00236639	1	3.68512613	1.881714	0.01833443	0.03577035	1	0.71583508	-0.4823008	0.60532303	0.92806272	0
ydjI	9.62436295	3.26669105	1.12E-13	1.01E-12	1	4.99134316	2.31942809	8.71E-06	4.17E-05	1	0.51861543	-0.947263	0.04750477	0.28816509	0
ydjJ	7.80160302	2.96377059	1.74E-11	1.29E-10	1	8.9589766	3.16333394	5.12E-10	5.67E-09	1	1.14835074	0.19956335	0.63966374	0.93163024	0
ydjK	6.4710455	2.69399882	3.47E-05	0.00010715	1	5.37074689	2.42512273	0.00027551	0.00087587	1	0.82996587	-0.2688761	1	1	0
ydjL	4.71031582	2.23582379	1.23E-08	6.49E-08	1	5.80239097	2.53664751	3.34E-08	2.67E-07	1	1.23184754	0.30082372	0.46112587	0.88879788	0
accA	0.23939721	-2.0625217	4.77E-10	3.03E-09	-1	0.32569596	-1.6184023	2.22E-05	9.46E-05	-1	1.36048353	0.44411949	0.30836218	0.81055096	0
yeaC	0.53764876	-0.8952641	0.00339355	0.00673373	0	0.30301557	-1.7225362	2.79E-05	0.00011641	-1	0.56359391	-0.8272721	0.03375871	0.22797859	0
msrB	0.51767855	-0.9498715	0.00225728	0.00467217	0	0.36378623	-1.4588372	5.29E-05	0.00020498	-1	0.7027261	-0.5089656	0.0760195	0.38578363	0
gapA	0.16576568	-2.5927828	5.05E-17	6.17E-16	-1	0.26604621	-1.9102513	2.74E-08	2.23E-07	-1	1.6049535	0.6825315	0.12250706	0.52159375	0
yeaD	0.51051083	-0.9699865	0.00137931	0.00299971	0	0.58567675	-0.7718235	0.05062768	0.08688299	0	1.14723668	0.19816306	0.51499994	0.90937129	0
yeaE	0.79228206	-0.335914	0.17460289	0.22956161	0	0.68777066	-0.5400005	0.13535808	0.20129292	0	0.86808815	-0.2040865	0.6004825	0.92806272	0
mipA	0.10104875	-3.3068766	9.01E-22	1.52E-20	-1	0.26284065	-1.9277397	8.11E-07	4.86E-06	-1	2.60112713	1.37913691	0.00024214	0.00539317	1
yeaG	4.18675132	2.06583123	0.00295243	0.00596019	1	0.7277262	-0.4585323	0.51453722	0.60183695	0	0.17381644	-2.5243636	0.0019544	0.02807459	-1
yeaH	4.04009141	2.01438794	1.18E-06	4.74E-06	1	1.07244652	0.1009057	0.94737906	0.96329223	0	0.26545105	-1.9134822	4.26E-05	0.00135248	-1
yeaI	12.6770015	3.66414164	1.62E-08	8.40E-08	1	9.85147926	3.30034037	1.15E-05	5.33E-05	1	0.77711431	-0.3638013	0.53697312	0.9187668	0
yeaJ	2.09520264	1.06708978	0.00961818	0.01723469	1	1.62700928	0.70222248	0.18336792	0.26021286	0	0.7765403	-0.3648673	0.43704505	0.88087171	0
ldcC	3.69844169	1.88691753	9.55E-09	5.12E-08	1	1.36497168	0.44887102	0.11218081	0.17141321	0	0.3690667	-1.4380465	0.00070635	0.01297926	-1
yeaK	0.93547541	-0.0962284	0.62412369	0.6843978	0	0.67669858	-0.5634147	0.07284533	0.11962226	0	0.72337398	-0.4671864	0.13100357	0.53993058	0
yoaI	8.98399409	3.16735698	6.81E-06	2.41E-05	1	4.93573659	2.3032654	0.02216541	0.04229229	1	0.54939223	-0.8640916	0.12650917	0.5319293	0
yeaL	26.5691592	4.73168067	3.90E-11	2.80E-10	1	12.3941128	3.6315831	6.53E-05	0.00024608	1	0.46648495	-1.1000976	0.05319671	0.30937079	0

yeaM	1.67565699	0.74472685	0.04988605	0.07585084	0	1.14525389	0.19566746	0.41755465	0.50803248	0	0.68346559	-0.5490594	0.40905775	0.86959974	0
yeaN	2.19022927	1.1310819	0.00707504	0.01308097	1	3.11386038	1.63870426	0.00049901	0.00146812	1	1.42170522	0.50762236	0.2153711	0.69785935	0
yeaO	0.28373424	-1.8173878	5.31E-07	2.22E-06	-1	0.23997622	-2.0590367	2.36E-06	1.28E-05	-1	0.84577813	-0.2416488	0.41952159	0.87375001	0
yoaF	0.46566199	-1.102645	0.0454156	0.06994446	0	0.48452615	-1.0453536	0.05463715	0.0927195	0	1.04051041	0.0572914	0.84782426	0.98167799	0
yeaP	0.65256202	-0.6158131	0.09113975	0.12853265	0	1.19396782	0.25576396	1	1	0	1.82966185	0.87157704	0.15498597	0.59433748	0
yoaK	0.8721947	-0.1972779	0.57110795	0.6360524	0	1.03768389	0.05336702	0.85963644	0.89399438	0	1.18973881	0.25064488	0.88159013	0.99128314	0
yoaJ	1.91767517	0.93935837	0.60496806	0.66703543	0	2.77501489	1.47249551	0.29314562	0.38314418	0	1.44707244	0.53313715	0.59363316	0.92806272	0
yaeR	2.71442706	1.44064772	0.00240841	0.00495249	1	0.79883717	-0.3240266	0.73382267	0.79583961	0	0.29429311	-1.7646743	0.00283556	0.03645714	-1
yeaQ	1.79711957	0.8456864	0.48026122	0.55027595	0	0.453439	-1.1410196	0.19541091	0.27456942	0	0.25231432	-1.986706	0.05250956	0.30699184	0
yoaG	1.66080243	0.73188046	0.54896911	0.61574316	0	1.39247344	0.47764981	0.8513335	0.88769296	0	0.83843413	-0.2542306	0.71465515	0.9525362	0
yeaR	1.83127448	0.87284804	0.03657967	0.05747862	0	1.18517849	0.24510435	0.79270681	0.84146384	0	0.64718779	-0.6277437	0.14278625	0.57181814	0
leuE	1.43597043	0.52202604	0.52695293	0.59421779	0	1.57913803	0.65913728	0.24808394	0.33368455	0	1.09970094	0.13711124	0.63996324	0.93163024	0
dmlR	4.22556182	2.07914317	4.42E-10	2.83E-09	1	1.02166262	0.03091885	0.38191887	0.47314255	0	0.24178149	-2.0482243	2.93E-09	4.03E-07	-1
dmlA	0.59331084	-0.75314	0.05923047	0.08802859	0	0.65662756	-0.6068528	0.34783473	0.43841462	0	1.10671761	0.14628716	0.46524874	0.89206388	0
yeaV	6.68865331	2.74171577	4.34E-06	1.59E-05	1	6.13197924	2.61635281	2.57E-05	0.00010768	1	0.91677337	-0.125363	0.86960404	0.98621446	0
yeaW	3.96041048	1.98564997	0.00011427	0.00031427	1	4.47679343	2.16246575	6.09E-05	0.00023119	1	1.13038622	0.17681578	0.49524279	0.90539057	0
yeaX	3.48936154	1.80296309	0.0164099	0.02788862	1	2.7430023	1.45575583	0.03516165	0.06362791	0	0.78610435	-0.3472073	0.9519265	1	0
rnd	0.73961918	-0.4351455	0.24828519	0.31244321	0	1.35415537	0.43739328	0.31620097	0.40618956	0	1.8308819	0.87253874	0.04073554	0.25960079	0
tilS	2.1713531	1.11859435	0.00124617	0.00273983	1	1.45052902	0.53657916	0.1266289	0.1898789	0	0.66803	-0.5820152	0.21383542	0.69446947	0
fadD	2.67702187	1.42062893	8.89E-05	0.00025076	1	1.12642998	0.17175764	0.68676004	0.75774655	0	0.42077728	-1.2488713	0.00302805	0.03804477	-1
yeaY	0.59390709	-0.7516908	0.02553639	0.0415908	0	0.6749322	-0.5671855	0.13702267	0.20318899	0	1.13642725	0.18450533	0.7377363	0.95683847	0
tsaB	0.93479358	-0.0972803	0.59387137	0.65643889	0	2.20172385	1.13863353	0.03208292	0.05870576	0	2.35530486	1.23591381	0.00798179	0.07927863	0
yoaA	1.78625041	0.83693434	0.01624888	0.02765389	0	2.48428901	1.31283302	0.00125865	0.0033832	1	1.39078429	0.47589868	0.21090555	0.69203383	0
yoaB	0.09211141	-3.4404763	1.04E-19	1.50E-18	-1	0.2725198	-1.875567	4.45E-07	2.82E-06	-1	2.95858899	1.56490929	0.0029759	0.03766955	1
yoaC	1.8887327	0.91741854	0.04315907	0.06681865	0	0.46161675	-1.1152325	0.00305628	0.00734405	-1	0.24440555	-2.0326511	3.74E-06	0.00018258	-1
yoaH	1.29789696	0.37617585	0.66863735	0.72297948	0	0.73741731	-0.4394468	0.34113649	0.43132537	0	0.56816321	-0.8156227	0.15682034	0.59825059	0
pabB	1.81730936	0.86180403	0.03269784	0.05193325	0	1.56292127	0.64424511	0.11662615	0.17722522	0	0.86001938	-0.2175589	0.77500831	0.96302807	0
nudL	1.00030595	0.00044133	0.76922273	0.81381002	0	0.87636155	-0.1904019	0.85453062	0.88997662	0	0.87609351	-0.1908432	0.94297771	1	0
sdaA	0.57283966	-0.8037967	0.00954934	0.01712523	0	0.63038549	-0.6656938	0.07749881	0.12609251	0	1.10045712	0.13810294	0.69903718	0.94869712	0
rof	0.46346447	-1.1094693	0.00212211	0.00442976	-1	0.16517271	-2.5979527	1.12E-08	9.90E-08	-1	0.356387	-1.4884834	0.00047037	0.00913804	-1
yoaD	3.66833099	1.87512382	0.00033095	0.00081985	1	2.5035268	1.3239619	0.00569174	0.01278038	1	0.68247026	-0.5511619	0.62872846	0.93109012	0
yoaE	0.63526713	-0.6545647	0.07187661	0.10402325	0	0.6621345	-0.5948038	0.15710761	0.2286001	0	1.04229301	0.05976091	0.89458893	0.9954996	0
manX	0.05456096	-4.195987	4.41E-35	1.93E-33	-1	0.02423022	-5.3670489	1.76E-34	6.48E-32	-1	0.44409437	-1.1710618	0.00096739	0.0162831	-1
manY	0.07005503	-3.8353676	4.53E-30	1.46E-28	-1	0.04132594	-4.5968085	1.09E-27	1.72E-25	-1	0.58990688	-0.7614409	0.02162746	0.16501225	0
manZ	0.1006127	-3.3131157	1.05E-23	2.02E-22	-1	0.06645583	-3.9114604	7.93E-22	5.47E-20	-1	0.66051138	-0.5983447	0.06200335	0.33924908	0
yobD	0.27453705	-1.8649272	6.12E-05	0.00018048	-1	0.30924532	-1.6931763	0.00201065	0.0051005	-1	1.12642474	0.17175092	0.7660385	0.96081621	0
mntP	2.69173707	1.42855713	0.00980767	0.01753866	1	2.90099675	1.53654868	0.00913756	0.01943162	1	1.0777415	0.10801118	0.71262481	0.95223057	0
rlmA	1.194629	0.25656265	0.46868055	0.53840364	0	2.42695516	1.27914746	0.01987792	0.03840862	1	2.03155553	1.0225848	0.06289786	0.3424439	0
cspC	0.04957433	-4.3342629	7.68E-36	3.50E-34	-1	0.12621398	-2.9860564	7.16E-17	2.18E-15	-1	2.54595432	1.34820653	0.00926114	0.08802074	0
yobF	0.03107022	-5.0083238	7.52E-44	5.83E-42	-1	0.09696177	-3.3664402	3.00E-20	1.56E-18	-1	3.12073026	1.64188366	0.00173097	0.02597927	1
yaeP	0.35002977	-1.5144505	2.80E-05	8.76E-05	-1	0.09831772	-3.3464048	3.16E-13	5.57E-12	-1	0.28088387	-1.8319543	1.03E-05	0.00043047	-1
yebO	0.86146406	-0.2151375	0.6479314	0.70438419	0	1.31921099	0.39967532	1	1	0	1.53135929	0.61481281	0.76115154	0.95858924	0
mgrB	1.12795586	0.17371062	0.81051747	0.84714994	0	1.16759947	0.22354546	0.73944895	0.79881412	0	1.03514642	0.04983484	0.60480041	0.92806272	0
yobH	0.36547185	-1.4521678	0.23315301	0.29567081	0	1.95958538	0.97054843	0.75395773	0.81094672	0	5.36179562	2.42271623	0.17569962	0.63459078	0
kdgR	0.24516136	-2.0281965	8.73E-09	4.70E-08	-1	0.28766821	-1.7975223	6.04E-06	2.98E-05	-1	1.17338314	0.23067417	0.81161749	0.96998188	0
yebQ	4.87582983	2.28564777	5.43E-09	3.00E-08	1	5.70975745	2.51342946	3.89E-08	3.08E-07	1	1.17103296	0.22778169	0.57344482	0.92806272	0
htpX	2.7090397	1.43778154	0.01640493	0.02788862	1	2.10283225	1.07233377	0.4005878	0.4913121	0	0.77622792	-0.3654478	0.27216377	0.77484973	0
prc	0.55873074	-0.8397749	0.00790364	0.01444965	0	0.60166479	-0.7329682	0.04244577	0.07496718	0	1.07684211	0.10680674	0.86961433	0.98621446	0
proQ	0.23630604	-2.0812716	2.43E-10	1.60E-09	-1	0.37871999	-1.4007965	0.00017902	0.00059839	-1	1.60266739	0.68047505	0.09096902	0.43700805	0
msrC	0.70029412	-0.5139671	0.35288695	0.42310034	0	0.92995364	-0.1047693	0.63982904	0.71743327	0	1.32794723	0.40919782	0.74355773	0.95683847	0
yebS	1.85648846	0.89257635	0.02069321	0.03429715	0	1.73568165	0.79550236	0.1275474	0.19099694	0	0.93492725	-0.097074	0.69028022	0.94554543	0
yaeQ	0.60661861	-0.7211383	0.03799271	0.05961414	0	0.67260048	-0.5721783	0.28060396	0.36948418	0	1.10876993	0.14896004	0.53648965	0.9187668	0
yebT	1.98436286	0.98867586	0.0032547	0.00649323	0	1.83846206	0.8784994	0.01890908	0.03674548	0	0.92647474	-0.1101765	0.86575022	0.98578839	0
rsmF	2.35114983	1.23336648	0.00039216	0.00095594	1	2.5144969	1.33026978	0.00256755	0.00629285	1	1.0694754	0.0969033	0.85613542	0.98270619	0
yebV	5.04483964	2.33480841	0.00011118	0.00099625	1	2.40602102	1.26664924	0.21530442	0.29750788	0	0.47692715	-1.0681592	0.08048418	0.40105677	0
yebW	1.7962127	0.8449582	0.10291095	0.14334836	0	1.9587755	0.96995206	0.39685935	0.48768673	0	1.09050309	0.12499386	0.71898888	0.95399541	0
pphA	6.33512076	2.66337212	8.05E-11	5.51E-10	1	5.17945051	2.37279905	3.49E-06	1.81E-05	1	0.81757723	-0.2905731	0.36308301	0.85329623	0
ryeA	0.42689879	-1.228034	0.14750589	0.19773399	0	0.9865495	-0.0195367	0.60480857	0.68511896	0	2.31096812	1.20849736	0.47962808	0.89897261	0
yebY	6.47145172	2.69408938	0.00250879	0.00513976	1	4.86966071	2.28382126	0.08608126	0.13794141	0	0.75248351	-0.4102681	0.42042747	0.87375001	0
yebZ	21.3432351	4.41570696	1.16E-05	3.92E-05	1	20.6843155	4.37046531	0.00056009	0.00163438	1	0.96912748	-0.0452416	0.6917124	0.94599088	0
yobA	61.0347496	5.93155896	2.89E-09	2.14E-08	1	48.1188911	5.58853149	2.80E-06	1.49E-05	1	0.78838517	-0.3430275	0.44932303	0.88755478	0
arfB	0.94078866	-0.0880574	0.83462725	0.86721914	0	0.58569527	-0.7717779	0.19772304	0.2768284	0	0.62255775	-0.6837204	0.23937552	0.73308752	0
holE	1.53690189	0.62002507	0.2970795	0.365007	0	1.56911097	0.64994739	0.42319756	0.51376648	0	1.02095715	0.02992231	0.9119104	1	0
yobB	1.72739997	0.78860217	0.06943902	0.10089215	0	1.98292356	0.98762906	0.18868966	0.26690769	0	1.14792381	0.1990269	0.91813736	1	0
exoX	1.34303416	0.425496	0.27118853	0.33751472	0	1.49908781	0.58408489	0.17024306	0.24528989	0	1.11619485	0.15858889	0.6557031	0.93664426	0
ptrB	1.52717847	0.61086867	0.29908259	0.36685582	0	0.61810367	-0.6940793	0.31017734	0.40054754	0	0.40473571	-1.3049479	0.03764287	0.24408514	0
yebE	0.73216938	-0.4497507	0.20254136	0.26158631	0	0.64184302	-0.6397076	0.19814467	0.27717673	0	0.87663188	-0.189957	0.78098811	0.9635071	0
yebF	1.44778188	0.53384426	0.30749569	0.3755069	0	0.90347411	-0.1464448	0.53027227	0.61681316	0	0.62404021	-0.6802891	0.11857759	0.51429541	0
yebG	0.3757496	-1.4121565	4.05E-05	0.00012352	-1	0.49848957	-1.0043648	0.01107608	0.02300056	-1	1.32665361	0.40779174	0.40007255	0.86854717	0
purT	2.80681947	1.48893627	0.00264513	0.00539157	1	3.40669458	1.76837261	0.00078027	0.00220179	1	1.21372059	0.27943634	0.41960872	0.87375001	0
eda	0.27087752	-1.8842874	1.19E-08	6.28E-08	-1	0.30788042	-1.699558	1.63E-05	7.20E-05	-1	1.13660383	0.18472948	0.65244728	0.93479289	0
edd	0.64191118	-0.6395544	0.06508975	0.09553668	0	0.72278022	-0.4683711	0.39927892	0.49011487	0	1.12598166	0.17118333	0.46143756	0.88879788	0
nhaR	0.72217877	-0.4695721	0.1776201	0.23280355	0	3.19153297	1.67424955	0.00248307	0.00612532	1	4.41931153	2.14382163	1.72E-05	0.00064833	1
nlpE	0.45803991	-1.1264548	0.00168367	0.0035909	-1	0.42619358	-1.2304192	0.00520904	0.01180448	-1	0.93047259	-0.1039644	0.80352826	0.96977549	0
zwf	0.35308375	-1.5019177	1.25E-05	4.20E-05	-1	0.29781072	-1.7475324	5.82E-06	2.88E-05	-1	0.84345633	-0.2456147	0.30845289	0.81055096	0
yebK	0.11788989	-3.0844881	8.60E-19	1.19E-17	-1	0.2441926	-2.0339086	3.35E-08	2.68E-07	-1	2.07136167	1.05057948	0.03275993	0.22329409	0
pykA	0.07968206	-3.6496012	3.20E-27	8.31E-26	-1	0.09006169	-3.4729426	1.40E-17	4.95E-16	-1	1.13026308	0.17665861	0.57945016	0.92806272	0
lpxM	1.56331965	0.6446128	0.05982565	0.08885337	0	1.64284173	0.7161935	0.21175356	0.29333511	0	1.05086745	0.0715807	0.79957454	0.96818334	0
mepM	0.44610354	-1.1645495	0.00035516	0.00087202	-1	0.81679638	-0.2919516	0.37388316	0.46527449	0	1.83095698	0.8725979	0.02865111	0.20346441	0
znuA	0.50677083	-0.9805946	0.04507382	0.06946652	0	1.14835596	0.19956991	0.85123283	0.88769296	0	2.26602616	1.18016452	0.12379874	0.52495428	0
znuC	0.52682649	-0.9246002	0.05514962	0.08282757	0	1.04921166	0.06930575	0.68551874	0.75694835	0	1.9915697	0.99390597	0.19923126	0.67679424	0
znuB	1.22095485	0.28800985	0.43445243	0.5057924	0	1.34003117	0.42226656	0.24160821	0.32670339	0	1.09752721	0.13425671	0.6033506	0.92806272	0
ruvB	0.43427261	-1.2033271	0.00036148	0.00088654	-1	0.57477959	-0.7989193	0.05033946	0.08648914	0	1.32354557	0.40440787	0.31871048	0.81858662	0
ruvA	0.44808904	-1.1581427	0.00208297	0.00436249	-1	0.49324451	-1.0196251	0.03221679	0.05890195	0	1.10077343	0.13851756	0.712316	0.95223057	0
yaeF	0.88533767	-0.1757003	0.77871715	0.82090541	0	1.33550382	0.4173841	0.48043555	0.56983699	0	1.5084683	0.59308438	0.48224316	0.89935549	0
yobI	2.31728729	1.21243692	0.63580367	0.69374066	0	4.30127882	2.10476565	0.35676258	0.4480062	0	1.85616986	0.89232874	0.61749326	0.92897598	0
yebB	6.72690135	2.7499421	0.00016484	0.00043888	1	3.93716174	1.97715598	0.00344982	0.00820052	1	0.58528608	-0.7727861	0.58844332	0.92806272	0
ruvC	0.10899105	-3.1977185	7.92E-17	9.55E-16	-1	0.20121238	-2.3132091	4.13E-08	3.25E-07	-1	1.84613676	0.88450943	0.07267378	0.37684625	0
yebC	0.08355791	-3.5810798	3.93E-24	7.93E-23	-1	0.19361172	-2.3687618	9.07E-10	9.62E-09	-1	2.31709629	1.212318	0.00346954	0.04215062	1
nudB	0.19798088	-2.3365087	1.33E-09	7.95E-09	-1	0.28982902	-1.786726	9.04E-05	0.00032858	-1	1.46386516	0.54978267	0.21024796	0.69080725	0
aspS	0.22907751	-2.1260923	4.20E-11	2.98E-10	-1	0.34640036	-1.5294877	5.86E-05	0.00022359	-1	1.51215353	0.59660462	0.09912708	0.458344	0
yecD	0.47985873	-1.0593184	0.01846531	0.03094131	-1	0.53539625	-0.9013211	0.12301052	0.18533362	0	1.11573723	0.1579973	0.58835568	0.92806272	0
yecE	1.43520943	0.52126127	0.34939912	0.41948788	0	1.59975252	0.67784874	0.26343257	0.35042399	0	1.11464745	0.15658747	0.78299234	0.9635071	0
yecN	0.50718181	-0.9794251	0.00710441	0.01312429	0	0.5653898	-0.8226822	0.03557728	0.06424846	0	1.1147675	0.15674285	1	1	0
cmoA	0.9426554	-0.0851976	0.86137882	0.89019893	0	0.76497475	-0.386516	0.40745086	0.49807064	0	0.81151049	-0.3013184	0.48318463	0.89935549	0
proS	0.3298162	-1.6002658	2.50E-07	1.09E-06	-1	0.44758669	-1.159761	0.00078156	0.00220403	-1	1.35707917	0.44050489	0.33761818	0.83714543	0
cmoB	0.81085253	-0.3024885	0.32369275	0.39311269	0	0.89749479	-0.1560245	0.66748298	0.74075638	0	1.10685328	0.146464	0.74775164	0.95683847	0
torZ	4.63913336	2.21385532	3.00E-05	9.35E-05	1	3.96580179	1.98761257	0.00026762	0.00085313	1	0.85485833	-0.2262427	0.98422401	1	0
torY	3.72395701	1.89683642	0.00259589	0.0052961	1	3.83987	1.94105747	0.00260603	0.00637302	1	1.0311263	0.04422105	0.68785813	0.94389281	0
cutC	0.54636377	-0.8720663	0.01105298	0.01955969	0	0.58415264	-0.7755827	0.03992572	0.07113511	0	1.0691643	0.09648358	1	1	0
yecM	0.78507783	-0.3490924	0.60528921	0.6670565	0	0.87141932	-0.198561	0.83934344	0.87892385	0	1.10997825	0.1505314	0.77313415	0.9628697	0
argS	0.40481204	-1.3046759	0.00021099	0.00054916	-1	0.93486288	-0.0971733	0.66316205	0.73686525	0	2.3093752	1.20750259	0.00452927	0.05095423	0

yecT	1.72477813	0.78641079	0.56742376	0.63258726	0	1.59908538	0.67724697	0.60854128	0.68793653	0	0.92712527	-0.1091638	0.92727507	1	0
flhE	0.66976745	-0.5782678	0.34890806	0.4190122	0	1.03848712	0.05448333	0.9537555	0.96866136	0	1.55051894	0.63275115	0.43666451	0.88087171	0
flhA	0.25119864	-1.9930995	1.43E-08	7.45E-08	-1	0.31761515	-1.6546484	0.00017696	0.00059453	-1	1.26439838	0.33845109	0.22318937	0.70531966	0
flhB	0.21147025	-2.2414734	5.06E-09	2.81E-08	-1	0.40299283	-1.3111739	0.00296911	0.00715793	-1	1.90567148	0.93029943	0.02849286	0.20284423	0
tsaA	1.03203618	0.04549354	0.95398054	0.96800644	0	1.60083025	0.67882034	0.21316325	0.2948258	0	1.55113773	0.63332679	0.2224348	0.70531966	0
cheZ	1.50867818	0.59328509	0.22631052	0.28815612	0	1.67386479	0.74318299	0.09512465	0.15018072	0	1.10949095	0.1498979	0.52602487	0.91473568	0
cheY	1.03867745	0.05474771	0.84742695	0.87845221	0	1.11459624	0.15652119	0.52484318	0.61175864	0	1.07309179	0.10177348	0.6373423	0.93163024	0
cheB	4.31817234	2.11042082	8.39E-06	2.93E-05	1	4.90957072	2.29559688	1.39E-05	6.27E-05	1	1.13695572	0.18517606	0.56852701	0.92771214	0
cheR	5.79482619	2.53476539	8.77E-05	0.00024772	1	5.454454	2.44743479	0.00046611	0.00138236	1	0.94126274	-0.0873306	1	1	0
tap	2.72105396	1.44416557	0.00560079	0.01057982	1	3.73084977	1.89950427	0.00076507	0.00216582	1	1.37110466	0.4553387	0.30682703	0.81011621	0
tar	3.16195258	1.66081573	3.73E-06	1.38E-05	1	3.33123454	1.73605693	2.68E-05	0.00011183	1	1.05353716	0.0752412	0.73952522	0.95683847	0
cheW	1.09177563	0.12667639	0.66087895	0.71616838	0	1.21236331	0.27782209	0.43498549	0.52540062	0	1.11045097	0.1511457	0.64683097	0.93208609	0
cheA	3.19015777	1.67362776	1.52E-05	5.00E-05	1	3.70691429	1.89021876	1.26E-05	5.76E-05	1	1.16198464	0.21659099	0.46939386	0.89538835	0
motB	2.51280411	1.32929821	0.00514412	0.00978113	1	4.58993751	2.19847451	8.99E-05	0.00032751	1	1.82661971	0.86917631	0.09831414	0.45734597	0
motA	3.24606142	1.6986903	0.00403007	0.00786254	1	2.84717138	1.50952934	0.00372811	0.00877238	1	0.87711568	-0.189161	0.81008285	0.96990832	0
rcsF	0.46477275	-1.1054026	0.00987683	0.01765519	-1	0.71630638	-0.4813513	0.21523338	0.29750276	0	1.54119733	0.62405131	0.33206548	0.83208693	0
flhC	0.90165865	-0.1493467	0.60344751	0.66585752	0	1.25554087	0.32830899	0.70083421	0.76867371	0	1.39247915	0.47765573	0.40007373	0.86854717	0
flhD	0.17850647	-2.4859518	1.17E-09	7.03E-09	-1	1.04959991	0.0698395	0.7631085	0.81829081	0	5.87989851	2.55579125	2.10E-07	1.61E-05	1
insB1	0.12433987	-3.0076391	0.80870128	0.84585213	0	0.77629558	-0.365322	1	1	0	6.24333602	2.64231711	0.71504293	0.9525362	0
uspC	0.37781678	-1.4042413	7.45E-05	0.00021521	-1	0.35583045	-1.4907381	0.00063595	0.00183557	-1	0.94180691	-0.0864968	0.69588334	0.94834535	0
otsA	3.86052943	1.94879871	0.00909132	0.01640373	1	0.81396978	-0.2969529	0.8254826	0.86728664	0	0.21084408	-2.2457516	0.01519884	0.12646579	0
otsB	5.25683292	2.39419388	0.00421871	0.00817283	1	1.4601376	0.54610433	0.49505083	0.58274631	0	0.27775994	-1.8480896	0.06597076	0.3524804	0
araH	166.372856	7.37827626	1.15E-07	5.26E-07	1	208.52374	7.70406783	9.66E-07	5.72E-06	1	1.25335193	0.32579157	1	1	0
araG	2.72537281	1.44645359	0.00122639	0.00270038	1	3.94020452	1.97827052	0.00045882	0.00136718	1	1.44574882	0.53181692	0.3915833	0.86854717	0
araF	1.03151815	0.0447692	0.86842573	0.89601412	0	0.84782157	-0.2381674	0.81808816	0.86115568	0	0.82191629	-0.2829366	0.69179605	0.94599088	0
metQ	0.23823298	-2.0695549	4.59E-10	2.93E-09	-1	0.1958606	-2.3521009	2.00E-08	1.66E-07	-1	0.82213889	-0.282546	0.52492766	0.91318777	0
ftnB	0.10092072	-3.3087056	2.67E-18	3.66E-17	-1	0.1104155	-3.1789854	4.10E-13	7.07E-12	-1	1.09408154	0.12972027	0.88625768	0.99349171	0
yecJ	1.95615535	0.96802095	0.06889628	0.10016948	0	2.96617856	1.56860545	0.06276641	0.10506242	0	1.51633077	0.6005845	0.6354351	0.93163024	0
azuC	2.27885115	1.18830669	0.34038857	0.40978367	0	3.60836812	1.85134653	0.25388904	0.3399805	0	1.58341545	0.66303984	0.72699674	0.95487077	0
yecR	0.40033	-1.3207384	0.00013711	0.00037132	-1	0.21061416	-2.2473257	2.01E-06	1.11E-05	-1	0.52610136	-0.9265873	0.04646074	0.28417734	0
ftnA	0.03558289	-4.8126725	4.21E-37	2.07E-35	-1	0.08263401	-3.5971205	7.16E-17	2.18E-15	-1	2.32229621	1.215552	0.00182273	0.02670518	1
yecH	1.11483388	0.15682875	0.79448794	0.83414624	0	0.34151653	-1.5499727	6.42E-05	0.00024243	-1	0.30633849	-1.7068015	1.46E-05	0.00057539	-1
tyrP	2.06885882	1.04883519	0.02074314	0.03436701	1	2.08276926	1.05850302	0.05603237	0.09465101	0	1.00672373	0.00966782	1	1	0
yecA	0.96241133	-0.0552745	0.85732153	0.88704361	0	0.6052863	-0.7243104	0.15325954	0.22398609	0	0.62892682	-0.6690359	0.17387414	0.63109871	0
leuZ	0.40880793	-1.2905049	0.14218248	0.19129455	0	2.08529671	1.06025268	0.49440504	0.58244285	0	5.10092035	2.35075757	0.04493209	0.27636053	0
cysT	0.38701955	-1.3695217	0.07618963	0.10958307	0	1.71528483	0.77844816	0.64352536	0.7206636	0	4.43203671	2.14796983	0.04143562	0.26141785	0
metI	0.69717111	-0.5204153	0.25468966	0.31950135	0	0.73539033	-0.4434179	0.73315858	0.79544016	0	1.05482044	0.07699743	0.57475011	0.92806272	0
glyW	0.32960699	-1.6011812	0.11195131	0.15452779	0	1.75128815	0.80841648	0.72342736	0.78817196	0	5.31326149	2.40959772	0.07589293	0.38578363	0
pgsA	0.22254654	-2.167821	2.35E-10	1.55E-09	-1	0.31282929	-1.6765525	1.44E-05	6.47E-05	-1	1.40568032	0.49126854	0.36013234	0.85248719	0
uvrC	0.50946557	-0.9729435	0.00193884	0.00408971	0	0.41765862	-1.2596039	0.00110065	0.00301722	-1	0.81979754	-0.2866604	0.40888027	0.86959974	0
uvrY	0.29388596	-1.7666716	1.81E-07	8.09E-07	-1	0.35709667	-1.4856134	7.04E-05	0.00026287	-1	1.21508582	0.28105822	0.80702448	0.96990832	0
yecF	2.22787721	1.15566972	0.02048063	0.03399783	1	0.98328138	-0.0243238	0.69652544	0.76508723	0	0.44135349	-1.1799935	0.01598125	0.13124263	0
sdiA	8.95869556	3.16328868	4.41E-07	1.86E-06	1	4.89149411	2.2902752	0.01396986	0.02802215	1	0.54600517	-0.8730135	0.07384657	0.37957754	0
yecC	1.57293561	0.65345961	0.07345663	0.10610135	0	0.5700063	-0.8109502	0.18717103	0.26524234	0	0.36238375	-1.4644098	0.0046108	0.05160818	0
yecS	1.40760276	0.49324025	0.41702357	0.49002211	0	0.28219379	-1.8252419	0.00199385	0.00507059	-1	0.20047829	-2.3184821	6.93E-05	0.00201037	-1
dcyD	0.98688159	-0.0190511	0.92511155	0.94369924	0	0.33161482	-1.5924196	0.0029255	0.00707983	-1	0.3360229	-1.5733685	0.00253731	0.0335918	-1
fliY	0.52343018	-0.933931	0.03572037	0.05626851	0	0.14995749	-2.7373745	9.12E-06	4.33E-05	-1	0.28648995	-1.8034435	0.00238236	0.03193373	-1
metN	0.88712892	-0.1727843	0.57841278	0.64276247	0	0.7820833	-0.3546058	0.42578445	0.51648133	0	0.88158923	-0.1818215	0.69430241	0.94773784	0
fliZ	0.07166789	-3.8025292	1.35E-25	3.14E-24	-1	0.10719421	-3.2217011	8.40E-14	1.64E-12	-1	1.49570762	0.58082819	0.10457337	0.47522837	0
fliA	0.01482118	-6.0761959	1.23E-52	1.51E-50	-1	0.10415289	-3.2632253	4.40E-15	1.02E-13	-1	7.02730077	2.81297065	1.17E-13	4.30E-11	1
fliC	0.2457598	-2.0246791	5.82E-10	3.66E-09	-1	0.26246175	-1.9298209	2.14E-06	1.17E-05	-1	1.06796044	0.0948582	0.77758169	0.9635071	0
fliD	0.57904545	-0.7882515	0.03919314	0.06130174	0	0.91768926	-0.1239224	0.76702866	0.82117105	0	1.58483113	0.66432912	0.12415854	0.5259742	0
fliS	0.22233116	-2.1692179	3.20E-06	1.20E-05	-1	0.23624358	-2.081653	0.00020041	0.00066189	-1	1.06257519	0.08756493	0.84477866	0.98090413	0
fliT	0.32227417	-1.6336395	0.00110524	0.00245318	-1	0.36418971	-1.4572379	0.02354132	0.04449491	-1	1.13006175	0.17640161	0.69642773	0.94850102	0
amyA	2.86268436	1.51736861	0.00455188	0.00873779	1	0.95061871	-0.0730613	0.87424654	0.90538914	0	0.33207249	-1.5904299	0.01066029	0.09794145	0
yedD	0.50673537	-0.9806956	0.0156861	0.02680291	0	0.54878287	-0.8656927	0.07925553	0.12843057	0	1.08297723	0.11500291	0.7548119	0.9573569	0
yedE	0.13573185	-2.8811688	2.32E-16	2.64E-15	-1	0.56250895	-0.830052	0.01456326	0.02909361	0	4.14426641	2.05111675	4.46E-07	3.12E-05	1
yedF	0.04910666	-4.3479375	2.11E-25	4.83E-24	-1	0.15650232	-2.6757441	8.09E-10	8.72E-09	-1	3.1869876	1.67219341	0.00125516	0.02005525	1
gmhB	0.47627274	-1.0701401	0.00174607	0.00371324	-1	0.59207596	-0.7561458	0.04659723	0.08103626	0	1.24314475	0.31399429	0.57696483	0.92806272	0
yedK	2.69438445	1.42995572	0.00355126	0.00701517	1	2.44780383	1.29148794	0.02105598	0.04040224	1	0.9084835	-0.1384678	0.94207014	1	0
yedL	4.47681912	2.16247403	0.00076995	0.00177499	1	8.39760861	3.06997855	0.00012053	0.0004234	1	1.87579806	0.90750452	0.27414266	0.77647342	0
yedN	11.4024824	3.51127604	0.0042918	0.00831078	1	13.0974123	3.7112099	0.01700352	0.03340976	1	1.14864569	0.19993386	0.95249254	1	0
intG	0.57724637	-0.7927409	0.38852384	0.46020644	0	0.78864135	-0.3425587	0.71352443	0.77968953	0	1.36621275	0.45018216	0.64513204	0.93208609	0
fliE	0.19995227	-2.3222725	4.43E-06	1.62E-05	-1	0.53579835	-0.900238	0.11150757	0.17067959	0	2.67963127	1.42203449	0.01131829	0.10249207	0
fliF	0.08091535	-3.6274428	1.77E-24	3.72E-23	-1	0.1929032	-2.374051	4.19E-09	3.95E-08	-1	2.38401253	1.25339182	0.00062805	0.01168659	1
fliG	0.0191899	-5.7035092	1.69E-47	1.49E-45	-1	0.07279158	-3.7800846	1.89E-18	7.95E-17	-1	3.79322412	1.92342461	2.89E-07	2.12E-05	1
fliH	0.07866352	-3.6681615	7.40E-23	1.35E-21	-1	0.20863499	-2.260947	1.71E-07	1.20E-06	-1	2.65224582	1.4072145	0.00013169	0.00333774	1
fliI	0.16476994	-2.601475	2.34E-12	1.88E-11	-1	0.30434617	-1.7162149	1.00E-04	0.00035952	-1	1.84709767	0.88526016	0.01447936	0.12256039	0
fliJ	0.12596498	-2.9889054	1.19E-11	8.95E-11	-1	0.27354054	-1.8701734	5.56E-05	0.00021352	-1	2.17156018	1.11873194	0.04914863	0.29394474	0
rrsH	2.13824284	1.09642571	0.02336172	0.03836013	1	2.55713713	1.35452953	0.01238282	0.02526301	1	1.19590585	0.25810382	0.54202176	0.91914725	0
fliK	0.13816327	-2.8555539	9.51E-16	1.03E-14	-1	0.19275373	-2.3751693	1.67E-08	1.43E-07	-1	1.39511558	0.48038465	0.19275085	0.66826357	0
fliL	0.01793906	-5.800752	1.51E-45	1.21E-43	-1	0.10163454	-3.2985374	1.05E-14	2.31E-13	-1	5.66554451	2.50221462	1.51E-09	2.23E-07	1
fliM	0.05322857	-4.2316554	1.20E-30	4.18E-29	-1	0.11691263	-3.0964973	1.40E-13	2.63E-12	-1	2.19642632	1.1351581	0.00259404	0.03414837	1
fliN	0.05916252	-4.0791727	8.39E-27	2.12E-25	-1	0.10054753	-3.3140505	7.73E-13	1.27E-11	-1	1.69951391	0.76512217	0.01710526	0.13866582	0
fliO	0.09579665	-3.3838809	7.03E-13	5.96E-12	-1	0.14199928	-2.8160445	5.19E-08	4.05E-07	-1	1.48229897	0.56783645	0.40234947	0.86854717	0
fliP	0.28502815	-1.8108237	3.50E-05	0.00010798	-1	0.34048224	-1.5543485	0.00153015	0.0040129	-1	1.19455653	0.25647513	0.64477288	0.93208609	0
fliQ	0.49108452	-1.0259568	0.10180356	0.14198494	0	0.60171358	-0.7328512	0.34053024	0.43092873	0	1.22527501	0.29310559	0.5739099	0.92806272	0
fliR	0.42731881	-1.2266153	0.01471245	0.02531551	-1	0.69703025	-0.5207068	0.56143553	0.65441718	0	1.63117144	0.70590842	0.13003928	0.53863917	0
rcsA	0.58239559	-0.7799287	0.09191414	0.12945943	0	0.35085935	-1.5110353	0.00596361	0.01330297	-1	0.60244163	-0.7311066	0.13249259	0.54403384	0
dsrB	2.47132728	1.30528608	0.4199068	0.49249292	0	2.06845979	1.04855691	0.68010643	0.75191151	0	0.83698335	-0.2567292	0.76043368	0.95840749	0
thrB	0.35006774	-1.514294	5.28E-06	1.90E-05	-1	0.37766708	-1.4048131	0.00194218	0.00494668	-1	1.07883997	0.10948088	0.48296387	0.89935549	0
insB1	1.49411515	0.57929134	0.57280935	0.63730451	0	7.76833534	2.95760548	0.05494919	0.09314173	0	5.19928824	2.37831414	0.13410939	0.54862932	0
yodD	28.4033855	4.827991	1.18E-26	2.94E-25	1	6.31762319	2.65938189	1.90E-05	8.25E-05	1	0.22242501	-2.1686091	2.77E-07	2.07E-05	-1
dsrA	12.1244034	3.59984186	4.43E-09	2.49E-08	1	3.47956022	1.79890498	0.07690292	0.12521519	0	0.28698816	-1.8009369	0.00191351	0.02766751	-1
yedP	4.68624025	2.22843092	2.47E-06	9.44E-06	1	1.74124423	0.80011857	0.41586011	0.50654454	0	0.37156529	-1.4283123	0.00199727	0.02850468	-1
yedQ	5.59884849	2.48513014	4.54E-13	3.90E-12	1	2.73979102	1.45406586	0.00096883	0.00269431	1	0.48934902	-1.0310643	0.00392202	0.04563613	-1
yodC	10.7481348	3.42601441	0.00146727	0.00317536	1	3.65571202	1.87015243	0.10835795	0.16698087	0	0.34012525	-1.555862	0.20168255	0.67864297	0
yedI	2.71020557	1.43840228	0.00010739	0.00029757	1	2.00937011	1.00674333	0.01281079	0.02601752	1	0.74140875	-0.431659	0.39432788	0.86854717	0
yedA	8.77203682	3.13291187	9.75E-15	9.74E-14	1	8.85369557	3.14627977	4.68E-11	6.02E-10	1	1.00930898	0.0133679	0.90581116	0.99890653	0
vsr	1.45009634	0.53614875	0.19814988	0.25628931	0	1.25474649	0.32739591	0.4562358	0.54563085	0	0.86528492	-0.2087528	0.84156894	0.98067952	0
dcm	0.8633554	-0.2119735	0.42921903	0.5010202	0	0.94581615	-0.0803683	0.87344214	0.90498026	0	1.09551194	0.1316052	0.45877424	0.88755478	0
yedJ	2.46279898	1.30029888	0.0019637	0.00413424	1	2.02938602	1.02104332	0.01611201	0.0317569	1	0.82401611	-0.2792556	0.77285221	0.9628697	0
yedR	7.75126949	2.95443261	3.78E-08	1.87E-07	1	5.39164976	2.43072678	4.16E-05	0.00016572	1	0.6955828	-0.5237058	0.46988856	0.89543069	0
rseX	5.84625613	2.54751304	0.24427918	0.30801631	0	2.67903828	1.4217152	0.44665869	0.53635455	0	0.45824853	-1.1257978	0.91917582	1	0
yedS	4.05685107	2.02036034	0.0004014	0.0009763	1	1.15865558	0.21245178	0.76857178	0.82195516	0	0.28560466	-1.8079086	0.00622633	0.06561167	0
hchA	2.4959187	1.31957094	0.20463682	0.26367585	0	0.34988054	-1.5150657	0.19015349	0.26863436	0	0.14018106	-2.8346366	0.01808938	0.1442571	0
yedV	1.72582626	0.78728723	0.04165934	0.06465541	0	1.31681891	0.39705696	0.2308496	0.31563255	0	0.76300781	-0.3902303	0.66181032	0.93664426	0
yedW	2.81939804	1.49538717	0.00190664	0.00402564	1	1.97400095	0.98112269	0.02679119	0.04991158	0	0.70014979	-0.5142645	0.57727704	0.92806272	0
hiuH	0.47264692	-1.0811652	0.31440059	0.38301243	0	0.46243892	-1.1126653	0.34621238	0.4367435	0	0.97840249	-0.0315	1	1	0
yedY	1.89728679	0.92393777	0.0239636	0.03917366	0	1.36634242	0.45031909	0.2115489	0.29314349	0	0.72015598	-0.4736187	0.45301017	0.88755478	0
yedZ	1.32877507	0.41009691	0.54375618	0.61082682	0	1.53115895	0.61462406	0.19443693	0.2735488	0	1.15230861	0.20452715	0.4604485	0.88879788	0
zinT	1.10250805	0.14078919	0.95758997	0.96966412	0	0.40503986	-1.3038642	0.27237384	0.36034979	0	0.36738041	-1.4446534	0.21609644	0.69815772	0

rrlH	8.12482016	3.02233588	8.65E-05	0.00024474	1	37.266722	5.21981602	2.45E-10	2.82E-09	1	4.586775	2.19748014	0.00140798	0.02209682	1
yodB	5.12654199	2.35798601	8.29E-05	0.00023662	1	3.22903156	1.69110154	0.00942167	0.0199781	1	0.62986543	-0.6668845	0.36704305	0.85342842	0
serU	0.17759454	-2.4933409	3.83E-10	2.47E-09	-1	0.26575476	-1.9118326	1.91E-05	8.28E-05	-1	1.49641287	0.58150828	0.29890593	0.80400362	0
mtfA	0.10323542	-3.2759901	6.70E-23	1.22E-21	-1	0.22361997	-2.1608791	8.32E-10	8.92E-09	-1	2.16611674	1.115111	0.0177358	0.14235426	0
asnT	0.18186252	-2.4590798	0.03986797	0.06222502	0	0.34965426	-1.515999	0.32485179	0.41498936	0	1.92262957	0.94308083	0.47760414	0.89855925	0
yeeJ	2.3673148	1.24325157	0.00012397	0.0003376	1	2.4856778	1.3136393	0.00030942	0.00097043	1	1.04999884	0.07038774	0.72068867	0.95399541	0
yeeL	4.69157581	2.23007258	0.01565251	0.02675586	1	2.82750526	1.49952971	0.0901126	0.14298297	0	0.60267709	-0.7305429	0.664551	0.93733835	0
shiA	2.28635479	1.1930493	0.00213591	0.00445224	1	1.51953216	0.60362721	0.36509235	0.45677891	0	0.66460908	-0.5894221	0.08226631	0.40809271	0
amn	0.42277619	-1.242034	6.79E-05	0.00019826	-1	0.25248778	-1.9857145	5.87E-07	3.61E-06	-1	0.59721382	-0.7436805	0.04982466	0.29533167	0
yeeN	0.09089477	-3.4596589	3.36E-21	5.38E-20	-1	0.05054915	-4.3061692	7.10E-19	3.14E-17	-1	0.5561283	-0.8465103	0.11829313	0.51396327	0
asnW	15.3758871	3.94259775	0.00375404	0.00736959	1	12.6902948	3.66565367	0.01854139	0.03614222	1	0.8253374	-0.2769441	0.88325952	0.99247405	0
yeeO	6.18732489	2.62931579	5.23E-12	4.07E-11	1	3.60961524	1.85184507	9.37E-07	5.56E-06	1	0.58338867	-0.7774707	0.19231667	0.66728286	0
asnU	0.97916984	-0.030369	0.85789105	0.88721722	0	2.17847918	1.12332133	0.62782857	0.70666695	0	2.2248226	1.15369031	0.56508564	0.92545162	0
cbl	1.93568025	0.95284066	0.04899978	0.0745803	0	7.6954878	2.94401278	5.34E-05	0.00020653	1	3.97559865	1.99117212	0.00786203	0.07826531	0
nac	4.95385102	2.30855048	6.42E-05	0.00018839	1	17.7845239	4.15255045	3.79E-09	3.61E-08	1	3.59004012	1.84399997	0.00612253	0.06490473	0
asnV	2.54262038	1.34631608	0.77444222	0.81776507	0	1.27242481	0.34758041	1	1	0	0.50043838	-0.9987357	0.82095465	0.97480076	0
ldtA	1.37697427	0.46150161	0.54894298	0.61574316	0	0.79987053	-0.3221616	0.5276874	0.61429152	0	0.58088996	-0.7836632	0.22018487	0.70312474	0
cobT	1.41886689	0.50473925	0.11247276	0.15519905	0	1.32777503	0.40901072	0.25125425	0.33716749	0	0.93579957	-0.0957285	0.89679359	0.99640384	0
cobS	2.75860381	1.46393827	0.00048335	0.00115465	1	2.47503832	1.30745086	0.0066813	0.01474885	1	0.89720688	-0.1564874	0.88035916	0.99065677	0
cobU	1.81964329	0.86365566	0.04204606	0.06520978	0	1.6290501	0.70403098	0.13487783	0.20074945	0	0.89525794	-0.1596247	0.86030554	0.98424873	0
yeeH	5.74940237	2.523412	0.03106039	0.0495151	1	5.55943428	2.47493808	0.04616376	0.08044071	0	0.96695864	-0.0484739	0.92489023	1	0
aspU	3.52143684	1.81616421	0.39214561	0.46399871	0	2.5568368	1.35436008	0.65337807	0.72789641	0	0.72607771	-0.4618041	0.8890911	0.99411016	0
insH1	0.29035249	-1.7841227	0.85018967	0.88048952	0	2.77317676	1.47153957	0.63018467	0.70824359	0	9.55106939	3.25566227	0.53890479	0.91897962	0
yoeG	2.02268682	1.01627296	0.17615336	0.23122418	0	1.79639348	0.84510339	0.12777361	0.19124086	0	0.88812241	-0.1711696	0.8240198	0.97661485	0
yoeH	2.083057	1.05870232	0.21029767	0.27010318	0	2.64007751	1.40058029	0.303874	0.39413481	0	1.26740531	0.34187797	0.97623474	1	0
yoeA	2.50272991	1.32350261	0.00756606	0.01390718	1	1.39897917	0.48437448	0.24217062	0.3272636	0	0.55898128	-0.8391281	0.19940386	0.67679424	0
yeeP	4.9519193	2.3079878	6.98E-08	3.28E-07	1	3.34966339	1.74401613	0.00245794	0.00608668	1	0.6764374	-0.5639717	0.12664983	0.5319293	0
isrC	2.75039818	1.45964049	0.13651771	0.1843469	0	0.91865656	-0.1224025	0.77338828	0.82621337	0	0.33400857	-1.582043	0.31089864	0.81168136	0
flu	2.66589322	1.41461899	1.50E-05	4.94E-05	1	2.33932381	1.22609158	0.00019808	0.00065516	1	0.87750094	-0.1885274	0.98048873	1	0
yeeR	3.90325904	1.96467921	1.76E-07	7.89E-07	1	2.95072089	1.56106746	0.00017031	0.00057406	1	0.75596338	-0.4036117	0.48250537	0.89935549	0
dkgB	0.56452514	-0.8248903	0.03332073	0.0527897	0	0.82176389	-0.2832042	0.26102568	0.34816555	0	1.45567279	0.54168609	0.52348679	0.91269012	0
yeeS	2.57117986	1.36243053	0.0144727	0.02497106	1	3.82161096	1.93418092	0.00180059	0.00462336	1	1.4863258	0.57175039	0.2735578	0.77568242	0
yeeT	1.61822098	0.69440863	0.32635813	0.39558833	0	1.95234101	0.96520506	0.21579794	0.29800347	0	1.20647367	0.27079643	0.68774208	0.94389281	0
cbeA	4.4479759	2.15314897	0.00018642	0.00049129	1	4.41414033	2.1421325	0.00042094	0.00126538	1	0.99239304	-0.0110165	0.77618423	0.9635071	0
cbtA	2.92779312	1.54981361	0.06446583	0.09481986	0	3.04088439	1.60449097	0.06129197	0.10294535	0	1.0386268	0.05467735	0.74033505	0.95683847	0
yeeW	4.91648613	2.29762757	0.00292217	0.00590449	1	3.12481019	1.64376856	0.03456483	0.06265052	0	0.63557795	-0.653859	0.63114494	0.93151288	0
yoeD	3.65008945	1.86793182	0.46399512	0.53385425	0	2.58566502	1.37053538	0.51954918	0.60673568	0	0.70838401	-0.4973964	0.94165524	1	0
yoeF	2.13040538	1.09112798	0.29633402	0.36419237	0	1.08514896	0.1178931	0.91952339	0.9405958	0	0.50936267	-0.9732349	0.25982656	0.75892335	0
yeeX	0.13223755	-2.9187962	2.25E-19	3.18E-18	-1	0.15306517	-2.707782	2.49E-14	5.12E-13	-1	1.15750158	0.21101417	0.63055276	0.93147872	0
yeeA	5.0094572	2.32465429	6.25E-07	2.60E-06	1	3.11131603	1.63752494	0.01918617	0.03721847	1	0.62108845	-0.6871293	0.06714011	0.3563031	0
sbmC	34.0069425	5.0877574	2.67E-15	2.83E-14	1	12.7974577	3.67778533	7.42E-06	3.59E-05	1	0.37631897	-1.4099721	0.00762118	0.07673379	0
yafC	0.47957093	-1.0601839	0.00178388	0.00378634	-1	0.46302055	-1.1108519	0.00253254	0.00622083	-1	0.96548918	-0.050668	0.60025031	0.92806272	0
dacD	2.5825408	1.36879114	0.00066325	0.00154269	1	3.48829319	1.8025213	0.00016408	0.0005556	1	1.35072143	0.43373016	0.32994747	0.8313479	0
sbcB	0.43331784	-1.2065024	0.00066779	0.00155243	-1	0.4681679	-1.0949021	0.00803123	0.0173291	-1	1.08042609	0.11160038	0.81113008	0.96998188	0
yeeD	1.36718475	0.45120821	0.48970401	0.55891875	0	1.28159611	0.35794167	0.81952294	0.8618448	0	0.9373979	-0.0932665	0.7031098	0.94896888	0
yeeE	5.80098771	2.53629856	1.51E-10	1.01E-09	1	4.94935444	2.30724036	2.88E-07	1.92E-06	1	0.85319168	-0.2290582	0.68790737	0.94389281	0
plaP	0.72149903	-0.4709306	0.33699905	0.40681192	0	0.88967513	-0.1686495	0.95817403	0.97114015	0	1.23309262	0.30228117	0.40321167	0.86854717	0
yoeI	0.30596308	-1.7085705	0.18808535	0.24500017	0	0.67249804	-0.572398	0.68850915	0.758619	0	2.19797123	1.1361725	0.48434385	0.90048751	0
yeeY	2.71130031	1.43898492	0.00103524	0.00231293	1	1.68009203	0.74854026	0.18008173	0.25645542	0	0.61966283	-0.6904447	0.11878831	0.51429541	0
yeeZ	0.57020155	-0.8104561	0.01782748	0.03003203	0	0.42272353	-1.2422137	0.00132563	0.00354806	-1	0.74135808	-0.4317576	0.19752354	0.67551679	0
yoeB	0.26881311	-1.8953246	0.00062926	0.00146913	-1	0.15496926	-2.689946	0.00030812	0.00096718	-1	0.57649443	-0.7946214	0.375046	0.85919629	0
yefM	0.3660904	-1.4497282	0.0010576	0.00235931	-1	0.41186756	-1.2797476	0.0059229	0.01323888	-1	1.12504333	0.16998057	0.92342407	1	0
yafD	0.88866533	-0.1702879	0.52424749	0.59177132	0	0.61974527	-0.6902527	0.02422235	0.0456722	0	0.69738883	-0.5199648	0.06884552	0.36403925	0
hisL	63.6257832	5.99153961	7.73E-25	1.67E-23	1	17.4635386	4.12627401	2.92E-08	2.36E-07	1	0.27447267	-1.8652656	0.00010426	0.00278672	-1
hisG	0.74736022	-0.4201243	0.22322704	0.28464025	0	0.851312	-0.2322401	0.58118197	0.66376456	0	1.13909193	0.18788418	0.66860109	0.9393217	0
hisD	1.06245954	0.0874079	0.60746256	0.66910672	0	1.34314103	0.4256108	0.19514847	0.27428788	0	1.26418088	0.3382029	0.33829851	0.83714543	0
hisC	0.90768119	-0.1397424	0.64179376	0.69891594	0	1.00912346	0.0131027	0.70502275	0.77173533	0	1.1117598	0.15284513	0.4693694	0.89538835	0
hisB	0.48886229	-1.0325	0.00781328	0.01430816	-1	0.56215096	-0.8309705	0.08701173	0.13901114	0	1.14991679	0.20152947	0.56085865	0.92432422	0
hisH	0.91660091	-0.1256344	0.7031249	0.75362841	0	0.66337915	-0.5920944	0.48147146	0.57040814	0	0.72373826	-0.46646	0.63460096	0.93163024	0
hisA	0.86146401	-0.2151376	0.60038603	0.66313371	0	0.93923415	-0.0904432	0.64854784	0.72463538	0	1.09027672	0.12469434	0.38126892	0.85988523	0
hisF	0.96998022	-0.0439728	0.78994828	0.83056453	0	0.85142064	-0.232056	0.71463219	0.780514	0	0.87777113	-0.1880833	0.82054439	0.97480076	0
hisI	0.53737565	-0.8959972	0.02892061	0.04643487	0	0.64384168	-0.6352221	0.16589288	0.2401968	0	1.19812218	0.26077503	0.63930085	0.93163024	0
wzzB	0.34801987	-1.5227584	2.44E-06	9.33E-06	-1	0.24957123	-2.0024765	4.05E-07	2.60E-06	-1	0.71711775	-0.4797181	0.13342132	0.54733771	0
yafE	12.6451178	3.66050857	1.52E-10	1.01E-09	1	6.6031332	2.72315075	0.00075294	0.00213695	1	0.52218835	-0.9373578	0.03518088	0.23400856	0
ugd	5.88760433	2.55768072	3.25E-05	0.00010085	1	3.6413993	1.86449295	0.05102956	0.0873832	0	0.61848574	-0.6931878	0.13002979	0.53863917	0
gnd	0.09685296	-3.36806	9.17E-24	1.78E-22	-1	0.13676153	-2.8702656	8.49E-14	1.64E-12	-1	1.41205319	0.49779443	0.27056007	0.77178386	0
wbbL	0.06273975	-3.9944765	2.42E-26	5.91E-25	-1	0.20485795	-2.2873042	4.12E-09	3.90E-08	-1	3.26520203	1.70717226	0.00019438	0.00445561	1
wbbK	0.07668371	-3.7049361	2.05E-25	4.73E-24	-1	0.27845301	-1.8444942	8.88E-08	6.63E-07	-1	3.6311886	1.86044186	6.27E-05	0.00188236	1
wbbJ	0.14159144	-2.820194	7.41E-14	6.82E-13	-1	0.20188563	-2.3083899	5.77E-08	4.45E-07	-1	1.42583215	0.51180416	0.34732213	0.84560166	0
wbbI	0.08082612	-3.6290346	6.40E-24	1.28E-22	-1	0.1860611	-2.4261516	4.61E-10	5.14E-09	-1	2.3019922	1.20288295	0.00827227	0.08143014	0
wbbH	0.23782158	-2.0720485	5.06E-09	2.81E-08	-1	0.41374296	-1.2731933	0.00056764	0.00165353	-1	1.73972002	0.79885515	0.14727451	0.58035154	0
glf	0.22304666	-2.1645826	8.70E-11	5.94E-10	-1	0.30333668	-1.7210081	1.18E-06	6.88E-06	-1	1.35996962	0.44357443	0.64146268	0.93164618	0
wzxB	0.2623407	-1.9304865	0.00023604	0.00060722	-1	0.55108427	-0.8596552	0.04801326	0.08297637	0	2.10064343	1.0708313	0.2782113	0.7803712	0
mltD	0.12895083	-2.955107	2.04E-16	2.34E-15	-1	0.4027411	-1.3120754	0.00030076	0.00094662	-1	3.12321451	1.64303166	0.00014256	0.00357207	1
rfbC	0.13168014	-2.9248903	3.15E-10	2.06E-09	-1	0.2050279	-2.2861079	3.23E-05	0.00013232	-1	1.55701458	0.63878246	0.22218659	0.70531966	0
rfbA	0.26425539	-1.9199952	4.65E-08	2.25E-07	-1	0.24142408	-2.0503585	4.22E-06	2.14E-05	-1	0.91360137	-0.1303633	0.83097391	0.97888613	0
rfbD	0.33539999	-1.5760454	1.02E-05	3.48E-05	-1	0.37547454	-1.413213	0.00349518	0.00829495	-1	1.11948287	0.16283245	0.46681103	0.89320173	0
rfbB	0.19243375	-2.3775662	5.03E-10	3.19E-09	-1	0.32759296	-1.6100237	0.00011752	0.00041445	-1	1.70236745	0.76754248	0.12864348	0.53723271	0
wcaN	0.51900214	-0.9461876	0.01366522	0.02374998	0	0.87085967	-0.1994878	0.41380605	0.50458303	0	1.67795005	0.74669977	0.18814847	0.66219851	0
wcaM	3.91368661	1.96852824	1.60E-06	6.29E-06	1	4.01165041	2.00419589	1.33E-05	6.04E-05	1	1.02503108	0.03566765	0.77797675	0.9635071	0
wcaL	5.95410445	2.57388453	6.68E-08	3.16E-07	1	6.04955176	2.59682825	4.63E-07	2.93E-06	1	1.01603051	0.02294372	0.72011403	0.95399541	0
wcaK	5.16204885	2.36794379	2.30E-08	1.16E-07	1	5.69148661	2.50880553	1.49E-07	1.06E-06	1	1.10256349	0.14086174	0.62427675	0.92989404	0
wzxC	6.8292887	2.77173532	1.91E-07	8.47E-07	1	8.17568602	3.03133979	2.54E-07	1.71E-06	1	1.19715045	0.25960447	0.49269537	0.90539057	0
wcaJ	8.51747293	3.09042546	1.75E-06	6.84E-06	1	8.86205362	3.14764106	6.48E-06	3.17E-05	1	1.04045574	0.0572156	0.74438915	0.95683847	0
gloB	0.19239073	-2.3778888	2.90E-10	1.90E-09	-1	0.17432801	-2.5201237	3.16E-07	2.08E-06	-1	0.90611441	-0.1422349	0.9322106	1	0
cpsG	5.75552412	2.52494731	4.60E-06	1.68E-05	1	4.5758249	2.19403185	5.75E-05	0.00022002	1	0.79503183	-0.3309155	0.94753153	1	0
cpsB	7.67321882	2.9398319	1.36E-09	8.12E-09	1	10.4094471	3.37982154	6.27E-10	6.82E-09	1	1.35659458	0.43998964	0.31130857	0.81168136	0
wcaI	3.26991091	1.70925133	0.00087442	0.00198577	1	5.1809817	2.37322549	0.00012024	0.00042303	1	1.58444124	0.66397416	0.25598415	0.75662874	0
wcaH	4.6649987	2.22187668	0.00166716	0.00356077	1	6.17711161	2.6269324	0.00046332	0.00137642	1	1.32414005	0.40505572	0.42507159	0.87719499	0
wcaG	6.01412593	2.58835508	0.00035085	0.00086335	1	8.88350987	3.1511298	0.00035623	0.00109393	1	1.47710739	0.56277472	0.51415213	0.90937129	0
gmd	4.58137484	2.19578061	0.00010681	0.0002967	1	4.1746248	2.06164654	0.00109114	0.00299302	1	0.9112166	-0.1341341	0.9948224	1	0
wcaF	3.77005707	1.91458636	0.0149218	0.02561585	1	5.5082402	2.46159147	0.01248115	0.02544013	1	1.46104955	0.54700511	0.58927814	0.92806272	0
wcaE	9.21102579	3.20336183	2.75E-11	2.00E-10	1	9.31787515	3.220001	3.14E-09	3.03E-08	1	1.01160016	0.01663917	0.87831399	0.98956956	0
wcaD	7.72114766	2.9488153	8.25E-10	5.11E-09	1	7.50050837	2.90698838	3.81E-07	2.45E-06	1	0.97142403	-0.0418269	0.86926633	0.98621446	0
wcaC	5.75267252	2.52423235	2.56E-07	1.11E-06	1	7.33965907	2.87571305	2.20E-07	1.51E-06	1	1.27586944	0.35148071	0.42527878	0.87721207	0
yafS	0.38002917	-1.395818	0.00018295	0.00048272	-1	0.46944895	-1.0909598	0.02442959	0.04595759	-1	1.23529716	0.30485814	0.39263372	0.86854717	0
wcaB	8.58816361	3.10234968	0.00395457	0.00772548	1	5.95793117	2.57481146	0.01313034	0.02661605	1	0.6937375	-0.5275382	0.87758752	0.98956956	0
wcaA	4.97334872	2.31421759	0.02069286	0.03429715	1	4.83301429	2.27292326	0.00889745	0.01897579	1	0.97178271	-0.0412943	0.63725449	0.93163024	0
wzc	7.03537906	2.81462816	0.00019132	0.00050182	1	7.1333789	2.83458561	0.00036824	0.00112847	1	1.01392957	0.01995745	0.76821454	0.96147692	0
wzb	11.9931084	3.58413372	2.46E-06	9.40E-06	1	10.0506838	3.32922175	5.04E-05	0.00019589	1	0.83803827	-0.254912	0.93195082	1	0
wza	9.60160732	3.26327593	0.00114896	0.00254383	1	7.91740651	2.98502793	0.0048361	0.0110329	1	0.82459178	-0.278248	0.96997009	1	0
yegH	1.73819327	0.79758851	0.02300424	0.03780124	0	1.23409411	0.30345242	0.3655658	0.45711242	0	0.7099867	-0.4941361	0.28454745	0.78899581	0

asmA	0.31772668	-1.6541418	3.38E-07	1.44E-06	-1	0.40430959	-1.3064677	0.00200147	0.00508598	-1	1.27250752	0.34767418	0.22295527	0.70531966	0
dcd	0.17112203	-2.5469026	3.94E-13	3.40E-12	-1	0.23653151	-2.0798957	2.40E-07	1.62E-06	-1	1.38223877	0.46700685	0.28314815	0.78651746	0
udk	0.30076994	-1.7332677	8.87E-06	3.07E-05	-1	0.55422151	-0.8514654	0.06644825	0.11051367	0	1.84267587	0.88180232	0.05723887	0.32074131	0
yegE	1.89919214	0.92538587	0.00556064	0.01050978	0	1.73540018	0.79526838	0.05368431	0.09141849	0	0.91375704	-0.1301175	0.67327648	0.93997784	0
rnhA	0.50942204	-0.9730667	0.04239819	0.06570975	0	0.53874793	-0.8923177	0.26169139	0.34863258	0	1.05756697	0.08074902	0.55619373	0.92183747	0
alkA	1.9878133	0.99118226	0.01299396	0.02267654	0	1.69291467	0.75950926	0.05431728	0.092283	0	0.85164672	-0.231673	0.84121375	0.98067952	0
yegD	1.2403661	0.310766	0.38450672	0.45596289	0	2.14606923	1.10169662	0.01786203	0.03489493	1	1.73019017	0.79093062	0.08542953	0.417214	0
yegI	2.23960783	1.16324613	0.11002873	0.15215933	0	1.97283266	0.98026859	0.26401515	0.35088209	0	0.88088308	-0.1829775	0.83533824	0.98014902	0
yegJ	3.28669997	1.71663976	0.01554162	0.02657659	1	3.85477587	1.94664698	0.009576	0.02027617	1	1.17284082	0.23000722	0.60356296	0.92806272	0
yegK	5.83725473	2.54529003	0.00031124	0.00077495	1	6.55619162	2.71285802	0.00069759	0.0019901	1	1.12316353	0.16756799	0.72854234	0.95488877	0
yegL	7.54648299	2.91580444	0.00011443	0.00031453	1	5.19965282	2.3784153	0.00317826	0.00760407	1	0.6890167	-0.5373891	0.67034332	0.93979017	0
sibA	1.6757188	0.74478007	0.35645064	0.42702536	0	1.38491853	0.46980111	0.48383016	0.5724352	0	0.82646237	-0.274979	0.92157119	1	0
sibB	2.29727916	1.19992618	0.73493391	0.78353924	0	4.02361257	2.0084914	0.29094011	0.38048664	0	1.75146871	0.80856521	0.50814107	0.90925684	0
dnaQ	0.44143382	-1.1797309	0.00162532	0.00347653	-1	0.3971291	-1.33232	0.00370648	0.00872612	-1	0.89963452	-0.1525891	0.68431068	0.943596	0
mdtA	2.35349471	1.23480461	0.00405271	0.00789277	1	3.22507149	1.68933114	0.00052312	0.00153395	1	1.37033301	0.45452653	0.28558501	0.78928465	0
mdtB	3.5697223	1.83581185	1.47E-05	4.86E-05	1	3.47310856	1.79622751	0.00014879	0.00051248	1	0.97293522	-0.0395843	1	1	0
mdtC	3.99291415	1.99744205	1.65E-08	8.53E-08	1	4.28712175	2.10000939	8.99E-08	6.70E-07	1	1.07368243	0.10256734	0.60412972	0.92806272	0
iceT	5.0049335	2.3233509	3.45E-09	1.97E-08	1	5.83103293	2.54375147	6.40E-09	5.91E-08	1	1.16505702	0.22040057	0.44363711	0.88326847	0
baeS	2.70962031	1.43809071	0.00181584	0.00384863	1	3.31988815	1.73113464	0.00058595	0.00170239	1	1.22522264	0.29304393	0.43813575	0.88087171	0
baeR	0.76971353	-0.3776065	0.20591336	0.26516599	0	0.89677196	-0.1571869	0.91235888	0.93413204	0	1.16507237	0.22041957	0.39341622	0.86854717	0
yegP	3.29559771	1.72054014	0.05667004	0.08482262	0	0.35260465	-1.5038766	0.14529394	0.21401797	0	0.10699262	-3.2244167	0.00294233	0.03750197	-1
yegQ	1.18911835	0.24989231	0.49595992	0.56547625	0	1.6821221	0.75028243	0.05175836	0.088514	0	1.41459603	0.50039012	0.16332764	0.6114388	0
cyaR	0.23102428	-2.1138836	0.05633922	0.08444191	0	0.21903095	-2.1907934	0.11513531	0.17520074	0	0.94808627	-0.0769097	1	1	0
ogrK	0.1947915	-2.3599974	6.49E-05	0.00018998	-1	0.32077866	-1.6403499	0.01316462	0.02663666	-1	1.6467796	0.71964748	0.40081436	0.86854717	0
aspV	3.92889756	1.97412455	0.02882432	0.04629711	1	3.26529839	1.70721483	0.19694706	0.2760257	0	0.83109787	-0.2669097	0.59856304	0.92806272	0
yegZ	4.46628376	2.15907491	0.01587431	0.02708259	1	3.30215616	1.72340835	0.05523466	0.09357283	0	0.73935207	-0.4356666	0.8251865	0.97661485	0
yegR	5.51806955	2.46416364	0.0002681	0.00067878	1	4.06960406	2.02488844	0.00986038	0.02081846	1	0.73750503	-0.4392752	0.65887625	0.93664426	0
yegS	2.71858403	1.44285542	0.00021635	0.00056034	1	1.5647758	0.64595596	0.09711994	0.15278498	0	0.57558486	-0.7968995	0.13963326	0.56235861	0
gatR	1.50659975	0.5912962	0.16379963	0.2170726	0	1.20757965	0.27211835	0.76121941	0.81705819	0	0.80152652	-0.3191778	0.35438502	0.84775072	0
gatD	1.09485131	0.13073495	1	1	0	0.65098237	-0.6193096	0.18791658	0.26606965	0	0.59458519	-0.7500446	0.14532858	0.57686682	0
gatC	0.72869313	-0.4566167	0.06185385	0.09143523	0	0.36689509	-1.4465605	1.45E-05	6.51E-05	-1	0.50349739	-0.9899438	0.00197701	0.02830719	0
gatB	0.41010277	-1.2859426	3.87E-06	1.43E-05	-1	0.20633539	-2.2769368	1.88E-10	2.22E-09	-1	0.50313092	-0.9909942	0.00188695	0.02737318	0
gatA	0.44440447	-1.1700548	1.95E-05	6.28E-05	-1	0.22412711	-2.157611	1.02E-09	1.07E-08	-1	0.50433135	-0.9875562	0.00203301	0.0288282	0
yafT	4.73404043	2.24307203	0.00012253	0.0003347	1	3.75437596	1.90857313	0.0041509	0.00965411	1	0.79305955	-0.3344989	0.62752299	0.93095788	0
gatZ	0.4621744	-1.1134907	6.94E-05	0.00020161	-1	0.26030086	-1.941748	8.23E-08	6.18E-07	-1	0.56320915	-0.8282573	0.01515579	0.12645175	0
gatY	0.38725814	-1.3686325	2.34E-06	8.99E-06	-1	0.22674185	-2.1408774	6.49E-09	5.98E-08	-1	0.58550571	-0.7722449	0.02414185	0.17953722	0
fbaB	5.56487616	2.47634958	0.00923559	0.01664366	1	0.91103479	-0.1344219	0.9004697	0.92367121	0	0.1637116	-2.6107715	0.02316972	0.17358733	0
yegT	2.69900881	1.43242969	0.00121992	0.00268933	1	3.36672356	1.75134527	0.00017849	0.00059753	1	1.24739258	0.31891558	0.32318724	0.82325896	0
yegU	0.61757241	-0.6953198	0.23632417	0.29909566	0	0.89097082	-0.1665499	1	1	0	1.44269855	0.52876988	0.24715948	0.74758115	0
yegV	0.99923264	-0.0011075	0.99231845	0.99773972	0	1.27146523	0.34649201	0.21774608	0.30022463	0	1.27244165	0.3475995	0.2292533	0.71702627	0
yegW	1.41579927	0.50161674	0.15139256	0.20239132	0	0.98563784	-0.0208705	0.91184566	0.93382293	0	0.69617061	-0.5224872	0.20527911	0.68468503	0
yegX	3.32884523	1.7350218	0.00021501	0.00055767	1	2.86829941	1.52019563	0.00150213	0.00395585	1	0.86164998	-0.2148262	0.99907753	1	0
thiD	1.59761877	0.67592319	0.42608298	0.49828131	0	1.79769712	0.84614998	0.10466916	0.16252039	0	1.12523535	0.17022679	0.41820012	0.87364403	0
thiM	1.73816529	0.79756528	0.0552435	0.08291218	0	2.99042181	1.580349	0.00588168	0.01316673	1	1.72044732	0.78278372	0.19612591	0.67373807	0
ykfM	5.19894923	2.37822007	0.01213719	0.02132457	1	3.47760325	1.79809335	0.14049011	0.20784258	0	0.66890502	-0.5801267	0.53053616	0.91717489	0
rcnR	2.1866896	1.12874845	0.02408721	0.03934659	1	1.52984576	0.6133862	0.27299292	0.36096819	0	0.69961724	-0.5153622	0.41559664	0.87131399	0
rcnA	4.01721092	2.00619421	2.72E-05	8.56E-05	1	3.89632752	1.96211495	0.00010795	0.00038564	1	0.96990862	-0.0440793	0.83488225	0.98014902	0
rcnB	0.41630769	-1.2642779	0.00797648	0.01456475	-1	0.51349927	-0.9615659	0.02370492	0.04478497	0	1.23346092	0.30271201	0.97814296	1	0
yehA	7.92166063	2.9858029	8.23E-05	0.00023522	1	5.22534516	2.38552634	0.00285435	0.00692281	1	0.65962749	-0.6002766	0.5613151	0.92432422	0
yehB	4.25076388	2.08772212	0.00045352	0.00108988	1	3.47095738	1.79533365	0.00271208	0.00661407	1	0.81654909	-0.2923885	0.93606406	1	0
yehC	1.57396985	0.6544079	0.32214757	0.39166689	0	1.26968063	0.34446566	0.73613735	0.79749717	0	0.80667405	-0.3099422	0.63562157	0.93163024	0
yehD	0.08514296	-3.553969	3.67E-14	3.45E-13	-1	0.04426783	-4.4975977	7.50E-13	1.24E-11	-1	0.51992353	-0.9436286	0.17407492	0.63130789	0
yehE	0.82659564	-0.2747463	0.43581222	0.5069746	0	0.49013555	-1.0287473	0.04664522	0.08108782	0	0.59295685	-0.754001	0.13212503	0.54303016	0
mrp	0.4387972	-1.1883738	0.00013152	0.00035684	-1	0.36955515	-1.4361384	0.0002454	0.00079387	-1	0.84220034	-0.2477646	0.5630917	0.92432422	0
metG	0.5452415	-0.8750327	0.01781554	0.03002336	0	0.82500912	-0.277518	0.5886136	0.67003696	0	1.51310773	0.59751471	0.1101502	0.49166233	0
yafU	4.74319706	2.24585981	0.00699576	0.01294523	1	8.11102653	3.01988451	0.0034938	0.00829495	1	1.71003364	0.77402471	0.48113519	0.89935549	0
yehH	6.41728141	2.68196225	0.00034549	0.00085302	1	5.37040315	2.4250304	0.00087705	0.00245141	1	0.83686577	-0.2569319	0.92851683	1	0
yehI	5.16971799	2.37008558	4.44E-07	1.87E-06	1	4.58672128	2.19746324	9.90E-06	4.65E-05	1	0.88722853	-0.1726223	0.98171855	1	0
yehK	6.48417488	2.696923	0.04176392	0.06479495	0	7.09484789	2.82677175	0.05423346	0.09217942	0	1.09417899	0.12984875	0.85543308	0.98266248	0
yehL	7.26877345	2.86171194	2.12E-08	1.07E-07	1	8.8902998	3.15223207	2.92E-08	2.36E-07	1	1.22308115	0.29052013	0.46358191	0.89060394	0
yehM	4.57890231	2.19500178	2.90E-05	9.07E-05	1	7.67215192	2.93963129	2.68E-06	1.44E-05	1	1.67554392	0.7446295	0.20222344	0.6797297	0
yehP	4.00533847	2.00192416	8.13E-05	0.00023302	1	5.95022864	2.57294511	1.28E-05	5.85E-05	1	1.48557448	0.57102094	0.26781174	0.76966758	0
yehQ	2.9688332	1.56989604	0.0077396	0.01418498	1	4.38984813	2.13417103	0.00075372	0.00213779	1	1.47864425	0.56427499	0.25863238	0.75892335	0
yehR	2.22417536	1.15327054	0.17437994	0.2293735	0	2.12813774	1.08959153	0.13316956	0.19874241	0	0.95682102	-0.063679	0.82016737	0.97465322	0
yehS	0.39366584	-1.3449566	0.00133844	0.00291945	-1	0.71522711	-0.4835267	0.13512839	0.20105466	0	1.81683816	0.86142991	0.22215114	0.70531966	0
yehT	0.32637539	-1.6153958	4.85E-05	0.000146	-1	0.39134887	-1.3534728	0.00438613	0.01011207	-1	1.19907592	0.26192301	0.59905369	0.92806272	0
yafF	2.40972726	1.26886986	0.31601454	0.38474386	0	2.84500119	1.50842956	0.24522106	0.33087995	0	1.18063228	0.2395597	0.78093476	0.9635071	0
yehU	0.63429182	-0.6567814	0.06510423	0.09553668	0	0.79707908	-0.3272052	0.42149477	0.51212137	0	1.25664412	0.32957613	0.4474263	0.88755478	0
mlrA	4.06086797	2.02178812	3.95E-07	1.68E-06	1	2.32209734	1.21542845	0.01635682	0.03221069	1	0.57182291	-0.8063597	0.07097453	0.36997363	0
yohO	4.42289273	2.14499025	0.02809665	0.04522701	1	3.8070488	1.92867306	0.23768472	0.32218674	0	0.86075992	-0.2163172	0.60436145	0.92806272	0
yehW	5.56608489	2.47666291	1.68E-07	7.53E-07	1	4.48555933	2.16528789	1.65E-05	7.26E-05	1	0.80587332	-0.311375	0.75608669	0.9573569	0
yehX	4.38835945	2.1336817	1.08E-05	3.67E-05	1	4.76447271	2.25231656	2.76E-05	0.00011512	1	1.08570703	0.11863486	0.66098644	0.93664426	0
yehY	3.76576649	1.91294354	3.19E-06	1.20E-05	1	3.06542167	1.61608554	0.00020579	0.00067714	1	0.8140233	-0.296858	0.69939401	0.94869712	0
osmF	1.55848727	0.64014637	0.05908028	0.08786451	0	0.61822086	-0.6938058	0.35218049	0.44300757	0	0.39668008	-1.3339521	0.01516848	0.12645175	0
bglX	0.67901016	-0.5584949	0.11270805	0.15547515	0	1.09620902	0.13252291	0.87021219	0.90205669	0	1.61442212	0.69101785	0.20123683	0.67848197	0
dld	0.20575525	-2.2809988	1.23E-12	1.01E-11	-1	0.19645015	-2.3477648	8.66E-10	9.27E-09	-1	0.95477587	-0.066766	0.72222302	0.95399541	0
pbpG	0.47739109	-1.0667565	0.00433264	0.00837518	-1	0.57439701	-0.7998798	0.08129516	0.13129087	0	1.20320011	0.26687661	0.50977321	0.90925684	0
yafV	0.59544422	-0.7479617	0.05683254	0.08500823	0	0.44317517	-1.174051	0.00856322	0.01828945	-1	0.74427655	-0.4260893	0.28535258	0.78928465	0
yohC	5.40767447	2.43500831	0.00093393	0.00210898	1	1.50923852	0.59382083	0.31137583	0.40163708	0	0.27909197	-1.8411875	0.05547286	0.31647516	0
yohD	1.64410001	0.71729806	0.0380715	0.05970771	0	1.71681749	0.77973668	0.15995303	0.23235781	0	1.04422935	0.06243861	0.84250457	0.98067952	0
yohF	3.69467178	1.8854462	7.91E-07	3.24E-06	1	1.68649606	0.75402894	0.10636266	0.16445647	0	0.45646709	-1.1314173	0.0127939	0.11152756	0
mdtQ	4.08240635	2.02941979	9.03E-05	0.00025418	1	4.56028641	2.18912444	0.00012799	0.00044746	1	1.11705843	0.15970465	0.62625517	0.92989404	0
yohP	9.63989774	3.26901784	1.54E-10	1.03E-09	1	1.27186083	0.34694082	0.73723775	0.79810231	0	0.13193717	-2.922077	1.33E-07	1.08E-05	-1
dusC	2.15777757	1.10954616	0.01714267	0.02903342	1	4.10277401	2.03659969	0.00012069	0.00042363	1	1.90138875	0.92705353	0.05645641	0.31960562	0
yohJ	0.94433618	-0.0826276	0.64326571	0.70000115	0	0.71112184	-0.4918313	0.41854744	0.50910023	0	0.75303886	-0.4092038	0.57211756	0.92806272	0
yohK	1.2233911	0.29088569	0.50212628	0.5719164	0	0.63951241	-0.6449557	0.22885489	0.31339007	0	0.5227375	-0.9358414	0.07139579	0.37173016	0
cdd	0.01509216	-6.0500573	1.14E-57	1.80E-55	-1	0.01629492	-5.9394343	7.75E-39	4.28E-36	-1	1.07969437	0.11062298	0.79053419	0.96513738	0
sanA	0.44711146	-1.1612936	0.0147934	0.02543498	-1	0.63872775	-0.646727	0.23807903	0.32262228	0	1.42856494	0.51456662	0.39190264	0.86854717	0
rpsT	0.15608443	-2.6796014	0.00022094	0.00057102	-1	0.72786	-0.4582671	0.2968491	0.38649735	0	4.66324531	2.22133432	0.02643627	0.19243551	0
ivy	0.83266586	-0.2641904	0.47880124	0.54895419	0	0.57265772	-0.804255	0.12113289	0.18306642	0	0.68774012	-0.5400646	0.28001432	0.78304576	0
yeiS	0.52973398	-0.91666	0.63149853	0.69008635	0	1.15972362	0.21378103	0.98583228	0.99688623	0	2.18925661	1.13044107	0.5536508	0.92069223	0
preT	0.07192303	-3.7974025	6.01E-27	1.53E-25	-1	0.07890795	-3.6636855	5.25E-17	1.67E-15	-1	1.09711669	0.13371698	0.52387279	0.91279296	0
preA	0.21045573	-2.2484113	4.05E-10	2.60E-09	-1	0.15153049	-2.72232	7.08E-09	6.48E-08	-1	0.72001121	-0.4739087	0.49706155	0.90539057	0
mglC	1.25086777	0.32292928	0.27737727	0.34405817	0	1.20505569	0.26909982	0.27854918	0.36699726	0	0.96337576	-0.0538295	0.82382245	0.97661485	0
mglA	0.84676596	-0.2399648	0.59138452	0.65418117	0	0.79015187	-0.3397981	0.58989753	0.67115273	0	0.9331408	-0.0998333	0.92837423	1	0
mglB	0.31256673	-1.6777639	3.26E-07	1.39E-06	-1	0.17835856	-2.4871476	1.90E-09	1.91E-08	-1	0.57062554	-0.8093838	0.03466605	0.23131824	0
galS	1.13934077	0.18819931	0.74450497	0.79221355	0	1.31742935	0.3977256	0.22809727	0.3125463	0	1.15630845	0.20952629	0.3903009	0.86854717	0
yeiB	2.88713784	1.52963999	6.82E-05	0.00019877	1	3.62152049	1.85659554	2.84E-05	0.00011791	1	1.25436356	0.32695555	0.39401084	0.86854717	0
folE	0.57818729	-0.7903912	0.01001325	0.01787734	0	0.62233672	-0.6842327	0.05670755	0.09571836	0	1.07635835	0.10615847	0.89019071	0.99411016	0
yeiG	0.71258581	-0.4888643	0.24088634	0.30434639	0	0.44983381	-1.152536	0.01152395	0.02378892	-1	0.63126968	-0.6636716	0.12839457	0.53670148	0
fadE	32.9608782	5.04268277	9.41E-16	1.03E-14	1	6.54166683	2.70965829	1.39E-05	6.28E-05	1	0.19846761	-2.3330245	0.00118352	0.01911846	-1
cirA	1.38069696	0.46539671	0.11601968	0.15949546	0	1.74021953	0.79926932	0.02384571	0.04501247	0	1.26039209	0.33387261	0.30928631	0.81096368	0

lysP	1.01896658	0.02710673	0.89247649	0.91695444	0	1.20920874	0.27406331	0.48448506	0.57275	0	1.18670108	0.24695658	0.53617982	0.9187668	0
yeiE	0.233122	-2.1008429	9.73E-09	5.19E-08	-1	0.19247543	-2.3772538	7.80E-08	5.89E-07	-1	0.82564249	-0.2764109	0.5345187	0.9187668	0
yeiH	3.25122628	1.70098397	7.13E-06	2.51E-05	1	2.29786321	1.20029292	0.00159619	0.00416877	1	0.70676816	-0.5006911	0.41894714	0.87375001	0
nfo	0.50134339	-0.996129	0.00522119	0.00991488	0	0.53310645	-0.9075045	0.01878798	0.03654229	0	1.06335589	0.08862453	0.94826513	1	0
yeiI	1.13068887	0.177202	0.52569824	0.59310578	0	0.77530324	-0.3671674	0.6568349	0.73075363	0	0.68569105	-0.5443694	0.32997995	0.8313479	0
nupX	4.86716694	2.28308226	0.0002973	0.00074317	1	4.4931688	2.16773326	0.00123136	0.00331994	1	0.92315897	-0.115349	1	1	0
rihB	5.10770738	2.35267588	4.96E-07	2.08E-06	1	5.16539748	2.36887937	4.72E-06	2.37E-05	1	1.01129471	0.01620349	0.78372859	0.9635071	0
yeiL	1.63611333	0.71027269	0.1769308	0.23209031	0	1.21569977	0.28178698	0.41687371	0.50748345	0	0.74304129	-0.4284857	0.76009907	0.95840749	0
psuT	2.8172224	1.49427346	0.00103247	0.00230791	1	2.78866653	1.47957543	0.00379717	0.00892537	1	0.98986382	-0.014698	0.87809002	0.98956956	0
gmhA	0.14618218	-2.7741607	3.19E-15	3.33E-14	-1	0.16964961	-2.55937	1.61E-10	1.92E-09	-1	1.16053553	0.21479069	0.84043433	0.98067952	0
psuG	5.0225032	2.32840658	4.24E-08	2.08E-07	1	3.90838792	1.96657366	1.84E-05	7.99E-05	1	0.7781753	-0.3618329	0.64403838	0.93208609	0
psuK	3.88523703	1.95800262	0.00186107	0.00393506	1	4.42868016	2.14687681	0.00119259	0.00323516	1	1.13987387	0.18887419	0.59281122	0.92806272	0
fruA	18.6076123	4.21782103	2.16E-15	2.30E-14	1	5.81189653	2.53900902	2.68E-05	0.00011183	1	0.31233973	-1.678812	0.00263532	0.03451832	-1
fruK	21.5476512	4.42945871	8.92E-22	1.51E-20	1	5.01981912	2.32763538	3.88E-06	1.98E-05	1	0.23296363	-2.1018233	2.87E-05	0.00095215	-1
fruB	20.2887907	4.34261097	1.87E-12	1.52E-11	1	5.25835229	2.3946108	0.00037786	0.00115315	1	0.25917524	-1.9480002	0.00385209	0.04536137	-1
setB	3.33181474	1.73630818	0.00217909	0.00453156	1	3.15721956	1.65865459	0.00700731	0.0153903	1	0.94759757	-0.0776536	1	1	0
yeiW	1.76209794	0.81729411	0.10123552	0.14132657	0	2.31156897	1.20887241	0.10742865	0.1658146	0	1.31182775	0.3915783	0.72903195	0.95488877	0
yeiP	1.66601784	0.73640385	0.02809178	0.04522701	0	0.92497084	-0.1125202	0.43473312	0.52531738	0	0.55519864	-0.8489241	0.01011812	0.09400856	0
yeiQ	1.8233798	0.86661509	0.01396851	0.02417668	0	1.72851489	0.78953303	0.04123712	0.07308195	0	0.94797304	-0.0770821	0.98537626	1	0
yeiR	1.96296009	0.97303084	0.01461518	0.02516773	0	2.29841712	1.20064064	0.00833453	0.01785277	1	1.17089345	0.2276098	0.53587715	0.9187668	0
yafJ	0.44558876	-1.1662152	0.00179179	0.0037995	-1	0.57124254	-0.8078247	0.0647637	0.10807809	0	1.28199494	0.35839057	0.40055771	0.86854717	0
lpxT	2.823618	1.49754492	0.0484439	0.0739125	0	2.06819165	1.04836988	0.20946405	0.29043666	0	0.73246156	-0.449175	0.62109177	0.92938245	0
mepS	0.20601408	-2.2791852	2.27E-05	7.23E-05	-1	0.70701983	-0.5001774	0.18673471	0.26473554	0	3.43190059	1.77900776	0.01665758	0.13578543	0
rtn	1.4011259	0.4865866	0.17486146	0.22980157	0	1.75201119	0.80901199	0.07429773	0.12178103	0	1.25043095	0.32242539	0.4870746	0.90208042	0
yejA	1.25478521	0.32744043	0.41399475	0.48711104	0	1.20173494	0.26511872	0.44377412	0.53372473	0	0.95772163	-0.0623217	0.98693775	1	0
yejB	1.26437076	0.33841958	0.33944438	0.40875841	0	1.09384424	0.12940732	0.65551615	0.72983771	0	0.86512934	-0.2090123	0.74345116	0.95683847	0
yejE	3.68463692	1.88152246	2.47E-07	1.07E-06	1	2.67488727	1.41947809	0.0002357	0.00076529	1	0.72595681	-0.4620444	0.44302966	0.88245745	0
yejF	0.93869633	-0.0912696	0.78846396	0.82939874	0	0.8449151	-0.2431217	0.50726585	0.59481719	0	0.90009418	-0.1518521	0.64534082	0.93208609	0
yejG	0.59023824	-0.7606307	0.02238444	0.03686506	0	0.52428109	-0.9315876	0.00479134	0.01095908	0	0.88825334	-0.1709569	0.29540826	0.79986628	0
bcr	1.1153223	0.15746068	0.92607288	0.94424375	0	1.35335113	0.4365362	0.36363225	0.45556625	0	1.21341708	0.27907552	0.45635015	0.88755478	0
rsuA	0.56067259	-0.8347696	0.01667336	0.02831459	0	0.66867403	-0.580625	0.20962414	0.29056746	0	1.19262837	0.25414456	0.46644995	0.89320173	0
yafK	0.8379121	-0.2551292	0.3943543	0.46636223	0	1.60937562	0.68650108	0.36485087	0.45660606	0	1.92069743	0.94163027	0.09862495	0.45734597	0
yejH	2.30983103	1.20778732	0.00030431	0.00075855	1	2.19494256	1.13418319	0.00364926	0.00860977	1	0.95026109	-0.0736041	0.93367766	1	0
rplY	0.07729628	-3.6934571	2.12E-21	3.49E-20	-1	0.2019148	-2.3081814	1.80E-09	1.82E-08	-1	2.61221874	1.38527571	0.02491384	0.18403694	0
yejK	0.3709762	-1.4306015	0.0001599	0.00042702	-1	0.54075493	-0.8869532	0.02868651	0.05304003	0	1.45765398	0.54364829	0.26945243	0.76991797	0
yejL	0.37444491	-1.4171746	0.00410439	0.00797936	-1	0.71364122	-0.4867291	0.23860893	0.32324123	0	1.90586439	0.93044547	0.24669306	0.74719533	0
yejM	1.15154795	0.20357449	0.82175798	0.85715491	0	1.24518008	0.3163544	0.73691876	0.79795247	0	1.08130979	0.11277991	0.95713611	1	0
proL	3.13064611	1.64646044	0.02775026	0.04476732	1	2.05110025	1.03639801	0.22118491	0.304491	0	0.65516835	-0.6100624	0.53153149	0.91754792	0
yejO	2.55201709	1.35163799	0.00015112	0.00040675	1	2.43860598	1.28605667	0.00040987	0.00123717	1	0.95556021	-0.0655813	0.82419626	0.97661485	0
narP	0.25844057	-1.9520955	1.06E-07	4.88E-07	-1	0.20982303	-2.2527551	7.29E-06	3.53E-05	-1	0.81188114	-0.3006596	0.77129281	0.96223954	0
ccmH	0.51465591	-0.9583199	0.0620714	0.09172611	0	0.7331942	-0.4477327	0.4219751	0.51256404	0	1.42462991	0.51058719	0.34939786	0.84754927	0
yafQ	0.86527725	-0.2087656	0.38956757	0.46131902	0	0.69751898	-0.5196956	0.28717763	0.37668089	0	0.80612195	-0.31093	0.59893782	0.92806272	0
dsbE	0.59906296	-0.7392205	0.35224931	0.42256524	0	0.7403613	-0.4336986	0.74972494	0.80726962	0	1.2358656	0.30552186	0.50844619	0.90925684	0
ccmF	1.55997035	0.64151861	0.0323862	0.05145677	0	1.91860289	0.94005613	0.00656364	0.01451687	0	1.22989702	0.29853752	0.32178825	0.82265867	0
ccmE	0.55494013	-0.849596	0.12198151	0.16675714	0	0.85262608	-0.2300149	0.66957808	0.74231449	0	1.53642894	0.61958104	0.28591549	0.78944262	0
ccmD	0.26348118	-1.9242282	0.00965721	0.01728359	-1	0.31928134	-1.6470999	0.11094992	0.17003106	0	1.21178045	0.27712834	0.48957767	0.90336299	0
ccmC	0.21749625	-2.2009376	3.95E-08	1.95E-07	-1	0.33916438	-1.5599434	0.00158486	0.00414164	-1	1.55940335	0.64099414	0.09696591	0.45510989	0
ccmB	0.43811972	-1.1906029	0.00259489	0.0052961	-1	0.52988198	-0.916257	0.15327765	0.22398609	0	1.20944561	0.27434589	0.30418625	0.80892599	0
ccmA	0.11736761	-3.0908937	1.63E-14	1.60E-13	-1	0.2491995	-2.0046269	3.04E-06	1.59E-05	-1	2.12323906	1.08626682	0.03464086	0.23131824	0
napC	0.05315899	-4.2335425	8.69E-28	2.35E-26	-1	0.04128875	-4.5981074	3.87E-20	1.97E-18	-1	0.77670304	-0.364565	0.77646704	0.9635071	0
napB	0.05809774	-4.1053741	2.14E-23	4.04E-22	-1	0.05586202	-4.1619885	4.63E-15	1.07E-13	-1	0.96151787	-0.0566144	0.59770208	0.92806272	0
napH	0.08269011	-3.5961414	3.52E-20	5.21E-19	-1	0.11409473	-3.1316959	5.10E-11	6.51E-10	-1	1.37978693	0.4644455	0.1557729	0.59528465	0
dinJ	0.30072397	-1.7334882	8.90E-06	3.08E-05	-1	0.50339094	-0.9902489	0.00681624	0.01500049	0	1.67393021	0.74323938	0.33725646	0.83714543	0
napG	0.04189168	-4.5771924	5.49E-26	1.30E-24	-1	0.04360151	-4.5194781	3.09E-20	1.59E-18	-1	1.04081543	0.05771426	0.74223079	0.95683847	0
napA	0.00813821	-6.9410734	1.62E-67	5.98E-65	-1	0.02062189	-5.59968	8.02E-35	3.22E-32	-1	2.53395948	1.34139346	0.00011453	0.00298872	1
napD	0.00753681	-7.0518306	6.81E-39	4.06E-37	-1	0.01186856	-6.3967114	1.04E-21	6.85E-20	-1	1.57474607	0.65511921	0.07402066	0.37957754	0
napF	0.03936922	-4.6667881	4.86E-30	1.56E-28	-1	0.09919497	-3.3335893	1.67E-15	4.20E-14	-1	2.51960719	1.33319883	0.03668131	0.2400068	0
eco	0.17847088	-2.4862394	1.45E-13	1.30E-12	-1	0.18083028	-2.4672918	2.21E-10	2.57E-09	-1	1.01322008	0.01894757	0.72435456	0.95459348	0
mqo	3.04203756	1.60503797	4.27E-06	1.57E-05	1	2.16246045	1.11267374	0.0039174	0.00917836	1	0.71085922	-0.4923642	0.30677905	0.81011621	0
yojI	3.12208145	1.64250817	1.23E-05	4.14E-05	1	3.23398455	1.69331279	9.61E-05	0.00034776	1	1.03584247	0.05080461	0.84050243	0.98067952	0
alkB	4.48532295	2.16521186	2.43E-07	1.06E-06	1	3.17073464	1.66481714	0.00016643	0.00056315	1	0.70691334	-0.5003947	0.462694	0.8898689	0
ada	1.41011754	0.49581542	0.31924044	0.38845317	0	0.8238756	-0.2795016	0.65531687	0.72979971	0	0.58426024	-0.775317	0.13954016	0.56235861	0
apbE	1.26105383	0.33462986	0.24370452	0.30746726	0	1.66544427	0.73590708	0.10799433	0.16657136	0	1.32067659	0.40127722	0.43166132	0.88005519	0
yafL	7.04141856	2.8158661	6.65E-10	4.15E-09	1	5.84017663	2.546012	7.14E-06	3.47E-05	1	0.82940342	-0.2698541	0.44466461	0.88411674	0
ompC	0.04686292	-4.4154095	1.70E-37	8.86E-36	-1	0.10568779	-3.2421193	3.57E-17	1.18E-15	-1	2.25525432	1.17329013	0.00227562	0.03143336	1
micF	11.561132	3.53121077	0.00392827	0.00768092	1	16.1056205	4.00949234	0.01197849	0.02456286	1	1.39308334	0.47828157	0.93195343	1	0
rcsD	0.50756412	-0.978338	0.00276959	0.00562878	0	0.4455192	-1.1664405	0.00179967	0.00462336	-1	0.87775944	-0.1881025	0.49843149	0.90539057	0
rcsB	0.1571984	-2.6693416	1.52E-16	1.78E-15	-1	0.14227333	-2.8132628	3.07E-12	4.62E-11	-1	0.90505586	-0.1439213	0.75081452	0.95683847	0
rcsC	0.65283448	-0.6152108	0.08741818	0.12379804	0	0.87430145	-0.1937973	0.46704079	0.55659473	0	1.33923909	0.42141354	0.43755445	0.88087171	0
atoS	0.31295381	-1.6759784	6.28E-06	2.24E-05	-1	0.32309998	-1.6299475	0.00012928	0.00045091	-1	1.03242065	0.0460309	0.99517411	1	0
atoC	0.2011035	-2.3139899	3.76E-10	2.43E-09	-1	0.22380101	-2.1597115	3.08E-06	1.61E-05	-1	1.11286484	0.15427838	0.47965591	0.89897261	0
atoD	6.36913601	2.67109768	0.00017094	0.00045429	1	6.22480305	2.63802819	0.00039106	0.00118769	1	0.97733869	-0.0330695	0.78697338	0.9635071	0
atoA	6.71899342	2.74824512	0.016253	0.02765389	1	7.41968447	2.89135784	0.01755815	0.03434681	1	1.10428512	0.14311272	0.73674729	0.95683847	0
atoE	6.75604661	2.75617928	0.00045901	0.00110067	1	5.28817598	2.40277019	0.00231587	0.00579821	1	0.78273231	-0.3534091	0.93489969	1	0
rayT	0.41079984	-1.2834925	0.00043001	0.00103564	-1	0.46769404	-1.096363	0.02602937	0.04858654	-1	1.13849615	0.18712942	0.5196419	0.91269012	0
atoB	8.31678631	3.05602616	5.41E-09	3.00E-08	1	8.7042967	3.12172773	1.59E-07	1.13E-06	1	1.04659377	0.06570157	0.81775768	0.97348376	0
yfaP	4.61812655	2.20730771	0.00963712	0.01726163	1	4.69910937	2.23238735	0.01161563	0.02395216	1	1.01753586	0.02507964	0.75606702	0.9573569	0
yfaQ	5.03183359	2.33108421	6.84E-07	2.83E-06	1	5.03919342	2.33319283	3.03E-06	1.59E-05	1	1.00146265	0.00210862	0.7220905	0.95399541	0
yfaS	3.52418489	1.81728961	9.49E-08	4.39E-07	1	3.74397421	1.9045705	3.40E-07	2.21E-06	1	1.062366	0.08728088	0.59277961	0.92806272	0
yfaT	5.11727348	2.35537534	4.26E-05	0.00012951	1	6.235017	2.64039349	0.00011297	0.0004	1	1.2184256	0.28501816	0.6394062	0.93163024	0
yfaA	2.48826197	1.31513838	0.01726362	0.02921586	1	1.49927476	0.5842648	0.16874508	0.24360813	0	0.60253895	-0.7308736	0.46289325	0.8898689	0
gyrA	0.20375855	-2.2950675	2.21E-12	1.79E-11	-1	0.41999808	-1.2515453	0.00055316	0.00161668	-1	2.06125379	1.04352215	0.00623385	0.06561167	0
ubiG	1.05005894	0.07047031	0.92506306	0.94369924	0	1.04750236	0.06695349	0.69249802	0.76179954	0	0.99756529	-0.0035168	0.72297067	0.95399541	0
yfaL	2.96592198	1.56848065	1.74E-05	5.65E-05	1	3.34556372	1.74224932	1.26E-05	5.77E-05	1	1.12800126	0.17376868	0.47005035	0.89543069	0
nrdA	0.2584095	-1.952269	1.82E-08	9.35E-08	-1	0.36032671	-1.4726225	0.00068821	0.00196588	-1	1.39440196	0.4796465	0.13961729	0.56235861	0
lfhA	3.51529479	1.81364568	0.00283272	0.00574742	1	4.86527586	2.28252161	0.00096946	0.00269437	1	1.38403069	0.46887593	0.40656206	0.86959974	0
nrdB	0.16558727	-2.5943363	2.39E-12	1.92E-11	-1	0.22275285	-2.1664842	3.35E-07	2.18E-06	-1	1.34522934	0.42785215	0.35036394	0.84775072	0
yfaE	0.30981575	-1.6905176	0.00402715	0.00786034	-1	0.37500132	-1.4150324	0.05229378	0.08929143	0	1.21040107	0.27548517	0.48674273	0.90190565	0
inaA	2.10150958	1.07142604	0.01891839	0.03160459	1	1.76450384	0.81926257	0.14566804	0.21428331	0	0.83963635	-0.2521635	0.54182458	0.91914725	0
yfaH	1.18293077	0.24236564	0.88324655	0.9089613	0	1.3056244	0.38473992	0.96887393	0.98063535	0	1.10372004	0.14237428	0.88864854	0.99411016	0
glpQ	0.12253104	-3.0287808	7.41E-21	1.15E-19	-1	0.16729558	-2.5795287	8.39E-13	1.37E-11	-1	1.36533224	0.44925206	0.51469788	0.90937129	0
glpT	0.04476829	-4.4813789	3.13E-38	1.77E-36	-1	0.15847013	-2.6577172	3.56E-12	5.26E-11	-1	3.53978489	1.82366169	1.85E-07	1.46E-05	1
glpA	0.33391812	-1.5824337	1.36E-07	6.15E-07	-1	0.25380603	-1.9782017	2.71E-09	2.67E-08	-1	0.76008463	-0.395768	0.05412643	0.3120921	0
glpB	0.06938009	-3.8493344	9.49E-30	2.97E-28	-1	0.14884561	-2.7481114	7.28E-13	1.21E-11	-1	2.14536474	1.10122295	0.00308245	0.03839997	1
glpC	0.07177709	-3.8003327	2.66E-29	8.15E-28	-1	0.18322556	-2.4483073	6.60E-11	8.14E-10	-1	2.55270245	1.35202538	0.00027022	0.00592866	1
yfaD	2.06887368	1.04884556	0.18290348	0.23887779	0	1.89426653	0.92163934	0.18867934	0.26690769	0	0.9156028	-0.1272062	0.91070697	0.99971546	0
yaaY	0.22470517	-2.1538948	0.00626819	0.01171175	-1	0.87469704	-0.1931447	0.47183501	0.5612487	0	3.89264309	1.96075007	0.09229731	0.44194476	0
lafU	2.06674645	1.04736141	0.06477098	0.09514247	0	2.70831092	1.43739337	0.03206465	0.05870576	0	1.31042244	0.39003196	0.49909398	0.90539057	0
ypaA	1.43550125	0.52155458	0.58421461	0.64803514	0	1.57807075	0.65816188	0.33190638	0.42146387	0	1.09931688	0.1366073	0.66610541	0.93829234	0
rhmA	3.43992612	1.78237758	0.00221935	0.00460229	1	4.80900042	2.26573705	0.00026357	0.00084318	1	1.39799526	0.48335947	0.30059732	0.80487807	0
rhmT	9.69589857	3.27737461	3.41E-14	3.22E-13	1	9.17330754	3.19744201	5.59E-11	7.04E-10	1	0.94610185	-0.0799326	0.96435163	1	0
rhmD	5.3725292	2.42560142	1.44E-09	8.52E-09	1	3.83695923	1.93996343	9.90E-06	4.65E-05	1	0.71418118	-0.485638	0.42877981	0.87784805	0

rhmR	3.45239226	1.78759639	0.00049763	0.00118557	1	2.72033419	1.4437839	0.00246469	0.00609753	1	0.78795629	-0.3438125	0.92904245	1	0
yfaY	0.41563158	-1.2666228	0.00025298	0.00064445	-1	0.47658059	-1.0692079	0.02905103	0.05360188	0	1.14664191	0.19741491	0.34661672	0.84500832	0
yfaZ	3.63478062	1.86186829	5.42E-05	0.00016168	1	2.80379553	1.48738114	0.00244681	0.00606418	1	0.77137957	-0.3744871	0.66746223	0.93861876	0
nudI	0.18120076	-2.4643391	1.36E-05	4.52E-05	-1	0.28509386	-1.8104911	0.00232444	0.00581637	-1	1.57335912	0.653848	0.43283904	0.88049119	0
ais	9.51862772	3.2507536	1.94E-07	8.60E-07	1	5.52579325	2.46618159	0.00019596	0.00064866	1	0.58052415	-0.784572	0.37778628	0.85988523	0
arnB	6.00328075	2.58575114	1.19E-09	7.16E-09	1	4.44067716	2.15077969	1.25E-06	7.20E-06	1	0.73970839	-0.4349714	0.59661577	0.92806272	0
dinB	1.59050609	0.6694859	0.05435096	0.08179495	0	1.61856097	0.69471172	0.0713146	0.11763316	0	1.01763897	0.02522582	0.83551973	0.98014902	0
arnC	5.293977	2.40435193	0.0093715	0.01684543	1	4.8484887	2.27753512	0.01167385	0.02406098	1	0.91584997	-0.1268168	0.85120264	0.98212032	0
arnA	2.6650325	1.41415313	6.35E-05	0.00018688	1	3.14385474	1.65253456	0.00026492	0.00084585	1	1.17966844	0.23838143	0.68402935	0.943596	0
arnD	1.623248	0.69888343	0.27147665	0.33777813	0	2.07879424	1.05574697	0.09702561	0.15269095	0	1.28063872	0.35686354	0.4876361	0.90208042	0
arnT	3.21672724	1.68559361	4.73E-06	1.72E-05	1	2.7481457	1.45845849	0.00021879	0.00071672	1	0.85432973	-0.2271351	0.82503514	0.97661485	0
arnE	2.605546	1.38158572	0.00443179	0.00853701	1	2.64207984	1.40167407	0.01386331	0.02784635	1	1.01402157	0.02008834	0.91051114	0.99971546	0
arnF	0.98868191	-0.0164217	0.86108325	0.89010173	0	1.13783489	0.18629123	0.63054474	0.70846103	0	1.15086043	0.20271289	0.56540848	0.92555731	0
pmrD	0.33796778	-1.5650424	0.00171639	0.00365539	-1	0.64147506	-0.6405349	0.07480558	0.1224318	0	1.89803611	0.92450744	0.38298072	0.86038969	0
menE	0.44304227	-1.1744837	0.00089358	0.00202824	-1	0.55151572	-0.8585261	0.0672691	0.11162679	0	1.2448377	0.31595766	0.31420113	0.81404427	0
menC	0.27581918	-1.8582053	4.33E-08	2.12E-07	-1	0.24301846	-2.0408622	9.94E-07	5.87E-06	-1	0.88107891	-0.1826569	0.70148406	0.94869712	0
menB	0.23119079	-2.1128442	5.92E-11	4.13E-10	-1	0.14792224	-2.7570891	2.51E-11	3.33E-10	-1	0.63982759	-0.6442449	0.11149419	0.49465734	0
yafN	1.57282754	0.65336049	0.37067278	0.4413625	0	0.67250098	-0.5723917	0.7179522	0.78336562	0	0.42757452	-1.2257522	0.22310692	0.70531966	0
menH	0.69001427	-0.5353019	0.23941025	0.30274122	0	0.76564341	-0.3852555	0.43464199	0.52531738	0	1.10960519	0.15004644	0.77530217	0.96312185	0
menD	0.7600497	-0.3958343	0.24636401	0.31030245	0	0.60305057	-0.7296491	0.14410987	0.21255725	0	0.7934357	-0.3338148	0.60107773	0.92806272	0
menF	2.32455625	1.21695533	0.00331598	0.00659463	1	1.30568252	0.38480414	0.50506011	0.59294915	0	0.56169108	-0.8321512	0.06636204	0.35344999	0
elaB	2.66045903	1.41167519	0.19291019	0.25009813	0	0.66291058	-0.5931138	0.39635586	0.48733905	0	0.24917151	-2.004789	0.0496651	0.29478208	0
elaA	0.54427011	-0.8776053	0.05501887	0.08265931	0	0.32949818	-1.6016576	0.00638161	0.0141497	-1	0.60539459	-0.7240523	0.25945716	0.75892335	0
rbn	0.62225888	-0.6844132	0.06758703	0.09875352	0	0.63026052	-0.6659798	0.37154829	0.46289029	0	1.01285902	0.01843338	0.56783242	0.92745961	0
elaD	3.56853584	1.83533226	0.04367808	0.06755114	0	2.28131627	1.18986647	0.15679645	0.22826478	0	0.63928636	-0.6454658	0.6774119	0.94128448	0
yfbK	2.61568345	1.38718796	0.0043038	0.00832672	1	3.22296923	1.68839042	0.00289634	0.00701697	1	1.23217098	0.30120246	0.51538378	0.90937129	0
yfbL	8.50894831	3.08898083	2.94E-08	1.46E-07	1	8.13350551	3.02387728	2.63E-06	1.41E-05	1	0.95587671	-0.0651035	0.95346802	1	0
yfbM	0.59292401	-0.7540809	0.21925168	0.28046182	0	0.48994474	-1.0293091	0.13338399	0.19890539	0	0.82631962	-0.2752282	0.65806704	0.93664426	0
yafO	4.41922451	2.14379323	0.00195205	0.00411366	1	1.49018278	0.5754893	0.446007	0.53586323	0	0.33720459	-1.5683039	0.05499085	0.31535713	0
yfbN	16.0674138	4.00606583	8.76E-06	3.04E-05	1	8.84099597	3.1442089	0.00161504	0.00421303	1	0.55024387	-0.8618569	0.36955239	0.85484012	0
yfbO	7.27497935	2.86294315	5.50E-08	2.65E-07	1	5.08214967	2.34543886	0.00031909	0.00099369	1	0.69857926	-0.5175043	0.28862305	0.79106754	0
yfbP	1.25161532	0.32379123	0.341474	0.41086643	0	1.22851302	0.29691314	0.54078071	0.62623427	0	0.98154201	-0.0268781	0.93182411	1	0
nuoN	0.27080819	-1.8846567	1.52E-09	8.97E-09	-1	0.32201598	-1.6347958	2.96E-06	1.56E-05	-1	1.18909248	0.24986093	0.75051243	0.95683847	0
nuoM	0.44338638	-1.1733636	5.41E-05	0.00016142	-1	0.45681138	-1.1303295	0.0011433	0.00311483	-1	1.03027833	0.04303413	1	1	0
nuoL	0.38613459	-1.3728243	4.06E-06	1.50E-05	-1	0.42726568	-1.2267947	0.00047753	0.00140773	-1	1.10652009	0.14602964	0.78106005	0.9635071	0
nuoK	0.41825698	-1.2575385	0.00019011	0.00049953	-1	0.50167554	-0.9951735	0.01377926	0.02769011	0	1.19944334	0.26236501	0.58782248	0.92806272	0
nuoJ	0.49015812	-1.0286809	0.0013072	0.00285837	-1	0.51752501	-0.9502995	0.00717209	0.0156976	0	1.05583279	0.07838138	1	1	0
nuoI	0.42186753	-1.2451381	5.91E-05	0.00017464	-1	0.40295955	-1.3112931	0.00063878	0.00184253	-1	0.95518029	-0.066155	0.86102155	0.98424873	0
nuoH	0.36177517	-1.4668347	1.94E-06	7.52E-06	-1	0.33772058	-1.566098	1.78E-05	7.76E-05	-1	0.93350956	-0.0992633	0.64305761	0.93208609	0
yafP	3.70214365	1.88836088	0.00505692	0.00963608	1	3.49278046	1.80437597	0.00668993	0.01475927	1	0.94344812	-0.0839849	0.81055319	0.96998188	0
nuoG	0.29250006	-1.7734912	6.44E-09	3.52E-08	-1	0.25495639	-1.9716776	9.47E-08	7.02E-07	-1	0.87164562	-0.1981864	0.49493943	0.90539057	0
nuoF	0.36186013	-1.4664959	2.49E-06	9.47E-06	-1	0.26584798	-1.9113266	5.67E-07	3.49E-06	-1	0.73467054	-0.4448307	0.20536948	0.68468503	0
nuoE	0.35444066	-1.496384	1.53E-06	6.04E-06	-1	0.20809268	-2.2647019	1.16E-08	1.02E-07	-1	0.58710161	-0.7683179	0.04032367	0.25809488	0
nuoC	0.34370031	-1.5407769	2.41E-07	1.05E-06	-1	0.21967754	-2.1865407	1.42E-08	1.23E-07	-1	0.63915433	-0.6457638	0.09129047	0.43807505	0
nuoB	0.4665714	-1.0998302	0.00015776	0.00042231	-1	0.24839548	-2.0092892	1.70E-08	1.45E-07	-1	0.5323847	-0.909459	0.00375977	0.04445196	0
nuoA	1.47035184	0.55616142	0.20270613	0.26172258	0	0.66264226	-0.5936979	0.02603597	0.04858654	0	0.45066918	-1.1498593	0.00078289	0.01403468	-1
lrhA	0.23406836	-2.0949982	6.07E-10	3.81E-09	-1	0.43649167	-1.195974	0.00030582	0.00096188	-1	1.86480428	0.89902422	0.10945275	0.48954021	0
alaA	0.46961576	-1.0904473	0.00389781	0.0076281	-1	0.60429634	-0.7266719	0.10953865	0.16836567	0	1.28678887	0.36377536	0.34579599	0.84391827	0
yfbR	0.41748809	-1.2601931	0.00187767	0.00396637	-1	0.3589404	-1.4781838	0.00457866	0.01052162	-1	0.85976202	-0.2179907	0.74637566	0.95683847	0
yfbS	0.20054257	-2.3180196	1.43E-11	1.07E-10	-1	0.22993911	-2.1206762	3.69E-07	2.38E-06	-1	1.14658503	0.19734334	0.51037902	0.90925684	0
ykfJ	4.72987668	2.24180257	0.03279499	0.05203142	0	4.75416797	2.24919288	0.02437249	0.0459088	1	1.00513571	0.00739031	0.72650336	0.95487077	0
yfbT	0.44389021	-1.1717252	8.98E-05	0.00025294	-1	0.1860107	-2.4265425	7.18E-10	7.75E-09	-1	0.41904663	-1.2548173	0.00076851	0.01383322	-1
yfbU	0.15320563	-2.7064588	6.21E-16	6.89E-15	-1	0.08068031	-3.6316396	4.98E-18	1.91E-16	-1	0.52661452	-0.9251808	0.00876075	0.08472569	0
yfbV	0.72586491	-0.462227	0.40821488	0.48159325	0	0.62596622	-0.6758433	0.11712548	0.17786168	0	0.86237289	-0.2136163	0.44032555	0.88087171	0
ackA	0.09023019	-3.4702459	4.47E-25	9.92E-24	-1	0.14556562	-2.7802584	7.99E-13	1.31E-11	-1	1.61326955	0.6899875	0.0736434	0.37957754	0
pta	0.0988766	-3.338227	3.28E-23	6.09E-22	-1	0.13756198	-2.8618463	2.52E-13	4.52E-12	-1	1.39124912	0.47638078	0.23753166	0.72998439	0
yfcC	0.81278839	-0.2990483	0.4136011	0.48677752	0	0.80147653	-0.3192678	0.76835955	0.8219271	0	0.98608264	-0.0202195	0.72258816	0.95399541	0
yfcD	0.38459476	-1.378589	0.00017708	0.00046864	-1	0.41518705	-1.2681666	0.00556634	0.0125179	-1	1.07954423	0.11042236	0.70849885	0.95069307	0
yfcE	0.41374565	-1.273184	0.00029226	0.0007339	-1	0.31960715	-1.6456284	0.0001893	0.00062858	-1	0.77247254	-0.3724445	0.40291346	0.86854717	0
yfcF	1.27688316	0.35262652	0.40446021	0.47729114	0	0.92086439	-0.1189394	0.84964327	0.8865977	0	0.72118141	-0.4715659	0.35326908	0.84775072	0
yfcG	3.66954408	1.87560083	1.06E-05	3.60E-05	1	1.53617922	0.61934654	0.24586984	0.33124964	0	0.41862945	-1.2562543	0.01030269	0.09525131	0
prfH	12.7389872	3.67117868	1.01E-13	9.18E-13	1	9.55016826	3.25552615	9.78E-09	8.69E-08	1	0.74968034	-0.4156525	0.40018658	0.86854717	0
folX	0.43562758	-1.1988328	0.00029434	0.00073827	-1	0.33093186	-1.5953939	4.68E-05	0.00018397	-1	0.75966693	-0.3965611	0.17160149	0.62594091	0
yfcH	0.30367525	-1.7193988	3.20E-07	1.38E-06	-1	0.18883939	-2.4047684	2.79E-09	2.73E-08	-1	0.62184649	-0.6853696	0.04943522	0.29426243	0
yfcI	0.82821226	-0.2719275	0.26956601	0.3361584	0	0.63634375	-0.6521218	0.13763353	0.20402636	0	0.76833413	-0.3801943	0.43711409	0.88087171	0
hisP	4.27755886	2.09678771	1.86E-06	7.25E-06	1	3.3680599	1.7519178	0.00179869	0.00462336	1	0.78737897	-0.3448699	0.40190793	0.86854717	0
hisM	3.97555358	1.99115576	7.62E-05	0.0002194	1	2.91110818	1.54156845	0.00243499	0.00603829	1	0.73225228	-0.4495873	0.64331451	0.93208609	0
hisQ	2.93405931	1.55289803	0.01802142	0.03028942	1	2.02310548	1.01657154	0.09543299	0.1505062	0	0.6895244	-0.5363265	0.66985989	0.93979017	0
hisJ	0.47473983	-1.074791	0.0015753	0.00338758	-1	0.29475978	-1.7623884	4.56E-05	0.00017978	-1	0.62088699	-0.6875974	0.10352086	0.47259525	0
argT	3.21911086	1.68666226	0.04154601	0.06452487	0	0.98233374	-0.0257148	0.94234108	0.96037944	0	0.30515685	-1.7123771	0.06555529	0.35213014	0
ubiX	2.29462109	1.19825594	0.01445114	0.0249436	1	2.86236645	1.51720838	0.01185389	0.02435256	1	1.24742445	0.31895245	0.60658088	0.92806272	0
purF	0.68332757	-0.5493508	0.1654759	0.21889998	0	1.33395904	0.41571437	0.44446743	0.53424864	0	1.95215164	0.96506512	0.04347061	0.27028009	0
pepD	0.10517235	-3.2491726	8.62E-22	1.46E-20	-1	0.10370227	-3.2694806	1.13E-16	3.35E-15	-1	0.98602219	-0.020308	0.63827093	0.93163024	0
cvpA	0.64965664	-0.6222507	0.26923707	0.33584302	0	0.52263062	-0.9361365	0.11844348	0.17952536	0	0.80447205	-0.3138858	0.53771817	0.9187668	0
dedD	0.4259953	-1.2310906	0.00076888	0.00177344	-1	0.53707257	-0.8968111	0.04124607	0.07308195	0	1.26074764	0.33427952	0.40292421	0.86854717	0
folC	0.49552673	-1.0129652	0.00248037	0.00508625	-1	0.59399019	-0.751489	0.171939	0.24708893	0	1.19870462	0.2614762	0.24074097	0.73370247	0
accD	0.18365918	-2.4448971	7.33E-14	6.76E-13	-1	0.3499219	-1.5148951	1.62E-05	7.18E-05	-1	1.90527858	0.93000196	0.03137544	0.21619641	0
dedA	3.91332888	1.96839636	8.40E-08	3.92E-07	1	1.50822297	0.59284973	0.4533983	0.54267797	0	0.38540665	-1.3755466	0.0002119	0.00479218	-1
truA	1.25317207	0.32558452	0.53886991	0.60641742	0	1.44707164	0.53313635	0.30285175	0.3930402	0	1.15472701	0.20755183	0.64073673	0.93163024	0
usg	0.72724875	-0.4594792	0.25270791	0.31744741	0	0.97284288	-0.0397213	0.79845513	0.84597888	0	1.33770307	0.41975792	0.44074526	0.88087171	0
pdxB	0.41212348	-1.2788514	6.81E-05	0.00019877	-1	0.4791909	-1.0613276	0.01035766	0.02170246	-1	1.16273625	0.21752387	0.47730451	0.89855925	0
flk	1.66862789	0.73866227	0.04725309	0.07239574	0	1.96671338	0.97578672	0.01332841	0.02690598	0	1.17864108	0.23712445	0.42049707	0.87375001	0
yfcJ	2.51813053	1.33235307	0.00159747	0.00342858	1	3.30832412	1.72610059	0.00073763	0.00209755	1	1.31380168	0.39374752	0.42820099	0.87784805	0
gpt	0.46427525	-1.1069477	0.00592728	0.01113603	-1	1.10707701	0.14675558	0.84282618	0.88110927	0	2.38452732	1.25370331	0.0279111	0.19981812	0
fabB	0.40076747	-1.3191627	1.48E-05	4.89E-05	-1	0.22832628	-2.1308312	1.78E-08	1.50E-07	-1	0.56972259	-0.8116685	0.01805781	0.1442571	0
mnmC	0.54968875	-0.8633131	0.00860663	0.0156057	0	0.54881655	-0.8656041	0.05596319	0.0945703	0	0.99841329	-0.002291	0.83537244	0.98014902	0
yfcL	0.40743547	-1.2953565	0.00331357	0.00659282	-1	0.35810402	-1.4814043	0.01595877	0.03156751	-1	0.87901041	-0.1860478	0.95671947	1	0
epmC	0.55114218	-0.8595035	0.05382935	0.08114821	0	0.5854647	-0.7723459	0.16846968	0.2433635	0	1.06227526	0.08715764	0.81993575	0.97464061	0
yfcA	1.03308745	0.04696238	0.83511405	0.86752088	0	1.52714204	0.61083425	0.27145381	0.35957866	0	1.47823114	0.56387187	0.31598933	0.81560659	0
mepA	0.86983651	-0.2011838	0.82734354	0.86086605	0	1.56842337	0.64931504	0.19476766	0.27383973	0	1.80312432	0.85049887	0.10225727	0.46828094	0
aroC	0.53685599	-0.897393	0.01789648	0.03013676	0	0.6833312	-0.5493431	0.24622665	0.33162925	0	1.27283893	0.34804987	0.36159854	0.85310733	0
prmB	0.33436161	-1.5805189	1.36E-05	4.54E-05	-1	0.49111052	-1.0258804	0.03074956	0.05659404	0	1.46880058	0.55463854	0.10607027	0.47927242	0
smrB	0.48053459	-1.0572878	0.00319025	0.00638131	-1	0.55958388	-0.8375737	0.03900065	0.06963389	0	1.16450279	0.2197141	0.71645058	0.95281877	0
yfcO	0.68867653	-0.5381016	0.0802192	0.11466273	0	0.51406326	-0.9599822	0.0133041	0.02687889	0	0.74645097	-0.4218806	0.22554648	0.7094579	0
frsA	0.31404407	-1.6709611	1.38E-06	5.47E-06	-1	0.34535113	-1.5338642	0.00013238	0.00046097	-1	1.09969	0.13709689	0.85125075	0.98212032	0
yfcP	6.58312757	2.71877316	1.86E-08	9.53E-08	1	6.7611362	2.75726571	1.42E-06	8.07E-06	1	1.02704013	0.03849255	1	1	0
yfcQ	2.72059429	1.44392183	0.0590348	0.08785603	0	4.20495473	2.07209027	0.01528484	0.03041139	1	1.54560154	0.62816844	0.40790195	0.86959974	0
yfcR	5.83148584	2.54386352	4.77E-05	0.00014396	1	5.52788697	2.46672812	0.00013783	0.00047884	1	0.94793799	-0.0771354	0.79761755	0.96743774	0
yfcS	2.9279983	1.54991472	0.0358048	0.05638139	0	4.25524134	2.08924096	0.00240066	0.00596655	1	1.45329365	0.53932624	0.24919608	0.74860676	0
yfcU	4.87276058	2.28473934	8.02E-09	4.35E-08	1	5.41160349	2.43605614	1.97E-08	1.64E-07	1	1.11058268	0.1513168	0.52054678	0.91269012	0
yfcV	7.74152278	2.95261738	0.00231591	0.00478232	1	4.97857977	2.31573425	0.01059655	0.0220878	1	0.64310084	-0.6368831	0.86194299	0.98424873	0
sixA	1.18046883	0.23935995	0.568026	0.63309887	0	0.52745228	-0.9228875	0.01302994	0.02643678	0	0.44681593	-1.1622475	0.00233719	0.0319102	-1

fadJ	3.13888357	1.65025151	4.25E-07	1.80E-06	1	1.12345752	0.16794557	0.6963505	0.76508524	0	0.35791628	-1.4823059	9.82E-05	0.00264067	-1
fadI	2.99069766	1.58048207	4.15E-06	1.53E-05	1	1.35623627	0.43960854	0.23763646	0.32218674	0	0.45348491	-1.1408735	0.00551889	0.05978403	0
yfcZ	0.05524481	-4.1780173	8.99E-33	3.48E-31	-1	0.13171952	-2.924459	4.22E-16	1.14E-14	-1	2.38428766	1.2535583	0.0316207	0.21686982	0
ribF	0.40205872	-1.3145219	3.58E-05	0.00011039	-1	0.58827231	-0.765444	0.02629294	0.04904537	0	1.46315026	0.54907793	0.21367906	0.69446947	0
crl	1.4071349	0.49276064	0.60489402	0.66703543	0	0.95705626	-0.0633244	0.60779806	0.68744807	0	0.68014535	-0.556085	0.2947274	0.79886161	0
fadL	0.86608788	-0.2074147	0.62837498	0.68854684	0	0.31464724	-1.6681928	0.0011816	0.00321126	-1	0.36329713	-1.4607781	0.00317973	0.03938936	-1
yfdF	2.01379009	1.00991331	0.11597969	0.15949013	0	2.32308492	1.21604189	0.30871972	0.39924859	0	1.15358842	0.20612858	0.8431183	0.98067952	0
mlaA	0.48699344	-1.0380258	0.00108352	0.0024074	-1	0.46487136	-1.1050966	0.00967176	0.02044953	-1	0.95457417	-0.0670708	1	1	0
yfdC	8.16486071	3.02942827	1.76E-16	2.04E-15	1	5.18912256	2.37549061	6.46E-08	4.96E-07	1	0.63554331	-0.6539377	0.08161877	0.40533646	0
argW	5.13145909	2.3593691	0.03536136	0.05578254	0	4.40471545	2.13904882	0.10055369	0.15717961	0	0.85837486	-0.2203203	0.90499824	0.99890653	0
intS	1.38264077	0.46742637	0.30982942	0.37804325	0	1.34323932	0.42571637	0.31288175	0.40309751	0	0.97150276	-0.04171	0.902334	0.99781668	0
gtrA	0.55914287	-0.8387111	0.38488722	0.45626593	0	1.51884118	0.60297102	0.82516837	0.86716267	0	2.71637405	1.44168216	0.29054487	0.79337639	0
gtrB	0.39616369	-1.3358314	0.02020865	0.03359489	-1	0.94831705	-0.0765586	0.53588759	0.6217084	0	2.39375054	1.25927281	0.17952197	0.64470024	0
gtrS	0.26836554	-1.8977287	1.80E-05	5.83E-05	-1	0.52404428	-0.9322394	0.01986105	0.03839282	0	1.95272568	0.96548929	0.2047275	0.68449451	0
tfaS	1.84900102	0.88674602	0.21620469	0.27701016	0	1.0568272	0.07973951	0.88518239	0.91307679	0	0.57156659	-0.8070065	0.35237951	0.84775072	0
phoE	20.7484595	4.37493232	1.16E-26	2.91E-25	1	13.61405	3.76702441	8.23E-15	1.85E-13	1	0.65614751	-0.6079079	0.06668751	0.35432759	0
yfdK	2.2054609	1.14108018	0.05818877	0.08683101	0	3.21804779	1.68618575	0.04571411	0.07975155	0	1.45912711	0.54510557	0.574742	0.92806272	0
yfdL	11.6981606	3.5482098	1.88E-07	8.34E-07	1	11.6154751	3.53797627	5.49E-06	2.73E-05	1	0.99293175	-0.0102335	1	1	0
yfdM	11.2943116	3.49752444	1.02E-07	4.70E-07	1	12.5267479	3.64694002	3.43E-07	2.23E-06	1	1.10912009	0.14941558	0.68233338	0.94348666	0
yfdN	9.68496258	3.27574648	0.00184802	0.00390934	1	10.2832022	3.36221769	0.0030834	0.00739714	1	1.06176995	0.08647122	0.77364106	0.96287211	0
oweS	10.2251871	3.35405534	7.17E-07	2.95E-06	1	14.38468	3.84646122	2.85E-07	1.90E-06	1	1.40678892	0.49240588	0.3883229	0.86621345	0
yfdP	7.26463038	2.8608894	0.01620071	0.02759681	1	5.64853517	2.49787678	0.03325788	0.06060979	0	0.77753924	-0.3630126	0.98623374	1	0
yfdQ	1.68232493	0.75054638	0.11730621	0.16111366	0	1.5920589	0.67089371	0.43248734	0.52331916	0	0.94634447	-0.0795627	0.74566164	0.95683847	0
yfdR	9.04987282	3.17789752	9.71E-06	3.34E-05	1	6.59901584	2.72225088	0.00044638	0.00133371	1	0.72918327	-0.4556466	0.736888	0.95683847	0
yfdS	5.19840699	2.37809604	0.00898	0.01623604	1	6.5427384	2.70989459	0.02233727	0.04252839	1	1.25860449	0.331825	0.83327376	0.98014902	0
yfdT	13.8448235	3.79127476	1.89E-08	9.65E-08	1	5.31945471	2.41127836	0.00436041	0.01007004	1	0.38421976	-1.3799964	0.04119081	0.26089831	0
proB	0.44326087	-1.1737721	0.00045728	0.00109772	-1	0.55181071	-0.8577546	0.03716197	0.06675559	0	1.24488928	0.31601744	0.38008217	0.85988523	0
ypdJ	3.14434933	1.6527615	0.43535264	0.5065734	0	0.47611835	-1.0706079	1	1	0	0.15142031	-2.7233694	0.62998031	0.93109012	0
torI	0.33228686	-1.5894988	0.01311053	0.02286191	-1	0.68751496	-0.540537	0.28859223	0.3779103	0	2.06904046	1.04896186	0.27909952	0.78147864	0
pawZ	3.54236186	1.82471159	0.30934446	0.37755581	0	0.92011634	-0.1201118	0.7633764	0.8183795	0	0.25974657	-1.9448234	0.64206498	0.93164618	0
dsdC	2.09007326	1.06355351	0.048999	0.0745803	0	2.57641237	1.36536353	0.12100362	0.18293363	0	1.23268998	0.30181001	0.98790064	1	0
dsdX	3.72911926	1.89883494	1.67E-05	5.46E-05	1	4.228646	2.08019579	1.56E-05	6.95E-05	1	1.13395301	0.18136085	0.53739123	0.9187668	0
dsdA	1.48132184	0.56688512	0.08771949	0.12411879	0	1.31273842	0.39257947	0.21847746	0.30104518	0	0.88619393	-0.1743057	0.90742761	0.99909148	0
emrY	6.58923266	2.72011047	0.00056252	0.00133012	1	5.10221593	2.35112396	0.00308259	0.00739714	1	0.77432627	-0.3689865	0.91425795	1	0
emrK	12.0020305	3.5852066	7.40E-13	6.26E-12	1	8.00308764	3.00055671	6.25E-09	5.78E-08	1	0.66681114	-0.5846499	0.50271089	0.90598897	0
evgA	0.31626237	-1.6608062	8.85E-07	3.59E-06	-1	0.37579941	-1.4119653	4.39E-05	0.00017375	-1	1.18825207	0.24884091	1	1	0
evgS	1.54214109	0.62493476	0.16831741	0.22212668	0	1.20161747	0.2649777	0.77987582	0.83122799	0	0.77918777	-0.3599571	0.3152974	0.81560659	0
proA	0.34794504	-1.5230686	6.24E-06	2.22E-05	-1	0.40826479	-1.2924229	0.00202653	0.00513782	-1	1.17336	0.23064572	0.45820878	0.88755478	0
yfdE	7.09484312	2.82677078	5.38E-09	2.99E-08	1	4.21078047	2.07408766	1.11E-05	5.13E-05	1	0.59349874	-0.7526831	0.38727427	0.86562571	0
yfdV	11.1592796	3.48017199	0.00484066	0.00923595	1	5.58427155	2.4813691	0.02440811	0.04595631	1	0.50041506	-0.9988029	0.68685304	0.94389281	0
oxc	5.12776056	2.3583289	0.00116305	0.00257036	1	4.73279904	2.24269366	0.00120722	0.00326481	1	0.92297583	-0.1156352	0.74819515	0.95683847	0
frc	4.47960338	2.163371	0.0001041	0.00028955	1	4.06397253	2.02289065	0.00133665	0.00356898	1	0.90721704	-0.1404804	0.96447292	1	0
yfdX	6.21194813	2.63504578	4.67E-05	0.00014115	1	4.39644646	2.1363379	0.00112315	0.00306938	1	0.70774037	-0.4987079	0.72245637	0.95399541	0
ypdI	3.77421467	1.91617648	0.00359942	0.00710395	1	3.47987585	1.79903584	0.00260802	0.00637437	1	0.92201323	-0.1171406	0.72485598	0.95459348	0
yfdY	1.35424023	0.43748368	0.36519096	0.4360769	0	0.73353286	-0.4470665	0.60678831	0.68678759	0	0.54165638	-0.8845502	0.15784689	0.59957345	0
lpxP	1.74762017	0.80539166	0.12012943	0.16447975	0	3.01194429	1.59069509	0.00810057	0.01744464	1	1.72345476	0.78530343	0.16888709	0.62093602	0
ypdK	0.85827284	-0.2204917	0.75693861	0.80292935	0	1.86870477	0.90203866	0.46437991	0.55379079	0	2.17728522	1.12253041	0.52909842	0.91648924	0
alaC	1.46708613	0.55295357	0.07476434	0.10777875	0	1.04375111	0.06177773	0.81362059	0.85747422	0	0.71144501	-0.4911758	0.21985276	0.70257294	0
thrW	0.30389938	-1.7183344	0.00178307	0.00378634	-1	0.71793342	-0.478078	0.23981692	0.32457917	0	2.36240501	1.24025632	0.1530539	0.591558	0
ypdA	0.93465181	-0.0974991	0.63839342	0.69589924	0	1.33173452	0.41330652	0.41294496	0.50367203	0	1.42484561	0.5108056	0.2275045	0.71459748	0
ypdB	0.33894783	-1.5608649	8.48E-05	0.00024114	-1	0.38381235	-1.381527	0.00254772	0.00624771	-1	1.13236408	0.1793379	0.75909635	0.95807677	0
ypdC	1.35138225	0.43443581	0.34116768	0.41060972	0	0.961209	-0.0570779	1	1	0	0.71127839	-0.4915138	0.38262438	0.86038969	0
fryA	3.33918734	1.73949704	1.16E-05	3.90E-05	1	4.58265845	2.19618476	2.03E-06	1.11E-05	1	1.37238734	0.45668773	0.26038247	0.75894691	0
ypdE	3.23989243	1.69594592	0.00075328	0.00174019	1	5.97832056	2.57974026	2.04E-05	8.78E-05	1	1.84522193	0.88379434	0.12585521	0.5319293	0
ypdF	4.07970802	2.0284659	0.00709131	0.01310558	1	4.06447177	2.02306787	0.01196706	0.0245508	1	0.99626536	-0.005398	0.8078848	0.96990832	0
fryC	3.94185239	1.97887375	0.00436047	0.00842528	1	5.98113495	2.58041927	0.00059328	0.00172253	1	1.51734118	0.60154552	0.30057677	0.80487807	0
fryB	2.43747883	1.28538969	0.08069159	0.11526998	0	2.20463384	1.14053907	0.17984371	0.25619902	0	0.90447302	-0.1448506	0.98427609	1	0
glk	0.21715925	-2.2031747	2.10E-11	1.55E-10	-1	0.2730393	-1.8728195	8.10E-07	4.86E-06	-1	1.25732291	0.33035522	0.49431528	0.90539057	0
yfeO	2.06827397	1.0484273	0.00256371	0.00524497	1	2.20089242	1.13808863	0.00578754	0.01297572	1	1.06412035	0.08966133	0.77727206	0.9635071	0
ykfN	0.53229753	-0.9096952	0.58149055	0.64533763	0	0.66677381	-0.5847307	0.63151212	0.70929898	0	1.25263367	0.32496457	0.99710904	1	0
ypeC	2.34121542	1.22725768	0.01492777	0.02561614	1	1.92244173	0.94293987	0.06875252	0.11366158	0	0.82112979	-0.2843178	0.73401239	0.95599369	0
mntH	4.65368256	2.2183728	3.18E-05	9.86E-05	1	2.27959235	1.18877586	0.03305726	0.06028892	0	0.48984698	-1.0295969	0.13775161	0.55834982	0
nupC	0.08794148	-3.5073124	2.52E-24	5.19E-23	-1	0.29706886	-1.7511307	7.47E-07	4.53E-06	-1	3.37802899	1.75618171	1.72E-05	0.00064833	1
yfeA	1.5444315	0.62707588	0.06872454	0.09995268	0	1.32885531	0.41018403	0.10488108	0.16267795	0	0.86041713	-0.2168918	1	1	0
alaX	1.73404681	0.79414285	0.57734658	0.6419218	0	5.25316263	2.39318625	0.2664589	0.35359816	0	3.02942377	1.5990434	0.46705741	0.89320173	0
alaW	1.0066067	0.0095001	0.75173949	0.79837301	0	4.09659043	2.03442366	0.34472463	0.43511515	0	4.06970314	2.02492356	0.40698305	0.86959974	0
yfeC	0.1749626	-2.5148815	6.06E-09	3.34E-08	-1	0.4501557	-1.151504	0.00392251	0.00918091	-1	2.57286819	1.36337755	0.03455147	0.23131824	0
yfeD	0.33327528	-1.5852138	5.17E-05	0.00015526	-1	0.49503808	-1.0143886	0.00960357	0.02031506	-1	1.48537293	0.57082519	0.41071619	0.87071045	0
gltX	0.34128624	-1.5509459	1.60E-06	6.29E-06	-1	0.52894476	-0.918811	0.00928499	0.01969773	0	1.5498567	0.63213483	0.13723685	0.55728775	0
ykfI	2.713593	1.44020436	0.02665815	0.04322652	1	1.70469857	0.76951666	0.12683171	0.19011849	0	0.62820717	-0.6706877	0.60355563	0.92806272	0
valU	0.40846181	-1.2917269	0.29557456	0.36346125	0	2.44763614	1.29138911	0.52677634	0.61339253	0	5.99232559	2.58311601	0.11270819	0.49654656	0
valX	0.33634749	-1.5719756	0.16500827	0.2184122	0	2.24443219	1.16635051	0.61402853	0.6925452	0	6.6729566	2.73832612	0.0849514	0.41665961	0
valY	0.54330777	-0.8801584	0.46456343	0.53436892	0	3.30259168	1.72359861	0.39787407	0.48879775	0	6.07867556	2.60375702	0.12984218	0.53863917	0
lysV	0	#NAME?	0.96905265	0.97925087	0	21.8627786	4.45040487	0.51006441	0.59757012	0	Inf	Inf	0.42790858	0.87784805	0
xapR	1.5071062	0.59178108	0.13158257	0.17844649	0	2.39638515	1.2608598	0.01226577	0.02506477	1	1.59005726	0.66907872	0.18448403	0.65399884	0
xapB	2.94775788	1.55961803	0.00023807	0.00061208	1	2.28847442	1.19438617	0.00706763	0.015492	1	0.77634409	-0.3652319	0.63366878	0.93163024	0
xapA	7.82822585	2.96868538	3.62E-06	1.35E-05	1	6.90225339	2.78706744	5.90E-05	0.00022452	1	0.88171362	-0.1816179	0.9941689	1	0
yfeN	7.03603757	2.81476319	4.53E-06	1.66E-05	1	6.49160507	2.69857523	6.53E-05	0.00024608	1	0.92262229	-0.116188	0.97129425	1	0
yfeR	1.63035063	0.70518227	0.10761598	0.1491496	0	1.70604215	0.77065329	0.07678855	0.12512117	0	1.04642653	0.06547103	0.69581917	0.94834535	0
yfeH	0.55362912	-0.8530083	0.01847445	0.03094379	0	0.50346219	-0.9900447	0.04488956	0.07846795	0	0.9093853	-0.1370364	0.92834832	1	0
yafW	3.52019263	1.81565438	0.00090261	0.00204348	1	5.2426108	2.39028545	0.00041777	0.00125757	1	1.48929656	0.57463107	0.38826819	0.86621345	0
ypeB	0.74493946	-0.4248049	0.51323218	0.58201452	0	0.75875025	-0.398303	0.40190893	0.49265896	0	1.01853949	0.02650191	0.76977576	0.96177657	0
ligA	0.85254741	-0.230148	0.60244652	0.66508529	0	0.72785416	-0.4582787	0.24828124	0.33368455	0	0.85374039	-0.2281307	0.48772134	0.90208042	0
zipA	0.39274155	-1.3483479	1.89E-05	6.11E-05	-1	0.49709131	-1.0084172	0.0019151	0.00488615	-1	1.26569576	0.33993066	0.66313293	0.93720148	0
cysZ	0.68628327	-0.5431239	0.12448947	0.16981778	0	0.83037434	-0.2681662	0.47200562	0.56130055	0	1.20995859	0.27495767	0.60100747	0.92806272	0
cysK	0.26333068	-1.9250525	6.37E-07	2.64E-06	-1	0.08800814	-3.5062192	8.32E-12	1.18E-10	-1	0.3342115	-1.5811667	0.0007217	0.0131516	-1
ptsH	0.11697952	-3.0956722	1.98E-21	3.27E-20	-1	0.10417011	-3.2629868	6.03E-17	1.88E-15	-1	0.89049869	-0.1673146	0.42123343	0.87377207	0
ptsI	0.16940002	-2.5614941	1.68E-16	1.95E-15	-1	0.122903	-3.0244079	8.40E-16	2.17E-14	-1	0.72551942	-0.4629139	0.08512705	0.41665961	0
crr	0.23221995	-2.1064361	3.60E-12	2.84E-11	-1	0.1136499	-3.1373317	6.36E-17	1.96E-15	-1	0.48940625	-1.0308956	0.00071178	0.01302476	-1
pdxK	0.17073073	-2.5502053	1.07E-11	8.07E-11	-1	0.2166782	-2.2063741	8.07E-07	4.86E-06	-1	1.26912242	0.34383124	0.35943524	0.85183882	0
yfeK	1.29907172	0.37748108	0.61911823	0.67958381	0	0.65377807	-0.6131271	0.70731633	0.77386256	0	0.50326557	-0.9906082	0.36946544	0.85484012	0
ykfH	4.28197159	2.09827523	0.04010658	0.06257533	0	4.8453645	2.2766052	0.04718477	0.08183261	0	1.13157325	0.17832997	0.76177149	0.95858924	0
yfeS	0.95542291	-0.0657886	0.52368866	0.59144281	0	0.53887132	-0.8919873	0.40019692	0.49096896	0	0.5640134	-0.8261987	0.57112773	0.92806272	0
cysM	1.12878266	0.17476773	0.63749346	0.69515052	0	1.2991877	0.37760988	0.29680403	0.38649735	0	1.15096355	0.20284215	0.5186514	0.91161924	0
cysA	0.83069199	-0.2676145	0.62102853	0.68134203	0	0.51595777	-0.9546751	0.10838522	0.16698087	0	0.62111803	-0.6870607	0.23227681	0.72276201	0
cysW	2.57940658	1.3670392	0.00151368	0.00326622	1	3.41477551	1.77179074	0.00034756	0.00107256	1	1.3238609	0.40475154	0.34013249	0.83938683	0
cysU	4.54537388	2.18439897	3.25E-06	1.22E-05	1	4.50438742	2.17133092	3.42E-05	0.00013919	1	0.99098282	-0.0130681	0.84622534	0.98157127	0
cysP	5.24541416	2.39105669	4.98E-05	0.00014992	1	3.30687727	1.7254695	0.00741793	0.01618758	1	0.63043206	-0.6655872	0.4202449	0.87375001	0
ucpA	0.07271434	-3.7816163	8.30E-29	2.43E-27	-1	0.10043247	-3.3157023	9.49E-17	2.85E-15	-1	1.3811921	0.46591399	0.23074173	0.71964005	0
murR	1.22334104	0.29082665	0.70976838	0.75927511	0	1.4992719	0.58426205	0.27343251	0.3614413	0	1.22555514	0.2934354	0.47989809	0.89904443	0
murQ	0.12925076	-2.9517553	1.18E-14	1.17E-13	-1	0.19035575	-2.3932299	4.61E-07	2.92E-06	-1	1.47276313	0.55852542	0.09512919	0.45012846	0
murP	0.41454674	-1.2703933	0.00021853	0.00056512	-1	0.41680027	-1.2625719	0.01017734	0.02138549	-1	1.00543614	0.00782145	0.64222322	0.93164618	0

ykfG	4.0486231	2.01743134	1.38E-05	4.59E-05	1	4.98453212	2.31745809	1.37E-05	6.20E-05	1	1.23116724	0.30002675	0.48028619	0.89935549	0
yfeW	0.36267876	-1.4632358	0.00040936	0.00099239	-1	0.46629916	-1.1006723	0.05201291	0.08888053	0	1.28570848	0.36256357	0.27095851	0.77208217	0
yfeX	0.06466437	-3.9508851	4.10E-29	1.25E-27	-1	0.1174221	-3.0902241	1.06E-14	2.31E-13	-1	1.81587018	0.86066107	0.03093888	0.21452903	0
yfeY	0.43842794	-1.1895884	0.00349835	0.00691994	-1	0.42789402	-1.2246746	0.02244364	0.0427125	-1	0.97597344	-0.0350862	0.9030294	0.99833533	0
yfeZ	1.52811538	0.61175348	0.15272194	0.20386002	0	2.59155295	1.37381687	0.01119399	0.0232018	1	1.69591445	0.76206339	0.15388421	0.59217223	0
ypeA	1.96963801	0.97793051	0.04672023	0.07162904	0	1.70913118	0.77326313	0.15155345	0.22198035	0	0.86773873	-0.2046674	0.73484142	0.95650847	0
amiA	0.34595714	-1.5313348	0.00020507	0.00053564	-1	0.71507434	-0.4838349	0.13266071	0.1981838	0	2.06694489	1.04749992	0.07813939	0.39270146	0
hemF	0.83156691	-0.2660957	0.5115482	0.58085048	0	0.70056438	-0.5134105	0.57947079	0.66270223	0	0.84246303	-0.2473147	0.96300698	1	0
eutR	1.24739685	0.31892052	0.41598427	0.4891913	0	1.28605024	0.36294701	0.22492288	0.30896307	0	1.03098725	0.04402648	0.591699	0.92806272	0
eutK	0.78908964	-0.3417389	0.58540509	0.64891565	0	1.14737259	0.19833395	0.48668157	0.575039	0	1.45404594	0.54007285	0.2117724	0.69333058	0
eutL	0.80286199	-0.3167761	0.63594203	0.69374066	0	1.03243399	0.04604954	0.90408597	0.9267353	0	1.28594204	0.36282562	0.70370917	0.94896888	0
ileS	0.33285934	-1.5870155	2.01E-07	8.87E-07	-1	0.45889096	-1.1237767	0.00169708	0.0043959	-1	1.37863329	0.46323876	0.21363634	0.69446947	0
yafX	2.14274168	1.09945793	0.00125457	0.00275694	1	2.97821767	1.5744492	0.00069308	0.0019785	1	1.38990981	0.47499127	0.37689209	0.85988523	0
eutC	7.91168603	2.98398518	3.81E-07	1.62E-06	1	6.69341731	2.74274296	0.00060113	0.0017419	1	0.84601655	-0.2412422	0.39795025	0.86854717	0
eutB	0.7068247	-0.5005756	0.13167857	0.17852186	0	0.95158377	-0.0715974	0.73869696	0.79881412	0	1.34627974	0.42897822	0.33100692	0.83208693	0
intZ	1.0076016	0.01092532	0.82483976	0.85957919	0	1.17220139	0.22922045	0.47961168	0.56927317	0	1.16335801	0.21829513	0.5573565	0.92299744	0
yffL	1.35722257	0.44065733	0.43238047	0.50404448	0	1.01409008	0.02018581	0.85327078	0.8892933	0	0.74718038	-0.4204715	0.58278753	0.92806272	0
yffM	2.60608471	1.38188398	0.07909685	0.11321152	0	2.81417155	1.49271028	0.08000726	0.12945884	0	1.07984653	0.11082629	0.78352223	0.9635071	0
yffN	2.84902022	1.51046586	0.0684438	0.09957716	0	3.33187197	1.73633297	0.03546068	0.06409029	0	1.16947993	0.22586711	0.59459323	0.92806272	0
yffO	1.6882996	0.75557094	0.22483501	0.28652517	0	2.34607959	1.23025196	0.17070846	0.24580016	0	1.38961094	0.47468101	0.65016102	0.93394466	0
yffP	2.35183655	1.2337878	0.00623004	0.01165526	1	5.19508145	2.37714637	4.99E-05	0.00019476	1	2.20894665	1.14335857	0.05255756	0.30699184	0
yffQ	1.92492147	0.94479959	0.14929918	0.19995588	0	1.34596458	0.42864044	0.28183299	0.37077105	0	0.6992309	-0.5161591	0.84410333	0.98070401	0
yffR	0.82685564	-0.2742926	0.43790679	0.50911814	0	0.59645249	-0.7455209	0.28591172	0.37568953	0	0.72135021	-0.4712283	0.58208771	0.92806272	0
ykfF	3.59389707	1.84554909	0.00173603	0.00369365	1	5.31259159	2.40941581	0.00029722	0.00093749	1	1.47822586	0.56386672	0.31561144	0.81560659	0
yffS	0.50546917	-0.984305	0.01503948	0.02579568	0	0.57491966	-0.7985677	0.08621522	0.13798807	0	1.13739808	0.18573727	0.75070462	0.95683847	0
eutA	5.8897023	2.55819471	0.00086311	0.00196209	1	7.47346706	2.90177769	0.00029362	0.00092723	1	1.26890404	0.34358297	0.45876751	0.88755478	0
eutH	6.11875443	2.613238	6.70E-06	2.37E-05	1	6.6757474	2.73892937	1.77E-05	7.71E-05	1	1.09103045	0.12569137	0.6641167	0.93720148	0
eutG	4.11585614	2.04119256	0.00038022	0.00092941	1	7.16421875	2.84080939	1.70E-05	7.45E-05	1	1.74063877	0.79961683	0.16879981	0.62093602	0
eutJ	5.44113359	2.44390725	2.31E-07	1.02E-06	1	12.7197478	3.66899816	2.54E-11	3.36E-10	1	2.3377018	1.22509091	0.01423287	0.12119871	0
eutE	2.8583633	1.51518929	0.0022891	0.00473358	1	6.18307716	2.62832501	2.40E-05	0.00010148	1	2.16315301	1.11313572	0.07023491	0.36820631	0
eutN	2.58999081	1.37294698	0.33562492	0.40548558	0	3.21598121	1.68525898	0.13386276	0.19950743	0	1.24169599	0.312312	0.56238227	0.92432422	0
eutM	1.24696081	0.31841613	0.48341426	0.55331453	0	0.73144083	-0.4511869	1	1	0	0.58657884	-0.7696031	0.68304334	0.94348666	0
eutD	2.90934913	1.54069643	0.00204365	0.00428827	1	2.93738671	1.55453321	0.0121258	0.02483037	1	1.00963706	0.01383678	0.95586072	1	0
eutT	2.65178052	1.40696137	0.07766392	0.11134098	0	2.58448477	1.3698767	0.04328652	0.07621804	0	0.97462243	-0.0370847	0.70355402	0.94896888	0
ykfB	0.73492844	-0.4443243	0.4266487	0.49881083	0	0.62871822	-0.6695145	0.30170233	0.39177861	0	0.85548223	-0.2251902	0.7868022	0.9635071	0
eutQ	2.21831222	1.14946244	0.11433923	0.15738123	0	2.72938361	1.44857518	0.04758706	0.08240094	0	1.23038749	0.29911274	0.5611114	0.92432422	0
eutP	2.66549428	1.41440309	0.04608036	0.07079547	0	2.60129534	1.37923021	0.05024216	0.08635555	0	0.97591481	-0.0351729	0.80809338	0.96990832	0
eutS	6.56767572	2.7153829	5.29E-05	0.00015852	1	6.30787963	2.65715513	0.00166339	0.0043213	1	0.96044322	-0.0582278	0.84256736	0.98067952	0
maeB	0.56269763	-0.8295682	0.00277951	0.00564463	0	0.35459626	-1.4957508	3.67E-05	0.00014818	-1	0.63017194	-0.6661826	0.05609301	0.31836574	0
talA	4.88231897	2.28756655	0.00044581	0.00107193	1	0.90590642	-0.1425661	0.68459706	0.75611957	0	0.18554839	-2.4301326	0.00082524	0.01453465	-1
tktB	8.38326786	3.06751273	1.04E-09	6.32E-09	1	1.15085214	0.20270249	0.70486909	0.77173533	0	0.13727966	-2.8648102	1.61E-06	8.98E-05	-1
ypfG	3.09727355	1.63099881	9.84E-05	0.00027503	1	1.74927902	0.80676042	0.05385579	0.09158832	0	0.56478028	-0.8242384	0.14822073	0.58206003	0
nudK	0.33492871	-1.578074	8.58E-05	0.0002431	-1	0.30819204	-1.6980985	0.00028284	0.00089597	-1	0.92017206	-0.1200244	0.6772363	0.94128448	0
aegA	0.24093526	-2.0532825	4.19E-09	2.36E-08	-1	0.26459782	-1.9181269	9.08E-06	4.32E-05	-1	1.09821127	0.13515563	0.57713035	0.92806272	0
narQ	0.98604313	-0.0202773	0.7909816	0.8310575	0	1.06242615	0.08736256	0.9594843	0.97202227	0	1.07746417	0.1076399	0.83499847	0.98014902	0
yafY	1.11690721	0.15950933	0.47078284	0.54053751	0	1.43559978	0.52165361	0.32475736	0.41498936	0	1.28533487	0.36214428	0.60092886	0.92806272	0
acrD	2.94326551	1.55741769	7.36E-06	2.59E-05	1	3.44094158	1.7828034	3.22E-06	1.68E-05	1	1.16908977	0.22538571	0.39247921	0.86854717	0
ypfM	0.01604486	-5.9617454	1.46E-50	1.61E-48	-1	0.07526184	-3.7319376	2.45E-21	1.48E-19	-1	4.6907148	2.22980779	2.17E-05	0.00077001	1
yffB	0.17381752	-2.5243546	1.53E-09	9.00E-09	-1	0.21167945	-2.2400469	1.97E-05	8.48E-05	-1	1.21782576	0.28430774	0.39202394	0.86854717	0
dapE	0.58425343	-0.7753338	0.03504758	0.05538647	0	0.51640075	-0.953437	0.0679795	0.11263645	0	0.88386431	-0.1781032	0.96679956	1	0
ypfN	0.72538632	-0.4631786	0.20396592	0.26303713	0	0.62305387	-0.6825712	0.0716592	0.11811339	0	0.85892697	-0.2193926	0.3955403	0.86854717	0
ypfH	0.94695724	-0.0786288	0.9352187	0.95246968	0	0.53726194	-0.8963024	0.11034879	0.16925765	0	0.56735607	-0.8176736	0.11980533	0.51693513	0
tmcA	1.97996027	0.98547148	0.003729	0.00733168	0	1.40466413	0.49022521	0.25373791	0.33988112	0	0.70944057	-0.4952463	0.18135705	0.64917581	0
ypfJ	1.24757802	0.31913004	0.53256313	0.59993148	0	0.71795545	-0.4780338	0.36371073	0.45556625	0	0.5754794	-0.7971638	0.12069902	0.51879402	0
purC	0.64548472	-0.6315451	0.06219513	0.09187823	0	0.55576242	-0.8474598	0.05062016	0.08688299	0	0.86100012	-0.2159147	0.61680964	0.92897598	0
bamC	0.26440579	-1.9191743	2.13E-09	1.24E-08	-1	0.32565187	-1.6185976	2.21E-05	9.40E-05	-1	1.2316367	0.30057676	0.42897963	0.87784805	0
ykfL	2.75720091	1.46320439	0.1070736	0.14852555	0	3.96526828	1.98741848	0.0384649	0.06887212	0	1.43814993	0.52421409	0.48071593	0.89935549	0
dapA	0.20728289	-2.270327	1.01E-12	8.51E-12	-1	0.25148611	-1.9914494	6.75E-08	5.17E-07	-1	1.21325065	0.27887764	0.6094489	0.92806272	0
gcvR	0.24742557	-2.0149335	3.34E-08	1.66E-07	-1	0.33724367	-1.5681367	0.00012192	0.0004276	-1	1.3630106	0.44679678	0.44046548	0.88087171	0
bcp	0.25203748	-1.9882898	2.08E-09	1.21E-08	-1	0.41865156	-1.2561781	0.00035601	0.00109393	-1	1.66106866	0.73211171	0.14857444	0.5824118	0
hyfA	8.35942564	3.06340382	1.44E-07	6.51E-07	1	9.43772624	3.23843932	2.94E-07	1.95E-06	1	1.12899219	0.1750355	0.57275013	0.92806272	0
hyfB	5.93892131	2.57020092	3.24E-09	1.85E-08	1	6.96039624	2.79916944	2.81E-09	2.74E-08	1	1.17199671	0.22896852	0.4110701	0.87071045	0
hyfC	4.25841047	2.09031502	2.73E-05	8.57E-05	1	6.85141048	2.77640102	3.11E-07	2.05E-06	1	1.60891265	0.686086	0.11991488	0.51693513	0
hyfD	5.87470528	2.55451648	5.99E-06	2.14E-05	1	7.60811169	2.92753843	2.94E-06	1.55E-05	1	1.2950627	0.37302195	0.4086973	0.86959974	0
hyfE	7.94540184	2.99012019	1.08E-06	4.33E-06	1	6.7817004	2.76164705	8.61E-05	0.00031472	1	0.85353775	-0.2284731	0.80614029	0.96990832	0
hyfF	4.17367722	2.06131903	0.00082767	0.00188931	1	4.00178943	2.00064526	0.00333236	0.00794695	1	0.95881622	-0.0606738	1	1	0
hyfG	2.26559199	1.17988807	0.00554536	0.01049192	1	2.5771731	1.36578944	0.00459693	0.01055265	1	1.13752746	0.18590137	0.5752931	0.92806272	0
ykfK	3.04700559	1.60739215	0.17660458	0.23174761	0	5.18040954	2.37306616	0.03663535	0.06591678	0	1.70016411	0.76567401	0.35813828	0.85142308	0
hyfH	8.19794301	3.03526196	2.33E-11	1.71E-10	1	8.99891156	3.16975051	1.97E-08	1.64E-07	1	1.0977036	0.13448855	0.9582407	1	0
hyfI	5.40800489	2.43509646	1.89E-08	9.63E-08	1	6.17639503	2.62676503	6.87E-07	4.17E-06	1	1.14208385	0.19166857	0.80968569	0.96990832	0
hyfJ	3.79006905	1.92222413	0.00858362	0.01557677	1	2.60165238	1.37942821	0.02563096	0.04795225	1	0.68643931	-0.5427959	0.8735827	0.98834554	0
hyfR	3.85315172	1.94603899	0.00025563	0.00065042	1	4.19668845	2.06925136	0.00017643	0.00059334	1	1.08915733	0.12321237	0.56505142	0.92545162	0
focB	2.85440563	1.51319037	0.00016872	0.00044867	1	3.56159132	1.83252198	4.97E-05	0.00019428	1	1.24775234	0.31933161	0.36918646	0.85484012	0
yfgO	0.93839062	-0.0917395	0.7908047	0.8310575	0	1.19612669	0.2583702	0.36979898	0.46097086	0	1.27465754	0.3501097	0.26239293	0.76128474	0
bepA	0.48979846	-1.0297399	0.00161902	0.0034681	-1	0.54309312	-0.8807285	0.05684039	0.09590596	0	1.10880937	0.14901135	0.48214622	0.89935549	0
yfgD	0.28814471	-1.7951346	3.01E-07	1.30E-06	-1	0.24915383	-2.0048913	1.28E-05	5.85E-05	-1	0.86468299	-0.2097568	0.75886582	0.95807677	0
hda	0.68602579	-0.5436653	0.09269086	0.13038711	0	0.75861226	-0.3985654	0.29395293	0.38397222	0	1.10580719	0.14509986	0.76191787	0.95858924	0
uraA	2.33950427	1.22620286	0.00214885	0.0044735	1	2.39915769	1.26252799	0.00243313	0.00603705	1	1.02549832	0.03632512	0.70747913	0.95005571	0
yafZ	5.26865009	2.39743337	0.00021637	0.00056034	1	7.37625443	2.88288842	7.94E-05	0.00029255	1	1.40002739	0.48545505	0.39826034	0.86854717	0
upp	0.07100786	-3.8158775	3.10E-23	5.77E-22	-1	0.16953434	-2.5603506	2.77E-09	2.72E-08	-1	2.3875433	1.2555269	0.00305201	0.03823681	1
purM	2.34446002	1.22925567	0.00115836	0.00256335	1	2.76331488	1.46639997	0.00102283	0.00282846	1	1.17865729	0.23714429	0.54541971	0.91914725	0
purN	1.19579252	0.25796709	0.46272191	0.53266684	0	1.20392682	0.2677477	0.43645881	0.52682641	0	1.00680244	0.00978062	0.86402251	0.98520074	0
ppk	0.44242345	-1.1765002	0.00100624	0.00225842	-1	0.34111964	-1.5516503	0.0001844	0.00061315	-1	0.77102523	-0.37515	0.32810454	0.8290655	0
ppx	0.43590426	-1.1979168	0.00083699	0.00190861	-1	0.47364176	-1.0781318	0.00780651	0.01692281	-1	1.08657291	0.11978499	0.86775385	0.98621446	0
yfgF	4.05411961	2.01938865	3.62E-08	1.80E-07	1	3.05071464	1.60914724	4.83E-05	0.00018941	1	0.75249744	-0.4102414	0.47863513	0.89894957	0
yfgG	0.306405	-1.7064883	1.93E-05	6.22E-05	-1	0.40002448	-1.3218398	0.00056342	0.00164231	-1	1.30554162	0.38464845	1	1	0
yfgH	3.58361202	1.84141445	0.05836062	0.08705803	0	3.16384569	1.66167923	0.17728409	0.25336946	0	0.88286502	-0.1797352	0.83944205	0.98067952	0
yfgI	1.16182629	0.21639438	0.90572023	0.92694584	0	1.93892461	0.95525671	0.23068148	0.31556545	0	1.66885931	0.73886233	0.29241664	0.795532	0
guaA	0.14113	-2.8249034	9.30E-17	1.11E-15	-1	0.24279344	-2.0421986	3.66E-07	2.36E-06	-1	1.72035322	0.78270481	0.02501926	0.18419859	0
ykfA	3.05382907	1.61061931	8.59E-06	2.99E-05	1	3.84752422	1.94393041	3.54E-06	1.82E-05	1	1.25990163	0.3333111	0.35591628	0.84842747	0
guaB	0.36573619	-1.4511247	0.00026996	0.00068256	-1	0.6806443	-0.555027	0.31311961	0.40316388	0	1.86102528	0.89609765	0.01874323	0.14760294	0
xseA	1.53207099	0.61548314	0.10070175	0.14075938	0	2.2090004	1.14339368	0.01582937	0.0313677	1	1.44183946	0.52791053	0.27672406	0.77913531	0
yfgJ	0.84425631	-0.244247	0.44417704	0.51467208	0	0.79457172	-0.3317506	0.25477895	0.34094727	0	0.94114988	-0.0875036	0.53289824	0.91800048	0
der	0.4388564	-1.1881791	0.00018953	0.0004986	-1	0.54381446	-0.8788136	0.03174917	0.05821559	0	1.23916264	0.30936555	0.35407043	0.84775072	0
bamB	0.44314738	-1.1741415	0.00017982	0.00047505	-1	0.42550172	-1.2327631	0.00132641	0.00354806	-1	0.96018106	-0.0586216	0.8770635	0.98956956	0
yfgM	0.42443664	-1.2363789	0.00012313	0.00033613	-1	0.33659188	-1.5709277	0.00013673	0.00047537	-1	0.7930321	-0.3345488	0.44143457	0.88087171	0
hisS	0.42736971	-1.2264434	5.79E-05	0.00017146	-1	0.51232899	-0.9648576	0.01028307	0.02157688	0	1.19879572	0.26158584	0.44044338	0.88087171	0
ispG	0.27683106	-1.8529223	2.39E-09	1.38E-08	-1	0.21163611	-2.2403423	8.98E-09	8.08E-08	-1	0.76449554	-0.38742	0.28782252	0.79034703	0
rodZ	0.42332419	-1.2401652	0.00021146	0.00055006	-1	0.33606342	-1.5731946	0.00010465	0.00037535	-1	0.79386773	-0.3330294	0.40834619	0.86959974	0
rlmN	2.23299619	1.15898079	0.30702443	0.37503509	0	5.7975141	2.53543442	0.11170619	0.17092439	0	2.59629377	1.37645363	0.40760277	0.86959974	0
perR	3.10873101	1.63632579	0.00048301	0.00115446	1	2.60521811	1.38140416	0.00398077	0.00929758	1	0.83803266	-0.2549216	0.91846659	1	0
ndk	0.58198286	-0.7809514	0.27987383	0.34666368	0	2.22647394	1.15476072	0.23206057	0.31679816	0	3.82566923	1.93571214	0.02419016	0.1795936	0

pbpC	2.3906389	1.25739623	8.90E-05	0.00025087	1	3.66683207	1.8745342	1.98E-06	1.09E-05	1	1.53382933	0.61713796	0.10768194	0.48409953	0
yfhM	0.57263505	-0.8043121	0.00946509	0.01699483	0	0.50448507	-0.9871165	0.00679596	0.0149633	0	0.88098882	-0.1828044	0.54717597	0.91914725	0
sseA	0.22145475	-2.1749161	1.72E-11	1.27E-10	-1	0.1582208	-2.6599888	6.03E-12	8.67E-11	-1	0.71446107	-0.4850727	0.106292	0.47951706	0
ryfA	0.12654208	-2.9823109	2.57E-09	1.48E-08	-1	0.08537077	-3.550114	1.34E-09	1.39E-08	-1	0.67464335	-0.5678031	0.1595907	0.60308053	0
sseB	0.09549431	-3.3884414	1.14E-14	1.14E-13	-1	0.08976455	-3.4777104	8.62E-13	1.41E-11	-1	0.93999889	-0.089269	0.38825719	0.86621345	0
pepB	0.36328682	-1.4608191	1.36E-06	5.40E-06	-1	0.27941301	-1.8395289	5.29E-06	2.64E-05	-1	0.76912509	-0.3787098	0.45127857	0.88755478	0
iscX	0.38500757	-1.3770413	0.00034952	0.0008615	-1	0.30213811	-1.7267199	0.00023258	0.0007557	-1	0.78475889	-0.3496786	0.36133782	0.85310733	0
fdx	0.33970334	-1.5576527	9.41E-06	3.24E-05	-1	0.18827644	-2.4090756	3.66E-07	2.36E-06	-1	0.55423783	-0.8514229	0.07623417	0.38642837	0
hscA	0.37162998	-1.4280612	2.46E-05	7.80E-05	-1	0.334773	-1.5787449	0.0001195	0.00042077	-1	0.90082346	-0.1506837	0.73221484	0.95562813	0
insN	0.97866282	-0.0311162	0.81436286	0.85036424	0	0.97246751	-0.040278	0.73690859	0.79795247	0	0.99366963	-0.0091618	0.84854038	0.98167799	0
hscB	0.62819594	-0.6707135	0.0703243	0.10211125	0	0.66382464	-0.5911259	0.24826443	0.33368455	0	1.05671589	0.07958755	0.79790029	0.96743774	0
iscA	0.64814361	-0.6256146	0.03597849	0.05661454	0	0.26839627	-1.8975635	1.78E-05	7.75E-05	-1	0.4141	-1.2719489	0.00342903	0.04178382	-1
iscU	0.5355776	-0.9008325	0.00160148	0.00343553	0	0.14353655	-2.80051	6.91E-11	8.51E-10	-1	0.26800327	-1.8996775	5.75E-06	0.00026411	-1
iscS	0.68581828	-0.5441017	0.0481413	0.07352701	0	0.26951414	-1.8915672	1.09E-06	6.37E-06	-1	0.39298185	-1.3474654	0.0003218	0.00682275	-1
iscR	9.29090154	3.21581859	4.30E-08	2.10E-07	1	3.57961268	1.83980349	0.03265555	0.05960549	0	0.38528152	-1.3760151	0.01115676	0.10123728	0
trmJ	0.25507733	-1.9709934	7.36E-09	4.00E-08	-1	0.43950635	-1.1860441	0.00186175	0.00476931	-1	1.72303182	0.78494934	0.05528098	0.31619863	0
suhB	0.75090866	-0.4132907	0.36051899	0.43108077	0	1.28922044	0.36649896	1	1	0	1.71688051	0.77978964	0.45067827	0.88755478	0
yfhR	25.5048129	4.67269761	1.06E-20	1.62E-19	1	10.8075965	3.43397381	1.31E-08	1.13E-07	1	0.42374733	-1.2387238	0.00599249	0.06398764	0
csiE	0.43362682	-1.2054741	0.00013457	0.00036487	-1	0.2871756	-1.7999949	1.32E-06	7.53E-06	-1	0.6622644	-0.5945208	0.05403288	0.3120921	0
hcaT	1.3162671	0.39645228	0.23249318	0.29517746	0	1.26277544	0.3365981	0.26879982	0.35627667	0	0.95936109	-0.0598542	0.90712362	0.99909148	0
lspA	0.460303	-1.1193442	0.00032559	0.00080793	-1	0.68313695	-0.5497533	0.13084156	0.19553224	0	1.48410275	0.56959098	0.15729826	0.59902934	0
insI1	0	#NAME?	0.97732894	0.98468566	0	0	#NAME?	1	1	0	NA	NA	0.1274523	NA	0
hcaR	2.71846372	1.44279157	0.00044452	0.00106941	1	1.57898569	0.6589981	0.12353296	0.185804	0	0.58083751	-0.7837935	0.1274523	0.53479032	0
hcaE	2.99676509	1.583406	5.67E-05	0.00016852	1	2.89612943	1.53412608	0.00028908	0.00091506	1	0.96641857	-0.0492799	0.88140131	0.99128314	0
hcaF	1.9992753	0.99947714	0.12610539	0.17180984	0	2.138076	1.09631314	0.07263689	0.1193687	0	1.06942551	0.09683599	0.63593391	0.93163024	0
hcaC	3.99154821	1.99694843	0.0180205	0.03028942	1	5.59413855	2.48391598	0.00705908	0.01548093	1	1.40149593	0.48696755	0.49537372	0.90539057	0
hcaB	3.90702998	1.96607233	0.00250448	0.00513331	1	4.14997201	2.0531016	0.00162815	0.00423722	1	1.06218074	0.08702928	0.61466252	0.92897598	0
hcaD	2.69921346	1.43253907	0.00565237	0.01066033	1	2.71843371	1.44277565	0.01054529	0.02201211	1	1.00712068	0.01023657	0.84843331	0.98167799	0
yphA	1.34054618	0.42282092	0.29923365	0.36693921	0	0.8558079	-0.2246411	0.75837204	0.81459554	0	0.6384024	-0.647462	0.22870651	0.71633217	0
yphB	2.84207481	1.50694453	0.00011807	0.00032392	1	3.97668727	1.99156711	2.90E-05	0.00012038	1	1.39921977	0.48462258	0.29986385	0.8048689	0
yphC	3.76087671	1.91106901	6.39E-06	2.27E-05	1	4.01774907	2.00638746	3.17E-05	0.00012999	1	1.06830119	0.09531845	0.71592062	0.95281877	0
eyeA	0.2484186	-2.0091549	0.06806926	0.09919563	0	0.91079367	-0.1348038	1	1	0	3.66636669	1.87435108	0.08510602	0.41665961	0
yphD	5.71320282	2.51429975	6.01E-06	2.14E-05	1	6.6173777	2.72625963	4.07E-06	2.07E-05	1	1.1582606	0.21195988	0.49451269	0.90539057	0
yphE	4.69032762	2.2296887	9.94E-09	5.28E-08	1	6.53053703	2.70720163	3.03E-09	2.94E-08	1	1.39234134	0.47751293	0.2484737	0.74826869	0
yphF	3.14947468	1.65511121	0.00155897	0.00335573	1	3.37352832	1.75425827	0.00189325	0.0048332	1	1.07114	0.09914706	0.69039825	0.94554543	0
yphG	5.86069728	2.55107232	1.81E-06	7.07E-06	1	6.11193387	2.61162893	1.23E-05	5.67E-05	1	1.04286804	0.06055661	0.77269202	0.9628697	0
yphH	1.47631875	0.56200425	0.07624682	0.10962963	0	1.45502951	0.54104841	0.15065397	0.22095582	0	0.98557951	-0.0209558	1	1	0
glyA	0.07382196	-3.7598062	1.43E-28	4.07E-27	-1	0.14385295	-2.7973333	1.06E-12	1.70E-11	-1	1.94864716	0.96247289	0.00553104	0.05978403	0
hmp	18.6354042	4.2199742	2.84E-05	8.88E-05	1	6.44637478	2.68848807	0.01598434	0.03158981	1	0.34592085	-1.5314861	0.16513824	0.61352961	0
glnB	0.69169912	-0.5317835	0.09207084	0.1296388	0	0.62311631	-0.6824266	0.15190653	0.2223867	0	0.90084877	-0.1506432	0.84058138	0.98067952	0
glrR	0.86738993	-0.2052474	0.46811061	0.53788881	0	0.84660607	-0.2402373	0.8337616	0.87411448	0	0.97603861	-0.0349899	0.76269172	0.95858924	0
yfhG	0.86083498	-0.2161914	0.73772457	0.78613497	0	1.2263156	0.29433031	0.38579907	0.47688003	0	1.42456524	0.5105217	0.22037857	0.70323407	0
ykfC	1.61627187	0.69266989	0.02231571	0.03679302	0	2.11755098	1.0823967	0.01078282	0.02243375	1	1.31014529	0.38972682	0.41021388	0.87015066	0
glrK	0.9757119	-0.0354729	0.82570251	0.86002474	0	1.20577204	0.26995719	0.50421729	0.59211698	0	1.23578696	0.30543006	0.54357404	0.91914725	0
glmY	0.53511924	-0.9020677	0.01775442	0.02994321	0	0.3262857	-1.6157924	0.00355086	0.00840452	-1	0.60974391	-0.7137247	0.20282146	0.68122061	0
purL	1.26682011	0.34121167	0.2193445	0.28049932	0	1.96707678	0.97605327	0.00480882	0.010982	0	1.55276726	0.6348416	0.06002046	0.33223226	0
mltF	1.67553386	0.74462084	0.04828218	0.07371669	0	3.29066322	1.71837838	0.00054857	0.00160538	1	1.9639491	0.97375754	0.05156582	0.30320704	0
tadA	0.66968598	-0.5784433	0.10828371	0.14988692	0	0.78774783	-0.3441942	0.43702722	0.52722449	0	1.17629433	0.2342491	0.61665526	0.92897598	0
pgpC	0.58242616	-0.7798529	0.00823829	0.01499938	0	0.39151745	-1.3528515	0.00273473	0.00666561	-1	0.67221817	-0.5729986	0.28161757	0.78561671	0
yfhH	0.69537264	-0.5241418	0.20791811	0.26743572	0	0.69562185	-0.5236248	0.40284876	0.49367406	0	1.00035839	0.00051696	0.82617421	0.97697604	0
yfhL	1.32991254	0.41133137	0.46956696	0.53928166	0	2.92816423	1.54999647	0.07305076	0.11991505	0	2.20177203	1.1386651	0.19646874	0.67373807	0
shoB	0.15882462	-2.6544936	0.00014117	0.00038161	-1	0.14827257	-2.7536764	0.00026804	0.00085338	-1	0.93356162	-0.0991828	0.49695171	0.90539057	0
ohsC	1.8496459	0.8872491	0.86541103	0.89352981	0	6.44895221	2.68906478	0.49780814	0.58536833	0	3.48658747	1.80181567	0.61420925	0.92897598	0
insH1	7.48868797	2.90471298	0.15806545	0.21004064	0	10.1342581	3.34116858	0.23632533	0.32093474	0	1.35327552	0.4364556	1	1	0
acpS	0.37181442	-1.4273454	0.00026361	0.00066917	-1	0.25615598	-1.9649055	0.00040179	0.00121694	-1	0.68893502	-0.5375602	0.44825431	0.88755478	0
pdxJ	0.41498478	-1.2688697	6.90E-05	0.00020065	-1	0.31734565	-1.655873	3.11E-05	0.00012805	-1	0.76471636	-0.3870034	0.29815572	0.80359844	0
recO	1.31248984	0.39230625	0.61394499	0.67474372	0	0.7204249	-0.47308	0.78353316	0.83472348	0	0.54889941	-0.8653863	0.42900822	0.87784805	0
era	0.35287518	-1.5027701	3.40E-05	0.00010512	-1	0.42643574	-1.2295997	0.00353383	0.00837768	-1	1.20846057	0.2731704	0.57983809	0.92806272	0
rnc	0.48359979	-1.0481145	0.00084714	0.00192743	-1	0.74730069	-0.4202392	0.16392084	0.23741927	0	1.54528745	0.62787523	0.19223369	0.66728286	0
lepB	0.491794	-1.023874	0.00475735	0.00908876	-1	0.80216461	-0.3180298	0.36809611	0.45919268	0	1.63109881	0.70584418	0.1197545	0.51693513	0
lepA	0.43301798	-1.2075012	0.00015686	0.00042094	-1	0.60538033	-0.7240863	0.04882402	0.08424574	0	1.39804897	0.48341489	0.21770493	0.70102268	0
rseC	0.92045384	-0.1195827	0.59881759	0.66157511	0	1.18080352	0.23976893	0.63427781	0.71193133	0	1.28284925	0.35935165	0.38050363	0.85988523	0
rseB	0.59344747	-0.7528078	0.00937425	0.01684543	0	0.48297031	-1.0499936	0.00218002	0.0054767	-1	0.81383836	-0.2971858	0.28113945	0.78519632	0
rseA	0.31946501	-1.6462701	1.21E-07	5.52E-07	-1	0.23011419	-2.1195781	9.47E-09	8.47E-08	-1	0.72031108	-0.473308	0.10542567	0.47782856	0
mmuP	6.44163511	2.68742694	6.49E-12	5.02E-11	1	8.66893008	3.11585395	1.58E-12	2.48E-11	1	1.34576547	0.42842701	0.23549273	0.72813869	0
rpoE	0.4058394	-1.3010192	1.73E-05	5.65E-05	-1	0.3397485	-1.5574609	4.71E-06	2.37E-05	-1	0.83715013	-0.2564417	0.21894919	0.70102268	0
nadB	2.33144367	1.22122358	0.00386567	0.00757528	1	2.41786412	1.27373317	0.00287931	0.00697953	1	1.03706735	0.05250959	0.62001358	0.92907233	0
trmN	3.31630092	1.72957492	0.00090157	0.00204217	1	3.35673794	1.74705991	0.00310795	0.00744619	1	1.01219341	0.01748499	0.90718228	0.99909148	0
srmB	0.55756381	-0.8427912	0.02614889	0.04246125	0	0.77855154	-0.3611355	0.32981968	0.41929607	0	1.39634518	0.48165562	0.32368171	0.82367936	0
yfiE	2.38506568	1.25402899	0.00508336	0.0096781	1	2.10325792	1.07262578	0.04525374	0.07907327	0	0.88184487	-0.1814032	0.66463878	0.93733835	0
eamB	2.71770054	1.4423865	0.00150905	0.00325782	1	3.03187015	1.60020797	0.00175069	0.00450834	1	1.11560126	0.15782147	0.67622689	0.94128448	0
grcA	0.023758	-5.3954428	3.52E-50	3.61E-48	-1	0.09972407	-3.3259144	1.23E-19	5.89E-18	-1	4.19749427	2.06952835	1.99E-06	0.00010307	1
ung	0.22627956	-2.1438218	8.62E-09	4.65E-08	-1	0.17973639	-2.4760456	7.77E-08	5.88E-07	-1	0.79431119	-0.3322238	0.53159522	0.91754792	0
yfiF	0.35130835	-1.5091902	1.25E-05	4.18E-05	-1	0.38506118	-1.3768404	0.00086561	0.00242097	-1	1.09607752	0.13234984	0.72730406	0.95487077	0
trxC	1.45684741	0.54284977	0.51664678	0.58512803	0	1.4911824	0.57645673	0.78613089	0.83688567	0	1.02356801	0.03360696	0.75195684	0.95683847	0
mmuM	0.34175303	-1.548974	2.77E-05	8.69E-05	-1	0.50863535	-0.9752964	0.03677684	0.06614443	0	1.48831261	0.57367758	0.11140088	0.49465734	0
yfiP	2.06345638	1.04506294	0.13960337	0.18811108	0	4.22422061	2.07868518	0.03103158	0.05704182	0	2.0471577	1.03362224	0.30859752	0.81055096	0
pka	0.11632863	-3.1037219	2.60E-20	3.89E-19	-1	0.15628302	-2.677767	5.72E-12	8.29E-11	-1	1.34346142	0.42595489	0.31430242	0.81404427	0
pssA	0.16074563	-2.6371486	2.10E-16	2.40E-15	-1	0.17051037	-2.5520686	5.32E-11	6.76E-10	-1	1.06074655	0.08507999	0.87108373	0.987273	0
yfiM	1.21470542	0.28060649	0.54836717	0.61538053	0	0.87422771	-0.193919	0.55177883	0.63597044	0	0.71970347	-0.4745255	0.25049187	0.7487007	0
kgtP	4.12448877	2.04421496	1.14E-05	3.85E-05	1	1.71181948	0.77553057	0.60322098	0.68367107	0	0.41503808	-1.2686844	0.00220038	0.03080531	-1
rrfG	2.62161723	1.39045706	0.4468821	0.51712817	0	7.15449112	2.83884916	0.25284636	0.33909805	0	2.72903726	1.44839209	0.56929408	0.92806272	0
gltW	0.07817595	-3.6771314	0.30108203	0.36910334	0	2.43347118	1.28301569	0.79018072	0.83998283	0	31.1281326	4.96014713	0.24337979	0.74021027	0
rrsG	4.81982643	2.26898119	0.23516539	0.2977144	0	14.2013027	3.82795137	0.02872995	0.05309814	0	2.94643446	1.55897017	0.24799391	0.74826869	0
clpB	0.68743449	-0.5407059	0.04885058	0.07443015	0	0.36755278	-1.4439767	0.00011029	0.00039208	-1	0.53467318	-0.9032708	0.01237916	0.10874916	0
afuC	2.12340278	1.08637805	0.02391722	0.03911231	1	2.50966974	1.32749753	0.00840816	0.01799305	1	1.18190942	0.24111947	0.4768005	0.89855925	0
ryfD	0.29118992	-1.7799677	0.00029495	0.00073897	-1	0.15865388	-2.6560453	2.30E-05	9.76E-05	-1	0.54484674	-0.8760776	0.08567625	0.4179268	0
yfiH	0.96977272	-0.0442814	0.95615733	0.96887978	0	0.97937696	-0.0300638	0.92287184	0.94336587	0	1.0099036	0.01421759	0.88225684	0.99177993	0
rluD	0.89545935	-0.1593001	0.71243242	0.76194044	0	0.85459712	-0.2266836	0.33491866	0.42504467	0	0.95436729	-0.0673835	0.53148333	0.91754792	0
bamD	0.31124659	-1.6838701	4.38E-07	1.85E-06	-1	0.50707854	-0.9797189	0.00497913	0.0113183	0	1.62918585	0.70415119	0.1338811	0.54820394	0
raiA	0.13308032	-2.9096308	1.26E-15	1.36E-14	-1	0.08860491	-3.4964696	2.59E-14	5.31E-13	-1	0.66580022	-0.5868387	0.14759344	0.58035154	0
pheL	0.23430891	-2.0935162	0.01850282	0.03096892	-1	0.41088664	-1.2831877	0.22643711	0.31056039	0	1.75361077	0.81032857	0.49800452	0.90539057	0
pheA	0.47291607	-1.0803439	0.0027577	0.00560808	-1	0.44690712	-1.1619531	0.01391089	0.02792923	-1	0.94500304	-0.0816091	1	1	0
tyrA	0.86321959	-0.2122005	0.69309661	0.74504934	0	0.81712237	-0.291376	0.84976772	0.8865977	0	0.9465985	-0.0791755	0.87256782	0.98793943	0
aroF	0.84514736	-0.2427252	0.49420907	0.56362548	0	0.66498166	-0.5886135	0.25490739	0.34094727	0	0.78682334	-0.3458883	0.53257174	0.91800048	0
yfiL	2.28095152	1.18963578	0.06570912	0.0963283	0	0.59686812	-0.7445159	0.44091819	0.53090395	0	0.26167506	-1.9341517	0.01540835	0.12772709	0
afuB	5.62289344	2.49131271	7.66E-05	0.00022029	1	5.36629056	2.42392517	0.00034573	0.00106837	1	0.95436462	-0.0673875	0.88785251	0.99411016	0
yfiR	2.2150583	1.14734467	0.10606783	0.14723494	0	2.64652913	1.40410153	0.10844877	0.16698087	0	1.19478983	0.25675686	0.83312375	0.98014902	0
yfiN	1.08758768	0.12113171	0.64861247	0.7049511	0	1.26022869	0.33368556	0.63019095	0.70824359	0	1.15873756	0.21255385	0.8591886	0.98424873	0
yfiB	2.24555972	1.16707509	0.00637291	0.0118823	1	1.66749596	0.73768326	0.38919927	0.4802769	0	0.74257475	-0.4293918	0.15928798	0.60245284	0
rplS	0.02715858	-5.2024483	3.25E-20	4.83E-19	-1	0.08530535	-3.5512199	3.41E-09	3.27E-08	-1	3.14100961	1.65122836	0.01111275	0.10104583	0
trmD	0.03771547	-4.7286996	4.09E-22	7.09E-21	-1	0.13714408	-2.8662358	4.77E-08	3.74E-07	-1	3.63628139	1.86246385	0.0003346	0.00699324	1

rimM	0.03309398	-4.9172874	3.75E-26	9.10E-25	-1	0.16767675	-2.5762454	8.23E-08	6.18E-07	-1	5.06668448	2.34104199	2.12E-06	0.00010877	1
rpsP	0.04813442	-4.3767871	4.97E-21	7.84E-20	-1	0.21941191	-2.1882863	2.77E-06	1.48E-05	-1	4.55831572	2.18850085	4.35E-05	0.00137054	1
ffh	0.29531064	-1.7596948	1.75E-07	7.83E-07	-1	0.34592549	-1.5314668	7.39E-05	0.00027467	-1	1.1713953	0.22822801	0.61915378	0.92897598	0
ypjD	0.92584474	-0.1111578	0.78244776	0.82424797	0	1.13899057	0.18775581	0.73819672	0.7987491	0	1.23021768	0.29891361	0.56052306	0.92432422	0
yfjD	1.13196103	0.17882429	0.45570466	0.52592863	0	1.54500453	0.62761106	0.14281892	0.21079385	0	1.36489198	0.44878678	0.33799307	0.83714543	0
grpE	0.11723503	-3.0925243	3.33E-20	4.94E-19	-1	0.16511538	-2.5984535	8.65E-12	1.22E-10	-1	1.40841334	0.4940708	0.36563731	0.85342842	0
nadK	0.39559541	-1.3379024	0.00012372	0.00033733	-1	0.29405274	-1.7658531	0.00021488	0.0007065	-1	0.74331687	-0.4279508	0.52818702	0.91626596	0
recN	0.91127951	-0.1340345	0.66612253	0.72078961	0	1.58201576	0.66176397	0.26137376	0.34852463	0	1.73603789	0.79579843	0.1140088	0.50027742	0
bamE	0.32893839	-1.6041107	2.36E-05	7.51E-05	-1	0.52476512	-0.9302563	0.00665692	0.01470847	0	1.59532951	0.67385444	0.42005325	0.87375001	0
ratB	0.45930593	-1.1224727	0.02762148	0.04459213	-1	0.89499334	-0.1600511	0.53534876	0.62157282	0	1.94857781	0.96242154	0.21585356	0.69799995	0
ratA	0.63872251	-0.6467388	0.06148999	0.09092779	0	0.73961442	-0.4351547	0.14383207	0.21221833	0	1.15795891	0.21158406	0.97674563	1	0
smpB	0.507643	-0.9781138	0.00223973	0.00464019	0	0.41495204	-1.2689835	0.00014963	0.00051456	-1	0.81740916	-0.2908697	0.15337295	0.59175389	0
ssrA	0.285898	-1.8064276	3.33E-05	0.00010318	-1	0.33950113	-1.5585117	0.00030005	0.00094574	-1	1.1874904	0.24791585	0.72817319	0.95488877	0
intA	0.27789956	-1.8473645	4.04E-08	1.99E-07	-1	0.2315241	-2.1107657	5.35E-07	3.31E-06	-1	0.83312151	-0.2634012	0.5136498	0.90937129	0
yfjH	7.53808667	2.91419838	3.92E-08	1.94E-07	1	3.93653299	1.97692557	0.02030908	0.03912198	1	0.52221912	-0.9372728	0.02675182	0.1937201	0
alpA	4.62953983	2.2108688	0.33875177	0.40825828	0	1.57844509	0.65850407	0.5238828	0.61098921	0	0.34095075	-1.5523647	0.77414712	0.96287211	0
yfjI	4.03327788	2.01195281	0.03540622	0.05583337	0	3.18911104	1.67315433	0.04173169	0.073824	0	0.79069956	-0.3387985	0.95237039	1	0
yfjJ	2.52843268	1.33824337	0.19070962	0.24782712	0	1.95745615	0.96897999	0.18028788	0.25666629	0	0.77417768	-0.3692634	0.94664849	1	0
yfjK	0.60909086	-0.7152706	0.04762498	0.07288966	0	0.56062257	-0.8348983	0.04981248	0.08571703	0	0.92042519	-0.1196276	0.75841004	0.95806023	0
yfjL	0.31666202	-1.6589842	1.71E-07	7.69E-07	-1	0.30482549	-1.7139445	5.16E-05	0.00020038	-1	0.96262093	-0.0549603	0.8687578	0.98621446	0
yfjM	3.05836569	1.61276092	0.01770818	0.02987664	1	3.08374111	1.62468165	0.03117071	0.05722617	0	1.00829705	0.01192073	0.91041237	0.99971546	0
rnlA	0.35831974	-1.4806806	1.04E-05	3.55E-05	-1	0.32449177	-1.6237462	2.57E-05	0.00010768	-1	0.90559277	-0.1430657	0.4812484	0.89935549	0
rnlB	1.2090593	0.273885	0.78465281	0.82637374	0	0.87680802	-0.1896671	0.32335412	0.41369481	0	0.72519853	-0.4635521	0.1769141	0.63784355	0
yfjP	4.64719762	2.216361	2.37E-06	9.07E-06	1	4.88174877	2.28739805	5.63E-06	2.79E-05	1	1.05047152	0.07103705	0.6631151	0.93720148	0
yfjQ	8.13413315	3.02398861	4.43E-11	3.13E-10	1	6.92611565	2.79204648	1.76E-08	1.48E-07	1	0.85148786	-0.2319421	0.87579609	0.98905525	0
fkpB	0.52940631	-0.9175527	0.02948888	0.04722712	0	0.8887785	-0.1701042	0.84238394	0.88085533	0	1.67882112	0.74744852	0.08029252	0.40055431	0
insI1	1.32040669	0.40080753	0.98971016	0.99625109	0	2.60903731	1.38351758	0.5946363	0.67532711	0	1.9761741	0.98271005	0.61081889	0.92897598	0
yfjR	2.54677245	1.34867007	0.1080443	0.14964932	0	2.08731091	1.06164551	0.0862079	0.13798807	0	0.81959066	-0.2870246	0.84376775	0.98070401	0
ypjK	3.06396311	1.61539893	0.1500311	0.20081436	0	7.44439448	2.89615451	0.00959462	0.02030585	1	2.42966192	1.28075558	0.17154074	0.62594091	0
yfjS	3.23701802	1.6946654	0.00207517	0.00435028	1	5.51354224	2.46297949	0.00015129	0.00051785	1	1.7032782	0.7683141	0.20811046	0.69005046	0
yfjT	2.96526891	1.56816294	0.01074643	0.01908604	1	2.84140515	1.50660456	0.01570991	0.03117293	1	0.95822849	-0.0615584	0.83136728	0.97899324	0
yfjU	1.54260461	0.62536832	0.41004249	0.48323311	0	2.19446322	1.13386809	0.31542161	0.40542411	0	1.42257012	0.50849977	0.70649275	0.94959861	0
yfjV	1.81032472	0.8562485	0.10299632	0.14337685	0	2.59441872	1.37541134	0.01025163	0.0215219	1	1.43312341	0.51916285	0.23712807	0.72975213	0
yfjW	1.24974438	0.32163304	0.97017535	0.97993701	0	0.75822833	-0.3992957	0.52207745	0.60952714	0	0.60670673	-0.7209288	0.40453421	0.86854717	0
ypjI	10.1227536	3.33952988	0.00472224	0.0090334	1	7.29108296	2.86613312	0.00792668	0.01714606	1	0.72026676	-0.4733968	1	1	0
yfjX	4.36548894	2.12614325	0.01127454	0.01991189	1	3.40824632	1.76902961	0.02209765	0.04218122	1	0.78072499	-0.3571136	1	1	0
yfjY	7.57237636	2.92074612	1.10E-07	5.06E-07	1	10.15442	3.34403594	4.12E-08	3.25E-07	1	1.34098195	0.42328982	0.35417096	0.84775072	0
insX	1.5876632	0.6669049	0.61307524	0.67407845	0	0.89078077	-0.1668577	0.64236207	0.71990819	0	0.56106407	-0.8337626	0.29120522	0.79411569	0
ypjJ	8.9806444	3.16681897	0.00092956	0.00210019	1	12.0031413	3.58534012	0.00055417	0.00161854	1	1.3365568	0.41852115	0.54308814	0.91914725	0
yfjZ	13.0063432	3.70114349	1.98E-12	1.61E-11	1	14.6845585	3.87622799	3.08E-11	4.04E-10	1	1.12903053	0.17508449	0.61422838	0.92897598	0
ypjF	2.3268747	1.21839353	0.10822599	0.14985398	0	1.2999867	0.37849686	0.42555283	0.51638303	0	0.55868358	-0.8398967	0.61429053	0.92897598	0
psaA	2.55014177	1.35057745	0.17717142	0.2322844	0	1.2151584	0.28114439	0.51024401	0.59757012	0	0.47650621	-1.0694331	0.53674429	0.9187668	0
ypjA	4.78924577	2.25979847	9.13E-09	4.90E-08	1	4.55171964	2.1864117	1.55E-07	1.10E-06	1	0.95040427	-0.0733868	0.80869884	0.96990832	0
pinH	9.97089177	3.31772254	3.00E-05	9.35E-05	1	7.70500938	2.94579671	0.00235507	0.00587969	1	0.77275028	-0.3719258	0.63635226	0.93163024	0
ypjC	4.74555184	2.24657586	0.00321657	0.00642588	1	4.37846012	2.13042357	0.00361963	0.00855725	1	0.92264509	-0.1161523	0.81920107	0.97429253	0
ileY	5.64180919	2.49615787	0.27065098	0.33709638	0	13.9990944	3.8072616	0.19950815	0.27873112	0	2.48131299	1.31110372	0.67559869	0.94091029	0
ygaQ	8.14801962	3.02644945	1.89E-08	9.65E-08	1	5.55481534	2.47373895	1.95E-05	8.41E-05	1	0.68173809	-0.5527105	0.50671491	0.90925684	0
csiD	3.89330069	1.96099377	0.00240537	0.00494855	1	1.01605646	0.02298057	0.79850145	0.84597888	0	0.26097559	-1.9380132	0.01629764	0.13359221	0
yagB	4.96933682	2.31305333	6.24E-09	3.43E-08	1	1.67181904	0.7414187	0.32492944	0.41498936	0	0.33642699	-1.5716346	0.00015597	0.00384263	-1
lhgO	2.93959685	1.55561831	0.00670661	0.01244667	1	1.4396347	0.52570278	0.26261643	0.34965412	0	0.48973882	-1.0299155	0.16502255	0.61352961	0
gabD	2.658074	1.41038127	0.00013913	0.00037655	1	0.79551802	-0.3300335	0.50706139	0.59481719	0	0.29928362	-1.7404148	8.27E-05	0.00229478	-1
gabT	3.56701513	1.83471734	2.93E-06	1.11E-05	1	1.20343905	0.26716307	0.4329256	0.52370606	0	0.33737986	-1.5675543	0.00101269	0.01678931	-1
gabP	5.22676225	2.38591754	4.58E-08	2.22E-07	1	2.81633462	1.49381876	0.00039038	0.00118727	1	0.53882968	-0.8920988	0.16948348	0.62233319	0
csiR	4.06944265	2.02483122	2.43E-07	1.06E-06	1	1.64848936	0.72114458	0.17262015	0.24782601	0	0.40508971	-1.3036866	0.00212534	0.02994491	-1
ygaU	3.25180361	1.70124013	0.09735628	0.13647321	0	0.43520118	-1.2002456	0.23890832	0.32354762	0	0.13383378	-2.9014858	0.01248048	0.10942128	0
yqaE	1.50918967	0.59377413	0.48663829	0.5559962	0	0.8314083	-0.2663709	0.53815955	0.62368924	0	0.55089716	-0.8601451	0.21366962	0.69446947	0
ygaV	0.6694544	-0.5789423	0.54942333	0.61593981	0	0.43438069	-1.2029681	0.2678746	0.35526347	0	0.64885777	-0.6240258	0.54870599	0.91937439	0
ygaP	0.83588095	-0.2586306	0.5385285	0.60618766	0	0.77682276	-0.3643426	0.43984232	0.52975285	0	0.92934617	-0.105712	0.76705445	0.96127028	0
stpA	1.50095885	0.58588442	0.43907805	0.50994918	0	2.2199244	1.15051054	0.15943027	0.23167458	0	1.47900417	0.56462612	0.45887186	0.88755478	0
yagA	4.67133956	2.22383632	0.00048925	0.0011675	1	3.61953584	1.8558047	0.00203559	0.00515487	1	0.77483895	-0.3680316	0.95516715	1	0
alaE	0.57511304	-0.7980825	0.07724761	0.11085208	0	0.4393596	-1.1865259	0.08716397	0.13915374	0	0.76395347	-0.3884433	0.66616628	0.93829234	0
ygaC	1.79522689	0.84416619	0.19061757	0.24782712	0	1.10368111	0.14232339	0.72920661	0.79253185	0	0.61478642	-0.7018428	0.39887915	0.86854717	0
ygaM	2.83635491	1.50403807	0.13522089	0.18276337	0	0.54721158	-0.8698293	0.33881385	0.42924839	0	0.19292775	-2.3738674	0.02851778	0.20284423	0
nrdH	5.12400883	2.35727296	6.70E-06	2.37E-05	1	2.64286361	1.40210197	0.01424887	0.02853001	1	0.51578046	-0.955171	0.11956543	0.51693513	0
nrdI	8.13566149	3.02425965	1.17E-10	7.89E-10	1	2.68581066	1.4253576	0.00188455	0.00481377	1	0.33012812	-1.5989021	0.00898684	0.08653271	0
nrdE	5.82770624	2.54292816	6.00E-12	4.66E-11	1	3.48934066	1.80295445	1.44E-05	6.47E-05	1	0.59875027	-0.7399737	0.08151259	0.40526552	0
nrdF	5.89097053	2.55850534	1.07E-05	3.65E-05	1	2.76505844	1.46730997	0.01110958	0.02304847	1	0.46937231	-1.0911954	0.15243067	0.59018372	0
proV	0.24203473	-2.046714	7.50E-09	4.07E-08	-1	0.32543113	-1.6195758	6.65E-05	0.00025024	-1	1.34456378	0.4271382	0.39244345	0.86854717	0
proW	0.43447935	-1.2026405	0.01358287	0.02364822	-1	0.44727243	-1.1607743	0.05485598	0.0930194	0	1.02944463	0.04186624	0.70161147	0.94869712	0
proX	0.06505302	-3.94224	6.67E-28	1.82E-26	-1	0.07033814	-3.829549	6.25E-18	2.34E-16	-1	1.08124316	0.11269101	0.50041044	0.90594357	0
yagE	0.20624377	-2.2775775	9.93E-09	5.28E-08	-1	0.18950069	-2.399725	5.31E-07	3.30E-06	-1	0.91881895	-0.1221475	0.78426457	0.9635071	0
ygaY	2.61325435	1.38584755	0.05738456	0.08574683	0	3.76322616	1.91197	0.01761289	0.03442342	1	1.44005354	0.52612245	0.41677971	0.8719158	0
ygaZ	0.33006571	-1.5991748	3.71E-05	0.00011402	-1	0.4182028	-1.2577254	0.0026167	0.00639205	-1	1.26702893	0.34144946	0.61940851	0.92897598	0
ygaH	0.57788505	-0.7911455	0.18184129	0.23763106	0	0.59564566	-0.7474737	0.17626952	0.25200097	0	1.03073381	0.0436718	0.86518869	0.9856241	0
mprA	0.15897523	-2.6531261	5.49E-14	5.10E-13	-1	0.26666235	-1.9069139	4.27E-07	2.73E-06	-1	1.67738306	0.74621219	0.21805895	0.70102268	0
emrA	0.71183946	-0.4903762	0.12231752	0.16712113	0	0.60944724	-0.7144268	0.29440302	0.38433292	0	0.85615827	-0.2240506	0.92564617	1	0
emrB	1.92746759	0.9467066	0.00609378	0.01143097	0	2.36422854	1.2413695	0.0015184	0.00398827	1	1.22659833	0.2946629	0.36637147	0.85342842	0
luxS	0.41153106	-1.2809268	0.00101814	0.00228279	-1	0.16583991	-2.5921368	5.53E-08	4.29E-07	-1	0.40298273	-1.3112101	0.00175163	0.02600907	-1
micA	0.49912401	-1.0025298	0.01668775	0.02832814	-1	0.1536703	-2.7020898	2.87E-06	1.53E-05	-1	0.30788	-1.69956	0.00174162	0.02597927	-1
gshA	0.37637857	-1.4097436	4.88E-06	1.77E-05	-1	0.33417141	-1.5813398	1.64E-05	7.22E-05	-1	0.8878598	-0.1715962	0.50042597	0.90594357	0
yqaA	0.30514112	-1.7124515	2.07E-05	6.63E-05	-1	0.27749458	-1.8494685	0.00016226	0.00055024	-1	0.90939753	-0.137017	0.74712023	0.95683847	0
yagF	0.22913933	-2.125703	7.19E-09	3.91E-08	-1	0.18618279	-2.4252083	1.31E-08	1.14E-07	-1	0.81253094	-0.2995053	0.27707255	0.77922796	0
yqaB	0.20538445	-2.2836011	4.75E-10	3.03E-09	-1	0.20326809	-2.2985444	1.26E-06	7.23E-06	-1	0.98969559	-0.0149432	0.86181122	0.98424873	0
argQ	0.25996836	-1.943592	0.07402815	0.10678718	0	0.48156495	-1.0541977	0.38490829	0.47617854	0	1.85239828	0.88939433	0.45164827	0.88755478	0
argZ	0.47070554	-1.0871032	0.20543549	0.26462776	0	3.76004043	1.91074817	0.16652218	0.241029	0	7.98809462	2.99785142	0.01204616	0.10710485	0
argY	0.57819243	-0.7903784	0.28849505	0.35564685	0	3.40064057	1.76580653	0.04783791	0.08273805	0	5.88150307	2.5561849	0.00245483	0.0326828	1
argV	0.66924732	-0.5793886	0.44476914	0.5151513	0	2.56703849	1.36010493	0.24097348	0.32594484	0	3.83570978	1.93949357	0.05519497	0.31611664	0
serV	0.37187965	-1.4270923	0.1646331	0.21798094	0	2.37905374	1.25038786	0.47030427	0.55974867	0	6.39737542	2.67748015	0.04865189	0.29310768	0
csrA	0.40253988	-1.3127964	0.00027285	0.00068868	-1	0.17934378	-2.4792004	2.25E-08	1.87E-07	-1	0.44553046	-1.166404	0.00158471	0.02437244	-1
alaS	0.17567248	-2.5090399	7.51E-15	7.63E-14	-1	0.23951387	-2.0618189	6.01E-08	4.63E-07	-1	1.36341143	0.44722098	0.229037	0.71685817	0
recX	5.05631324	2.33808584	0.00417353	0.00809951	1	6.4292817	2.68465756	0.0157531	0.0312446	1	1.27153548	0.34657172	0.95358857	1	0
recA	0.32202223	-1.6347678	1.04E-07	4.79E-07	-1	0.49825498	-1.0050439	0.00226652	0.00568432	-1	1.54726886	0.62972391	0.14765406	0.58035154	0
yagG	1.78564385	0.83644436	0.48321022	0.55322436	0	1.01451053	0.02078384	0.68709899	0.75774655	0	0.56814831	-0.8156605	0.66379234	0.93720148	0
pncC	0.36175747	-1.4669053	0.0012663	0.00277718	-1	0.53916455	-0.8912025	0.06522906	0.1087315	0	1.49040335	0.57570283	0.35352883	0.84775072	0
mltB	1.87900573	0.90996946	0.0184808	0.03094379	0	2.62433035	1.39194934	0.00082361	0.00231082	1	1.39665905	0.48197988	0.16981505	0.62286269	0
srlA	0.12066446	-3.0509273	3.33E-15	3.45E-14	-1	0.20740973	-2.2694445	5.57E-07	3.44E-06	-1	1.71889659	0.78148276	0.0494441	0.29426243	0
srlE	0.30129057	-1.7307726	8.78E-07	3.57E-06	-1	0.46543142	-1.1033595	0.03345297	0.06088864	0	1.5447925	0.62741307	0.02907191	0.20546011	0
srlB	0.29194449	-1.776234	0.00021755	0.00056293	-1	0.48948892	-1.0306519	0.06191938	0.10384127	0	1.67665061	0.74558209	0.14467577	0.57607933	0
srlD	0.16109647	-2.6340032	2.52E-12	2.02E-11	-1	0.29505507	-1.7609439	5.02E-05	0.00019568	-1	1.83154268	0.87305932	0.02906517	0.20546011	0
srlM	0.45214515	-1.1451421	0.03568024	0.05622534	0	0.57199751	-0.8059192	0.1713529	0.24664322	0	1.26507497	0.33922288	0.67069527	0.93979017	0
srlR	0.11329609	-3.1418301	1.14E-17	1.47E-16	-1	0.1761553	-2.5050803	3.07E-09	2.97E-08	-1	1.55482241	0.63674981	0.16373939	0.61194129	0
srlQ	0.47266044	-1.081124	0.00051968	0.00123677	-1	0.57372755	-0.8015623	0.04650595	0.08090937	0	1.21382605	0.27956168	0.38358528	0.8613091	0

norR	10.1706332	3.3463376	1.76E-08	9.06E-08	1	9.03040149	3.17479013	4.60E-06	2.31E-05	1	0.8878898	-0.1715475	0.72859016	0.95488877	0
yagH	0.90521927	-0.1436608	0.65030938	0.70627405	0	1.00948911	0.01362535	0.95550826	0.96999564	0	1.11518738	0.15728614	0.70268563	0.94896888	0
norV	2.74767711	1.45821248	0.0008037	0.00184031	1	3.28445236	1.71565284	0.00031337	0.00098071	1	1.19535601	0.25744036	0.43498069	0.88087171	0
norW	1.96583043	0.97513888	0.07235204	0.10464275	0	2.6477079	1.40474397	0.00390061	0.00914419	1	1.34686485	0.42960509	0.20170243	0.67864297	0
hypF	0.68865215	-0.5381527	0.143329	0.19271969	0	1.05708648	0.08009341	0.50149466	0.5893896	0	1.53500789	0.61824607	0.04711139	0.28656723	0
hydN	4.05740524	2.0205574	0.00416516	0.00808681	1	3.54809473	1.82704453	0.01032993	0.02166492	1	0.87447384	-0.1935129	1	1	0
ascG	2.40108966	1.26368928	0.00833279	0.0151652	1	2.83903983	1.50540309	0.00161632	0.00421387	1	1.18239642	0.24171381	0.38133487	0.85988523	0
ascF	1.79137643	0.84106853	0.01226786	0.02150283	0	2.0563445	1.04008198	0.00814095	0.01751453	1	1.14791312	0.19901346	0.52788106	0.91615721	0
ascB	1.0839136	0.11624976	0.82912108	0.86210636	0	1.12762638	0.17328913	0.33778406	0.42831213	0	1.04032865	0.05703937	0.47369304	0.89828993	0
hycI	1.10997922	0.15053267	0.98007998	0.98700713	0	0.84199702	-0.248113	0.65595645	0.73014397	0	0.75857008	-0.3986456	0.52134595	0.91269012	0
hycH	3.81146668	1.93034626	4.24E-05	0.00012903	1	4.06969226	2.02491971	0.00024471	0.00079223	1	1.06774966	0.09457344	0.81607348	0.97214588	0
hycG	3.50478292	1.80932509	0.00027904	0.00070348	1	4.94211816	2.3051295	7.65E-06	3.69E-05	1	1.41010678	0.49580441	0.18963177	0.66370038	0
yagI	0.53678851	-0.8975743	0.13246737	0.17948109	0	1.05601595	0.07863162	0.73161767	0.79435343	0	1.96728493	0.97620593	0.29230582	0.795532	0
hycF	9.06629105	3.18051248	1.63E-08	8.44E-08	1	7.68626686	2.94228307	5.21E-07	3.24E-06	1	0.84778514	-0.2382294	0.94245711	1	0
hycE	6.9333373	2.79354995	5.13E-13	4.38E-12	1	10.5634221	3.40100537	1.76E-14	3.71E-13	1	1.52356962	0.60745542	0.12043528	0.51816545	0
hycD	9.54955251	3.25543313	5.03E-10	3.19E-09	1	11.8156641	3.56262881	1.49E-09	1.52E-08	1	1.23730029	0.30719568	0.4853303	0.90075713	0
hycC	8.94059374	3.16037064	4.34E-17	5.33E-16	1	13.0390688	3.70476894	6.92E-18	2.55E-16	1	1.45841195	0.54439829	0.15139924	0.58738081	0
hycB	9.58139552	3.2602358	2.40E-09	1.39E-08	1	15.6244403	3.9657326	3.72E-11	4.84E-10	1	1.63070612	0.7054968	0.13580771	0.55250184	0
hycA	7.28988285	2.86589563	4.54E-05	0.00013755	1	4.15053799	2.05329835	0.00822634	0.01765527	1	0.56935593	-0.8125973	0.35767754	0.85078639	0
hypA	0.03641349	-4.7793833	2.78E-34	1.16E-32	-1	0.04884805	-4.3555551	1.03E-19	5.06E-18	-1	1.34148241	0.42382814	0.16832409	0.62014138	0
hypB	0.0224344	-5.4781439	6.75E-46	5.62E-44	-1	0.04839334	-4.3690476	3.29E-23	3.03E-21	-1	2.15710474	1.10909623	0.00180424	0.02661107	1
hypC	0.02260421	-5.4672645	2.89E-37	1.45E-35	-1	0.06174651	-4.0174987	9.45E-17	2.85E-15	-1	2.73163704	1.4497658	0.00082589	0.01453465	1
hypD	0.06671467	-3.9058522	3.05E-28	8.54E-27	-1	0.11243505	-3.1528363	1.17E-14	2.53E-13	-1	1.68531234	0.75301599	0.05473697	0.31430997	0
argF	0.86124732	-0.2155005	0.66909241	0.72329447	0	0.81812431	-0.289608	0.84581227	0.88360388	0	0.94992959	-0.0741075	0.88557475	0.99348376	0
hypE	0.09798957	-3.351228	3.06E-20	4.57E-19	-1	0.16315649	-2.6156718	2.12E-10	2.50E-09	-1	1.66503928	0.73555622	0.09165527	0.43934752	0
fhlA	1.1578896	0.2114977	0.37267934	0.44346031	0	1.169511	0.22590543	0.49114483	0.57922846	0	1.01003671	0.01440774	1	1	0
ygbA	10.387246	3.3767413	3.36E-13	2.91E-12	1	6.68614699	2.74117507	1.45E-07	1.03E-06	1	0.64368813	-0.6355662	0.20985688	0.69013336	0
mutS	25.2152839	4.65622656	0.28556353	0.35272207	0	5.69673041	2.51013414	0.50732428	0.59481719	0	0.22592371	-2.1460924	0.89065606	0.99437803	0
pphB	3.04297403	1.60548202	0.01216336	0.02135356	1	4.88988965	2.28980191	0.00156727	0.00410295	1	1.60694426	0.68431989	0.28107006	0.78519632	0
ygbI	1.02540681	0.03619639	0.86600929	0.89393854	0	1.08456833	0.11712095	0.53587462	0.6217084	0	1.05769565	0.08092456	0.63234086	0.93163024	0
ygbJ	3.05940851	1.61325276	0.0856168	0.12151973	0	3.40996063	1.76975508	0.04904882	0.08460059	0	1.11458166	0.15650232	0.64921048	0.93327989	0
ygbK	2.75891237	1.46409963	0.00940298	0.01689019	1	3.03540836	1.60189062	0.00730925	0.01595829	1	1.1002192	0.13779098	0.63394752	0.93163024	0
ygbL	2.04540676	1.03238777	0.04413398	0.06811313	0	2.34468679	1.22939522	0.01474099	0.02943536	1	1.1463181	0.19700744	0.47370852	0.89828993	0
ygbM	3.66208035	1.87266344	0.00452238	0.00869628	1	3.60244192	1.84897517	0.00918312	0.01950972	1	0.98371461	-0.0236883	0.85581621	0.98266248	0
ispH	0.53192031	-0.910718	0.00354652	0.00700894	0	0.62465167	-0.6788762	0.1285159	0.19231688	0	1.17433317	0.23184178	0.38469409	0.8629201	0
ykgS	1.80601024	0.85280607	0.70108473	0.75162409	0	3.93428911	1.97610298	0.39355873	0.484439	0	2.1784423	1.1232969	0.6002638	0.92806272	0
ygbN	4.62076582	2.20813198	1.40E-07	6.33E-07	1	4.6244944	2.20929564	9.54E-07	5.66E-06	1	1.00080692	0.00116367	0.73868773	0.95683847	0
rpoS	0.47283344	-1.080596	0.00086659	0.001969	-1	0.26177668	-1.9335915	2.06E-06	1.13E-05	-1	0.55363402	-0.8529955	0.02399513	0.17874748	0
nlpD	0.47730119	-1.0670281	0.00046267	0.00110884	-1	0.36245621	-1.4641214	2.25E-05	9.54E-05	-1	0.75938677	-0.3970932	0.13570335	0.55250184	0
pcm	0.33469587	-1.5790773	9.69E-06	3.33E-05	-1	0.24182413	-2.0479699	6.52E-06	3.19E-05	-1	0.72251901	-0.4688925	0.35688532	0.84936009	0
umpG	0.25216747	-1.9875459	1.18E-08	6.23E-08	-1	0.23110014	-2.11341	2.66E-06	1.43E-05	-1	0.91645499	-0.1258641	1	1	0
truD	0.36739968	-1.4445777	1.81E-05	5.86E-05	-1	0.31853092	-1.6504947	1.54E-05	6.87E-05	-1	0.86698747	-0.2059169	0.38104381	0.85988523	0
ispF	0.55404345	-0.851929	0.08762258	0.12404774	0	0.41867962	-1.2560814	0.04091508	0.07265859	0	0.75568011	-0.4041524	0.54372348	0.91914725	0
ispD	1.28350196	0.36008549	0.33936195	0.40875841	0	1.39278601	0.47797362	0.19188147	0.27047025	0	1.08514522	0.11788813	0.62230664	0.92989404	0
ftsB	0.86010005	-0.2174236	0.51203613	0.58110575	0	1.15085364	0.20270437	0.77627533	0.82858953	0	1.33804625	0.42012798	0.40844311	0.86959974	0
ygbE	4.35684615	2.12328417	1.07E-05	3.65E-05	1	3.1671777	1.66319782	0.00502646	0.01142001	1	0.72694275	-0.4600864	0.37059354	0.85484012	0
cysC	1.52379581	0.60766959	0.29099137	0.35842412	0	1.41776632	0.50361977	0.31654753	0.40639847	0	0.93041753	-0.1040498	0.94031274	1	0
cysN	1.0309071	0.04391434	0.99213168	0.99773972	0	0.77361515	-0.370312	0.65053341	0.72593615	0	0.75042179	-0.4142264	0.58050033	0.92806272	0
cysD	1.9718474	0.9795479	0.03000923	0.0479735	0	1.89062898	0.91886627	0.03274847	0.0597504	0	0.958811	-0.0606816	0.78205987	0.9635071	0
iap	4.62630645	2.20986083	0.03089887	0.0493243	1	6.93243605	2.7933624	0.04628974	0.08059667	0	1.49848181	0.58350157	0.8095691	0.96990832	0
ygbF	2.87844695	1.52529062	0.02693894	0.04361777	1	2.01666003	1.0119679	0.0716343	0.11811339	0	0.70060698	-0.5133227	0.89268128	0.99488354	0
ygbT	1.82833813	0.87053291	0.22288658	0.2842882	0	1.26688615	0.34128689	0.25359659	0.33979482	0	0.69291677	-0.529246	0.95386574	1	0
casE	1.36025976	0.44388218	0.50886728	0.57825232	0	1.19011845	0.25110517	0.70290714	0.76999173	0	0.87491998	-0.192777	0.83860001	0.98067952	0
casD	4.24595047	2.08608754	0.0117891	0.0207874	1	3.26716383	1.7080388	0.04487459	0.07846795	0	0.76947761	-0.3780487	0.85462149	0.98236299	0
casC	2.56982236	1.36166864	0.0317817	0.05062379	0	1.670689	0.7404432	0.28150465	0.37052292	0	0.65011848	-0.6212254	0.48380469	0.89986448	0
casB	2.68938468	1.42727613	0.16076671	0.21330926	0	2.6633123	1.41322161	0.06280574	0.10508843	0	0.99030545	-0.0140545	0.6280088	0.93095788	0
casA	4.80937646	2.26584986	2.39E-06	9.14E-06	1	5.19902847	2.37824206	4.51E-06	2.28E-05	1	1.08101924	0.1123922	0.62338632	0.92989404	0
ygcB	1.20375496	0.26754174	0.50455033	0.57423314	0	0.9700186	-0.0439157	0.89297292	0.91878132	0	0.8058273	-0.3114574	0.60930689	0.92806272	0
sokX	6.47673897	2.6952676	0.05255016	0.0794639	0	8.34946468	3.0616837	0.08405719	0.13521977	0	1.28914639	0.3664161	0.88815575	0.99411016	0
cysH	0.78938115	-0.341206	0.42144165	0.49389964	0	0.63268712	-0.6604359	0.41587318	0.50654454	0	0.80149763	-0.3192298	0.84168161	0.98067952	0
cysI	2.05788054	1.04115923	0.00257632	0.00526589	1	1.65874126	0.73008886	0.03264837	0.05960549	0	0.80604351	-0.3110704	0.67553131	0.94091029	0
cysJ	3.15386299	1.65711999	2.86E-05	8.95E-05	1	3.21032439	1.68271908	0.00010782	0.00038548	1	1.0179023	0.02559909	0.75128721	0.95683847	0
queD	0.41415982	-1.2717405	0.0645023	0.09481986	0	1.80321225	0.85056922	0.39021651	0.4812634	0	4.35390442	2.12230974	0.00954941	0.09037106	0
ygcN	0.87847407	-0.1869284	0.90056348	0.92249279	0	1.52242544	0.60637157	0.17920131	0.25565735	0	1.73303401	0.79329997	0.10182216	0.46725882	0
ygcO	0.19691862	-2.3443285	0.00661088	0.01227933	-1	0.60557528	-0.7236218	0.64263674	0.72003341	0	3.07525654	1.62070677	0.05094998	0.30038692	0
ygcP	0.88514	-0.1760224	0.82646642	0.86035875	0	0.69124281	-0.5327355	0.36816467	0.45919268	0	0.78094178	-0.3567131	0.48572545	0.90075713	0
yagJ	0.56966919	-0.8118037	0.05413851	0.08153078	0	0.75226726	-0.4106828	0.55672281	0.64116709	0	1.32053352	0.40112092	0.29891991	0.80400362	0
ygcQ	7.05921045	2.81950683	6.40E-07	2.65E-06	1	7.27176443	2.86230546	6.06E-06	2.99E-05	1	1.03011016	0.04279863	0.79877635	0.96774826	0
ygcR	6.48979389	2.69817266	1.32E-06	5.26E-06	1	6.49882147	2.70017812	7.05E-06	3.43E-05	1	1.00139104	0.00200546	0.76249914	0.95858924	0
ygcS	9.92063893	3.31043304	8.82E-07	3.59E-06	1	10.6159698	3.40816427	3.71E-06	1.90E-05	1	1.07008933	0.09773123	0.75524859	0.9573569	0
ygcU	4.27176464	2.09483216	1.05E-05	3.58E-05	1	4.81896285	2.26872268	1.58E-05	7.02E-05	1	1.12809653	0.17389052	0.56360722	0.92432422	0
ygcW	6.64374604	2.73199693	1.23E-06	4.91E-06	1	6.51791291	2.70441008	1.55E-05	6.91E-05	1	0.98105991	-0.0275869	0.90041942	0.99683105	0
yqcE	4.43156121	2.14781504	0.00025117	0.00064129	1	3.66988199	1.87573367	0.00098958	0.00274338	1	0.82812396	-0.2720814	0.9900714	1	0
ygcE	3.08828852	1.62680754	0.00013544	0.00036701	1	3.46143215	1.79136907	4.25E-05	0.00016885	1	1.12082538	0.16456153	0.43960923	0.88087171	0
queE	2.28260059	1.19067844	0.0938484	0.13180552	0	1.14100869	0.19030978	0.76528129	0.82000129	0	0.49987225	-1.0003687	0.21127292	0.69220919	0
yqcG	2.07317934	1.05184492	0.79734898	0.83615632	0	1.63037804	0.70520653	0.64295602	0.72020853	0	0.78641438	-0.3466384	0.9221842	1	0
ygcG	4.15954288	2.05642499	0.00679113	0.01259296	1	2.49681563	1.32008929	0.03638988	0.06560867	0	0.60026202	-0.7363357	0.68380246	0.943596	0
yagK	1.02630396	0.03745808	0.89472276	0.91842678	0	1.04800762	0.0676492	0.58145115	0.66376456	0	1.0211474	0.03019113	0.57672339	0.92806272	0
eno	0.12677662	-2.9796394	7.67E-21	1.18E-19	-1	0.0811826	-3.6226857	7.73E-20	3.88E-18	-1	0.64035938	-0.6430463	0.05031742	0.29745283	0
pyrG	0.42083428	-1.2486759	6.91E-05	0.00020103	-1	0.37762918	-1.4049579	0.00012833	0.0004483	-1	0.89733465	-0.156282	0.54422984	0.91914725	0
mazG	0.53972771	-0.8896963	0.00404066	0.00787278	0	0.43486596	-1.2013573	0.00188335	0.00481349	-1	0.80571361	-0.311661	0.34498958	0.84269271	0
mazF	0.7328723	-0.4483663	0.24821157	0.31243958	0	0.56176502	-0.8319613	0.10997159	0.16885492	0	0.76652512	-0.383595	0.45077338	0.88755478	0
mazE	0.4326122	-1.2088537	0.03981185	0.0621594	0	0.46305213	-1.1107535	0.15193153	0.2223867	0	1.07036308	0.09810026	0.77364559	0.96287211	0
relA	0.36261046	-1.4635075	7.94E-06	2.78E-05	-1	0.50913585	-0.9738774	0.00873397	0.01863613	0	1.40408483	0.4896301	0.2349093	0.727493	0
rlmD	0.3973997	-1.3313373	5.79E-05	0.00017146	-1	0.4339513	-1.204395	0.00300837	0.00723679	-1	1.0919769	0.12694233	0.7328083	0.95599369	0
barA	2.30104937	1.20229194	0.02077502	0.03440692	1	2.17160701	1.11876305	0.20323651	0.28286681	0	0.94374638	-0.0835289	0.54171314	0.91914725	0
gudD	1.8370098	0.87735932	0.0710462	0.10305782	0	1.73624162	0.79596773	0.08263514	0.13327178	0	0.94514554	-0.0813916	0.8687835	0.98621446	0
gudX	1.15217839	0.2043641	0.38355007	0.45529177	0	1.55850727	0.64016489	0.1334138	0.19890539	0	1.35266144	0.43580079	0.36238934	0.85329623	0
yagL	1.76583233	0.82034836	0.69929074	0.75042934	0	0.8929819	-0.1632972	1	1	0	0.50570028	-0.9836455	0.70567401	0.94896888	0
gudP	1.20801974	0.27264403	0.48189496	0.55200468	0	1.22883999	0.29729707	0.29379672	0.38388164	0	1.01723503	0.02465305	0.66043102	0.93664426	0
yqcA	0.13495258	-2.8894755	7.03E-17	8.51E-16	-1	0.1049718	-3.2519262	4.43E-14	8.94E-13	-1	0.77784211	-0.3624507	0.38128366	0.85988523	0
truC	0.86469651	-0.2097342	0.75297585	0.79928856	0	1.77260145	0.8258682	0.10117144	0.15808931	0	2.04996947	1.03560242	0.04172171	0.26247184	0
yqcC	1.29141097	0.36894819	0.36428049	0.43522502	0	1.29932138	0.37775831	0.700046	0.76841352	0	1.0061254	0.00881012	0.78389468	0.9635071	0
csrB	2.56486348	1.35888204	0.2633336	0.32968948	0	1.38016163	0.46483723	0.95907912	0.97183459	0	0.53810335	-0.8940448	0.35339682	0.84775072	0
syd	0.66653059	-0.585257	0.14342237	0.19278655	0	0.80414119	-0.3144793	0.25331925	0.33962918	0	1.20645804	0.27077774	0.94234794	1	0
queF	0.46669252	-1.0994557	0.0006077	0.0014255	-1	0.51430754	-0.9592968	0.02460955	0.04625673	0	1.10202653	0.14015896	0.64795321	0.93290031	0
ygdH	0.14270773	-2.8088646	3.31E-18	4.52E-17	-1	0.06826444	-3.8727219	6.08E-21	3.49E-19	-1	0.47835139	-1.0638573	0.00325504	0.04009696	-1
sdaC	0.07137417	-3.8084542	3.81E-26	9.19E-25	-1	0.24336513	-2.0388056	1.21E-07	8.76E-07	-1	3.40970897	1.76964861	6.24E-06	0.00027816	1
sdaB	0.0970922	-3.3645008	3.64E-19	5.10E-18	-1	0.2937823	-1.7671806	2.93E-05	0.00012143	-1	3.02580737	1.59732015	3.91E-05	0.00125098	1
yagM	2.24703745	1.16802417	0.11950637	0.1638298	0	2.44768388	1.29141725	0.11528315	0.17536532	0	1.08929376	0.12339308	0.78646465	0.9635071	0
ygdG	0.37424242	-1.417955	0.00106355	0.0023678	-1	0.59214278	-0.755983	0.11975536	0.18123251	0	1.58224387	0.66197198	0.21778205	0.70102268	0
fucO	0.53418823	-0.9045799	0.01020169	0.01819907	0	0.58676852	-0.7691366	0.08696093	0.13898023	0	1.09843027	0.13544328	0.63462978	0.93163024	0

fucA	0.46105662	-1.1169842	0.00691675	0.01280439	-1	0.49012441	-1.0287801	0.06746826	0.11187326	0	1.06304603	0.08820406	0.70213262	0.94894418	0
fucP	0.34724382	-1.5259791	9.79E-05	0.00027374	-1	0.55212955	-0.8569213	0.14192226	0.20954042	0	1.5900342	0.6690578	0.05671212	0.32023107	0
fucI	0.2672309	-1.9038413	1.05E-07	4.83E-07	-1	0.34644249	-1.5293122	0.00123645	0.00332758	-1	1.29641629	0.37452905	0.15743197	0.59902934	0
fucK	0.6661566	-0.5860667	0.12672671	0.17244359	0	0.77126685	-0.374698	0.47759535	0.5670322	0	1.1577861	0.21136875	0.55291557	0.92069223	0
fucU	0.35294321	-1.502492	0.00293749	0.00593274	-1	0.5298548	-0.916331	0.13615384	0.20210408	0	1.50124662	0.586161	0.24978967	0.7487007	0
fucR	0.20112349	-2.3138465	2.69E-11	1.96E-10	-1	0.24974371	-2.0014797	7.44E-08	5.65E-07	-1	1.24174314	0.31236678	0.93535446	1	0
rlmM	0.83129151	-0.2665736	0.42576067	0.49803625	0	0.82348473	-0.2801862	0.58643081	0.66875813	0	0.99060886	-0.0136126	0.9391085	1	0
ygdD	2.42757484	1.27951577	0.00609462	0.01143097	1	2.11737853	1.0822792	0.03998474	0.07116091	0	0.87221967	-0.1972366	0.77928087	0.9635071	0
yagN	0.11811875	-3.0816901	3.07E-16	3.45E-15	-1	0.1974851	-2.3401843	4.48E-09	4.20E-08	-1	1.67192	0.74150582	0.3533551	0.84775072	0
gcvA	1.92233087	0.94285668	0.03231158	0.0513567	0	1.9535175	0.96607418	0.05293774	0.09028633	0	1.01622334	0.0232175	0.93123706	1	0
gcvB	0.01075609	-6.5387023	6.74E-66	1.97E-63	-1	0.07009662	-3.8345113	1.27E-25	1.51E-23	-1	6.51692316	2.70419098	5.49E-09	6.55E-07	1
ygdI	5.86564203	2.55228903	0.00026701	0.00067663	1	1.5465627	0.62906532	0.8849715	0.91307679	0	0.26366469	-1.9232237	0.00477627	0.052923	0
csdA	0.76023092	-0.3954904	0.25206428	0.31674763	0	0.90679779	-0.1411472	0.98769077	0.99853707	0	1.19279256	0.25434316	0.33667904	0.83714543	0
csdE	0.29841687	-1.744599	3.49E-05	0.00010782	-1	0.33911361	-1.5601594	0.00246951	0.00610333	-1	1.13637544	0.18443956	0.66169784	0.93664426	0
tcdA	1.14632604	0.19701744	0.63914427	0.69620228	0	1.36979887	0.45396408	0.19824588	0.27723056	0	1.194947	0.25694663	0.38173378	0.85988523	0
mltA	0.95310062	-0.0692996	0.67943478	0.73232392	0	1.25100179	0.32308386	0.44943336	0.53895414	0	1.31256004	0.39238342	0.29135582	0.79411569	0
metZ	0.44370061	-1.1723416	0.13453353	0.18205717	0	0.96259819	-0.0549944	0.59371354	0.67479942	0	2.16947683	1.11734718	0.45433928	0.88755478	0
metW	0.44118767	-1.1805356	0.23422909	0.29678424	0	1.44292251	0.52899382	0.96230723	0.97465864	0	3.27054135	1.70952946	0.27549472	0.77762465	0
metV	0.74906087	-0.4168451	0.68595411	0.73826981	0	3.01316473	1.59127955	0.32327276	0.41369481	0	4.02258996	2.00812469	0.15900052	0.60188179	0
intF	0.36985803	-1.4349565	8.62E-05	0.00024398	-1	0.41308688	-1.2754829	0.00142822	0.00378376	-1	1.11687957	0.15947363	1	1	0
amiC	1.21031182	0.27537879	0.70562814	0.75576128	0	2.49982858	1.32182917	0.07855731	0.12748613	0	2.06544176	1.04645038	0.13128257	0.54057528	0
argA	1.29579112	0.37383317	0.61692211	0.67767843	0	1.07934364	0.11015426	0.55892377	0.643199	0	0.83296114	-0.2636789	0.95790029	1	0
recD	0.72455819	-0.4648265	0.18675766	0.24362333	0	0.82900737	-0.2705432	0.34052105	0.43092873	0	1.14415567	0.19428335	0.87445714	0.98855575	0
recB	0.61962316	-0.690537	0.05201332	0.07878698	0	0.78123809	-0.3561658	0.47043579	0.55974867	0	1.26082777	0.33437122	0.32240159	0.82321404	0
ptrA	0.78650708	-0.3464683	0.27827335	0.34497148	0	0.77708485	-0.363856	0.4211822	0.51202315	0	0.98802016	-0.0173876	0.94849986	1	0
recC	0.81628405	-0.2928568	0.33867371	0.40825828	0	0.94152239	-0.0869327	0.96630451	0.97848296	0	1.15342496	0.20592415	0.45566691	0.88755478	0
ppdC	1.71756329	0.78036326	0.16805859	0.22185141	0	1.79269062	0.84212653	0.09781758	0.15371831	0	1.04374065	0.06176327	0.65127001	0.9347567	0
ygdB	0.96212278	-0.0557071	0.74416412	0.79204167	0	1.17699824	0.23511216	0.49256608	0.58059469	0	1.22333476	0.29081924	0.39070555	0.86854717	0
ppdB	3.52911349	1.81930583	0.0011153	0.00247426	1	2.76336088	1.46642398	0.00799965	0.01728012	1	0.78301842	-0.3528818	0.84856157	0.98167799	0
ppdA	5.25589882	2.3939375	0.00210869	0.00440592	1	5.09701724	2.34965323	0.0040962	0.00953694	1	0.96977081	-0.0442843	0.82536135	0.97661485	0
ptwF	1.10323684	0.14174254	0.83415127	0.86692851	0	0.63396571	-0.6575233	1	1	0	0.57464153	-0.7992658	0.98816277	1	0
thyA	0.1866918	-2.4212695	9.02E-10	5.51E-09	-1	0.36994909	-1.4346013	0.00132279	0.00354267	-1	1.9816033	0.98666818	0.02582343	0.18885794	0
lgt	0.7485904	-0.4177515	0.21292698	0.27308318	0	0.97022672	-0.0436062	0.78467838	0.83574205	0	1.29607154	0.37414536	0.19543226	0.67373807	0
ptsP	0.45070154	-1.1497557	0.00026132	0.00066374	-1	0.49924505	-1.00218	0.00763179	0.01660507	-1	1.10770655	0.14757574	0.71769648	0.95355641	0
rppH	0.25320457	-1.9816247	9.05E-10	5.52E-09	-1	0.28995294	-1.7861093	1.28E-06	7.34E-06	-1	1.14513316	0.19551536	0.90718125	0.99909148	0
mutH	1.40958256	0.49526798	0.13074965	0.17753495	0	1.35830207	0.44180435	0.19736303	0.27646017	0	0.96362009	-0.0534636	1	1	0
ygdQ	1.21765344	0.28410359	0.32364526	0.39311269	0	1.5379889	0.6210451	0.26769224	0.35512819	0	1.26307605	0.33694151	0.67139247	0.93979017	0
ygdR	0.17050124	-2.5521458	5.23E-13	4.46E-12	-1	0.19776938	-2.3381091	3.65E-09	3.49E-08	-1	1.15992923	0.21403679	1	1	0
tas	0.24809451	-2.0110383	6.11E-10	3.84E-09	-1	0.24328418	-2.0392856	5.73E-07	3.52E-06	-1	0.9806109	-0.0282473	0.94069632	1	0
lplT	2.21465576	1.14708247	0.00394072	0.00770184	1	2.22391835	1.15310382	0.01346025	0.02709833	1	1.0041824	0.00602135	1	1	0
aas	0.8947325	-0.1604717	0.80408554	0.84182172	0	0.95422371	-0.0676006	0.8794819	0.90986091	0	1.0664905	0.09287112	0.89960712	0.99683105	0
rihC	0.05619813	-4.153334	1.59E-30	5.45E-29	-1	0.0918975	-3.4438306	8.12E-16	2.11E-14	-1	1.63524123	0.70950348	0.03121877	0.21564879	0
yagP	1.33604342	0.4179669	0.54399479	0.61093949	0	0.70527929	-0.5037334	0.88995445	0.91692905	0	0.52788651	-0.9217003	0.47350465	0.89828993	0
omrA	5.19481663	2.37707282	0.17946454	0.23473346	0	16.3076442	4.02747648	0.10031878	0.15692343	0	3.1392146	1.65040366	0.54473826	0.91914725	0
omrB	5.18799717	2.37517769	0.00296563	0.00598409	1	4.58753257	2.1977184	0.05954912	0.1002467	0	0.88425888	-0.1774593	0.62963729	0.93109012	0
galR	0.4909504	-1.0263508	0.00346162	0.00685649	-1	0.41851533	-1.2566476	0.00384752	0.0090341	-1	0.85245949	-0.2302968	0.61555984	0.92897598	0
lysA	4.28844527	2.10045471	4.44E-09	2.49E-08	1	3.71107688	1.89183789	9.60E-06	4.53E-05	1	0.8653665	-0.2086168	0.60513505	0.92806272	0
lysR	1.15035692	0.20208156	0.50306838	0.57269408	0	1.60020123	0.67825334	0.10571592	0.16374295	0	1.39104759	0.47617178	0.25983082	0.75892335	0
ygeA	1.63926292	0.71304726	0.04832633	0.0737586	0	1.83691027	0.87728116	0.02913203	0.0537289	0	1.12057087	0.16423389	0.57161543	0.92806272	0
araE	1.00480461	0.00691499	0.87872113	0.90557892	0	0.66686535	-0.5845326	0.54563253	0.63035622	0	0.66367664	-0.5914476	0.44907545	0.88755478	0
kduD	2.90812732	1.54009043	6.60E-05	0.0001931	1	2.47150692	1.30539095	0.00166966	0.00433504	1	0.84986201	-0.2346995	0.79567886	0.96638496	0
kduI	2.61122777	1.3847283	0.01099938	0.01948046	1	1.71985594	0.78228773	0.09742717	0.15321375	0	0.65863881	-0.6024406	0.58082623	0.92806272	0
yqeF	1.02518834	0.03588897	0.76212834	0.80785238	0	1.08050678	0.11170812	0.63312085	0.71081327	0	1.05395929	0.07581915	0.77919851	0.9635071	0
ykgT	0	#NAME?	1	1	0	0	#NAME?	1	1	0	NA	NA	NA	NA	0
yqeG	0.83038391	-0.2681496	0.65305795	0.70873636	0	1.19016081	0.25115652	0.73106332	0.79414179	0	1.43326575	0.51930614	0.41193248	0.8712535	0
yqeH	2.36694115	1.24302383	0.02777919	0.04479763	1	2.82314158	1.49730148	0.12630743	0.18952547	0	1.19273839	0.25427765	0.92144398	1	0
yqeI	2.22747315	1.15540804	0.15610108	0.20780545	0	0.89947721	-0.1528414	0.65715101	0.7309213	0	0.40381057	-1.3082494	0.31330991	0.81404427	0
yqeJ	5.18608475	2.37464578	0.01425854	0.02464969	1	4.35255959	2.12186405	0.06690548	0.11110685	0	0.8392766	-0.2527817	0.78289377	0.9635071	0
yqeL	4.69948425	2.23250243	0.32477831	0.39421341	0	5.41113361	2.43593086	0.36793729	0.45916828	0	1.15143137	0.20342843	0.97145433	1	0
yqeK	4.64445693	2.21550991	0.12439976	0.16974783	0	5.06634832	2.34094627	0.15587175	0.22732584	0	1.09083761	0.12543635	0.95633694	1	0
ygeF	3.89757537	1.96257692	0.12176776	0.16651646	0	2.65249587	1.40735051	0.12973241	0.19394029	0	0.68055024	-0.5552264	1	1	0
ygeG	8.70574695	3.12196809	0.00053382	0.00126769	1	0.91524818	-0.1277651	0.83101049	0.87185075	0	0.10513149	-3.2497332	0.0064815	0.06789413	0
ygeH	6.91493687	2.78971608	4.64E-06	1.69E-05	1	3.9411583	1.9786197	0.00407841	0.00950053	1	0.56994856	-0.8110964	0.25287405	0.75146534	0
ygeI	3.66932179	1.87551343	0.16802017	0.22185141	0	1.385442	0.47034631	0.46014467	0.54956198	0	0.37757441	-1.4051671	0.60021646	0.92806272	0
paoD	3.1106883	1.63723384	0.00020026	0.00052371	1	4.20961986	2.07368996	1.37E-05	6.20E-05	1	1.35327601	0.43645612	0.22792523	0.71536133	0
pbl	4.99423871	2.32026478	0.05846331	0.08712295	0	0.6470828	-0.6279778	1	1	0	0.12956585	-2.9482425	0.15832269	0.60034657	0
ygeK	10.1963488	3.34998073	4.83E-09	2.70E-08	1	7.47246922	2.90158505	3.06E-05	0.00012611	1	0.73285736	-0.4483957	0.36383515	0.85329623	0
ygeN	4.97700555	2.315278	0.01582456	0.02701003	1	2.19144622	1.13188327	0.38046022	0.47173225	0	0.4403142	-1.1833947	0.26679405	0.76922645	0
ygeO	6.09091855	2.60665981	0.11372815	0.15663774	0	1.7304331	0.79113317	0.41114853	0.50189651	0	0.28410052	-1.8155266	0.50574598	0.90848871	0
ygeQ	24.2165689	4.59792257	2.01E-33	8.09E-32	1	3.80889659	1.92937312	6.00E-06	2.96E-05	1	0.15728473	-2.6685495	2.67E-10	4.91E-08	-1
glyU	1.41729408	0.50313914	0.6998045	0.75079827	0	1.97014424	0.97830126	0.38551727	0.47679843	0	1.39007442	0.47516212	0.61405434	0.92897598	0
ygeR	0.67938701	-0.5576945	0.15412585	0.20560318	0	0.92307478	-0.1154806	0.63161156	0.70929898	0	1.35868772	0.44221391	0.45281932	0.88755478	0
xdhA	0.84027692	-0.2510632	0.71514316	0.76428438	0	1.0289411	0.04116039	0.49466124	0.58244285	0	1.22452619	0.29222364	0.27799248	0.7803608	0
paoC	4.06020146	2.02155131	0.00105965	0.00236268	1	8.55162091	3.0961979	4.85E-06	2.43E-05	1	2.10620606	1.07464659	0.06332974	0.34394598	0
xdhB	1.44366148	0.52973249	0.26681637	0.33329409	0	1.81070079	0.85654817	0.04678822	0.08127246	0	1.25424195	0.32681568	0.31177211	0.81168136	0
xdhC	0.99453608	-0.0079044	0.82493914	0.85957919	0	1.40024534	0.48567963	0.41132323	0.5019711	0	1.4079382	0.49358401	0.33063901	0.83208693	0
ygeV	0.04834179	-4.3705853	2.66E-35	1.19E-33	-1	0.06802286	-3.8778365	1.77E-21	1.12E-19	-1	1.40712337	0.49274883	0.3051687	0.81005169	0
ygeW	0.3005637	-1.7342573	1.50E-06	5.93E-06	-1	0.26259412	-1.9290935	9.77E-05	0.00035312	-1	0.87367212	-0.1948361	0.97938927	1	0
ygeX	1.07443999	0.1035849	0.84987706	0.88048952	0	1.39987126	0.48529415	0.15448913	0.22560723	0	1.30288455	0.38170925	0.21648586	0.69890383	0
ygeY	1.08818722	0.12192678	0.64198023	0.69894668	0	1.00248792	0.00358486	0.7516832	0.80878697	0	0.92124582	-0.1183419	0.96705417	1	0
hyuA	3.196119	1.67632113	1.80E-05	5.83E-05	1	4.14161391	2.05019307	6.87E-06	3.35E-05	1	1.29582594	0.37387194	0.34093431	0.8405152	0
yqeA	1.59013365	0.66914803	0.10237691	0.14269448	0	1.7430139	0.80158408	0.03228663	0.05900522	0	1.09614302	0.13243605	0.46367951	0.89060394	0
yqeB	0.13321169	-2.9082074	1.16E-16	1.37E-15	-1	0.1647768	-2.601415	1.92E-09	1.92E-08	-1	1.23695446	0.30679238	0.22279767	0.70531966	0
yqeC	0.05495175	-4.1856908	5.04E-28	1.39E-26	-1	0.08495219	-3.5572051	3.15E-15	7.64E-14	-1	1.54594152	0.62848574	0.07789621	0.39259692	0
paoB	4.82283054	2.26988012	2.07E-07	9.11E-07	1	7.71880841	2.94837815	3.23E-09	3.11E-08	1	1.60047266	0.67849803	0.1159094	0.50522834	0
mocA	0.21178887	-2.2393013	3.20E-10	2.08E-09	-1	0.10373403	-3.2690389	5.77E-11	7.25E-10	-1	0.48979923	-1.0297376	0.07087216	0.36987717	0
ygfK	0.35962152	-1.4754488	6.36E-06	2.26E-05	-1	0.45154339	-1.1470635	0.01178943	0.02425396	-1	1.25560727	0.32838529	0.18757252	0.6609229	0
ssnA	1.37357976	0.45794069	0.19084079	0.24792464	0	1.70850488	0.77273437	0.07001896	0.11566871	0	1.24383376	0.31479368	0.43793871	0.88087171	0
ygfM	2.00449917	1.00324182	0.04057047	0.0632321	0	2.61886623	1.38894237	0.00478516	0.01095416	1	1.30649405	0.38570055	0.29384427	0.79843084	0
xdhD	1.79888926	0.84710638	0.02308312	0.03791674	0	1.80847289	0.85477197	0.01242679	0.02534103	0	1.00532753	0.00766559	0.62548298	0.92989404	0
xanQ	4.10612133	2.03777626	1.04E-05	3.56E-05	1	3.4856446	1.80142548	6.97E-05	0.0002612	1	0.84888982	-0.2363508	1	1	0
guaD	2.69800134	1.43189106	0.0143925	0.0248521	1	2.40919081	1.26854866	0.01513559	0.03015516	1	0.8929539	-0.1633424	0.81911893	0.97429253	0
ghxQ	2.78188782	1.47606424	0.00455893	0.00874752	1	2.54779267	1.34924788	0.00725883	0.01585603	1	0.91585025	-0.1268164	0.83772033	0.98067952	0
ygfS	2.16207036	1.11241347	0.00622575	0.00932043	0	3.37485046	1.75482358	0.02206293	0.04213314	1	1.56093461	0.64241011	0.40411999	0.86854717	0
ygfT	2.06968173	1.04940893	0.00618111	0.01157353	1	2.80795792	1.48952131	0.00066711	0.00191177	1	1.35671001	0.44011238	0.24887419	0.74826869	0
paoA	36.0654244	5.1725445	1.57E-21	2.63E-20	1	20.3789297	4.34900638	4.67E-12	6.86E-11	1	0.56505448	-0.8235381	0.06197003	0.33924908	0
uacT	3.85383104	1.94629332	0.00209546	0.00438656	1	3.95586793	1.98399426	0.00277234	0.00674612	1	1.02647674	0.03770094	0.75077867	0.95683847	0
yqfG	2.37340064	1.24695565	0.34841156	0.41852975	0	1.17366446	0.23102001	0.79525888	0.84343948	0	0.49450752	-1.0159356	0.64485454	0.93208609	0
idi	2.84248976	1.50715515	8.86E-05	0.00025035	1	0.80516872	-0.312637	0.19663376	0.27584908	0	0.28326178	-1.8197921	5.46E-06	0.00025356	-1
lysS	0.28620213	-1.8048937	2.19E-08	1.11E-07	-1	0.33558467	-1.5752513	3.05E-05	0.00012592	-1	1.17254427	0.22964239	0.61136412	0.92897598	0
prfB	0.23432454	-2.09342	6.70E-10	4.18E-09	-1	0.37801996	-1.4034657	0.00032709	0.00101647	-1	1.61323247	0.68995435	0.08005191	0.40025955	0
recJ	0.6195833	-0.6906298	0.04411274	0.06810415	0	0.67403987	-0.5690942	0.33614307	0.42635368	0	1.08789225	0.12153567	0.44696965	0.88755478	0

dsbC	0.27543762	-1.8602025	1.05E-07	4.83E-07	-1	0.30551844	-1.7106686	9.72E-05	0.00035154	-1	1.10921103	0.14953387	0.64162089	0.93164618	0
xerD	0.51192773	-0.9659879	0.00818935	0.01491644	0	0.84708374	-0.2394235	0.30743884	0.39794149	0	1.65469398	0.72656443	0.20743496	0.68832819	0
fldB	0.40665047	-1.2981388	0.01102368	0.01951568	-1	0.80832744	-0.3069883	0.27513371	0.36336397	0	1.98776958	0.99115053	0.28910955	0.79141721	0
cptA	0.23571958	-2.0848565	8.36E-09	4.52E-08	-1	0.37244407	-1.4249043	0.0006462	0.0018603	-1	1.58003025	0.65995218	0.14547666	0.57693533	0
yagU	0.17345702	-2.5273498	3.23E-10	2.10E-09	-1	0.24371267	-2.0367468	5.03E-05	0.00019583	-1	1.40503202	0.49060301	0.1468308	0.58035154	0
sdhE	0.15578512	-2.6823707	2.34E-12	1.88E-11	-1	0.21997105	-2.1846144	1.82E-07	1.27E-06	-1	1.4120158	0.49775624	0.49515008	0.90539057	0
ygfZ	0.11424522	-3.1297943	5.95E-21	9.35E-20	-1	0.11029083	-3.1806153	1.10E-14	2.38E-13	-1	0.9653868	-0.050821	1	1	0
yqfA	0.63849752	-0.6472471	0.04114775	0.06401888	0	0.44683577	-1.1621834	0.00133202	0.00356091	-1	0.69982382	-0.5149363	0.10283062	0.46993059	0
yqfB	0.13657159	-2.8722707	7.10E-11	4.90E-10	-1	0.14353505	-2.800525	1.70E-08	1.45E-07	-1	1.05098762	0.07174567	0.78365514	0.9635071	0
bglA	0.79862889	-0.3244028	0.22020745	0.28127713	0	0.32135817	-1.6377459	6.14E-06	3.02E-05	-1	0.40238737	-1.3133431	0.00017515	0.00419784	-1
ygfF	2.53081594	1.33960259	0.01944275	0.03241927	1	1.85797249	0.89372914	0.06867013	0.11356786	0	0.73413971	-0.4458734	0.70705308	0.95005571	0
gcvP	0.23505342	-2.0889394	1.25E-11	9.36E-11	-1	0.15398329	-2.6991543	8.90E-12	1.25E-10	-1	0.65509913	-0.6102149	0.12315367	0.52272154	0
gcvH	0.05586627	-4.1618787	9.22E-34	3.73E-32	-1	0.06296049	-3.9894094	6.03E-22	4.30E-20	-1	1.12698576	0.17246929	0.67810902	0.94128448	0
gcvT	0.25479989	-1.9725635	6.12E-10	3.84E-09	-1	0.28484682	-1.8117418	8.10E-07	4.86E-06	-1	1.11792365	0.16082166	0.84313854	0.98067952	0
ubiI	0.86167474	-0.2147847	0.43731898	0.50859346	0	0.58884266	-0.7640459	0.06445261	0.10759958	0	0.68336999	-0.5492612	0.18914842	0.66370038	0
ykgJ	2.63556982	1.39811491	0.05840886	0.08710059	0	1.80508649	0.85206796	0.12461585	0.18717793	0	0.6848942	-0.5460469	0.89753146	0.99640384	0
ubiH	1.00740892	0.01064941	0.93130983	0.9489263	0	0.52405424	-0.9322119	0.00907391	0.01931485	0	0.52020012	-0.9428614	0.00629846	0.0661338	0
pepP	3.52044626	1.81575832	1.59E-08	8.24E-08	1	0.52823671	-0.9207435	0.00123275	0.00332167	0	0.15004822	-2.7365019	3.46E-14	1.39E-11	-1
ygfB	0.20562025	-2.2819457	7.29E-12	5.61E-11	-1	0.18120139	-2.464334	8.19E-10	8.81E-09	-1	0.88124292	-0.1823883	0.52856703	0.91626596	0
zapA	0.21921147	-2.1896048	1.64E-12	1.34E-11	-1	0.19731234	-2.3414469	5.30E-11	6.75E-10	-1	0.90010046	-0.1518421	0.26160097	0.7605509	0
ssrS	0.52263304	-0.9361298	0.07632318	0.10970371	0	0.49813993	-1.005377	0.02248056	0.04274595	-1	0.95313518	-0.0692473	0.34801973	0.84560166	0
fau	0.48892038	-1.0323285	0.00718621	0.01325323	-1	0.79206048	-0.3363175	0.33158557	0.42117753	0	1.62001936	0.69601105	0.20904991	0.69006731	0
sibC	0.49671669	-1.0095049	0.09804112	0.1373882	0	0.45148941	-1.147236	0.11391683	0.17352585	0	0.90894753	-0.1377311	0.74132898	0.95683847	0
serA	1.32635303	0.40746483	0.26676533	0.33329409	0	1.08134654	0.11282894	0.72101439	0.78593059	0	0.81527807	-0.2946359	0.52466775	0.91309582	0
rpiA	0.15508064	-2.6889095	1.63E-15	1.75E-14	-1	0.19273965	-2.3752747	4.32E-10	4.85E-09	-1	1.24283497	0.31363474	0.67063913	0.93979017	0
ecpE	2.55986403	1.35606718	0.00206783	0.00433694	1	2.96484606	1.5679572	0.00199427	0.00507059	1	1.15820451	0.21189002	0.58757121	0.92806272	0
yqfE	2.45620217	1.29642931	0.06105578	0.09037646	0	2.70903962	1.43778149	0.03783231	0.0678219	0	1.10293837	0.14135218	0.62583901	0.92989404	0
argP	98.3383978	6.61968294	3.00E-24	6.11E-23	1	43.0636151	5.42839753	6.40E-13	1.07E-11	1	0.43791252	-1.1912854	0.01201252	0.10710485	0
scpA	3.84782304	1.94404245	2.52E-05	7.98E-05	1	5.20098652	2.3787853	2.25E-06	1.22E-05	1	1.35166988	0.43474285	0.2599008	0.75892335	0
argK	5.71069162	2.51366548	1.33E-09	7.95E-09	1	6.5607689	2.7138649	1.05E-08	9.26E-08	1	1.14885715	0.20019942	0.57973604	0.92806272	0
scpB	4.15718634	2.05560741	0.00015962	0.00042653	1	2.76024391	1.46479576	0.00843106	0.01803333	1	0.66396925	-0.5908117	0.50209508	0.90598897	0
scpC	3.32577992	1.7336927	2.30E-05	7.32E-05	1	3.38473094	1.75904115	5.77E-05	0.00022079	1	1.01772547	0.02534845	0.70142767	0.94869712	0
ygfI	1.02407651	0.03432351	0.89313537	0.91722366	0	0.82991075	-0.2689719	0.9670613	0.97902494	0	0.81039916	-0.3032954	0.85419658	0.98236299	0
yggE	0.66750642	-0.5831464	0.05718628	0.08547946	0	0.34879151	-1.5195632	0.00015106	0.00051748	-1	0.52252907	-0.9364168	0.01640659	0.13423575	0
argO	0.54408038	-0.8781083	0.018241	0.03063517	0	0.49850676	-1.004315	0.09537609	0.15047016	0	0.91623734	-0.1262067	0.81348313	0.9712234	0
mscS	1.05364962	0.07539519	0.6972993	0.74902018	0	0.60467144	-0.7257766	0.09961256	0.156095	0	0.57388285	-0.8011718	0.0490108	0.29394474	0
ecpD	1.75073956	0.80796449	0.01839472	0.03085814	0	2.72426904	1.44586919	0.00100513	0.00278299	1	1.55606756	0.6379047	0.14897502	0.58294573	0
fbaA	0.5330185	-0.9077425	0.01705609	0.02892005	0	0.40732537	-1.2957464	0.00604043	0.01346074	-1	0.76418619	-0.3880039	0.35273593	0.84775072	0
pgk	0.53286731	-0.9081518	0.00278987	0.00566307	0	0.29663173	-1.7532551	1.40E-05	6.31E-05	-1	0.55667091	-0.8451034	0.02898684	0.20546011	0
epd	0.40253071	-1.3128293	3.13E-05	9.71E-05	-1	0.38996148	-1.3585965	0.00026777	0.00085313	-1	0.96877449	-0.0457672	0.79494675	0.96579786	0
yggC	1.52073793	0.60477155	0.20348474	0.2624977	0	1.18692396	0.24722752	0.46399319	0.55379079	0	0.78049212	-0.357544	0.63144627	0.93151288	0
yggD	0.60752004	-0.7189961	0.05669646	0.08483342	0	0.45449391	-1.1376671	0.06865909	0.11356786	0	0.74811345	-0.418671	0.73141611	0.95551996	0
yggF	7.6123824	2.92834804	9.61E-06	3.31E-05	1	7.38551463	2.88469845	3.22E-05	0.0001319	1	0.97019753	-0.0436496	0.79435255	0.96579786	0
yggP	6.57577081	2.71716002	7.56E-07	3.09E-06	1	8.68324516	3.11823432	3.23E-07	2.12E-06	1	1.32049085	0.4010743	0.37141731	0.85532655	0
cmtA	8.86226058	3.14767475	1.88E-05	6.07E-05	1	9.53952101	3.25391683	3.96E-05	0.00015922	1	1.07642073	0.10624208	0.69505601	0.94809681	0
cmtB	6.79977866	2.76548779	0.09573373	0.13432525	0	4.50123492	2.17032086	0.1843072	0.26137789	0	0.66196786	-0.5951669	0.87767098	0.98956956	0
tktA	0.25536463	-1.9693694	8.91E-10	5.47E-09	-1	0.40895916	-1.2899713	0.00062232	0.00179858	-1	1.60147143	0.67939806	0.05578815	0.31745258	0
thrC	0.22388484	-2.1591712	1.00E-10	6.78E-10	-1	0.20108175	-2.314146	2.63E-08	2.15E-07	-1	0.89814811	-0.1549747	0.76953189	0.96177657	0
dapB	0.26211403	-1.9317335	1.18E-07	5.37E-07	-1	0.27319879	-1.871977	5.97E-06	2.96E-05	-1	1.04228983	0.05975651	0.8697025	0.98621446	0
ecpC	5.44799118	2.44572437	0.00013955	0.00037745	1	7.70963265	2.94666212	3.13E-05	0.00012868	1	1.4151331	0.50093775	0.35113745	0.84775072	0
loiP	0.26212362	-1.9316808	1.70E-08	8.79E-08	-1	0.20186707	-2.3085225	4.98E-09	4.65E-08	-1	0.77012166	-0.3768417	0.17454905	0.63250725	0
speB	0.49572415	-1.0123905	0.00210174	0.00439762	-1	0.29023214	-1.7847208	1.81E-05	7.87E-05	-1	0.58547106	-0.7723302	0.05721126	0.32074131	0
speA	0.73523695	-0.4437188	0.15050491	0.20132652	0	0.50855495	-0.9755244	0.00779364	0.01690726	0	0.69168851	-0.5318056	0.12038168	0.51816545	0
yqgB	0.56504832	-0.8235538	0.23282647	0.29551567	0	1.51725778	0.60146621	0.85743851	0.89264238	0	2.68518235	1.42502006	0.23561313	0.72813869	0
yqgC	1.41979149	0.50567907	0.67910973	0.73232392	0	2.70545186	1.43586957	0.26558747	0.35265355	0	1.9055276	0.9301905	0.43350046	0.88049119	0
metK	0.52599767	-0.9268717	0.00635598	0.01185572	0	0.40421416	-1.3068082	0.00135061	0.0036019	-1	0.7684714	-0.3799365	0.29459834	0.79886161	0
galP	3.27314032	1.71067545	2.36E-06	9.05E-06	1	3.46106481	1.79121596	4.07E-05	0.00016264	1	1.05741412	0.0805405	0.88954272	0.99411016	0
yggI	4.40294077	2.13846744	5.61E-06	2.01E-05	1	5.21959339	2.38393743	4.14E-05	0.00016503	1	1.1854789	0.24546999	0.74891109	0.95683847	0
endA	1.39476591	0.48002301	0.25313009	0.31772538	0	2.64918623	1.40554926	0.01992278	0.03847847	1	1.89937696	0.92552626	0.13705098	0.55704593	0
rsmE	0.42266333	-1.2424192	0.00089978	0.00204021	-1	0.62675537	-0.6740256	0.12323455	0.18554462	0	1.48287143	0.56839352	0.18960911	0.66370038	0
ecpB	6.74212673	2.75320375	0.00015416	0.00041444	1	6.62268182	2.72741555	0.00067681	0.00193706	1	0.98228379	-0.0257882	1	1	0
gshB	0.33239365	-1.5890353	1.44E-06	5.68E-06	-1	0.25266599	-1.9846966	2.69E-06	1.44E-05	-1	0.76014086	-0.3956613	0.40427083	0.86854717	0
yqgE	0.42173013	-1.245608	0.00031506	0.00078357	-1	0.6778008	-0.5610668	0.10295767	0.16048323	0	1.60719083	0.68454124	0.16666539	0.61557317	0
yqgF	0.62399267	-0.680399	0.10455366	0.14536151	0	0.75634378	-0.402886	0.42601986	0.51662507	0	1.21210363	0.27751305	0.58849155	0.92806272	0
yggR	4.19514598	2.06872101	1.25E-06	4.97E-06	1	5.86352527	2.5517683	8.37E-08	6.27E-07	1	1.39769279	0.48304729	0.20371602	0.68295505	0
yggS	0.43448277	-1.2026291	0.00448866	0.00863896	-1	0.67817167	-0.5602776	0.17813519	0.25442128	0	1.56087126	0.64235155	0.26849989	0.76966758	0
yggT	0.4726588	-1.081129	0.00458535	0.00879439	-1	0.55751858	-0.8429082	0.11053098	0.16947828	0	1.17953709	0.23822078	0.45247486	0.88755478	0
yggU	0.90259296	-0.1478526	0.69585426	0.7478317	0	0.73726359	-0.4397476	0.64476669	0.73827696	0	0.81682843	-0.291895	0.85108991	0.98212032	0
rdgB	0.94318349	-0.0843896	0.79591492	0.83504898	0	0.94555643	-0.0807645	0.79571076	0.84362905	0	1.00251588	0.00362508	0.9514748	1	0
yggW	0.90027437	-0.1515634	0.74881084	0.79621991	0	1.05111739	0.0719238	0.76766332	0.82158009	0	1.16755228	0.22348715	0.54978517	0.91965346	0
yggM	1.56540997	0.64654054	0.16725462	0.22105436	0	1.48298536	0.56850436	0.32679499	0.41689003	0	0.94734631	-0.0780362	0.88548416	0.99348376	0
ecpA	1.15767138	0.21122579	0.62981509	0.68875793	0	1.27712949	0.35290481	0.29638612	0.38635111	0	1.10318827	0.14167902	0.52336533	0.91269012	0
ansB	0.00438631	-7.832776	3.39E-82	5.74E-79	-1	0.00768084	-7.0245194	5.29E-48	1.17E-45	-1	1.75109419	0.80825669	0.02078098	0.16049758	0
yggN	1.21701274	0.28334427	0.32151129	0.39100093	0	1.29326025	0.37101263	0.51896381	0.60621228	0	1.06265137	0.08766836	0.94850014	1	0
yggL	0.29460033	-1.763169	5.41E-08	2.62E-07	-1	0.23105201	-2.1137105	2.37E-07	1.61E-06	-1	0.78428971	-0.3505414	0.33170708	0.83208693	0
trmI	0.77693466	-0.3641348	0.25705468	0.32210227	0	0.67377527	-0.5696606	0.16310795	0.23639687	0	0.86722257	-0.2055258	0.59959873	0.92806272	0
mutY	0.72405065	-0.4658375	0.23788139	0.30098027	0	1.18861046	0.24927598	0.53963232	0.6252321	0	1.64161231	0.71511346	0.09366442	0.44558801	0
yggX	0.1499533	-2.7374148	2.68E-12	2.14E-11	-1	0.36394507	-1.4582074	0.00025471	0.0008186	-1	2.42705606	1.27920743	0.01654638	0.13512873	0
mltC	1.99462311	0.99611617	0.00764758	0.01403963	0	2.37537887	1.24815764	0.0041712	0.00969622	1	1.19089108	0.25204147	0.52229801	0.91269012	0
nupG	0.08188429	-3.6102695	1.92E-27	5.07E-26	-1	0.07514954	-3.7340918	6.71E-21	3.80E-19	-1	0.91775288	-0.1238224	0.48957739	0.90336299	0
speC	2.27953806	1.1887415	0.00122016	0.00268933	1	2.87152701	1.52181813	0.000343	0.00106068	1	1.25969689	0.33307663	0.36701857	0.85342842	0
yqgA	0.38689029	-1.3700036	0.000253	0.00064445	-1	0.41575967	-1.2661783	0.0060889	0.01355508	-1	1.07461904	0.10382531	0.79432838	0.96579786	0
ecpR	4.70264503	2.23347244	0.04847726	0.07393786	0	4.09778438	2.03484408	0.09370286	0.14820076	0	0.87137863	-0.1986284	1	1	0
pheV	1.13795054	0.18643786	0.97800167	0.98513874	0	0.59160493	-0.757294	0.89703493	0.92143128	0	0.51988633	-0.9437319	0.80150041	0.96905452	0
yghD	6.63288525	2.72963657	1.36E-08	7.14E-08	1	7.98979971	2.99815934	1.56E-07	1.11E-06	1	1.20457379	0.26852277	0.66314147	0.93720148	0
yghE	0.93589644	-0.0955792	0.58530347	0.64891565	0	1.28049905	0.35670618	0.45697318	0.54636485	0	1.36820592	0.45228538	0.25259385	0.75113883	0
yghF	3.77615527	1.91691809	0.00876296	0.01585662	1	5.56394758	2.47610883	0.0020827	0.00525022	1	1.47344248	0.55919074	0.37890815	0.85988523	0
yghG	4.05803941	2.02078288	0.01297463	0.02265176	1	5.0438706	2.33453126	0.01280203	0.02601752	1	1.24293288	0.31374839	0.65927343	0.93664426	0
pppA	4.98212026	2.31675985	1.94E-06	7.52E-06	1	4.84763068	2.27727979	4.05E-05	0.00016205	1	0.97300555	-0.0394801	1	1	0
sslE	2.47267608	1.30607326	0.00019393	0.00050807	1	2.71851593	1.44281928	5.39E-05	0.0002079	1	1.09942259	0.13674602	0.42762661	0.87784805	0
glcA	8.60746134	3.1055878	9.57E-09	5.12E-08	1	3.93843173	1.97762127	0.00032515	0.00101184	1	0.4575602	-1.1279665	0.11073827	0.49378744	0
glcB	2.46305803	1.30045062	0.0093292	0.01678762	1	0.30659845	-1.7055777	0.00282159	0.00684713	-1	0.12447878	-3.0060283	4.20E-07	2.99E-05	-1
glcG	2.21298867	1.14599607	0.00136059	0.00296191	1	0.31340349	-1.6739068	3.05E-05	0.00012608	-1	0.14162002	-2.8199029	1.35E-11	3.21E-09	-1
ykgL	0.85225813	-0.2306376	0.82595086	0.86002474	0	1.45083432	0.53688278	0.68773309	0.75806747	0	1.70234143	0.76752042	0.55471433	0.92071004	0
glcF	6.93491594	2.7938784	1.90E-06	7.36E-06	1	2.31753662	1.21259214	0.22489848	0.30896307	0	0.33418381	-1.5812863	0.00549731	0.05978403	0
glcE	10.8400829	3.43830388	3.78E-10	2.44E-09	1	1.05450968	0.07657234	0.8190212	0.86152218	0	0.09727875	-3.3617315	7.02E-07	4.62E-05	-1
glcD	8.8174379	3.14035951	8.85E-07	3.59E-06	1	1.27232489	0.34746712	0.67610414	0.7484229	0	0.14429644	-2.7928924	0.000195	0.00445561	-1
glcC	1.74727616	0.80510765	0.05846319	0.08712295	0	1.86370454	0.89817317	0.10220005	0.15952739	0	1.06663422	0.09306552	0.96688548	1	0
yghO	2.64879095	1.40533398	0.04266075	0.06609349	0	3.37187264	1.75355004	0.01281153	0.02601752	1	1.27298556	0.34821606	0.45605154	0.88755478	0
yghQ	4.3168689	2.10998528	0.00019277	0.00050532	1	3.62106185	1.85641282	0.00174875	0.00450597	1	0.83881673	-0.2535725	0.93387488	1	0
yghR	6.01738332	2.58913626	0.00079701	0.00182783	1	7.37542075	2.88272535	0.00097869	0.0027166	1	1.22568571	0.29358909	0.64012779	0.93163024	0
yghS	6.11726017	2.61288564	8.93E-06	3.09E-05	1	5.15280555	2.36535815	6.26E-05	0.00023682	1	0.84233879	-0.2475275	1	1	0

yghT	6.41932379	2.68242133	0.00112578	0.00249501	1	7.80482655	2.96436657	0.00204421	0.00517376	1	1.21583313	0.28194524	0.75391901	0.9573569	0
ykgO	0.44492631	-1.1683617	0.41308737	0.48630248	0	0.36732251	-1.4448808	0.7426143	0.80156634	0	0.82558056	-0.2765191	0.80823309	0.96990832	0
pitB	7.61775065	2.92936507	7.49E-06	2.63E-05	1	9.50377684	3.24850096	6.36E-06	3.12E-05	1	1.24758308	0.31913589	0.50680836	0.90925684	0
gss	0.75775561	-0.4001955	0.22526355	0.28698849	0	0.65952573	-0.6004991	0.26316335	0.35020595	0	0.87036733	-0.2003037	0.89175704	0.99488354	0
yghU	0.34332011	-1.5423737	3.49E-06	1.30E-05	-1	0.22660003	-2.14178	3.34E-07	2.18E-06	-1	0.66002551	-0.5994063	0.14130009	0.56803408	0
hybG	0.26967473	-1.8907077	0.00310601	0.00622754	-1	0.3275815	-1.6100742	0.04795271	0.08290415	0	1.2147282	0.28063354	0.54720028	0.91914725	0
hybF	0.27876591	-1.8428739	0.01213372	0.02132457	-1	0.70855424	-0.4970498	0.56440599	0.64832599	0	2.5417535	1.34582412	0.08428324	0.41436912	0
hybE	0.13230549	-2.9180552	6.69E-09	3.66E-08	-1	0.25581615	-1.9668208	0.00082469	0.00231236	-1	1.93352629	0.95123438	0.06348291	0.3443538	0
hybD	0.30628684	-1.7070447	2.03E-05	6.53E-05	-1	0.42485289	-1.2349647	0.00814826	0.01752173	-1	1.38710788	0.47207999	0.33466623	0.83443729	0
hybC	0.13851695	-2.8518656	7.22E-15	7.35E-14	-1	0.23323094	-2.1001689	2.94E-06	1.55E-05	-1	1.68377185	0.75169667	0.02264109	0.17126454	0
hybB	0.22293036	-2.165335	1.74E-08	8.97E-08	-1	0.35091409	-1.5108102	0.00118608	0.00322027	-1	1.57409735	0.65452477	0.06238606	0.34092013	0
hybA	0.08127577	-3.6210309	1.06E-22	1.90E-21	-1	0.13232377	-2.9178558	1.18E-11	1.62E-10	-1	1.62808399	0.70317512	0.05723686	0.32074131	0
ykgM	0.53890956	-0.8918849	0.3587848	0.42935585	0	1.65783851	0.72930348	0.77902559	0.83092303	0	3.07628337	1.6211884	0.2710166	0.77208217	0
hybO	0.03516909	-4.8295482	1.97E-37	1.00E-35	-1	0.04958502	-4.3339519	5.84E-23	5.03E-21	-1	1.40990337	0.49559629	0.19593833	0.67373807	0
yghW	6.29532684	2.65428128	0.02578691	0.04193952	1	2.57557776	1.3648961	0.20167849	0.28114109	0	0.40912534	-1.2893852	0.46144877	0.88879788	0
yghX	4.38424169	2.13232733	8.50E-05	0.00024154	1	2.71457218	1.44072484	0.00403769	0.00941559	1	0.61916572	-0.6916025	0.46962205	0.89538835	0
gpr	0.17222604	-2.5376248	1.29E-14	1.28E-13	-1	0.09315274	-3.424258	3.31E-16	9.07E-15	-1	0.54087488	-0.8866332	0.02527319	0.18575793	0
yqhA	0.32085179	-1.6400211	4.74E-06	1.73E-05	-1	0.52568297	-0.9277351	0.01916265	0.03718918	0	1.63839813	0.71228597	0.12948355	0.53863917	0
yghA	5.76488634	2.52729217	4.11E-05	0.00012527	1	1.51942811	0.60352842	0.30404666	0.39413481	0	0.26356601	-1.9237637	0.01193508	0.10697905	0
exbD	0.35149425	-1.508427	0.00306619	0.00616167	-1	0.44839329	-1.1571634	0.04190337	0.07409804	0	1.27567745	0.35126359	0.68285889	0.94348666	0
exbB	0.37798102	-1.4036143	0.0012631	0.00277293	-1	0.56649958	-0.8198532	0.06541824	0.10896465	0	1.49875138	0.58376108	0.3770446	0.85988523	0
metC	2.12762807	1.08924597	0.19475562	0.25215448	0	3.2813914	1.71430769	0.19054357	0.26901343	0	1.5422768	0.62506171	0.76318511	0.9588736	0
yghB	2.48032899	1.31053149	0.05354021	0.08074365	0	2.51957812	1.33318219	0.20340995	0.28301907	0	1.01582416	0.0226507	0.74321824	0.95683847	0
ykgR	0.8204081	-0.2855864	0.80361233	0.84152577	0	0.37118114	-1.4298047	0.14853734	0.2180686	0	0.45243475	-1.1442183	0.20894439	0.69006731	0
yqhC	0.84395435	-0.2447631	0.36678342	0.4371512	0	0.64251769	-0.6381919	0.42132688	0.51205816	0	0.76131806	-0.3934288	0.82656494	0.97699045	0
yqhD	0.19937574	-2.3264382	3.90E-11	2.80E-10	-1	0.23039018	-2.1178489	1.53E-06	8.67E-06	-1	1.15555776	0.20858938	0.35952096	0.85183882	0
dkgA	0.76721242	-0.382302	0.19478177	0.25215448	0	0.29077314	-1.7820341	1.92E-05	8.28E-05	-1	0.37899952	-1.3997321	0.00057312	0.01089421	-1
yqhG	3.78483055	1.92022871	5.14E-05	0.00015458	1	3.96005985	1.98552223	0.0007737	0.00218606	1	1.04629779	0.06529352	1	1	0
yqhH	5.510479	2.46217773	7.53E-05	0.00021715	1	4.29912198	2.10404205	0.00767243	0.01666887	1	0.7801721	-0.3581357	0.50215259	0.90598897	0
ygiQ	1.08323699	0.11534891	0.69824587	0.74950172	0	1.39770082	0.48305558	0.13958816	0.2066466	0	1.29030012	0.36770667	0.23958904	0.73317437	0
ftsP	0.6391321	-0.6458139	0.05918609	0.08799225	0	0.75881248	-0.3981847	0.23328554	0.31797928	0	1.18725453	0.24762926	0.66800747	0.93878679	0
plsC	0.44246531	-1.1763637	0.00083367	0.00190201	-1	0.43072311	-1.2151674	0.00480456	0.01097795	-1	0.97346188	-0.0388036	0.86830211	0.98621446	0
parC	0.77631696	-0.3652823	0.31442225	0.38301243	0	0.90183236	-0.1490688	0.64784366	0.72421481	0	1.16168061	0.21621347	0.66077295	0.93664426	0
ygiS	0.24505572	-2.0288183	2.05E-08	1.04E-07	-1	0.41914506	-1.2544785	0.00354645	0.00839859	-1	1.71040713	0.77433977	0.04405853	0.27212623	0
ykgP	2.68115837	1.42285644	0.44052332	0.51110888	0	7.40482146	2.88846495	0.15129413	0.22174752	0	2.76179936	1.46560851	0.42391769	0.87522333	0
mqsA	0.36325323	-1.4609525	9.31E-05	0.00026144	-1	0.16326931	-2.6146745	1.29E-06	7.38E-06	-1	0.44946417	-1.153722	0.03419048	0.23019849	0
mqsR	0.27130372	-1.8820193	1.16E-05	3.93E-05	-1	0.22707348	-2.1387689	7.29E-05	0.00027132	-1	0.83697147	-0.2567496	0.55421086	0.92071004	0
ygiV	2.07300124	1.05172098	0.03106324	0.0495151	1	1.51591432	0.60018821	0.1714056	0.24664322	0	0.73126551	-0.4515328	0.5788742	0.92806272	0
ygiW	1.4834276	0.56893451	0.47480713	0.54459181	0	0.68553627	-0.5446951	0.30405195	0.39413481	0	0.46212992	-1.1136296	0.08814081	0.42620721	0
qseB	1.32116602	0.40181177	0.40865161	0.48197975	0	1.560777	0.64226442	0.18068354	0.2568985	0	1.18136326	0.24045265	0.53006544	0.91706103	0
qseC	1.84594725	0.88436133	0.02615736	0.04246125	0	2.03028169	1.02167991	0.01147171	0.02373292	1	1.099859	0.13731858	0.52267258	0.91269012	0
ygiZ	3.72088139	1.8956444	0.04068174	0.06338315	0	3.25292469	1.70173742	0.09614423	0.15146572	0	0.87423499	-0.193907	0.94110627	1	0
mdaB	0.57858776	-0.7893923	0.08795495	0.12435884	0	1.0844588	0.11697524	0.72908555	0.79253185	0	1.87432032	0.90636753	0.28717653	0.78998378	0
ygiN	0.12013051	-3.0573254	5.79E-20	8.49E-19	-1	0.11148777	-3.1650426	8.91E-16	2.29E-14	-1	0.92805537	-0.1077172	0.40788866	0.86959974	0
parE	0.35034531	-1.5131505	6.29E-06	2.24E-05	-1	0.70314288	-0.5081102	0.21579268	0.29800347	0	2.00699955	1.00504029	0.00579207	0.06199763	0
eaeH	8.83522605	3.14326705	2.25E-05	7.17E-05	1	8.98086499	3.1668544	5.36E-05	0.00020718	1	1.0164839	0.02358736	0.76424077	0.95937995	0
yqiA	0.35892365	-1.4782511	0.00017355	0.00046041	-1	0.96716078	-0.0481723	0.54537825	0.63035622	0	2.69461427	1.43007877	0.00552583	0.05978403	0
cpdA	0.21575853	-2.2125105	0.01337416	0.02331242	-1	1.37518722	0.45962804	0.94892343	0.96441873	0	6.37373294	2.67213857	0.02307742	0.17358733	0
yqiB	0.25561678	-1.9679456	0.0310542	0.0495151	-1	1.22803072	0.29634665	0.89362347	0.91878132	0	4.8041867	2.26429222	0.07652527	0.38702701	0
nudF	1.11127582	0.15221694	0.93977191	0.95512483	0	2.02584255	1.01852205	0.23100383	0.31574573	0	1.82298806	0.86630511	0.24502742	0.74368268	0
tolC	0.15989009	-2.6448476	2.00E-16	2.29E-15	-1	0.13497207	-2.8892672	2.28E-13	4.10E-12	-1	0.84415532	-0.2444196	0.45518714	0.88755478	0
ygiB	0.29443742	-1.7639671	7.17E-08	3.37E-07	-1	0.25360528	-1.9793433	1.03E-06	6.04E-06	-1	0.86132151	-0.2153762	0.63659762	0.93163024	0
ygiC	0.33213915	-1.5901403	3.04E-07	1.31E-06	-1	0.2865165	-1.8033099	1.30E-06	7.42E-06	-1	0.86263994	-0.2131696	0.43985515	0.88087171	0
ygiD	1.19095219	0.2521155	0.57739536	0.6419218	0	1.01982093	0.02831585	0.73879078	0.79881412	0	0.8563072	-0.2237996	0.85587451	0.98266248	0
zupT	0.41886923	-1.2554282	0.00032933	0.00081675	-1	0.42299113	-1.2413007	0.00419235	0.00974028	-1	1.00984054	0.0141275	0.91357575	1	0
ribB	0.10350645	-3.2722075	2.33E-22	4.07E-21	-1	0.1354798	-2.8838504	1.70E-12	2.66E-11	-1	1.30890203	0.38835712	0.23638753	0.72899932	0
carA	3.08095874	1.62337936	7.44E-05	0.00021484	1	2.87880128	1.52546821	0.00076557	0.00216586	1	0.93438489	-0.0979112	0.98569808	1	0
yqiC	0.4117215	-1.2802593	0.00074374	0.00171994	-1	0.42149704	-1.2464056	0.00343494	0.00817393	-1	1.02374308	0.0338537	0.81392826	0.9712234	0
ygiL	4.29634356	2.10310937	0.01434853	0.02478586	1	3.94141999	1.97871549	0.06493351	0.10827969	0	0.91738939	-0.1243939	0.86176579	0.98424873	0
yqiG	5.47704447	2.45339759	0.00033046	0.00081911	1	3.87737972	1.95508203	0.00174874	0.00450597	1	0.70793285	-0.4983156	0.85567811	0.98266248	0
yqiH	2.76238682	1.46591535	0.05762294	0.0860448	0	3.77958544	1.918228	0.02880066	0.05318434	0	1.36823178	0.45261265	0.56155811	0.92432422	0
yqiI	4.47432307	2.16166943	1.94E-06	7.50E-06	1	3.88115798	1.95648716	0.00016184	0.00054928	1	0.86742909	-0.2051823	0.72946551	0.95488877	0
glgS	1.63048692	0.70530287	0.10490324	0.14580164	0	0.65641189	-0.6073267	0.01881299	0.03657484	0	0.40258642	-1.3126296	0.00018647	0.00432801	-1
yqiJ	0.44730425	-1.1606716	0.08409141	0.11966659	0	0.72994951	-0.4541314	0.64409885	0.72112308	0	1.63188592	0.7065402	0.26845292	0.76966758	0
yqiK	2.22478089	1.15366325	0.00084529	0.00192455	1	2.12478671	1.08731803	0.00425653	0.00986344	1	0.95505437	-0.0663452	1	1	0
sibD	1.11623489	0.15864065	0.50738372	0.57671485	0	1.60647225	0.68389606	0.68874845	0.758619	0	1.43918834	0.52525541	1	1	0
sibE	1.00400782	0.0057705	0.92818688	0.94618081	0	2.44735655	1.2912243	0.38321373	0.47434775	0	2.43758718	1.28545379	0.3767397	0.85988523	0
hldE	0.64494239	-0.6327578	0.0718409	0.10400566	0	0.87674851	-0.189765	0.53407585	0.62058406	0	1.35942144	0.44299278	0.34115133	0.8405152	0
glnE	0.88691027	-0.1731399	0.55687757	0.6228737	0	0.75141613	-0.412316	0.44748362	0.53719916	0	0.84722903	-0.2391761	0.74263265	0.95683847	0
ygiF	3.00209872	1.58597142	1.79E-06	6.98E-06	1	1.29581042	0.37385466	0.65178557	0.72678285	0	0.43163485	-1.2121168	0.00047302	0.00914926	-1
ygiM	0.23662843	-2.0793047	8.67E-09	4.67E-08	-1	0.54782831	-0.8682043	0.03920225	0.06993732	0	2.31514156	1.21110041	0.00281218	0.03633558	1
cca	0.6481384	-0.6256262	0.07674003	0.11019529	0	0.68842736	-0.5386237	0.18200834	0.25836648	0	1.06216104	0.08700251	0.86638481	0.98621446	0
bacA	3.1343946	1.64818682	2.47E-06	9.44E-06	1	1.62860998	0.70364115	0.05537576	0.09372098	0	0.51959316	-0.9445457	0.03189643	0.21842123	0
ykgA	2.05146351	1.03665349	0.02155305	0.03562868	1	1.65576702	0.72749969	0.1744244	0.24979853	0	0.80711503	-0.3091538	0.57598724	0.92806272	0
folB	1.12984203	0.17612107	0.43906093	0.50994918	0	1.80355378	0.85084244	0.20028919	0.27961071	0	1.59628845	0.67472137	0.41513132	0.87131399	0
plsY	0.89562342	-0.1590358	0.52166138	0.58945467	0	1.34195904	0.42434064	0.35279635	0.44365596	0	1.49835189	0.58337648	0.15125148	0.58738081	0
ttdR	0.20187866	-2.3084397	3.63E-08	1.80E-07	-1	0.19532209	-2.356073	1.50E-05	6.71E-05	-1	0.96752223	-0.0476333	0.67280891	0.93997784	0
ttdA	5.76307236	2.52683813	0.00244009	0.00501439	1	8.92221306	3.1574016	0.00135823	0.00362002	1	1.54816954	0.63056347	0.46909439	0.89538835	0
ttdB	7.59940257	2.925886	5.41E-05	0.00016159	1	10.6632693	3.41457793	5.00E-05	0.00019508	1	1.40317206	0.48869192	0.49806685	0.90539057	0
ttdT	5.13545733	2.36049276	8.75E-10	5.38E-09	1	7.4997239	2.90683748	9.18E-12	1.28E-10	1	1.46038092	0.54634473	0.10801676	0.48409953	0
tsaD	0.99109196	-0.0129092	0.62321082	0.68356647	0	1.76740049	0.82162899	0.03176553	0.05822144	0	1.78328607	0.83453816	0.0504399	0.29777774	0
rpsU	0.18079153	-2.467601	0.00372975	0.00733168	-1	1.03081847	0.0437903	0.70790145	0.77431102	0	5.70169684	2.51139133	0.02846821	0.20284423	0
dnaG	0.67071634	-0.5762253	0.06530817	0.09580411	0	1.03486003	0.04943565	0.86550538	0.89780945	0	1.54291758	0.62566099	0.16471303	0.61352961	0
rpoD	0.49085725	-1.0266246	0.00033131	0.00082029	-1	0.39816022	-1.328579	0.0002828	0.00089597	-1	0.81115279	-0.3019544	0.39735104	0.86854717	0
ykgQ	3.05064137	1.60911259	0.2512903	0.31595481	0	4.00515508	2.00185811	0.22695379	0.31117245	0	1.31288952	0.39274552	0.77965377	0.9635071	0
mug	0.74380424	-0.4270051	0.18736328	0.24426907	0	0.22750838	-2.1360084	6.47E-07	3.95E-06	-1	0.30587132	-1.7090033	3.82E-05	0.00123071	-1
ileX	2.44179574	1.28794252	0.31504618	0.38366666	0	2.19736665	1.13577562	0.4804766	0.56983699	0	0.89989781	-0.1521669	0.94494641	1	0
yqjH	0.72869937	-0.4566044	0.2425159	0.30614253	0	0.88062924	-0.1833933	0.85431156	0.88995823	0	1.20849459	0.27321101	0.42636443	0.87780913	0
yqjI	0.15951237	-2.6482598	7.84E-11	5.39E-10	-1	0.19914895	-2.3280802	3.27E-06	1.70E-05	-1	1.24848598	0.32017962	0.35214336	0.84775072	0
aer	1.83276086	0.87401855	0.00905597	0.01634664	0	2.22929784	1.15658938	0.00151895	0.00398827	1	1.21636046	0.28257083	0.317969	0.81858662	0
patA	19.6699746	4.29792319	6.98E-06	2.46E-05	1	1.83947911	0.8792973	0.31639075	0.40631524	0	0.09351711	-3.4186259	0.00371595	0.04413297	-1
ygjH	0.88098203	-0.1828155	0.88002033	0.90649482	0	0.66460882	-0.5894226	0.38257016	0.47381657	0	0.75439544	-0.4066071	0.48144745	0.89935549	0
ebgR	0.35677157	-1.4869274	7.18E-05	0.00020818	-1	0.37080686	-1.4312602	0.0023908	0.00594752	-1	1.03933969	0.05566725	0.74950857	0.95683847	0
ebgA	1.96636592	0.97553182	0.02157442	0.03565066	0	2.36370359	1.24104913	0.00816079	0.01754014	1	1.20206701	0.26551732	0.44875291	0.88755478	0
ebgC	0.58957003	-0.7622649	0.38015578	0.45150526	0	0.56672006	-0.8192918	0.45241768	0.54194463	0	0.961243	-0.0570269	1	1	0
rclC	6.00999568	2.58736395	0.00182653	0.00386943	1	4.67940541	2.22632523	0.00563742	0.01266485	1	0.77860379	-0.3610387	0.95428965	1	0
ygjI	3.97659927	1.99153519	0.00151701	0.00327179	1	4.17374516	2.06134252	0.00205068	0.00518494	1	1.0495765	0.06980733	0.73359099	0.95599369	0
ygjJ	7.78354112	2.96042666	1.80E-05	5.83E-05	1	8.53950274	3.09415206	2.27E-05	9.65E-05	1	1.0971231	0.13372541	0.62723255	0.93095788	0
ygjK	4.21266809	2.07473425	0.00040174	0.00097659	1	4.63640169	2.21300556	0.00011044	0.00039231	1	1.10058556	0.13827131	0.47768937	0.89855925	0
fadH	3.37099249	1.75317341	7.35E-08	3.45E-07	1	2.87755593	1.52484397	0.00043104	0.00129137	1	0.85362276	-0.2283294	0.32504476	0.82473426	0
higA	1.25129684	0.32342407	0.33749256	0.40729634	0	0.78569608	-0.3479567	0.80407808	0.84984957	0	0.62790543	-0.6713808	0.30968021	0.81146151	0
higB	1.06691665	0.09344747	0.95387829	0.96800644	0	1.03692501	0.05231156	0.94545902	0.96266899	0	0.97188943	-0.0411359	0.9687467	1	0

rlmG	1.61421993	0.69083715	0.03608878	0.05675007	0	3.26189869	1.70571198	0.00065029	0.00186965	1	2.02072756	1.01487483	0.05943988	0.3301384	0
ygjP	1.81284363	0.85825449	0.04606893	0.07079547	0	2.01279233	1.00919833	0.03389156	0.06158174	0	1.11029561	0.15094384	0.68591146	0.94379705	0
ygjQ	11.5313751	3.52749266	0.00073806	0.0017095	1	22.8727052	4.5155551	0.00047204	0.00139528	1	1.98351931	0.98806245	0.50425506	0.90701715	0
ygjR	0.10128276	-3.3035394	1.74E-22	3.07E-21	-1	0.10402436	-3.2650067	6.52E-15	1.48E-13	-1	1.02706871	0.0385327	0.7103616	0.95218683	0
rclB	2.9168697	1.54442094	0.49714834	0.56653876	0	0.39912988	-1.3250698	0.89966905	0.92349304	0	0.136835	-2.8694908	0.41565428	0.87131399	0
alx	4.30149362	2.1048377	2.52E-06	9.58E-06	1	5.06372343	2.34019861	1.72E-06	9.62E-06	1	1.1772012	0.23536091	0.4428318	0.88245745	0
sstT	2.3776536	1.24953854	0.00014999	0.00040471	1	2.47155251	1.30541756	0.00119938	0.00324959	1	1.03949226	0.05587901	0.90884729	0.99971546	0
ygjV	2.39624948	1.26077812	0.01256933	0.02197891	1	2.16586033	1.11494021	0.0256009	0.04791629	1	0.90385427	-0.1458379	1	1	0
uxaA	0.17204376	-2.5391525	1.15E-12	9.63E-12	-1	0.174428	-2.5192965	2.99E-09	2.91E-08	-1	1.01385832	0.01985606	1	1	0
uxaC	0.1363041	-2.8750992	2.68E-17	3.33E-16	-1	0.12288071	-3.0246696	4.51E-13	7.69E-12	-1	0.90151883	-0.1495705	0.77312793	0.9628697	0
exuT	0.56628499	-0.8203998	0.01801688	0.03028942	0	0.70977518	-0.494566	0.36414485	0.45598076	0	1.25338867	0.32583385	0.26837598	0.76966758	0
exuR	0.36813091	-1.4417092	4.94E-06	1.79E-05	-1	0.34473949	-1.5364215	3.33E-05	0.00013601	-1	0.93645895	-0.0947123	0.58187297	0.92806272	0
yqjA	0.66477599	-0.5890598	0.08471688	0.12039719	0	0.67325074	-0.5707842	0.17386152	0.24936516	0	1.01274827	0.01827562	0.98480693	1	0
mzrA	0.88369986	-0.1783716	0.65931612	0.71500106	0	0.65240928	-0.6161508	0.548707	0.63292515	0	0.73827021	-0.4377792	0.76274142	0.95858924	0
yqjC	1.12300673	0.16736657	0.84094558	0.87275767	0	0.38989838	-1.3588299	0.01959472	0.03794438	-1	0.34719149	-1.5261965	0.00937144	0.08887753	0
rclA	2.10517952	1.07394326	0.00639443	0.01191236	1	1.63111072	0.70585472	0.03368375	0.06122932	0	0.77480838	-0.3680885	0.80790381	0.96990832	0
yqjD	1.67419859	0.74347067	0.27912159	0.34582891	0	0.34380948	-1.5403188	0.07221816	0.1187689	0	0.20535765	-2.2837894	0.00718942	0.07309818	0
yqjE	2.25168177	1.17100294	0.09348838	0.13134167	0	0.49526324	-1.0137325	0.21725142	0.29966213	0	0.21995259	-2.1847355	0.00917731	0.08760157	0
yqjK	1.91125252	0.9345184	0.14535978	0.19509393	0	0.41530414	-1.2677599	0.08942029	0.14213966	0	0.21729423	-2.2022783	0.00414925	0.04777601	-1
yqjF	5.32491295	2.41275794	2.01E-07	8.88E-07	1	2.6703877	1.41704922	0.00217462	0.00546624	1	0.50148946	-0.9957087	0.12967741	0.53863917	0
yqjG	40.4684068	5.33872415	3.39E-14	3.21E-13	1	20.2379405	4.33899057	3.91E-07	2.51E-06	1	0.50009235	-0.9997336	0.05895605	0.32827801	0
yhaH	0.82692785	-0.2741666	0.45591595	0.52592863	0	0.55516974	-0.8489992	0.01542053	0.03066756	0	0.67136418	-0.5748325	0.06081225	0.33554689	0
yhaI	7.66607466	2.93848805	4.77E-06	1.73E-05	1	5.20541267	2.38001254	0.00054297	0.00159005	1	0.6790193	-0.5584755	0.59776304	0.92806272	0
yhaJ	1.96504209	0.97456022	0.00527963	0.01001724	0	1.97990737	0.98543293	0.00848561	0.01813245	0	1.00756486	0.01087272	0.79497647	0.96579786	0
yhaK	1.67431393	0.74357006	0.17202305	0.22654317	0	1.78755206	0.83798526	0.11783101	0.17874879	0	1.06763255	0.0944152	0.6527384	0.93490625	0
yhaL	0.28660967	-1.8028408	0.02868006	0.04609891	-1	0.93566024	-0.0959433	0.54331539	0.62867523	0	3.2645802	1.70689748	0.18113881	0.64917581	0
rclR	2.06459964	1.04586204	0.01395086	0.0241556	1	1.48803613	0.57340956	0.11619323	0.1766281	0	0.72073835	-0.4724525	0.62558649	0.92989404	0
yhaM	0.69694665	-0.5208799	0.2649376	0.33122824	0	0.96332799	-0.053901	0.80778344	0.85294982	0	1.38221195	0.46697886	0.18470533	0.65425743	0
yhaO	0.60744503	-0.7191742	0.21805311	0.27900943	0	0.57828108	-0.7901572	0.31967983	0.41002247	0	0.95198915	-0.070983	1	1	0
tdcG	0.38645842	-1.3716149	0.00051265	0.00122069	-1	0.44277394	-1.1753578	0.007927	0.01714606	-1	1.14572206	0.1962571	0.67057186	0.93979017	0
tdcF	0.80611897	-0.3109353	0.39515946	0.46718932	0	0.34097604	-1.5522577	7.07E-05	0.00026361	-1	0.42298476	-1.2413224	0.00069138	0.01275731	-1
tdcE	0.11103111	-3.1709641	5.94E-20	8.69E-19	-1	0.14356866	-2.8001873	1.70E-11	2.29E-10	-1	1.29304889	0.37077682	0.23674436	0.72916279	0
tdcD	0.02780474	-5.1685251	1.13E-42	8.49E-41	-1	0.02589277	-5.2713071	9.26E-30	2.05E-27	-1	0.93123555	-0.102782	1	1	0
tdcC	0.01094596	-6.5134574	1.01E-61	1.85E-59	-1	0.02359122	-5.4056059	3.31E-33	1.13E-30	-1	2.15524451	1.10785155	0.00167583	0.02530956	1
tdcB	0.0052208	-7.5815134	1.40E-76	1.24E-73	-1	0.01355633	-6.2048892	4.05E-41	3.58E-38	-1	2.5966008	1.37662423	0.00020669	0.00469844	1
tdcA	0.00940857	-6.7318091	1.53E-61	2.70E-59	-1	0.05173465	-4.2727253	1.09E-25	1.33E-23	-1	5.49867429	2.45908383	9.65E-08	8.19E-06	1
tdcR	0.74878161	-0.4173831	0.8812517	0.90734004	0	3.36937039	1.75247903	0.35690618	0.4480592	0	4.49980387	2.16986212	0.36833605	0.85457865	0
ykgE	0.04095793	-4.6097133	6.61E-34	2.73E-32	-1	0.04201203	-4.5730536	5.91E-23	5.03E-21	-1	1.02573617	0.0366597	0.68121234	0.94348666	0
yhaB	2.84981871	1.51087015	0.02661454	0.04317166	1	2.73619529	1.4521712	0.03153041	0.05783847	0	0.9601296	-0.0586989	0.80987047	0.96990832	0
yhaC	2.97730047	1.57400482	0.37089701	0.44145839	0	1.34454863	0.42712194	0.53810091	0.62368924	0	0.45159991	-1.1468829	0.78902248	0.96411212	0
rnpB	0.14160014	-2.8201054	7.25E-18	9.50E-17	-1	0.15728563	-2.6685412	1.05E-13	1.99E-12	-1	1.11077317	0.15156424	0.66528007	0.9379428	0
garK	0.43051051	-1.2158796	0.00151788	0.00327207	-1	0.41967052	-1.252671	0.01354929	0.02725277	-1	0.97482061	-0.0367913	0.74542085	0.95683847	0
garR	0.11103344	-3.1709339	1.05E-15	1.14E-14	-1	0.10308013	-3.2781618	3.42E-12	5.10E-11	-1	0.92837016	-0.1072279	0.93276554	1	0
garL	0.08001858	-3.6435211	1.27E-16	1.50E-15	-1	0.08406155	-3.5724102	5.53E-11	6.99E-10	-1	1.0505253	0.0711109	0.55329463	0.92069223	0
garP	0.14990025	-2.7379253	1.51E-08	7.89E-08	-1	0.1867213	-2.4210416	6.62E-05	0.00024924	-1	1.24563701	0.31688371	0.3437573	0.84269271	0
garD	0.1016014	-3.2990078	6.19E-12	4.80E-11	-1	0.125701	-2.991932	4.81E-07	3.02E-06	-1	1.23719755	0.30707588	0.30833076	0.81055096	0
prlF	0.59872255	-0.7400405	0.1547846	0.20624235	0	0.72831631	-0.4573629	0.12743581	0.19089453	0	1.21645045	0.28267755	0.77462908	0.96287211	0
yhaV	0.49715322	-1.0082376	0.00203689	0.00427612	-1	0.56919062	-0.8130162	0.02160976	0.041375	0	1.14489981	0.19522135	0.89789316	0.99640384	0
ykgF	0.08593081	-3.5406807	1.19E-22	2.12E-21	-1	0.07795193	-3.6812715	1.33E-16	3.87E-15	-1	0.90714755	-0.1405909	1	1	0
agaR	0.33998823	-1.5564433	5.42E-06	1.95E-05	-1	0.3440076	-1.5394876	0.00015079	0.00051696	-1	1.01182211	0.01695567	0.89839409	0.99645823	0
kbaZ	0.61093048	-0.7109199	0.15462239	0.20614763	0	0.88039823	-0.1837719	1	1	0	1.4410776	0.52714803	0.14498902	0.57607933	0
agaV	0.22605579	-2.1452492	3.58E-05	0.00011036	-1	0.47859863	-1.0631118	0.14633944	0.21512094	0	2.11717038	1.08213738	0.02648997	0.19245595	0
agaW	0.65582016	-0.6086278	0.74998441	0.79708401	0	0.66057033	-0.5982159	0.9459364	0.96267854	0	1.00724309	0.01041191	0.73840694	0.95683847	0
agaA	1.13117288	0.17781944	0.50273635	0.57246364	0	1.54746826	0.62990982	0.10292383	0.16048323	0	1.368021	0.45209038	0.25170558	0.74992188	0
agaS	1.95878315	0.96995769	0.05199586	0.07878698	0	1.97115284	0.97903964	0.03995414	0.07113511	0	1.00631499	0.00908195	0.69455009	0.94773784	0
kbaY	2.27456303	1.18558941	0.03774549	0.05924727	0	2.75769718	1.46346404	0.03477915	0.06298732	0	1.21240746	0.27787463	0.66596948	0.93829234	0
agaB	3.36548583	1.75081478	0.02285094	0.03757989	1	1.93903205	0.95533665	0.08125943	0.13129087	0	0.57615219	-0.7954781	0.71254973	0.95223057	0
agaC	6.43689803	2.68636561	7.16E-06	2.52E-05	1	7.73800779	2.95196218	3.83E-06	1.95E-05	1	1.20213304	0.26559657	0.45799332	0.88755478	0
agaD	3.69710005	1.88639409	0.00366205	0.00721144	1	6.11563953	2.61250337	0.00126907	0.00340914	1	1.65417204	0.72610929	0.36628089	0.85342842	0
carB	1.85694591	0.89293179	0.01091943	0.01936993	0	2.45822292	1.29761575	0.00022668	0.00073927	1	1.32379889	0.40468396	0.13448385	0.54914241	0
ykgG	0.08984117	-3.4764795	1.74E-19	2.48E-18	-1	0.08662141	-3.5291326	1.42E-14	3.00E-13	-1	0.96416162	-0.0526531	0.91703934	1	0
agaI	6.26401106	2.64708676	0.00057456	0.00135496	1	7.44704426	2.89666793	0.00045143	0.00134698	1	1.18886193	0.24958117	0.59185694	0.92806272	0
yraH	2.36858016	1.2440225	0.32747628	0.39683477	0	2.17221256	1.11916528	0.28628635	0.37606161	0	0.91709481	-0.1248572	0.89452568	0.9954996	0
yraI	8.22089345	3.0392952	1.57E-08	8.18E-08	1	7.15126452	2.83819837	1.11E-06	6.48E-06	1	0.86988897	-0.2010968	0.91532976	1	0
yraJ	3.51552188	1.81373887	4.55E-07	1.92E-06	1	4.2472917	2.08654319	1.45E-05	6.51E-05	1	1.20815397	0.27280432	0.82808277	0.97793095	0
yraK	1.76745139	0.82167053	0.01752875	0.0295852	0	2.03432325	1.02454894	0.01721133	0.03380305	1	1.15099248	0.20287841	0.61820477	0.92897598	0
rsmI	1.2642315	0.33826067	0.4331028	0.50462017	0	1.26805397	0.34261616	0.28525979	0.37505594	0	1.00302355	0.00435548	0.72017866	0.95399541	0
lpoA	0.34506285	-1.5350689	3.43E-06	1.28E-05	-1	0.40569889	-1.3015187	0.00063063	0.00182141	-1	1.17572465	0.23355022	0.64038586	0.93163024	0
yraN	0.44216127	-1.1773554	0.0063165	0.01178706	-1	0.4449142	-1.168401	0.03656807	0.06584935	0	1.00622608	0.00895449	0.8897447	0.99411016	0
diaA	0.17748166	-2.4942582	8.71E-11	5.94E-10	-1	0.38180473	-1.3890931	0.00050332	0.00147884	-1	2.15123484	1.10516503	0.0235308	0.17558512	0
yraP	0.1439414	-2.7964465	8.05E-14	7.38E-13	-1	0.1864029	-2.4235038	2.60E-08	2.13E-07	-1	1.29499156	0.37294269	0.38238659	0.86036984	0
ykgH	1.56411856	0.64534987	0.39695788	0.46906446	0	1.94573812	0.96031755	0.17439548	0.24979853	0	1.24398378	0.31496768	0.54892243	0.91938773	0
yraQ	2.83694742	1.50433941	0.00078588	0.00180606	1	3.28289202	1.7149673	0.00049165	0.00144839	1	1.1571917	0.21062788	0.51190745	0.90925684	0
yraR	1.96058441	0.97128376	0.01416814	0.02450301	0	1.33655554	0.41851979	0.14087017	0.2082654	0	0.68171283	-0.552764	0.51575624	0.90937129	0
yhbO	6.98343585	2.80393702	0.00554644	0.01049192	1	2.28449351	1.19187434	0.20191336	0.28137974	0	0.32713031	-1.6120627	0.21299135	0.69446947	0
yhbP	1.71379947	0.77719831	0.04150301	0.06448076	0	1.88350963	0.91342341	0.0414374	0.07336213	0	1.09902568	0.1362251	0.6877304	0.94389281	0
yhbQ	0.33966241	-1.5578265	0.00214915	0.0044735	-1	0.79111382	-0.3380428	0.65164153	0.72678285	0	2.32911792	1.21978368	0.02498631	0.18419859	0
yhbS	0.0969718	-3.3662909	1.06E-22	1.90E-21	-1	0.14869321	-2.7495893	2.84E-11	3.74E-10	-1	1.53336547	0.6167016	0.05880076	0.32782724	0
yhbT	0.07034705	-3.8293663	1.97E-27	5.17E-26	-1	0.11482815	-3.1224517	1.51E-15	3.83E-14	-1	1.63230947	0.70691461	0.1892468	0.66370038	0
yhbU	0.07829627	-3.6749126	8.60E-19	1.19E-17	-1	0.17408186	-2.5221622	9.57E-08	7.08E-07	-1	2.22337365	1.15275042	0.00705786	0.07204903	0
yhbV	0.3700195	-1.4343268	7.82E-05	0.00022457	-1	0.41755112	-1.2599753	0.01046611	0.02188819	-1	1.12845707	0.17435153	0.43318617	0.88049119	0
yhbW	1.38294718	0.46774605	0.11955995	0.16385241	0	1.23781009	0.30778999	0.26808815	0.35544001	0	0.89505233	-0.1599561	0.86932359	0.98621446	0
betA	1.13410963	0.18156011	0.39190979	0.46384393	0	0.86329822	-0.2120691	0.87896797	0.90963922	0	0.76121232	-0.3936292	0.42361094	0.87508931	0
mtr	10.0539322	3.32968796	1.67E-14	1.63E-13	1	1.4527583	0.53879469	0.09208897	0.14596167	0	0.14449653	-2.7908933	4.58E-07	3.15E-05	-1
deaD	0.23656886	-2.0796679	5.42E-10	3.42E-09	-1	0.41190922	-1.2796017	0.00208274	0.00525022	-1	1.74118109	0.80006626	0.01673597	0.13617276	0
yrbN	0.34142755	-1.5503486	0.07182826	0.10400566	0	0.35594397	-1.490278	0.13794714	0.20435415	0	1.04251682	0.06007066	1	1	0
nlpI	0.24834025	-2.00961	5.19E-10	3.29E-09	-1	0.41615029	-1.2648234	9.02E-05	0.00032814	-1	1.67572629	0.74478652	0.1970164	0.674567	0
pnp	0.24158703	-2.0493851	6.35E-11	4.42E-10	-1	0.29233472	-1.7743069	1.65E-06	9.28E-06	-1	1.21005965	0.27507816	0.4960511	0.90539057	0
psrO	0.2855406	-1.8082322	0.000553	0.00130971	-1	0.66835463	-0.5813143	0.20613606	0.28645134	0	2.3406641	1.22691791	0.11222713	0.4958313	0
rpsO	0.26214578	-1.9315588	0.0169822	0.02880583	-1	0.8566831	-0.2231665	0.48688104	0.57512091	0	3.26796447	1.7083923	0.1802197	0.6466793	0
truB	0.25469355	-1.9731656	1.91E-08	9.72E-08	-1	0.33366224	-1.5835397	6.73E-05	0.00025291	-1	1.31005373	0.38962598	0.45189398	0.88755478	0
rbfA	0.12530845	-2.9964444	1.47E-14	1.45E-13	-1	0.12016491	-3.0569124	1.36E-11	1.85E-10	-1	0.95895299	-0.060468	0.64605663	0.93208609	0
infB	0.15676392	-2.6733346	9.84E-17	1.17E-15	-1	0.18547147	-2.4307308	3.29E-10	3.77E-09	-1	1.18312605	0.24260379	0.48180446	0.89935549	0
betB	0.96806259	-0.0468278	0.99388842	0.99886351	0	0.76065618	-0.3946836	0.54190508	0.62720759	0	0.78575103	-0.3478558	0.54312472	0.91914725	0
nusA	0.22278746	-2.16626	4.45E-11	3.13E-10	-1	0.30430864	-1.7163928	8.31E-06	4.00E-05	-1	1.36591455	0.44986724	0.24819757	0.74826869	0
rimP	0.36614806	-1.4495009	0.00034831	0.00085948	-1	0.63832836	-0.6476294	0.06865678	0.11356786	0	1.7433613	0.80187159	0.18376947	0.65304057	0
metY	0.82257271	-0.2817849	0.61834483	0.67890358	0	1.75757384	0.8135853	0.71860869	0.78388837	0	2.13667899	1.09537018	0.42982818	0.87797234	0
argG	0.60558562	-0.7235971	0.02581938	0.04197431	0	0.57250346	-0.8046437	0.06648652	0.11053572	0	0.94537162	-0.0810465	0.98347806	1	0
yhbX	2.22411974	1.15323446	0.1854192	0.24202028	0	1.48070132	0.56628065	0.38574859	0.47688003	0	0.66574712	-0.5869538	0.70105513	0.94869712	0
leuU	0.3820405	-1.3882025	0.14492353	0.1946267	0	1.77902407	0.83108603	0.58856818	0.67003696	0	4.65663739	2.21928854	0.05654713	0.31970877	0
secG	0.32852799	-1.6059118	0.01592605	0.02716038	-1	1.1409199	0.19019751	0.81760149	0.86105362	0	3.472824	1.7961093	0.06153111	0.33750271	0
glmM	0.11499553	-3.1203503	7.61E-21	1.18E-19	-1	0.12459369	-3.0046971	2.06E-13	3.75E-12	-1	1.08346548	0.11565319	0.59211018	0.92806272	0

folP	0.18541549	-2.4311663	8.01E-11	5.49E-10	-1	0.258907	-1.9494941	1.63E-05	7.22E-05	-1	1.39636123	0.4816722	0.200788	0.67800543	0
ftsH	0.30535951	-1.7114193	1.59E-08	8.26E-08	-1	0.2719252	-1.8787183	2.14E-07	1.48E-06	-1	0.89050836	-0.1672989	0.4952287	0.90539057	0
betI	0.66227602	-0.5944955	0.07664819	0.11009921	0	0.57226135	-0.8052539	0.09421687	0.14890714	0	0.86408286	-0.2107584	0.75611657	0.9573569	0
rlmE	0.42569185	-1.2321186	0.0002335	0.00060158	-1	0.38980079	-1.3591911	9.87E-05	0.00035558	-1	0.9156877	-0.1270725	0.35529329	0.84810955	0
yhbY	0.27556537	-1.8595335	1.44E-07	6.51E-07	-1	0.40478457	-1.3047738	0.00051428	0.00150904	-1	1.46892393	0.55475969	0.3658944	0.85342842	0
greA	0.14820301	-2.7543533	7.11E-11	4.91E-10	-1	0.3079452	-1.6992544	0.00015675	0.00053447	-1	2.07786062	1.05509888	0.06843279	0.36229126	0
dacB	1.4776325	0.5632875	0.05181251	0.07853668	0	1.65093162	0.72328036	0.0434352	0.0764093	0	1.11728161	0.15999286	0.60639505	0.92806272	0
obgE	0.57203132	-0.8058339	0.02187511	0.03610701	0	0.80924625	-0.3053493	0.70625235	0.77288983	0	1.4146887	0.50048463	0.11599527	0.50522834	0
yhbE	0.97479726	-0.0368259	0.8984339	0.92102287	0	1.22106908	0.28814482	0.50532078	0.59309761	0	1.25263901	0.32497071	0.43399119	0.88076446	0
rpmA	0.10706301	-3.2234679	6.89E-21	1.07E-19	-1	0.39317142	-1.3467696	8.27E-05	0.00030396	-1	3.67233659	1.8766983	4.71E-06	0.00022554	1
rplU	0.12830607	-2.9623387	1.71E-12	1.39E-11	-1	0.45802377	-1.1265056	0.00296336	0.00714862	-1	3.56977485	1.83583308	0.00088774	0.01526037	1
ispB	0.33443057	-1.5802214	4.25E-07	1.80E-06	-1	0.43153758	-1.2124419	0.00108086	0.00296666	-1	1.29036522	0.36777946	0.36673617	0.85342842	0
sfsB	1.08010534	0.11117203	1	1	0	1.18015646	0.23897813	0.82789058	0.86940315	0	1.09263088	0.12780611	0.87216669	0.98773886	0
betT	2.49154163	1.31703868	0.0001205	0.00032998	1	3.08297689	1.62432407	4.51E-05	0.00017793	1	1.23737723	0.30728539	0.37445475	0.85857675	0
murA	0.43507778	-1.2006548	0.00068436	0.00158845	-1	0.63199324	-0.662019	0.12182723	0.18398992	0	1.4525983	0.53863579	0.16038388	0.60475422	0
ibaG	0.34719642	-1.526176	0.00080681	0.00184552	-1	0.54979303	-0.8630395	0.11302149	0.17239971	0	1.58352159	0.66313653	0.2375346	0.72998439	0
mlaB	0.38466493	-1.3783258	0.00073687	0.00170764	-1	0.29833473	-1.7449962	0.00020305	0.0006691	-1	0.7755704	-0.3666704	0.30897466	0.81096368	0
mlaC	0.41699552	-1.2618962	0.00010484	0.00029142	-1	0.30894878	-1.6945604	3.76E-05	0.00015165	-1	0.74089231	-0.4326642	0.26351674	0.762024	0
mlaD	0.72137042	-0.4711878	0.53333098	0.60064328	0	0.71275976	-0.4885122	0.32947312	0.41905204	0	0.98806346	-0.0173244	0.80223112	0.96927102	0
mlaE	1.21462663	0.28051291	0.42539194	0.49786863	0	1.25747725	0.3305323	0.2891972	0.37854337	0	1.03527884	0.0500194	0.71393959	0.95223057	0
mlaF	0.63571082	-0.6535575	0.07800354	0.11179157	0	0.98650935	-0.0195954	0.75675705	0.81365193	0	1.55182093	0.63396209	0.21037509	0.69080725	0
yrbG	0.67536473	-0.5662613	0.13116467	0.17793438	0	0.85673594	-0.2230775	0.53243085	0.61883533	0	1.26855299	0.34318378	0.51115823	0.90925684	0
kdsD	0.40421924	-1.3067901	0.00056778	0.00133969	-1	0.44788268	-1.1588072	0.00985282	0.02081243	-1	1.10801918	0.14798285	0.70147991	0.94869712	0
kdsC	0.33427672	-1.5808852	0.00015916	0.00042554	-1	0.47046409	-1.0878435	0.01005766	0.02117427	-1	1.40740905	0.4930417	0.47379559	0.89828993	0
yahA	2.1407452	1.09811309	0.09308191	0.13085385	0	1.97178355	0.97950119	0.08356884	0.13458116	0	0.92107344	-0.1186119	0.79458009	0.96579786	0
lptC	0.51986756	-0.943784	0.00219631	0.00456308	0	0.51787342	-0.9493286	0.01194798	0.02452305	0	0.99616412	-0.0055446	1	1	0
lptA	0.29589339	-1.7568506	2.26E-07	9.94E-07	-1	0.32013289	-1.6432572	5.31E-05	0.00020559	-1	1.08191971	0.11359344	0.8178588	0.97348376	0
lptB	0.44555951	-1.16631	0.00041258	0.00099912	-1	0.40070107	-1.3194017	0.00141314	0.00375055	-1	0.89932111	-0.1530918	0.73297477	0.95599369	0
rpoN	0.34755505	-1.5246866	1.57E-06	6.18E-06	-1	0.31141295	-1.6830992	7.39E-06	3.58E-05	-1	0.89601043	-0.1584126	0.55024175	0.91976447	0
hpf	0.27957049	-1.838716	1.83E-09	1.07E-08	-1	0.12244274	-3.0298208	4.41E-14	8.94E-13	-1	0.43796734	-1.1911048	0.00083829	0.01461211	-1
ptsN	0.31383417	-1.6719257	4.90E-07	2.06E-06	-1	0.22403772	-2.1581864	1.02E-07	7.57E-07	-1	0.71387294	-0.4862608	0.17056287	0.62318331	0
yhbJ	0.46939496	-1.0911257	0.00062705	0.00146544	-1	0.33085549	-1.5957269	0.00018262	0.00060813	-1	0.70485522	-0.5046011	0.2875111	0.78998378	0
npr	1.70768528	0.77204212	0.06375841	0.09395217	0	1.77871101	0.83083213	0.22732746	0.3115881	0	1.04159181	0.05879001	0.8366619	0.98025477	0
yrbL	1.22733362	0.29552746	0.54282462	0.61009067	0	0.4445993	-1.1694224	0.00150027	0.00395332	-1	0.36224812	-1.4649499	0.00015477	0.00383444	-1
mtgA	0.85363688	-0.2283056	0.63578617	0.69374066	0	0.4517276	-1.146475	0.00486018	0.01107639	-1	0.52918004	-0.9181695	0.01489449	0.12511373	0
yahB	1.60756352	0.68487574	0.0625907	0.09243167	0	1.58492222	0.66441204	0.16675414	0.24112779	0	0.98591577	-0.0204637	0.91059129	0.99971546	0
elbB	0.18341491	-2.4468171	1.26E-13	1.13E-12	-1	0.15121645	-2.725313	8.93E-11	1.09E-09	-1	0.82445013	-0.2784959	0.59961841	0.92806272	0
arcZ	0.21544677	-2.2145967	3.79E-05	0.00011635	-1	0.16224315	-2.6237705	0.00016801	0.00056717	-1	0.75305446	-0.4091739	0.58649484	0.92806272	0
arcB	0.24890503	-2.0063327	8.61E-10	5.31E-09	-1	0.27692865	-1.8524138	2.30E-06	1.25E-05	-1	1.1125876	0.15391894	0.67081931	0.93979017	0
yhcC	1.3525817	0.43571574	0.15847453	0.21045761	0	1.61826434	0.69444729	0.12258936	0.1848251	0	1.19642631	0.25873154	0.60247407	0.92806272	0
gltB	0.63741819	-0.6496879	0.05988908	0.08890592	0	0.96286627	-0.0545927	0.88512977	0.91307679	0	1.51057231	0.59509524	0.06644676	0.35347432	0
gltD	0.30696692	-1.7038449	4.74E-06	1.73E-05	-1	0.56459125	-0.8247213	0.06658955	0.11062377	0	1.83925764	0.87912358	0.02662364	0.19310898	0
gltF	0.69446768	-0.5260205	0.2907024	0.35816807	0	0.57427964	-0.8001747	0.38089513	0.47213902	0	0.826935	-0.2741542	0.94544158	1	0
yhcA	5.88029192	2.55588778	0.00029417	0.00073827	1	5.7114979	2.51386916	0.00203152	0.00514752	1	0.97129496	-0.0420186	0.99722666	1	0
yhcD	2.50213033	1.32315694	0.00082381	0.00188147	1	2.77248454	1.47117942	0.00102874	0.00284303	1	1.10804961	0.14802248	0.64585077	0.93208609	0
yhcE	1.79929267	0.84742987	0.18809046	0.24500017	0	1.9261455	0.94571669	0.14620565	0.21500259	0	1.0705015	0.09828682	0.78453466	0.9635071	0
yahC	7.00069265	2.80749767	5.04E-06	1.83E-05	1	7.38806424	2.88519641	1.32E-05	6.02E-05	1	1.05533332	0.07769874	0.68538428	0.943596	0
yhcF	2.27356013	1.18495316	0.01504407	0.02579568	1	1.49885081	0.58385679	0.2456529	0.33109899	0	0.65925277	-0.6010964	0.37051008	0.85484012	0
yhcG	2.99280693	1.58149921	0.00105477	0.00235419	1	2.23887412	1.16277341	0.04427273	0.07754309	0	0.74808505	-0.4187258	0.42043378	0.87375001	0
yhcH	0.27252898	-1.8755184	8.79E-06	3.05E-05	-1	0.14257974	-2.8101591	3.19E-07	2.09E-06	-1	0.52317274	-0.9346407	0.08910978	0.42947991	0
nanK	0.28994167	-1.7861654	1.30E-05	4.34E-05	-1	0.26323069	-1.9256004	0.00023909	0.00077574	-1	0.90787462	-0.139435	1	1	0
nanE	0.22171039	-2.1732517	4.31E-08	2.11E-07	-1	0.19326032	-2.3713826	6.40E-06	3.13E-05	-1	0.87167912	-0.1981309	0.986673	1	0
nanT	0.30561242	-1.7102249	1.88E-06	7.30E-06	-1	0.30232788	-1.7258141	0.0001634	0.00055375	-1	0.9892526	-0.0155891	0.77846364	0.9635071	0
nanA	0.09694272	-3.3667236	6.85E-21	1.07E-19	-1	0.08523206	-3.5524599	1.63E-15	4.10E-14	-1	0.87920025	-0.1857363	0.79953198	0.96818334	0
nanR	0.31592015	-1.6623682	1.87E-06	7.27E-06	-1	0.34167767	-1.5492921	0.00026389	0.00084318	-1	1.08153174	0.11307601	0.77934451	0.9635071	0
dcuD	5.25732673	2.3943294	1.76E-08	9.06E-08	1	3.65600615	1.8702685	1.26E-05	5.79E-05	1	0.69541163	-0.5240609	0.52042137	0.91269012	0
yahD	1.59956	0.67767511	0.08511753	0.12088878	0	0.94373642	-0.0835441	0.89311931	0.91878132	0	0.58999751	-0.7612192	0.25010812	0.7487007	0
sspB	0.23533812	-2.087193	8.39E-10	5.19E-09	-1	0.33447113	-1.5800464	3.57E-05	0.00014472	-1	1.42123649	0.50714664	0.28564587	0.78928465	0
sspA	0.16032208	-2.640955	3.31E-16	3.71E-15	-1	0.24675809	-2.0188307	4.35E-08	3.42E-07	-1	1.53913978	0.62212426	0.14475303	0.57607933	0
rpsI	0.03890497	-4.6839018	1.32E-19	1.90E-18	-1	0.21708091	-2.2036952	2.59E-05	0.00010826	-1	5.57977358	2.48020658	9.64E-06	0.00040492	1
rplM	0.02995176	-5.0612154	2.46E-25	5.57E-24	-1	0.24106484	-2.0525069	1.97E-05	8.48E-05	-1	8.04843624	3.0087085	4.21E-09	5.16E-07	1
zapE	1.24320623	0.31406564	0.31175432	0.3801819	0	1.58056057	0.66043632	0.14551315	0.21419807	0	1.27135831	0.34637068	0.48762668	0.90208042	0
yhcB	0.1759692	-2.5066052	2.22E-15	2.36E-14	-1	0.16253224	-2.6212021	5.67E-12	8.27E-11	-1	0.92364031	-0.114597	0.51853311	0.91161924	0
degQ	0.36479261	-1.4548516	5.80E-05	0.00017146	-1	0.27823322	-1.8456334	8.48E-05	0.00031024	-1	0.76271616	-0.3907818	0.55098151	0.92038542	0
degS	0.79140879	-0.337505	0.40126037	0.47389493	0	0.87997264	-0.1844694	0.67739724	0.74947882	0	1.11190657	0.15303557	0.76888879	0.96147692	0
mdh	0.17297488	-2.5313655	9.73E-16	1.05E-14	-1	0.14260785	-2.8098746	1.30E-13	2.46E-12	-1	0.82444256	-0.2785091	0.30410493	0.80892599	0
argR	0.36239279	-1.4643739	1.42E-05	4.69E-05	-1	0.29035638	-1.7841034	1.93E-05	8.33E-05	-1	0.80122008	-0.3197295	0.36328986	0.85329623	0
caiF	0.17694065	-2.4986626	6.99E-09	3.81E-08	-1	0.10619013	-3.2352784	1.09E-07	8.03E-07	-1	0.60014548	-0.7366158	0.53838586	0.91897962	0
yahE	6.78885721	2.76316874	6.74E-08	3.18E-07	1	6.2933777	2.65383453	2.00E-06	1.10E-05	1	0.92701577	-0.1093342	1	1	0
yhcN	0.49747775	-1.0072961	0.00329017	0.0065551	-1	0.24805551	-2.0112651	8.34E-05	0.00030625	-1	0.49862634	-1.003969	0.05767423	0.32277076	0
yhcO	3.47311144	1.7962287	0.00306908	0.00616469	1	1.24291132	0.31372336	0.66856326	0.74156151	0	0.35786681	-1.4825053	0.03526803	0.23423493	0
aaeB	3.41155448	1.77042926	7.14E-05	0.00020701	1	3.95543225	1.98383536	0.00017732	0.00059453	1	1.15942227	0.21340611	0.64471794	0.93208609	0
aaeA	2.02564813	1.01838359	0.03423474	0.05417946	0	2.04216887	1.03010217	0.03326063	0.06060979	0	1.00815578	0.01171858	0.7872853	0.96350911	0
aaeX	1.33849644	0.4206133	0.55751076	0.62342406	0	1.03269342	0.04641202	0.61051118	0.68963418	0	0.77153244	-0.3742013	0.92929487	1	0
aaeR	0.79221311	-0.3360395	0.43800066	0.50911814	0	0.78100937	-0.3565882	0.6287198	0.70712975	0	0.98585766	-0.0205487	0.84385149	0.98070401	0
tldD	0.4158385	-1.2659048	0.00011014	0.00030424	-1	0.32718801	-1.6118082	4.27E-05	0.00016948	-1	0.7868151	-0.3459034	0.3349102	0.83443729	0
yhdP	0.62251671	-0.6838155	0.03180149	0.05063706	0	0.80831976	-0.307002	0.48381207	0.5724352	0	1.29847079	0.37681356	0.26179507	0.7605509	0
rng	0.38453579	-1.3788102	2.54E-05	8.02E-05	-1	0.54827274	-0.8670343	0.01597962	0.03158981	0	1.42580419	0.51177587	0.26021274	0.75894691	0
yhdE	0.99397498	-0.0087186	0.97178311	0.98133653	0	1.0995634	0.13693079	0.79236464	0.84146384	0	1.10622844	0.14564934	0.79743559	0.96743774	0
yahF	3.7026187	1.88854599	0.00155738	0.00335394	1	4.24690966	2.08641342	0.00066028	0.00189713	1	1.14700162	0.19786743	0.51306172	0.90937129	0
mreD	1.00672805	0.00967402	0.51700561	0.58512803	0	1.54059151	0.62348438	0.19399846	0.27301886	0	1.5302956	0.61381036	0.32332982	0.82325896	0
mreC	0.78489475	-0.3494289	0.3377895	0.40743207	0	2.06593367	1.04679393	0.02525319	0.04736581	1	2.63211553	1.39622281	0.00134595	0.02119871	1
mreB	0.37737969	-1.4059113	9.72E-06	3.34E-05	-1	0.74999681	-0.4150436	0.21788765	0.30032612	0	1.98737988	0.99086767	0.00976266	0.0919608	0
csrD	0.84180338	-0.2484448	0.4313007	0.50305128	0	0.87858494	-0.1867463	0.63824979	0.71602585	0	1.04369377	0.06169847	0.87026315	0.98659653	0
acuI	0.26735131	-1.9031913	4.58E-08	2.22E-07	-1	0.23180287	-2.1090296	2.97E-06	1.57E-05	-1	0.86703473	-0.2058383	0.91258806	1	0
yrdF	0.36854848	-1.4400737	0.15943301	0.21166685	0	0.51933911	-0.9452512	0.66215749	0.73630446	0	1.40914736	0.49482249	0.49381629	0.90539057	0
yrdE	2.45346996	1.29482361	0.33180245	0.40141644	0	3.40768347	1.76879133	0.23515317	0.31969115	0	1.38892407	0.47396773	0.74405908	0.95683847	0
accB	0.12974281	-2.9462735	1.75E-17	2.21E-16	-1	0.47795002	-1.0650683	0.00550564	0.01240032	-1	3.68382671	1.8812052	5.88E-07	3.99E-05	1
accC	0.10201253	-3.2931817	1.57E-20	2.37E-19	-1	0.35556796	-1.4918028	0.00013993	0.00048459	-1	3.48553208	1.80137891	1.54E-06	8.73E-05	1
yhdT	1.31568708	0.3958164	0.55113787	0.61754845	0	2.64530166	1.40343225	0.14187179	0.20953591	0	2.01058573	1.00761585	0.30597507	0.81005169	0
yahG	3.68536739	1.88180845	0.00075663	0.001747	1	7.5681544	2.91994152	2.14E-05	9.15E-05	1	2.0535685	1.03813307	0.11868332	0.51429541	0
panF	2.36336642	1.24084333	0.00213996	0.00445859	1	3.06182509	1.61439187	0.00032858	0.00102021	1	1.2955355	0.37354854	0.31607067	0.81560659	0
prmA	0.99835758	-0.0023715	0.88034559	0.90661843	0	1.13122995	0.17789223	0.79628326	0.84403351	0	1.13309096	0.18026368	0.84159707	0.98067952	0
dusB	0.15367155	-2.702078	2.41E-13	2.12E-12	-1	0.49031573	-1.028217	0.00692057	0.01522251	-1	3.19067336	1.67386092	7.96E-05	0.00223485	1
fis	0.09605595	-3.3799812	3.11E-15	3.25E-14	-1	0.4352292	-1.2001528	0.00308177	0.00739714	-1	4.53099672	2.17982845	2.34E-05	0.0008078	1
yhdJ	14.7350573	3.88118077	0.00052982	0.00125954	1	24.4083126	4.60930066	0.00057369	0.00166895	1	1.65647898	0.72811989	0.63919491	0.93163024	0
yhdU	3.75946695	1.91052812	0.20938523	0.26900947	0	6.32812786	2.66177875	0.08946026	0.14215206	0	1.68325136	0.75125063	0.5535943	0.92069223	0
acrS	5.50852207	2.4616653	0.00058165	0.00136948	1	3.7171093	1.89418111	0.00830507	0.01780695	1	0.67479248	-0.5674842	0.68311857	0.94348666	0
acrE	5.33994835	2.41682579	1.18E-07	5.38E-07	1	6.0219723	2.59023607	2.67E-07	1.79E-06	1	1.12772108	0.17341028	0.547536	0.91914725	0
acrF	4.36203297	2.12500067	1.45E-09	8.60E-09	1	4.44630148	2.15260577	2.56E-07	1.73E-06	1	1.01931863	0.0276051	1	1	0
yhdV	5.66672179	2.50251437	0.05173835	0.07847812	0	7.49075056	2.90511028	0.04350101	0.07649461	0	1.3218843	0.40259591	0.7406135	0.95683847	0
yahI	2.56125832	1.35685277	0.00319138	0.00638131	1	4.51059108	2.1733165	0.00010133	0.00036404	1	1.76108401	0.81646373	0.1262576	0.5319293	0

yhdW	3.33755899	1.73879334	5.28E-05	0.00015848	1	4.13024936	2.04622889	2.28E-05	9.68E-05	1	1.23750603	0.30743555	0.40846348	0.86959974	0
yhdX	5.56800823	2.47716134	2.24E-06	8.65E-06	1	4.01788823	2.00643743	0.00028004	0.00088836	1	0.72160242	-0.4707239	0.64082282	0.93163024	0
yhdY	4.16706495	2.05903159	8.19E-05	0.00023438	1	5.06404438	2.34029005	0.00012038	0.00042319	1	1.21525449	0.28125846	0.57061877	0.92806272	0
yhdZ	3.86687197	1.951167	0.01928075	0.03217344	1	3.52283598	1.8167373	0.03871946	0.06921573	0	0.9110299	-0.1344297	1	1	0
rrfF	3.80706753	1.92868016	0.4631464	0.53301658	0	4.0539785	2.01933844	0.31593016	0.40595969	0	1.06485595	0.09065828	0.74806689	0.95683847	0
thrV	7.76495575	2.9569777	0.04934138	0.07507436	0	9.63775883	3.2686977	0.0222118	0.04234423	1	1.24118658	0.31172	0.60491441	0.92806272	0
rrfD	NA	NA	NA	NA	0	Inf	Inf	0.6063402	0.68651393	0	Inf	Inf	0.68088579	0.94335731	0
rrlD	9.51294296	3.24989173	0.24000953	0.30332535	0	23.4110006	4.54911469	0.15665958	0.2281736	0	2.460963	1.29922297	0.61683312	0.92897598	0
yahJ	1.45772788	0.54372143	0.09090091	0.12827774	0	1.55722673	0.63897901	0.06816451	0.11290066	0	1.06825612	0.09525759	0.63001654	0.93109012	0
rrsD	58.3799074	5.86740002	0.07063473	0.1025283	0	49.695642	5.63504744	0.12343244	0.18577928	0	0.85124565	-0.2323526	1	1	0
yrdA	0.4025485	-1.3127655	6.86E-05	0.00019969	-1	0.37565044	-1.4125373	0.00021551	0.00070754	-1	0.93318056	-0.0997718	0.60340157	0.92806272	0
yrdB	0.58359874	-0.7769513	0.11767449	0.1614187	0	0.41953162	-1.2531485	0.04610217	0.0803968	0	0.71887	-0.4761972	0.46173151	0.88879788	0
aroE	2.05650067	1.04019154	0.0043171	0.00834879	1	1.4040584	0.48960294	0.27646483	0.36457716	0	0.68274152	-0.5505886	0.16896218	0.62093602	0
tsaC	1.61959085	0.6956294	0.09320336	0.13098289	0	1.18306666	0.24253137	0.64716833	0.72364293	0	0.73047255	-0.453098	0.29453186	0.79886161	0
yrdD	0.32846265	-1.6061988	2.05E-06	7.94E-06	-1	0.38601413	-1.3732744	0.00023212	0.00075478	-1	1.17521469	0.23292433	0.83410087	0.98014902	0
smg	0.21998648	-2.1845132	1.12E-11	8.46E-11	-1	0.16521223	-2.5976076	2.09E-11	2.80E-10	-1	0.75101084	-0.4130944	0.15190943	0.58868241	0
smf	0.47664257	-1.0690203	0.00554951	0.01049324	-1	0.42578349	-1.2318081	0.01034128	0.02167842	-1	0.89329723	-0.1627878	0.85419388	0.98236299	0
def	0.48259195	-1.0511242	0.00077857	0.00179182	-1	0.29546985	-1.7589172	1.64E-06	9.25E-06	-1	0.61225605	-0.707793	0.02147388	0.16422516	0
fmt	0.61900753	-0.6919711	0.01393541	0.02414779	0	0.39413165	-1.3432505	0.00039892	0.00121075	-1	0.63671544	-0.6512793	0.07691037	0.38841113	0
yahK	1.64527181	0.71832595	0.04165577	0.06465541	0	1.25393294	0.3264602	0.31075572	0.40117719	0	0.76214333	-0.3918658	0.43273489	0.88049119	0
rsmB	0.9644937	-0.0521563	0.71749957	0.76661722	0	0.41173301	-1.280219	0.00111753	0.00305591	-1	0.42689031	-1.2280627	0.00166903	0.02529357	-1
trkA	4.74870132	2.24753302	6.18E-09	3.40E-08	1	2.18951055	1.1306084	0.08925601	0.14198068	0	0.46107565	-1.1169246	0.00164432	0.02509868	-1
mscL	1.81400516	0.85917856	0.19267031	0.25008075	0	0.3400539	-1.5561647	0.04361605	0.07660546	0	0.18746027	-2.4153432	0.00177897	0.02632639	-1
arfA	1.12457637	0.16938164	0.84121416	0.87283132	0	2.2237528	1.15299642	0.26357011	0.35050145	0	1.97741377	0.98361479	0.31212527	0.81207812	0
zntR	0.57835773	-0.789966	0.06838182	0.09951977	0	0.61472722	-0.7019817	0.12320565	0.18554462	0	1.06288409	0.08798427	0.95782186	1	0
yhdN	1.047541	0.06700671	0.86920161	0.89660521	0	0.56132537	-0.8330908	0.1044252	0.16227215	0	0.5358505	-0.9000975	0.06908644	0.36443923	0
rplQ	0.04930304	-4.3421797	1.85E-30	6.25E-29	-1	0.15944611	-2.6488592	6.16E-11	7.67E-10	-1	3.23400193	1.69332054	6.14E-05	0.00185548	1
rpoA	0.04587755	-4.4460677	8.08E-37	3.84E-35	-1	0.13686165	-2.8692098	5.91E-14	1.18E-12	-1	2.98319419	1.57685789	2.84E-05	0.00095018	1
rpsD	0.04664067	-4.4222676	1.55E-37	8.13E-36	-1	0.14204474	-2.8155827	2.14E-13	3.89E-12	-1	3.04551236	1.60668496	6.10E-06	0.00027743	1
rpsK	0.07339223	-3.768229	4.59E-23	8.44E-22	-1	0.20925614	-2.2566582	7.00E-08	5.33E-07	-1	2.85120306	1.51157079	0.00028901	0.00621731	1
yahL	8.16229386	3.02897465	2.00E-05	6.43E-05	1	5.18823371	2.37524347	0.00106119	0.00292175	1	0.63563427	-0.6537312	0.61279772	0.92897598	0
rpsM	0.05902634	-4.0824973	1.09E-28	3.16E-27	-1	0.18019441	-2.4723739	6.19E-10	6.75E-09	-1	3.05277955	1.61012341	6.45E-05	0.0019081	1
rpmJ	0.04589079	-4.4456515	5.42E-23	9.93E-22	-1	0.12371428	-3.0149161	8.89E-10	9.49E-09	-1	2.69584104	1.43073543	0.00574675	0.06166217	0
secY	0.04735674	-4.4002865	2.81E-19	3.97E-18	-1	0.12198748	-3.035195	1.23E-08	1.07E-07	-1	2.57592655	1.36509145	0.01800191	0.14408064	0
rplO	0.03896817	-4.68156	5.08E-18	6.78E-17	-1	0.11763146	-3.0876542	1.39E-07	9.99E-07	-1	3.01865485	1.59390581	0.00770871	0.07726234	0
rpmD	0.03113297	-5.0054131	6.17E-18	8.18E-17	-1	0.10839452	-3.2056363	3.09E-07	2.04E-06	-1	3.48166355	1.7997768	0.00372278	0.04413297	1
rpsE	0.03457233	-4.8542385	9.27E-21	1.42E-19	-1	0.13992127	-2.8373128	2.00E-07	1.39E-06	-1	4.04720422	2.01692565	0.00036716	0.00746166	1
rplR	0.04827682	-4.3725255	2.11E-17	2.65E-16	-1	0.14197285	-2.816313	6.64E-07	4.04E-06	-1	2.94080774	1.55621247	0.00499798	0.05510278	0
rplF	0.07584529	-3.7207966	7.06E-24	1.39E-22	-1	0.21066669	-2.2469659	3.53E-08	2.81E-07	-1	2.77758426	1.47383068	0.00025858	0.00570161	1
rpsH	0.05492095	-4.1864997	5.10E-32	1.93E-30	-1	0.25444634	-1.9745667	9.42E-08	7.01E-07	-1	4.63295607	2.211933	8.39E-09	9.25E-07	1
rpsN	0.07623573	-3.7133888	7.01E-27	1.78E-25	-1	0.2414497	-2.0502054	2.47E-08	2.04E-07	-1	3.16714605	1.6631834	2.23E-05	0.00077961	1
yahM	0.88411637	-0.1776918	0.93818377	0.9537302	0	0.60061533	-0.7354868	0.54100254	0.62632702	0	0.67933968	-0.557795	0.55472258	0.92071004	0
rplE	0.07690374	-3.7008024	8.94E-28	2.41E-26	-1	0.30802011	-1.6989036	1.12E-06	6.55E-06	-1	4.00526818	2.00189884	1.64E-07	1.32E-05	1
rplX	0.11298448	-3.1458035	2.73E-21	4.43E-20	-1	0.34338244	-1.5421118	1.27E-05	5.80E-05	-1	3.03920012	1.60369167	2.72E-05	0.00092348	1
rplN	0.09671435	-3.3701262	3.40E-21	5.42E-20	-1	0.37879771	-1.4005005	8.11E-05	0.00029804	-1	3.91666494	1.96962572	2.56E-06	0.00012987	1
rpsQ	0.0270139	-5.2101543	4.38E-29	1.32E-27	-1	0.06391053	-3.9678025	5.19E-15	1.20E-13	-1	2.36583883	1.24235179	0.03075086	0.21423585	0
rpmC	0.02407877	-5.3760946	1.68E-47	1.49E-45	-1	0.06735998	-3.8919646	9.43E-22	6.32E-20	-1	2.79748442	1.4841301	0.00022405	0.00504113	1
rplP	0.00840781	-6.8940535	2.03E-66	6.39E-64	-1	0.05796639	-4.1086395	1.64E-23	1.65E-21	-1	6.89434755	2.78541403	7.66E-13	2.41E-10	1
rpsC	0.00831659	-6.9097923	7.69E-56	1.03E-53	-1	0.04800851	-4.3805661	3.98E-21	2.35E-19	-1	5.7726196	2.52922616	7.09E-10	1.12E-07	1
rplV	0.00523244	-7.5782993	1.24E-65	3.23E-63	-1	0.04656946	-4.4244722	1.09E-22	8.95E-21	-1	8.90013468	3.15382717	2.73E-14	1.33E-11	1
rpsS	0.01044636	-6.5808565	9.60E-59	1.57E-56	-1	0.0590057	-4.0830019	4.79E-21	2.78E-19	-1	5.6484485	2.49785465	6.12E-11	1.23E-08	1
rplB	0.00730947	-7.0960179	1.88E-53	2.38E-51	-1	0.05453605	-4.1966459	3.55E-18	1.40E-16	-1	7.4610151	2.89937193	5.91E-12	1.53E-09	1
yahN	0.27107699	-1.8832254	8.67E-06	3.02E-05	-1	0.2484661	-2.0088791	0.00022165	0.00072501	-1	0.91658869	-0.1256536	1	1	0
rplW	0.0088756	-6.8159395	2.11E-64	5.17E-62	-1	0.06487279	-3.9462426	2.11E-22	1.66E-20	-1	7.30911566	2.86969686	2.04E-12	6.00E-10	1
rplD	0.01482518	-6.075807	6.22E-56	8.59E-54	-1	0.08339121	-3.5839609	7.44E-19	3.26E-17	-1	5.62497277	2.49184611	4.69E-11	9.85E-09	1
rplC	0.00936503	-6.7385009	7.00E-64	1.47E-61	-1	0.07842216	-3.6725948	1.19E-19	5.78E-18	-1	8.37393761	3.06590617	2.12E-15	1.57E-12	1
rpsJ	0.01268919	-6.3002563	1.75E-54	2.27E-52	-1	0.1262925	-2.9851591	8.75E-15	1.95E-13	-1	9.95276342	3.31509715	1.24E-14	6.82E-12	1
gspB	3.55112376	1.82827564	0.00258451	0.00527775	1	3.71290927	1.89255006	0.00253136	0.00622083	1	1.04555896	0.06427442	0.71406496	0.95223057	0
gspA	7.06613172	2.82092064	0.00039324	0.00095805	1	4.69672418	2.23165487	0.00559472	0.0125753	1	0.6646811	-0.5892658	0.7130624	0.95223057	0
gspC	5.15065593	2.36475617	0.00081485	0.00186198	1	3.64899549	1.86749937	0.00458188	0.01052355	1	0.70845258	-0.4972568	0.84444497	0.98077491	0
gspD	4.96520627	2.31185365	1.79E-09	1.05E-08	1	5.40350119	2.4338945	1.30E-08	1.12E-07	1	1.08827326	0.12204085	0.60014307	0.92806272	0
gspE	5.23105492	2.38710192	5.22E-06	1.89E-05	1	7.21469578	2.85093856	3.09E-06	1.61E-05	1	1.37920475	0.46383665	0.39341383	0.86854717	0
gspF	2.74693264	1.45782154	0.00123304	0.00271368	1	3.23657174	1.69446648	0.00026594	0.00084853	1	1.1782494	0.23664495	0.39808728	0.86854717	0
yahO	1.01419373	0.02033326	0.79503363	0.8345208	0	0.11295766	-3.146146	0.00895243	0.01907463	-1	0.1113768	-3.1664793	0.00923737	0.08798446	0
gspG	4.0640716	2.02292582	0.0049824	0.00949818	1	5.00708299	2.32397037	0.00207305	0.00523475	1	1.23203611	0.30104455	0.49478845	0.90539057	0
gspH	5.34743961	2.41884829	5.15E-06	1.87E-05	1	7.3722495	2.8821049	5.00E-07	3.13E-06	1	1.37865035	0.46325661	0.25974314	0.75892335	0
gspI	2.83902104	1.50539354	0.08772897	0.12411879	0	2.20776736	1.14258816	0.17901718	0.2555158	0	0.77765093	-0.3628054	0.94232885	1	0
gspJ	4.31953313	2.11087539	0.01450561	0.0250083	1	3.17084257	1.66486625	0.02033334	0.03914023	1	0.73407067	-0.4460091	1	1	0
gspK	1.92934035	0.94810767	0.07121997	0.10324208	0	1.69712201	0.76309028	0.081593	0.13168718	0	0.87963848	-0.1850174	0.86916707	0.98621446	0
gspL	1.09372786	0.12925382	0.89605171	0.91957723	0	1.36702238	0.45103686	0.23568691	0.32026459	0	1.24987433	0.32178304	0.3109847	0.81168136	0
gspM	3.43079689	1.77854372	0.00288278	0.00583826	1	3.69649728	1.88618885	0.01174092	0.02417666	1	1.07744568	0.10761514	1	1	0
gspO	2.3815951	1.25192816	0.00158373	0.00340239	1	2.44488731	1.28976797	0.00192197	0.0048989	1	1.02657556	0.03783981	0.67495286	0.94091029	0
bfr	1.79516371	0.84411542	0.41039222	0.48351625	0	0.46225912	-1.1132263	0.20558165	0.28577078	0	0.25750249	-1.9573417	0.04260119	0.26648404	0
bfd	5.24833775	2.39186056	6.82E-06	2.41E-05	1	3.03486484	1.60163226	0.03809753	0.06824199	0	0.57825258	-0.7902283	0.08452298	0.41508502	0
caiE	0.96206909	-0.0557876	0.95842734	0.97028961	0	1.50976414	0.59432319	0.28156053	0.37052292	0	1.56928869	0.65011078	0.26900651	0.76983694	0
prpR	5.85457501	2.54956445	3.29E-10	2.14E-09	1	5.47227604	2.45214101	3.58E-07	2.32E-06	1	0.93470082	-0.0974234	0.82408444	0.97661485	0
chiA	1.53366303	0.61698153	0.02563996	0.04174409	0	2.32758249	1.2188323	0.00162721	0.00423722	1	1.51766226	0.60185077	0.14315451	0.57235848	0
tufA	0.03860012	-4.6952508	9.04E-41	6.14E-39	-1	0.06330973	-3.9814289	9.96E-23	8.31E-21	-1	1.64014334	0.71382191	0.0781841	0.39270146	0
fusA	0.03499994	-4.8365038	1.72E-43	1.31E-41	-1	0.11165326	-3.1629028	2.27E-16	6.51E-15	-1	3.19009874	1.67360108	2.78E-06	0.00013943	1
rpsG	0.03844503	-4.7010591	2.31E-40	1.52E-38	-1	0.15735098	-2.6679419	1.97E-12	3.06E-11	-1	4.0928822	2.03311715	2.76E-08	2.76E-06	1
rpsL	0.04228458	-4.5637245	5.51E-38	3.01E-36	-1	0.19143708	-2.3850578	6.05E-11	7.58E-10	-1	4.52734937	2.17866664	2.20E-08	2.26E-06	1
tusB	0.85848787	-0.2201303	0.57844256	0.64276247	0	1.06795296	0.09484811	0.79211039	0.84142688	0	1.24399307	0.31497845	0.86706161	0.98621446	0
tusC	0.85955192	-0.2183433	0.91214486	0.93241005	0	1.10462294	0.14355399	0.94927322	0.9645524	0	1.28511485	0.3618973	0.78871617	0.96411212	0
tusD	0.78900372	-0.341896	0.51290356	0.58179123	0	0.59741413	-0.7431967	0.19136036	0.27008031	0	0.75717531	-0.4013007	0.44959276	0.88755478	0
yheO	0.49007017	-1.0289397	0.00224466	0.00464823	-1	0.43686704	-1.1947338	0.00574345	0.01288341	-1	0.89143771	-0.1657941	0.73039554	0.9551867	0
fkpA	0.19527257	-2.3564388	6.72E-14	6.21E-13	-1	0.23518288	-2.0881451	4.68E-09	4.38E-08	-1	1.20438258	0.26829375	0.83895892	0.98067952	0
prpB	3.98401841	1.99422431	9.76E-06	3.35E-05	1	6.63518437	2.73013655	4.82E-07	3.02E-06	1	1.66545023	0.73591224	0.15713439	0.59893056	0
slyX	0.15757319	-2.665906	5.39E-07	2.25E-06	-1	0.18036735	-2.4709899	9.98E-05	0.00035922	-1	1.14465761	0.19491612	0.75757925	0.95806023	0
slyD	0.23036638	-2.1179979	4.14E-11	2.95E-10	-1	0.28581808	-1.8068309	2.20E-06	1.20E-05	-1	1.24071091	0.31116701	0.38877979	0.86679418	0
yheV	0.32591488	-1.6174329	0.0002349	0.00060462	-1	0.20530672	-2.2841472	8.41E-05	0.00030786	-1	0.62993971	-0.6667143	0.26864308	0.76966758	0
kefB	1.75365183	0.81036234	0.01366021	0.02374998	0	1.64938527	0.72192843	0.02744412	0.05095612	0	0.94054318	-0.0884339	1	1	0
kefG	3.78129955	1.91888214	1.35E-06	5.34E-06	1	2.99348065	1.58182394	0.00060086	0.0017419	1	0.79165393	-0.3370582	0.5443309	0.91914725	0
yheS	0.9010375	-0.1503409	0.8456447	0.87681048	0	1.2185994	0.28522393	0.351186	0.44200824	0	1.35244026	0.43556487	0.23187249	0.72214524	0
yheT	1.21489532	0.28083201	0.51476024	0.5831485	0	1.85218057	0.88922475	0.04761548	0.0824079	0	1.52455981	0.60839275	0.14572454	0.57739913	0
yheU	0.85093637	-0.2328768	0.60734985	0.66910672	0	0.72049304	-0.4729436	0.36227886	0.45415894	0	0.84670613	-0.2400668	0.59825821	0.92806272	0
prkB	0.66579104	-0.5868586	0.14104675	0.18999802	0	0.73867704	-0.4369844	0.39454189	0.48537879	0	1.10947278	0.14987428	0.64584564	0.93208609	0
yhfA	0.37631571	-1.4099846	1.19E-05	4.02E-05	-1	0.42656751	-1.229154	0.00072097	0.00205148	-1	1.13353628	0.18083057	0.91645451	1	0
prpC	5.61393998	2.48901364	0.0002714	0.00068581	1	5.16675859	2.36925948	0.00098049	0.00271988	1	0.92034447	-0.1197542	1	1	0
crp	0.07563705	-3.724763	6.39E-29	1.89E-27	-1	0.11201407	-3.1582482	4.53E-16	1.21E-14	-1	1.4809417	0.56651485	0.1537355	0.59217223	0
yhfK	0.56313452	-0.8284485	0.01070435	0.01901896	0	0.82819047	-0.2719655	0.3393813	0.42984407	0	1.47067962	0.556483	0.21885239	0.70102268	0
argD	3.6446346	1.86577418	6.99E-05	0.00020285	1	3.30333591	1.72392368	0.00066587	0.00191071	1	0.90635586	-0.1418505	0.98769128	1	0
pabA	6.27375321	2.64932878	8.82E-10	5.42E-09	1	4.42444267	2.14549574	3.06E-06	1.60E-05	1	0.70523059	-0.503833	0.46209636	0.88911212	0

fic	4.21181661	2.07444262	6.53E-06	2.32E-05	1	1.00687236	0.0098808	0.83210079	0.87278742	0	0.23905893	-2.0645618	0.0003751	0.00758803	-1
yhfG	3.08600333	1.62573962	0.00045759	0.00109786	1	1.09721408	0.13384504	1	1	0	0.35554533	-1.4918946	0.00430573	0.04906526	-1
ppiA	0.36314611	-1.461378	1.52E-05	5.00E-05	-1	0.45640973	-1.1315985	0.00238242	0.00593455	-1	1.25682121	0.32977944	0.58909517	0.92806272	0
tsgA	0.89726606	-0.1563923	0.72462763	0.77348484	0	0.93932416	-0.090305	0.80240368	0.84889199	0	1.04687361	0.06608727	0.59579268	0.92806272	0
nirB	0.06799077	-3.8785173	9.40E-31	3.30E-29	-1	0.13104932	-2.9318183	2.12E-14	4.44E-13	-1	1.92745754	0.94669908	0.01070335	0.09813255	0
nirD	0.08838976	-3.4999769	9.22E-25	1.99E-23	-1	0.24855106	-2.0083858	3.78E-08	3.01E-07	-1	2.8119892	1.49159105	0.00018146	0.00425664	1
prpD	5.37661117	2.42669714	1.34E-07	6.10E-07	1	5.57829834	2.47982509	4.17E-07	2.66E-06	1	1.03751195	0.05312795	0.65628701	0.93664426	0
nirC	0.16050325	-2.6393256	5.11E-14	4.76E-13	-1	0.58829129	-0.7653974	0.02282855	0.04331445	0	3.66529203	1.87392815	5.30E-06	0.00025141	1
cysG	0.22255832	-2.1677447	1.63E-09	9.54E-09	-1	0.77312436	-0.3712276	0.23359902	0.31821026	0	3.47380572	1.79651707	1.88E-05	0.00069005	1
yhfL	1.05383268	0.07564582	0.82483245	0.85957919	0	2.30723687	1.20616613	0.32610914	0.41625542	0	2.18937686	1.13052031	0.24702458	0.74758115	0
frlA	4.08351335	2.02981094	0.0001575	0.00042187	1	3.86909018	1.95199436	0.00112836	0.00308171	1	0.94749052	-0.0778166	1	1	0
frlB	1.7380903	0.79750304	0.09584518	0.13443892	0	1.99510655	0.9964658	0.01893486	0.03676526	0	1.14787278	0.19896276	0.3951799	0.86854717	0
frlC	2.32411093	1.21667893	0.02768929	0.04468527	1	2.8705374	1.52132085	0.01076115	0.02240976	1	1.23511204	0.30464192	0.47092691	0.89609045	0
frlD	1.51450724	0.59884848	0.10194265	0.14213406	0	2.57908586	1.3668598	0.00339575	0.00809375	1	1.70292078	0.76801132	0.0954419	0.45064109	0
frlR	0.46609176	-1.1013141	0.00580892	0.01092294	-1	0.4819661	-1.0529964	0.03418112	0.06200142	0	1.0340584	0.04831767	0.86893247	0.98621446	0
yhfS	2.23583754	1.16081536	0.0044007	0.00848816	1	2.95602568	1.5636588	0.0015835	0.00414053	1	1.32211112	0.40284344	0.37944572	0.85988523	0
yhfT	2.60393163	1.38069157	0.03901541	0.06105885	0	2.80030343	1.48558316	0.01617031	0.03185761	1	1.07541358	0.10489159	0.58246412	0.92806272	0
prpE	4.35766035	2.12355375	9.16E-06	3.17E-05	1	5.24434345	2.39076217	2.26E-06	1.23E-05	1	1.20347687	0.26720842	0.37341121	0.85857675	0
yhfU	1.72009419	0.78248757	0.23355167	0.29601083	0	1.81417377	0.85931265	0.27500164	0.36329812	0	1.05469443	0.07682508	0.90170818	0.99737474	0
php	2.43493825	1.28388519	0.0189908	0.03170157	1	2.84660986	1.50924478	0.02636981	0.04914749	1	1.1690686	0.22535959	0.71331939	0.95223057	0
yhfW	5.63052351	2.49326907	2.18E-08	1.10E-07	1	7.51569517	2.90990655	9.26E-09	8.31E-08	1	1.33481285	0.41663749	0.31030179	0.81168136	0
yhfX	6.44835088	2.68893025	1.20E-05	4.03E-05	1	7.29929852	2.86775782	1.10E-05	5.08E-05	1	1.13196361	0.17882758	0.55180315	0.92038542	0
yhfY	0.52814241	-0.9210011	0.552825	0.61899219	0	0.68743256	-0.5407099	0.81798259	0.86115568	0	1.30160456	0.38029121	0.62983914	0.93109012	0
yhfZ	0.94714779	-0.0783385	0.92343221	0.94263926	0	1.13370571	0.18104619	0.47355083	0.56283516	0	1.19696811	0.25938472	0.41521529	0.87131399	0
trpS	0.24204819	-2.0466338	6.19E-10	3.87E-09	-1	0.27806526	-1.8465046	2.91E-06	1.54E-05	-1	1.14880124	0.20012922	0.66799237	0.93878679	0
gph	0.28347663	-1.8186881	7.06E-07	2.91E-06	-1	0.31904128	-1.648185	5.62E-05	0.00021553	-1	1.12545092	0.17050315	0.93747044	1	0
rpe	0.19298534	-2.3734368	6.74E-11	4.67E-10	-1	0.27723807	-1.8508027	7.51E-06	3.63E-05	-1	1.43657579	0.52263411	0.27684921	0.77913531	0
dam	0.41678087	-1.262639	0.00048003	0.00114798	-1	0.56113293	-0.8335855	0.0533769	0.0910002	0	1.34635001	0.42905352	0.32649146	0.82653693	0
codB	3.01054515	1.59002475	3.76E-05	0.00011536	1	3.17718054	1.66774707	4.34E-05	0.00017191	1	1.05535057	0.07772232	0.58265928	0.92806272	0
damX	0.37208721	-1.4262873	7.45E-06	2.62E-05	-1	0.51648292	-0.9532075	0.00722835	0.01580508	0	1.38806953	0.47307984	0.27538571	0.77762465	0
aroB	0.40488868	-1.3044028	8.24E-05	0.00023533	-1	0.48035216	-1.0578356	0.00495316	0.01127086	-1	1.1863808	0.24656715	0.62316338	0.92989404	0
aroK	0.22981815	-2.1214354	4.39E-09	2.47E-08	-1	0.45139256	-1.1475455	0.00247809	0.0061177	-1	1.96412927	0.97388989	0.03615762	0.23885718	0
hofQ	7.52471258	2.91163648	1.19E-13	1.07E-12	1	6.46643318	2.69297016	1.33E-09	1.38E-08	1	0.85935949	-0.2186663	0.69957907	0.94869712	0
hofP	5.74623002	2.52261575	7.90E-05	0.00022682	1	5.56085375	2.4753064	9.80E-05	0.00035357	1	0.9677395	-0.0473093	0.70047672	0.94869712	0
hofO	5.6403538	2.49578566	0.00133698	0.00291771	1	6.60699899	2.72399512	0.00138677	0.003685	1	1.17138024	0.22820946	0.67110172	0.93979017	0
hofN	2.88423024	1.52818634	0.00453758	0.00871792	1	4.68051906	2.22666853	0.00047066	0.00139307	1	1.62279661	0.69848219	0.23134739	0.72101907	0
hofM	4.1654806	2.05848296	1.31E-06	5.20E-06	1	6.07407426	2.60266455	2.83E-07	1.89E-06	1	1.45819291	0.54418159	0.24872647	0.74826869	0
mrcA	2.06198004	1.04403037	0.00368621	0.00725578	1	3.07008314	1.61827772	5.78E-05	0.00022079	1	1.48890051	0.57424736	0.10474421	0.47522837	0
nudE	0.97709243	-0.033433	0.96529267	0.97589785	0	1.34139403	0.42373309	0.65295727	0.72772211	0	1.37284252	0.45716614	0.57486891	0.92806272	0
codA	0.47728733	-1.0670701	0.01222281	0.02144088	-1	0.55258065	-0.8557431	0.05633777	0.09513053	0	1.15775261	0.211327	0.78263783	0.9635071	0
yrfF	1.26390524	0.33788831	0.31265231	0.38117175	0	1.14322436	0.19310857	0.69119257	0.76074221	0	0.90451746	-0.1447797	0.64855102	0.93327989	0
yrfG	1.01901047	0.02716888	0.80931484	0.84629348	0	0.90894404	-0.1377366	0.74243178	0.80156512	0	0.89198695	-0.1649055	0.79873013	0.96774826	0
hslR	1.22074035	0.28775637	0.61052755	0.67182366	0	0.87323731	-0.1955543	0.8574942	0.89264238	0	0.71533419	-0.4833107	0.70400804	0.94896888	0
hslO	0.50212648	-0.9938773	0.00566014	0.0106659	0	0.51869037	-0.9470545	0.06634374	0.11038139	0	1.03298749	0.04682278	0.64448543	0.93208609	0
yhgE	4.44221999	2.15128084	4.84E-05	0.000146	1	4.21053895	2.07400491	0.00586948	0.01314609	1	0.94784566	-0.0772759	0.55809206	0.92317654	0
pck	0.04118509	-4.601734	1.08E-39	6.51E-38	-1	0.07495723	-3.7377886	1.69E-20	9.01E-19	-1	1.82000883	0.86394545	0.01696017	0.13774283	0
envZ	0.34283518	-1.5444129	3.68E-06	1.37E-05	-1	0.39252923	-1.349128	0.0010498	0.00289513	-1	1.14495025	0.19528492	0.54290237	0.91914725	0
ompR	0.09079223	-3.4612874	1.05E-22	1.89E-21	-1	0.09047544	-3.46633	1.87E-17	6.44E-16	-1	0.9965108	-0.0050427	0.59573785	0.92806272	0
greB	0.90859032	-0.1382982	0.66049907	0.71593236	0	0.64869577	-0.6243861	0.36528025	0.45688463	0	0.71395848	-0.4860879	0.52855753	0.91626596	0
yhgF	0.49564601	-1.012618	0.00272246	0.00554153	-1	0.74762724	-0.419609	0.45369794	0.54288957	0	1.50838949	0.593009	0.06515616	0.35126976	0
cynR	1.65122105	0.72353327	0.15416748	0.20560318	0	1.42588027	0.51185285	0.15136581	0.22177902	0	0.86353082	-0.2116804	0.90607951	0.99895266	0
feoA	0.0551042	-4.1816939	5.56E-08	2.67E-07	-1	0.07986629	-3.6462695	4.71E-05	0.00018501	-1	1.44936847	0.53542442	0.62950907	0.93109012	0
feoB	0.06717774	-3.895873	4.02E-13	3.47E-12	-1	0.15503002	-2.6893805	9.22E-06	4.37E-05	-1	2.30775886	1.20649249	0.03769206	0.24408514	0
feoC	0.03444027	-4.8597598	3.82E-23	7.05E-22	-1	0.3095877	-1.6915799	7.42E-05	0.0002757	-1	8.98911978	3.16817985	3.66E-08	3.50E-06	1
yhgA	1.40881299	0.49448011	0.54321767	0.61037712	0	0.92769789	-0.1082732	0.87292932	0.90466104	0	0.65849605	-0.6027533	0.63622908	0.93163024	0
bioH	0.69997372	-0.5146273	0.2689778	0.33570923	0	0.9870391	-0.0188209	0.57462275	0.65835931	0	1.4101088	0.49580648	0.11232097	0.4958313	0
gntX	0.41545664	-1.2672302	0.00260181	0.00530571	-1	0.52123538	-0.9399931	0.04384806	0.07698235	0	1.25460837	0.3272371	0.52914029	0.91648924	0
nfuA	0.29971424	-1.7383405	3.19E-07	1.37E-06	-1	0.27235191	-1.8764561	2.12E-06	1.16E-05	-1	0.90870529	-0.1381156	0.51064957	0.90925684	0
gntT	1.68867512	0.75589179	0.11739203	0.16118141	0	1.42329686	0.5092366	0.2172703	0.29966213	0	0.84284825	-0.2466552	0.85444063	0.98236299	0
malQ	0.22115274	-2.176885	3.74E-11	2.69E-10	-1	0.12533951	-2.9960868	1.07E-12	1.71E-11	-1	0.56675542	-0.8192018	0.05324545	0.30937079	0
malP	0.31353437	-1.6733045	5.03E-08	2.44E-07	-1	0.10113027	-3.3057133	1.02E-15	2.60E-14	-1	0.32254922	-1.6324088	2.62E-05	0.00089618	-1
cynT	2.69146697	1.42839272	0.00401328	0.00783671	1	2.87678061	1.5244552	0.00395532	0.00924792	1	1.06885228	0.09606248	0.66020932	0.93664426	0
malT	0.06776925	-3.8832254	1.66E-30	5.66E-29	-1	0.07449098	-3.7467904	2.39E-21	1.47E-19	-1	1.09918555	0.13643494	1	1	0
rtcA	1.01070818	0.01536652	0.93747522	0.95340358	0	0.72520566	-0.4635379	0.64509287	0.72205304	0	0.7175223	-0.4789044	0.61491596	0.92897598	0
rtcB	1.74169631	0.80049309	0.02831653	0.04555004	0	1.19077249	0.2518978	0.36579321	0.45726739	0	0.68368549	-0.5485953	0.37803977	0.85988523	0
rtcR	2.58967786	1.37277265	0.02045387	0.03397697	1	3.5662235	1.83439712	0.00622941	0.01383304	1	1.37709155	0.46162448	0.42286174	0.87468118	0
glpR	0.99744941	-0.0036844	0.93760677	0.95340358	0	1.21426205	0.2800798	0.43826678	0.5282872	0	1.21736705	0.28376422	0.41313573	0.87131399	0
glpG	1.01539375	0.02203928	0.92559337	0.94397273	0	1.55240435	0.63450438	0.22524644	0.30921529	0	1.52886932	0.6124651	0.19091437	0.66608574	0
glpE	1.12191674	0.16596561	0.87707735	0.90409583	0	0.98518898	-0.0215276	0.86306918	0.89629718	0	0.87813021	-0.1874932	0.69639619	0.94850102	0
glpD	0.40529136	-1.3029687	1.35E-05	4.49E-05	-1	0.18493964	-2.4348736	9.39E-11	1.15E-09	-1	0.45631281	-1.1319049	0.00100906	0.01678931	-1
yzgL	1.89142692	0.91947504	0.01845622	0.03093781	0	1.11959941	0.16298264	1	1	0	0.59193374	-0.7564924	0.03292336	0.22406179	0
glgP	1.28948029	0.36678972	0.42034442	0.49287531	0	0.57982728	-0.7863049	0.04408774	0.07730577	0	0.44965967	-1.1530946	0.00421109	0.04823614	-1
cynS	2.22803861	1.15577423	0.07290851	0.10537857	0	2.2260661	1.15449643	0.09909816	0.1555095	0	0.99911469	-0.0012778	0.92544457	1	0
glgA	1.69942568	0.76504727	0.02754965	0.04451739	0	0.81422097	-0.2965077	0.32719558	0.41703988	0	0.47911538	-1.061555	0.00336759	0.04125292	-1
glgC	0.84160555	-0.2487839	0.28165149	0.34867002	0	0.38017526	-1.3952634	0.00034173	0.0010575	-1	0.45172618	-1.1464796	0.00334828	0.04113062	-1
glgX	0.61018865	-0.7126728	0.0136682	0.02374998	0	0.40762629	-1.294681	0.00046139	0.00137205	-1	0.66803323	-0.5820082	0.09987519	0.45975948	0
glgB	0.51468074	-0.9582503	0.00097786	0.0022003	0	0.34418131	-1.5387593	5.08E-05	0.00019739	-1	0.66872778	-0.580509	0.12651874	0.5319293	0
asd	0.27451107	-1.8650638	4.81E-09	2.69E-08	-1	0.21908907	-2.1904105	1.50E-08	1.29E-07	-1	0.79810653	-0.3253468	0.32917738	0.83053586	0
yhgN	1.90761815	0.93177241	0.01801556	0.03028942	0	1.74649129	0.80445945	0.05379385	0.09153447	0	0.91553506	-0.127313	0.96468927	1	0
gntU	3.1269884	1.64477387	0.00010921	0.00030187	1	4.02757519	2.00991152	6.19E-05	0.00023444	1	1.28800452	0.36513766	0.4071525	0.86959974	0
gntK	1.65891059	0.73023613	0.15565408	0.20727286	0	1.24400207	0.31498889	0.30780837	0.39818652	0	0.74989097	-0.4152472	0.82916413	0.97848911	0
gntR	0.51670149	-0.9525971	0.00841969	0.01530443	0	0.73804857	-0.4382123	0.2631894	0.35020595	0	1.42838485	0.51438474	0.2361917	0.7289051	0
yhhW	0.56709929	-0.8183268	0.04737008	0.07252466	0	0.47119507	-1.0856036	0.0416107	0.07363943	0	0.83088638	-0.2672769	0.73209797	0.95562813	0
caiD	3.30645753	1.72528637	8.23E-05	0.00023522	1	7.99925365	2.9998654	5.09E-07	3.17E-06	1	2.41928214	1.27457903	0.04688938	0.28561071	0
cynX	4.34950209	2.12085026	0.00290558	0.00587776	1	5.48386369	2.45519271	0.00111558	0.00305247	1	1.26080263	0.33434245	0.47382733	0.89828993	0
yhhX	0.59098413	-0.7588087	0.0148923	0.02558511	0	0.25882704	-1.9499397	3.28E-06	1.70E-05	-1	0.43795938	-1.191131	0.00410084	0.04746647	-1
ryhB	0.91552283	-0.1273322	0.75173691	0.79837301	0	1.21597409	0.28211249	0.80774685	0.85294982	0	1.32817452	0.40944472	0.67790132	0.94128448	0
yhhY	0.41808659	-1.2581263	0.0280119	0.04512348	-1	0.68563348	-0.5444905	0.48202176	0.57084888	0	1.63993176	0.71363578	0.18227303	0.65078561	0
yhhZ	4.08449267	2.03015689	0.00143416	0.00311134	1	2.75061006	1.45975163	0.01796827	0.03508696	1	0.67342759	-0.5704053	0.634793	0.93163024	0
yrhA	5.02050235	2.32783173	0.15170773	0.2027513	0	1.96626415	0.97545715	0.26259877	0.34965412	0	0.39164689	-1.3523746	0.72557763	0.95459348	0
yrhB	4.29158045	2.10150904	0.01805485	0.03033407	1	3.07049646	1.61847194	0.10803373	0.16657399	0	0.71546986	-0.4830371	0.63609076	0.93163024	0
ggt	2.2635046	1.17855824	0.00220989	0.00458698	1	1.75421461	0.81082526	0.01198411	0.02456297	0	0.77499936	-0.367733	0.78336945	0.9635071	0
yhhA	0.83649556	-0.2575702	0.56987596	0.63500053	0	0.32728807	-1.6113671	0.01607389	0.03172422	-1	0.39126098	-1.3537969	0.0374065	0.2433078	0
lacA	5.28876553	2.40293102	3.96E-05	0.00012094	1	4.3671441	2.12669014	0.00034135	0.00105705	1	0.82573978	-0.2762409	0.90756473	0.99909148	0
ugpQ	2.76435726	1.46694408	0.07333224	0.10595633	0	0.98249414	-0.0254793	0.87722186	0.90825759	0	0.35541504	-1.4924234	0.15333624	0.59175389	0
ugpC	4.61762008	2.20714948	4.72E-09	2.64E-08	1	1.94036801	0.9563303	0.00678353	0.01494339	0	0.42020954	-1.2508192	0.01406346	0.12019352	0
ugpE	5.81655355	2.54016458	1.14E-07	5.24E-07	1	5.03614805	2.3323207	1.02E-05	4.75E-05	1	0.86583026	-0.2078439	0.85307292	0.98236299	0
ugpA	8.43477998	3.07635044	5.07E-12	3.95E-11	1	7.25299808	2.85857747	1.59E-09	1.62E-08	1	0.85989179	-0.217773	0.93611245	1	0
ugpB	4.04006425	2.01437824	1.85E-07	8.25E-07	1	2.36820628	1.24379475	0.0042919	0.0099298	1	0.58618035	-0.7705835	0.08720633	0.42308413	0
livF	2.04693837	1.03346767	0.03753169	0.05895358	0	1.98030164	0.9857202	0.02726739	0.05069188	0	0.96744565	-0.0477475	0.68905145	0.94487465	0
livG	2.2626795	1.17803224	0.00687889	0.012745	1	5.34601285	2.41846331	1.67E-05	7.36E-05	1	2.36269116	1.24043106	0.02685298	0.19413385	0
livM	4.79108011	2.26035094	1.11E-06	4.45E-06	1	7.03863813	2.81529632	1.73E-08	1.46E-07	1	1.46911301	0.55494538	0.14145497	0.56813883	0

livH	5.26646746	2.39683558	4.58E-05	0.00013854	1	7.20905803	2.84981076	9.75E-06	4.59E-05	1	1.36886026	0.45297518	0.33966297	0.83879867	0
livK	3.47999668	1.79908593	0.00212436	0.00443235	1	4.08535353	2.03046093	0.00200824	0.00509903	1	1.17395328	0.231375	0.5966305	0.92806272	0
lacY	6.93806861	2.79453411	1.99E-12	1.61E-11	1	5.86857309	2.55300976	2.27E-08	1.88E-07	1	0.84585112	-0.2415243	0.58496582	0.92806272	0
panM	0.39371968	-1.3447593	0.00115956	0.00256474	-1	0.62530937	-0.677358	0.13785057	0.20427956	0	1.58820959	0.66740132	0.19429524	0.67203294	0
livJ	1.91988026	0.94101634	0.05434439	0.08179495	0	2.81829815	1.49482424	0.00466503	0.01069231	1	1.46795517	0.55380791	0.21885473	0.70102268	0
rpoH	0.32937047	-1.6022169	5.44E-07	2.27E-06	-1	0.39405768	-1.3435213	0.00011658	0.00041148	-1	1.19639651	0.2586956	0.744142	0.95683847	0
ftsX	2.20455295	1.14048613	0.03057266	0.04882119	1	1.98136783	0.98649673	0.30441069	0.39448412	0	0.89876173	-0.1539894	0.47698369	0.89855925	0
ftsE	0.6027331	-0.7304088	0.06431337	0.09465917	0	0.74151928	-0.4314439	0.4388887	0.52886252	0	1.23026142	0.29896491	0.3923561	0.86854717	0
ftsY	0.52016535	-0.9429578	0.00864628	0.01566474	0	0.74696759	-0.4208825	0.25499677	0.34094727	0	1.43601949	0.52207533	0.24100612	0.73370247	0
rsmD	0.71181345	-0.4904289	0.36410517	0.43513326	0	0.78917203	-0.3415883	0.73388695	0.79583961	0	1.10867816	0.14884063	0.62454581	0.92989404	0
yhhL	1.26680341	0.34119266	0.56683181	0.63208687	0	1.03114587	0.04424844	0.89727743	0.92146618	0	0.81397466	-0.2969442	0.71167072	0.95223057	0
yhhM	0.514422	-0.9589758	0.11057329	0.15276891	0	0.42875565	-1.2217724	0.10794221	0.16654911	0	0.83347068	-0.2627967	0.85943917	0.98424873	0
yhhN	0.57735041	-0.7924809	0.03202855	0.05096185	0	0.5268449	-0.9245498	0.03651068	0.06577281	0	0.91252191	-0.1320689	0.77466446	0.96287211	0
lacZ	72.5780592	6.18146157	1.74E-26	4.32E-25	1	50.7947303	5.66660693	1.53E-19	7.13E-18	1	0.69986344	-0.5148546	0.29669239	0.80055472	0
zntA	0.3657059	-1.4512442	1.22E-05	4.10E-05	-1	0.37896118	-1.399878	0.00078243	0.00220508	-1	1.03624573	0.05136615	0.72260901	0.95399541	0
tusA	1.01781766	0.02547913	0.92900334	0.94679459	0	1.19738848	0.25989129	0.79271645	0.84146384	0	1.17642729	0.23441216	0.8245063	0.97661485	0
yhhQ	1.32556531	0.40660775	0.19476089	0.25215448	0	1.28801524	0.36514966	0.28549061	0.37524777	0	0.97167241	-0.0414581	1	1	0
dcrB	0.19284987	-2.3744499	1.51E-12	1.24E-11	-1	0.20837565	-2.2627414	1.03E-08	9.10E-08	-1	1.0805071	0.11170855	0.86463887	0.98554082	0
yhhS	1.37166429	0.45592743	0.14448037	0.19409057	0	1.4611693	0.54712334	0.06141987	0.10312097	0	1.06525285	0.09119592	0.51375934	0.90937129	0
yhhT	242.422447	7.92137948	4.43E-68	1.78E-65	1	45.4484679	5.50615976	5.51E-29	9.37E-27	1	0.18747632	-2.4152197	4.31E-10	7.31E-08	-1
acpT	6.9868074	2.80463337	9.46E-15	9.48E-14	1	2.82850561	1.50004003	0.00274959	0.00669813	1	0.40483521	-1.3045933	0.0003331	0.00699324	-1
nikA	0.12150109	-3.0409589	8.65E-17	1.04E-15	-1	0.19159779	-2.3838472	9.39E-09	8.42E-08	-1	1.57692241	0.65711167	0.10794893	0.48409953	0
nikB	0.15520749	-2.6877299	7.63E-12	5.85E-11	-1	0.45189301	-1.1459469	0.01519926	0.03026837	-1	2.9115412	1.54178303	0.00011972	0.00306958	1
nikC	0.26492558	-1.916341	1.41E-05	4.67E-05	-1	1.0713657	0.09945101	0.84077891	0.87959328	0	4.04402515	2.01579197	2.32E-05	0.00080586	1
lacI	2.7456854	1.45716633	2.61E-05	8.22E-05	1	3.87355483	1.95365816	9.36E-06	4.43E-05	1	1.41077883	0.49649183	0.28300193	0.78651746	0
nikD	0.09564517	-3.3861641	3.33E-15	3.45E-14	-1	0.42785547	-1.2248046	0.00998174	0.02103448	-1	4.47336214	2.16135956	1.76E-06	9.44E-05	1
nikE	0.15493796	-2.6902374	2.77E-10	1.82E-09	-1	0.32006778	-1.6435507	0.00027153	0.00086384	-1	2.0657802	1.04668676	0.05411236	0.3120921	0
nikR	0.52566155	-0.9277939	0.01224326	0.02146823	0	0.58823599	-0.765533	0.0617586	0.10361095	0	1.1190394	0.16226084	0.91537114	1	0
rhsB	2.1206941	1.08453653	0.04622695	0.07097383	0	2.58257844	1.36881217	0.09671173	0.15225121	0	1.21779866	0.28427563	0.87622426	0.98928545	0
yhhH	2.74452357	1.45655573	0.24390042	0.30762655	0	1.06924465	0.09659198	0.64715475	0.72364293	0	0.38959208	-1.3599637	0.50446595	0.90701715	0
yrhC	2.15999436	1.11102755	0.57902459	0.64324739	0	1.67676417	0.74567979	0.60635073	0.68651393	0	0.77628173	-0.3653478	1	1	0
yhhI	1.75575135	0.81208855	0.54996441	0.61638995	0	3.80868973	1.92929476	0.18055008	0.2568985	0	2.16926487	1.11720622	0.40166711	0.86854717	0
yhhJ	1.02909514	0.04137636	0.69106192	0.74304297	0	1.16189504	0.21647975	0.36635355	0.45770888	0	1.12904532	0.17510339	0.51654764	0.90937129	0
rbbA	0.48038525	-1.0577363	0.00102657	0.00229821	-1	0.51579803	-0.9551218	0.04287747	0.07560875	0	1.07371747	0.10261442	0.4588685	0.88755478	0
yhiI	0.29791739	-1.7470158	2.14E-07	9.42E-07	-1	0.31312317	-1.6751978	3.13E-05	0.00012857	-1	1.05104025	0.07181792	0.90118973	0.99705135	0
mhpR	3.2478223	1.6994727	8.57E-06	2.99E-05	1	2.21639661	1.14821607	0.0086358	0.01843555	1	0.68242546	-0.5512566	0.25184451	0.74992188	0
yhiJ	87.3419968	6.44860361	4.15E-25	9.30E-24	1	32.0002665	5.00001201	5.72E-12	8.29E-11	1	0.36637862	-1.4485916	0.00297146	0.03766955	-1
yhiL	4.33806558	2.11705186	9.73E-05	0.00027244	1	3.68356076	1.88110104	0.00120702	0.00326481	1	0.84912519	-0.2359508	0.88896821	0.99411016	0
yhiM	5.98829529	2.58214537	4.59E-10	2.93E-09	1	4.97562158	2.31487677	2.83E-06	1.51E-05	1	0.83089115	-0.2672686	0.4771637	0.89855925	0
yhiN	0.46658445	-1.0997899	0.00922067	0.01662355	-1	0.79486189	-0.3312239	0.66750212	0.74075638	0	1.7035756	0.76856597	0.05300058	0.30917007	0
pitA	0.75832491	-0.399112	0.27791846	0.34462825	0	0.90327294	-0.1467661	0.66458071	0.73825595	0	1.19114239	0.25234588	0.59298892	0.92806272	0
uspB	8.24704469	3.04387722	5.93E-09	3.28E-08	1	2.03313092	1.02370312	0.3232536	0.41369481	0	0.24652842	-2.0201741	0.00011952	0.00306958	-1
uspA	0.07192074	-3.7974483	3.00E-30	9.73E-29	-1	0.06534303	-3.9358229	5.08E-23	4.49E-21	-1	0.90854218	-0.1383746	0.41501893	0.87131399	0
dtpB	0.12615985	-2.9866752	1.57E-18	2.16E-17	-1	0.11122375	-3.1684632	1.18E-14	2.54E-13	-1	0.88160969	-0.181788	0.61694701	0.92897598	0
rsmJ	1.71245818	0.77606876	0.03321737	0.05264483	0	1.52061821	0.60465798	0.13660664	0.20264007	0	0.88797392	-0.1714108	0.77007552	0.96177657	0
prlC	0.80008365	-0.3217772	0.2201629	0.28127713	0	0.3984876	-1.3273933	0.00155267	0.00406713	-1	0.49805741	-1.005616	0.01890682	0.14836137	0
mhpA	8.75570344	3.13022309	4.27E-06	1.57E-05	1	10.9964129	3.45896107	1.84E-06	1.02E-05	1	1.25591426	0.32873798	0.44115667	0.88087171	0
rlmJ	0.52861033	-0.9197235	0.01385823	0.0240329	0	1.02554962	0.03639729	0.90220509	0.92523655	0	1.94008622	0.95612077	0.02148706	0.16422516	0
gor	0.37637732	-1.4097484	1.60E-05	5.24E-05	-1	0.30078364	-1.733202	2.19E-05	9.33E-05	-1	0.79915452	-0.3234536	0.45212942	0.88755478	0
dinQ	0.15401488	-2.6988583	2.69E-11	1.96E-10	-1	0.28416503	-1.8151991	1.89E-06	1.05E-05	-1	1.84504917	0.88365926	0.41733351	0.872394	0
agrA	0.88192834	-0.1812667	0.53049564	0.59790744	0	0.48826344	-1.0342683	0.52645521	0.61334183	0	0.55363165	-0.8530017	0.74646218	0.95683847	0
agrB	4.70447931	2.23403505	0.0610038	0.09033433	0	4.32968724	2.11426281	0.09594126	0.1512538	0	0.92033293	-0.1197722	1	1	0
arsR	5.31355285	2.40967682	0.0003681	0.00090126	1	3.87500046	1.95419648	0.03545988	0.06409029	0	0.72926732	-0.4554803	0.38614726	0.86529949	0
arsB	2.75397315	1.46151449	0.0013036	0.00285333	1	3.60836321	1.85134456	0.00043028	0.00128997	1	1.31023907	0.38983007	0.37763569	0.85988523	0
arsC	0.97596327	-0.0351012	0.91479972	0.93490754	0	0.92590729	-0.1110604	0.84549552	0.88348184	0	0.9487112	-0.0759591	0.81699574	0.97298169	0
yhiS	1.70446792	0.76932145	0.34237209	0.41183483	0	2.15945981	1.11067047	0.1184955	0.17952536	0	1.26694072	0.34134902	0.49716376	0.90539057	0
mhpB	10.1977728	3.3501822	0.00158897	0.003412	1	11.4847684	3.52164986	0.00089462	0.00249737	1	1.1262036	0.17146767	0.60361006	0.92806272	0
slp	3.60078785	1.8483126	0.14215623	0.19129455	0	0.71503664	-0.4839109	0.69460204	0.76373387	0	0.19857783	-2.3322235	0.09754903	0.45619431	0
dctR	6.25830479	2.64577192	0.00227816	0.00471318	1	2.35690629	1.2368944	0.08371992	0.13477534	0	0.37660459	-1.4088775	0.2756071	0.77762465	0
yhiD	14.7180992	3.87951946	5.78E-17	7.05E-16	1	4.45904332	2.15673422	1.84E-05	7.99E-05	1	0.30296326	-1.7227852	0.00089334	0.01526996	-1
hdeB	2.45406763	1.29517501	0.29837288	0.36618866	0	0.40266166	-1.31236	0.2375965	0.32218674	0	0.16407928	-2.607535	0.03695619	0.24108994	0
hdeA	1.80492535	0.85193917	0.44591172	0.51627577	0	0.40710189	-1.2965382	0.12890171	0.19282893	0	0.22555054	-2.1484773	0.02644352	0.19243551	0
hdeD	3.26198489	1.7057501	0.03217984	0.05118414	0	1.31746149	0.39776079	0.54576667	0.63035622	0	0.40388338	-1.3079893	0.1700514	0.62286269	0
arrS	5.37097895	2.42518507	0.00077854	0.00179182	1	1.17687527	0.23496143	0.71597757	0.78140403	0	0.21911746	-2.1902236	0.0035971	0.04322407	-1
gadE	1.32393627	0.40483368	0.38628889	0.45780468	0	0.7891763	-0.3415805	0.12350026	0.185804	0	0.5960833	-0.7464141	0.02279379	0.17212435	0
mdtE	2.93593751	1.55382126	0.00012495	0.00033986	1	2.77674106	1.47339265	0.00113644	0.00310188	1	0.94577662	-0.0804286	0.98637291	1	0
mdtF	4.47537667	2.16200911	6.92E-11	4.79E-10	1	3.56789291	1.83507231	8.57E-07	5.12E-06	1	0.7972274	-0.3269368	0.47923592	0.89894957	0
mhpC	9.44843767	3.24007579	2.07E-10	1.37E-09	1	9.10347912	3.18641801	7.06E-09	6.47E-08	1	0.96349041	-0.0536578	0.83652072	0.98025477	0
gadW	13.4162499	3.74590956	4.29E-25	9.58E-24	1	1.36960011	0.45375472	0.45597034	0.545461	0	0.10208517	-3.2921548	6.43E-15	4.05E-12	-1
gadY	9.76320796	3.28735526	5.59E-06	2.00E-05	1	2.69349685	1.42948038	0.13294845	0.19847946	0	0.27588236	-1.8578749	0.01863973	0.14705045	0
gadX	4.29373818	2.10223422	0.00104892	0.00234229	1	1.77936892	0.83136565	0.66669358	0.74023088	0	0.4144102	-1.2708686	0.0219363	0.16707962	0
gadA	3.40966415	1.76962964	5.54E-05	0.00016481	1	0.64632327	-0.6296722	0.20268849	0.28228189	0	0.18955629	-2.3993018	6.37E-06	0.00028083	-1
yhjA	0.33572771	-1.5746365	1.05E-05	3.60E-05	-1	0.37957035	-1.3975608	0.00142651	0.00378149	-1	1.1305899	0.17707571	0.61488581	0.92897598	0
treF	601.04821	9.2313369	2.07E-111	9.13E-108	1	46.5923752	5.54202197	6.17E-39	3.89E-36	1	0.07751853	-3.6893149	4.35E-23	9.60E-20	-1
yhjB	9.54708281	3.25505997	0.0001013	0.00028211	1	5.37042142	2.4250353	0.01097444	0.02281093	1	0.56251962	-0.8300247	0.35162546	0.84775072	0
yhjC	1.49185528	0.57710759	0.34807807	0.41824288	0	2.27836441	1.18799851	0.2421669	0.3272636	0	1.52720203	0.61089093	0.64439691	0.93208609	0
yhjD	1.52730499	0.61098818	0.08162523	0.11649067	0	0.68977859	-0.5357947	0.20083224	0.28015468	0	0.45163121	-1.1467829	0.00505502	0.05559259	0
yhjE	3.5406532	1.82401554	0.00014454	0.00039047	1	4.71136542	2.23614523	5.41E-05	0.00020846	1	1.33064865	0.41212969	0.3732887	0.85857675	0
mhpD	7.10921429	2.82969012	0.00015065	0.000406	1	8.79240852	3.13625842	0.00022908	0.00074542	1	1.23676234	0.3065683	0.62272547	0.92989404	0
yhjG	3.63264383	1.86101992	8.64E-08	4.01E-07	1	1.87625294	0.90785433	0.0074228	0.01619021	0	0.51649791	-0.9531656	0.04081706	0.25974493	0
yhjH	3.9199452	1.97083349	0.00095626	0.00215499	1	5.13381171	2.36003038	0.00078739	0.00221763	1	1.30966415	0.3891969	0.51696948	0.90975076	0
kdgK	0.1466505	-2.7695461	3.58E-14	3.37E-13	-1	0.21137871	-2.242098	2.66E-07	1.79E-06	-1	1.44137734	0.52744807	0.1433735	0.57271479	0
yhjJ	0.63726488	-0.6500349	0.08150817	0.11636122	0	1.03308746	0.0469624	0.77183205	0.82464358	0	1.62112724	0.69699733	0.06069951	0.33544465	0
dctA	0.75923516	-0.3973813	0.13644074	0.18433044	0	0.51691382	-0.9520043	0.00235075	0.00587222	0	0.68083493	-0.554623	0.04850073	0.29299759	0
yhjK	4.56844733	2.19170392	9.58E-11	6.50E-10	1	1.83982475	0.87956835	0.04687158	0.08135331	0	0.4027243	-1.3121356	0.00040013	0.00794858	-1
bcsC	1.80148585	0.84918732	0.0029732	0.00599664	0	2.60242836	1.37985845	0.00016097	0.00054717	1	1.44460106	0.53067113	0.15015261	0.58599383	0
bcsZ	3.28673596	1.71665556	0.01250896	0.02189961	1	4.54883992	2.18549867	0.00370542	0.00872612	1	1.3839992	0.46884311	0.41521087	0.87131399	0
bcsB	3.06820023	1.61739263	0.00343398	0.00680785	1	3.19876299	1.6775141	0.00242329	0.0060194	1	1.04255353	0.06012147	0.62557529	0.92989404	0
bcsA	4.36678208	2.12657054	0.00040936	0.00099239	1	4.9440623	2.30569692	0.00037597	0.00114817	1	1.13219808	0.17912639	0.58959073	0.92806272	0
caiC	3.25883244	1.70435517	0.00017312	0.00045955	1	3.492962	1.80445095	0.00019514	0.00064641	1	1.07184462	0.10009578	0.60490519	0.92806272	0
mhpF	3.9728241	1.99016492	0.00018699	0.00049251	1	3.04775759	1.60774816	0.0025105	0.00617357	1	0.76715141	-0.3824168	0.80999039	0.96990832	0
bcsQ	3.94897763	1.9814792	9.19E-06	3.17E-05	1	3.64608019	1.86634629	0.00012322	0.00043182	1	0.92329725	-0.1151329	1	1	0
yhjR	8.17337382	3.03093172	0.0003519	0.00086545	1	4.83466785	2.27341678	0.07342753	0.12039936	0	0.59151434	-0.7575149	0.2257236	0.70950897	0
bcsE	0.52712835	-0.9237738	0.00216666	0.00450785	0	0.36387655	-1.458479	0.00024791	0.00079964	-1	0.69029971	-0.5347052	0.18341914	0.65259624	0
bcsF	0.53267518	-0.908672	0.13841816	0.18662791	0	0.49142129	-1.0249677	0.0977924	0.15371831	0	0.92255339	-0.1162957	0.62606891	0.92989404	0
bcsG	1.05299151	0.07449381	0.86692716	0.89467693	0	1.05432622	0.07632132	0.72291792	0.78781117	0	1.00126754	0.00182752	0.85742842	0.98347354	0
ldrD	0.68226432	-0.5515973	0.48736087	0.55667777	0	0.6634225	-0.5920002	0.28636509	0.37606161	0	0.97238341	-0.0404028	0.67951303	0.94263998	0
rdlD	0.60975385	-0.7137011	0.33769417	0.40742834	0	0.58821036	-0.7655959	0.6756276	0.74808277	0	0.96466854	-0.0518948	0.72954391	0.95488877	0
yhjV	5.24206747	2.39013592	1.56E-09	9.15E-09	1	3.04749454	1.60762364	0.00014013	0.00048492	1	0.58135355	-0.7825123	0.12791991	0.53573295	0
dppF	12.3676499	3.62849948	1.41E-09	8.36E-09	1	5.53024278	2.46734282	0.00016749	0.00056628	1	0.44715389	-1.1611567	0.0874517	0.42333918	0
dppD	15.2815706	3.93372093	2.16E-23	4.07E-22	1	5.34880787	2.41921738	1.11E-08	9.78E-08	1	0.35001689	-1.5145035	0.00076168	0.01376644	-1
mhpE	5.06815171	2.34145971	9.04E-07	3.67E-06	1	5.84513938	2.54723743	1.88E-06	1.04E-05	1	1.15330789	0.20577772	0.54823979	0.91919682	0

dppC	10.044626	3.32835194	1.62E-12	1.34E-11	1	4.27360222	2.09545263	4.65E-05	0.00018267	1	0.42546156	-1.2328993	0.02319431	0.17358733	0
dppB	11.2544357	3.49242181	1.50E-11	1.12E-10	1	5.41697865	2.4374884	2.86E-05	0.00011858	1	0.48131944	-1.0549334	0.0626837	0.34169979	0
dppA	2.48429452	1.31283622	0.05214647	0.07893452	0	0.95405983	-0.0678483	0.86355029	0.89629718	0	0.38403652	-1.3806846	0.06114134	0.33620111	0
proK	1.68601757	0.75361957	0.26985128	0.33622936	0	2.07576434	1.05364266	0.40794803	0.49854047	0	1.23116412	0.30002309	1	1	0
eptB	0.6078962	-0.7181031	0.08316207	0.11849253	0	0.89643079	-0.1577359	0.68965103	0.75925086	0	1.47464452	0.56036722	0.23524068	0.72800801	0
yhjX	4.46057596	2.15723001	3.93E-09	2.22E-08	1	4.88195174	2.28745803	1.70E-06	9.50E-06	1	1.09446667	0.13022803	0.95307439	1	0
yhjY	3.3000107	1.7224707	6.88E-07	2.84E-06	1	1.85657786	0.89264582	0.07645017	0.12470775	0	0.56259753	-0.8298249	0.02140319	0.16415314	0
tag	1.01722586	0.02464004	0.84328101	0.87456497	0	0.90030292	-0.1515176	0.84322938	0.88132229	0	0.88505705	-0.1761576	0.72969958	0.95488877	0
yiaC	0.84144372	-0.2490613	0.43360906	0.5050768	0	0.73539054	-0.4434175	0.76284454	0.81829081	0	0.87396283	-0.1943562	0.83541344	0.98014902	0
bisC	0.94590996	-0.0802252	0.82584054	0.86002474	0	1.17428765	0.23178584	0.27895095	0.36741706	0	1.24143703	0.31201108	0.20460027	0.68449451	0
mhpT	4.21813157	2.0766041	0.00161147	0.00345522	1	4.7545662	2.24931372	0.00150589	0.00396339	1	1.12717352	0.17270962	0.61952797	0.92897598	0
yiaD	0.92032566	-0.1197836	0.77601947	0.81903895	0	1.28100505	0.35727616	0.41025265	0.50107973	0	1.3919041	0.47705981	0.27555413	0.77762465	0
ghrB	0.2658369	-1.9113867	2.56E-08	1.28E-07	-1	0.29029039	-1.7844313	3.60E-05	0.00014569	-1	1.09198681	0.12695543	0.55366798	0.92069223	0
yiaF	0.2491851	-2.0047103	9.67E-10	5.87E-09	-1	0.24464809	-2.0312201	5.48E-08	4.26E-07	-1	0.98179263	-0.0265098	0.6012363	0.92806272	0
yiaG	0.75959366	-0.3967002	0.30462078	0.37230492	0	0.38613861	-1.3728093	0.02785835	0.05166063	0	0.5083489	-0.9761091	0.11600701	0.50522834	0
cspA	0.43813793	-1.190543	0.32603859	0.39541803	0	2.19786486	1.13610268	0.70214441	0.76934692	0	5.01637659	2.32664566	0.21846866	0.70102268	0
mokA	3.15824949	1.65912514	0.26304302	0.32941906	0	3.82189311	1.93428743	0.30116893	0.39131593	0	1.21013021	0.27516229	0.92480491	1	0
insJ	1.97330363	0.98061296	0.41820167	0.49101456	0	1.79380339	0.84302177	0.55861194	0.64300759	0	0.90903567	-0.1375912	0.89459522	0.9954996	0
insK	2.16142471	1.11198259	0.23317954	0.29567081	0	2.27461792	1.18562423	0.07726943	0.12576561	0	1.05236972	0.07364164	0.57561625	0.92806272	0
sokA	3.70331206	1.88881612	0.48982895	0.55891875	0	0.6239514	-0.6804944	1	1	0	0.16848469	-2.5693106	0.71731556	0.9533941	0
yaiL	0.54919999	-0.8645965	0.06574423	0.0963478	0	0.59856184	-0.7404278	0.11121251	0.17028693	0	1.08987957	0.12416873	1	1	0
glyS	0.15856795	-2.6568269	6.31E-16	6.99E-15	-1	0.27751497	-1.8493625	5.54E-07	3.43E-06	-1	1.75013278	0.80746439	0.04202297	0.26361494	0
glyQ	0.32659348	-1.6144321	9.37E-07	3.79E-06	-1	0.65404835	-0.6125308	0.11776275	0.17870658	0	2.00263752	1.00190132	0.00623078	0.06561167	0
ysaB	4.10750261	2.03826149	0.09106283	0.12846519	0	1.80175755	0.84940489	0.24563625	0.33109899	0	0.43865037	-1.1888566	0.63070192	0.93147872	0
wecH	3.5178884	1.81470972	4.38E-06	1.60E-05	1	2.77424933	1.47209745	0.00074461	0.00211602	1	0.78861209	-0.3426123	0.58498289	0.92806272	0
yiaA	5.94021732	2.57051571	0.01392239	0.02413469	1	3.1535431	1.65697365	0.07220952	0.1187689	0	0.53088009	-0.9135421	0.65201954	0.9347567	0
yiaB	3.40974489	1.7696638	0.00305114	0.00613423	1	4.36012903	2.12437083	0.00439813	0.01013313	1	1.27872588	0.35470703	0.65778338	0.93664426	0
xylB	3.48896637	1.80279969	2.33E-06	8.98E-06	1	3.73514787	1.90116536	9.44E-06	4.46E-05	1	1.07056001	0.09836567	0.64924677	0.93327989	0
xylA	1.85250439	0.88947696	0.00614438	0.01150963	0	1.46087622	0.54683394	0.12420502	0.18662427	0	0.78859528	-0.342643	0.47839933	0.89890969	0
xylF	0.91483044	-0.1284237	0.57943866	0.64347281	0	0.71288143	-0.488266	0.3092532	0.3998215	0	0.77924979	-0.3598422	0.49668765	0.90539057	0
xylG	1.27582808	0.35143393	0.23310494	0.29567081	0	1.27372676	0.34905582	0.33549913	0.42565911	0	0.99835298	-0.0023781	1	1	0
frmB	0.50947183	-0.9729257	0.01889912	0.03158434	0	0.49544351	-1.0132075	0.04092608	0.07265859	0	0.97246498	-0.0402818	0.89866761	0.99646774	0
xylH	2.40710081	1.26729656	0.00700242	0.01295214	1	2.95670332	1.56398949	0.00129576	0.0034745	1	1.22832551	0.29669293	0.38178124	0.85988523	0
xylR	1.63293566	0.70746794	0.04392935	0.06786479	0	1.29929001	0.37772348	0.77963601	0.83117286	0	0.79567741	-0.3297445	0.19156034	0.66643604	0
bax	0.10866241	-3.2020751	2.13E-19	3.02E-18	-1	0.26807316	-1.8993013	1.17E-07	8.53E-07	-1	2.46702752	1.30277381	0.00911507	0.0871962	0
malS	0.31190031	-1.6808431	5.13E-07	2.15E-06	-1	0.27943335	-1.8394239	4.03E-05	0.00016179	-1	0.89590599	-0.1585807	0.90975655	0.99971546	0
avtA	0.99047253	-0.0138111	0.97222885	0.98133794	0	1.32333849	0.40418213	0.36185815	0.45376026	0	1.33606784	0.41799326	0.34466453	0.84269271	0
ysaA	0.03890347	-4.6839574	4.10E-35	1.81E-33	-1	0.04327262	-4.5304016	8.92E-22	6.07E-20	-1	1.11230766	0.15355588	0.39841556	0.86854717	0
yiaJ	1.17747297	0.23569394	0.52326197	0.59111205	0	1.64598948	0.71895512	0.05386695	0.09158832	0	1.39790002	0.48326118	0.16029573	0.60475422	0
yiaK	2.64119437	1.40119048	0.00514391	0.00978113	1	2.63354202	1.39700448	0.00554059	0.01246633	1	0.99710269	-0.004186	0.75529271	0.9573569	0
yiaL	2.69404713	1.42977509	0.0036412	0.00717359	1	5.52656583	2.46638328	0.00093062	0.00258967	1	2.05139909	1.03660819	0.2600302	0.75892335	0
yiaM	3.00408131	1.58692386	0.04721679	0.07236522	0	4.84750128	2.27724128	0.00506457	0.01150069	1	1.61363851	0.69031742	0.26847974	0.76966758	0
frmA	0.55014511	-0.8621159	0.03272662	0.05196028	0	0.37053895	-1.4323029	0.00176816	0.00455068	-1	0.67352947	-0.570187	0.1862549	0.65763341	0
yiaN	8.53166821	3.09282786	3.35E-14	3.18E-13	1	10.2416429	3.35637525	6.19E-14	1.23E-12	1	1.20042676	0.26354739	0.36334965	0.85329623	0
yiaO	2.91432713	1.54316283	0.001267	0.00277734	1	4.8259581	2.27081539	4.09E-05	0.00016311	1	1.65594248	0.72765256	0.14272818	0.57181814	0
lyxK	4.06004651	2.02149625	0.00268762	0.00547313	1	7.97724045	2.99588976	5.54E-05	0.00021277	1	1.96481504	0.97439351	0.12272466	0.52159375	0
sgbH	2.65956492	1.41119025	0.00370158	0.00728279	1	5.25792535	2.39449366	9.83E-06	4.63E-05	1	1.97698703	0.9833034	0.04185468	0.26293323	0
sgbU	3.18449267	1.67106355	0.00780888	0.01430602	1	4.64931601	2.21701849	0.00031898	0.00099369	1	1.45998641	0.54595494	0.20380246	0.68295505	0
sgbE	3.30715071	1.72558879	2.94E-05	9.18E-05	1	4.78905765	2.2597418	3.32E-06	1.72E-05	1	1.44809175	0.53415301	0.22279433	0.70531966	0
ysaC	1.06528894	0.09124478	0.7589134	0.80483084	0	3.64949168	1.86769553	0.2604813	0.34775434	0	3.42582332	1.77645075	0.36434394	0.85329623	0
ysaD	3.31868585	1.73061207	0.0229808	0.03777678	1	5.16710495	2.36935619	0.0091768	0.01950567	1	1.5569732	0.63874412	0.4458799	0.88613355	0
yiaT	4.38897864	2.13388525	2.06E-05	6.61E-05	1	5.99113451	2.58282923	4.24E-06	2.15E-05	1	1.36504071	0.44894398	0.30786106	0.81054764	0
yiaU	1.7171523	0.780018	0.07119744	0.10324208	0	1.65993947	0.73113064	0.13577101	0.20173911	0	0.96668157	-0.0488874	0.95524541	1	0
frmR	0.90606355	-0.1423159	0.91080335	0.93125426	0	0.61036864	-0.7122472	0.25277255	0.33909805	0	0.67364883	-0.5699314	0.3075135	0.81011621	0
yiaV	3.97901388	1.99241093	0.00012016	0.00032926	1	5.46453657	2.45009915	2.55E-05	0.00010733	1	1.37333941	0.45768822	0.32118169	0.82206109	0
yiaW	5.57177423	2.4781368	0.00052219	0.00124207	1	5.90587512	2.56215085	0.00046108	0.00137205	1	1.05996311	0.08401405	0.65427785	0.93589534	0
aldB	7.5914887	2.92438283	0.00019692	0.00051559	1	2.19992231	1.13745258	0.1901352	0.26863436	0	0.289788	-1.7869303	0.05025017	0.29745283	0
yiaY	4.82534632	2.27063249	5.03E-06	1.82E-05	1	7.26894787	2.86174656	2.43E-07	1.64E-06	1	1.50640957	0.59111407	0.19463181	0.6726695	0
selB	0.15138909	-2.7236669	4.43E-15	4.56E-14	-1	0.29255907	-1.7732002	1.02E-05	4.77E-05	-1	1.93249773	0.95046672	0.00990115	0.09270506	0
selA	0.20520132	-2.2848881	2.88E-11	2.09E-10	-1	0.30133501	-1.7305598	1.95E-05	8.40E-05	-1	1.46848474	0.55432827	0.13857653	0.56089533	0
yibF	1.13997028	0.18899621	0.78809811	0.82921138	0	1.37308631	0.45742231	0.57728955	0.66072067	0	1.20449308	0.2684261	0.75216427	0.95683847	0
rhsA	2.01828944	1.01313309	0.21472412	0.27523663	0	1.67190497	0.74149285	0.37638575	0.46759872	0	0.82837721	-0.2716402	0.80676534	0.96990832	0
yibA	9.93687101	3.31279164	0.01361332	0.02368257	1	14.5932395	3.86722828	0.02805412	0.05195774	0	1.46859505	0.55443664	0.8922201	0.99488354	0
rhsJ	4.96587445	2.31204779	9.01E-05	0.00025377	1	3.44298622	1.78366041	0.00295628	0.0071426	1	0.69332929	-0.5283874	0.59300073	0.92806272	0
yaiO	5.30433092	2.40717078	1.76E-07	7.86E-07	1	5.62197749	2.49107768	1.94E-06	1.07E-05	1	1.05988438	0.08390689	0.74175935	0.95683847	0
yibG	2.72140656	1.4443525	0.48440924	0.55387927	0	2.93528902	1.55350256	0.4415727	0.5315472	0	1.07859261	0.10915006	0.90076457	0.99683105	0
yibS	2.79903973	1.48493196	0.44047315	0.51110888	0	1.98320878	0.98783656	0.37904737	0.47037639	0	0.70853184	-0.4970954	0.94178607	1	0
yibW	2.23659803	1.161306	0.13263523	0.17965342	0	1.28105326	0.35733045	0.70424951	0.77127103	0	0.57276866	-0.8039755	0.38175973	0.85988523	0
yibV	2.45499006	1.29571718	0.2041774	0.26316065	0	0.9900082	-0.0144876	0.92791054	0.94763963	0	0.40326363	-1.3102048	0.28321763	0.78651746	0
yibU	5.05792373	2.33854528	0.02928068	0.04692771	1	2.40364559	1.26522419	0.15729402	0.22879601	0	0.47522377	-1.0733211	0.67330209	0.93997784	0
yibH	2.14247505	1.0992784	0.01347819	0.02348448	1	1.53733505	0.62043162	0.10655182	0.1645762	0	0.71755097	-0.4788468	0.5580509	0.92317654	0
yibI	1.04863573	0.06851361	0.96952751	0.97950664	0	0.80422247	-0.3143335	0.80402844	0.84984957	0	0.76692263	-0.3828471	0.72644042	0.95487077	0
mtlA	0.12144041	-3.0416795	1.68E-20	2.53E-19	-1	0.05205932	-4.2636998	1.88E-23	1.85E-21	-1	0.42868199	-1.2220203	0.00122375	0.01969613	-1
mtlD	0.10122503	-3.304362	6.44E-24	1.28E-22	-1	0.04902173	-4.3504347	2.28E-25	2.65E-23	-1	0.48428466	-1.0460728	0.00263779	0.03451832	-1
mtlR	0.46330487	-1.1099662	7.53E-05	0.00021715	-1	0.14323274	-2.8035668	1.91E-13	3.52E-12	-1	0.30915441	-1.6936005	1.67E-06	9.16E-05	-1
yaiX	5.65923036	2.50060586	0.00713524	0.01317023	1	5.08510977	2.34627892	0.01729076	0.03390843	1	0.89855147	-0.1543269	1	1	0
yibT	2.00176988	1.00127614	0.42996755	0.5016288	0	0.37756792	-1.4051919	0.19038478	0.26887515	0	0.18861704	-2.406468	0.04293494	0.26819136	0
yibL	0.32232986	-1.6333902	7.99E-05	0.00022912	-1	0.28208243	-1.8258113	0.00015071	0.00051696	-1	0.87513587	-0.1924211	0.54956359	0.91965346	0
lldP	8.67387218	3.11667618	1.54E-17	1.97E-16	1	6.42130833	2.68286727	1.23E-11	1.68E-10	1	0.7403047	-0.4338089	0.44181705	0.88123618	0
lldR	4.44237086	2.15132984	2.65E-05	8.33E-05	1	2.30453956	1.20447854	0.01318569	0.02665487	1	0.51876343	-0.9468513	0.20059912	0.67788667	0
lldD	3.31336357	1.72829651	7.33E-07	3.01E-06	1	2.18111611	1.12506657	0.00245864	0.00608668	1	0.65827853	-0.6032299	0.20615647	0.68468503	0
trmL	4.19308035	2.06801047	3.19E-07	1.37E-06	1	2.89813143	1.53512302	0.00035533	0.00109269	1	0.69117002	-0.5328875	0.35539829	0.84810955	0
cysE	4.35228007	2.1217714	8.65E-07	3.52E-06	1	1.93782529	0.9544385	0.24670274	0.33216922	0	0.4452437	-1.1673329	0.00307805	0.03839997	-1
gpsA	0.47606694	-1.0707636	0.0004208	0.00101677	-1	0.40862363	-1.2911555	0.00040538	0.00122697	-1	0.85833229	-0.2203918	0.47399561	0.89828993	0
secB	0.10562864	-3.242927	1.95E-22	3.41E-21	-1	0.16517934	-2.5978949	5.82E-12	8.40E-11	-1	1.56377416	0.64503218	0.15299307	0.591558	0
grxC	0.14059264	-2.830407	1.84E-16	2.12E-15	-1	0.1618846	-2.6269624	2.18E-09	2.16E-08	-1	1.15144432	0.20344465	0.35319001	0.84775072	0
yibN	0.12963235	-2.9475023	1.36E-12	1.13E-11	-1	0.50490252	-0.9859232	0.00535799	0.01211098	0	3.89488053	1.96157908	0.00027424	0.00598713	1
gpmM	0.05345566	-4.2255133	6.73E-34	2.75E-32	-1	0.07864574	-3.6684876	1.56E-19	7.20E-18	-1	1.47123303	0.55702577	0.1428897	0.57181814	0
envC	0.57562872	-0.7967895	0.02340217	0.03840159	0	0.97871132	-0.0310447	0.9245962	0.94469147	0	1.70024756	0.76574482	0.06004288	0.33223226	0
yibQ	1.46220678	0.54814734	0.17878331	0.2339117	0	2.02524395	1.0180957	0.01729568	0.03390843	1	1.38505989	0.46994836	0.20543036	0.68468503	0
waaH	2.65632498	1.40943166	0.00807069	0.01471851	1	2.67387976	1.41893459	0.00668187	0.01474885	1	1.00660867	0.00950293	0.71942964	0.95399541	0
tdh	0.10448692	-3.2586057	1.61E-22	2.84E-21	-1	0.08799645	-3.5064109	7.37E-18	2.65E-16	-1	0.84217664	-0.2478052	0.46484615	0.89206388	0
kbl	0.09204664	-3.4414911	2.60E-25	5.86E-24	-1	0.08587887	-3.541553	6.35E-18	2.36E-16	-1	0.93299295	-0.1000619	0.93737489	1	0
yibB	0.55009485	-0.8622477	0.07513725	0.10817511	0	0.90601922	-0.1423864	0.4533481	0.54267797	0	1.64702365	0.71986127	0.4615739	0.88879788	0
hldD	0.16578991	-2.5925719	1.35E-16	1.58E-15	-1	0.15886774	-2.6541019	1.42E-11	1.92E-10	-1	0.95824732	-0.06153	1	1	0
waaF	0.41472877	-1.26976	0.00023876	0.00061349	-1	0.34890548	-1.5190918	0.00030614	0.00096219	-1	0.84128594	-0.2493319	0.55039874	0.91976447	0
caiB	3.29020298	1.71817659	0.0006208	0.00145166	1	2.80083105	1.48585496	0.00278806	0.00677318	1	0.85126391	-0.2323216	0.99779174	1	0
waaC	1.03271997	0.04644911	0.95538845	0.96887978	0	1.27100416	0.34596876	0.51367867	0.60099181	0	1.23073456	0.29951965	0.54091866	0.91914725	0
waaL	0.27091403	-1.884093	1.26E-07	5.73E-07	-1	0.38450212	-1.3789366	0.00047098	0.00139307	-1	1.41927722	0.50515641	0.39642163	0.86854717	0
waaU	0.23109031	-2.1134714	7.66E-08	3.59E-07	-1	0.401632	-1.3160539	0.00081776	0.00229731	-1	1.73798721	0.79741747	0.25475923	0.75459294	0
waaZ	1.69573443	0.76191024	0.44482387	0.5151513	0	2.29510295	1.19855887	0.5307884	0.61708865	0	1.3534566	0.43664863	0.9783559	1	0
waaY	1.89126967	0.91935509	0.41932612	0.49220395	0	3.62023064	1.85608161	0.31782005	0.40791368	0	1.91418003	0.93672653	0.68894019	0.94487465	0

waaR	2.38089361	1.25150315	0.21999753	0.28117162	0	3.21551379	1.68504928	0.30773701	0.39818652	0	1.35054913	0.43354612	0.96344456	1	0
waaO	0.4430098	-1.1745895	0.00642648	0.01196701	-1	0.70155634	-0.5113691	0.11365	0.17317909	0	1.58361358	0.66322035	0.50853307	0.90925684	0
waaB	0.3932508	-1.3464784	0.00015721	0.00042136	-1	0.39666652	-1.3340015	0.00249162	0.00613395	-1	1.00868586	0.01247694	0.97867347	1	0
waaS	0.43498877	-1.2009499	0.01258592	0.02199921	-1	0.61281282	-0.7064816	0.08131775	0.13129087	0	1.40880148	0.49446833	0.74778236	0.95683847	0
waaP	0.95566022	-0.0654303	0.71320636	0.76258351	0	0.83987004	-0.251762	0.55083782	0.63505148	0	0.8788375	-0.1863317	0.70103885	0.94869712	0
yaiP	6.90967909	2.78861871	7.95E-08	3.72E-07	1	7.68285137	2.94164184	3.11E-07	2.05E-06	1	1.11189699	0.15302313	0.62802714	0.93095788	0
waaG	0.77140712	-0.3744356	0.30238079	0.3701818	0	0.8598423	-0.217856	0.76709529	0.82117105	0	1.11464139	0.15657963	0.58092576	0.92806272	0
waaQ	0.71143067	-0.4912049	0.22669496	0.2884793	0	0.57652063	-0.7945559	0.08656363	0.1384648	0	0.81036798	-0.3033509	0.47887503	0.89894957	0
waaA	1.26587851	0.34013896	0.42848328	0.5003014	0	1.55877932	0.6404167	0.11331671	0.17279039	0	1.23138145	0.30027774	0.37945419	0.85988523	0
coaD	0.43481009	-1.2015427	0.0251834	0.04104616	-1	0.7280219	-0.4579462	0.56029726	0.64444393	0	1.67434456	0.74359645	0.14730691	0.58035154	0
mutM	2.40078373	1.26350545	0.00145167	0.00314469	1	2.82364552	1.49755899	0.00066645	0.00191112	1	1.1761349	0.23405354	0.47100627	0.89609045	0
rpmG	0.06096814	-4.0358006	6.23E-28	1.71E-26	-1	0.20450383	-2.2898002	1.49E-10	1.79E-09	-1	3.35427358	1.74600036	0.00082746	0.01453465	1
rpmB	0.05606788	-4.1566816	1.55E-32	5.96E-31	-1	0.17616834	-2.5049734	2.31E-12	3.56E-11	-1	3.14205437	1.65170814	0.00025663	0.00568714	1
yicR	0.13122712	-2.9298622	2.96E-14	2.82E-13	-1	0.23324928	-2.1000555	7.50E-07	4.54E-06	-1	1.77744721	0.82980671	0.10391347	0.47389701	0
dfp	0.85230832	-0.2305527	0.51077694	0.58012387	0	1.19051581	0.25158678	0.47393508	0.5631404	0	1.39681355	0.48213946	0.19153688	0.66643604	0
dut	0.25317607	-1.9817871	5.13E-07	2.15E-06	-1	0.46411009	-1.107461	0.01216802	0.02489611	-1	1.83315149	0.87432602	0.06379735	0.34521019	0
yaiS	6.49058452	2.69834841	0.00162413	0.00347568	1	5.58032837	2.48035002	0.00591435	0.01322646	1	0.85975745	-0.2179984	0.99055996	1	0
slmA	0.52898797	-0.9186932	0.02578848	0.04193952	0	0.99224521	-0.0112314	0.73128641	0.79418891	0	1.8757425	0.90746179	0.11260785	0.49654656	0
pyrE	1.64890454	0.72150788	0.10536427	0.1463043	0	2.25041592	1.17019166	0.02598422	0.04854915	1	1.36479455	0.44868379	0.35862188	0.85183882	0
rph	0.61568463	-0.6997365	0.09186102	0.12942588	0	1.15754369	0.21106665	0.56550968	0.64942497	0	1.88009191	0.91080319	0.03676637	0.24020698	0
yicC	0.26355209	-1.92384	2.55E-08	1.28E-07	-1	0.41458634	-1.2702555	0.0005492	0.00160618	-1	1.57307173	0.65358446	0.20618179	0.68468503	0
dinD	0.80877681	-0.3061865	0.26410514	0.33056175	0	1.11257599	0.15390387	0.88077749	0.91044578	0	1.37562796	0.46009034	0.45924667	0.88789032	0
yicG	3.14801034	1.65444028	1.29E-05	4.33E-05	1	2.77673748	1.47339079	0.00106765	0.00293588	1	0.8820611	-0.1810495	0.67145421	0.93979017	0
ligB	5.11310724	2.35420029	2.43E-11	1.78E-10	1	5.29286142	2.40404788	8.86E-09	7.99E-08	1	1.03515557	0.0498476	0.9434556	1	0
gmk	0.83621801	-0.258049	0.26438411	0.33081718	0	0.39436545	-1.3423949	0.00042602	0.00127893	-1	0.47160603	-1.0843459	0.00431794	0.04907765	-1
rpoZ	0.29150347	-1.778415	8.04E-08	3.75E-07	-1	0.52139828	-0.9395423	0.00848367	0.01813245	0	1.78865204	0.83887275	0.05632167	0.31925265	0
spoT	0.67701724	-0.5627355	0.06279313	0.09269961	0	0.86378202	-0.2112608	0.41674247	0.50746348	0	1.27586414	0.35147471	0.45445969	0.88755478	0
tauA	7.43240375	2.89382888	1.28E-07	5.82E-07	1	10.9729845	3.45588406	1.29E-07	9.28E-07	1	1.47637088	0.56205519	0.38161655	0.85988523	0
trmH	0.96668727	-0.0488789	0.70506089	0.75533688	0	1.25068927	0.3227234	0.38382212	0.47496779	0	1.29378891	0.37160225	0.45788823	0.88755478	0
recG	1.14421221	0.19435465	0.42711186	0.49922018	0	1.23451407	0.30394328	0.35808442	0.44936629	0	1.07892055	0.10958863	0.74791081	0.95683847	0
gltS	0.67123474	-0.5751107	0.15796314	0.20996786	0	0.68471114	-0.5464326	0.30284045	0.3930402	0	1.02007704	0.02867811	0.81566989	0.97192764	0
xanP	2.38775956	1.25565757	0.00116559	0.0025742	1	2.96258948	1.56685873	0.00018191	0.00060623	1	1.24074028	0.31120116	0.31435768	0.81404427	0
yicH	0.39568339	-1.3375816	6.95E-05	0.00020189	-1	0.42437721	-1.2365809	0.002962	0.00714862	-1	1.07251711	0.10100066	0.69209298	0.94603882	0
yicI	1.90974763	0.933382	0.00653104	0.01214636	0	2.60562043	1.38162694	0.00038984	0.00118719	1	1.36437945	0.44824493	0.19642388	0.67373807	0
yicJ	5.6780629	2.50539883	3.51E-11	2.54E-10	1	5.0111934	2.32515422	1.43E-08	1.23E-07	1	0.88255334	-0.1802446	0.92153478	1	0
selC	6.42859265	2.68450294	0.0004642	0.00111193	1	8.87115231	3.14912151	6.85E-05	0.00025719	1	1.37995247	0.46461858	0.35338343	0.84775072	0
yicT	5.42063211	2.4384611	0.00625486	0.0116918	1	5.58157427	2.48067209	0.00769951	0.01671948	1	1.02969066	0.04221099	0.74985151	0.95683847	0
setC	7.99011492	2.99821625	1.70E-10	1.13E-09	1	6.90352746	2.78733372	1.13E-08	9.94E-08	1	0.86400853	-0.2108825	1	1	0
tauB	7.75166056	2.9545054	2.37E-05	7.52E-05	1	6.63652958	2.73042902	0.00012885	0.00044976	1	0.85614296	-0.2240764	1	1	0
yicL	1.05072607	0.0713866	0.77231267	0.81629697	0	1.25850417	0.33171	0.59440693	0.67524016	0	1.19774717	0.2603234	0.72553623	0.95459348	0
nlpA	0.37913995	-1.3991976	0.00112173	0.00248728	-1	0.26508587	-1.9154683	0.00122642	0.00330864	-1	0.69917683	-0.5162707	0.53599505	0.9187668	0
yicS	1.80782568	0.85425557	0.13080571	0.17755649	0	2.14613838	1.1017431	0.05459204	0.09267854	0	1.1871379	0.24748753	0.51253433	0.90925684	0
nepI	2.8484804	1.51019248	7.34E-05	0.00021237	1	4.39661646	2.13639368	1.29E-06	7.38E-06	1	1.54349542	0.62620121	0.11317177	0.49759471	0
yicN	0.45214846	-1.1451315	0.02740855	0.04432938	-1	0.99626171	-0.0054033	0.89268473	0.91878132	0	2.20339513	1.13972823	0.06902078	0.36443923	0
adeQ	3.74295867	1.90417912	0.00033704	0.00083308	1	4.38815482	2.13361443	0.00014176	0.00049017	1	1.17237597	0.22943531	0.46846363	0.89538835	0
adeD	0.66503096	-0.5885066	0.10709845	0.14852555	0	0.81270783	-0.2991913	0.72940191	0.79253185	0	1.22206014	0.28931528	0.28747703	0.78998378	0
uhpT	1.7164558	0.77943271	0.0643828	0.09472979	0	2.71818971	1.44264615	0.00133519	0.00356723	1	1.583606	0.66321344	0.09477587	0.44916863	0
uhpC	3.85285031	1.94592614	5.13E-05	0.00015414	1	4.741891	2.2454625	2.58E-05	0.00010818	1	1.23074883	0.29953636	0.42780238	0.87784805	0
uhpB	2.44573179	1.2902662	0.00198452	0.00417411	1	3.91840943	1.97026815	3.80E-06	1.94E-05	1	1.60214192	0.68000195	0.04859697	0.29310768	0
tauC	5.79597152	2.53505051	5.46E-08	2.63E-07	1	7.86988588	2.97634272	5.63E-09	5.24E-08	1	1.35781997	0.44129221	0.24007586	0.73319565	0
uhpA	0.91315416	-0.1310697	0.84284835	0.87432155	0	1.92308651	0.94342366	0.08536226	0.13711952	0	2.1059823	1.07449331	0.04444412	0.27412389	0
ilvN	0.63725525	-0.6500567	0.28352077	0.35049293	0	0.89384182	-0.1619085	0.53504679	0.62138548	0	1.40264332	0.48814819	0.75591823	0.9573569	0
ilvB	1.3120977	0.39187515	0.17290033	0.2275628	0	1.47335784	0.55910787	0.12389752	0.18628893	0	1.12290254	0.16723272	0.64020583	0.93163024	0
ivbL	0.36818901	-1.4414815	0.03950077	0.06175377	0	0.91926429	-0.1214484	0.5267128	0.61339253	0	2.49671842	1.32003312	0.28286485	0.78651746	0
istR	4.93251984	2.30232485	0.08777733	0.12414744	0	4.97873591	2.31577949	0.0989096	0.15532392	0	1.00936967	0.01345464	0.90542986	0.99890653	0
tisB	0.40892406	-1.2900952	0.05293512	0.07999126	0	0.78932167	-0.3413147	0.33403589	0.42404613	0	1.93024025	0.94878042	0.54615498	0.91914725	0
emrD	0.42215751	-1.2441467	0.00257474	0.00526511	-1	1.20418516	0.26805724	0.58663806	0.66882187	0	2.85245466	1.51220395	0.00105961	0.01737129	1
yidF	0.54355041	-0.8795143	0.01547813	0.02648854	0	0.52477822	-0.9302203	0.0099063	0.02089545	0	0.96546375	-0.050706	0.51067057	0.90925684	0
yidG	1.87915143	0.91008133	0.07075604	0.10267064	0	2.42736518	1.27939117	0.06257579	0.10478303	0	1.29173474	0.36930984	0.67724295	0.94128448	0
yidH	1.14517478	0.19556781	0.94236895	0.95732375	0	1.49739663	0.58245641	0.65633547	0.73038188	0	1.30757038	0.3868886	0.73385026	0.95599369	0
tauD	2.87626702	1.52419761	0.00297534	0.0059982	1	3.16207221	1.66087031	0.00208871	0.00526227	1	1.09936671	0.1366727	0.57577685	0.92806272	0
yidI	0.95336773	-0.0688953	0.95610645	0.96887978	0	0.53754176	-0.8955513	0.29585292	0.38588372	0	0.56383465	-0.826656	0.30085257	0.80507272	0
yidJ	3.02260152	1.5957908	0.0001979	0.00051786	1	3.60353043	1.84941103	5.90E-05	0.00022452	1	1.192195	0.25362023	0.41181753	0.8712535	0
yidK	5.48166177	2.45461331	1.46E-06	5.77E-06	1	6.66256784	2.73607832	2.70E-06	1.44E-05	1	1.21542848	0.281465	0.52993361	0.91706103	0
yidL	0.70919502	-0.4957457	0.30141491	0.36940889	0	0.61410688	-0.7034383	0.4152041	0.50614812	0	0.86592103	-0.2076926	1	1	0
glvG	6.23727921	2.64091684	1.07E-08	5.70E-08	1	4.44786023	2.15311145	2.21E-05	9.40E-05	1	0.71310905	-0.4878054	0.46961247	0.89538835	0
glvC	4.81359503	2.26711477	0.00024757	0.00063392	1	4.8094116	2.2658604	0.00047356	0.00139884	1	0.99913091	-0.0012544	0.75804904	0.95806023	0
yidP	5.86786416	2.55283547	0.00229771	0.00474695	1	4.79318933	2.26098593	0.00839125	0.01796556	1	0.81685417	-0.2918495	0.94544532	1	0
yidE	0.48093762	-1.0560783	0.00140855	0.00305878	-1	0.51325413	-0.9622548	0.01741532	0.03409761	0	1.06719481	0.09382355	0.75595114	0.9573569	0
ibpB	374.668637	8.54947141	6.14E-09	3.38E-08	1	245.083899	7.9371319	2.67E-07	1.79E-06	1	0.65413508	-0.6123395	0.60241997	0.92806272	0
ibpA	59.3162095	5.8903545	1.01E-06	4.08E-06	1	41.6847212	5.38144678	6.29E-05	0.00023759	1	0.7027543	-0.5089077	0.58382696	0.92806272	0
hemB	0.20612765	-2.2783901	2.10E-12	1.70E-11	-1	0.26108434	-1.9374122	1.66E-06	9.33E-06	-1	1.26661485	0.3409779	0.26299533	0.762024	0
yidQ	0.18255028	-2.4536342	1.15E-12	9.61E-12	-1	0.27486608	-1.8631992	4.69E-07	2.96E-06	-1	1.50570066	0.59043498	0.34795213	0.84560166	0
yidR	0.38343784	-1.3829354	5.40E-05	0.00016131	-1	0.53149463	-0.911873	0.03085173	0.05675846	0	1.38612983	0.47106239	0.19964658	0.67679424	0
cbrA	3.84617617	1.94342484	0.00785401	0.01437082	1	4.88316918	2.28781776	0.00354076	0.00838961	1	1.26961662	0.34439292	0.51052312	0.90925684	0
dgoT	2.85123443	1.51158666	0.0565952	0.08475121	0	3.47611376	1.7974753	0.01316031	0.02663666	1	1.21916098	0.28588863	0.44001895	0.88087171	0
dgoD	1.51316321	0.59756761	0.08181653	0.11672598	0	1.72327701	0.78515463	0.04925245	0.08491868	0	1.13885733	0.18758702	0.54924443	0.9195778	0
dgoA	0.72787065	-0.458246	0.76376198	0.80927666	0	1.06695163	0.09349478	0.56060531	0.64463047	0	1.46585336	0.55174079	0.31178871	0.81168136	0
dgoK	0.3084558	-1.6968643	0.00011061	0.00030535	-1	0.3788446	-1.4003219	0.0468504	0.08134849	0	1.22819738	0.29654243	0.18946038	0.66370038	0
dgoR	0.10603887	-3.2373349	6.83E-16	7.54E-15	-1	0.11577167	-3.1106458	7.09E-10	7.68E-09	-1	1.09178529	0.12668917	0.58808697	0.92806272	0
yidX	10.8702417	3.44231211	0.01480846	0.02545096	1	20.7147161	4.37258414	0.01733523	0.0339709	1	1.90563528	0.93027203	0.71637934	0.95281877	0
yidA	0.4691618	-1.0918425	0.00080256	0.00183865	-1	0.54058923	-0.8873953	0.01253688	0.02554194	0	1.15224476	0.20444721	0.78665132	0.9635071	0
yaiT	0.77786104	-0.3624156	0.36927768	0.43988659	0	1.05248605	0.07380111	0.85912887	0.89392288	0	1.35305151	0.43621676	0.33729628	0.83714543	0
yidB	0.72399513	-0.4659481	0.67292168	0.72654487	0	0.86945048	-0.2018242	0.98905704	0.99968963	0	1.20090653	0.26412386	0.64217145	0.93164618	0
gyrB	0.27818689	-1.8458737	1.87E-08	9.57E-08	-1	0.40056823	-1.3198801	0.00038401	0.0011711	-1	1.43992491	0.52599358	0.19888185	0.67679424	0
recF	0.99944886	-0.0007953	0.75562347	0.80172685	0	1.60862094	0.68582441	0.07625685	0.12443834	0	1.609508	0.68661975	0.1020538	0.467835	0
dnaN	0.27291397	-1.8734819	1.67E-08	8.59E-08	-1	0.52714705	-0.9237226	0.01871738	0.036421	0	1.93155028	0.94975923	0.01192851	0.10697905	0
dnaA	0.29024107	-1.7846764	2.97E-08	1.48E-07	-1	0.38653407	-1.3713325	0.00040961	0.00123717	-1	1.331769	0.41334387	0.27056187	0.77178386	0
rpmH	0.12853383	-2.95978	4.28E-06	1.57E-05	-1	0.65971559	-0.6000839	0.1917581	0.2703826	0	5.13262242	2.35969613	0.00481468	0.05321494	0
rnpA	0.36220003	-1.4651414	0.00752337	0.01384038	-1	1.54432585	0.62697719	0.53800396	0.62368924	0	4.26373756	2.09211864	0.00238235	0.03193373	1
yidD	0.46197804	-1.1141038	0.02780979	0.0448306	-1	1.41369225	0.49946809	0.55304516	0.63714171	0	3.06008539	1.61357191	0.01088299	0.09916108	0
yidC	0.21372015	-2.2262052	3.94E-11	2.81E-10	-1	0.68707662	-0.5414571	0.11537879	0.1754504	0	3.21484253	1.68474807	1.14E-05	0.00046272	1
mnmE	0.55056594	-0.8610127	0.02383185	0.03901604	0	0.77678938	-0.3644046	0.52896632	0.61561817	0	1.41089253	0.4966081	0.16493008	0.61352961	0
tnaC	0.03931969	-4.6686043	6.42E-40	4.00E-38	-1	0.06559925	-3.9301769	3.59E-24	3.87E-22	-1	1.6683563	0.73842743	0.31572299	0.81560659	0
tnaA	0.01679388	-5.895921	3.36E-57	4.79E-55	-1	0.08868982	-3.4950877	7.78E-19	3.37E-17	-1	5.28108095	2.40083325	1.38E-11	3.21E-09	1
tnaB	0.01486283	-6.0721474	1.52E-57	2.31E-55	-1	0.05522581	-4.1785135	1.04E-24	1.15E-22	-1	3.71569969	1.89363391	1.36E-06	7.77E-05	1
mdtL	1.0322654	0.04581395	0.67416893	0.72771363	0	1.76332746	0.81830042	0.06245796	0.10462537	0	1.70821134	0.77248647	0.0982761	0.45734597	0
yidZ	1.02528456	0.03602437	0.85964933	0.8888275	0	1.31752733	0.39783289	0.29899591	0.38894993	0	1.28503577	0.36180852	0.35504743	0.84810955	0
yieE	0.3180238	-1.6527933	5.91E-07	2.46E-06	-1	0.42315908	-1.240728	0.00090573	0.00252358	-1	1.33058933	0.41206537	0.40995237	0.87001729	0
chrR	0.51392728	-0.9603639	0.00235295	0.00484974	0	0.40440883	-1.3061136	0.00031631	0.00098916	-1	0.78689893	-0.3457497	0.22276845	0.70531966	0
adeP	1.59454247	0.67314253	0.07283304	0.10530395	0	1.74258845	0.80123189	0.04978624	0.08570525	0	1.09284543	0.12808936	0.6497034	0.9335914	0
yieH	1.65966442	0.73089156	0.17364222	0.22847116	0	1.79022571	0.84014149	0.12253969	0.18481327	0	1.07866728	0.10924993	0.70964021	0.95150907	0

cbrB	2.6327996	1.39659771	0.26452254	0.33089665	0	1.86340594	0.89794199	0.34616733	0.4367435	0	0.70776596	-0.4986557	0.90933065	0.99971546	0
caiA	1.35203543	0.43513296	0.35899402	0.42948986	0	1.67172248	0.74133537	0.10364955	0.16150471	0	1.23644872	0.30620241	0.39090125	0.86854717	0
cbrC	1.49654625	0.58163687	0.34762847	0.41781632	0	1.13341239	0.18067288	0.6534431	0.72789641	0	0.75735206	-0.400964	0.62949706	0.93109012	0
yieK	2.89075042	1.53144406	0.00090558	0.00204915	1	3.15008106	1.65538895	0.00103036	0.00284573	1	1.08971049	0.1239449	0.65648779	0.93664426	0
yieL	3.98521644	1.99465808	0.00062082	0.00145166	1	4.34361053	2.11889475	0.00044485	0.00133003	1	1.0899309	0.12423667	0.58871857	0.92806272	0
bglH	5.06780043	2.34135971	2.04E-06	7.91E-06	1	4.97505824	2.31471342	4.59E-05	0.00018066	1	0.98169972	-0.0266463	1	1	0
bglB	6.00932894	2.58720389	5.16E-07	2.16E-06	1	7.1578119	2.83951863	1.89E-06	1.04E-05	1	1.19111667	0.25231474	0.60286692	0.92806272	0
bglF	2.68035736	1.42242536	0.00201335	0.00422871	1	3.07477522	1.62048095	0.00258338	0.00632812	1	1.14715122	0.19805559	0.62605291	0.92989404	0
bglG	2.93130634	1.55154375	0.12909376	0.17555639	0	2.06982168	1.04950648	0.15853086	0.23044338	0	0.70610896	-0.5020373	1	1	0
phoU	0.10603857	-3.237339	2.62E-16	2.97E-15	-1	0.18098611	-2.4660491	5.47E-08	4.25E-07	-1	1.70679518	0.77128994	0.07030152	0.36820631	0
pstB	0.2852692	-1.8096041	2.65E-06	1.01E-05	-1	0.35103509	-1.5103129	0.00290115	0.00702476	-1	1.23053974	0.29929125	0.28641909	0.78944262	0
pstA	2.18435649	1.12720832	0.01221215	0.02143069	1	3.07977204	1.62282357	0.00071472	0.00203633	1	1.4099219	0.49561525	0.19966197	0.67679424	0
yaiV	2.28336327	1.1911604	0.16389264	0.21713071	0	5.28549595	2.40203885	0.06300304	0.10537867	0	2.3147854	1.21087845	0.40991819	0.87001729	0
pstC	2.40280335	1.26471858	0.00025007	0.00063922	1	3.09865712	1.63164312	0.00010489	0.0003759	1	1.2896008	0.36692454	0.36411865	0.85329623	0
pstS	1.62390493	0.69946717	0.04074065	0.06345256	0	1.8335862	0.87466809	0.0378535	0.06783237	0	1.12912164	0.17520092	0.66050393	0.93664426	0
glmS	0.18238102	-2.4549725	3.30E-14	3.14E-13	-1	0.22209934	-2.170723	2.43E-08	2.00E-07	-1	1.21777663	0.28424953	0.39210488	0.86854717	0
glmU	0.17469384	-2.5170993	2.89E-15	3.03E-14	-1	0.27577886	-1.8584162	6.15E-07	3.76E-06	-1	1.57864098	0.65868311	0.07386864	0.37957754	0
atpC	0.03359409	-4.8956487	4.35E-41	3.10E-39	-1	0.09580324	-3.3837817	2.09E-17	7.18E-16	-1	2.8517885	1.51186699	0.00010703	0.00284349	1
atpD	0.0391786	-4.6737905	7.32E-41	5.13E-39	-1	0.0932724	-3.4224059	7.06E-18	2.56E-16	-1	2.38069799	1.25138461	0.00033251	0.00699324	1
atpG	0.05312394	-4.2344942	1.79E-34	7.62E-33	-1	0.10388744	-3.2669069	1.28E-16	3.75E-15	-1	1.95556737	0.96758724	0.01009999	0.09400856	0
atpA	0.04296876	-4.5405681	5.74E-38	3.09E-36	-1	0.11427959	-3.1293603	3.91E-16	1.06E-14	-1	2.65959722	1.41120777	0.00034612	0.00716618	1
atpH	0.06350195	-3.9770554	1.94E-30	6.48E-29	-1	0.18667243	-2.4214192	3.56E-10	4.04E-09	-1	2.93963316	1.55563613	2.16E-05	0.00077001	1
atpF	0.06988857	-3.8387997	9.98E-28	2.67E-26	-1	0.20407989	-2.292794	1.49E-09	1.52E-08	-1	2.92007543	1.54600564	8.78E-05	0.00237616	1
ampH	0.53924127	-0.8909972	0.03135458	0.04996146	0	0.85582064	-0.2246196	0.78741977	0.83785408	0	1.58708297	0.66637755	0.09283105	0.44325786	0
atpE	0.09582567	-3.383444	3.59E-21	5.71E-20	-1	0.23235607	-2.1055907	7.66E-08	5.81E-07	-1	2.42477908	1.27785331	0.00187933	0.02737318	1
atpB	0.14813802	-2.7549862	1.77E-16	2.05E-15	-1	0.34394377	-1.5397554	2.13E-05	9.12E-05	-1	2.32177928	1.21523083	0.0023717	0.03193373	1
atpI	0.33977595	-1.5573443	8.89E-05	0.00025076	-1	0.79259835	-0.3353381	0.20380634	0.28348134	0	2.33270879	1.22200622	0.03237197	0.22099131	0
rsmG	0.51401104	-0.9601288	0.06809538	0.09920095	0	1.11241137	0.1536904	1	1	0	2.16417798	1.11381915	0.0996413	0.45916208	0
mnmG	0.8210856	-0.2843955	0.49410077	0.56362548	0	1.30613772	0.38530702	0.23430676	0.31887945	0	1.59074488	0.66970248	0.06149852	0.33750271	0
mioC	0.78152763	-0.3556312	0.48980993	0.55891875	0	1.46333159	0.54925672	0.725027	0.78972007	0	1.87239904	0.90488793	0.33186709	0.83208693	0
asnC	2.72167053	1.44449243	0.00145257	0.0031451	1	1.69603509	0.76216602	0.12131169	0.18327397	0	0.62315959	-0.6823264	0.22327126	0.70531966	0
asnA	0.50166932	-0.9951914	0.01203671	0.0211733	0	0.80585887	-0.3114009	0.45090969	0.54043122	0	1.60635471	0.6837905	0.13029655	0.53903168	0
viaA	0.30543501	-1.7110626	4.29E-08	2.10E-07	-1	0.2577544	-1.955931	1.22E-07	8.80E-07	-1	0.84389276	-0.2448684	0.3225655	0.82321404	0
ravA	0.05670816	-4.1402998	2.73E-33	1.08E-31	-1	0.06855386	-3.8666184	1.07E-20	5.92E-19	-1	1.20888867	0.27368139	0.466933	0.89320173	0
sbmA	2.41829857	1.27399237	0.04455239	0.06873351	0	3.67858214	1.87914981	0.04484787	0.07845713	0	1.52114474	0.60515743	0.66651942	0.93840962	0
kup	1.56330323	0.64459764	0.03558693	0.05609832	0	2.00894404	1.00643738	0.00713154	0.01562432	1	1.28506357	0.36183973	0.30550872	0.81005169	0
rbsD	0.17194317	-2.5399963	3.67E-15	3.80E-14	-1	0.21942371	-2.1882086	3.27E-10	3.75E-09	-1	1.27614089	0.35178762	0.96900718	1	0
rbsA	0.51219734	-0.9652283	0.00387736	0.00759145	0	0.5055	-0.984217	0.01178155	0.02424904	0	0.98692431	-0.0189887	0.90024866	0.99683105	0
rbsC	0.24573064	-2.0248503	1.14E-09	6.84E-09	-1	0.24165906	-2.048955	3.94E-07	2.53E-06	-1	0.9834307	-0.0241047	0.97155569	1	0
rbsB	0.06465065	-3.9511914	7.47E-32	2.80E-30	-1	0.06636605	-3.9134108	2.23E-22	1.72E-20	-1	1.02653339	0.03778055	1	1	0
rbsK	0.1999873	-2.3220197	1.97E-11	1.45E-10	-1	0.24836654	-2.0094573	1.52E-06	8.61E-06	-1	1.24191152	0.31256239	0.36944363	0.85484012	0
rbsR	0.34493282	-1.5356127	0.00038191	0.00093301	-1	0.43353279	-1.205787	0.02250547	0.04277491	-1	1.25686152	0.3298257	0.42084631	0.87375001	0
hsrA	0.5811671	-0.7829751	0.01531686	0.02624305	0	0.53405813	-0.9049313	0.03700032	0.06649224	0	0.91894075	-0.1219563	0.88463947	0.9934454	0
yieP	1.13555751	0.18340077	0.70720551	0.75690011	0	0.39551104	-1.3382101	8.27E-06	3.99E-05	-1	0.34829679	-1.5216109	9.63E-07	6.16E-05	-1
rrsC	18.6778354	4.22325536	0.11008883	0.15216405	0	27.5003779	4.78137954	0.07493861	0.12260411	0	1.47235358	0.55812417	0.71787013	0.95355641	0
yaiW	2.19792735	1.1361437	0.00162096	0.00347057	1	1.48662766	0.57204336	0.23072912	0.31556545	0	0.67637707	-0.5641003	0.13741691	0.55750561	0
rrlC	8.13303436	3.02379371	5.99E-07	2.49E-06	1	15.5658229	3.96030994	1.83E-09	1.84E-08	1	1.91390103	0.93651623	0.07949609	0.39838382	0
rrfC	0	#NAME?	1	1	0	0	#NAME?	1	1	0	NA	NA	NA	NA	0
aspT	0	#NAME?	1	1	0	0	#NAME?	1	1	0	NA	NA	NA	NA	0
trpT	0.86731624	-0.20537	0.99562966	1	0	3.39384666	1.76292138	0.40367958	0.49455505	0	3.91304406	1.96829135	0.34493472	0.84269271	0
hdfR	0.41213077	-1.2788259	8.12E-05	0.00023262	-1	0.62016559	-0.6892746	0.02297153	0.04354764	0	1.50477867	0.5895513	0.32520111	0.82473426	0
yifE	0.04598656	-4.442644	1.41E-34	6.09E-33	-1	0.08351237	-3.5818664	5.61E-18	2.12E-16	-1	1.81601689	0.86077762	0.05942152	0.3301384	0
yifB	1.92431009	0.9443413	0.0183294	0.03077193	0	2.10274011	1.07227055	0.00999008	0.02104202	1	1.09272415	0.12792925	0.55194943	0.92038542	0
ilvL	0.88466288	-0.1768003	0.66518046	0.7199466	0	0.73059454	-0.4528571	0.14733235	0.21644337	0	0.82584514	-0.2760568	0.24891387	0.74826869	0
ilvX	0.60737096	-0.7193502	0.588665	0.65182585	0	1.14101757	0.19032101	0.94598509	0.96267854	0	1.87861727	0.90967117	0.53915989	0.91897962	0
yaiY	2.19026332	1.13110432	0.08462149	0.12030033	0	2.24983588	1.16981976	0.17935389	0.25571553	0	1.02719881	0.03871544	0.96553644	1	0
ilvG	15.3744623	3.94246405	1.18E-14	1.17E-13	1	8.39982659	3.07035955	4.33E-06	2.19E-05	1	0.54634929	-0.8721045	0.03152685	0.21686982	0
ilvM	0.57812444	-0.790548	0.24636027	0.31030245	0	0.50867907	-0.9751724	0.23519256	0.31969115	0	0.87987817	-0.1846243	0.77969692	0.9635071	0
ilvE	0.58310639	-0.778169	0.01750604	0.0295611	0	0.63104439	-0.6641866	0.15373417	0.22457894	0	1.08221141	0.11398236	0.6238368	0.92989404	0
ilvD	0.87862577	-0.1866793	0.8889563	0.9135691	0	1.04440083	0.06267551	0.7412023	0.80043328	0	1.18867539	0.24935479	0.55648591	0.9219019	0
ilvA	0.71414154	-0.4857181	0.07950266	0.11371867	0	0.91457174	-0.1288318	0.73219769	0.79463591	0	1.28065893	0.3568863	0.30566759	0.81005169	0
ilvY	0.57365002	-0.8017573	0.02340437	0.03840159	0	0.65821466	-0.6033699	0.35940322	0.45080977	0	1.14741505	0.19838734	0.36487783	0.85342842	0
ilvC	0.64407456	-0.6347004	0.06580552	0.09640563	0	0.9337611	-0.0988746	0.73818224	0.7987491	0	1.44977175	0.53582578	0.19760015	0.67551679	0
ppiC	0.29569649	-1.757811	6.47E-05	0.00018962	-1	0.4031587	-1.3105802	0.00411985	0.00958695	-1	1.36342064	0.44723073	0.60644604	0.92806272	0
yifN	0.6915842	-0.5320232	0.20696724	0.26644544	0	0.57523229	-0.7977834	0.15623051	0.22763276	0	0.83176031	-0.2657602	0.62514561	0.92989404	0
rep	1.38996081	0.47504421	0.10738726	0.14887933	0	1.63331884	0.70780645	0.04315496	0.07603738	0	1.17508266	0.23276224	0.45759617	0.88755478	0
yaiZ	1.13508158	0.18279599	0.9462148	0.96078868	0	1.13336615	0.18061402	0.77554551	0.82801054	0	0.99848872	-0.002182	0.68479593	0.943596	0
gpp	0.55847609	-0.8404326	0.00580038	0.01091154	0	0.52755191	-0.922615	0.0115257	0.02378892	0	0.94462757	-0.0821825	0.75120007	0.95683847	0
rhlB	1.98571539	0.98965886	0.0036051	0.00711198	0	0.67096059	-0.5757001	0.01579943	0.03132242	0	0.33789363	-1.5653589	1.21E-06	7.12E-05	-1
trxA	0.13299404	-2.9105665	4.29E-19	5.98E-18	-1	0.13583116	-2.8801136	9.98E-15	2.20E-13	-1	1.02133272	0.03045293	0.53346344	0.9186153	0
rho	0.28159971	-1.8282823	9.60E-09	5.13E-08	-1	0.64739105	-0.6272907	0.04832834	0.08348825	0	2.29897629	1.20099159	0.00218252	0.03065257	1
wecA	0.20156085	-2.3107126	1.57E-10	1.04E-09	-1	0.34676308	-1.5279778	0.00032687	0.00101647	-1	1.72038903	0.78273483	0.04325736	0.26944204	0
wzzE	0.26321226	-1.9257014	1.03E-07	4.76E-07	-1	0.35568667	-1.4913212	0.00039009	0.00118719	-1	1.35133018	0.43438022	0.30748678	0.81011621	0
wecB	0.47467096	-1.0750003	0.00304552	0.00612693	-1	0.68843525	-0.5386071	0.23243499	0.31721131	0	1.45034202	0.53639316	0.17334485	0.63021498	0
wecC	0.61617623	-0.6985851	0.03182029	0.05064873	0	0.66083486	-0.5976383	0.20834556	0.28915799	0	1.07247704	0.10094677	0.62100682	0.92938245	0
rffG	0.56445231	-0.8250764	0.02385811	0.03903009	0	0.59929142	-0.7386704	0.1048314	0.16267795	0	1.06172197	0.08640602	0.77815848	0.9635071	0
rffH	0.82990307	-0.2689853	0.42780478	0.4998978	0	0.66014768	-0.5991393	0.2361871	0.32084562	0	0.79545154	-0.330154	0.5611837	0.92432422	0
ddlA	48.6565927	5.60456339	7.98E-30	2.52E-28	1	13.3594047	3.73978382	9.94E-10	1.05E-08	1	0.27456515	-1.8647796	1.49E-05	0.00058038	-1
wecD	0.63440818	-0.6565167	0.12655724	0.17226605	0	0.45701236	-1.1296949	0.04737613	0.08210004	0	0.7203759	-0.4731782	0.45650394	0.88755478	0
wecE	0.38596118	-1.3734724	7.23E-05	0.0002093	-1	0.46244093	-1.112659	0.01182702	0.02430866	-1	1.198154	0.26081335	0.40719854	0.86959974	0
wzxE	1.53522177	0.61844708	0.04650626	0.07137531	0	1.65994503	0.73113547	0.05344636	0.09108348	0	1.0812412	0.11268839	0.70292139	0.94896888	0
wecF	1.31128403	0.39098021	0.50705286	0.57648712	0	1.08299525	0.11502691	0.70178102	0.76933027	0	0.8259044	-0.2759533	0.74654326	0.95683847	0
wzyE	2.95761144	1.56443253	2.52E-06	9.59E-06	1	3.47795753	1.79824031	3.57E-06	1.84E-05	1	1.17593457	0.23380778	0.46508964	0.89206388	0
wecG	1.61619173	0.69259835	0.07351587	0.10615221	0	1.277509	0.35333346	0.42947586	0.52024501	0	0.79044397	-0.3392649	0.46100803	0.88879788	0
yifK	1.71346082	0.7769132	0.02023899	0.03363266	0	1.29717742	0.37537581	0.13459049	0.20045681	0	0.75705111	-0.4015374	0.60734686	0.92806272	0
argX	0.44981921	-1.1525828	0.25160307	0.31625805	0	2.00923393	1.00664554	0.6952642	0.76427176	0	4.46675878	2.15922835	0.16664114	0.61557317	0
hisR	0.3189799	-1.6484626	0.15260055	0.20375956	0	1.62969768	0.70460436	0.88068538	0.91044578	0	5.10909209	2.35306694	0.16270301	0.61065554	0
leuT	0.21814835	-2.1966185	0.03306319	0.05241927	0	1.251688	0.323875	0.91026109	0.93241627	0	5.73778339	2.52049351	0.07593923	0.38578363	0
iraP	0.05340103	-4.2269886	6.01E-29	1.79E-27	-1	0.06356049	-3.9757259	2.25E-18	9.06E-17	-1	1.19024844	0.25126274	0.6384982	0.93163024	0
proM	0.60526384	-0.7243639	0.51373996	0.58229135	0	2.11335221	1.07953322	0.58005937	0.66320372	0	3.49162146	1.80389716	0.23677123	0.72916279	0
aslB	2.14440726	1.10057893	0.00743855	0.01369574	1	2.46873324	1.30377095	0.00293131	0.00709001	1	1.15124271	0.20319203	0.48328117	0.89935549	0
aslA	2.46417853	1.30110679	0.00011426	0.00031427	1	3.07814557	1.62206146	9.54E-05	0.00034592	1	1.24915688	0.32095467	0.43550552	0.88087171	0
glmZ	0.04507633	-4.471486	1.47E-34	6.31E-33	-1	0.10763041	-3.2158424	2.19E-18	8.86E-17	-1	2.38773641	1.25564358	0.08530647	0.41707487	0
hemY	0.33191534	-1.5911128	3.33E-07	1.42E-06	-1	0.24553128	-2.0260213	1.21E-07	8.76E-07	-1	0.73974067	-0.4349085	0.19315634	0.66866033	0
hemX	0.31482815	-1.6673636	6.61E-08	3.13E-07	-1	0.24536214	-2.0270155	1.73E-07	1.21E-06	-1	0.77935261	-0.3596519	0.32824226	0.8290655	0
hemD	0.48784164	-1.0355152	0.00186304	0.00393734	-1	0.4676417	-1.0965245	0.0122819	0.0250687	-1	0.95859324	-0.0610093	0.96344729	1	0
hemC	0.25735438	-1.9581717	8.98E-10	5.49E-09	-1	0.2926989	-1.7725108	8.80E-06	4.20E-05	-1	1.13733794	0.18566099	0.49581754	0.90539057	0
cyaA	0.53323287	-0.9071624	0.00198097	0.00416863	0	0.47775295	-1.0656633	0.00275617	0.00671048	-1	0.89595555	-0.1585009	0.58417446	0.92806272	0
cyaY	0.61369123	-0.7044151	0.09423695	0.13230916	0	0.66517817	-0.5881873	0.31529754	0.40541094	0	1.08389713	0.11622784	0.72685672	0.95487077	0
phoA	0.48660686	-1.0391714	0.00207626	0.0043505	-1	0.47516092	-1.0735119	0.03502058	0.06339858	0	0.97647806	-0.0343405	0.70037327	0.94869712	0
yifL	0.13846589	-2.8523975	1.66E-14	1.61E-13	-1	0.19978483	-2.3234811	1.68E-08	1.43E-07	-1	1.44284505	0.52891637	0.3973192	0.86854717	0
dapF	0.31995769	-1.6440469	3.31E-07	1.42E-06	-1	0.41312978	-1.275333	0.00167265	0.00434024	-1	1.29120129	0.36871393	0.25038316	0.7487007	0

yigA	0.411342	-1.2815897	0.00040012	0.00097374	-1	0.43159413	-1.2122528	0.01113745	0.02309545	-1	1.0492343	0.06933688	0.65427294	0.93589534	0
xerC	0.58712961	-0.7682491	0.01580432	0.02699447	0	0.50854168	-0.9755621	0.03105173	0.05705514	0	0.86614892	-0.207313	0.80839697	0.96990832	0
yigB	0.55852947	-0.8402947	0.05552515	0.08325003	0	0.50937845	-0.9731902	0.06102587	0.10257638	0	0.91199924	-0.1328955	0.85161635	0.98212032	0
uvrD	0.47710551	-1.0676197	0.00286748	0.0058126	-1	0.62041128	-0.6887032	0.1328443	0.198391	0	1.30036493	0.37891655	0.2563466	0.75668576	0
yigE	1.80813379	0.85450143	0.0738849	0.10662114	0	1.75167203	0.80873268	0.07520609	0.1229962	0	0.96877346	-0.0457688	0.80120076	0.96905452	0
corA	0.42245428	-1.2431329	0.00236309	0.00486836	-1	0.74240271	-0.4297261	0.49829945	0.58579018	0	1.75735635	0.81340676	0.0385841	0.2487659	0
yigF	0.35171888	-1.5075053	0.0672584	0.09837098	0	1.21779995	0.28427716	0.97249649	0.98407648	0	3.46242414	1.79178246	0.10575073	0.4783187	0
yigG	2.79861222	1.4847116	0.2978462	0.36574553	0	2.00950052	1.00683695	0.28830562	0.37771198	0	0.71803464	-0.4778746	1	1	0
caiT	6.72267259	2.74903489	1.10E-05	3.72E-05	1	6.98662509	2.80459573	1.18E-05	5.44E-05	1	1.03926303	0.05556084	0.62386259	0.92989404	0
psiF	1.86170076	0.8966212	0.14804905	0.19834172	0	0.41650007	-1.2636113	0.09392317	0.14849606	0	0.2237202	-2.1602325	0.00388925	0.04537455	-1
rarD	1.63287399	0.70741346	0.05263812	0.07956966	0	2.428869	1.28028468	0.01076713	0.02241167	1	1.48748098	0.57287123	0.28464679	0.78899581	0
yigI	4.39278468	2.13513579	1.22E-05	4.09E-05	1	4.70275705	2.2335068	5.87E-05	0.00022371	1	1.07056398	0.09837102	0.77450937	0.96287211	0
pldA	0.60646831	-0.7214958	0.0376484	0.05911588	0	1.20503892	0.26907974	0.79990175	0.8466505	0	1.98697756	0.99057558	0.04143548	0.26141785	0
recQ	1.11810724	0.16105857	0.43427308	0.5057169	0	1.96834676	0.9769844	0.01255458	0.02556622	0	1.76042753	0.81592584	0.04513342	0.27721225	0
rhtC	0.96183007	-0.0561461	0.79806218	0.83670559	0	0.91977716	-0.1206437	1	1	0	0.95627823	-0.0644977	0.8499206	0.98212032	0
rhtB	1.20663051	0.27098397	0.63345279	0.69170852	0	1.53085383	0.61433654	0.11026218	0.16918353	0	1.26870141	0.34335257	0.25776206	0.75883223	0
pldB	0.39388134	-1.344167	0.00017271	0.00045873	-1	0.52171949	-0.9386538	0.02785863	0.05166063	0	1.32456005	0.40551325	0.31169276	0.81168136	0
yigL	0.4711169	-1.085843	0.00307316	0.00616725	-1	0.52724793	-0.9234466	0.06652344	0.1105555	0	1.1191446	0.16239645	0.51256519	0.90925684	0
yigM	1.46650644	0.5523834	0.16530742	0.21874262	0	1.57180195	0.65241945	0.08855528	0.14112002	0	1.07180024	0.10003604	0.57599259	0.92806272	0
yaiC	4.02159954	2.00776943	0.00023358	0.00060158	1	4.40707849	2.13982259	0.0004094	0.00123717	1	1.09585215	0.13205316	0.66372686	0.93720148	0
metE	3.11638524	1.63987359	0.32486683	0.39421341	0	2.60793355	1.38290711	0.33984929	0.43031347	0	0.83684569	-0.2569665	1	1	0
ysgA	4.62536099	2.20956597	0.00148005	0.0031999	1	1.02811308	0.03999896	0.76688446	0.82117105	0	0.22227737	-2.169567	0.01366164	0.11800874	0
udp	0.01156918	-6.4335699	3.75E-63	7.53E-61	-1	0.0220114	-5.505605	3.51E-36	1.72E-33	-1	1.90259026	0.9279649	0.02065745	0.15982343	0
rmuC	0.30255988	-1.7247074	7.42E-07	3.04E-06	-1	0.52204329	-0.9377587	0.02456245	0.04618785	0	1.72542138	0.78694874	0.03797575	0.24556165	0
ubiE	0.20392261	-2.2939063	3.68E-12	2.90E-11	-1	0.31406784	-1.6708519	1.63E-05	7.20E-05	-1	1.54013248	0.62305446	0.11207746	0.49574884	0
ubiJ	0.32844628	-1.6062707	2.50E-05	7.92E-05	-1	0.61134158	-0.7099494	0.07753769	0.12610933	0	1.86131379	0.89632129	0.06094627	0.33554689	0
ubiB	0.22126375	-2.176161	7.32E-11	5.04E-10	-1	0.23692874	-2.0774749	2.05E-07	1.42E-06	-1	1.07079781	0.09868609	0.84653701	0.98157978	0
tatA	0.04815311	-4.3762273	5.54E-31	1.99E-29	-1	0.08333424	-3.5849468	1.65E-18	7.00E-17	-1	1.73060981	0.79128048	0.25984357	0.75892335	0
tatB	0.12012338	-3.0574111	2.00E-18	2.74E-17	-1	0.18395791	-2.4425524	1.22E-09	1.26E-08	-1	1.53140809	0.61485878	0.15490169	0.59433748	0
proC	0.41935523	-1.2537552	0.00059089	0.00138969	-1	0.43698716	-1.1943372	0.00619109	0.01375486	-1	1.04204532	0.05941802	0.86336267	0.98510462	0
tatC	0.27011688	-1.8883443	6.08E-08	2.90E-07	-1	0.4284093	-1.2229383	0.00315115	0.0075433	-1	1.58601451	0.66540597	0.0943742	0.44848086	0
tatD	0.8150214	-0.2950902	0.45403716	0.52430905	0	1.02103315	0.03002971	0.7792564	0.83096864	0	1.25276852	0.32511986	0.35218492	0.84775072	0
rfaH	0.63183237	-0.6623863	0.18173785	0.23756616	0	0.82267166	-0.2816113	0.72935741	0.79253185	0	1.30204102	0.3807749	0.42247907	0.87468118	0
ubiD	0.19494039	-2.358895	1.16E-12	9.67E-12	-1	0.27387282	-1.868422	1.07E-06	6.29E-06	-1	1.40490548	0.49047307	0.23239831	0.72276201	0
fre	0.22994103	-2.1206642	3.45E-10	2.24E-09	-1	0.27939363	-1.839629	2.59E-06	1.40E-05	-1	1.21506644	0.28103521	0.55250054	0.92069223	0
fadA	2.96428361	1.56768348	0.00011748	0.00032271	1	3.05611113	1.611697	0.00015038	0.00051674	1	1.03097798	0.04401352	0.65894192	0.93664426	0
fadB	12.4776902	3.64127899	3.41E-24	6.91E-23	1	4.77152597	2.25445072	1.38E-09	1.42E-08	1	0.38240459	-1.3868283	0.00126384	0.02012098	-1
pepQ	0.21442717	-2.2214404	1.24E-11	9.30E-11	-1	0.18971206	-2.3981167	1.86E-09	1.88E-08	-1	0.88473889	-0.1766764	0.65930577	0.93664426	0
yigZ	0.43133698	-1.2131127	0.00078713	0.00180715	-1	0.46710673	-1.0981759	0.01200568	0.02459578	-1	1.08292761	0.1149368	0.75595104	0.9573569	0
trkH	0.95977058	-0.0592385	0.90034794	0.92248593	0	1.00597023	0.00858762	0.73944404	0.79881412	0	1.04813614	0.06782612	0.75161029	0.95683847	0
yaiI	0.44364231	-1.1725311	0.00410869	0.0079842	-1	0.85191848	-0.2312127	0.49577141	0.58343912	0	1.92028232	0.94131843	0.07805596	0.39270146	0
hemG	0.60966378	-0.7139143	0.04135133	0.06429033	0	0.62888851	-0.6691238	0.16664634	0.24112779	0	1.03153333	0.04479044	0.8095605	0.96990832	0
rrsA	1.57845628	0.6585143	0.95422349	0.96803058	0	16.7434777	4.06552731	0.54421476	0.62938629	0	10.6075017	3.40701301	0.63156996	0.93151288	0
rrlA	3.14919158	1.65498153	1.30E-05	4.34E-05	1	10.9002563	3.44629016	1.05E-14	2.31E-13	1	3.46128714	1.79130863	1.65E-05	0.00063404	1
mobB	0.77479348	-0.3681163	0.32400448	0.39338312	0	0.63177827	-0.6625098	0.68692812	0.75774655	0	0.81541505	-0.2943935	0.78474898	0.9635071	0
mobA	0.695862	-0.5231269	0.06080218	0.0901219	0	0.72543346	-0.4630848	0.42558655	0.51638303	0	1.04249615	0.06004206	0.56048148	0.92432422	0
yihD	0.07022575	-3.8318561	4.64E-22	7.97E-21	-1	0.07060646	-3.8240559	3.19E-15	7.71E-14	-1	1.00542131	0.00780017	0.96325553	1	0
srkA	0.47990704	-1.0591731	0.0046741	0.00894907	-1	0.51877864	-0.946809	0.02762338	0.05126742	0	1.0809982	0.11236412	0.84972475	0.98212032	0
aroL	1.0274353	0.03904755	0.9503187	0.96473401	0	0.90594856	-0.142499	0.52357024	0.61078588	0	0.88175728	-0.1815465	0.51458444	0.90937129	0
dsbA	12.7208976	3.66912857	1.14E-08	6.04E-08	1	7.03674578	2.81490839	0.00237735	0.00592526	1	0.55316425	-0.8542202	0.06979563	0.36774043	0
yihF	6.64482171	2.73223049	2.92E-06	1.10E-05	1	4.54253474	2.18349755	0.00075093	0.00213261	1	0.68362026	-0.5487329	0.44104483	0.88087171	0
yihG	2.42323988	1.27693723	0.00757376	0.01390989	1	2.88776549	1.52995359	0.00200892	0.00509903	1	1.19169609	0.25301636	0.4257007	0.87726173	0
polA	0.16457757	-2.6031604	4.48E-15	4.60E-14	-1	0.14957739	-2.741036	1.00E-11	1.39E-10	-1	0.90885647	-0.1378756	0.73466421	0.95650847	0
spf	0.10436612	-3.2602746	5.45E-08	2.63E-07	-1	0.18817993	-2.4098153	0.00060076	0.0017419	-1	1.80307488	0.85045931	0.23439756	0.72641829	0
yihA	0.16396741	-2.608519	1.12E-13	1.01E-12	-1	0.21501468	-2.2174929	3.80E-08	3.01E-07	-1	1.3113257	0.39102606	0.47912503	0.89894957	0
csrC	0.40989932	-1.2866585	5.40E-06	1.94E-05	-1	0.19175228	-2.3826843	1.51E-12	2.40E-11	-1	0.46780337	-1.0960259	5.04E-05	0.0015544	-1
yihI	0.53893538	-0.8918158	0.01092713	0.01937581	0	0.25231349	-1.9867108	9.09E-06	4.32E-05	-1	0.46817021	-1.094895	0.01203163	0.10710485	0
hemN	0.48702795	-1.0379235	0.00140829	0.00305878	-1	0.46467983	-1.1056911	0.00361569	0.0085534	-1	0.95441327	-0.0677675	0.75897281	0.95807677	0
yshB	0.17064088	-2.5509648	1.23E-08	6.49E-08	-1	0.41633217	-1.2641931	0.00363578	0.00858712	-1	2.43981497	1.28677174	0.04383211	0.27110746	0
yaiA	2.26661808	1.18054132	0.03275103	0.05198035	0	0.86838872	-0.2035871	0.31228752	0.40256668	0	0.38312088	-1.3841284	0.00438879	0.04955387	-1
glnG	0.37719576	-1.4066146	2.57E-05	9.26E-05	-1	1.23003123	0.29869494	0.5484472	0.63279065	0	3.26098899	1.70530957	1.50E-05	0.00058038	1
glnL	0.48666847	-1.0389888	0.01195604	0.02104816	-1	1.53414604	0.61743583	0.11854605	0.17952536	0	3.15234323	1.65642463	8.76E-05	0.00237616	1
glnA	0.11345583	-3.1397974	2.41E-21	3.96E-20	-1	0.70906207	-0.4960162	0.07835452	0.1272505	0	6.24967511	2.64378119	2.23E-12	6.15E-10	1
typA	0.20377746	-2.2949336	3.32E-12	2.63E-11	-1	0.45728398	-1.1288377	0.00147413	0.00389605	-1	2.24403613	1.1660959	0.002916	0.03727414	1
yihL	1.49497777	0.58012403	0.33655024	0.40649232	0	1.39213701	0.47730121	0.31101321	0.40139234	0	0.93120917	-0.1028228	0.85116882	0.98212032	0
yihM	0.37371501	-1.4199896	0.00021302	0.00055316	-1	0.37524348	-1.4141011	0.00716984	0.0156976	-1	1.00408995	0.00588851	0.72833319	0.95488877	0
yihN	0.85276302	-0.2297832	0.55854694	0.62410873	0	1.28747498	0.36454439	0.29693562	0.38649735	0	1.50976878	0.59432762	0.12091648	0.51922266	0
ompL	4.40247717	2.13831552	0.00016089	0.00042941	1	4.16856017	2.05954916	0.00086519	0.00242097	1	0.94686696	-0.0787664	0.95911419	1	0
yihO	3.81248629	1.93073215	0.000175	0.00046369	1	4.19841689	2.06984543	0.00045808	0.00136588	1	1.10122806	0.13911328	0.74446238	0.95683847	0
yihP	5.65793408	2.50027537	0.0061872	0.01158002	1	5.18503328	2.37435325	0.01135619	0.02351593	1	0.91641811	-0.1259221	0.88612158	0.99349171	0
aroM	2.35887481	1.23809885	0.00713268	0.01317023	1	2.47992041	1.31029382	0.03884383	0.06940436	0	1.05131498	0.07219497	0.88696307	0.99377722	0
yihQ	4.77721805	2.25617073	5.68E-05	0.00016852	1	7.1518187	2.83831017	8.74E-06	4.17E-05	1	1.49706767	0.58213944	0.27767585	0.77996846	0
yihR	5.30461364	2.40724768	0.00026485	0.00067192	1	5.29912223	2.40575341	0.00050994	0.0014973	1	0.99896479	-0.0014943	0.75071991	0.95683847	0
yihS	2.57830844	1.36642486	0.00030253	0.00075496	1	3.91956442	1.97069334	1.36E-05	6.18E-05	1	1.52020773	0.60426847	0.15384021	0.59217223	0
yihT	2.10484394	1.07371327	0.01350542	0.02352265	1	2.08580743	1.06060597	0.0189359	0.03676526	1	0.99095585	-0.0131073	0.77916568	0.9635071	0
yihU	1.35132299	0.43437255	0.3019515	0.36996387	0	2.02825477	1.02023888	0.10550811	0.16347838	0	1.50094003	0.58586634	0.38395982	0.86171135	0
yihV	1.32428706	0.40521588	0.2849204	0.35212462	0	1.98387779	0.98832316	0.0544997	0.09255733	0	1.49807232	0.58310727	0.26093233	0.75954558	0
yihW	0.55131613	-0.8590483	0.06822589	0.09932557	0	0.7369588	-0.4403441	0.43373808	0.52454531	0	1.33672637	0.41870417	0.34758297	0.84560166	0
yihX	0.1733262	-2.5284384	2.51E-13	2.20E-12	-1	0.18994889	-2.3963168	1.87E-08	1.57E-07	-1	1.09590411	0.13212157	0.62054314	0.92923437	0
yihY	0.24419053	-2.0339208	4.36E-08	2.13E-07	-1	0.22935556	-2.1243422	1.03E-06	6.04E-06	-1	0.93924836	-0.0904214	0.73852783	0.95683847	0
dtd	0.46354213	-1.1092276	0.00418098	0.0081104	-1	0.28138114	-1.8294025	0.00011398	0.00040294	-1	0.60702387	-0.7201749	0.12276968	0.52159375	0
yaiE	0.11535253	-3.1158784	2.93E-17	3.63E-16	-1	0.14856114	-2.7508713	2.84E-11	3.74E-10	-1	1.28788797	0.36500711	0.68079756	0.94335731	0
yiiD	0.56157485	-0.8324498	0.01256613	0.02197891	0	0.52985808	-0.9163221	0.03660384	0.06588691	0	0.94352175	-0.0838723	0.9688246	1	0
yiiE	5.12839174	2.35850647	0.01634084	0.02779264	1	2.95460241	1.56296401	0.07904409	0.12813493	0	0.5761265	-0.7955425	0.70881606	0.95069307	0
yiiF	0.46274986	-1.1116956	0.20180656	0.26071353	0	0.6773396	-0.5620488	0.75969699	0.8156222	0	1.4637273	0.54964679	0.42844207	0.87784805	0
fdhE	0.21662589	-2.2067225	1.33E-10	8.93E-10	-1	0.21994294	-2.1847988	8.09E-08	6.10E-07	-1	1.01531236	0.02192365	0.98982532	1	0
fdoI	0.19738063	-2.3409477	9.06E-13	7.63E-12	-1	0.15320426	-2.7064717	1.30E-11	1.77E-10	-1	0.7761869	-0.365524	0.23275511	0.72303736	0
fdoH	0.17101012	-2.5478464	1.92E-14	1.85E-13	-1	0.11327378	-3.1421141	3.41E-14	6.95E-13	-1	0.66238058	-0.5942677	0.11183692	0.49567923	0
fdoG	0.17832525	-2.4874171	2.55E-15	2.71E-14	-1	0.09647887	-3.3736431	4.05E-17	1.30E-15	-1	0.54102756	-0.886226	0.01439194	0.12205469	0
fdhD	0.49944856	-1.001592	0.01896837	0.03167611	-1	0.58179098	-0.7814272	0.13333526	0.19890539	0	1.16486666	0.22016482	0.60467343	0.92806272	0
yiiG	3.31586076	1.72938343	0.04799532	0.07337066	0	2.74960573	1.45922477	0.08255437	0.13319013	0	0.82922835	-0.2701587	0.99883723	1	0
frvR	4.76351336	2.25202604	0.00012617	0.00034274	1	5.00414895	2.32312473	0.00025156	0.00080964	1	1.05051641	0.0710987	0.72553522	0.95459348	0
ykiA	0.61517186	-0.7009386	0.28332474	0.35034864	0	0.76555107	-0.3854295	0.86677442	0.89891484	0	1.24445072	0.3155091	0.53440147	0.9187668	0
frvX	3.0416671	1.60486226	0.00042698	0.00103002	1	3.93384142	1.9759388	9.00E-05	0.00032757	1	1.29331754	0.37107654	0.33355507	0.83424743	0
frvB	5.97902295	2.57990975	7.26E-05	0.00021008	1	8.23137357	3.04113319	3.16E-05	0.00012951	1	1.37670881	0.46122344	0.41461715	0.87131399	0
frvA	8.13437238	3.02403104	4.11E-05	0.00012518	1	6.22638073	2.6383938	0.0004066	0.0012298	1	0.76544083	-0.3856372	0.86848133	0.98621446	0
rhaM	1.57138588	0.6520375	0.28842747	0.35564685	0	3.89880419	1.9630317	0.06051554	0.10179604	0	2.48112462	1.3109942	0.25655106	0.75678273	0
rhaD	6.45362958	2.69011077	0.00060882	0.00142737	1	5.99483319	2.58371961	0.00119765	0.00324689	1	0.92890878	-0.1063912	0.85827295	0.98413513	0
rhaA	4.99400395	2.32019696	2.32E-06	8.92E-06	1	6.12837714	2.61550508	1.84E-06	1.02E-05	1	1.22714703	0.29530812	0.44425669	0.88410289	0
rhaB	4.91028724	2.29580742	4.49E-08	2.19E-07	1	6.28130311	2.65106389	5.13E-08	4.01E-07	1	1.27921297	0.35525647	0.40303738	0.86854717	0
rhaS	3.37131553	1.75331166	3.32E-06	1.24E-05	1	4.65093047	2.21751937	8.73E-06	4.17E-05	1	1.37955953	0.46420772	0.48571895	0.90075713	0
rhaR	1.90492635	0.92973522	0.00756914	0.01390718	0	2.0324611	1.02322774	0.0053367	0.01207378	1	1.06694996	0.09349252	0.57489935	0.92806272	0

rhaT	3.85817678	1.94791925	0.00580031	0.01091154	1	4.49042605	2.16685234	0.00589654	0.01319332	1	1.16387255	0.21893309	0.65221006	0.9347567	0
rdgC	0.40215269	-1.3141847	0.00066153	0.00153951	-1	0.67145316	-0.5746413	0.30506945	0.39510607	0	1.66964735	0.73954342	0.05566634	0.31716867	0
sodA	0.2674234	-1.9028024	3.67E-09	2.09E-08	-1	0.12785581	-2.9674103	4.33E-13	7.45E-12	-1	0.47810256	-1.064608	0.00231404	0.03169235	-1
kdgT	3.01322258	1.59130725	0.0002885	0.00072569	1	2.74601062	1.45733721	0.00387214	0.00908708	1	0.91132021	-0.13397	0.89978341	0.99683105	0
yiiM	2.07720907	1.05464643	0.00321639	0.00642588	1	1.68666154	0.7541705	0.03400932	0.06177032	0	0.81198448	-0.3004759	0.68773444	0.94389281	0
cpxA	0.49689316	-1.0089924	0.00194175	0.00409389	-1	0.48796467	-1.0351514	0.01173553	0.02417666	-1	0.98203137	-0.026159	0.96949344	1	0
cpxR	0.22414733	-2.1574808	5.69E-10	3.59E-09	-1	0.21964119	-2.1867794	1.06E-07	7.82E-07	-1	0.97989652	-0.0292987	0.84054961	0.98067952	0
cpxP	1.1712535	0.22805336	0.80245409	0.84070042	0	0.80904108	-0.3057151	0.25866229	0.34563914	0	0.69074805	-0.5337685	0.14969099	0.58470971	0
fieF	0.49572426	-1.0123902	0.01068432	0.018991	-1	0.6037328	-0.7280179	0.09907758	0.1555095	0	1.21788027	0.28437231	0.54161736	0.91914725	0
pfkA	0.06707307	-3.8981225	2.16E-30	7.12E-29	-1	0.06567108	-3.9285981	1.06E-21	6.90E-20	-1	0.97909748	-0.0304756	0.84176065	0.98067952	0
sbp	3.65736796	1.87080578	0.00078717	0.00180715	1	2.76867911	1.46919785	0.01401117	0.02809226	1	0.7570141	-0.4016079	0.61837164	0.92897598	0
cdh	1.30084517	0.37944926	0.45905067	0.52871625	0	1.79430976	0.84342897	0.07621049	0.12440864	0	1.37934152	0.46397971	0.25889146	0.75892335	0
yaaX	1.26737291	0.34184108	0.6078523	0.66938011	0	0.85946587	-0.2184877	0.8933102	0.91878132	0	0.67814758	-0.5603288	0.51988168	0.91269012	0
fixA	2.10449429	1.07347359	0.26206738	0.3282903	0	3.46177547	1.79151216	0.0433819	0.07634593	0	1.64494411	0.71803856	0.29925183	0.8042051	0
mak	0.89426401	-0.1612273	0.75314444	0.79928856	0	0.52792327	-0.9215998	0.13614647	0.20210408	0	0.59034386	-0.7603726	0.20828154	0.69006731	0
tpiA	0.0940225	-3.4108501	4.66E-25	1.03E-23	-1	0.10396104	-3.2658851	5.28E-17	1.67E-15	-1	1.10570382	0.14496499	0.89726694	0.99640384	0
yiiQ	0.99842324	-0.0022766	0.61119045	0.67221818	0	0.72083031	-0.4722684	0.3675373	0.45905803	0	0.72196869	-0.4699918	0.48304929	0.89935549	0
yiiR	1.09024169	0.12464799	0.80139372	0.83999907	0	1.37671153	0.4612263	0.39901865	0.48993149	0	1.26275811	0.33657831	0.52300953	0.91269012	0
yiiS	0.39327281	-1.3463976	2.64E-05	8.32E-05	-1	0.13167753	-2.924919	7.50E-11	9.21E-10	-1	0.33482489	-1.5785213	0.00019114	0.00441334	-1
uspD	0.36908096	-1.4379908	6.24E-06	2.22E-05	-1	0.10219081	-3.2906626	4.80E-14	9.64E-13	-1	0.27687913	-1.8526718	5.37E-06	0.00025184	-1
fpr	0.51761762	-0.9500414	0.00329676	0.00656528	0	0.3455541	-1.5330165	0.0002155	0.00070754	-1	0.66758566	-0.5829751	0.15473526	0.59433748	0
glpX	0.5142215	-0.9595382	0.00220062	0.00456988	0	0.29612626	-1.7557157	7.16E-06	3.47E-05	-1	0.57587296	-0.7961775	0.0292667	0.20617598	0
glpK	0.3134691	-1.6736049	1.95E-08	9.91E-08	-1	0.09179127	-3.4454992	3.80E-18	1.47E-16	-1	0.292824	-1.7718943	1.05E-06	6.55E-05	-1
glpF	0.33128374	-1.5938607	8.48E-08	3.95E-07	-1	0.15046366	-2.732513	3.05E-13	5.39E-12	-1	0.45418366	-1.1386523	0.00057808	0.01092063	-1
zapB	0.10049748	-3.3147688	7.45E-24	1.46E-22	-1	0.1621831	-2.6243046	9.32E-14	1.79E-12	-1	1.61380274	0.69046424	0.37458088	0.85857675	0
araJ	1.91701887	0.93886454	0.01118112	0.01977061	0	2.29798453	1.20036909	0.0104228	0.02180793	1	1.19872817	0.26150455	0.59829134	0.92806272	0
rraA	0.10819129	-3.2083438	2.92E-21	4.73E-20	-1	0.11492525	-3.1212322	3.30E-15	7.81E-14	-1	1.06224133	0.08711157	0.89787032	0.99640384	0
menA	1.02292126	0.0326951	0.85332379	0.88352817	0	1.48907793	0.57441926	0.18729256	0.2652711	0	1.45571119	0.54172416	0.22196036	0.70531966	0
hslU	0.52683436	-0.9245786	0.00442852	0.00853701	0	0.5323337	-0.9095972	0.02230061	0.04247689	0	1.01043846	0.01498146	0.94428188	1	0
hslV	0.76964139	-0.3777417	0.34414128	0.41373762	0	1.09573387	0.13189745	0.99913834	1	0	1.42369406	0.50963916	0.39346395	0.86854717	0
ftsN	0.49695742	-1.0088059	0.00113437	0.00251279	-1	0.73462652	-0.4449171	0.19554705	0.27467336	0	1.47824843	0.56388875	0.16503132	0.61352961	0
cytR	0.47658214	-1.0692032	0.00210802	0.00440592	-1	0.79658898	-0.3280926	0.38045028	0.47173225	0	1.67146208	0.74111063	0.07370242	0.37957754	0
priA	1.33119544	0.41272239	0.27317151	0.33950438	0	1.67320391	0.74261327	0.04353785	0.07652894	0	1.2569183	0.32989088	0.27944974	0.78196278	0
rpmE	0.021834	-5.5172794	4.08E-40	2.65E-38	-1	0.10873779	-3.2010747	3.21E-15	7.71E-14	-1	4.98020378	2.31620478	7.56E-06	0.00032671	1
yiiX	1.13014297	0.17650529	0.78141213	0.82335338	0	1.21015484	0.27519165	0.51698498	0.60437876	0	1.07079801	0.09868636	0.7107893	0.95223057	0
metJ	0.27591804	-1.8576883	1.22E-06	4.87E-06	-1	0.2533584	-1.9807484	5.72E-06	2.84E-05	-1	0.91823789	-0.1230601	0.51749037	0.91030416	0
sbcC	1.1819567	0.24117719	0.5807856	0.64471726	0	1.56343199	0.64471646	0.07569059	0.12369702	0	1.32274895	0.40353927	0.20006645	0.67712436	0
metB	1.48953514	0.57486216	0.05906258	0.08786451	0	1.87523868	0.90707423	0.01835528	0.03579523	0	1.25894222	0.33221207	0.37434483	0.85857675	0
metL	0.63088093	-0.6645604	0.05711854	0.0854071	0	1.18003435	0.23882886	0.55065448	0.63500577	0	1.87045496	0.90338923	0.02106328	0.1618276	0
metF	2.40662626	1.26701211	0.00947063	0.01699788	1	2.75207217	1.4605183	0.00723467	0.01581109	1	1.14353949	0.19350619	0.60088911	0.92806272	0
katG	0.15353565	-2.7033544	1.53E-16	1.79E-15	-1	0.12005566	-3.0582247	7.34E-14	1.44E-12	-1	0.78193994	-0.3548703	0.44718665	0.88755478	0
yijE	1.48790941	0.57328669	0.05534956	0.08304314	0	1.92670465	0.94613543	0.01444031	0.02887409	0	1.29490722	0.37284874	0.33139861	0.83208693	0
yijF	3.75572588	1.90909177	0.00133622	0.00291749	1	4.4988134	2.16954453	0.00029376	0.00092723	1	1.19785457	0.26045276	0.41489704	0.87131399	0
gldA	0.06606895	-3.9198839	2.12E-30	7.03E-29	-1	0.07097735	-3.8164976	1.24E-19	5.89E-18	-1	1.07429209	0.1033863	0.62336625	0.92989404	0
fsaB	0.28590956	-1.8063692	1.93E-05	6.22E-05	-1	0.37625295	-1.4102252	0.01010419	0.0212621	-1	1.31598589	0.39614403	0.27329815	0.77568242	0
frwA	0.69674167	-0.5213042	0.19124021	0.24837049	0	0.90449301	-0.1448187	0.80133173	0.84796095	0	1.29817557	0.37648551	0.16247396	0.61031528	0
frwC	2.25330501	1.17204261	0.01614299	0.0275091	1	2.32676486	1.21832542	0.0201065	0.03876555	1	1.03260094	0.04628281	0.75776829	0.95806023	0
sbcD	1.24610809	0.31742921	0.27244321	0.33878988	0	1.20372016	0.26750003	0.45143575	0.54091502	0	0.96598374	-0.0499292	0.93662587	1	0
frwB	0.46145102	-1.1157506	0.17095758	0.22527435	0	0.7470294	-0.4207631	0.79970801	0.84664822	0	1.61887038	0.69498748	0.30749884	0.81011621	0
pflD	0.73542117	-0.4433574	0.21686182	0.27772649	0	0.82746017	-0.2732382	0.86363985	0.89629718	0	1.12515142	0.17011916	0.40610344	0.86959974	0
pflC	2.03748049	1.02678624	0.03889194	0.06091691	0	2.27909547	1.18846136	0.01048225	0.0219012	1	1.11858517	0.16167511	0.44929041	0.88755478	0
frwD	2.01884645	1.01353119	0.29497158	0.36288563	0	3.22644854	1.68994701	0.03207733	0.05870576	0	1.5981644	0.67641583	0.23990263	0.73317437	0
yijO	1.12725086	0.17280862	0.4837819	0.55345031	0	1.39369199	0.47891176	0.20032757	0.27961071	0	1.23636365	0.30610314	0.43185939	0.88005519	0
eptC	0.66738686	-0.5834048	0.13068629	0.17753495	0	0.94052221	-0.0884661	0.86024821	0.894035	0	1.4092609	0.49493873	0.24886743	0.74826869	0
ppc	0.31802002	-1.6528105	8.52E-08	3.96E-07	-1	0.30602436	-1.7082816	2.23E-06	1.21E-05	-1	0.96228016	-0.0554711	0.68995602	0.94554543	0
argE	0.5112863	-0.9677967	0.00268368	0.00546762	0	0.43532781	-1.1998259	0.00780847	0.01692281	-1	0.85143648	-0.2320292	0.78900684	0.96411212	0
argC	1.51169056	0.59616286	0.13910368	0.18749494	0	1.63430684	0.70867887	0.06535371	0.10889821	0	1.08111202	0.11251601	0.56513406	0.92545162	0
argB	1.55302092	0.63507726	0.09138135	0.12883225	0	1.90056675	0.92642969	0.06796627	0.11263645	0	1.22378696	0.29135243	0.61441555	0.92897598	0
phoB	0.89034304	-0.1675668	0.93744561	0.95340358	0	0.95749104	-0.0626691	1	1	0	1.07541813	0.1048977	0.83104566	0.97888613	0
argH	0.9667637	-0.0487648	0.68742799	0.73967587	0	0.95511704	-0.0662506	0.83467006	0.87485934	0	0.98795295	-0.0174858	0.66938447	0.93979017	0
oxyS	2.24436819	1.16630937	0.23321646	0.29567081	0	1.42406745	0.51001748	0.70064656	0.76865868	0	0.63450706	-0.6562919	0.60381462	0.92806272	0
oxyR	0.15004994	-2.7364854	8.43E-16	9.24E-15	-1	0.14806139	-2.7557327	1.00E-11	1.39E-10	-1	0.98674739	-0.0192473	0.86750393	0.98621446	0
sthA	0.40739476	-1.2955007	1.74E-05	5.66E-05	-1	0.18183551	-2.4592941	1.21E-09	1.26E-08	-1	0.44633739	-1.1637934	0.00342987	0.04178382	-1
fabR	0.11989253	-3.0601864	4.12E-19	5.76E-18	-1	0.17313515	-2.5300294	4.94E-11	6.32E-10	-1	1.44408626	0.53015692	0.36061179	0.85270669	0
yijD	0.08143826	-3.6181494	1.95E-24	4.05E-23	-1	0.15172381	-2.7204806	6.45E-12	9.25E-11	-1	1.86305313	0.89766881	0.04845753	0.29299759	0
trmA	0.39059119	-1.3562687	4.40E-05	0.00013336	-1	0.34661718	-1.5285849	0.00020296	0.0006691	-1	0.88741679	-0.1723162	0.65627135	0.93664426	0
btuB	1.47424514	0.55997644	0.11010104	0.15216405	0	0.80372307	-0.3152296	0.21260818	0.29424227	0	0.545176	-0.875206	0.00778836	0.07770743	0
murI	0.38860179	-1.3636356	0.00047416	0.00113455	-1	0.57186188	-0.8062613	0.05012251	0.08618342	0	1.47158841	0.55737422	0.26845846	0.76966758	0
rrsB	0	#NAME?	1	1	0	0	#NAME?	1	1	0	NA	NA	NA	NA	0
phoR	2.29261173	1.19699205	0.00290628	0.00587776	1	2.24144627	1.16442992	0.0063904	0.01416208	1	0.97768246	-0.0325621	0.83482251	0.98014902	0
gltT	0	#NAME?	0.33837339	0.40802491	0	6.70355008	2.74492532	0.5344293	0.62083151	0	Inf	Inf	0.14498213	0.57607933	0
murB	0.48518815	-1.0433838	0.00100246	0.00225107	-1	0.50721974	-0.9793172	0.0150303	0.02997243	0	1.04540834	0.06406657	0.84841326	0.98167799	0
birA	0.89819963	-0.154892	0.93764563	0.95340358	0	1.10434802	0.14319488	0.61441104	0.69270545	0	1.22951288	0.29808685	0.49148567	0.90539057	0
coaA	0.5163043	-0.9537065	0.01059178	0.01883409	0	0.89654651	-0.1575497	0.37090798	0.46222289	0	1.73646921	0.79615683	0.21375477	0.69446947	0
thrU	0.46115161	-1.116687	0.07924501	0.11338685	0	1.36747726	0.45151684	0.94835625	0.96406401	0	2.96535289	1.5682038	0.12187801	0.52159375	0
tyrU	0.62249414	-0.6838678	0.28583081	0.3528881	0	1.74665015	0.80459067	0.58806258	0.67003696	0	2.8058901	1.4884585	0.13538717	0.55180907	0
glyT	0.36971047	-1.4355322	0.02006265	0.03336474	-1	1.19052978	0.25160371	0.91483541	0.93645071	0	3.22016789	1.68713591	0.0546643	0.31430189	0
thrT	0.29649911	-1.7539003	1.08E-05	3.67E-05	-1	0.87511074	-0.1924625	0.40015859	0.49096896	0	2.95147847	1.56143782	0.00273306	0.03555391	1
brnQ	1.35888588	0.4424243	0.17787552	0.23300006	0	1.00452828	0.00651818	0.81275134	0.85676244	0	0.73922932	-0.4359061	0.17366044	0.6308423	0
tufB	0.0362754	-4.7848646	1.60E-38	9.16E-37	-1	0.11279766	-3.148191	4.93E-16	1.30E-14	-1	3.10948058	1.63667361	0.0001827	0.00426305	1
secE	0.36120231	-1.469121	8.04E-06	2.81E-05	-1	0.64036624	-0.6430308	0.07313569	0.12000988	0	1.77287414	0.82609012	0.04357577	0.27028009	0
nusG	0.32176255	-1.6359317	2.12E-06	8.18E-06	-1	0.39214332	-1.3505471	0.0001843	0.00061315	-1	1.21873512	0.28538461	0.80667067	0.96990832	0
rplK	0.02492519	-5.3262516	4.52E-16	5.03E-15	-1	0.1534842	-2.7038379	7.56E-05	0.00028021	-1	6.15779407	2.62241362	7.80E-05	0.00221717	1
rplA	0.02956325	-5.0800511	3.20E-13	2.78E-12	-1	0.12873033	-2.9575761	7.97E-05	0.00029307	-1	4.35440303	2.12247494	0.00276906	0.03591635	1
rplJ	0.02694877	-5.213637	1.16E-46	9.89E-45	-1	0.09342961	-3.4199763	2.00E-18	8.26E-17	-1	3.46693493	1.79366076	3.25E-06	0.00016115	1
rplL	0.02149426	-5.5399049	6.56E-51	7.43E-49	-1	0.08362653	-3.5798955	2.86E-20	1.51E-18	-1	3.8906452	1.96000942	1.09E-06	6.56E-05	1
rpoB	0.14501067	-2.7857691	8.52E-19	1.18E-17	-1	0.20072226	-2.3167275	6.05E-10	6.64E-09	-1	1.38418961	0.46904158	0.18763616	0.6609229	0
rpoC	0.06598087	-3.9218083	2.78E-31	1.01E-29	-1	0.105174	-3.24915	1.26E-16	3.70E-15	-1	1.59400744	0.67265837	0.06576931	0.35237234	0
yjaZ	1.42753847	0.51352962	0.54721243	0.61424074	0	1.39271395	0.47789897	0.51034841	0.59757012	0	0.9756052	-0.0356307	0.93513123	1	0
proY	1.65475478	0.72661744	0.0489087	0.07449301	0	1.87845106	0.90954353	0.02033627	0.03914023	0	1.13518395	0.18292609	0.52700081	0.91534998	0
sroH	0.85200275	-0.23107	0.95930048	0.97095101	0	1.26333245	0.33723434	0.9542639	0.968955	0	1.48277978	0.56830435	0.84311504	0.98067952	0
thiH	1.74840888	0.80604261	0.10024508	0.1402542	0	2.39017395	1.25711562	0.01749249	0.03423354	1	1.36705663	0.451073	0.30687837	0.81011621	0
thiG	2.32691118	1.21841614	0.00139005	0.00302158	1	4.44805155	2.15317351	3.07E-05	0.00012663	1	1.91156912	0.93475737	0.08720714	0.42308413	0
thiS	1.6864564	0.75399502	0.44395839	0.51455372	0	2.85055312	1.51124189	0.10641865	0.16448424	0	1.69026197	0.75724686	0.34403114	0.84269271	0
thiF	2.301082	1.20231239	0.09893631	0.13855475	0	2.84670696	1.50929399	0.04762837	0.0824079	0	1.2371167	0.3069816	0.53860629	0.91897962	0
thiE	2.08838867	1.06239024	0.07388891	0.10662114	0	3.28106815	1.71416556	0.00427328	0.00989707	1	1.57110034	0.65177532	0.18601216	0.65730257	0
thiC	2.65975195	1.41129171	0.00530076	0.010053	1	2.8211528	1.49628481	0.00174462	0.00450057	1	1.06068267	0.0849931	0.51252604	0.90925684	0
rsd	0.20445056	-2.2901761	2.08E-10	1.38E-09	-1	0.25075865	-1.9956286	2.01E-07	1.39E-06	-1	1.22650016	0.29454743	1	1	0
nudC	1.51992407	0.60399926	0.06111092	0.09042778	0	1.52721936	0.6109073	0.0767721	0.12512117	0	1.00479977	0.00690804	0.8153545	0.9719041	0
hemE	0.32082482	-1.6401424	2.67E-06	1.01E-05	-1	0.37462086	-1.4164968	0.00039993	0.00121295	-1	1.16768044	0.22364551	0.66386228	0.93720148	0
malZ	2.14021842	1.09775804	0.00059118	0.00138969	1	1.28378525	0.36040389	0.23707611	0.32165776	0	0.59983843	-0.7373541	0.08237053	0.40815058	0
nfi	0.47671589	-1.0687984	0.0034707	0.0068714	-1	0.58955077	-0.762312	0.07473882	0.12236785	0	1.23669209	0.30648634	0.52209496	0.91269012	0

yjaG	0.19794215	-2.3368492	1.53E-11	1.14E-10	-1	0.20096681	-2.3149708	1.21E-07	8.77E-07	-1	1.01528051	0.02187839	0.89735393	0.99640384	0
hupA	0.01033561	-6.5962333	1.37E-66	4.67E-64	-1	0.01140727	-6.4539028	5.57E-45	6.15E-42	-1	1.10368656	0.14233052	1	1	0
yjaH	0.14162513	-2.8198509	5.42E-11	3.79E-10	-1	0.22021074	-2.1830433	9.31E-06	4.41E-05	-1	1.55488468	0.63680758	0.21588933	0.69799995	0
zraP	3.13290158	1.64749945	0.00403618	0.00787101	1	2.45672038	1.29673366	0.02417641	0.04561724	1	0.78416775	-0.3507658	0.76896351	0.96147692	0
zraS	0.44817462	-1.1578671	0.00273258	0.00555956	-1	0.51924765	-0.9455053	0.05552402	0.09392812	0	1.15858333	0.21236181	0.48064229	0.89935549	0
zraR	0.37141582	-1.4288928	8.47E-05	0.00024078	-1	0.43088169	-1.2146363	0.00697816	0.01534154	-1	1.16010594	0.21425656	0.52011373	0.91269012	0
purD	1.00680469	0.00978384	0.87008201	0.89730381	0	1.90267102	0.92802614	0.03649662	0.06577281	0	1.88981145	0.9182423	0.03897032	0.25088919	0
purH	2.11421789	1.08012407	0.00651827	0.01212772	1	2.69107535	1.42818279	0.00121744	0.00328842	1	1.27284674	0.34805872	0.35270015	0.84775072	0
acpH	2.31791803	1.21282955	0.00348966	0.00690584	1	1.9349894	0.95232567	0.06096565	0.10251415	0	0.8347963	-0.2605039	0.58478361	0.92806272	0
rrfE	0	#NAME?	1	1	0	10.1229439	3.33955701	0.59390971	0.6748488	0	Inf	Inf	0.57322796	0.92806272	0
yjaA	1.53237754	0.61577178	0.54183307	0.60918864	0	1.09570473	0.13185908	0.58755372	0.66969303	0	0.71503576	-0.4839127	0.8885556	0.99411016	0
yjaB	2.54139952	1.34562319	0.00252939	0.00517717	1	1.78247709	0.83388353	0.04872775	0.08411247	0	0.70137618	-0.5117397	0.51524993	0.90937129	0
metA	7.91105098	2.98386937	4.53E-11	3.18E-10	1	5.00255461	2.32266501	1.20E-05	5.52E-05	1	0.63235019	-0.6612044	0.15552606	0.59527229	0
aceB	2.80670723	1.48887859	0.00026827	0.00067878	1	0.4757323	-1.0717781	0.01052748	0.02198532	-1	0.16949837	-2.5606567	3.07E-08	3.01E-06	-1
aceA	2.52194461	1.33453659	0.00067679	0.00157172	1	0.63352773	-0.6585203	0.14103301	0.20843642	0	0.25120605	-1.9930569	1.24E-05	0.00049092	-1
aceK	2.59036169	1.37315355	0.00136498	0.00297001	1	0.85372976	-0.2281486	0.61333657	0.69231492	0	0.32957936	-1.6013022	0.00080218	0.01432234	-1
arpA	4.23303602	2.08169276	2.37E-06	9.09E-06	1	3.57185852	1.83667494	6.93E-05	0.00025983	1	0.84380537	-0.2450178	0.86178292	0.98424873	0
fixB	2.8073716	1.48922004	0.00077406	0.00178354	1	5.11049087	2.35346187	4.59E-05	0.00018066	1	1.82038276	0.86424183	0.14850449	0.5824118	0
queA	1.29210499	0.3697233	0.27306961	0.33947325	0	2.94291394	1.55724535	0.0007616	0.00215739	1	2.27761208	1.18752205	0.00907074	0.08696078	0
iclR	0.45910844	-1.1230931	0.00074571	0.00172361	-1	0.60787346	-0.7181571	0.05477995	0.09292614	0	1.32403024	0.40493607	0.38013649	0.85988523	0
metH	0.46077237	-1.1178739	0.00157971	0.00339541	-1	0.59830776	-0.7410403	0.10877249	0.16736268	0	1.2984888	0.37683357	0.22457473	0.70791607	0
yjbB	1.64517183	0.71823827	0.04387772	0.06781242	0	1.38016766	0.46484353	0.14079679	0.20822658	0	0.83892007	-0.2533947	0.75517133	0.9573569	0
pepE	0.13346122	-2.9055075	4.58E-15	4.68E-14	-1	0.22293694	-2.1652924	3.59E-07	2.32E-06	-1	1.67042491	0.74021513	0.07053987	0.36864032	0
rluF	0.47142746	-1.0848923	0.00471365	0.00902089	-1	0.55601249	-0.8468108	0.08657575	0.1384648	0	1.17942321	0.2380815	0.50942596	0.90925684	0
yjbD	0.15443985	-2.694883	3.21E-12	2.55E-11	-1	0.15084655	-2.7288464	1.63E-08	1.40E-07	-1	0.97673333	-0.0339634	1	1	0
lysC	0.43479379	-1.2015968	0.00200537	0.00421395	-1	0.78693419	-0.3456851	0.53565089	0.6217084	0	1.80990208	0.85591164	0.03649992	0.23988772	0
pgi	0.22255023	-2.1677971	2.62E-12	2.09E-11	-1	0.14846307	-2.751824	5.62E-13	9.48E-12	-1	0.66709914	-0.5840269	0.06634283	0.35344999	0
yjbE	0.93171755	-0.1020354	0.98549101	0.99223018	0	1.0284052	0.04040881	0.7540575	0.81094672	0	1.10377357	0.14244424	0.72875553	0.95488877	0
yjbF	4.91154672	2.29617742	0.01098423	0.01946706	1	6.82768265	2.771396	0.0011462	0.00312081	1	1.39012882	0.47521858	0.33861297	0.83714543	0
tgt	0.18831508	-2.4087796	6.52E-12	5.03E-11	-1	0.51941022	-0.9450537	0.00996789	0.02101532	0	2.75819777	1.46372591	0.0003994	0.00794858	1
yjbG	4.73077851	2.24207762	4.50E-05	0.00013655	1	7.46943722	2.90099955	4.50E-06	2.27E-05	1	1.57890233	0.65892193	0.23421695	0.72636902	0
yjbH	3.05943121	1.61326346	2.06E-05	6.62E-05	1	5.43095141	2.44120496	1.75E-07	1.22E-06	1	1.77515069	0.82794149	0.07454462	0.38181389	0
yjbT	1.83415403	0.8751148	0.12649188	0.17223016	0	4.03052671	2.01096838	0.01866988	0.03634459	1	2.19748541	1.13585358	0.21957465	0.70219304	0
psiE	2.48433611	1.31286037	0.04622859	0.07097383	0	3.24388736	1.69772373	0.0248124	0.04659838	1	1.30573611	0.38486335	0.5831344	0.92806272	0
xylE	2.58419746	1.36971631	0.00042471	0.00102566	1	2.86385952	1.51796073	0.00022261	0.00072761	1	1.10822008	0.14824441	0.49593682	0.90539057	0
malG	0.29770278	-1.7480554	6.32E-08	3.00E-07	-1	0.11066822	-3.1756871	3.83E-13	6.64E-12	-1	0.37174064	-1.4276317	0.00090927	0.01542264	-1
malF	0.18642274	-2.4233502	8.92E-14	8.14E-13	-1	0.0848164	-3.559513	1.06E-16	3.18E-15	-1	0.45496808	-1.1361628	0.00660279	0.06883756	0
malE	0.0232871	-5.4243255	2.41E-50	2.54E-48	-1	0.01546619	-6.0147379	7.97E-40	5.87E-37	-1	0.66415302	-0.5904124	0.09884797	0.45789869	0
malK	0.1917431	-2.3827535	4.57E-14	4.27E-13	-1	0.05831384	-4.1000179	2.26E-22	1.72E-20	-1	0.30412483	-1.7172645	6.18E-06	0.00027797	-1
lamB	0.30940528	-1.6924303	2.52E-08	1.27E-07	-1	0.04232692	-4.5622806	2.59E-26	3.70E-24	-1	0.1368009	-2.8698503	4.10E-13	1.39E-10	-1
yajC	0.1540499	-2.6985303	2.04E-14	1.96E-13	-1	0.1938826	-2.3667447	2.51E-09	2.47E-08	-1	1.25857011	0.33178559	0.70048649	0.94869712	0
malM	0.19903731	-2.3288892	1.05E-12	8.80E-12	-1	0.09357854	-3.4176785	3.03E-15	7.39E-14	-1	0.47015576	-1.0887893	0.01188541	0.10697905	0
yjbI	12.1762797	3.6060015	2.08E-07	9.15E-07	1	7.58119906	2.92242605	0.00110316	0.00302224	1	0.62262031	-0.6835754	0.21293405	0.69446947	0
ubiC	0.63311714	-0.6594556	0.13470109	0.18222809	0	0.75059732	-0.413889	0.38010195	0.47155265	0	1.18555836	0.24556668	0.66163673	0.93664426	0
ubiA	0.81272816	-0.2991552	0.36621489	0.43670928	0	0.72264791	-0.4686352	0.31138462	0.40163708	0	0.88916312	-0.16948	0.75605196	0.9573569	0
plsB	0.18035639	-2.4710776	1.54E-13	1.37E-12	-1	0.31505285	-1.6663342	1.06E-05	4.93E-05	-1	1.74683499	0.80474334	0.04121456	0.26089831	0
dgkA	0.48526035	-1.0431691	0.00716696	0.01322325	-1	0.70225657	-0.5099299	0.12029431	0.18192353	0	1.44717484	0.53323923	0.5006571	0.90598897	0
lexA	0.15886011	-2.6541712	3.70E-15	3.81E-14	-1	0.25167506	-1.9903658	5.88E-08	4.53E-07	-1	1.58425587	0.66380536	0.21372493	0.69446947	0
dinF	1.15644195	0.20969284	0.46616739	0.53593477	0	1.76505597	0.81971393	0.04356321	0.07654307	0	1.52628152	0.61002109	0.13474818	0.54971274	0
yjbJ	8.67689774	3.11717933	1.71E-06	6.72E-06	1	1.00881099	0.01265589	0.46425263	0.55379079	0	0.11626402	-3.1045234	7.20E-06	0.00031432	-1
zur	0.44439318	-1.1700914	0.00107681	0.00239611	-1	0.26785067	-1.9004992	2.95E-05	0.00012193	-1	0.60273354	-0.7304078	0.10053467	0.46231274	0
secD	0.29932547	-1.7402131	4.59E-08	2.22E-07	-1	0.58076123	-0.783983	0.02422621	0.0456722	0	1.94023327	0.95623011	0.01367403	0.11800874	0
yjbL	5.39621139	2.43194687	0.02831796	0.04555004	1	3.49407382	1.80491009	0.08560202	0.13735487	0	0.64750499	-0.6270368	0.81946019	0.97433794	0
yjbM	5.20873259	2.38093237	0.0001116	0.00030791	1	6.89296845	2.78512541	0.00024287	0.00078682	1	1.3233485	0.40419304	0.64678956	0.93208609	0
dusA	0.85958562	-0.2182867	0.8289631	0.86210636	0	1.29812546	0.37642983	0.27227749	0.36034979	0	1.51017587	0.59471657	0.15545669	0.59527229	0
pspG	1.44353536	0.52960645	0.3198619	0.38910217	0	1.23089418	0.29970674	0.63867204	0.7163177	0	0.85269416	-0.2298997	0.7740407	0.96287211	0
qorA	0.71894166	-0.4760534	0.13108406	0.17787965	0	0.2372495	-2.075523	1.62E-06	9.15E-06	-1	0.32999827	-1.5994697	0.00016551	0.00403258	-1
dnaB	0.47307614	-1.0798557	0.00028815	0.00072522	-1	0.80922241	-0.3053918	0.37420726	0.46541568	0	1.71055424	0.77446385	0.03380907	0.22797859	0
alr	0.76359812	-0.3891145	0.21473068	0.27523663	0	1.22245364	0.28977976	0.40538365	0.4959705	0	1.60091233	0.67889431	0.05716591	0.32074131	0
tyrB	0.34256023	-1.5455704	2.36E-06	9.05E-06	-1	0.28393489	-1.816368	2.89E-05	0.00011974	-1	0.82886121	-0.2707975	0.73699601	0.95683847	0
yjbS	2.04310057	1.03076022	0.62958567	0.68875793	0	2.17786324	1.12291336	0.27549308	0.36365698	0	1.06595988	0.09215314	0.59754424	0.92806272	0
aphA	0.0439841	-4.5068741	3.26E-38	1.82E-36	-1	0.04620353	-4.4358532	2.55E-26	3.70E-24	-1	1.05045979	0.07102095	1	1	0
secF	0.44115837	-1.1806314	0.00022839	0.0005896	-1	0.53435883	-0.9041192	0.00803905	0.017335	0	1.21126304	0.2765122	0.71975045	0.95399541	0
yjbQ	0.34256098	-1.5455673	5.22E-06	1.89E-05	-1	0.14479107	-2.7879554	6.22E-11	7.70E-10	-1	0.42267241	-1.2423882	0.00157024	0.02429746	-1
yjbR	0.35230762	-1.5050924	1.44E-05	4.74E-05	-1	0.11948684	-3.0650763	3.76E-11	4.87E-10	-1	0.33915486	-1.5599839	0.00051033	0.0097851	-1
uvrA	0.73325093	-0.4476211	0.10899498	0.15077696	0	0.68728608	-0.5410174	0.16825307	0.24313614	0	0.93731361	-0.0933963	0.89824638	0.99645823	0
ssb	0.21274592	-2.2327966	2.34E-11	1.71E-10	-1	0.21672407	-2.2060687	1.42E-07	1.02E-06	-1	1.01869905	0.02672791	0.86002051	0.98424873	0
yjcB	0.42743357	-1.2262279	0.01716769	0.02906465	-1	0.52019651	-0.9428714	0.06860496	0.11356786	0	1.21702306	0.28335651	0.97044943	1	0
yjcC	2.88663229	1.52938734	0.00012395	0.0003376	1	2.42592964	1.27853771	0.00612865	0.01363671	1	0.84040134	-0.2508496	0.59171189	0.92806272	0
soxS	0.50867373	-0.9751875	0.1113335	0.15372306	0	1.17175504	0.228671	0.85821715	0.89318454	0	2.30354935	1.2038585	0.22352441	0.70561393	0
soxR	0.7694522	-0.3780964	0.77430178	0.81776507	0	1.00864888	0.01242405	0.86311829	0.89629718	0	1.31086619	0.39052042	0.56496777	0.92545162	0
ryjA	8.37537264	3.06615338	0.07802971	0.11179262	0	1.70008545	0.76560726	0.56768226	0.65158127	0	0.20298625	-2.3005461	0.32532087	0.82473426	0
ghxP	5.24418884	2.39071964	4.82E-05	0.00014546	1	5.4907017	2.45699053	0.00011899	0.00041933	1	1.04700686	0.0662709	0.71317099	0.95223057	0
yajD	0.1843464	-2.4395088	8.36E-10	5.17E-09	-1	0.17055466	-2.5516939	8.30E-08	6.23E-07	-1	0.92518574	-0.1121851	0.5876601	0.92806272	0
yjcE	0.76152396	-0.3930387	0.33023938	0.39974441	0	0.63102405	-0.6642331	0.27552015	0.36365698	0	0.82863321	-0.2711945	0.80161245	0.96905452	0
yjcF	1.6934601	0.75997399	0.62959729	0.68875793	0	1.58177807	0.6615472	0.24908582	0.33466411	0	0.93405098	-0.0984268	0.59350664	0.92806272	0
actP	7.96967322	2.99452057	1.07E-11	8.06E-11	1	6.3612669	2.66931412	4.77E-09	4.46E-08	1	0.79818416	-0.3252065	0.83400133	0.98014902	0
yjcH	8.69842552	3.12075429	6.91E-07	2.85E-06	1	3.405077	1.76768742	0.00172541	0.00446144	1	0.39145901	-1.3530669	0.14896665	0.58294573	0
acs	15.4720726	3.95159456	6.88E-13	5.85E-12	1	4.08160118	2.02913522	0.00011182	0.00039624	1	0.26380442	-1.9224593	0.00471767	0.05264765	0
nrfA	0.01914024	-5.7072469	4.38E-51	5.09E-49	-1	0.02615939	-5.2565272	2.01E-30	4.66E-28	-1	1.36672192	0.45071973	0.07652779	0.38702701	0
nrfB	0.0396272	-4.6573651	3.60E-32	1.37E-30	-1	0.05556452	-4.1696923	5.76E-19	2.57E-17	-1	1.40218119	0.48767279	0.18594935	0.65730257	0
nrfC	0.06982585	-3.840095	1.43E-20	2.16E-19	-1	0.15208954	-2.7170071	6.77E-09	6.22E-08	-1	2.17812664	1.12308783	0.00759623	0.07668786	0
nrfD	0.47755785	-1.0662526	0.00171564	0.00365539	-1	0.61801115	-0.6942952	0.19783301	0.2768284	0	1.29410739	0.37195734	0.16132167	0.60662351	0
nrfE	1.95665904	0.96839238	0.08435573	0.11996112	0	2.99746817	1.58374443	0.01426545	0.02855028	1	1.53193178	0.61535205	0.2903617	0.79336747	0
tsx	0.00753583	-7.052018	2.47E-71	1.56E-68	-1	0.03948364	-4.6626014	3.62E-29	6.39E-27	-1	5.23945459	2.38941664	5.44E-10	8.88E-08	1
nrfF	2.31971381	1.21394683	0.02216389	0.03657	1	2.79380737	1.48223255	0.00957151	0.02027617	1	1.20437588	0.26828572	0.50828907	0.90925684	0
nrfG	1.17330562	0.23057885	0.90026089	0.92248593	0	1.26525994	0.33943381	0.54553066	0.63035622	0	1.07837201	0.10885495	0.59388312	0.92806272	0
gltP	0.78476872	-0.3496605	0.4198071	0.49294292	0	1.13018774	0.17656244	0.75906181	0.81513831	0	1.4401539	0.52622299	0.26699027	0.76922645	0
yjcO	0.22550455	-2.1487716	6.28E-09	3.44E-08	-1	0.28409181	-1.8155709	3.59E-06	1.85E-05	-1	1.25980522	0.3332007	0.92834782	1	0
fdhF	0.10340813	-3.2735784	6.34E-21	9.93E-20	-1	0.12459349	-3.0046994	1.32E-13	2.47E-12	-1	1.20487123	0.26887897	0.59551874	0.92806272	0
mdtP	5.07948439	2.34468206	0.00020578	0.00053719	1	6.58860866	2.71997384	4.42E-05	0.00017472	1	1.29710186	0.37529178	0.37703084	0.85988523	0
mdtO	10.5535082	3.39965075	1.01E-13	9.18E-13	1	14.754637	3.88309652	9.11E-12	1.27E-10	1	1.39807889	0.48344577	0.59228642	0.92806272	0
mdtN	4.93940805	2.30433816	2.80E-06	1.06E-05	1	5.83152457	2.5438731	5.89E-07	3.61E-06	1	1.18061203	0.23953495	0.36766537	0.8542699	0
ytcA	7.30264283	2.86841867	0.00219503	0.00456257	1	9.01316027	3.17203304	0.00517678	0.01174343	1	1.23423266	0.30361437	0.82073636	0.97480076	0
yjcS	7.00085553	2.80753124	7.74E-06	2.71E-05	1	9.60403824	3.26364115	5.17E-06	2.58E-05	1	1.3718378	0.45610991	0.43330541	0.88049119	0
yajI	0.74430854	-0.4260273	0.68986993	0.74194192	0	0.96094017	-0.0574815	0.77149851	0.82448656	0	1.29105086	0.36854583	0.84604126	0.98157127	0
alsK	2.19565329	1.13465026	0.01958237	0.03262744	1	3.02899238	1.59883795	0.00369256	0.00870261	1	1.37954038	0.46418769	0.32012638	0.82031222	0
alsE	1.88747643	0.91645863	0.07724468	0.11085208	0	2.25684429	1.17430688	0.02481087	0.04659838	1	1.19569402	0.25784825	0.47657555	0.89855925	0
alsC	2.7060763	1.43620252	0.00301407	0.00607074	1	2.81854368	1.49494992	0.00122514	0.0033072	1	1.04156105	0.05874741	0.53957332	0.91908781	0
alsA	1.66995417	0.73980851	0.13748405	0.18553836	0	1.7760533	0.82867488	0.09303432	0.1472488	0	1.06353416	0.08886637	0.69247467	0.94603882	0
alsB	2.31672259	1.21208531	0.00550296	0.0104186	1	2.00688138	1.00495535	0.19475981	0.27383973	0	0.86625882	-0.20713	0.33678866	0.83714543	0
alsR	0.34080935	-1.5529632	4.07E-05	0.00012422	-1	0.33542062	-1.5759567	0.00067837	0.00194027	-1	0.98418845	-0.0229935	1	1	0

rpiB	1.54780326	0.6302221	0.30873632	0.37691773	0	1.05953231	0.08342759	0.95772709	0.97114015	0	0.6845394	-0.5467945	0.32205994	0.8228762	0
yjdP	0.88539662	-0.1756042	0.80761916	0.84512055	0	1.03167356	0.04498655	0.81856419	0.86129979	0	1.16521064	0.22059078	1	1	0
phnP	1.6498234	0.72231161	0.17010363	0.22421598	0	1.50782381	0.59246786	0.22253232	0.30625049	0	0.91393043	-0.1298437	1	1	0
phnO	1.48723313	0.57263081	0.33689172	0.40679352	0	1.08343798	0.11561657	0.91930525	0.94059039	0	0.72849237	-0.4570142	0.36143104	0.85310733	0
nrdR	0.42659877	-1.2290483	0.00022935	0.00059173	-1	0.3187764	-1.6493833	0.0003122	0.00097846	-1	0.74725109	-0.420335	0.415663	0.87131399	0
phnN	1.46033512	0.54629948	0.44271249	0.51324438	0	1.43511837	0.52116974	0.40717992	0.49787717	0	0.98273222	-0.0251297	0.85448559	0.98236299	0
phnM	3.18019965	1.66911734	0.00957535	0.0171649	1	2.71894039	1.44304452	0.02341084	0.04428616	1	0.85495902	-0.2260728	0.98459945	1	0
phnL	0.57391302	-0.801096	0.18242196	0.23831937	0	0.66662217	-0.5850588	0.44386477	0.53372473	0	1.16153868	0.21603719	0.69174043	0.94599088	0
phnK	3.3750003	1.75488763	0.00134173	0.00292518	1	4.6294615	2.21084439	0.00105021	0.00289513	1	1.37169217	0.45595676	0.50433916	0.90701715	0
phnJ	4.44189914	2.15117663	0.00025044	0.00063978	1	6.66795431	2.73724422	7.52E-05	0.00027873	1	1.50114942	0.58606758	0.36170645	0.85310733	0
phnI	3.74264054	1.90405649	0.00056654	0.00133817	1	4.49961218	2.16980066	0.00173964	0.00449245	1	1.20225604	0.26574417	0.74104072	0.95683847	0
phnH	2.76743023	1.46854694	0.06720697	0.09832832	0	3.99790986	1.99924594	0.08965956	0.14241754	0	1.44462896	0.530699	0.7685285	0.96147692	0
phnG	2.97213942	1.57150179	0.10127885	0.14134239	0	3.5796836	1.83983208	0.12537705	0.18825729	0	1.20441308	0.26833028	0.83662914	0.98025477	0
phnF	1.6627707	0.73358923	0.38452836	0.45596289	0	3.73269523	1.90021772	0.02255304	0.04284689	1	2.24486469	1.16662849	0.12207968	0.52159375	0
phnE	3.30606504	1.72511511	0.01624801	0.02765389	1	3.77087351	1.91489876	0.00703061	0.01543382	1	1.14059266	0.18978365	0.54991523	0.91965346	0
ribD	0.55472053	-0.850167	0.01182088	0.02083512	0	0.51160416	-0.9669001	0.02722396	0.05063243	0	0.9222737	-0.1167331	0.89885457	0.99646774	0
phnD	4.79534402	2.26163432	1.52E-05	5.00E-05	1	8.80703404	3.13865624	4.30E-07	2.74E-06	1	1.83658023	0.87702192	0.13443459	0.54914241	0
phnC	4.27722533	2.09667521	0.00093669	0.00211412	1	8.4593921	3.08055399	1.06E-05	4.93E-05	1	1.97777565	0.98387878	0.09820147	0.45734597	0
yjdN	3.95862284	1.98499862	0.06024124	0.08932043	0	1.36310792	0.44689979	0.58115858	0.66376456	0	0.34433892	-1.5380988	0.23865821	0.73190732	0
yjdM	0.19158481	-2.3839449	9.76E-09	5.20E-08	-1	0.18575026	-2.4285639	1.26E-06	7.23E-06	-1	0.96954589	-0.0446189	0.79808129	0.96743774	0
crfC	1.06804231	0.0949688	0.9751042	0.98314023	0	1.00831518	0.01194667	0.59939083	0.68020239	0	0.94407794	-0.0830221	0.69911929	0.94869712	0
yjcZ	2.2070395	1.14211245	0.00460271	0.0088162	1	2.71701857	1.44202443	0.001457	0.00385538	1	1.2310693	0.29991198	0.39430489	0.86854717	0
proP	71.8543	6.16700259	3.96E-64	9.20E-62	1	6.43303257	2.68549899	6.09E-09	5.65E-08	1	0.08952885	-3.4815036	3.70E-21	4.08E-18	-1
pmrR	2.98794658	1.57915436	0.14503776	0.19472091	0	0.87922532	-0.1856952	0.69868868	0.76727268	0	0.29425738	-1.7648495	0.11396066	0.50027742	0
basS	1.01508677	0.02160306	0.66194333	0.71714586	0	0.98749134	-0.01816	0.74398309	0.80225995	0	0.9728147	-0.0397631	1	1	0
basR	0.4567792	-1.1304312	0.00574378	0.01081427	-1	0.55430241	-0.8512548	0.2253325	0.30923737	0	1.21350188	0.27917634	0.24066296	0.73370247	0
fixC	3.68563066	1.8819115	0.00020816	0.00054308	1	6.59116278	2.720533	3.06E-06	1.60E-05	1	1.78834056	0.8386215	0.10775861	0.48409953	0
ribE	0.55593584	-0.8470097	0.01969265	0.03278644	0	0.75005356	-0.4149345	0.2320163	0.31679816	0	1.34917288	0.43207523	0.42869896	0.87784805	0
eptA	2.83888909	1.50532648	0.00077888	0.00179182	1	2.79743451	1.48410436	0.00238807	0.00594529	1	0.98539761	-0.0212221	0.85415978	0.98236299	0
adiC	2.00341134	1.00245866	0.00859438	0.01558989	1	2.2963142	1.19932006	0.00242649	0.00602397	1	1.14620206	0.19686139	0.42555733	0.87726173	0
adiY	0.19454228	-2.3618443	5.81E-10	3.66E-09	-1	0.12605263	-2.9879018	4.26E-09	4.01E-08	-1	0.64794466	-0.6260575	0.34463961	0.84269271	0
adiA	1.68449426	0.75231551	0.02129383	0.03523974	0	2.04847741	1.03455198	0.00522197	0.01182772	1	1.2160786	0.28223647	0.36350307	0.85329623	0
melR	0.22902241	-2.1264393	9.59E-09	5.13E-08	-1	0.24447431	-2.0322452	8.35E-06	4.01E-05	-1	1.06746892	0.09419406	0.69021579	0.94554543	0
melA	0.27444832	-1.8653936	1.69E-09	9.91E-09	-1	0.14089473	-2.8273104	2.88E-13	5.12E-12	-1	0.51337437	-0.9619168	0.00507741	0.05569993	0
melB	0.40091074	-1.318647	6.35E-06	2.26E-05	-1	0.18086025	-2.4670527	2.41E-10	2.79E-09	-1	0.45112348	-1.1484057	0.001811	0.02662176	-1
yjdF	1.89981948	0.92586234	0.01127364	0.01991189	0	2.62471943	1.39216322	0.0031089	0.00744619	1	1.38156255	0.46630088	0.33692069	0.83714543	0
fumB	0.45057032	-1.1501758	0.0077224	0.01415933	-1	0.5541696	-0.8516005	0.14262336	0.19984869	0	1.22992922	0.2985753	0.33407181	0.83424743	0
dcuB	0.78409677	-0.3508964	0.3648088	0.43573836	0	0.99232437	-0.0111163	0.79407506	0.84270357	0	1.26556365	0.33978006	0.28530656	0.78928465	0
nusB	0.36459837	-1.45562	2.43E-05	7.70E-05	-1	0.51144353	-0.9673531	0.01947789	0.03773468	0	1.40275869	0.48826685	0.22070993	0.70378221	0
dcuR	0.32155942	-1.6368428	9.06E-06	3.13E-05	-1	0.37228601	-1.4255167	0.00181712	0.00466039	-1	1.15775185	0.21132607	0.50143031	0.90598897	0
dcuS	0.60450619	-0.726171	0.03958435	0.06184296	0	0.62187505	-0.6853034	0.10912354	0.16778599	0	1.0287323	0.04086762	0.91250399	1	0
yjdI	6.05705588	2.59861672	1.44E-05	4.74E-05	1	1.44983172	0.53588546	0.19004047	0.26863436	0	0.23936245	-2.0627313	0.00892179	0.08609434	0
yjdJ	6.21879185	2.63663433	0.00459015	0.00879596	1	0.83550099	-0.2592866	0.74604001	0.80408556	0	0.13435101	-2.8959209	0.00700383	0.07166332	0
ghoS	0.07909534	-3.6602634	1.39E-13	1.24E-12	-1	0.08937872	-3.4839249	3.82E-09	3.64E-08	-1	1.13001236	0.17633856	0.8424725	0.98067952	0
ghoT	0.10415511	-3.2631945	1.55E-08	8.08E-08	-1	0.10467553	-3.2560038	4.56E-05	0.00017981	-1	1.00499663	0.00719067	0.50162356	0.90598897	0
lysU	0.14613342	-2.7746419	1.28E-17	1.65E-16	-1	0.07617239	-3.714588	8.24E-20	4.09E-18	-1	0.52125236	-0.9399461	0.0069101	0.07086869	0
dtpC	2.62557004	1.39263068	0.00038951	0.00095053	1	2.78372332	1.47701582	0.00042197	0.00126762	1	1.06023579	0.08438514	0.62321867	0.92989404	0
cadA	0.58219918	-0.7804153	0.07570332	0.10895457	0	0.59685409	-0.7445498	0.32953289	0.41905204	0	1.02517164	0.03586547	0.53896323	0.91897962	0
cadB	0.76766624	-0.3814489	0.77151316	0.81564711	0	0.80000881	-0.3219122	0.81198079	0.8565188	0	1.04213102	0.05953667	1	1	0
thiL	0.72477962	-0.4643857	0.30228215	0.37016365	0	1.02226264	0.03176591	0.77536602	0.82801054	0	1.41044617	0.49615161	0.20927876	0.69013336	0
cadC	1.8948993	0.92212118	0.05321245	0.08032788	0	1.67326576	0.7426666	0.24981658	0.33554392	0	0.88303677	-0.1794546	0.60701222	0.92806272	0
yjdQ	7.12229087	2.83234136	0.00038504	0.00094014	1	9.22171729	3.20503544	0.00035924	0.0011024	1	1.29476842	0.37269408	0.58868249	0.92806272	0
pheU	1.97023648	0.9783688	0.24921841	0.31352826	0	1.57968143	0.65963364	0.61345806	0.69231492	0	0.8017725	-0.3187352	0.67531395	0.94091029	0
yjdC	1.60420043	0.6818544	0.05115378	0.07767145	0	0.69675746	-0.5212716	0.1696368	0.24473557	0	0.43433317	-1.203126	0.00226006	0.03134232	-1
dsbD	1.80290459	0.85032305	0.01451872	0.02502115	0	1.43051343	0.51653305	0.32806221	0.41742208	0	0.79344933	-0.33379	0.28630559	0.78944262	0
cutA	4.91259947	2.29648662	5.40E-06	1.94E-05	1	2.15028198	1.10452586	0.16890933	0.24376562	0	0.43770757	-1.1919608	0.01235775	0.10874916	0
dcuA	0.07357313	-3.7646772	2.32E-29	7.21E-28	-1	0.24741662	-2.0149857	2.73E-09	2.68E-08	-1	3.36286649	1.7496915	2.20E-05	0.00077572	1
aspA	0.23775606	-2.072446	7.58E-12	5.82E-11	-1	0.31871501	-1.6496611	1.10E-07	8.06E-07	-1	1.3405127	0.42278489	0.80198443	0.96923852	0
fxsA	1.08903112	0.12304519	0.47453123	0.54441674	0	1.68780766	0.7551505	0.20145259	0.2809148	0	1.54982499	0.63210532	0.37733709	0.85988523	0
yjeH	3.51595967	1.81391852	5.50E-07	2.29E-06	1	4.44739075	2.15295917	1.15E-06	6.69E-06	1	1.26491518	0.33904064	0.45609981	0.88755478	0
pgpA	0.4252235	-1.2337068	0.0002961	0.00074143	-1	0.41042142	-1.2848221	0.00121104	0.00327313	-1	0.96518988	-0.0511153	0.75950939	0.95807677	0
groS	0.13923066	-2.8444511	9.59E-18	1.25E-16	-1	0.24943205	-2.0032812	5.62E-08	4.35E-07	-1	1.79150226	0.84116987	0.05543442	0.31647516	0
groL	0.18835211	-2.4084959	3.51E-14	3.31E-13	-1	0.27899956	-1.8416653	2.97E-07	1.97E-06	-1	1.48126593	0.56683067	0.18226978	0.65078561	0
yjeI	0.09867454	-3.3411783	7.18E-22	1.23E-20	-1	0.12081875	-3.0490837	4.36E-13	7.47E-12	-1	1.22441666	0.29209458	0.34900283	0.84705695	0
yjeJ	0.55598708	-0.8468767	0.0750378	0.10806716	0	0.70269218	-0.5090353	0.56223104	0.64616358	0	1.26386423	0.3378415	0.34510273	0.84269271	0
epmB	0.64607801	-0.6302197	0.03431433	0.05426652	0	1.03632794	0.05148061	0.88327801	0.91217703	0	1.60402912	0.68170033	0.05985154	0.33200664	0
efp	0.14816487	-2.7547246	4.05E-17	5.00E-16	-1	0.27329716	-1.8714576	2.24E-07	1.53E-06	-1	1.84454755	0.88326698	0.04232832	0.26515328	0
ecnA	0.45068824	-1.1497983	0.0641549	0.09445739	0	0.76118318	-0.3936844	0.61448413	0.69270545	0	1.68893509	0.75611388	0.29899454	0.80400362	0
ecnB	1.82791802	0.87020137	0.5144236	0.58291664	0	1.15813857	0.21180788	0.75109558	0.80835152	0	0.63358343	-0.6583935	0.36425284	0.85329623	0
sugE	2.52704225	1.33744978	0.0011945	0.00263674	1	1.40788658	0.49353111	0.4350398	0.52540062	0	0.55712823	-0.8439187	0.05717298	0.32074131	0
blc	2.2515988	1.17094979	0.05462274	0.08217596	0	0.61562553	-0.699875	0.17152352	0.24673257	0	0.27341706	-1.8708248	0.00193969	0.02795437	-1
yajO	0.6453689	-0.631804	0.04207573	0.06523289	0	0.25618076	-1.964766	1.19E-06	6.89E-06	-1	0.39695244	-1.3329619	0.00057946	0.01092063	-1
ampC	1.38763397	0.47262707	0.21726418	0.27816113	0	1.58053455	0.66041257	0.07033722	0.11610765	0	1.13901402	0.18778551	0.43716084	0.88087171	0
frdD	0.0664072	-3.9125165	4.68E-27	1.21E-25	-1	0.10982374	-3.1867382	8.24E-15	1.85E-13	-1	1.65379263	0.72577835	0.1016756	0.46707227	0
frdC	0.03469464	-4.8491435	6.02E-40	3.80E-38	-1	0.06515489	-3.9399826	1.45E-20	7.82E-19	-1	1.87795281	0.90916081	0.01423604	0.12119871	0
frdB	0.02813121	-5.1516847	8.22E-45	6.49E-43	-1	0.05456064	-4.1959956	3.45E-23	3.11E-21	-1	1.93950574	0.95568904	0.0076639	0.07698818	0
frdA	0.03375332	-4.888827	6.78E-42	4.91E-40	-1	0.06916351	-3.8538451	1.30E-20	7.09E-19	-1	2.04908795	1.03498191	0.00288811	0.03702485	1
epmA	1.45208895	0.53812983	0.24803593	0.3123075	0	1.75354562	0.81027496	0.09533268	0.15045539	0	1.20760207	0.27214514	0.50190344	0.90598897	0
yjeM	3.95585847	1.98399081	2.33E-07	1.02E-06	1	3.67585795	1.87808101	3.52E-05	0.00014304	1	0.92921877	-0.1059098	0.77062576	0.96191893	0
yjeN	1.78762582	0.83804479	0.20710046	0.26653925	0	1.86261622	0.89733044	0.12642372	0.18963557	0	1.04194972	0.05928566	0.64717633	0.93208609	0
yjeO	1.7364057	0.79610407	0.19197161	0.24924709	0	2.15487708	1.10760558	0.08293522	0.13360945	0	1.24099862	0.31150151	0.53100747	0.91754792	0
mscM	0.81762074	-0.2904963	0.59196882	0.65466356	0	1.14868871	0.19998789	0.34639937	0.43685468	0	1.40491632	0.4904842	0.12944413	0.53863917	0
dxs	0.74829051	-0.4183296	0.19038309	0.24762135	0	0.82140872	-0.2838278	0.51712064	0.60437876	0	1.09771368	0.1345018	0.657208	0.93664426	0
psd	0.45141547	-1.1474722	0.00053816	0.00127661	-1	0.74143458	-0.4316087	0.20304876	0.28269454	0	1.64246604	0.71586354	0.08652688	0.42070951	0
rsgA	0.25657226	-1.9625629	1.40E-08	7.33E-08	-1	0.60643687	-0.7215706	0.03214864	0.05880168	0	2.3636104	1.24099225	0.00477029	0.052923	0
orn	0.22566512	-2.1477446	2.20E-08	1.11E-07	-1	0.31374501	-1.6723356	0.00015228	0.00052053	-1	1.39031236	0.47540905	0.31364873	0.81404427	0
glyV	2.60641977	1.38206945	0.08378845	0.11930805	0	2.32613242	1.21793323	0.52313624	0.61060197	0	0.8924627	-0.1641362	0.47467252	0.89845244	0
glyX	1.70079405	0.76620846	0.4209118	0.49340961	0	2.1856181	1.12804134	0.5460793	0.6305525	0	1.28505747	0.36183288	1	1	0
glyY	3.28177119	1.71447466	0.04088997	0.06366267	0	3.33537493	1.73784894	0.24827005	0.33368455	0	1.01633378	0.02337429	0.65984402	0.93664426	0
yjeV	1.38111702	0.46583557	1	1	0	1.4044381	0.48999304	0.94730421	0.96329223	0	1.01688567	0.02415748	1	1	0
queG	0.96284978	-0.0546174	0.89780493	0.92073471	0	1.19068695	0.25179416	0.47213803	0.56130696	0	1.23662795	0.30641152	0.39529847	0.86854717	0
nnr	1.17873933	0.23724471	0.27373631	0.34001499	0	1.04727124	0.06663515	0.64972941	0.72549312	0	0.88846721	-0.1706096	0.72513196	0.95459348	0
tsaE	1.27129402	0.34629775	0.46261697	0.53266684	0	1.25344403	0.32589758	0.55308342	0.63714171	0	0.9859592	-0.0204002	0.96651824	1	0
ispA	0.48227761	-1.0520643	0.00289514	0.00586059	-1	0.86226346	-0.2137994	0.64980805	0.72549312	0	1.78789859	0.83826491	0.02985917	0.20967987	0
amiB	0.79848918	-0.3246552	0.26979512	0.33622936	0	0.8328095	-0.2639416	0.60992587	0.68914917	0	1.04298157	0.06071366	0.7295455	0.95488877	0
mutL	0.73171409	-0.4506481	0.15080185	0.20166267	0	0.80355332	-0.3155343	0.48498923	0.57319267	0	1.09817937	0.13511372	0.63326267	0.93163024	0
miaA	0.218424	-2.1947967	8.20E-12	6.27E-11	-1	0.17030491	-2.5538081	1.38E-11	1.87E-10	-1	0.7796987	-0.3590114	0.16621561	0.61554659	0
hfq	0.09408905	-3.4098294	8.76E-24	1.71E-22	-1	0.10137094	-3.302284	2.77E-17	9.26E-16	-1	1.07739363	0.10754544	0.76831081	0.96147692	0
hflX	0.39998227	-1.3219921	1.57E-05	5.13E-05	-1	0.29207331	-1.7755976	3.03E-06	1.59E-05	-1	0.73021564	-0.4536055	0.19683752	0.6744782	0
hflK	0.39803149	-1.3290455	1.10E-05	3.72E-05	-1	0.30958881	-1.6915748	8.29E-06	3.99E-05	-1	0.77779978	-0.3625293	0.32496532	0.82473426	0
hflC	0.3785366	-1.4014953	7.62E-06	2.67E-05	-1	0.24983099	-2.0009757	1.65E-07	1.16E-06	-1	0.65999164	-0.5994804	0.07697763	0.38841113	0
yjeT	2.79958063	1.48521073	0.02759287	0.04456223	1	3.04302385	1.60550564	0.11174664	0.1709271	0	1.08695703	0.12029491	0.92062006	1	0

purA	0.08836007	-3.5004616	5.93E-25	1.30E-23	-1	0.20512599	-2.2854178	1.49E-09	1.52E-08	-1	2.32147836	1.21504383	0.00116386	0.01893952	1
nsrR	0.69477661	-0.5253789	0.38848763	0.46020644	0	1.08082961	0.11213911	0.67270022	0.74521491	0	1.55565055	0.63751802	0.73317788	0.95599369	0
xseB	0.22433311	-2.1562855	2.85E-09	1.64E-08	-1	0.46407723	-1.1075632	0.00618647	0.01375151	-1	2.06869703	1.04872237	0.018489	0.14612278	0
rnr	0.45524385	-1.1352886	0.00011903	0.00032637	-1	0.22624933	-2.1440146	1.04E-08	9.18E-08	-1	0.49698492	-1.008726	0.00365777	0.04377371	-1
rlmB	0.42618183	-1.230459	0.00020903	0.00054472	-1	0.36056605	-1.4716645	0.00064757	0.00186303	-1	0.84603805	-0.2412055	0.63435752	0.93163024	0
yjfI	2.1255106	1.08780945	0.19278477	0.25008232	0	1.29461641	0.3725247	0.36444066	0.45622189	0	0.6090849	-0.7152848	0.84199483	0.98067952	0
yjfJ	3.70012573	1.8875743	0.0031779	0.006363	1	4.15363263	2.05437362	0.00300145	0.00722407	1	1.12256527	0.16679933	0.67262372	0.93997784	0
yjfK	5.0968952	2.34961869	0.00033429	0.0008272	1	6.67821953	2.73946352	0.00010624	0.00038014	1	1.31025247	0.38984483	0.44061394	0.88087171	0
yjfL	10.8962522	3.44576009	0.00125911	0.00276554	1	5.16977285	2.37010089	0.02311452	0.04378186	1	0.47445422	-1.0756592	0.53815985	0.91897962	0
yjfM	6.15787443	2.62243245	0.00019123	0.00050182	1	8.49112619	3.08595591	3.54E-05	0.00014345	1	1.37890538	0.46352347	0.35418997	0.84775072	0
yjfC	18.0118535	4.17087474	6.39E-25	1.40E-23	1	10.9048562	3.44689884	7.46E-13	1.24E-11	1	0.60542666	-0.7239759	0.04123495	0.26089831	0
aidB	6.4905028	2.69833024	5.13E-11	3.60E-10	1	2.23421884	1.15977051	0.00637155	0.01413447	1	0.34422893	-1.5385597	0.00228801	0.03143336	-1
yjfN	0.08115456	-3.6231841	1.38E-24	2.92E-23	-1	0.1178994	-3.0843717	5.26E-14	1.05E-12	-1	1.45277612	0.53881239	0.23849312	0.73190732	0
thiI	1.00888648	0.01276386	0.77642591	0.81927216	0	1.23831933	0.30838339	0.46487043	0.55415765	0	1.22741195	0.29561953	0.37532123	0.85938038	0
bsmA	0.07869456	-3.6675923	1.97E-26	4.86E-25	-1	0.13888574	-2.8480296	1.35E-14	2.89E-13	-1	1.76487099	0.81956273	0.18349645	0.65259624	0
yjfP	5.56344864	2.47597945	1.14E-06	4.55E-06	1	2.99581153	1.58294686	0.04615419	0.08044071	0	0.53848103	-0.8930326	0.03644145	0.23986086	0
ulaR	0.50646899	-0.9814542	0.01604048	0.02734496	0	0.60631909	-0.7218508	0.11851858	0.17952536	0	1.19714948	0.25960331	0.60237413	0.92806272	0
ulaG	2.11493208	1.08061133	0.09942887	0.13920042	0	2.30024607	1.2017882	0.08692474	0.13897266	0	1.08762172	0.12117687	0.72301731	0.95399541	0
ulaA	3.3987031	1.76498434	0.00016213	0.00043219	1	3.88542744	1.95807332	0.00015664	0.00053447	1	1.14320884	0.19308898	0.57581854	0.92806272	0
ulaB	1.86454682	0.89882502	0.3730135	0.44373838	0	2.54715394	1.34888615	0.10876338	0.16736268	0	1.36609814	0.45006113	0.4515145	0.88755478	0
ulaC	1.77675513	0.82924486	0.204022	0.26303713	0	2.06893775	1.04889024	0.04047769	0.07198025	0	1.16444732	0.21964537	0.40186816	0.86854717	0
ulaD	1.51159529	0.59607193	0.11750247	0.1612328	0	2.35349127	1.2348025	0.00549311	0.01238148	1	1.55695859	0.63873057	0.12656142	0.5319293	0
ulaE	2.34797086	1.2314145	0.0033428	0.00664199	1	2.7033716	1.43475984	0.00250181	0.00615563	1	1.15136506	0.20334533	0.5617265	0.92432422	0
ulaF	4.07560542	2.02701438	3.18E-06	1.20E-05	1	5.70510343	2.51225304	1.60E-06	9.04E-06	1	1.39981741	0.48523866	0.33008847	0.8313479	0
yajL	0.82108532	-0.284396	0.36601994	0.43670928	0	0.37730072	-1.4062132	0.00296363	0.00714862	-1	0.45951463	-1.1218173	0.01754275	0.14117431	0
yjfY	2.78419233	1.47725887	0.01196084	0.02104822	1	3.01178893	1.59062067	0.01947772	0.03773468	1	1.08174601	0.1133618	0.79071232	0.96513738	0
rpsF	0.02237258	-5.4821243	6.20E-24	1.25E-22	-1	0.25750042	-1.9573533	0.0002573	0.00082571	-1	11.5096411	3.52477094	1.64E-10	3.15E-08	1
priB	0.02054171	-5.6052999	1.48E-23	2.84E-22	-1	0.21912932	-2.1901455	7.75E-05	0.00028671	-1	10.6675309	3.41515438	1.86E-09	2.64E-07	1
rpsR	0.03869238	-4.6918066	3.15E-21	5.06E-20	-1	0.17432499	-2.5201487	1.61E-06	9.07E-06	-1	4.50540843	2.1716579	2.78E-05	0.000937	1
rplI	0.02573869	-5.2799177	2.52E-21	4.12E-20	-1	0.18199122	-2.4580593	1.24E-05	5.70E-05	-1	7.07072642	2.82185844	1.12E-06	6.68E-05	1
yjfZ	2.9787922	1.57472749	0.08523156	0.12101183	0	3.1310992	1.64666922	0.0783102	0.12723209	0	1.05113046	0.07194173	0.81266007	0.97070176	0
ytfA	2.96073791	1.56595678	0.04394234	0.06786479	0	2.21769619	1.14906174	0.17600698	0.25170707	0	0.74903496	-0.416895	0.71515813	0.9525362	0
ytfB	0.38671807	-1.3706459	0.00020045	0.00052391	-1	0.26510575	-1.9153601	2.19E-05	9.33E-05	-1	0.68552717	-0.5447142	0.21800284	0.70102268	0
fklB	0.19575318	-2.3528923	9.78E-12	7.43E-11	-1	0.23719303	-2.0758665	5.03E-07	3.15E-06	-1	1.21169436	0.27702584	0.46670558	0.89320173	0
cycA	2.10853214	1.07623901	0.01841738	0.03088443	1	1.11544014	0.15761309	0.6017999	0.68223545	0	0.52901263	-0.9186259	0.11146733	0.49465734	0
fixX	5.81317498	2.53932634	0.00025375	0.00064601	1	8.59888608	3.10414978	8.65E-05	0.00031597	1	1.47920648	0.56482345	0.40337049	0.86854717	0
panE	0.4177791	-1.2591878	0.00027969	0.00070473	-1	0.3325347	-1.5884232	0.00024603	0.00079474	-1	0.79595821	-0.3292354	0.4518785	0.88755478	0
ytfE	30.0632959	4.90993128	2.81E-12	2.24E-11	1	31.4181778	4.9735276	3.48E-10	3.96E-09	1	1.04506764	0.06359632	0.98816041	1	0
ytfF	3.10798742	1.63598067	3.88E-05	0.00011878	1	3.57024593	1.83602346	0.00016152	0.00054862	1	1.14873243	0.20004279	0.67806926	0.94128448	0
qorB	0.63182961	-0.6623926	0.17857738	0.23373578	0	0.74923292	-0.4165138	0.62282267	0.70138974	0	1.18581483	0.24587874	0.46477815	0.89206388	0
ytfH	2.27827956	1.18794479	0.00900101	0.01626071	1	3.14738396	1.65415319	0.00391934	0.00917836	1	1.38147399	0.4662084	0.42605158	0.87757472	0
cpdB	0.24975638	-2.0014066	8.11E-09	4.39E-08	-1	0.28330187	-1.819588	3.82E-05	0.00015377	-1	1.13431285	0.1818186	0.40828169	0.86959974	0
cysQ	0.68866209	-0.5381318	0.07194275	0.10408488	0	0.30143266	-1.7300924	1.58E-05	7.02E-05	-1	0.43770764	-1.1919605	0.00203208	0.0288282	-1
ytfI	2.96612415	1.56857899	0.06919709	0.10057372	0	1.46215499	0.54809625	0.25353621	0.33979482	0	0.49295138	-1.0204827	0.53210293	0.91785957	0
ytfJ	0.6289567	-0.6689674	0.05549937	0.08323963	0	0.39239167	-1.3496337	0.00215743	0.00542612	-1	0.62387708	-0.6806663	0.10436243	0.47496212	0
ytfK	0.32512702	-1.6209246	2.85E-06	1.08E-05	-1	0.2035759	-2.2963613	2.17E-09	2.16E-08	-1	0.62614267	-0.6754367	0.00860814	0.08380111	0
ytfL	0.7890122	-0.3418805	0.51370593	0.58229135	0	1.46256544	0.54850118	0.33877086	0.42924839	0	1.85366645	0.89038167	0.09709554	0.45510989	0
yajQ	0.08620053	-3.5361595	1.03E-24	2.18E-23	-1	0.12294272	-3.0239417	3.23E-15	7.72E-14	-1	1.42624097	0.51221776	0.51578868	0.90937129	0
msrA	0.73716322	-0.439944	0.18994942	0.24727575	0	0.51816248	-0.9485235	0.00595116	0.01328862	0	0.70291418	-0.5085795	0.0837732	0.41278193	0
tamA	1.21560063	0.28166933	0.33225556	0.40174454	0	0.94758277	-0.0776761	0.7710515	0.82440759	0	0.77951816	-0.3593455	0.58875544	0.92806272	0
tamB	0.95218722	-0.0706828	0.81322218	0.84957482	0	0.85806004	-0.2208495	0.73592963	0.79746764	0	0.90114634	-0.1501667	0.85909707	0.98424873	0
ytfP	0.76430377	-0.3877819	0.36558408	0.43642835	0	0.53861	-0.8926871	0.08122921	0.13129087	0	0.70470671	-0.5049051	0.28955901	0.79166474	0
chpS	1.06923964	0.09658523	0.85757788	0.88710106	0	1.25566381	0.32845025	0.45735712	0.54667599	0	1.17435209	0.23186501	0.56336433	0.92432422	0
chpB	1.55521006	0.63710945	0.15360617	0.20497838	0	1.73966866	0.79881255	0.07878577	0.12780996	0	1.11860687	0.1617031	0.59029802	0.92806272	0
ppa	0.06469226	-3.9502632	1.54E-31	5.62E-30	-1	0.16691082	-2.5828506	6.76E-13	1.13E-11	-1	2.58007429	1.36741261	0.00142064	0.02221644	1
ytfQ	1.20034397	0.26344788	0.6349738	0.69319805	0	0.28987904	-1.7864771	3.41E-05	0.00013903	-1	0.24149664	-2.049925	1.99E-06	0.00010307	-1
ytfR	2.03317718	1.02373594	0.00285151	0.00578288	1	1.31278046	0.39262567	0.17866535	0.25509602	0	0.64567932	-0.6311103	0.20976333	0.69013336	0
ytfT	2.88238982	1.52726546	2.62E-05	8.27E-05	1	2.6288312	1.39442151	0.00035453	0.00109101	1	0.91203181	-0.132844	0.98660446	1	0
yajR	1.27235775	0.34750437	0.50691425	0.57647793	0	1.31329104	0.39318667	0.239076	0.32367551	0	1.03217122	0.0456823	0.60908215	0.92806272	0
yjfF	2.95378214	1.56256342	2.51E-05	7.96E-05	1	1.53951096	0.62247214	0.04243119	0.07496718	0	0.5211999	-0.9400913	0.10787575	0.48409953	0
fbp	0.2146264	-2.2201005	3.88E-11	2.79E-10	-1	0.26901518	-1.8942405	2.89E-06	1.54E-05	-1	1.25341139	0.32586001	0.3512116	0.84775072	0
mpl	0.38596258	-1.3734671	6.18E-05	0.00018202	-1	0.46391952	-1.1080535	0.00631419	0.01401426	-1	1.20198055	0.26541355	0.48826892	0.90208042	0
yjgA	0.4835218	-1.0483472	0.00102815	0.00230057	-1	0.22380013	-2.1597172	4.36E-07	2.77E-06	-1	0.46285427	-1.1113701	0.00684606	0.07037554	0
pmbA	1.50607288	0.59079158	0.45841048	0.52839224	0	2.24690079	1.16793642	0.39053444	0.4813868	0	1.4918938	0.57714484	0.77151662	0.96223954	0
cybC	1.02655689	0.03781358	0.97512731	0.98314023	0	1.17127067	0.2280745	0.93948701	0.95769161	0	1.14097005	0.19026093	0.95551028	1	0
nrdG	0.91396194	-0.129794	0.97217873	0.98133794	0	1.86555245	0.89960292	0.07843176	0.12732915	0	2.04117083	1.02939693	0.04925741	0.29394474	0
nrdD	0.39404317	-1.3435744	5.82E-06	2.08E-05	-1	0.38466998	-1.3783069	0.0002009	0.000663	-1	0.97621278	-0.0347325	1	1	0
treC	0.01084962	-6.5262122	3.20E-62	6.15E-60	-1	0.14071925	-2.8291084	1.95E-13	3.59E-12	-1	12.9699755	3.69710385	3.38E-22	4.97E-19	1
treB	0.00755246	-7.0488375	1.26E-70	6.97E-68	-1	0.14553895	-2.7805228	2.93E-13	5.20E-12	-1	19.2704009	4.26831468	2.87E-29	1.27E-25	1
cyoE	0.14922767	-2.7444131	1.97E-14	1.89E-13	-1	0.15603442	-2.6800637	2.35E-10	2.73E-09	-1	1.04561324	0.06434931	1	1	0
treR	1.06819771	0.0951787	0.66352591	0.71850795	0	1.3376915	0.41974544	0.34082198	0.43117444	0	1.2522883	0.32456674	0.5047695	0.90710411	0
mgtL	0.61851983	-0.6931083	0.24000365	0.30332535	0	1.15686612	0.21022192	0.89080695	0.91754305	0	1.87037839	0.90333017	0.40481831	0.86873419	0
mgtA	2.36325792	1.24077709	0.00035269	0.0008669	1	2.65531281	1.40888183	0.00025533	0.00081998	1	1.12358147	0.16810474	0.534461	0.9187668	0
ridA	0.08645657	-3.5318805	2.11E-26	5.18E-25	-1	0.15041327	-2.7329963	9.00E-14	1.74E-12	-1	1.73975512	0.79888426	0.11087024	0.49387651	0
pyrI	0.55213417	-0.8569092	0.1026956	0.14309352	0	0.3530793	-1.5019359	0.00790273	0.01711032	-1	0.63948097	-0.6450267	0.21373709	0.69446947	0
pyrB	0.55488161	-0.8497481	0.08634541	0.12243585	0	0.66246223	-0.5940899	0.52495786	0.61175864	0	1.19388031	0.25565821	0.37865371	0.85988523	0
pyrL	2.88263831	1.52738983	0.05990129	0.08890592	0	2.67736821	1.42081556	0.10629063	0.16440262	0	0.92879089	-0.1065743	1	1	0
yjgH	2.6204204	1.38979829	0.05223838	0.07904656	0	1.87464254	0.90661553	0.08913582	0.14184054	0	0.71539763	-0.4831828	0.85716707	0.98347354	0
bdcA	1.55867019	0.64031569	0.16766427	0.22146324	0	1.99602568	0.99713029	0.03353705	0.06101285	0	1.28059527	0.35681459	0.34120077	0.8405152	0
bdcR	0.42614589	-1.2305807	0.0137863	0.02393636	-1	1.21831926	0.28489224	0.77785435	0.82987406	0	2.85892529	1.51547292	0.01330008	0.11545938	0
cyoD	0.11361061	-3.1378305	3.32E-15	3.45E-14	-1	0.08573377	-3.5439926	2.56E-12	3.92E-11	-1	0.75462817	-0.4061621	0.42365435	0.87508931	0
tabA	0.09455986	-3.4026282	2.09E-17	2.63E-16	-1	0.08660329	-3.5294343	2.24E-12	3.46E-11	-1	0.91585677	-0.1268061	0.96205008	1	0
yjgL	1.23321999	0.30243018	0.38402819	0.45573683	0	1.15503005	0.20793038	0.80647839	0.85218269	0	0.93659692	-0.0944998	0.69573161	0.94834535	0
argI	4.21377162	2.07511212	1.37E-05	4.56E-05	1	3.12689978	1.64473298	0.00039106	0.00118769	1	0.74206674	-0.4303791	0.7041396	0.94896888	0
rraB	0.13773908	-2.8599902	4.33E-18	5.81E-17	-1	0.15107864	-2.7266284	8.84E-13	1.44E-11	-1	1.09684661	0.13336179	1	1	0
yjgM	1.27538083	0.3509281	0.42838062	0.5003014	0	1.13467406	0.18227794	0.69207129	0.76151968	0	0.88967471	-0.1686502	0.77929591	0.9635071	0
yjgN	5.316226	2.41040244	5.37E-05	0.00016061	1	3.67332021	1.87708466	0.00113742	0.00310263	1	0.69096389	-0.5333178	0.68063116	0.94335731	0
valS	0.3280398	-1.6080572	2.89E-07	1.25E-06	-1	0.36852738	-1.4401563	0.00010872	0.00038776	-1	1.12342276	0.16790093	0.69455041	0.94773784	0
holC	0.36259664	-1.4635625	0.00036258	0.00088873	-1	0.38537985	-1.375647	0.00460588	0.0105677	-1	1.06283346	0.08791555	0.8942681	0.9954996	0
pepA	0.24612538	-2.0225346	9.47E-10	5.75E-09	-1	0.18321602	-2.4483824	1.80E-09	1.82E-08	-1	0.74440116	-0.4258478	0.28168318	0.78561671	0
lptF	0.74833707	-0.4182399	0.19017939	0.24742927	0	0.8150783	-0.2949894	0.41725536	0.50780816	0	1.08918606	0.12325042	0.80513447	0.96990832	0
cyoC	0.17880905	-2.4835084	2.05E-13	1.80E-12	-1	0.17869055	-2.4844648	4.41E-10	4.94E-09	-1	0.99933728	-0.0009564	0.82848363	0.97794776	0
lptG	0.71352694	-0.4869602	0.29031885	0.3577953	0	0.75289854	-0.4094726	0.35501406	0.44619088	0	1.05517887	0.07748758	1	1	0
yjgR	3.58982133	1.84391204	5.61E-08	2.69E-07	1	2.88677318	1.52945776	2.43E-05	0.00010273	1	0.80415511	-0.3144543	0.61816014	0.92897598	0
idnR	1.27918239	0.35522199	0.53966329	0.60711274	0	1.71655366	0.77951496	0.0724154	0.11904897	0	1.3419147	0.42429297	0.2241695	0.70714413	0
idnT	2.56779562	1.36053038	0.00089405	0.00202826	1	3.96466217	1.98719794	1.67E-05	7.36E-05	1	1.54399444	0.62666756	0.12232805	0.52159375	0
idnO	1.72849493	0.78951637	0.19559524	0.25313336	0	2.02228286	1.0159848	0.04411975	0.07730577	0	1.16996748	0.22646843	0.42063974	0.87375001	0
idnD	1.78773447	0.83813247	0.0773761	0.11100041	0	1.87664678	0.90815713	0.02575464	0.04814287	0	1.04973463	0.07002466	0.5236068	0.91269012	0
idnK	1.5115712	0.59604894	0.16115998	0.21376685	0	1.03514619	0.04983452	0.67898142	0.75104353	0	0.6848147	-0.5462144	0.43304014	0.88049119	0
ahr	3.58008739	1.83999481	0.00027344	0.00068977	1	2.13431844	1.09377544	0.04475551	0.07832657	0	0.59616378	-0.7462194	0.26936368	0.76991797	0
leuX	6.78553928	2.76246348	0.06376921	0.09395217	0	4.83859857	2.27458925	0.17444632	0.24979853	0	0.71307502	-0.4878742	0.78330706	0.9635071	0
intB	0.54194579	-0.8837795	0.02385144	0.03903009	0	0.52777172	-0.922014	0.04262238	0.07521897	0	0.97384596	-0.0382345	0.87871918	0.98956956	0

cyoB	0.34556715	-1.532962	3.24E-07	1.39E-06	-1	0.23556613	-2.085796	4.52E-08	3.55E-07	-1	0.68167975	-0.552834	0.13051002	0.5394088	0
insD1	0.15776987	-2.6641064	0.65100354	0.70685414	0	0.96712124	-0.0482313	0.7395222	0.79881412	0	6.12994901	2.61587507	0.47593026	0.89855925	0
yjgX	1.4032474	0.48876938	0.21259638	0.27273837	0	1.27081385	0.34575272	0.34139408	0.43152758	0	0.90562352	-0.1430167	0.88081824	0.99092052	0
yjgZ	4.72585497	2.24057536	0.01036287	0.01845678	1	6.54210632	2.70975521	0.00394274	0.00922339	1	1.38432228	0.46917985	0.4695169	0.89538835	0
insG	1.30012339	0.37864855	0.21786721	0.27885235	0	1.96732985	0.97623887	0.01593081	0.03152795	0	1.51318703	0.59759032	0.13931974	0.56235861	0
yjhB	0.80597274	-0.3111971	0.30635524	0.37432119	0	0.67718682	-0.5623742	0.30104617	0.39131593	0	0.84021058	-0.2511771	0.75223559	0.95683847	0
yjhC	0.35331091	-1.5009898	6.39E-05	0.00018756	-1	0.26888851	-1.89492	0.0001133	0.00040085	-1	0.76105352	-0.3939302	0.54631321	0.91914725	0
ythA	1.90527795	0.93000148	0.48529811	0.55460847	0	3.47650921	1.79763941	0.34818314	0.43860357	0	1.82467299	0.86763793	0.68539454	0.943596	0
yjhD	2.61966454	1.38938208	0.03902403	0.06105885	0	3.36333329	1.74989175	0.08449706	0.13582849	0	1.28387938	0.36050967	0.8609245	0.98424873	0
yjhE	1.27990041	0.35603156	0.99196636	0.99773972	0	2.80573231	1.48837737	0.23291496	0.31767013	0	2.19214893	1.13234582	0.25730807	0.75850842	0
cyoA	0.4667855	-1.0991683	0.00021642	0.00056034	-1	0.50400799	-0.9884815	0.0042564	0.00986344	0	1.07974217	0.11068685	0.89549094	0.99588104	0
insO	1.89274222	0.92047794	0.03525774	0.05563896	0	2.30852805	1.20697326	0.00831898	0.01782811	1	1.21967377	0.28649532	0.38708788	0.86562571	0
insI1	0	#NAME?	1	1	0	3.79782028	1.92517163	0.74669735	0.80440165	0	Inf	Inf	0.79760308	0.96743774	0
insM	3.1636801	1.66160373	0.01942012	0.03239376	1	2.81382673	1.49253349	0.10414552	0.16204897	0	0.88941569	-0.1690702	0.70522598	0.94896888	0
yjhV	2.35557059	1.23607657	0.08938884	0.12618424	0	1.84718757	0.88533037	0.17247515	0.2476983	0	0.7841784	-0.3507462	0.84925392	0.98212032	0
fecE	0.70990905	-0.4942939	0.15968252	0.2119344	0	0.77628058	-0.3653499	0.68031013	0.75194859	0	1.093493	0.12894398	0.47353633	0.89828993	0
fecD	1.1621416	0.21678587	0.5056775	0.57536773	0	1.61116712	0.68810615	0.10843021	0.16698087	0	1.38637763	0.47132028	0.2687728	0.76966758	0
fecC	0.89850096	-0.154408	0.666525	0.72104847	0	1.33527893	0.41714115	0.23388176	0.31840697	0	1.48611853	0.57154919	0.11670445	0.50756076	0
fecB	0.76479124	-0.3868621	0.15661794	0.20836791	0	0.9503072	-0.0735341	0.7953411	0.84343948	0	1.24257073	0.31332798	0.39658898	0.86854717	0
fecA	0.86836116	-0.2036329	0.51703751	0.58512803	0	0.96685465	-0.0486291	0.74395543	0.80225995	0	1.11342457	0.15500382	0.82479898	0.97661485	0
fecR	1.87808148	0.90925966	0.03098262	0.04944011	0	1.60986187	0.68693691	0.11764115	0.17858339	0	0.85718425	-0.2223227	0.79400175	0.96579786	0
ampG	1.41165709	0.49738969	0.11358381	0.15648774	0	1.67120224	0.74088633	0.10031578	0.15692343	0	1.18385849	0.24349665	0.67270082	0.93997784	0
fecI	1.44789492	0.53395691	0.20116089	0.2600315	0	1.33839382	0.42050269	0.49053047	0.57865834	0	0.9243722	-0.1134542	0.72528565	0.95459348	0
insA7	0.31230793	-1.6789589	0.04568582	0.07028711	0	1.39387701	0.47910327	0.90033857	0.92367121	0	4.46314963	2.15806217	0.05413843	0.3120921	0
insB1	1.99275353	0.99476328	0.08613529	0.12217713	0	2.5171549	1.331794	0.02636993	0.04914749	1	1.26315416	0.33703072	0.44799033	0.88755478	0
yjhU	0.31237516	-1.6786484	1.01E-06	4.09E-06	-1	0.33342765	-1.5845544	6.94E-05	0.0002601	-1	1.06739489	0.09409401	0.93447894	1	0
yjhF	4.13539813	2.04802623	8.57E-05	0.0002431	1	4.15621452	2.05527012	0.00017706	0.00059453	1	1.00503371	0.00724389	0.72038036	0.95399541	0
yjhG	1.35492639	0.43821448	0.11515347	0.15845261	0	1.7757389	0.82841947	0.01639138	0.03226437	0	1.31057961	0.39020499	0.23986927	0.73317437	0
yjhH	0.9559562	-0.0649836	0.89850296	0.92102287	0	1.22910134	0.29760387	0.50659649	0.59443703	0	1.28572976	0.36258745	0.51144206	0.90925684	0
yjhI	2.09597117	1.06761887	0.01749662	0.0295611	1	2.36297779	1.24060607	0.06489342	0.10825368	0	1.12739041	0.1729872	0.97254982	1	0
sgcR	1.44904015	0.53509757	0.22618085	0.28807405	0	1.38399626	0.46884005	0.19637009	0.27565421	0	0.95511243	-0.0662575	0.80608126	0.96990832	0
sgcE	1.50554528	0.5902861	0.17534771	0.23037206	0	1.22588508	0.29382374	0.39668039	0.4876024	0	0.81424657	-0.2964624	0.78169684	0.9635071	0
yajG	0.31292945	-1.6760907	9.95E-06	3.41E-05	-1	0.50597353	-0.9828662	0.00812401	0.0174866	0	1.61689332	0.69322449	0.25899887	0.75892335	0
sgcA	1.30385133	0.38277937	0.67590823	0.72941282	0	1.02487964	0.03545449	0.55748102	0.64187301	0	0.78604026	-0.3473249	0.95758712	1	0
ryjB	0.76145545	-0.3931685	0.50996129	0.57934645	0	0.6890393	-0.5373418	0.62123628	0.69995999	0	0.90489771	-0.1441734	0.96389115	1	0
sgcQ	1.59289849	0.67165434	0.10057156	0.14062189	0	2.04667112	1.03327929	0.01148846	0.02374533	1	1.28487228	0.36162496	0.26202293	0.76071172	0
sgcC	2.48003717	1.31036174	0.00183932	0.00389468	1	2.40679482	1.26711316	0.0049045	0.01116588	1	0.97046724	-0.0432486	0.84325096	0.98067952	0
sgcB	1.17699009	0.23510217	0.79213727	0.83207379	0	1.11559836	0.15781772	0.76240419	0.81813116	0	0.94784007	-0.0772844	0.97195727	1	0
sgcX	1.0271908	0.03870418	0.7712323	0.81554538	0	1.13681483	0.18499728	0.46383781	0.55379079	0	1.10672217	0.1462931	0.59130784	0.92806272	0
yjhY	0.64360456	-0.6357535	0.07651683	0.10994627	0	0.44490749	-1.1684227	0.01216918	0.02489611	-1	0.6912746	-0.5326692	0.24950282	0.7487007	0
yjhP	1.9058583	0.93044086	0.01394295	0.02415138	0	2.87207728	1.52209457	0.00127616	0.0034261	1	1.50697315	0.59165371	0.19907643	0.67679424	0
yjhQ	1.10380131	0.14248051	0.4984829	0.56791306	0	2.1261112	1.08821706	0.01599618	0.03159906	1	1.92617201	0.94573655	0.04678133	0.28534673	0
yjhX	0.33604646	-1.5732674	0.07126858	0.10327865	0	1.05456925	0.07665383	0.85958885	0.89399438	0	3.13816504	1.64992123	0.06398836	0.34581946	0
yaaU	7.39378292	2.88631269	0.00035386	0.0008693	1	7.30012728	2.86792162	0.00044426	0.00132918	1	0.98733319	-0.0183911	0.7383727	0.95683847	0
bolA	0.7240182	-0.4659021	0.1756086	0.2306462	0	0.20332493	-2.298141	1.86E-06	1.03E-05	-1	0.28082848	-1.8322388	6.81E-05	0.0020026	-1
yjhZ	3.71971607	1.8951925	0.05660314	0.08475121	0	3.05347586	1.61045244	0.07194093	0.11853355	0	0.8208895	-0.2847401	0.9659512	1	0
yjhR	6.13524394	2.61712071	1.26E-09	7.53E-09	1	5.20619658	2.38022979	8.69E-07	5.18E-06	1	0.84857206	-0.2368909	0.77035924	0.96185851	0
nanS	3.79486938	1.92405023	0.00067134	0.00155986	1	2.62038441	1.38977847	0.01148586	0.02374533	1	0.69050714	-0.5342718	0.65862486	0.93664426	0
nanM	1.13168268	0.17846949	0.88344371	0.9089613	0	0.63857235	-0.647078	0.46443735	0.55379079	0	0.56426803	-0.8255475	0.30056875	0.80487807	0
nanC	0.8274947	-0.273178	0.55240055	0.6188063	0	1.05470932	0.07684544	0.68656456	0.75774655	0	1.27458135	0.35002346	0.37914224	0.85988523	0
fimB	0.91522803	-0.1277969	0.57176045	0.63660387	0	0.62118501	-0.6869051	0.11409015	0.17372996	0	0.67872157	-0.5591082	0.20970789	0.69013336	0
fimE	3.65451434	1.86967969	0.00475029	0.00907921	1	3.13078072	1.64652246	0.01315944	0.02663666	1	0.85668858	-0.2231572	0.97455011	1	0
fimA	0.01271016	-6.297874	6.12E-59	1.04E-56	-1	0.0246025	-5.3450514	6.87E-33	2.17E-30	-1	1.93565601	0.95282259	0.01454521	0.12288196	0
fimI	0.62320826	-0.6822137	0.15016986	0.2009392	0	0.62032365	-0.688907	0.3480378	0.43854549	0	0.99537135	-0.0066932	0.73726217	0.95683847	0
fimC	0.99384765	-0.0089034	0.90754815	0.92835576	0	0.97631086	-0.0345875	0.56321112	0.64712167	0	0.98235466	-0.0256841	0.55296556	0.92069223	0
tig	0.04639886	-4.4297668	1.31E-36	6.10E-35	-1	0.13850196	-2.8520217	1.45E-13	2.70E-12	-1	2.98502928	1.57774508	1.81E-05	0.00067107	1
fimD	1.69829122	0.76408387	0.0236492	0.03874575	0	2.03067084	1.0219564	0.00444201	0.01022357	1	1.19571414	0.25787253	0.33642339	0.83714543	0
fimF	1.61514628	0.69166483	0.48947933	0.55891875	0	1.29776857	0.37603313	0.31004064	0.40048804	0	0.80349909	-0.3156317	0.79250231	0.96567635	0
fimG	2.18582756	1.12817959	0.06450842	0.09481986	0	1.77539523	0.82814023	0.1390058	0.20585343	0	0.81223023	-0.3000394	0.94647245	1	0
fimH	1.61621508	0.6926192	0.2087307	0.26840276	0	1.69941527	0.76503844	0.09845754	0.15466899	0	1.05147842	0.07241924	0.58647669	0.92806272	0
gntP	1.02879227	0.04095171	0.6005286	0.66313371	0	1.3548328	0.43811482	0.23388753	0.31840697	0	1.31691581	0.39716311	0.37648874	0.85988523	0
uxuA	0.13118443	-2.9303316	2.42E-16	2.75E-15	-1	0.17754635	-2.4937324	4.14E-09	3.91E-08	-1	1.3534102	0.43659917	0.25239615	0.75105736	0
uxuB	0.19421486	-2.3642745	2.31E-10	1.53E-09	-1	0.24967527	-2.0018751	8.45E-06	4.06E-05	-1	1.28556213	0.36239933	0.25927303	0.75892335	0
uxuR	0.54990136	-0.8627553	0.02343656	0.03842586	0	0.425194	-1.2338068	0.00824017	0.01767637	-1	0.77321869	-0.3710516	0.44125328	0.88087171	0
yjiC	4.25264091	2.08835904	0.00611523	0.01146476	1	3.94806778	1.98114676	0.00423766	0.00983521	1	0.92838024	-0.1072123	0.72405568	0.95458462	0
iraD	0.43714414	-1.193819	0.06558825	0.09618303	0	2.04838481	1.03448677	0.32705363	0.41697923	0	4.68583386	2.2283058	0.00832457	0.08158081	0
clpP	0.2511188	-1.993558	1.20E-10	8.05E-10	-1	0.18451591	-2.4381829	6.20E-11	7.70E-10	-1	0.73477536	-0.4446248	0.11450008	0.50143531	0
hypT	0.98767804	-0.0178873	0.68223509	0.73466298	0	1.52663668	0.61035676	0.22359203	0.30757058	0	1.54568252	0.62824402	0.13374635	0.54816114	0
iadA	0.4805123	-1.0573547	0.00128289	0.00281078	-1	0.55963981	-0.8374295	0.12253691	0.18481327	0	1.16467322	0.21992523	0.25816283	0.75892335	0
yjiG	0.98071114	-0.0280998	0.76577242	0.81074228	0	1.66221905	0.73311051	0.16222578	0.23534988	0	1.69491196	0.76121034	0.20174624	0.67864297	0
yjiH	6.1467362	2.61982057	0.00246267	0.00505466	1	6.77477376	2.76017277	0.00398325	0.00929845	1	1.10217415	0.1403522	0.75837572	0.95806023	0
kptA	1.10848558	0.14859001	0.68227022	0.73466298	0	0.77320721	-0.371073	0.68700689	0.75774655	0	0.69753475	-0.519663	0.43365688	0.88049119	0
yjiJ	2.80330334	1.48712787	0.00016224	0.00043222	1	3.09062982	1.62790087	0.00024603	0.00079474	1	1.10249568	0.140773	0.63789895	0.93163024	0
yjiK	1.5528761	0.63494272	0.16716656	0.2210041	0	1.46711772	0.55298463	0.21724798	0.29966213	0	0.94477449	-0.0819581	1	1	0
yjiL	1.3468723	0.42961308	0.41979981	0.49249292	0	0.54114646	-0.885909	0.10986973	0.16879854	0	0.40178008	-1.3155221	0.01232435	0.10870076	0
yjiM	0.22882242	-2.1276997	4.24E-09	2.39E-08	-1	0.297499	-1.7490433	0.00010257	0.00036821	-1	1.30013047	0.37865641	0.20610578	0.68468503	0
yjiN	2.84035657	1.50607205	0.00109003	0.00242065	1	1.25219713	0.32446171	0.44814068	0.53769581	0	0.44085913	-1.1816103	0.0346217	0.23131824	0
clpX	0.42140149	-1.2467327	5.06E-05	0.00015218	-1	0.21036505	-2.2490331	1.42E-09	1.46E-08	-1	0.49920337	-1.0023004	0.00235688	0.03193373	-1
mdtM	1.260483	0.33397666	0.24637326	0.31030245	0	1.41955687	0.50544065	0.17066307	0.24580016	0	1.12620073	0.17146399	0.61942457	0.92897598	0
yjiP	1.27418504	0.34957481	0.20921679	0.26887127	0	0.88892235	-0.1698707	1	1	0	0.69763992	-0.5194455	0.41586996	0.87131399	0
yjiR	3.51055414	1.81169878	5.53E-06	1.98E-05	1	3.19170517	1.67432739	5.49E-05	0.00021112	1	0.90917417	-0.1373714	0.9547277	1	0
yjiS	4.32314426	2.11208098	0.00406714	0.00791389	1	3.17613071	1.66727029	0.0193039	0.03743041	1	0.73468071	-0.4448107	0.85192806	0.98222294	0
yjiT	0.13773566	-2.8600259	4.69E-17	5.75E-16	-1	0.23159984	-2.1102939	6.18E-09	5.72E-08	-1	1.68148052	0.74973207	0.20108839	0.67848197	0
yjiV	1.39548859	0.48077032	0.19004895	0.24733241	0	1.54705861	0.62952785	0.07963429	0.12899704	0	1.1086143	0.14875753	0.54065797	0.91914725	0
mcrC	0.5006799	-0.9980395	0.04383409	0.0677687	0	0.35602166	-1.4899631	0.05268783	0.0898948	0	0.7110764	-0.4919235	0.74235329	0.95683847	0
mcrB	0.22244076	-2.1685069	3.67E-09	2.09E-08	-1	0.17890874	-2.4827043	1.42E-07	1.02E-06	-1	0.80429835	-0.3141973	0.55202255	0.92038542	0
symE	2.14902719	1.10368374	0.12517635	0.17070205	0	3.71729316	1.89425247	0.02222659	0.04235416	1	1.72975623	0.79056873	0.30563788	0.81005169	0
symR	0.70322202	-0.5079478	0.68030547	0.7330835	0	1.70398604	0.76891352	0.71339047	0.77968953	0	2.42311246	1.27686136	0.50685943	0.90925684	0
lon	0.28780801	-1.7968213	7.09E-09	3.86E-08	-1	0.27133082	-1.8818752	4.44E-07	2.82E-06	-1	0.94274937	-0.0850538	0.71635726	0.95281877	0
hsdS	0.52256815	-0.9363089	0.01040169	0.01851844	0	0.65627811	-0.6076208	0.2705725	0.3585187	0	1.25587086	0.32868813	0.31682587	0.81707725	0
hsdM	0.57162613	-0.8068562	0.0287112	0.04613218	0	0.80600812	-0.3111337	0.58065908	0.66363227	0	1.41002671	0.4957225	0.17035203	0.62318331	0
hsdR	0.14007813	-2.8356963	6.40E-18	8.47E-17	-1	0.17472087	-2.5168762	1.14E-10	1.38E-09	-1	1.24731007	0.31882015	0.38046271	0.85988523	0
mrr	0.69824666	-0.5181913	0.42277168	0.49507796	0	1.22201454	0.28926145	0.86392875	0.89638439	0	1.7501187	0.80745278	0.59129474	0.92806272	0
yjiA	0.28574748	-1.8071873	9.85E-08	4.55E-07	-1	0.31366201	-1.6727173	0.00011544	0.00040778	-1	1.09768948	0.13447	0.57334768	0.92806272	0
yjiX	0.31979433	-1.6447838	0.00350104	0.00692216	-1	0.31855413	-1.6503896	0.02166611	0.04146495	-1	0.99612189	-0.0056058	1	1	0
yjiY	0.83293858	-0.263718	0.63181235	0.6902585	0	1.05948511	0.08336331	0.93891196	0.95732625	0	1.27198467	0.34708129	0.55420889	0.92071004	0
tsr	1.01623763	0.02323779	0.76194464	0.80785153	0	1.16826321	0.22436535	0.3630934	0.45505098	0	1.14959648	0.20112755	0.47443824	0.89845244	0
lgoT	1.20050449	0.2636408	0.29578481	0.36361857	0	1.29670292	0.37484799	0.41885044	0.50932859	0	1.08013167	0.11120719	1	1	0
lgoR	0.25133671	-1.9923067	3.87E-09	2.19E-08	-1	0.23089593	-2.1146853	5.34E-07	3.31E-06	-1	0.91867176	-0.1223786	0.76989699	0.96177657	0
hupB	0.05120112	-4.2876807	4.51E-36	2.07E-34	-1	0.15349262	-2.7037588	2.39E-14	4.96E-13	-1	2.99783696	1.58392192	0.00030635	0.00652662	1
lgoD	2.97700751	1.57386286	0.00095952	0.00216125	1	2.50954805	1.32742757	0.00615681	0.01369248	1	0.84297673	-0.2464353	0.89208073	0.99488354	0
opgB	1.01564161	0.02239141	0.95613769	0.96887978	0	4.41660984	2.14293939	0.23700698	0.32165776	0	4.34859086	2.12054798	0.19540007	0.67373807	0

yjjA	0.51656938	-0.952966	0.00898934	0.01624628	0	0.97178306	-0.0412938	0.62852896	0.70712975	0	1.88122467	0.91167216	0.09364347	0.44558801	0
dnaC	0.69772241	-0.5192749	0.15653822	0.20832459	0	0.62013593	-0.6893436	0.34194083	0.43209508	0	0.88880037	-0.1700687	0.88583689	0.99349171	0
dnaT	0.67078054	-0.5760873	0.45581259	0.52592863	0	1.09080376	0.12539158	0.74965527	0.80726962	0	1.62617084	0.70147883	0.25753623	0.75867386	0
yjjB	0.40777389	-1.2941587	0.01709323	0.02896079	-1	0.38624421	-1.3724148	0.04094136	0.07265859	0	0.9472019	-0.0782561	1	1	0
yjjP	0.20182621	-2.3088146	7.78E-08	3.64E-07	-1	0.20952763	-2.2547876	3.47E-05	0.00014119	-1	1.0381587	0.054027	0.54638191	0.91914725	0
yjjQ	8.23391997	3.04157943	0.00478382	0.00913538	1	6.88999153	2.78450221	0.02289023	0.04341285	1	0.83678146	-0.2570772	0.86278742	0.98470303	0
bglJ	7.83926021	2.97071751	3.84E-08	1.90E-07	1	6.47195615	2.69420183	4.28E-05	0.00016963	1	0.82558251	-0.2765157	0.54549859	0.91914725	0
fhuF	3.55041831	1.82798901	0.02142721	0.03544719	1	2.58193616	1.36845333	0.19779589	0.2768284	0	0.72722027	-0.4595357	0.49666177	0.90539057	0
ppiD	0.29069241	-1.7824347	1.38E-08	7.21E-08	-1	0.47530794	-1.0730656	0.0016469	0.00428097	-1	1.63508889	0.70936907	0.09338238	0.44520681	0
yjjZ	2.14561297	1.10138986	0.05817795	0.08683101	0	2.47690205	1.30853682	0.09100605	0.14429699	0	1.154403	0.20714696	0.88032965	0.99065677	0
leuV	0.38875565	-1.3630644	0.47215474	0.54183099	0	2.38213484	1.25225508	0.70153386	0.76925016	0	6.12758893	2.61531952	0.29659619	0.80055472	0
leuP	0.27421026	-1.8666456	0.10516048	0.1461132	0	1.20446164	0.26838845	0.83758414	0.87749747	0	4.39247482	2.13503402	0.22507579	0.70898873	0
leuQ	0.57671244	-0.794076	0.50599465	0.57558032	0	2.26276077	1.17808407	0.67676675	0.74896876	0	3.92355118	1.97216002	0.31405325	0.81404427	0
rsmC	0.92771118	-0.1082524	0.76383845	0.80927666	0	1.21387924	0.27962491	0.28929227	0.37855568	0	1.30846676	0.38787728	0.18874706	0.66370038	0
holD	1.23745297	0.30737369	0.6648116	0.71972373	0	2.47172857	1.30552032	0.01610604	0.0317569	1	1.99743233	0.99814663	0.03988626	0.25602349	0
rimI	1.00003566	5.14E-05	0.58710464	0.65042418	0	1.36052344	0.44416181	0.31316596	0.40316388	0	1.36047493	0.44411037	0.45668662	0.88755478	0
yjjG	1.86480967	0.89902839	0.02633842	0.04273945	0	1.46835636	0.55420214	0.11199674	0.17119115	0	0.7874028	-0.3448262	0.67977899	0.94271237	0
prfC	0.58862238	-0.7645857	0.02918689	0.04681136	0	0.98880987	-0.016235	0.87404493	0.90538914	0	1.67987134	0.74835074	0.03622277	0.23885718	0
osmY	4.33805486	2.1170483	0.04802467	0.07337425	0	0.49825708	-1.0050378	0.40644327	0.49711398	0	0.11485726	-3.1220861	0.017181	0.13902426	0
ybaV	0.81449943	-0.2960144	0.69825664	0.74950172	0	0.73447034	-0.4452239	0.78118872	0.83242656	0	0.90174444	-0.1492095	1	1	0
ytjA	3.52413843	1.8172706	0.03959518	0.06184296	0	0.55703362	-0.8441637	0.30115486	0.39131593	0	0.15806236	-2.6614343	0.00719379	0.07309818	0
yjjU	3.41434403	1.77160843	4.17E-06	1.54E-05	1	2.08352769	1.05902827	0.01016718	0.02137429	1	0.61022781	-0.7125802	0.16046106	0.60475422	0
yjjV	1.64670848	0.71958518	0.06765743	0.0988237	0	1.15095095	0.20282636	0.57943209	0.66270223	0	0.69894032	-0.5167588	0.30397072	0.80892599	0
yjjW	0.0352715	-4.8253533	9.17E-36	4.14E-34	-1	0.09853274	-3.3432531	1.37E-14	2.90E-13	-1	2.79355122	1.48210027	7.00E-05	0.00201838	1
yjjI	0.02691136	-5.2156406	1.23E-45	1.00E-43	-1	0.04144798	-4.5925544	5.21E-26	6.78E-24	-1	1.5401664	0.62308623	0.0466618	0.2850118	0
deoC	0.01977095	-5.660474	1.74E-52	2.07E-50	-1	0.03379034	-4.8872455	1.15E-29	2.31E-27	-1	1.70909013	0.77322848	0.02922204	0.20617598	0
deoA	0.04233577	-4.5619789	1.54E-38	8.97E-37	-1	0.0443952	-4.4934525	2.87E-26	3.96E-24	-1	1.04864505	0.06852642	0.84574543	0.98157127	0
deoB	0.15489539	-2.6906339	9.18E-17	1.10E-15	-1	0.10363145	-3.2704661	2.75E-16	7.65E-15	-1	0.66904155	-0.5798323	0.09711075	0.45510989	0
deoD	0.09349586	-3.4189537	6.72E-25	1.46E-23	-1	0.08996208	-3.4745392	4.26E-19	1.92E-17	-1	0.96220386	-0.0555855	0.51603283	0.90937129	0
yjjJ	0.55696832	-0.8443328	0.01745366	0.02951487	0	0.64979033	-0.6219538	0.31267616	0.40295012	0	1.1666558	0.22237899	0.32059892	0.82104603	0
fadM	5.71519372	2.5148024	1.62E-05	5.28E-05	1	2.07255567	1.05141085	0.08609242	0.13794141	0	0.36263962	-1.4633915	0.03325318	0.22595764	0
lplA	0.84083106	-0.2501121	0.59724798	0.66000608	0	0.84600055	-0.2412695	0.79533218	0.84343948	0	1.00614807	0.00884264	0.82419629	0.97661485	0
ytjB	1.28267694	0.35915786	0.37547834	0.44643011	0	1.16004644	0.21418256	0.32703539	0.41697923	0	0.90439487	-0.1449753	0.86685706	0.98621446	0
serB	0.90593175	-0.1425257	1	1	0	1.66495999	0.73548751	0.0781504	0.1270591	0	1.83784264	0.87801325	0.05458655	0.3142646	0
radA	0.72071504	-0.4724991	0.15544865	0.20706173	0	1.30000625	0.37851856	0.40496152	0.49571328	0	1.8037729	0.85101771	0.03565978	0.23648065	0
nadR	1.10202693	0.14015947	0.80815782	0.84548391	0	0.5542588	-0.8513683	0.04620267	0.08047679	0	0.50294488	-0.9915278	0.01937017	0.15145823	0
ettA	0.23861145	-2.0672648	2.94E-10	1.92E-09	-1	0.26409	-1.9208984	1.15E-06	6.68E-06	-1	1.10677841	0.1463664	0.65929532	0.93664426	0
slt	0.48037635	-1.057763	0.00119661	0.00264007	-1	0.38373993	-1.3817992	0.00079264	0.00223099	-1	0.79883184	-0.3240363	0.46015942	0.88879788	0
trpR	0.88567146	-0.1751565	0.45245476	0.52261838	0	0.3320599	-1.5904846	0.00246578	0.00609753	-1	0.37492447	-1.4153281	0.00803624	0.07964006	0
yjjX	0.59771285	-0.7424755	0.12290753	0.16781532	0	0.75104124	-0.413036	0.35560852	0.44681093	0	1.25652516	0.32943956	0.67407925	0.94072452	0
ytjC	0.91026571	-0.1356404	0.58948561	0.65257091	0	0.8625643	-0.2132961	0.57165084	0.65545539	0	0.94759618	-0.0776557	0.85472262	0.98236299	0
queC	0.43646178	-1.1960728	0.00210626	0.004405	-1	0.49946061	-1.0015572	0.04053968	0.07206148	0	1.14433985	0.19451557	0.59611238	0.92806272	0
rob	1.136827	0.18501272	0.55894602	0.62439671	0	0.72765118	-0.4586811	0.06560206	0.10922966	0	0.64007204	-0.6436938	0.0187989	0.14777744	0
creA	0.26830981	-1.8980283	5.38E-06	1.94E-05	-1	0.35823161	-1.4810354	0.00236664	0.00590192	-1	1.33514168	0.41699285	0.45838163	0.88755478	0
creB	0.81644165	-0.2925783	0.7061154	0.75609981	0	1.00952882	0.0136821	0.60991738	0.68914917	0	1.23649843	0.30626041	0.348265	0.84573164	0
creC	1.90355936	0.92869956	0.00660262	0.01226915	0	2.29999993	1.20163382	0.00763765	0.01660964	1	1.20826278	0.27293426	0.60339235	0.92806272	0
creD	5.15579103	2.36619379	2.54E-08	1.27E-07	1	5.29848523	2.40557997	1.24E-06	7.18E-06	1	1.02767649	0.03938618	0.94684817	1	0
arcA	0.27888256	-1.8422704	9.19E-10	5.60E-09	-1	0.14555952	-2.7803189	9.77E-14	1.87E-12	-1	0.52193842	-0.9380485	0.00281786	0.03633558	0
yjjY	0.39147811	-1.3529964	0.00057526	0.00135589	-1	0.14469527	-2.7889103	9.45E-08	7.02E-07	-1	0.36961268	-1.4359138	0.00246047	0.0326828	-1
yjtD	0.6024677	-0.7310442	0.10376759	0.14440499	0	0.82895437	-0.2706354	0.62916583	0.70745135	0	1.37593163	0.46040879	0.35633834	0.84897465	0
kefF	1.41661742	0.50245019	0.22911486	0.29130695	0	1.65749368	0.72900337	0.07672097	0.1251033	0	1.17003621	0.22655318	0.43497805	0.88087171	0
ybaE	0.52201908	-0.9378256	0.01080139	0.01916824	0	0.45505588	-1.1358844	0.02092232	0.04016322	-1	0.8717227	-0.1980588	0.89198768	0.99488354	0
cof	2.67307738	1.4185016	0.02983107	0.04772322	1	3.10717917	1.63560543	0.12723941	0.19066495	0	1.16239776	0.21710383	0.85457066	0.98236299	0
ybaO	22.8125214	4.51175401	6.73E-17	8.19E-16	1	10.3209198	3.36749965	1.14E-06	6.64E-06	1	0.45242346	-1.1442544	0.01013429	0.09400856	0
mdlA	3.0868267	1.62612449	5.23E-06	1.89E-05	1	2.28564805	1.19260327	0.00987729	0.0208442	1	0.74045234	-0.4335212	0.19916052	0.67679424	0
mdlB	1.6910731	0.75793902	0.02357271	0.03863476	0	1.38852308	0.47355116	0.1568255	0.22826478	0	0.82108993	-0.2843879	0.64195402	0.93164618	0
glnK	10.4893913	3.39085905	6.48E-11	4.50E-10	1	26.9694934	4.75325651	2.23E-14	4.64E-13	1	2.57112092	1.36239746	0.02244759	0.17027457	0
amtB	4.88730589	2.2890394	4.37E-11	3.09E-10	1	9.77856947	3.28962342	6.78E-16	1.77E-14	1	2.00080979	1.00058402	0.00830287	0.08154933	0
tesB	0.87632448	-0.1904629	0.46688155	0.53661614	0	0.93971487	-0.089705	0.64910884	0.72507886	0	1.07233666	0.10075792	0.92421244	1	0
ybaY	3.80100747	1.92638186	0.03428998	0.05424744	0	0.39368483	-1.344887	0.23475989	0.31929946	0	0.10357381	-3.2712688	0.00458353	0.05143345	0
ybaZ	1.12753217	0.1731686	0.73428401	0.78303536	0	1.22748332	0.29570342	0.45850343	0.54774984	0	1.08864594	0.12253482	0.6717049	0.93979017	0
kefC	2.03106663	1.02223757	0.00386496	0.00757528	1	2.17702619	1.12235876	0.00570116	0.01279505	1	1.0718635	0.1001212	0.74938027	0.95683847	0
ffs	2.86681703	1.51944983	0.00054617	0.00129491	1	3.71635356	1.89388776	0.00047586	0.00140469	1	1.29633441	0.37443793	0.51570153	0.90937129	0
ybaA	5.29412161	2.40439133	0.00107906	0.0023999	1	1.9203844	0.94139512	0.14513569	0.21392748	0	0.36273901	-1.4629962	0.1543975	0.59362946	0
ylaB	1.76051762	0.81599967	0.0167438	0.02841235	0	1.15150348	0.20351877	0.58942098	0.67078324	0	0.65407098	-0.6124809	0.14615282	0.57857623	0
ylaC	0.90250185	-0.1479982	0.52033424	0.58825604	0	0.52513262	-0.9292463	0.0510378	0.0873832	0	0.58186321	-0.7812481	0.11105347	0.49419353	0
maa	1.87400422	0.9061242	0.0646007	0.09492392	0	2.39319599	1.25893855	0.0408923	0.07265859	0	1.27704941	0.35281435	0.61939205	0.92897598	0
hha	1.33759331	0.41963954	0.94133215	0.95649048	0	1.56715944	0.64815196	0.81267473	0.85676244	0	1.17162625	0.22851242	0.94467892	1	0
tomB	1.57455561	0.65494471	0.45863188	0.52850952	0	1.1032195	0.14171986	0.71997238	0.78518211	0	0.70065452	-0.5132249	0.28448879	0.78899581	0
acrB	0.28631992	-1.8043	4.12E-08	2.03E-07	-1	0.33526635	-1.5766204	2.81E-05	0.00011712	-1	1.17095015	0.22767966	0.6616737	0.93664426	0
acrA	0.43933428	-1.186609	0.00028214	0.00071049	-1	0.444235	-1.170605	0.0018066	0.00463609	-1	1.01115489	0.016004	0.85144139	0.98212032	0
acrR	0.72858268	-0.4568354	0.18743232	0.24428697	0	0.63510116	-0.6549417	0.16242912	0.23556754	0	0.87169401	-0.1981063	0.67876265	0.94189531	0
folA	0.7753056	-0.367163	0.45890943	0.52869163	0	1.06424692	0.08983292	0.89916274	0.92318776	0	1.37268056	0.45699593	0.39815232	0.86854717	0
mscK	0.85675319	-0.2230484	0.45048522	0.52075196	0	1.25084007	0.32289734	0.5446187	0.62968866	0	1.45997714	0.54594578	0.20403963	0.68323066	0
ybaM	0.22619691	-2.1443489	0.00184103	0.00389642	-1	0.48920419	-1.0314913	0.19242306	0.27114716	0	2.16273598	1.11285756	0.17524255	0.63397838	0
priC	0.39491915	-1.3403708	0.01144321	0.02018557	-1	0.57336415	-0.8024764	0.2456831	0.33109899	0	1.451852	0.53789439	0.26163753	0.7605509	0
ybaN	1.40846564	0.49412437	0.43982037	0.51056152	0	1.6547714	0.72663193	0.28814927	0.37761911	0	1.17487524	0.23250757	0.72109624	0.95399541	0
apt	0.90277247	-0.1475657	0.63866192	0.69602015	0	2.12933332	1.0904018	0.16987982	0.24500617	0	2.35866	1.23796747	0.07055157	0.36864032	0
dnaX	0.42642394	-1.2296397	0.00025231	0.00064346	-1	0.72170726	-0.4705143	0.18064932	0.2568985	0	1.69246423	0.75912534	0.06832232	0.36214114	0
ybaB	0.13951166	-2.8415424	1.30E-17	1.67E-16	-1	0.17102915	-2.5476858	6.02E-10	6.62E-09	-1	1.22591295	0.29385654	0.33125218	0.83208693	0
recR	0.21758738	-2.2003332	8.98E-11	6.10E-10	-1	0.37277161	-1.4236361	0.00046657	0.00138283	-1	1.71320417	0.77669709	0.03353919	0.22755048	0
htpG	0.26579192	-1.9116309	2.89E-09	1.66E-08	-1	0.33248683	-1.5886309	1.61E-05	7.12E-05	-1	1.25092904	0.32299996	0.50259388	0.90598897	0
adk	0.26919804	-1.8932602	1.36E-07	6.14E-07	-1	0.65347804	-0.6137893	0.06433988	0.10745198	0	2.42749929	1.27947087	0.00532947	0.05817561	0
apaH	1.15914724	0.21306383	0.60525809	0.6670565	0	1.04613576	0.06507009	0.94440583	0.96203996	0	0.90250464	-0.1479937	0.68294155	0.94348666	0
hemH	0.65097028	-0.6193364	0.05492776	0.08257859	0	0.47704346	-1.0678074	0.01824615	0.03561383	-1	0.7328191	-0.448471	0.36420539	0.85329623	0
aes	0.3796269	-1.3973459	0.00080512	0.00184261	-1	0.57866802	-0.7891922	0.04935697	0.0850657	0	1.52430722	0.6081537	0.35480205	0.84806343	0
gsk	1.02750106	0.03913988	0.86255161	0.89120245	0	2.02774796	1.01987834	0.02923595	0.05389807	0	1.9734753	0.98073846	0.03000491	0.21003439	0
ybaL	0.86339888	-0.2119009	0.47138411	0.54108722	0	0.70580884	-0.5026506	0.21923633	0.30199667	0	0.81747713	-0.2907497	0.4890504	0.90314583	0
fsr	0.95589344	-0.0650783	0.94266509	0.95740439	0	0.72207647	-0.4697765	0.44764988	0.53725281	0	0.75539431	-0.4046982	0.47623134	0.89855925	0
ushA	0.52078007	-0.9412538	0.00327252	0.0065229	0	0.33419745	-1.5812273	0.00010836	0.00038677	-1	0.64172473	-0.6399735	0.11476864	0.50161515	0
ybaK	1.19951749	0.26245419	0.59009562	0.65308252	0	1.04497609	0.06346994	0.88442488	0.91294518	0	0.8711637	-0.1989843	0.71838031	0.95394675	0
chiX	1.02508876	0.03574883	0.9064765	0.92747434	0	1.76698664	0.82129113	0.72612562	0.79033229	0	1.72374014	0.7855423	0.78641987	0.9635071	0
ybaP	1.53318167	0.61652866	0.20158509	0.26050361	0	1.21768079	0.28413598	0.4904186	0.57865834	0	0.7942182	-0.3323927	0.58288244	0.92806272	0
ybaQ	0.29017189	-1.7850203	5.63E-06	2.02E-05	-1	0.27105115	-1.8833629	0.00024647	0.00079557	-1	0.93410548	-0.0983426	1	1	0
apaG	0.96171162	-0.0563237	0.96260632	0.97385069	0	0.60105861	-0.7344224	0.07527928	0.12307035	0	0.6249884	-0.6780987	0.07272022	0.37684625	0
copA	0.80954403	-0.3048186	0.41143877	0.48462001	0	0.73374004	-0.4466591	0.30035624	0.39060453	0	0.90636212	-0.1418405	0.74283684	0.95683847	0
glsA	45.7661682	5.5162096	1.20E-07	5.47E-07	1	8.04495951	3.00808516	0.00424072	0.00983713	1	0.17578399	-2.5081244	0.03216968	0.21995081	0
ybaT	15.50656	3.95480677	9.19E-12	7.00E-11	1	3.32630835	1.73392191	0.00436163	0.01007004	1	0.21450975	-2.2208849	0.00103224	0.01701339	-1
cueR	1.07219295	0.10056455	0.80263384	0.84070042	0	0.49521332	-1.013878	0.03238499	0.0591605	0	0.46186959	-1.1144425	0.015159	0.12645175	0
ybbJ	0.8379103	-0.2551323	0.51939609	0.58749616	0	0.57424212	-0.8002689	0.20850087	0.28928268	0	0.68532648	-0.5451367	0.4353715	0.88087171	0
qmcA	0.58478457	-0.7740228	0.0239998	0.03921832	0	0.29066288	-1.7825813	0.00013928	0.0004831	-1	0.49704266	-1.0085584	0.0362348	0.23885718	0
fetA	0.86502107	-0.2091928	0.52027926	0.58825604	0	0.4994874	-1.0014798	0.08276867	0.13343844	0	0.57742802	-0.792287	0.18222267	0.65078561	0

fetB	0.68463202	-0.5465993	0.29231512	0.35995425	0	0.87076214	-0.1996494	0.78634059	0.83690729	0	1.27186885	0.34694992	0.48553159	0.90075713	0
ybbN	0.34790963	-1.5232155	8.61E-06	3.00E-05	-1	0.34306378	-1.5434513	0.00017835	0.00059751	-1	0.98607151	-0.0202358	0.9711897	1	0
ybbO	0.5940928	-0.7512398	0.04800571	0.07337066	0	0.80257918	-0.3172844	0.44184518	0.53173035	0	1.35093234	0.43395542	0.36710969	0.85342842	0
yaaA	0.20095915	-2.3150258	1.53E-09	9.00E-09	-1	0.29018451	-1.7849576	6.36E-05	0.00024035	-1	1.44399746	0.5300682	0.25981616	0.75892335	0
rsmA	0.68694156	-0.5417407	0.08149627	0.11636122	0	0.68164012	-0.5529178	0.0759707	0.12406302	0	0.99228254	-0.0111771	0.70574336	0.94896888	0
tesA	0.77770484	-0.3627054	0.44957745	0.51983863	0	0.68091766	-0.5544477	0.19648426	0.27572687	0	0.87554767	-0.1917424	0.52435006	0.91290318	0
ybbA	2.06448704	1.04578336	0.00842414	0.01530623	1	2.23779856	1.16208018	0.00478671	0.01095416	1	1.08394896	0.11629682	0.56861812	0.92771214	0
ybbP	2.11799365	1.08269826	0.00322843	0.00644665	1	2.16939567	1.1172932	0.00139313	0.00369966	1	1.0242692	0.03459494	0.54773673	0.91914725	0
rhsD	2.34837616	1.23166351	0.00053049	0.00126046	1	1.9144601	0.93693759	0.00762582	0.01660025	0	0.81522719	-0.2947259	0.76743275	0.96147115	0
ybbC	3.38905573	1.76088336	0.02794351	0.04502972	1	2.45405155	1.29516555	0.15623674	0.22763276	0	0.72411071	-0.4657178	0.73614606	0.95683847	0
rhsH	6.05505397	2.59813982	3.72E-06	1.38E-05	1	6.66047401	2.73562485	6.57E-06	3.21E-05	1	1.0999859	0.13748503	0.6399338	0.93163024	0
ybbD	8.58091579	3.10113163	0.00018862	0.0004965	1	4.8147548	2.26746233	0.01338545	0.02698463	1	0.56110034	-0.8336693	0.39296953	0.86854717	0
ylbI	9.01554701	3.17241503	0.01255891	0.02197809	1	3.91423099	1.96872889	0.08933844	0.14206066	0	0.43416456	-1.2036861	0.6199703	0.92907233	0
ylbG	0.96218123	-0.0556194	0.81088809	0.84733681	0	1.18410813	0.24380084	0.4344225	0.52522928	0	1.2306498	0.29942028	0.35681201	0.84936009	0
mnmH	1.25334657	0.3257854	0.25588316	0.32072529	0	1.45732443	0.54332208	0.11841746	0.17952536	0	1.16274657	0.21753668	0.48812178	0.90208042	0
pdxA	1.12136654	0.16525793	0.67770402	0.73099356	0	1.05183084	0.0729027	0.84988184	0.8865977	0	0.93799021	-0.0923552	0.84416155	0.98070401	0
allS	3.15853692	1.65925644	0.05238981	0.07924856	0	3.06972113	1.6181076	0.05364529	0.09138725	0	0.97188072	-0.0411488	0.83391745	0.98014902	0
allA	1.53722523	0.62032856	0.22282714	0.2842882	0	1.8547797	0.89124784	0.08500211	0.13659066	0	1.20657641	0.27091928	0.50177007	0.90598897	0
allR	0.72239361	-0.469143	0.12021268	0.16454274	0	0.3973231	-1.3316154	0.00018955	0.00062883	-1	0.55000916	-0.8624725	0.00840108	0.08214799	0
gcl	3.51929681	1.81528719	0.00078184	0.0017977	1	4.59862964	2.20120401	5.87E-05	0.00022371	1	1.30668991	0.38591682	0.27468501	0.77751019	0
hyi	8.77584272	3.13353767	1.26E-09	7.52E-09	1	5.69512302	2.50972701	3.64E-06	1.87E-05	1	0.64895455	-0.6238107	0.45532155	0.88755478	0
glxR	5.33556011	2.41563973	6.87E-08	3.24E-07	1	5.40811903	2.4351269	2.57E-06	1.39E-05	1	1.01359912	0.01948718	0.89385346	0.9954996	0
ybbW	10.8153584	3.43500957	6.33E-15	6.45E-14	1	7.78797383	2.96124804	1.00E-10	1.21E-09	1	0.72008467	-0.4737615	0.57532135	0.92806272	0
allB	3.61626073	1.8544987	5.18E-05	0.00015542	1	3.29659735	1.72097768	0.00038801	0.0011825	1	0.91160389	-0.133521	1	1	0
ybbY	5.98728387	2.58190167	3.78E-05	0.00011591	1	6.0768675	2.60332784	4.04E-05	0.00016182	1	1.01496232	0.02142616	0.65215632	0.9347567	0
glxK	6.30680344	2.65690897	2.92E-05	9.11E-05	1	6.13831198	2.61784197	7.29E-05	0.00027133	1	0.97328417	-0.039067	0.76789013	0.96147692	0
surA	0.55596964	-0.846922	0.0037827	0.00741927	0	0.5652894	-0.8229384	0.01393672	0.02796838	0	1.01676307	0.02398354	0.88655261	0.99356976	0
allE	0.63468997	-0.655876	0.1525318	0.20372935	0	0.67166342	-0.5741896	0.75035117	0.80774709	0	1.05825434	0.08168641	0.40108401	0.86854717	0
allC	3.34698909	1.74286385	3.84E-05	0.00011776	1	2.67952636	1.42197801	0.00044765	0.0013366	1	0.80057816	-0.3208858	0.89553769	0.99588104	0
allD	4.171248	2.06047909	0.00015847	0.00042396	1	4.3651737	2.12603906	0.00056033	0.00163438	1	1.04649105	0.06555997	0.77843758	0.9635071	0
fdrA	5.45714856	2.44814732	0.00061328	0.00143705	1	8.5221564	3.09121853	4.48E-05	0.00017724	1	1.56165007	0.64307121	0.25067062	0.7487007	0
ylbE	5.49573342	2.45831203	0.00133025	0.00290733	1	8.14667802	3.02621189	0.00042732	0.00128195	1	1.48236412	0.56789986	0.4039549	0.86854717	0
ylbF	5.89505504	2.55950528	0.00791279	0.01446041	1	10.9470683	3.45247265	0.00281087	0.00682485	1	1.8569917	0.89296737	0.39656463	0.86854717	0
ybcF	4.98268636	2.31692376	0.00199873	0.004202	1	6.78337344	2.76200292	0.00129239	0.00346757	1	1.36138881	0.44507975	0.5135335	0.90937129	0
purK	1.94952127	0.9631199	0.04671053	0.07162904	0	3.12963424	1.64599406	0.00329642	0.00786973	1	1.60533475	0.68287416	0.18239705	0.65078561	0
purE	3.4510696	1.78704357	8.58E-05	0.0002431	1	4.34494498	2.11933791	0.00014286	0.00049359	1	1.25901401	0.33229434	0.56312432	0.92432422	0
lpxH	1.39447579	0.47972289	0.28142692	0.34848968	0	1.67566886	0.74473708	0.10612278	0.16425799	0	1.20164786	0.26501418	0.49403339	0.90539057	0
lptD	0.51068893	-0.9694833	0.00277042	0.00562878	0	0.43598194	-1.1976597	0.00173809	0.00449158	-1	0.85371331	-0.2281764	0.4412326	0.88087171	0
ppiB	0.22086316	-2.1787753	3.47E-12	2.74E-11	-1	0.23640344	-2.080677	1.20E-08	1.05E-07	-1	1.07036158	0.09809824	1	1	0
cysS	0.6468683	-0.6284561	0.06682682	0.09780453	0	1.01732683	0.02478324	0.88700584	0.91453075	0	1.57269546	0.65323933	0.0835771	0.41227628	0
ybcI	5.26543463	2.39655262	0.00338607	0.00672192	1	7.14507018	2.83694818	0.00170028	0.00440162	1	1.35697633	0.44039556	0.49746036	0.90539057	0
ybcJ	0.39297656	-1.3474848	0.0103322	0.01841701	-1	0.74197783	-0.430552	0.55965619	0.64387417	0	1.88809692	0.91693282	0.09825372	0.45734597	0
folD	0.25950475	-1.9461672	2.10E-07	9.25E-07	-1	0.39385975	-1.3442461	0.00442359	0.01018648	-1	1.51773618	0.60192104	0.08259164	0.40863385	0
sfmA	4.50459192	2.17139642	0.10450786	0.14534359	0	3.56708127	1.83474409	0.1125887	0.17190536	0	0.79187667	-0.3366523	0.97694101	1	0
sfmC	4.4781213	2.16289361	0.01476996	0.02540457	1	5.54376181	2.47086527	0.0059612	0.01330297	1	1.23796598	0.30797166	0.52203642	0.91269012	0
sfmD	6.86404418	2.77905884	1.33E-05	4.44E-05	1	8.19686463	3.03507217	1.01E-05	4.73E-05	1	1.19417422	0.25601333	0.52265167	0.91269012	0
sfmH	7.00579484	2.80854874	2.17E-05	6.95E-05	1	9.92306976	3.3107865	3.97E-06	2.02E-05	1	1.41640848	0.50223776	0.33083605	0.83208693	0
sfmF	7.27517983	2.86298291	0.00010884	0.00030102	1	7.04985541	2.81759367	0.00031271	0.00097936	1	0.96902834	-0.0453892	0.86360357	0.98512461	0
djlA	0.84468008	-0.2435231	0.42848997	0.5003014	0	0.8906224	-0.1671142	0.84787889	0.88534424	0	1.05439021	0.07640888	0.68215295	0.94348666	0
sfmZ	6.6806078	2.73997936	0.00487075	0.00928934	1	3.78200986	1.91915312	0.06698079	0.11119012	0	0.56611763	-0.8208262	0.52693587	0.91534998	0
argU	1.65917177	0.73046325	0.51244255	0.58141761	0	1.6018424	0.67973221	0.80982986	0.85474748	0	0.96544699	-0.050731	0.80464227	0.96990832	0
intD	0.43527507	-1.2000007	0.00535521	0.01014757	-1	0.40863644	-1.2911102	0.01289045	0.02616578	-1	0.93880046	-0.0911095	0.91004508	0.99971546	0
xisD	3.64130372	1.86445508	0.2860915	0.353078	0	3.4924366	1.80423393	0.25124719	0.33716749	0	0.95911708	-0.0602212	0.81419794	0.9712234	0
exoD	5.80675949	2.53773328	0.01505355	0.0258019	1	3.35113085	1.74464802	0.07208451	0.11866437	0	0.5771086	-0.7930853	0.7007958	0.94869712	0
peaD	10.2773829	3.36140103	0.03525529	0.05563896	0	6.11954192	2.61342366	0.10485181	0.16267795	0	0.59543777	-0.7479774	0.79127116	0.96528515	0
renD	4.45009601	2.15383646	0.00172434	0.00367057	1	3.24385506	1.69770936	0.03111526	0.05714811	0	0.72894047	-0.4561271	0.61660927	0.92897598	0
emrE	0.31020582	-1.6887024	0.01989702	0.03310174	-1	0.34138471	-1.5505297	0.08017987	0.12969065	0	1.10051034	0.13817269	0.88341259	0.99247405	0
yabP	0.69688847	-0.5210003	0.29502429	0.36288563	0	0.69570473	-0.523453	0.26631623	0.35351499	0	0.99830139	-0.0024527	0.8513783	0.98212032	0
ybcK	5.34441095	2.41803095	0.00010837	0.00029991	1	3.98784656	1.9956099	0.0042793	0.00990583	1	0.74617139	-0.422421	0.58345138	0.92806272	0
ybcL	2.95857375	1.56490186	0.04036419	0.0629328	0	0.73639935	-0.4414397	1	1	0	0.2489035	-2.0063416	0.07598808	0.38578363	0
ybcM	1.41054684	0.49625457	0.66712474	0.72152056	0	0.49226478	-1.0224936	0.20198821	0.2813953	0	0.34898861	-1.5187481	0.06254423	0.34136143	0
ylcH	7.48841879	2.90466112	0.15737281	0.20924615	0	3.02283923	1.59590425	0.4641738	0.55379079	0	0.40366856	-1.3087569	0.61709949	0.92897598	0
ybcN	5.2072397	2.38051882	0.01750778	0.0295611	1	4.45949336	2.15687982	0.02008557	0.038759	1	0.85640255	-0.223639	0.91635973	1	0
ninE	6.68099527	2.74006304	0.08490522	0.12062604	0	7.44511437	2.89629401	0.10194026	0.15917809	0	1.11437205	0.15623097	0.8081094	0.96990832	0
ybcO	13.0497749	3.70595301	3.92E-06	1.45E-05	1	9.27118687	3.21275404	2.56E-05	0.00010763	1	0.71044803	-0.493199	0.96026681	1	0
rusA	10.6755022	3.41623203	2.23E-05	7.11E-05	1	11.471182	3.51994215	0.00010954	0.00039007	1	1.07453325	0.10371012	0.83105435	0.97888613	0
ylcG	4.66336197	2.22137042	0.02379057	0.0389629	1	5.94631174	2.5719951	0.03728445	0.06692119	0	1.27511263	0.35062469	0.78480656	0.9635071	0
quuD	5.43567021	2.44245793	0.001441	0.00312463	1	4.23097334	2.0809896	0.01722217	0.03380931	1	0.77837197	-0.3614683	0.7543653	0.9573569	0
rluA	3.39612489	1.76388952	7.55E-05	0.00021756	1	3.53559125	1.82195149	0.00497819	0.0113183	1	1.04106632	0.05806198	0.69742226	0.94869712	0
nmpC	0.04266572	-4.5507787	7.45E-37	3.57E-35	-1	0.06892065	-3.8589199	1.60E-21	1.03E-19	-1	1.61536336	0.69185872	0.18518803	0.65544077	0
essD	5.11054004	2.35347575	0.0034303	0.00680361	1	7.98364234	2.99704709	0.00604475	0.01346358	1	1.56219153	0.64357134	0.69457794	0.94773784	0
rrrD	3.75566984	1.90907024	0.0086275	0.01563712	1	1.97084178	0.97881196	0.06307565	0.1054602	0	0.52476439	-0.9302583	0.54636111	0.91914725	0
rzpD	0.32110139	-1.6388992	0.04024543	0.06276979	0	0.35834691	-1.4805712	0.14853669	0.2180686	0	1.11599301	0.158328	0.81160248	0.96998188	0
borD	0.05446156	-4.1986178	9.78E-23	1.77E-21	-1	0.05737075	-4.1235407	1.69E-15	4.22E-14	-1	1.05341728	0.07507703	0.81542974	0.9719041	0
ybcV	3.73118039	1.89963211	0.35238789	0.4226167	0	8.49174275	3.08606067	0.17396492	0.24943251	0	2.27588641	1.18642856	0.55878632	0.92363107	0
ybcW	0.66261353	-0.5937604	0.43833646	0.50937442	0	0.46829217	-1.0945192	0.50405266	0.59208099	0	0.706735	-0.5007587	0.87151632	0.9875095	0
ylcI	0.75249143	-0.4102529	0.88403853	0.90935705	0	1.2976837	0.37593878	0.81213256	0.8565188	0	1.72451625	0.78619172	0.69093402	0.94569182	0
rapA	2.72301551	1.4452052	7.02E-06	2.47E-05	1	1.97050617	0.97856627	0.04107164	0.07283129	0	0.72364853	-0.4666389	0.09633911	0.45439087	0
nohD	2.95875061	1.5649881	0.55778088	0.62356825	0	7.68465181	2.94197989	0.28661849	0.37616115	0	2.59726243	1.37699179	0.56304559	0.92432422	0
aaaD	9.55939267	3.25691896	0.00178583	0.00378867	1	6.78703601	2.76278167	0.01180478	0.02427422	1	0.70998611	-0.4941373	0.8477184	0.98167799	0
tfaD	8.7833552	3.13477215	0.00031852	0.00079174	1	3.47240423	1.7959349	0.03345499	0.06088864	0	0.39533916	-1.3388372	0.25034213	0.7487007	0
ybcY	7.59795276	2.92561074	8.73E-06	3.04E-05	1	5.10014858	2.35053928	0.00233835	0.00584455	1	0.671253	-0.5750715	0.40451316	0.86854717	0
tfaX	2.4352481	1.28406876	0.35850534	0.4291377	0	1.98361151	0.9881295	0.3506381	0.44157018	0	0.81454186	-0.2959393	1	1	0
appY	1.42597247	0.51194613	0.41777146	0.49063986	0	0.53408182	-0.9048673	0.02815927	0.05213063	0	0.37453866	-1.4168134	0.00301354	0.03797056	-1
ompT	0.11285594	-3.1474458	1.86E-21	3.11E-20	-1	0.10583528	-3.2401075	2.19E-17	7.44E-16	-1	0.93779098	-0.0926617	0.33479032	0.83443729	0
pauD	5.22757766	2.38614259	0.11276415	0.15550398	0	10.5486565	3.39898736	0.03202858	0.05867922	0	2.01788615	1.01284478	0.42275904	0.87468118	0
envY	2.74155874	1.45499638	0.00638798	0.01190535	1	2.75515353	1.46213271	0.02312872	0.04378998	1	1.00495878	0.00713633	1	1	0
ybcH	2.44375658	1.28910059	0.05003677	0.07605382	0	1.84160213	0.88096141	0.14545771	0.21418781	0	0.75359475	-0.4081392	0.81406966	0.9712234	0
polB	2.095434	1.06724908	0.00040471	0.00098328	1	2.42671498	1.27900467	0.00089668	0.00250108	1	1.15809659	0.21175559	0.62795185	0.93095788	0
nfrA	1.78976595	0.83977094	0.02694909	0.04361822	0	1.76177867	0.81703269	0.02428985	0.04577264	0	0.9843626	-0.0227382	0.74188882	0.95683847	0
nfrB	8.06562935	3.01178711	1.37E-17	1.76E-16	1	4.27745691	2.09675332	1.77E-06	9.86E-06	1	0.53033145	-0.9150338	0.00875408	0.08472569	0
cusS	3.26769331	1.70827259	0.00017839	0.00047155	1	3.29874025	1.72191518	0.00027685	0.00087889	1	1.00950118	0.0136426	0.68774779	0.94389281	0
cusR	2.2739513	1.18520136	0.00161223	0.00345522	1	3.76234716	1.91163298	0.00015231	0.00052053	1	1.65454166	0.72643162	0.18131846	0.64917581	0
cusC	5.59197501	2.48335791	0.00147859	0.00319829	1	4.7671619	2.25313062	0.0035161	0.00834012	1	0.85250057	-0.2302273	1	1	0
cusF	56.2466373	5.81369494	1.34E-40	8.94E-39	1	19.4962117	4.28512191	1.41E-16	4.07E-15	1	0.34662004	-1.528573	3.40E-05	0.00110274	-1
cusB	27.1559431	4.76319606	6.76E-31	2.39E-29	1	14.4699833	3.85499135	3.65E-17	1.20E-15	1	0.53284775	-0.9082047	0.02536632	0.18613225	0
cusA	9.87482164	3.30375469	1.09E-08	5.76E-08	1	9.27382324	3.21316423	2.21E-07	1.52E-06	1	0.9391383	-0.0905905	0.90527061	0.99890653	0
pheP	1.58539242	0.66483998	0.10142845	0.14150646	0	2.00602099	1.0043367	0.01262913	0.02568252	1	1.26531511	0.33949672	0.27334184	0.77568242	0
ybdG	0.89747556	-0.1560554	0.65198536	0.70774622	0	1.08644425	0.11961415	0.81861487	0.86129979	0	1.21055582	0.2756696	0.53281147	0.91800048	0
araD	2.33694551	1.22462409	0.0093205	0.01678297	1	3.90412145	1.96499793	0.00064569	0.00186003	1	1.67060868	0.74037384	0.1830614	0.65210078	0
nfsB	0.17243244	-2.5358969	1.90E-14	1.84E-13	-1	0.22213371	-2.1704998	8.47E-08	6.33E-07	-1	1.2882362	0.36539714	0.28223174	0.78575882	0
ybdF	1.9572151	0.96880232	0.01360426	0.02367612	0	0.79402909	-0.3327362	0.15314772	0.22394433	0	0.40569332	-1.3015386	0.00052736	0.01006778	-1

ybdJ	74.6354472	6.22178908	1.71E-19	2.44E-18	1	19.7395032	4.30301377	1.20E-07	8.75E-07	1	0.26447893	-1.9187753	0.00129945	0.02061354	-1
ybdK	1031.45985	10.0104719	5.46E-99	8.05E-96	1	131.725838	7.04139455	5.08E-48	7.49E-45	1	0.12770816	-2.9690774	3.02E-14	1.33E-11	-1
sokE	2.01217237	1.0087539	0.35631676	0.42698077	0	5.34930528	2.41935154	0.22637014	0.31056039	0	2.65847268	1.41059764	0.5717466	0.92806272	0
hokE	0.77353646	-0.3704588	0.68872404	0.74088994	0	1.60469089	0.68229542	0.38836061	0.47937585	0	2.07448642	1.05275421	0.24072673	0.73370247	0
entD	1.33235515	0.41397869	0.57675181	0.64152927	0	1.77398757	0.8269959	0.17214214	0.24730043	0	1.33146749	0.41301721	0.39972172	0.86854717	0
fepA	2.12213234	1.08551463	0.00060209	0.00141385	1	2.68064919	1.42258243	0.00014562	0.00050234	1	1.26318663	0.3370678	0.31353318	0.81404427	0
fes	5.29620263	2.40495832	9.77E-07	3.95E-06	1	5.79016439	2.53360431	7.98E-07	4.81E-06	1	1.09326716	0.12864599	0.51060323	0.90925684	0
yaaJ	1.12161227	0.16557404	0.7007918	0.75160271	0	1.04073701	0.05760555	0.82754827	0.86925025	0	0.92789374	-0.1079685	0.87481985	0.98871234	0
ybdZ	1.80572548	0.85257858	0.1413494	0.19028963	0	3.2332665	1.69299242	0.03405604	0.06180436	0	1.7905637	0.84041385	0.31591899	0.81560659	0
entF	1.32355826	0.4044217	0.21998115	0.28117162	0	1.55278575	0.63485878	0.04410372	0.07730577	0	1.17319033	0.23043708	0.33808075	0.83714543	0
fepE	6.66766127	2.73718081	1.06E-06	4.27E-06	1	5.74139082	2.52140026	3.11E-05	0.00012789	1	0.86108016	-0.2157806	0.89561547	0.99588104	0
fepC	2.72760134	1.4476328	0.0004306	0.0010365	1	2.85535531	1.51367028	0.00060646	0.0017562	1	1.04683748	0.06603748	0.66667513	0.93840962	0
fepG	1.78656331	0.83718704	0.0842923	0.11990953	0	2.64148619	1.40134987	0.01227693	0.0250687	1	1.4785293	0.56416283	0.28214227	0.78575882	0
fepD	2.24166412	1.16457013	0.00783994	0.01435103	1	2.48556538	1.31357405	0.02220108	0.04234206	1	1.10880366	0.14900393	0.90005219	0.99683105	0
entS	1.98402533	0.98843044	0.02341403	0.03840318	0	2.53689736	1.34306515	0.00374719	0.00881258	1	1.27866178	0.35463471	0.31342109	0.81404427	0
fepB	1.91788295	0.93951467	0.03643126	0.05726579	0	1.75086059	0.80806422	0.11265226	0.17190536	0	0.91291316	-0.1314505	0.86011638	0.98424873	0
entC	1.48649193	0.57191163	0.16177569	0.21451913	0	1.76188659	0.81712106	0.06124649	0.10290808	0	1.18526482	0.24520943	0.4916567	0.90539057	0
entE	1.47546403	0.56116875	0.06099926	0.09033433	0	2.42582135	1.27847331	0.00249057	0.00613395	1	1.64410742	0.71730456	0.10467343	0.47522837	0
entB	1.02060617	0.02942627	0.89684887	0.9201678	0	1.91365627	0.93633172	0.02800009	0.05190117	0	1.8750193	0.90690545	0.03088932	0.21452268	0
entA	0.73563192	-0.442944	0.72027076	0.76902005	0	1.71086947	0.77472969	0.08749674	0.13953377	0	2.32571401	1.2176737	0.02476604	0.18356008	0
entH	1.00563724	0.00810997	0.99454654	0.99929756	0	1.75133739	0.80845704	0.15231748	0.22287779	0	1.74152003	0.80034707	0.14210705	0.57023846	0
cstA	0.54892654	-0.865315	0.00570159	0.01073941	0	0.29790799	-1.7470613	1.08E-05	5.01E-05	-1	0.54271012	-0.8817463	0.0232237	0.17358733	0
ybdD	0.42526937	-1.2335511	0.00244078	0.00501439	-1	0.08890736	-3.4915533	1.75E-08	1.48E-07	-1	0.20906128	-2.2580022	0.00028535	0.00616868	-1
ybdH	0.94941227	-0.0748934	0.97444142	0.98312191	0	0.53304087	-0.9076819	0.1060459	0.16419651	0	0.561443	-0.8327885	0.10928998	0.48930843	0
ybdL	1.23780622	0.30778548	0.70335412	0.75369121	0	1.08418881	0.11661602	0.60731596	0.68707865	0	0.87589543	-0.1911695	0.93111475	1	0
ybdM	1.70537134	0.77008592	0.17769277	0.23282971	0	0.67637502	-0.5641047	0.8629421	0.89629718	0	0.39661451	-1.3341906	0.15144036	0.58738081	0
ybdN	5.36821585	2.42444268	0.00840005	0.01527502	1	4.15654067	2.05538333	0.01406451	0.02818642	1	0.77428717	-0.3690594	1	1	0
ybdO	3.36390229	1.7501358	0.07805489	0.11179262	0	3.05555185	1.61143296	0.16673574	0.24112779	0	0.9083355	-0.1387028	0.90061447	0.99683105	0
araC	2.72658225	1.44709367	0.74175204	0.78973659	0	1.44459598	0.53066606	0.76424129	0.819108	0	0.52981933	-0.9164276	1	1	0
dsbG	1.4811095	0.5666783	0.10780264	0.14936144	0	0.8905009	-0.167311	0.79162007	0.84110822	0	0.60123907	-0.7339893	0.11467125	0.50161515	0
ahpC	0.08118682	-3.6226107	1.13E-28	3.26E-27	-1	0.09955859	-3.3283104	1.96E-18	8.18E-17	-1	1.22629004	0.29430025	0.71347885	0.95223057	0
ahpF	0.16894565	-2.5653689	8.33E-15	8.44E-14	-1	0.20981655	-2.2527996	1.14E-08	1.00E-07	-1	1.24191747	0.31256931	0.3683796	0.85457865	0
uspG	0.14486111	-2.7872578	7.03E-17	8.51E-16	-1	0.09400019	-3.4111926	3.69E-17	1.20E-15	-1	0.64889871	-0.6239348	0.03467151	0.23131824	0
ybdR	2.82867856	1.50012825	3.26E-05	0.00010098	1	2.72761139	1.44763811	0.00080316	0.00225774	1	0.96427053	-0.0524901	0.94102205	1	0
rnk	0.33240624	-1.5889806	8.38E-05	0.00023901	-1	0.69672976	-0.5213289	0.1741039	0.24955081	0	2.09601893	1.06765174	0.03513764	0.23400856	0
rna	0.57527145	-0.7976852	0.06023255	0.08932043	0	0.57079814	-0.8089475	0.03100185	0.0570109	0	0.992224	-0.0112622	0.60582914	0.92806272	0
citT	5.9447243	2.5716099	0.00376273	0.00738337	1	6.1580603	2.62247599	0.0040717	0.00948989	1	1.03588661	0.05086609	0.73554027	0.95683847	0
citG	5.50762174	2.46142948	0.00315669	0.00632628	1	4.97931567	2.31594748	0.00594396	0.01327925	1	0.90407728	-0.145482	0.85284078	0.98236299	0
citX	3.33250261	1.73660601	0.00236982	0.00487994	1	4.23537759	2.08249059	0.00137523	0.00365872	1	1.27093001	0.34588459	0.49431665	0.90539057	0
yabI	2.37845671	1.25002577	0.00142033	0.00308284	1	2.26376468	1.17872399	0.00345779	0.00821504	1	0.9517788	-0.0713018	0.82824765	0.97793095	0
citF	4.89529854	2.29139684	0.00030273	0.00075502	1	5.49082302	2.45702241	0.00031651	0.00098916	1	1.12165233	0.16562557	0.61222655	0.92897598	0
citE	4.74603952	2.24672411	4.10E-06	1.51E-05	1	5.96293528	2.57602268	4.45E-07	2.82E-06	1	1.25640236	0.32929856	0.29361201	0.7982916	0
citD	6.98333028	2.80391521	8.23E-05	0.00023522	1	5.04787818	2.33567709	0.00253816	0.00623119	1	0.72284683	-0.4682381	0.70580905	0.94896888	0
citC	6.12987623	2.61585794	6.29E-05	0.00018516	1	5.71789363	2.51548378	0.0003004	0.00094616	1	0.93279104	-0.1003742	0.89618388	0.99626189	0
citA	2.53418507	1.34152189	0.00012129	0.00033192	1	3.09443282	1.629675	7.94E-05	0.00029254	1	1.2210761	0.28815312	0.42962223	0.87797234	0
citB	0.11278197	-3.1483916	1.56E-13	1.38E-12	-1	0.13897292	-2.8471243	1.43E-07	1.02E-06	-1	1.23222639	0.30126734	0.21877363	0.70102268	0
dcuC	0.02164216	-5.530012	1.78E-50	1.91E-48	-1	0.01616991	-5.9505445	8.91E-36	3.94E-33	-1	0.74714878	-0.4205325	0.84075459	0.98067952	0
pagP	2.94293712	1.55725672	0.19468991	0.25215448	0	1.12981855	0.17609109	0.51774271	0.6049458	0	0.38390849	-1.3811656	0.50451544	0.90701715	0
cspE	0.16849079	-2.5692584	0.0002596	0.00065974	-1	0.50381139	-0.9890443	0.07445487	0.12199335	0	2.99014209	1.58021404	0.19123791	0.66643604	0
flc	7.61155088	2.92819044	1.51E-13	1.35E-12	1	2.41274025	1.27067261	0.02704075	0.05033407	0	0.31698405	-1.6575178	5.81E-05	0.00176769	-1
thiQ	2.97233795	1.57159816	0.0051078	0.00972045	1	2.97863382	1.57465078	0.01611128	0.0317569	1	1.00211815	0.00305262	1	1	0
ybeM	1.7643792	0.81916065	0.04777141	0.07308843	0	1.08719397	0.12060936	0.52340875	0.61075872	0	0.61619066	-0.6985513	0.25411169	0.75391328	0
tatE	0.61152044	-0.7095274	0.08901187	0.12569227	0	0.2824157	-1.8241078	0.00141999	0.00376647	-1	0.46182544	-1.1145804	0.05221039	0.30577401	0
lipA	0.1763432	-2.5035421	8.85E-15	8.91E-14	-1	0.4164471	-1.2637948	0.00041383	0.00124741	-1	2.36157161	1.23974728	0.00090347	0.01538334	1
ybeF	2.16671199	1.11550739	0.06291116	0.09284283	0	2.76370929	1.46660587	0.02108295	0.04043644	1	1.27553145	0.35109847	0.49778452	0.90539057	0
lipB	1.91040241	0.93387656	0.03281318	0.05204159	0	2.2511793	1.17068097	0.01967286	0.03804568	1	1.17837964	0.23680441	0.6125106	0.92897598	0
ybeD	0.69414095	-0.5266995	0.31401496	0.3827274	0	1.11301236	0.15446962	0.72937279	0.79253185	0	1.60343857	0.68116908	0.61797158	0.92897598	0
dacA	0.36219979	-1.4651424	8.46E-05	0.00024078	-1	0.83109575	-0.2669134	0.26074361	0.34789432	0	2.29457822	1.19822899	0.01727377	0.13951891	0
rlpA	0.57172533	-0.8066059	0.03022571	0.04830208	0	0.68763372	-0.5402878	0.25731916	0.34394839	0	1.20273439	0.26631808	0.44448658	0.88411674	0
mrdB	2.47321252	1.30638621	0.00010073	0.00028071	1	2.13371384	1.0933667	0.01348998	0.02714582	1	0.86272968	-0.2130195	0.45589554	0.88755478	0
mrdA	1.03051735	0.04336879	0.85376003	0.88377268	0	1.34814367	0.43097425	0.22424571	0.30822451	0	1.30822026	0.38760546	0.27368749	0.77568242	0
thiP	4.31253614	2.10853655	4.35E-06	1.60E-05	1	6.09319323	2.60719849	2.73E-07	1.83E-06	1	1.41290253	0.49866195	0.20885637	0.69006731	0
rlmH	0.71254877	-0.4889393	0.46600545	0.53588807	0	1.32366907	0.40454248	0.30467679	0.3947132	0	1.857654	0.89348181	0.06537434	0.35158639	0
rsfS	0.31593921	-1.6622811	7.44E-05	0.00021484	-1	0.90223809	-0.1484199	0.42810081	0.51872155	0	2.85573317	1.51386119	0.00682205	0.07037554	0
cobC	3.28119693	1.71422219	0.02474615	0.04039311	1	2.3495993	1.23241474	0.0960714	0.15140497	0	0.71607994	-0.4818074	0.72715377	0.95487077	0
nadD	1.42595894	0.51193244	0.20916509	0.26887127	0	1.28117863	0.35747164	0.53988516	0.62536107	0	0.89846811	-0.1544608	0.66215053	0.9368251	0
holA	1.17005471	0.226576	0.55284677	0.61899219	0	1.18481755	0.24466492	0.58140488	0.66376456	0	1.01261722	0.01808893	0.96866016	1	0
lptE	0.42564434	-1.2322797	0.00049495	0.00117981	-1	0.55748734	-0.842989	0.0217401	0.04158853	0	1.30974922	0.38929061	0.53727654	0.9187668	0
leuS	0.46420989	-1.1071508	0.00031348	0.00078008	-1	0.43817409	-1.1904239	0.00097214	0.00270012	-1	0.94391374	-0.0832731	0.70870542	0.95069307	0
ybeL	0.26763014	-1.9016875	1.48E-09	8.75E-09	-1	0.10334983	-3.2743921	4.50E-16	1.21E-14	-1	0.38616662	-1.3727046	0.0001078	0.00284681	-1
ybeQ	1.20193654	0.26536072	0.67648409	0.72985594	0	0.56887491	-0.8138166	0.19961589	0.2787935	0	0.47329862	-1.0791774	0.07019129	0.36820631	0
ybeR	7.24095201	2.85617939	8.64E-05	0.0002444	1	6.42662452	2.68406118	0.00035152	0.00108324	1	0.88753862	-0.1721182	1	1	0
thiB	1.69928325	0.76492636	0.08569492	0.12159153	0	2.16192705	1.11231784	0.03464429	0.0627688	0	1.2722582	0.34739149	0.44982449	0.88755478	0
djlB	5.8463271	2.54753055	2.35E-07	1.03E-06	1	6.43016382	2.68485549	1.55E-07	1.10E-06	1	1.09986385	0.13732494	0.48563399	0.90075713	0
ybeT	4.62698405	2.21007213	0.02681409	0.04346342	1	4.97210097	2.31385559	0.01084072	0.02254359	1	1.07458788	0.10378347	0.60699143	0.92806272	0
ybeU	6.2234464	2.63771373	1.71E-05	5.57E-05	1	5.36430215	2.4233905	0.00011165	0.00039598	1	0.86195041	-0.2143232	0.98515821	1	0
djlC	4.45681759	2.15601391	3.58E-06	1.33E-05	1	3.62433942	1.85771807	0.0003172	0.00099059	1	0.81321242	-0.2982958	0.70543642	0.94896888	0
hscC	0.63527199	-0.6545537	0.1198677	0.16417229	0	0.83479677	-0.2605031	0.74284121	0.80161546	0	1.31407773	0.39405061	0.31057867	0.81168136	0
rihA	0.05942316	-4.0728309	1.39E-31	5.11E-30	-1	0.10243868	-3.2871675	2.81E-16	7.77E-15	-1	1.72388486	0.78566341	0.03805219	0.24569571	0
gltL	1.24627673	0.31762445	0.39413345	0.46622589	0	0.71969138	-0.4745497	0.47044728	0.55974867	0	0.57747317	-0.7921742	0.12893113	0.53792459	0
gltK	1.65151876	0.72379336	0.04786129	0.07320059	0	1.32753011	0.40874458	0.28669626	0.37616115	0	0.80382381	-0.3150488	0.56072638	0.92432422	0
gltJ	1.80866388	0.85492432	0.06779434	0.09895822	0	1.99800556	0.9985606	0.04637512	0.08071353	0	1.10468595	0.14363628	0.72060704	0.95399541	0
gltI	0.85414993	-0.2274388	0.546305	0.61337803	0	0.40794106	-1.2935674	0.04868783	0.08407641	0	0.47759889	-1.0661286	0.10281267	0.46993059	0
sgrR	0.91928116	-0.1214219	1	1	0	1.12513026	0.17009203	0.39448277	0.48537879	0	1.22392399	0.29151396	0.34662586	0.84500832	0
insH1	0	#NAME?	0.69801156	0.74950172	0	22.619615	4.49950247	0.32252824	0.4129969	0	Inf	Inf	0.17315604	0.63004798	0
lnt	1.46792745	0.55377772	0.10410039	0.14482249	0	1.58422326	0.66377566	0.1039577	0.16187072	0	1.0792267	0.10999794	0.78794412	0.9638928	0
ybeX	0.74626916	-0.422232	0.18041028	0.23590059	0	0.87860476	-0.1867138	0.34238181	0.43252865	0	1.17732958	0.23551824	0.92616917	1	0
ybeY	0.6414708	-0.6405445	0.05132385	0.0779029	0	0.35933184	-1.4766113	0.00022753	0.00074149	-1	0.56016865	-0.8360668	0.02286444	0.17236272	0
ybeZ	0.57458213	-0.799415	0.00892507	0.01614333	0	0.31576349	-1.6630837	2.14E-05	9.16E-05	-1	0.54955328	-0.8636687	0.02087111	0.16091186	0
miaB	0.47560423	-1.0721666	0.00391163	0.00765177	-1	0.86264477	-0.2131615	0.61351103	0.69231492	0	1.81378701	0.85900505	0.03962482	0.254731	0
ubiF	1.72153928	0.7836991	0.0105314	0.01873426	0	0.80607684	-0.3110107	0.64594754	0.72282658	0	0.46823029	-1.0947098	0.01041432	0.0960819	0
glnX	0.18264297	-2.4529018	1.34E-05	4.47E-05	-1	0.68749542	-0.540578	0.17021724	0.24528989	0	3.76414934	1.91232387	0.0140305	0.12014466	0
glnV	0.18974777	-2.3978451	6.04E-05	0.00017837	-1	0.82886071	-0.2707984	0.36777167	0.45909125	0	4.36822361	2.12704671	0.00755384	0.07640468	0
metU	0.23479581	-2.0905215	8.81E-06	3.06E-05	-1	1.2776352	0.35347596	0.77675457	0.82890086	0	5.44147366	2.44399742	1.77E-05	0.00066031	1
sgrS	2.01914709	1.01374601	0.47885907	0.54895419	0	6.19464583	2.6310218	0.19738218	0.27646017	0	3.06795173	1.61727579	0.44879407	0.88755478	0
glnW	0.27433295	-1.8660002	0.00691624	0.01280439	-1	1.66004782	0.7312248	0.51058286	0.59768627	0	6.05121552	2.59722497	0.0017405	0.02597927	1
glnU	0.25041772	-1.9975914	0.00076421	0.00176358	-1	1.48924786	0.57458389	0.67975456	0.75171057	0	5.94705467	2.57217534	0.00063276	0.01172458	1
leuW	0.27487051	-1.8631759	0.02147432	0.03551182	-1	1.56431114	0.64552749	0.75730298	0.81384287	0	5.69108384	2.50870343	0.01815	0.14447926	0
metT	0.21740448	-2.2015464	0.00654738	0.01217162	-1	1.34884815	0.43132794	0.93621659	0.95501873	0	6.20432536	2.63327434	0.01203246	0.10710485	0
asnB	0.29982159	-1.7378238	7.31E-07	3.00E-06	-1	0.33621833	-1.5725297	9.55E-05	0.000346	-1	1.12139466	0.16529411	0.80400937	0.96990832	0
umpH	0.23003837	-2.1200536	8.97E-10	5.49E-09	-1	0.28234953	-1.8244459	2.35E-05	9.94E-05	-1	1.22740185	0.29560767	0.32545113	0.82473426	0
nagC	0.43412517	-1.203817	0.00018477	0.00048724	-1	0.37359916	-1.4204369	0.00030817	0.00096718	-1	0.86057936	-0.2166199	0.53647126	0.9187668	0

nagA	0.35531157	-1.4928434	5.65E-06	2.02E-05	-1	0.21552541	-2.2140701	2.76E-07	1.85E-06	-1	0.60658146	-0.7212267	0.10634188	0.47951706	0
nagB	0.31787937	-1.6534487	6.34E-07	2.63E-06	-1	0.16490342	-2.6003068	4.05E-10	4.57E-09	-1	0.51876101	-0.9468581	0.0108389	0.09909497	0
nagE	0.17395553	-2.5232096	3.95E-14	3.70E-13	-1	0.19397558	-2.3660531	9.60E-09	8.55E-08	-1	1.11508717	0.1571565	0.45695532	0.88755478	0
glnS	0.33766693	-1.5663272	1.56E-06	6.14E-06	-1	0.39906082	-1.3253195	0.00061109	0.00176843	-1	1.18181787	0.24100772	0.55448559	0.92071004	0
chiP	0.72026525	-0.4733998	0.30247715	0.37019717	0	0.57852371	-0.789552	0.27242438	0.36034979	0	0.80320925	-0.3161522	0.79268671	0.96567635	0
chiQ	0.91580305	-0.1268907	0.8875313	0.91252928	0	1.59524876	0.67378141	0.60090811	0.68157416	0	1.74191247	0.80067213	0.58438944	0.92806272	0
fur	0.28686325	-1.8015649	1.50E-08	7.86E-08	-1	0.37424414	-1.4179484	2.19E-05	9.33E-05	-1	1.30460816	0.38361656	0.73182089	0.95562813	0
uof	0.34414254	-1.5389218	0.00021247	0.00055204	-1	0.60609323	-0.7223884	0.0513896	0.08795145	0	1.76116917	0.81653349	0.25582971	0.75662874	0
fldA	0.30062622	-1.7339572	6.39E-08	3.03E-07	-1	0.31016541	-1.6888903	4.52E-06	2.28E-05	-1	1.03173105	0.04506694	0.77153719	0.96223954	0
ybfE	0.62386195	-0.6807013	0.13297566	0.18005931	0	1.15030407	0.20201527	0.68865221	0.758619	0	1.84384392	0.88271654	0.09859602	0.45734597	0
ybfF	0.73169328	-0.4506891	0.28247706	0.34958201	0	0.64589538	-0.6306276	0.37581634	0.46702262	0	0.88274061	-0.1799385	1	1	0
seqA	0.19380422	-2.3673281	1.16E-12	9.65E-12	-1	0.25629564	-1.9641191	1.46E-07	1.04E-06	-1	1.32244617	0.403209	0.57261713	0.92806272	0
pgm	0.23596308	-2.0833669	4.27E-11	3.04E-10	-1	0.22551057	-2.1487331	3.98E-08	3.15E-07	-1	0.95570276	-0.0653661	0.92228201	1	0
talB	0.0961137	-3.3791141	1.84E-24	3.86E-23	-1	0.10086889	-3.3094468	2.81E-17	9.34E-16	-1	1.04947465	0.06966732	1	1	0
setA	2.23896624	1.16283278	0.00167626	0.00357683	1	2.77242581	1.47114886	0.00046463	0.00137891	1	1.23826155	0.30831608	0.38225858	0.86036984	0
ybfP	3.70218138	1.88837558	0.01491736	0.02561585	1	3.12492733	1.64382264	0.03849253	0.06889368	0	0.84407732	-0.2445529	0.95271563	1	0
ybfG	4.65065878	2.21743509	0.00565767	0.01066579	1	3.69748544	1.88654446	0.02852173	0.05275744	0	0.79504552	-0.3308906	0.87798325	0.98956956	0
ybfI	10.496555	3.39184401	0.00097844	0.0022005	1	5.57158514	2.47808784	0.00918864	0.01951205	1	0.53080131	-0.9137562	0.65656778	0.93664426	0
potE	4.29524636	2.10274088	1.01E-06	4.06E-06	1	4.5893236	2.19828154	6.24E-06	3.06E-05	1	1.06846574	0.09554065	0.71484739	0.9525362	0
speF	7.00378969	2.80813576	8.37E-12	6.39E-11	1	7.08132451	2.82401923	3.68E-10	4.17E-09	1	1.01107041	0.01588347	0.75287597	0.9573569	0
ybfK	4.30963891	2.10756699	0.27470137	0.34111778	0	0.99097436	-0.0130804	0.78816182	0.83844177	0	0.22994371	-2.1206474	0.50895834	0.90925684	0
kdpE	4.33608963	2.11639458	1.87E-07	8.29E-07	1	3.56747467	1.83490319	5.39E-05	0.0002079	1	0.82274006	-0.2814914	0.69882725	0.94869712	0
kdpD	2.51442118	1.33022633	2.72E-05	8.56E-05	1	1.90295443	0.92824101	0.01563116	0.03104453	0	0.7568161	-0.4019853	0.27894126	0.78147864	0
kdpC	4.09404447	2.03352677	2.79E-05	8.75E-05	1	3.52503705	1.81763842	0.00102261	0.00282846	1	0.86101582	-0.2158884	0.81023634	0.96990832	0
kdpB	4.32178833	2.11162841	6.38E-05	0.00018753	1	4.75979024	2.250898	8.98E-05	0.00032733	1	1.10134738	0.13926958	0.61561157	0.92897598	0
leuD	1.51683792	0.60106694	0.18315158	0.2391311	0	2.17160616	1.11876248	0.02509028	0.04710023	1	1.43166658	0.51769554	0.25421067	0.75391328	0
kdpA	9.55439736	3.25616488	2.81E-13	2.44E-12	1	9.59855039	3.26281654	8.80E-12	1.24E-10	1	1.00462123	0.00665166	0.69735079	0.94869712	0
kdpF	14.9638316	3.90340773	0.06508567	0.09553668	0	9.89949677	3.30735519	0.19863685	0.27768942	0	0.66156162	-0.5960525	0.82685338	0.97706951	0
ybfA	0.03253497	-4.9418648	5.10E-30	1.62E-28	-1	0.10182431	-3.2958461	1.16E-12	1.86E-11	-1	3.12968774	1.64601872	0.00158614	0.02437244	1
rhsC	2.05570057	1.03963014	0.02237951	0.03686506	1	2.45375155	1.29498918	0.02059639	0.03962362	1	1.19363277	0.25535904	0.65387861	0.93589534	0
ybfB	1.47968943	0.5652944	0.78610205	0.82730825	0	3.26437378	1.70680626	0.20630576	0.28659703	0	2.2061209	1.14151186	0.31856339	0.81858662	0
rhsO	3.99102834	1.99676052	0.00612961	0.01148685	1	6.30008677	2.6553717	0.0020812	0.00525022	1	1.57856227	0.65861117	0.42665698	0.87784805	0
ybfC	2.69826713	1.43203318	0.09283556	0.13054909	0	3.72209157	1.89611355	0.0733693	0.12034853	0	1.37943776	0.46408037	0.70069104	0.94869712	0
ybfQ	4.70412943	2.23392776	0.16894793	0.22289218	0	6.293533	2.65387013	0.13621711	0.20213009	0	1.33787412	0.41994238	0.74244844	0.95683847	0
ybfL	5.08391959	2.34594121	0.24137032	0.3047835	0	3.99799653	1.99927722	0.32776215	0.41741285	0	0.78640043	-0.346664	0.96427197	1	0
ybfD	0.35247277	-1.5044163	0.52533582	0.59284831	0	2.86817551	1.52013331	0.27595042	0.36411613	0	8.13729669	3.02454959	0.09780135	0.4568898	0
leuC	2.4628971	1.30035635	0.00452521	0.00869794	1	3.13863045	1.65013517	0.00118638	0.00322027	1	1.27436524	0.34977882	0.37440121	0.85857675	0
ybgA	4.68327699	2.22751837	0.00024652	0.00063159	1	1.30820029	0.38758344	0.65295415	0.72772211	0	0.27933436	-1.8399349	0.00812934	0.08038208	0
phr	4.81586307	2.26779437	8.43E-05	0.00024026	1	1.39333693	0.47854417	0.35162305	0.4424323	0	0.28932237	-1.7892502	0.011924	0.10697905	0
dtpD	4.31951078	2.11086792	4.16E-08	2.05E-07	1	4.50892392	2.17278317	1.19E-07	8.66E-07	1	1.0438506	0.06191524	0.59900342	0.92806272	0
ybgI	0.33192614	-1.5910659	2.88E-06	1.09E-05	-1	0.23673032	-2.0786836	6.59E-07	4.01E-06	-1	0.71320179	-0.4876178	0.20058541	0.67788667	0
ybgJ	0.44676008	-1.1624278	0.00130661	0.00285837	-1	0.33922488	-1.5596861	0.00075966	0.00215326	-1	0.75929989	-0.3972583	0.45798473	0.88755478	0
ybgK	0.49112028	-1.0258517	0.00406454	0.00791232	-1	0.43274346	-1.2084161	0.00891538	0.01900486	-1	0.8811354	-0.1825644	0.84693528	0.98159911	0
ybgL	0.45064976	-1.1499215	0.00362545	0.00714575	-1	0.29153769	-1.7782457	0.0002915	0.00092209	-1	0.64692742	-0.6283242	0.20378272	0.68295505	0
nei	0.80387172	-0.3149628	0.67119607	0.72485894	0	0.95239572	-0.070367	0.85989295	0.89399438	0	1.18476082	0.24459584	0.74767254	0.95683847	0
abrB	0.80093194	-0.3202484	0.21103207	0.27096763	0	0.80255028	-0.3173363	0.39832181	0.4892118	0	1.00202057	0.00291213	0.93952544	1	0
ybgO	33.311764	5.05795985	1.52E-13	1.35E-12	1	13.3624954	3.74011755	3.67E-06	1.88E-05	1	0.40113443	-1.3178423	0.03033856	0.2116979	0
leuB	2.56683141	1.35998854	0.01188399	0.02092966	1	3.5287457	1.81915546	0.00153822	0.00403166	1	1.37474775	0.45916692	0.29553766	0.79986628	0
ybgP	6.08942553	2.60630613	3.63E-05	0.00011165	1	4.69201558	2.2302078	0.0015275	0.00400832	1	0.77051859	-0.3760983	0.6611045	0.93664426	0
ybgQ	7.61702381	2.9292274	4.29E-13	3.70E-12	1	8.77329691	3.13311909	1.58E-12	2.48E-11	1	1.15180117	0.20389169	0.45457538	0.88755478	0
ybgD	18.6708524	4.22271589	1.67E-17	2.11E-16	1	13.2869065	3.73193335	5.05E-10	5.61E-09	1	0.71163899	-0.4907825	0.19913556	0.67679424	0
gltA	2.67455806	1.41930052	0.13646379	0.18433044	0	0.5734239	-0.8023261	0.45759132	0.54680801	0	0.21439949	-2.2216266	0.04916333	0.29394474	0
sdhC	1.85726658	0.89318091	0.20723247	0.26663146	0	1.05818644	0.08159383	0.94275713	0.96058192	0	0.56975474	-0.8115871	0.21557428	0.69799995	0
sdhD	2.3105674	1.20824717	0.00420965	0.00816091	1	1.25576865	0.3285707	0.86000682	0.89399438	0	0.5434893	-0.8796765	0.02771847	0.1990854	0
sdhA	1.27862142	0.35458917	0.32790371	0.39724374	0	0.36655574	-1.4478955	0.00022889	0.00074536	-1	0.28668043	-1.8024846	3.77E-06	0.00018258	-1
sdhB	0.72259379	-0.4687432	0.06797861	0.09912892	0	0.25202484	-1.9883621	7.09E-08	5.39E-07	-1	0.34877804	-1.5196189	2.13E-05	0.00077001	-1
sucA	0.95865989	-0.060909	0.56531181	0.63071035	0	0.36699722	-1.446159	0.00015835	0.00053911	-1	0.38282317	-1.3852499	0.00034964	0.00720529	-1
sucB	0.47269986	-1.0810037	0.00011794	0.00032377	-1	0.26705467	-1.904793	2.39E-07	1.62E-06	-1	0.56495611	-0.8237893	0.02019433	0.157067	0
leuA	2.22234748	1.15208441	0.02113551	0.03499083	1	2.78794314	1.47920114	0.00397819	0.00929646	1	1.2545037	0.32711673	0.36174846	0.85310733	0
sucC	0.54692394	-0.8705879	0.00166476	0.00355744	0	0.26433632	-1.9195534	1.83E-07	1.27E-06	-1	0.48331459	-1.0489656	0.0029811	0.03766955	-1
sucD	0.4349965	-1.2009243	3.14E-05	9.74E-05	-1	0.2219243	-2.1718604	7.89E-09	7.16E-08	-1	0.51017491	-0.9709361	0.00665635	0.06906944	0
mngR	0.62202983	-0.6849443	0.14287188	0.19216355	0	0.58976551	-0.7617866	0.25887946	0.34582477	0	0.94813059	-0.0768423	0.96181557	1	0
mngA	15.8709854	3.9883198	3.63E-16	4.06E-15	1	10.4011922	3.37867699	1.84E-11	2.47E-10	1	0.65535894	-0.6096428	0.42871124	0.87784805	0
mngB	2.03023665	1.0216479	0.01033984	0.01842318	1	2.26336118	1.17846682	0.002312	0.00579181	1	1.11482628	0.15681892	0.43880686	0.88087171	0
cydA	0.07548222	-3.7277194	2.39E-29	7.40E-28	-1	0.18878491	-2.4051846	9.58E-11	1.17E-09	-1	2.50105145	1.32253474	0.00022784	0.00510042	1
cydB	0.08573577	-3.5439589	7.47E-26	1.77E-24	-1	0.21164914	-2.2402535	1.47E-09	1.51E-08	-1	2.46862123	1.30370549	0.00062379	0.01168659	1
cydX	0.07140172	-3.8078973	2.89E-24	5.92E-23	-1	0.12351665	-3.0172226	1.30E-12	2.07E-11	-1	1.72988334	0.79067475	0.08829692	0.42649441	0
ybgE	0.19337595	-2.3705197	1.02E-11	7.74E-11	-1	0.36443908	-1.4562504	0.00016977	0.00057268	-1	1.88461436	0.91426934	0.04170695	0.26247184	0
ybgC	0.54799906	-0.8677547	0.15815984	0.21010287	0	1.88679682	0.91593907	0.40172108	0.49256533	0	3.44306577	1.78369374	0.03373446	0.22797856	0
leuL	1.64100189	0.7145769	0.6044878	0.66683881	0	0.51985309	-0.9438241	0.67133053	0.74407064	0	0.31679006	-1.658401	0.32310722	0.82325896	0
tolQ	0.54320352	-0.8804353	0.03224972	0.05127682	0	1.62142178	0.69725943	0.25475024	0.34094727	0	2.98492503	1.5776947	0.00188423	0.02737318	1
tolR	0.37426862	-1.417854	0.02755502	0.04451739	-1	1.14946779	0.20096604	0.85257712	0.88877997	0	3.0712374	1.61882003	0.08131013	0.40471519	0
tolA	0.69914432	-0.5163378	0.1869538	0.24380719	0	0.88142231	-0.1820947	0.60690327	0.68678759	0	1.26071583	0.33424312	0.52005154	0.91269012	0
tolB	0.31239691	-1.6785479	1.47E-07	6.63E-07	-1	0.39265851	-1.3486529	0.00017937	0.00059912	-1	1.25692186	0.32989496	0.51131826	0.90925684	0
pal	0.15499987	-2.6896611	8.33E-17	1.00E-15	-1	0.16766541	-2.576343	3.37E-12	5.05E-11	-1	1.08171324	0.11331809	0.84716278	0.9816048	0
ybgF	0.62648036	-0.6746588	0.06796184	0.09912892	0	0.58311878	-0.7781383	0.01758089	0.03437609	0	0.9307854	-0.1034795	0.34385067	0.84269271	0
lysT	0.86333905	-0.2120008	0.87691145	0.90409583	0	1.87342995	0.90568203	0.73546805	0.79716295	0	2.1699817	1.11768288	0.59378284	0.92806272	0
valT	0.74650232	-0.4217814	0.60871336	0.66999424	0	1.38761981	0.47261234	1	1	0	1.85882852	0.89439369	0.65324082	0.93532209	0
lysW	0.69207323	-0.5310034	0.40958777	0.48295493	0	1.32006256	0.4006063	1	1	0	1.90740299	0.93160969	0.49884198	0.90539057	0
valZ	0.93212635	-0.1014026	0.88798105	0.91277922	0	1.14511312	0.19549013	0.76544588	0.82000129	0	1.2284956	0.29689269	0.88872487	0.99411016	0
leuO	1.1450749	0.19544197	0.58744679	0.65064004	0	2.37293534	1.24667279	0.08906359	0.14177666	0	2.07229705	1.05123082	0.16532987	0.61372453	0
lysY	1.32833853	0.40962287	0.68309664	0.7353736	0	2.19422249	1.13370982	0.59813877	0.67895588	0	1.65185488	0.72408695	0.80871168	0.96990832	0
lysZ	1.41631976	0.50214702	0.55661236	0.62273475	0	3.08532116	1.62542067	0.39542782	0.4863333	0	2.17840719	1.12327365	0.63638752	0.93163024	0
lysQ	2.17355871	1.12005907	0.21195579	0.27199556	0	3.65721887	1.87074697	0.23112404	0.3157797	0	1.68259493	0.7506879	0.78525606	0.9635071	0
nadA	1.07715815	0.10723008	0.84968878	0.88048952	0	1.18760265	0.24805222	0.93291843	0.95253386	0	1.10253323	0.14082213	1	1	0
pnuC	0.6130956	-0.705816	0.09556608	0.13413262	0	0.38984991	-1.3590093	0.00513647	0.01165797	-1	0.63587132	-0.6531932	0.14686763	0.58035154	0
zitB	0.94115491	-0.0874959	0.66935638	0.72340277	0	0.48811076	-1.0347195	0.05208046	0.08896155	0	0.51862956	-0.9472236	0.07999243	0.40025955	0
ybgS	3.03671339	1.60251075	0.19070922	0.24782712	0	0.44323874	-1.1738441	0.34098634	0.43125891	0	0.14596002	-2.7763549	0.04378926	0.27110746	0
aroG	0.50167012	-0.9951891	0.00238417	0.00490722	-1	0.30126789	-1.7308812	1.84E-05	7.99E-05	-1	0.60052986	-0.7356921	0.04324644	0.26944204	0
gpmA	0.28084005	-1.8321794	1.12E-09	6.75E-09	-1	0.16427712	-2.6057965	2.98E-12	4.51E-11	-1	0.58494905	-0.7736171	0.01481731	0.12470296	0
galM	0.48023916	-1.058175	0.00094758	0.00213761	-1	0.35958137	-1.4756098	0.00027685	0.00087889	-1	0.74875479	-0.4174348	0.32682526	0.82690727	0
ilvI	3.02263478	1.59580667	0.00011361	0.00031284	1	2.6757093	1.41992138	0.00187569	0.00479946	1	0.88522415	-0.1758853	0.90338044	0.99847312	0
galK	0.28579531	-1.8069459	2.55E-08	1.28E-07	-1	0.19327575	-2.3712674	1.88E-08	1.57E-07	-1	0.67627336	-0.5643216	0.21890684	0.70102268	0
galT	0.31225013	-1.6792259	1.13E-06	4.55E-06	-1	0.24869785	-2.007534	2.17E-06	1.18E-05	-1	0.79646999	-0.3283081	0.40578073	0.86959974	0
galE	0.22577424	-2.1470472	1.18E-10	7.90E-10	-1	0.16811124	-2.5725119	2.11E-09	2.11E-08	-1	0.74459885	-0.4254647	0.37751416	0.85988523	0
modF	0.67721637	-0.5623113	0.19350846	0.25080014	0	0.80805953	-0.3074665	0.58073456	0.66363227	0	1.19320732	0.25484474	0.53430618	0.9187668	0
modE	0.59698986	-0.7442217	0.05897423	0.08779547	0	0.8198888	-0.2864998	0.37760135	0.46871359	0	1.3733714	0.45772182	0.44020221	0.88087171	0
acrZ	27.3252159	4.77216099	1.46E-13	1.30E-12	1	11.2875926	3.49666592	3.58E-05	0.00014501	1	0.41308338	-1.2754951	0.01400541	0.12014466	0
modA	0.42042167	-1.2500911	0.00037419	0.00091516	-1	0.52486757	-0.9299746	0.01480336	0.02954654	0	1.24843128	0.32011641	0.62494765	0.92989404	0
modB	1.18368284	0.24328258	0.65022669	0.70627405	0	1.28959869	0.36692218	0.31992701	0.41002247	0	1.08947992	0.12363961	0.58504102	0.92806272	0
modC	1.09415888	0.12982224	0.85010632	0.88048952	0	0.85487305	-0.2262179	0.95722419	0.97114015	0	0.78130614	-0.3560401	0.74225966	0.95683847	0
ybhA	0.73198615	-0.4501117	0.37362824	0.44435001	0	0.95241071	-0.0703443	1	1	0	1.30113214	0.37976749	0.38751591	0.86572704	0

ilvH	1.07176851	0.09999333	0.77328831	0.81693721	0	1.33440432	0.41619587	0.43898342	0.52886252	0	1.24504901	0.31620254	0.57296792	0.92806272	0
pgl	0.77147477	-0.3743091	0.16208563	0.21486561	0	0.38396785	-1.3809426	0.00117864	0.00320517	-1	0.4977063	-1.0066335	0.01995203	0.15545662	0
ybhD	3.53155615	1.82030403	0.01006163	0.01795644	1	3.11416545	1.63884559	0.02076143	0.03990637	1	0.88181111	-0.1814584	1	1	0
ybhH	8.89754967	3.15340808	3.85E-07	1.64E-06	1	8.04628465	3.00832278	3.76E-06	1.92E-05	1	0.9043259	-0.1450853	0.88354946	0.99247405	0
ybhI	6.92276369	2.7913481	0.00048365	0.00115475	1	7.97511659	2.99550561	0.00046348	0.00137642	1	1.15201341	0.20415751	0.62313551	0.92989404	0
ybhJ	4.00069432	2.0002504	5.91E-08	2.82E-07	1	4.88878765	2.28947674	1.26E-07	9.09E-07	1	1.2219848	0.28922634	0.44095543	0.88087171	0
ybhC	0.50723378	-0.9792773	0.00599745	0.01125829	0	0.81427601	-0.2964102	0.45270707	0.54214432	0	1.60532687	0.68286708	0.09746402	0.45619431	0
ybhB	0.82257422	-0.2817822	0.5176645	0.58568753	0	0.33227289	-1.5895595	0.0394831	0.07040993	0	0.40394274	-1.3077773	0.09000792	0.43286251	0
bioA	3.22432206	1.68899585	1.41E-05	4.66E-05	1	3.508041	1.81066561	1.56E-05	6.95E-05	1	1.08799336	0.12166975	0.54699332	0.91914725	0
bioB	1.59650579	0.67491778	0.06968191	0.10121177	0	1.61506953	0.69159627	0.0600636	0.10107428	0	1.01162773	0.01667849	0.71685016	0.95306277	0
bioF	1.29299162	0.37071293	0.30308136	0.3707312	0	2.10658868	1.07490865	0.02068389	0.03977464	1	1.62923614	0.70419572	0.12518334	0.52980664	0
cra	0.10980376	-3.1870007	4.26E-20	6.27E-19	-1	0.29079783	-1.7819116	9.16E-07	5.45E-06	-1	2.64834133	1.40508908	0.00099551	0.01662946	1
bioC	0.96024044	-0.0585324	0.921679	0.94150235	0	1.11531351	0.15719057	0.57574405	0.65929333	0	1.16128572	0.21572297	0.59840191	0.92806272	0
bioD	2.0922895	1.06508248	0.00993395	0.01775011	1	1.3372698	0.41929057	0.22004625	0.30301788	0	0.63914186	-0.6457919	0.36036941	0.85259072	0
uvrB	0.87772801	-0.1881541	0.4422954	0.51289545	0	0.67324775	-0.5707906	0.09302416	0.1472488	0	0.76703459	-0.3826364	0.25075531	0.7487007	0
ybhK	0.44540038	-1.1668253	0.00436996	0.00843994	-1	0.71191289	-0.4902274	0.30352389	0.39379684	0	1.59836615	0.67659794	0.15076052	0.58680838	0
moaA	0.17357001	-2.5264104	3.81E-14	3.57E-13	-1	0.21666273	-2.2064771	3.00E-09	2.92E-08	-1	1.24827286	0.31993333	0.74951121	0.95683847	0
moaB	0.22643305	-2.1428436	3.94E-11	2.81E-10	-1	0.14042101	-2.8321693	2.37E-11	3.15E-10	-1	0.62014363	-0.6893257	0.12217037	0.52159375	0
moaC	0.34460987	-1.5369641	3.45E-06	1.29E-05	-1	0.26506733	-1.9155692	1.65E-06	9.28E-06	-1	0.7691809	-0.3786052	0.25615813	0.75663587	0
moaD	0.36729077	-1.4450055	0.00029643	0.00074183	-1	0.28043634	-1.8342548	0.00040094	0.0012152	-1	0.76352679	-0.3892493	0.51650408	0.90937129	0
moaE	0.5380274	-0.8942485	0.00929985	0.01675263	0	0.54202328	-0.8835733	0.02740581	0.05090638	0	1.00742692	0.01067518	0.95985908	1	0
ybhL	0.33771798	-1.5661091	4.25E-07	1.80E-06	-1	0.17127424	-2.5456199	1.92E-11	2.56E-10	-1	0.50715166	-0.9795109	0.00223697	0.03112001	0
mog	0.22386255	-2.1593149	1.91E-09	1.11E-08	-1	0.28533542	-1.8092692	1.23E-05	5.67E-05	-1	1.27460094	0.35004563	0.50248196	0.90598897	0
mraZ	0.47520542	-1.0733768	0.0013565	0.00295448	-1	0.37067453	-1.4317751	0.00041245	0.0012441	-1	0.78003011	-0.3583983	0.31863496	0.81858662	0
ybhM	5.65877434	2.50048961	0.00010039	0.00027994	1	4.06410058	2.02293611	0.00369097	0.00870261	1	0.7181945	-0.4775535	0.56650125	0.92591251	0
ybhN	10.3892232	3.37701588	7.77E-13	6.57E-12	1	6.10535538	2.61007527	2.37E-08	1.96E-07	1	0.58766236	-0.7669406	0.33970395	0.83879867	0
clsB	7.56779608	2.91987321	2.86E-14	2.74E-13	1	3.41727557	1.77284659	1.56E-05	6.95E-05	1	0.45155492	-1.1470266	0.01775393	0.14235426	0
ybhP	5.39797777	2.43241904	1.26E-08	6.65E-08	1	2.08359162	1.05907254	0.01070534	0.02230405	1	0.38599485	-1.3733465	0.01386314	0.11917432	0
ybhQ	0.34325179	-1.5426609	1.34E-06	5.32E-06	-1	0.26610403	-1.9099377	3.50E-06	1.81E-05	-1	0.77524442	-0.3672769	0.37789595	0.85988523	0
ybhR	0.8394336	-0.2525119	0.54915795	0.6157986	0	0.8344449	-0.2611113	0.87884903	0.90963922	0	0.99405706	-0.0085994	0.73017695	0.9551867	0
ybhS	0.82532177	-0.2769714	0.64773758	0.70434685	0	0.77370649	-0.3701417	0.54388695	0.62917184	0	0.93746042	-0.0931703	0.88947573	0.99411016	0
ybhF	0.94029672	-0.088812	0.7298002	0.77862982	0	0.57119514	-0.8079444	0.05524587	0.09357283	0	0.60746265	-0.7191324	0.08303612	0.41006639	0
ybhG	0.50987907	-0.971773	0.00212572	0.00443311	0	0.33490219	-1.5781883	0.00026384	0.00084318	-1	0.65682671	-0.6064153	0.19161925	0.66643604	0
ybiH	0.27137731	-1.881628	1.50E-06	5.92E-06	-1	0.29000526	-1.785849	0.00032876	0.00102021	-1	1.06864226	0.09577897	0.67317286	0.93997784	0
rsmH	0.46276611	-1.1116449	0.00092615	0.00209356	-1	0.46683081	-1.0990283	0.00182147	0.00466885	-1	1.00878349	0.01261656	0.68360313	0.943596	0
rhlE	1.13773627	0.18616618	0.58419175	0.64803514	0	1.56066009	0.64215635	0.12005372	0.18162184	0	1.37172394	0.45599017	0.25585345	0.75662874	0
ybiA	0.27687157	-1.8527112	9.87E-05	0.00027589	-1	0.28471312	-1.8124191	0.00487178	0.0110971	-1	1.02832197	0.04029205	0.65655695	0.93664426	0
dinG	1.12508146	0.17002947	0.7640635	0.80932097	0	0.61454169	-0.7024172	0.07281974	0.11962226	0	0.54621973	-0.8724467	0.02785006	0.19970534	0
ybiB	16.2265257	4.02028223	4.49E-33	1.75E-31	1	2.37949703	1.25065666	0.04262124	0.07521897	0	0.14664242	-2.7696256	2.14E-15	1.57E-12	-1
ybiC	1.02509343	0.03575541	0.93491029	0.95237517	0	0.69709435	-0.5205742	0.15619322	0.22763276	0	0.68003006	-0.5563296	0.12218351	0.52159375	0
ybiJ	1.69755927	0.76329196	0.27479113	0.34113334	0	1.1413614	0.19075567	0.98545451	0.99673231	0	0.6724336	-0.5725363	0.35447898	0.84775072	0
ybiI	2.32258677	1.2157325	0.02743247	0.04435183	1	1.2379439	0.30794594	0.35658968	0.44791637	0	0.53300222	-0.9077866	0.29095278	0.79399862	0
ybiX	1.15457438	0.20736112	0.48498732	0.55439673	0	0.93098925	-0.1031636	0.70900589	0.77532715	0	0.80634844	-0.3105247	0.87359884	0.98834554	0
fiu	2.59933009	1.37813986	0.00078768	0.00180737	1	3.17599179	1.66720718	4.09E-05	0.00016311	1	1.22185012	0.28906733	0.25406604	0.75391328	0
mcbA	1.80854519	0.85482964	0.11653797	0.16015813	0	0.99216998	-0.0113408	0.89096565	0.91754305	0	0.54860115	-0.8661704	0.17183514	0.62627519	0
ftsL	0.57393176	-0.8010489	0.06790144	0.09908182	0	0.4948952	-1.014805	0.0155205	0.03085249	-1	0.86228928	-0.2137562	0.37018591	0.85484012	0
rlmF	0.73111382	-0.4518321	0.26476062	0.3311007	0	1.13611591	0.18411003	0.57400649	0.65798565	0	1.55395218	0.63594211	0.12149435	0.52119658	0
ybiO	4.65887367	2.21998121	8.52E-08	3.96E-07	1	2.90379185	1.53793804	0.00031796	0.00099158	1	0.62328195	-0.6820432	0.23325414	0.72389215	0
glnQ	0.35648539	-1.4880851	1.11E-05	3.77E-05	-1	1.92279374	0.94320401	0.05099132	0.0873832	0	5.39375188	2.43128916	3.97E-09	5.00E-07	1
glnP	0.5135162	-0.9615183	0.0043024	0.00832672	0	1.85444659	0.89098872	0.06354473	0.10616414	0	3.61127185	1.85250703	1.10E-05	0.00044768	1
glnH	0.09587858	-3.3826477	1.28E-22	2.27E-21	-1	0.46810599	-1.0950929	0.00120092	0.00325175	-1	4.88227934	2.28755484	5.71E-09	6.62E-07	1
dps	0.97387886	-0.0381858	0.81912383	0.85513353	0	0.12597969	-2.9887369	0.00041917	0.00126093	-1	0.12935869	-2.9505511	0.00045318	0.00888233	-1
rhtA	3.56325268	1.83319479	0.0001553	0.00041698	1	4.79037468	2.2601385	2.65E-05	0.00011083	1	1.34438254	0.42694371	0.32920106	0.83053586	0
ompX	0.62468885	-0.6787903	0.08184763	0.11673264	0	0.37743133	-1.4057139	0.00213011	0.00536352	-1	0.60419092	-0.7269236	0.06570262	0.35237234	0
opgE	2.42438022	1.27761598	0.00407157	0.00791904	1	2.06060313	1.04306667	0.01261277	0.02567288	1	0.84995048	-0.2345493	0.92872451	1	0
mntS	0.33565008	-1.5749701	0.00063355	0.00147829	-1	0.26703736	-1.9048865	7.93E-05	0.00029233	-1	0.79558258	-0.3299164	0.15776546	0.59957345	0
ftsI	0.44666785	-1.1627257	0.00056509	0.00133548	-1	0.43929461	-1.1867393	0.0014827	0.00391634	-1	0.98349278	-0.0240136	0.73706521	0.95683847	0
mntR	0.95289933	-0.0696043	1	1	0	1.23796569	0.30797133	0.66300995	0.73686525	0	1.29915685	0.37757562	0.60876523	0.92806272	0
ybiR	3.21176778	1.68336759	2.52E-06	9.59E-06	1	4.05756582	2.0206145	2.99E-06	1.58E-05	1	1.26334346	0.33724691	0.42102467	0.87375001	0
ldtB	0.76944939	-0.3781017	0.1746269	0.22956161	0	0.99371288	-0.009099	0.9892878	0.99969421	0	1.29145971	0.36900263	0.2962416	0.80050579	0
ybiT	0.53426005	-0.904386	0.00546689	0.01035474	0	0.7193781	-0.4751779	0.29619963	0.38622195	0	1.34649428	0.4292081	0.19638167	0.67373807	0
ybiU	2.48475762	1.31310513	0.04736888	0.07252466	0	2.99374864	1.5819531	0.00798742	0.01726831	1	1.20484534	0.26884797	0.38666443	0.86562571	0
ybiV	1.1728337	0.22999847	0.57189137	0.63660387	0	0.78279803	-0.353288	0.57590555	0.65930742	0	0.66744163	-0.5832864	0.30177089	0.80606277	0
ybiW	1.71594973	0.77900729	0.08320582	0.11851664	0	1.69599878	0.76213513	0.05589339	0.09448848	0	0.98837323	-0.0168722	0.68292296	0.94348666	0
ybiY	4.32369275	2.11226401	1.40E-05	4.65E-05	1	3.16432103	1.66189598	0.00050307	0.00147884	1	0.73185613	-0.450368	0.70522478	0.94896888	0
fsaA	4.0352954	2.01267429	6.26E-08	2.98E-07	1	1.39159327	0.47673761	0.29497267	0.38484919	0	0.34485536	-1.5359367	0.00040681	0.00804494	-1
moeB	0.34248181	-1.5459007	2.85E-05	8.91E-05	-1	0.34886582	-1.5192558	0.00041617	0.00125363	-1	1.01864043	0.02664489	0.9892615	1	0
murE	0.75080384	-0.4134921	0.23515576	0.2977144	0	0.60215135	-0.731802	0.09958616	0.156095	0	0.80200887	-0.3183099	0.49960481	0.90541644	0
moeA	0.32256081	-1.6323569	2.10E-06	8.11E-06	-1	0.40103445	-1.3182019	0.00134199	0.00358107	-1	1.24328322	0.31415498	0.39837124	0.86854717	0
iaaA	0.7300483	-0.4539362	0.15735774	0.20924615	0	0.49879252	-1.0034883	0.01674482	0.03293073	-1	0.68323223	-0.5495521	0.18443641	0.65399884	0
gsiA	0.51510484	-0.957062	0.01213543	0.02132457	0	0.44498176	-1.1681819	0.01057613	0.02206605	-1	0.86386639	-0.2111199	0.70021434	0.94869712	0
gsiB	0.46301707	-1.1108627	0.00150819	0.00325754	-1	0.55648121	-0.8455951	0.02715473	0.05052495	0	1.20185894	0.26526758	0.6409447	0.93163024	0
gsiC	0.88630446	-0.1741257	0.78074191	0.82284348	0	1.01818888	0.02600521	0.58261933	0.66475466	0	1.14880261	0.20013093	0.41546513	0.87131399	0
gsiD	1.62076695	0.69667666	0.10297839	0.14337685	0	2.02896322	1.02074271	0.01965553	0.0380288	1	1.25185377	0.32406605	0.33828221	0.83714543	0
yliE	4.7867508	2.2590467	0.00144536	0.00313256	1	3.84431722	1.94272739	0.010252	0.0215219	1	0.80311622	-0.3163193	0.81393238	0.9712234	0
yliF	4.25922555	2.09059113	0.0010224	0.00229119	1	3.88315737	1.95723018	0.00105996	0.00292018	1	0.91170503	-0.133361	0.75798967	0.95806023	0
rimO	1.81371372	0.85894676	0.00920855	0.01660849	0	2.34349962	1.22866456	0.00270195	0.00659301	1	1.29210007	0.36971781	0.35377312	0.84775072	0
bssR	0.07789123	-3.6823953	3.32E-28	9.21E-27	-1	0.06930792	-3.850836	6.30E-22	4.42E-20	-1	0.8898039	-0.1684407	0.26259118	0.76135903	0
murF	0.47456808	-1.075313	0.00055388	0.0013111	-1	0.36406498	-1.4577321	0.00034757	0.00107256	-1	0.76715016	-0.3824191	0.39398243	0.86854717	0
yliI	3.84921981	1.94456606	1.86E-07	8.25E-07	1	2.08050809	1.0569359	0.02533035	0.04747689	1	0.54050124	-0.8876302	0.02835701	0.20268136	0
gstB	0.94068851	-0.088211	0.53976267	0.60711274	0	0.51473303	-0.9581037	0.0156795	0.03112655	0	0.54718754	-0.8698927	0.03365718	0.22797859	0
dacC	0.75502487	-0.4054039	0.20109777	0.26002602	0	0.3247806	-1.6224626	0.00017897	0.00059839	-1	0.43015881	-1.2170587	0.00414115	0.04777601	-1
deoR	0.967623	-0.047483	0.80249896	0.84070042	0	1.01904503	0.0272178	0.92082153	0.94148781	0	1.05314262	0.07470083	0.79433481	0.96579786	0
ybjG	1.79836825	0.84668847	0.07837878	0.11222012	0	2.00090958	1.00065597	0.10989667	0.16879854	0	1.11262506	0.1539675	0.87440354	0.98855575	0
mdfA	2.73809592	1.45317299	0.000567	0.00133856	1	2.64920775	1.40556099	0.00106633	0.00293406	1	0.9675365	-0.047612	0.82615237	0.97697604	0
ybjH	1.64033853	0.71399358	0.31067647	0.3789721	0	0.94964697	-0.0745368	0.83846475	0.87821184	0	0.57893353	-0.7885304	0.4160259	0.87131399	0
ybjI	0.70425973	-0.5058205	0.13688357	0.18478445	0	0.68409574	-0.5477299	0.26440256	0.35129131	0	0.97136852	-0.0419094	0.97308672	1	0
ybjJ	2.36341551	1.24087329	0.00058071	0.001368	1	2.90547786	1.53877546	0.00018143	0.00060555	1	1.2293555	0.29790217	0.378229	0.85988523	0
rcdA	0.42121969	-1.2473552	0.00533764	0.0101186	-1	0.44879193	-1.1558814	0.08577734	0.13758623	0	1.06545809	0.09147385	0.50380772	0.90701715	0
mraY	0.89183336	-0.1651539	0.78606695	0.82730825	0	0.7756289	-0.3665615	0.43122038	0.52221509	0	0.86970161	-0.2014076	0.60777994	0.92806272	0
rybB	0.62001503	-0.6896249	0.32366278	0.39311269	0	0.3262127	-1.6161152	0.21184184	0.29336543	0	0.52613676	-0.9264902	0.56342614	0.92432422	0
ybjL	0.60418876	-0.7269287	0.04148668	0.06447806	0	0.89680191	-0.1571387	0.79152247	0.84110822	0	1.48430749	0.56978999	0.13863399	0.56089533	0
ybjM	1.09213511	0.12715134	0.9902452	0.9965336	0	0.81982461	-0.2866128	0.83314543	0.87367576	0	0.75066226	-0.4137641	0.7559022	0.9573569	0
grxA	0.35080018	-1.5112786	0.00564495	0.0106509	-1	0.26591026	-1.9109887	0.00302323	0.00726859	-1	0.75801061	-0.39971	0.43204523	0.88005519	0
ybjC	0.2299443	-2.1206437	1.55E-05	5.07E-05	-1	0.45379367	-1.1398916	0.03624096	0.06536685	0	1.9734939	0.98075206	0.09542971	0.45064109	0
nfsA	0.3163199	-1.6605438	1.18E-05	3.97E-05	-1	0.33451229	-1.5798689	0.00068511	0.00195827	-1	1.05751265	0.08067492	0.71609792	0.95281877	0
rimK	0.87189154	-0.1977794	0.59298486	0.65562306	0	0.82405265	-0.2791916	0.57762734	0.66093609	0	0.94513206	-0.0814122	0.90567321	0.99890653	0
ybjN	0.08861737	-3.4962667	5.49E-18	7.31E-17	-1	0.2621656	-1.9314497	2.91E-06	1.54E-05	-1	2.95839972	1.56481699	0.00221048	0.0308488	1
potF	12.9903518	3.6993686	1.32E-20	2.00E-19	1	5.31437066	2.40989885	6.05E-08	4.65E-07	1	0.40910136	-1.2894697	0.00353316	0.04277461	-1
potG	11.5500302	3.52982472	8.79E-08	4.08E-07	1	7.5609317	2.91856402	3.55E-05	0.00014377	1	0.65462441	-0.6112607	0.51322663	0.90937129	0
murD	0.75223476	-0.4107451	0.2429837	0.30664543	0	0.58323289	-0.777856	0.03862602	0.0691046	0	0.77533361	-0.3671109	0.26357727	0.762024	0
potH	11.6416431	3.54122279	1.13E-09	6.81E-09	1	6.38642041	2.67500753	5.99E-06	2.96E-05	1	0.54858411	-0.8662153	0.2881318	0.79070395	0

potI	5.81376115	2.5394718	9.85E-06	3.38E-05	1	5.18547469	2.37447606	0.0001816	0.00060565	1	0.89193115	-0.1649957	0.97022356	1	0
ybjO	2.10250718	1.07211073	0.07459531	0.10757019	0	2.38768408	1.25561196	0.02534578	0.04747902	1	1.13563659	0.18350124	0.53613947	0.9187668	0
rlmC	2.6125168	1.38544032	0.00021365	0.00055447	1	3.51685315	1.81428509	0.00013823	0.00047985	1	1.34615523	0.42884478	0.40876725	0.86959974	0
artJ	1.7278568	0.78898366	0.10106042	0.14112674	0	0.66894584	-0.5800387	0.38320101	0.47434775	0	0.38715352	-1.3690223	0.01430484	0.12154983	0
artM	1.19274386	0.25428426	0.45516536	0.52547449	0	0.68644473	-0.5427845	0.22422862	0.30822451	0	0.5755173	-0.7970688	0.05813887	0.3249587	0
artQ	1.55713037	0.63888974	0.13066585	0.17753495	0	0.63934126	-0.6453419	0.27684852	0.36486538	0	0.41058942	-1.2842316	0.01232298	0.10870076	0
artI	0.5072631	-0.9791939	0.00326275	0.00650636	0	0.21992293	-2.1849301	6.48E-07	3.95E-06	-1	0.43354805	-1.2057362	0.00388017	0.04537455	-1
artP	1.12304352	0.16741384	0.63108792	0.6898083	0	0.85021441	-0.2341014	0.40893553	0.4996091	0	0.75706275	-0.4015152	0.2090544	0.69006731	0
ybjP	0.6971662	-0.5204255	0.22391923	0.28544048	0	0.14025412	-2.8338849	0.00010508	0.0003763	-1	0.20117746	-2.3134594	0.0013291	0.0210084	-1
ftsW	0.98333626	-0.0242433	0.92420498	0.94321012	0	0.74766337	-0.4195392	0.31218654	0.40255393	0	0.76033337	-0.395296	0.32559237	0.82473426	0
ybjQ	0.38321658	-1.3837681	5.35E-05	0.00016	-1	0.1265418	-2.9823141	4.01E-11	5.16E-10	-1	0.33020961	-1.598546	0.00017733	0.00420433	-1
amiD	0.65397126	-0.6127009	0.05302564	0.08010063	0	0.23269603	-2.1034815	1.23E-06	7.14E-06	-1	0.35581996	-1.4907806	0.00045878	0.00895228	-1
ybjS	1.27098768	0.34595005	0.39745936	0.46953143	0	1.46488884	0.5507912	0.10682616	0.16494229	0	1.15255944	0.20484115	0.39827137	0.86854717	0
ybjT	1.33112896	0.41265035	0.2306948	0.29309933	0	1.02979603	0.04235861	0.7990243	0.84624972	0	0.77362604	-0.3702917	0.42977777	0.87797234	0
ltaE	1.01454595	0.02083421	0.73198143	0.78076841	0	0.41105575	-1.282594	0.00077111	0.00218013	-1	0.40516228	-1.3034282	0.00083055	0.01453465	-1
poxB	5.36937221	2.42475342	0.00010805	0.00029923	1	1.21104103	0.27624774	0.6499985	0.72552245	0	0.22554611	-2.1485057	0.00386755	0.04536137	-1
hcr	5.85263542	2.54908641	1.53E-14	1.50E-13	1	12.7540291	3.67288117	1.91E-21	1.19E-19	1	2.17919418	1.12379476	0.00166788	0.02529357	1
hcp	17.0307191	4.09006745	1.57E-23	2.99E-22	1	23.7104368	4.56745033	2.00E-23	1.92E-21	1	1.39221583	0.47738289	0.23802146	0.73097121	0
ybjE	3.4750042	1.79701472	9.56E-05	0.00026783	1	2.94950432	1.56047252	0.00669461	0.01476221	1	0.84877719	-0.2365422	0.55623798	0.92183747	0
aqpZ	3.73517671	1.9011765	0.00021093	0.00054916	1	2.51524545	1.33069919	0.00747309	0.01628383	1	0.67339396	-0.5704773	0.51631415	0.90937129	0
murG	0.79529289	-0.3304418	0.27113678	0.33751472	0	0.58766136	-0.7669431	0.10021518	0.15689393	0	0.73892444	-0.4365012	0.41180554	0.8712535	0
ybjD	0.21369458	-2.2263777	7.91E-11	5.43E-10	-1	0.17106859	-2.5473532	8.64E-09	7.81E-08	-1	0.80052843	-0.3209755	0.65201351	0.9347567	0
ybjX	0.48354621	-1.0482743	0.00166793	0.00356077	-1	0.39798539	-1.3292126	0.00066507	0.00190963	-1	0.82305555	-0.2809383	0.35311548	0.84775072	0
macA	1.19221208	0.2536409	0.63103684	0.6898083	0	0.88014257	-0.1841909	0.59352813	0.67476224	0	0.73824329	-0.4378317	0.29972715	0.8048689	0
macB	1.99858293	0.99897744	0.00126463	0.0027749	0	1.9397621	0.95587973	0.00801715	0.01730717	0	0.97056874	-0.0430977	1	1	0
cspD	0.10551821	-3.2444361	6.66E-24	1.32E-22	-1	0.0741972	-3.7524914	2.21E-23	2.08E-21	-1	0.70316964	-0.5080553	0.01060863	0.09767028	0
clpS	0.18090279	-2.4667134	2.79E-13	2.44E-12	-1	0.15276321	-2.710631	3.97E-11	5.13E-10	-1	0.84444916	-0.2439175	0.36988806	0.85484012	0
clpA	0.35760558	-1.4835588	8.24E-07	3.36E-06	-1	0.14864354	-2.7500714	3.47E-13	6.04E-12	-1	0.41566336	-1.2665125	0.00017932	0.00422881	-1
serW	2.10448072	1.07346429	0.33102129	0.4005811	0	1.20634677	0.27064467	0.88443501	0.91294518	0	0.57322776	-0.8028196	0.55832685	0.92321763	0
infA	0.23513092	-2.0884638	0.01185846	0.02089302	-1	0.95416802	-0.0676848	0.58843269	0.67003696	0	4.05802874	2.02077908	0.09917826	0.458344	0
aat	1.41446912	0.50026068	0.29403345	0.36196927	0	1.37724851	0.4617889	0.34419246	0.43469177	0	0.97368581	-0.0384718	1	1	0
satP	0.37102416	-1.430415	0.00096221	0.0021662	-1	0.77139032	-0.374467	0.33152411	0.42117753	0	2.07908382	1.05594792	0.06094434	0.33554689	0
murC	0.52963018	-0.9169428	0.00843251	0.01531512	0	0.49553272	-1.0129478	0.00652826	0.01444586	-1	0.93562025	-0.096005	0.61235283	0.92897598	0
cydC	0.47781222	-1.0654844	0.00165935	0.00354761	-1	0.68348841	-0.5490112	0.23324047	0.31797928	0	1.43045402	0.51647312	0.13093434	0.53993058	0
cydD	1.02925533	0.04160092	0.93573118	0.95277192	0	1.80023617	0.84818619	0.07685981	0.12519112	0	1.74906664	0.80658526	0.07287291	0.37719428	0
trxB	0.47550581	-1.0724651	0.00042872	0.00103308	-1	0.44923425	-1.1544602	0.00137925	0.00366722	-1	0.94475029	-0.081995	0.68555138	0.943596	0
lrp	0.1523003	-2.7150093	7.84E-16	8.61E-15	-1	0.24375443	-2.0364997	1.60E-08	1.37E-07	-1	1.60048549	0.67850959	0.25049689	0.7487007	0
ftsK	0.531368	-0.9122168	0.00439509	0.00848475	0	0.63488791	-0.6554262	0.05150552	0.08811572	0	1.19481775	0.25679058	0.63947807	0.93163024	0
lolA	0.54473188	-0.8763818	0.00472854	0.00904154	0	0.38357289	-1.3824273	0.00025424	0.00081769	-1	0.70414988	-0.5060456	0.12813528	0.53612579	0
rarA	0.85744202	-0.221889	0.34300966	0.41248943	0	1.0868812	0.12019425	0.82855684	0.86968948	0	1.26758564	0.34208323	0.34752545	0.84560166	0
serS	0.14128057	-2.823365	1.73E-17	2.19E-16	-1	0.20014046	-2.3209153	1.16E-09	1.21E-08	-1	1.416617	0.50244976	0.2138525	0.69446947	0
dmsA	0.04177415	-4.5812457	3.30E-37	1.64E-35	-1	0.05902003	-4.0826516	2.37E-22	1.78E-20	-1	1.41283604	0.49859405	0.19987232	0.67698688	0
dmsB	0.02184643	-5.5164586	3.43E-42	2.53E-40	-1	0.06250549	-3.9998733	1.87E-19	8.52E-18	-1	2.86113052	1.51658531	0.00038985	0.00785034	1
ddlB	0.41495965	-1.268957	0.00017821	0.00047135	-1	0.31734514	-1.6558753	1.36E-05	6.16E-05	-1	0.76476144	-0.3869183	0.17818502	0.64146607	0
dmsC	0.08379484	-3.5769947	2.23E-24	4.63E-23	-1	0.1372364	-2.8652649	6.88E-12	9.84E-11	-1	1.63776671	0.71172987	0.03577278	0.23687379	0
ycaC	1.08490831	0.11757312	1	1	0	0.5790839	-0.7881557	0.09492508	0.1499728	0	0.53376298	-0.9057288	0.07152291	0.37176435	0
ycaD	1.81646561	0.86113405	0.01383475	0.02400161	0	1.69547889	0.76169282	0.03580901	0.06464052	0	0.93339443	-0.0994412	1	1	0
ycaM	1.42480721	0.51076672	0.12031705	0.16463458	0	1.77120786	0.82473353	0.04533037	0.07917586	0	1.24312107	0.31396681	0.43513854	0.88087171	0
ycaN	1.42854614	0.51454763	0.3033884	0.37090135	0	1.96581146	0.97512496	0.18062356	0.2568985	0	1.37609238	0.46057733	0.59033349	0.92806272	0
ycaK	3.7442896	1.90469202	0.00124569	0.00273983	1	5.58950142	2.4827196	0.00045941	0.00136802	1	1.49280692	0.57802758	0.40679876	0.86959974	0
pflA	0.29560965	-1.7582347	9.26E-08	4.29E-07	-1	0.70146362	-0.5115598	0.07991305	0.12935377	0	2.37293883	1.24667491	0.00386062	0.04536137	1
pflB	0.04793116	-4.3828924	1.16E-37	6.15E-36	-1	0.16959071	-2.5598709	3.43E-12	5.10E-11	-1	3.53821437	1.82302146	6.23E-07	4.16E-05	1
focA	0.11635599	-3.1033826	8.44E-22	1.44E-20	-1	0.33421609	-1.5811469	1.83E-06	1.02E-05	-1	2.8723583	1.52223572	0.00017252	0.00415739	1
ycaO	0.35245562	-1.5044865	0.00010721	0.00029743	-1	0.74093487	-0.4325814	0.25498426	0.34094727	0	2.10220761	1.07190515	0.02208355	0.16791112	0
ftsQ	0.57805142	-0.7907303	0.02945008	0.04718208	0	0.52749871	-0.9227605	0.02261071	0.04291956	0	0.91254635	-0.1320303	0.65891977	0.93664426	0
ycaP	1.39422082	0.47945908	0.34965485	0.41968083	0	1.29565921	0.37368631	0.48414019	0.57264868	0	0.92930703	-0.1057728	0.89019022	0.99411016	0
serC	0.65730705	-0.6053606	0.07759881	0.11128375	0	0.56458964	-0.8247254	0.04142705	0.07336213	0	0.85894354	-0.2193648	0.51435858	0.90937129	0
aroA	1.54265412	0.62541463	0.13074185	0.17753495	0	1.35036394	0.43334828	0.40413157	0.49497156	0	0.87535107	-0.1920663	0.61740785	0.92897598	0
ycaL	2.10599302	1.07450065	0.05110056	0.07761732	0	1.73222982	0.79263035	0.29686221	0.38649735	0	0.82252401	-0.2818703	0.58104253	0.92806272	0
cmk	0.53077497	-0.9138278	0.01216192	0.02135356	0	0.63945912	-0.645076	0.10285167	0.16043118	0	1.20476503	0.2687518	0.61891501	0.92897598	0
rpsA	0.14506693	-2.7852094	3.44E-18	4.68E-17	-1	0.24301194	-2.0409009	3.52E-08	2.81E-07	-1	1.67517119	0.74430853	0.04063026	0.25948079	0
ihfB	0.58358702	-0.7769803	0.04296912	0.0665479	0	0.35079668	-1.511293	0.00186996	0.00478758	-1	0.60110433	-0.7343127	0.08576502	0.4179268	0
ycaI	2.74387802	1.45621635	1.78E-05	5.78E-05	1	2.33007963	1.22037926	0.0007959	0.00223876	1	0.84919213	-0.2358371	0.78834807	0.96411212	0
msbA	0.47031875	-1.0882893	0.00331938	0.00659843	-1	0.79508147	-0.3308254	0.32384006	0.41407674	0	1.69051623	0.75746386	0.10558889	0.47807701	0
lpxK	1.64238265	0.71579029	0.04456707	0.06873351	0	1.92671549	0.94614355	0.02137948	0.04098738	0	1.17312217	0.23035326	0.51202425	0.90925684	0
ftsA	0.40782712	-1.2939704	2.01E-05	6.47E-05	-1	0.29011091	-1.7853236	3.02E-06	1.59E-05	-1	0.71135757	-0.4913532	0.16433017	0.61352961	0
ycaQ	1.59150524	0.6703919	0.11413083	0.15714335	0	2.27769367	1.18757373	0.01601709	0.03162624	1	1.43115688	0.51718183	0.26368449	0.762024	0
ycaR	0.19051151	-2.3920499	4.21E-08	2.07E-07	-1	0.23669541	-2.0788964	1.49E-05	6.67E-05	-1	1.24242051	0.31315355	0.85908517	0.98424873	0
kdsB	0.27203307	-1.878146	5.81E-08	2.78E-07	-1	0.27263634	-1.8749502	5.36E-06	2.67E-05	-1	1.00221762	0.00319581	0.91666328	1	0
ycbJ	0.02336408	-5.4195641	3.11E-47	2.70E-45	-1	0.03217333	-4.957991	3.78E-28	6.18E-26	-1	1.37704256	0.46157315	0.09450273	0.44860823	0
elyC	0.20165795	-2.3100178	1.10E-10	7.42E-10	-1	0.25943763	-1.9465404	3.97E-06	2.02E-05	-1	1.28652315	0.36347742	0.41434376	0.87131399	0
smtA	0.4704265	-1.0879588	0.00357759	0.00706401	-1	0.7845201	-0.3501177	0.28660593	0.37616115	0	1.66767838	0.73784108	0.13883254	0.56118378	0
mukF	0.37703515	-1.4072291	6.34E-05	0.0001867	-1	0.71470561	-0.484579	0.11198927	0.17119115	0	1.89559411	0.92265009	0.06376911	0.34521019	0
mukE	0.24357791	-2.0375448	2.35E-07	1.03E-06	-1	0.32537901	-1.6198069	0.00026259	0.00084147	-1	1.33583138	0.41773792	0.4355447	0.88087171	0
mukB	0.21912667	-2.190163	1.10E-11	8.27E-11	-1	0.26064128	-1.9398625	2.19E-07	1.51E-06	-1	1.18945481	0.25030046	0.60919442	0.92806272	0
ldtD	0.96918906	-0.04515	0.76518731	0.81031703	0	0.33221361	-1.5898169	0.00072093	0.00205148	-1	0.34277482	-1.5446669	0.00092266	0.0155898	-1
ftsZ	0.28506095	-1.8106577	6.22E-09	3.42E-08	-1	0.20662428	-2.2749183	3.86E-09	3.66E-08	-1	0.72484245	-0.4642607	0.19527842	0.67373807	0
ycbK	0.92200031	-0.1171609	0.69674349	0.74860521	0	1.51681569	0.60104579	0.47694624	0.56641371	0	1.64513577	0.71820665	0.27835009	0.7803712	0
ycbL	0.88709375	-0.1728415	0.42972805	0.50148185	0	1.18752759	0.24796103	0.73931856	0.79881412	0	1.33867203	0.42080255	0.33429518	0.83431903	0
aspC	0.26284495	-1.9277161	8.72E-10	5.37E-09	-1	0.26934119	-1.8924932	5.32E-07	3.30E-06	-1	1.02471514	0.03522291	0.99135346	1	0
ompF	0.00156919	-9.3157677	7.50E-103	1.66E-99	-1	0.00756433	-7.0465717	6.01E-51	2.66E-47	-1	4.82054393	2.26919594	3.57E-09	4.63E-07	1
asnS	0.25553591	-1.968402	7.00E-10	4.36E-09	-1	0.48238173	-1.0517528	0.00543022	0.01226169	-1	1.88772578	0.91664921	0.01002892	0.09370238	0
pncB	0.57080792	-0.8089227	0.01973105	0.03283799	0	0.98347874	-0.0240342	0.71521998	0.78096296	0	1.72295917	0.78488852	0.09688672	0.45510989	0
pepN	0.32270617	-1.6317069	1.73E-07	7.74E-07	-1	0.1928382	-2.3745372	4.90E-10	5.45E-09	-1	0.59756591	-0.7428303	0.02563548	0.18779478	0
ssuB	1.82818758	0.87041411	0.02901682	0.0465555	0	2.21840419	1.14952225	0.01217884	0.02490436	1	1.21344451	0.27910814	0.45494004	0.88755478	0
ssuC	7.16225443	2.84041377	1.12E-05	3.79E-05	1	8.49466122	3.08655641	9.94E-05	0.00035797	1	1.18603176	0.24614264	0.80352665	0.96977549	0
ssuD	6.36019685	2.66907142	0.00026514	0.00067229	1	7.10889428	2.82962518	0.00017722	0.00059453	1	1.11771608	0.16055376	0.57657523	0.92806272	0
lpxC	0.37673783	-1.4083672	2.84E-06	1.07E-05	-1	0.35133799	-1.5090685	3.36E-06	1.74E-05	-1	0.93257953	-0.1007013	0.34276464	0.84269271	0
ssuA	5.70071823	2.5111437	7.80E-05	0.00022418	1	6.85474149	2.77710226	8.39E-05	0.00030754	1	1.20243471	0.26595856	0.56612297	0.92590304	0
ssuE	4.77463986	2.25539192	0.00013132	0.0003565	1	4.98498251	2.31758844	0.00137028	0.00364775	1	1.04405414	0.06219653	1	1	0
elfA	8.10111398	3.01812031	4.44E-11	3.13E-10	1	2.38385632	1.25329729	0.03130682	0.05745218	0	0.29426278	-1.764823	0.00039252	0.00786827	-1
elfD	26.6782676	4.73759308	1.74E-23	3.31E-22	1	9.6450103	3.26978278	1.94E-09	1.94E-08	1	0.36153061	-1.4678103	0.00164479	0.02509868	-1
elfC	9.53658724	3.25347308	1.87E-16	2.15E-15	1	7.16428826	2.84082339	2.37E-10	2.75E-09	1	0.75124236	-0.4126497	0.34974097	0.84775072	0
elfG	5.08701679	2.34681985	1.37E-08	7.19E-08	1	4.19383198	2.06826906	1.00E-05	4.70E-05	1	0.82441874	-0.2785508	0.68216557	0.94348666	0
ycbU	5.23900171	2.38929193	0.00025742	0.00065458	1	3.64270544	1.86501034	0.0053388	0.01207378	1	0.69530526	-0.5242816	0.65146496	0.9347567	0
ycbV	3.94332234	1.97941165	0.01885258	0.03151849	1	2.11004886	1.07727641	0.1126585	0.17190536	0	0.53509419	-0.9021352	0.65799458	0.93664426	0
ycbF	1.61199392	0.6888463	0.13357314	0.18081292	0	1.73215877	0.79257117	0.07831439	0.12723209	0	1.07454424	0.10372488	0.63874415	0.93163024	0
pyrD	1.27938543	0.35545096	0.40981994	0.48309973	0	1.6540658	0.72601663	0.20443254	0.28426287	0	1.29285965	0.37056567	0.53930142	0.91897962	0
secM	0.32448729	-1.6237661	3.02E-06	1.14E-05	-1	0.43941743	-1.186336	0.00177206	0.00455805	-1	1.35418996	0.43743013	0.43638045	0.88087171	0
zapC	0.72865856	-0.4566852	0.3664665	0.43689137	0	0.63458534	-0.6561139	0.27244369	0.36034979	0	0.87089534	-0.1994287	0.65942727	0.93664426	0
ycbX	0.43634682	-1.1964528	0.00234557	0.00483677	-1	0.6234433	-0.6816697	0.17938858	0.25571553	0	1.4287793	0.51478309	0.1898466	0.66370038	0
rlmL	0.80530847	-0.3123866	0.28839278	0.35564685	0	1.00957269	0.0137448	0.81546826	0.85901174	0	1.25364718	0.32613138	0.27260597	0.77560795	0

uup	0.38319023	-1.3838673	3.70E-05	0.00011387	-1	0.58705215	-0.7684394	0.05532296	0.09366749	0	1.53201229	0.61542788	0.11010094	0.49166233	0
pqiA	0.94961714	-0.0745821	0.74752044	0.79503919	0	1.06027841	0.08444314	0.90574019	0.92821566	0	1.11653251	0.15902525	0.72202058	0.95399541	0
pqiB	0.59675221	-0.7447961	0.01747518	0.02953994	0	0.48100379	-1.0558798	0.01591731	0.03152783	-1	0.80603604	-0.3110837	0.56900251	0.92799596	0
pqiC	0.72624001	-0.4614817	0.22873275	0.29098864	0	0.45514599	-1.1355987	0.01330867	0.02687889	-1	0.62671566	-0.6741171	0.11605359	0.50522834	0
rmf	3.29422015	1.71993697	0.02897617	0.04650717	1	1.11323428	0.15475724	0.6081683	0.68769082	0	0.3379356	-1.5651797	0.02097986	0.16146806	0
fabA	0.25749894	-1.9573616	9.34E-10	5.68E-09	-1	0.23173438	-2.109456	4.93E-07	3.09E-06	-1	0.89994303	-0.1520944	1	1	0
ycbZ	0.43580015	-1.1982614	0.00025162	0.00064205	-1	0.74773735	-0.4193965	0.10443584	0.16227215	0	1.7157804	0.77886492	0.12951831	0.53863917	0
secA	0.48816047	-1.0345726	0.00039091	0.00095342	-1	0.48663498	-1.0390881	0.00222939	0.00559436	-1	0.99687503	-0.0045154	0.82962738	0.97877388	0
matP	0.26372084	-1.9229165	6.95E-07	2.87E-06	-1	0.27418811	-1.8667621	2.62E-06	1.41E-05	-1	1.03969071	0.05615441	0.50094046	0.90598897	0
ompA	0.20213859	-2.3065833	8.20E-14	7.50E-13	-1	0.12191774	-3.0360201	2.57E-15	6.38E-14	-1	0.60313934	-0.7294368	0.02490395	0.18403694	0
sulA	0.60151771	-0.7333209	0.01867656	0.03124786	0	0.89303603	-0.1632097	0.39255484	0.48333793	0	1.48463796	0.57011117	0.27337631	0.77568242	0
sxy	0.61509	-0.7011306	0.11748589	0.1612328	0	0.56635472	-0.8202222	0.05554066	0.09392812	0	0.92076723	-0.1190916	0.55559553	0.92146532	0
yccS	1.77261131	0.82587623	0.04521517	0.06966007	0	2.42809199	1.27982308	0.02801252	0.05190245	0	1.36978252	0.45394685	0.54824972	0.91919682	0
yccF	1.88853285	0.91726588	0.1208669	0.16533573	0	1.4493241	0.53538025	0.28756876	0.377082	0	0.76743388	-0.3818856	0.68730511	0.94389281	0
helD	0.71975255	-0.4744271	0.12969531	0.17632016	0	0.55022936	-0.861895	0.02687927	0.05005458	0	0.76447017	-0.3874679	0.30385021	0.80892599	0
mgsA	0.04179693	-4.5804592	1.49E-38	8.78E-37	-1	0.0696699	-3.8433207	2.87E-21	1.72E-19	-1	1.66686639	0.73713847	0.07914752	0.39708826	0
yccT	2.11563819	1.08109292	0.04109872	0.06396513	0	2.35855372	1.23790246	0.05728986	0.09655374	0	1.11481903	0.15680954	0.88008435	0.99065677	0
yccU	1.16633895	0.22198712	0.70089463	0.75160271	0	0.46205945	-1.1138496	0.00473834	0.0108547	-1	0.39616224	-1.3358367	0.00103392	0.01701339	-1
mutT	1.10524648	0.14436814	0.9556149	0.96887978	0	1.25737133	0.33041077	0.38159611	0.47287527	0	1.13763885	0.18604263	0.42846639	0.87784805	0
hspQ	0.97492457	-0.0366375	0.74993721	0.79708401	0	0.84996182	-0.2345301	0.22517142	0.30920835	0	0.87182316	-0.1978926	0.30589194	0.81005169	0
rlmI	0.60188314	-0.7324447	0.06100686	0.09033433	0	1.01637084	0.02342689	0.85360424	0.88943106	0	1.68865146	0.75587159	0.0632404	0.34388428	0
yccX	0.94791269	-0.0771739	0.78543814	0.82700364	0	0.58981027	-0.7616771	0.36877287	0.45982148	0	0.62222004	-0.6845032	0.43633511	0.88087171	0
tusE	0.52436919	-0.9313452	0.04475412	0.06899789	0	0.42804497	-1.2241657	0.02893634	0.05341257	0	0.81630458	-0.2928205	0.52273408	0.91269012	0
yccA	0.55957914	-0.8375859	0.00581153	0.0109232	0	0.28450586	-1.8134697	1.19E-06	6.91E-06	-1	0.50842829	-0.9758838	0.00439355	0.04955387	0
serT	2.30086349	1.20217539	0.41751918	0.49047399	0	4.93376662	2.30268947	0.09944605	0.15594467	0	2.14431088	1.10051408	0.34766507	0.84560166	0
hyaA	3.79465449	1.92396853	0.01961975	0.0326774	1	5.62854954	2.49276319	0.00435017	0.01005409	1	1.4832838	0.56879466	0.37723721	0.85988523	0
hyaB	6.57066695	2.71603982	2.44E-08	1.23E-07	1	6.60123683	2.72273636	2.96E-07	1.96E-06	1	1.00465248	0.00669654	0.76660801	0.96109492	0
hyaC	5.67433478	2.50445127	8.57E-07	3.50E-06	1	6.14761261	2.62002626	1.05E-05	4.91E-05	1	1.08340675	0.11557499	0.79596448	0.96646568	0
hyaD	4.18105987	2.0638687	2.23E-05	7.11E-05	1	3.42594254	1.77650096	0.00037177	0.00113615	1	0.81939572	-0.2873677	0.90416976	0.99884485	0
yacG	0.29924502	-1.7406009	0.0006888	0.00159791	-1	0.41389236	-1.2726725	0.03552591	0.06418193	0	1.38312196	0.46792838	0.45388537	0.88755478	0
hyaE	2.67813063	1.42122633	0.02285252	0.03757989	1	3.7393441	1.90278523	0.01343391	0.02706998	1	1.39625157	0.4815589	0.51217379	0.90925684	0
hyaF	4.50488622	2.17149067	0.00041954	0.00101429	1	4.90776149	2.29506514	0.00015066	0.00051696	1	1.08943073	0.12357447	0.50703286	0.90925684	0
cbdA	4.70490004	2.23416407	5.91E-08	2.82E-07	1	5.42818873	2.44047088	1.13E-07	8.24E-07	1	1.15373094	0.20630681	0.48591864	0.90075713	0
cbdB	3.12649237	1.644545	2.15E-05	6.90E-05	1	3.54945834	1.82759888	5.92E-05	0.00022491	1	1.1352845	0.18305388	0.62314342	0.92989404	0
cbdX	2.02322449	1.0166564	0.5657867	0.63108077	0	1.1413919	0.19079422	0.60405184	0.6844372	0	0.56414496	-0.8258622	0.85068782	0.98212032	0
appA	1.95797672	0.96936361	0.02592619	0.04213244	0	1.9565189	0.96828905	0.01110215	0.02304388	0	0.99925545	-0.0010746	0.58109496	0.92806272	0
etk	1.16463255	0.21987484	0.59061692	0.65349573	0	1.02886609	0.04105522	0.51255001	0.59983011	0	0.8834255	-0.1788196	0.88524278	0.99348376	0
etp	1.54248723	0.62525855	0.65986986	0.71542591	0	0.55673558	-0.8449358	0.9370257	0.95562348	0	0.36093367	-1.4701944	0.56848919	0.92771214	0
gfcE	4.05460635	2.01956185	0.00344512	0.00682687	1	3.67258978	1.87679776	0.01696213	0.03334326	1	0.90578208	-0.1427641	0.95488544	1	0
gfcD	5.01669206	2.32673638	2.69E-07	1.17E-06	1	5.24901595	2.39204698	1.97E-06	1.09E-05	1	1.04631018	0.0653106	0.71389617	0.95223057	0
